# Supplementary material for: Regioselective Ni-Catalyzed reductive alkylsilylation of acrylonitrile with unactivated alkyl bromides and chlorosilanes
Source: Nat Commun. 2022 Nov 19;13:7093. doi: 10.1038/s41467-022-34901-6 (PMC9675790; doi:10.1038/s41467-022-34901-6)
Supplement: Supplementary file 1 — Supplementary Information [file 41467_2022_34901_MOESM1_ESM.pdf]

Supplementary Information

Regioselective Ni-Catalyzed Reductive Alkylsilylation of Acrylonitrile with Unactivated Alkyl Bromides and Chlorosilanes

Jinwei Sun,[1#] Yongze Zhou,[1#] Rui Gu,[1#] Xin Li,[1] Ao Liu[1] and Xuan Zhang[1*]

[1]School of Chemistry and Materials Science, Institute of Advanced Materials and Flexible Electronics (IAMFE), Nanjing University of Information Science and Technology, 219 Ningliu Road, Nanjing 210044, China.

[#]These authors contributed equally: Jinwei Sun, Yongze Zhou, Rui Gu.

E-mail: xuanzhang@nuist.edu.cn

Table of Contents

1. Supplementary Methods……….…………………………………S2

2. Supplementary Discussion…………………………………………S3

2.1 Optimization of Reaction Conditions………………………….S3

2.2 The Investigation of Activated Olefins………………………...S7

3. Supplementary Notes………………………………………………S11

3.1 Synthesis of Substrates……………………………...………S11

3.2 Experimental Procedures and Characterization of Products.S24

3.3 Gram-scale Reactions and Synthetic Applications…….…...S60

3.4 Mechanistic Studies…………………...……………………S65

3.5 Copies of NMR Spectra…...……………………………...S68

4. Supplementary References………..………………………….....S205

1. Supplementary Methods

All reactions were carried out under an atmosphere of nitrogen in vial equipped with magnetic stirring. Anhydrous *N,N*-Dimethylacetamide (DMA), *N,N*-dimethylformamide (DMF), *N*-methylpyrrolidone (NMP), CH_3CN, EtOH, Dichloroethane (DCE), Ethyl acetate (EA), Dimethyl sulfoxide (DMSO), Tetrahydrofuran (THF) and Acetone were purchased from J&K Scientific and Adamas-beta. Toluene was dried and purified according to the procedure from "Purification of Laboratory Chemicals"[1].

Nickel catalysts, Ligands and Substrates were purchased from Adamas-beta, TCI, Strem, Alfa Aesar, Acros Organics, Energy Chemical, Bidepharm, SCRC, J&K Scientific, Macklin, or synthesized according to the procedures outlined below. Unless otherwise noted, materials obtained from commercial suppliers were used without further purification.

^{1}H NMR, ^{13}C NMR, ^{29}Si NMR, ^{19}F NMR and ^{31}P NMR spectra were collected on JEOL JNM-ECS 400M spectrometer at room temperature carried out in deuterochloroform ($CDCl_3$). ^{1}H NMR spectra were reported in parts per million (ppm) and were referenced to the signal of $CHCl_3$ (7.26 ppm). Data were reported as follows: chemical shift (ppm), multiplicity (s = singlet, d = doublet, t = triplet, q = quartet, dd = doublet of doublets, m = multiplet), coupling constant (Hz), integration and assignment. ^{13}C NMR spectra were reported in ppm relative to residual $CHCl_3$ (77.16 ppm). Coupling constants, *J*, were reported in hertz (Hz). High resolution mass spectrometry (HRMS) HRMS were recorded on Bruker microTOF-Q111, Waters Xevo G2-XS QTOF and Thermo Scientific Q Exactive (APCI) Mass Spectrometer. Contact angle of glass surface was analyzed by Contact angle were recorded on Shanghai JianDuan Photoelectricity Technology Co., Ltd. JC2000A. Melting points were uncorrected. GC spectra were performed on Shimadzu QP2010 (EI Source).

Thin layer chromatography was carried out using JIANGYOU HSGF254 TLC plates. Flash chromatography was performed using TITAN silica gel (200-300 mesh). TLC plates were analyzed by an exposure to ultraviolet (UV) light and/or submersion in $KMnO_4$ solution.

Zinc powder was activated as follows: Zn powder (100 g) and water (0.9 L) were placed into a 1-L Erlenmeyer flask equipped with a stir bar. Concd. HCl (10 mL) was added with stirring over 1 min. The slurry was stirred for 20 min and the water was decanted. The metal was washed with water (3 × 250 mL), acetone (3 × 150 mL), and Et_2O (2 × 100 mL). The metal was then transferred to a flask equipped with a vacuum take-off

and dried under full vacuum for 3 h.

2. Supplementary Discussion

2.1 Optimization of Reaction Conditions

The procedure was conducted in a nitrogen-filled glove box. To a reaction vial equipped with a magnetic stir bar was added a catalyst (10 mol%), ligand, reductant (0.6 mmol, 3.0 equiv). A solution of **1a** (39.6 mg, 0.2 mmol), **2a** (20.0 μL, 0.3 mmol) and **3a** (72.4 mg, 0.6 mmol) in the solvent (1.0 mL) was added. The reaction vial was sealed and removed from the glove box, and stirred at 25-35 ^{o}C for 24-36 h. The reaction mixture was diluted with Dichloromethane (10.0 mL), washed with brine, and dried over anhydrous Na_2SO_4. The yield was determined by ^{1}H NMR analysis with dibromomethane as an internal standard.

Supplementary Table 1: Screening of ligands.[a,b]

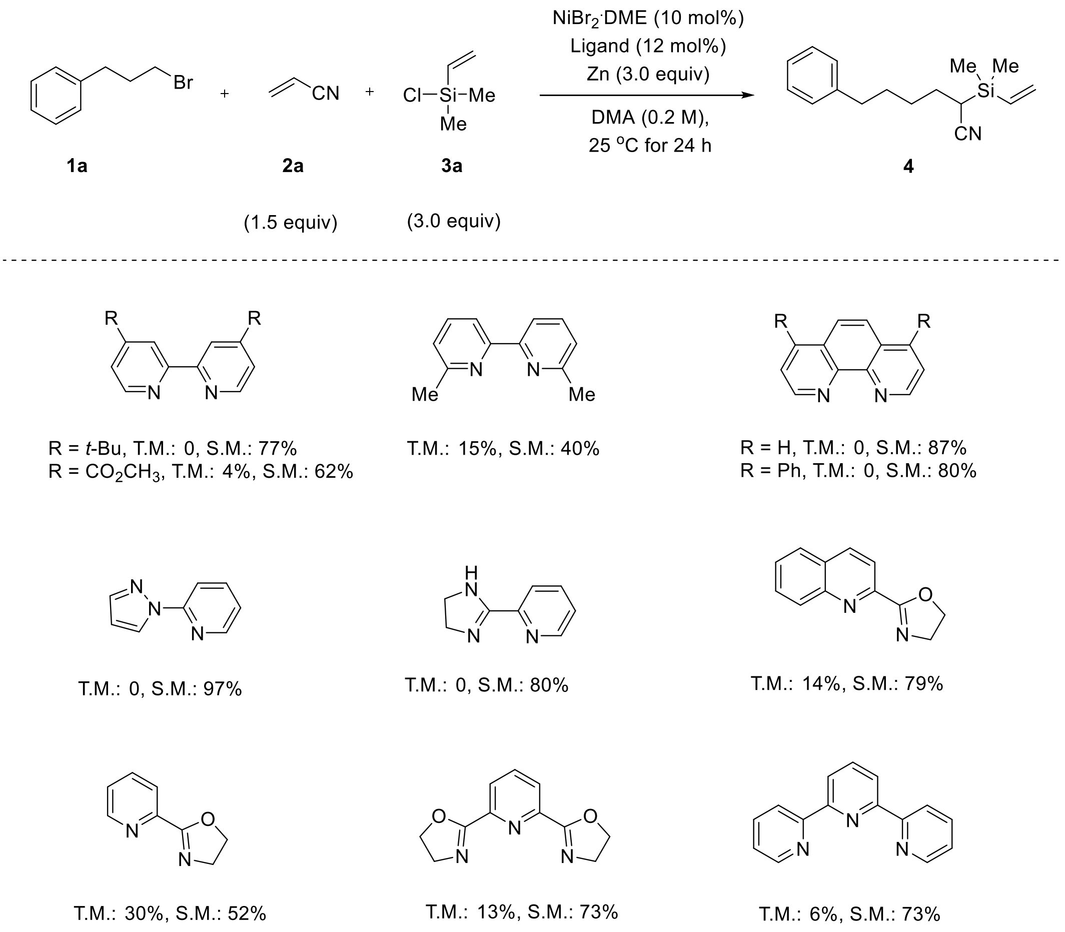

[a] All reactions were performed on a 0.2 mmol scale. [b] Yield was determined by ^{1}H NMR analysis with dibromomethane (14.0 μL, 0.2 mmol) as an internal standard.

Supplementary Table 2: Screening of nickel catalysts.[a,b]

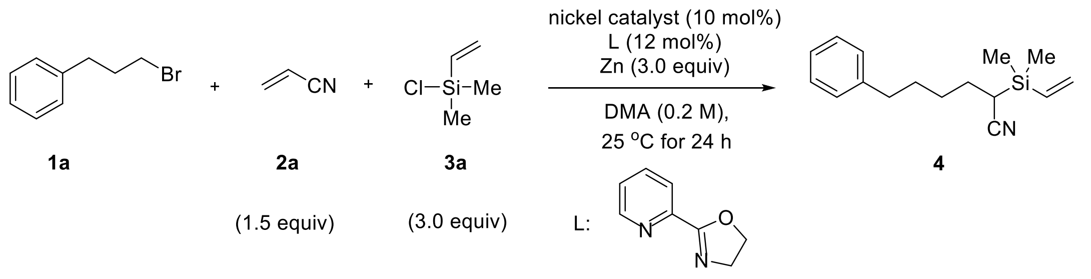

Entry	Nickel Catalyst	Yield(%)	S.M.(%)
1	$NiCl_2$	30	55
2	$NiBr_2$	12	55
3	NiI_2	42	22
4	$Ni(acac)_2$	15	83
5	$Ni(OTf)_2$	25	55
6	$Ni(COD)_2$	12	54
7	$NiBr_2$·DME	30	52
8	$NiCl_2$·DME	16	70
**9**	**$Ni(PPh)_3Cl_2$**	**35**	**47**
10	$Ni(PCy_3)_2Cl_2$	18	51
11	$Ni(dppp)Cl_2$	17	72

[a] All reactions were performed on a 0.2 mmol scale. [b] Yield was determined by ^{1}H NMR analysis with dibromomethane (14.0 μL, 0.2 mmol) as an internal standard.

Supplementary Table 3: Screening of solvents.[a,b]

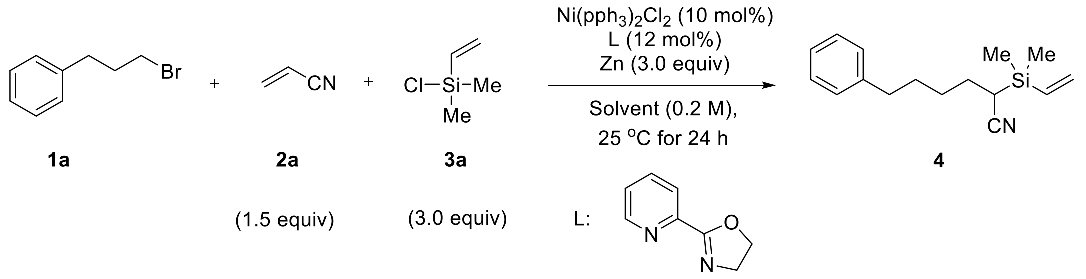

Entry	Solvent	Yield(%)	S.M.(%)
1	DMF	30	48
2	NMP	21	67
3	CH_3CN	0	94
4	EtOH	0	87
5	DCE	0	90
6	Ethyl Acetate	0	88
7	Toluene	0	89
8	DMSO	0	70
9	THF	0	85
10	Acetone	0	85
**11**	**DMA**	**35**	**47**

[a] All reactions were performed on a 0.2 mmol scale. [b] Yield was determined by ^{1}H NMR analysis with dibromomethane (14.0 μL, 0.2 mmol) as an internal standard.

Supplementary Table 4: Screening of other conditions.[a,b]

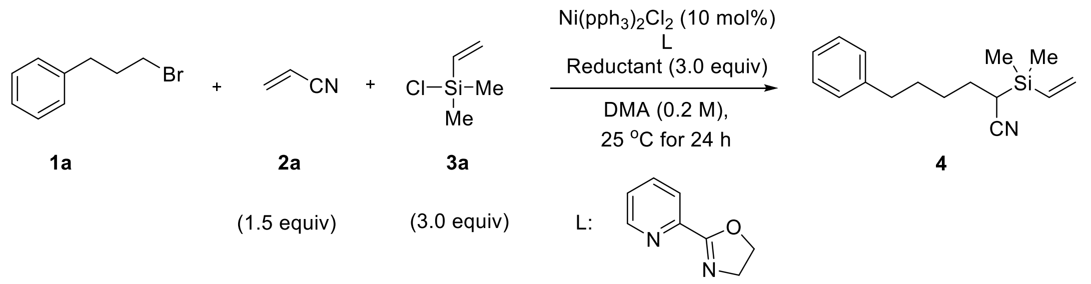

Entry	Ligand	Temperature	Time(h)	Reductant	Yield(%)	S.M.(%)
1	L (12 mol%)	25 °C	24	Zn	35	47
2	L (20 mol%)	25 °C	24	Zn	60	21
3[c]	L (20 mol%)	25 °C	24	Zn	55	63
4	L (20 mol%)	25 °C	24	Mn	4	64
5	L (20 mol%)	25 °C	24	Mg	0	3
6	L (20 mol%)	25 °C	24	TDAE[d]	Trace	72
7	L (20 mol%)	25 °C	36	Zn	65	2
8	L (20 mol%)	35 °C	24	Zn	74	7
**9**	**L (20 mol%)**	**35 °C**	**36**	**Zn**	**79**	**0**

[a] All reactions were performed on a 0.2 mmol scale. [b] Yield was determined by ^{1}H NMR analysis with dibromomethane (14.0 μL, 0.2 mmol) as an internal standard. [c] **1a : 2a** = 1.5 : 1. [d] TDAE = Tetrakis(dimethylamino)ethylene.

2.2 The Investigation of Activated Olefins

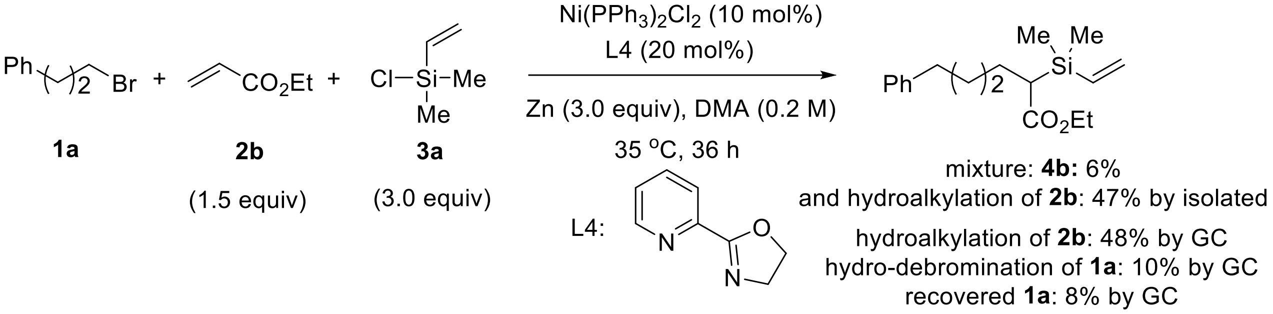

Prepared according to general procedure using (3-bromopropyl)benzene (79.2 mg, 0.4 mmol), ethyl acrylate (60.1 mg, 0.6 mmol), chlorodimethyl(vinyl)silane (144.8 mg, 1.2 mmol), $Ni(PPh_3)_2Cl_2$ (26.4 mg, 0.04 mmol), 2-(pyridin-2-yl)-4,5-dihydrooxazole (12.0 mg, 0.08 mmol), Zn (78.0 mg, 1.2 mmol), DMA (2.0 mL) at 35 °C for 36 h. In this reaction system, the mixture of **4b**: 6% and hydroalkylation of **2b**: 47% were isolated. Hydroalkylation of **2b**: 48%, hydro-debromination of **1a**: 10% and recovered **1a**: 8% were determined by GC analysis with dodecane as an internal standard.

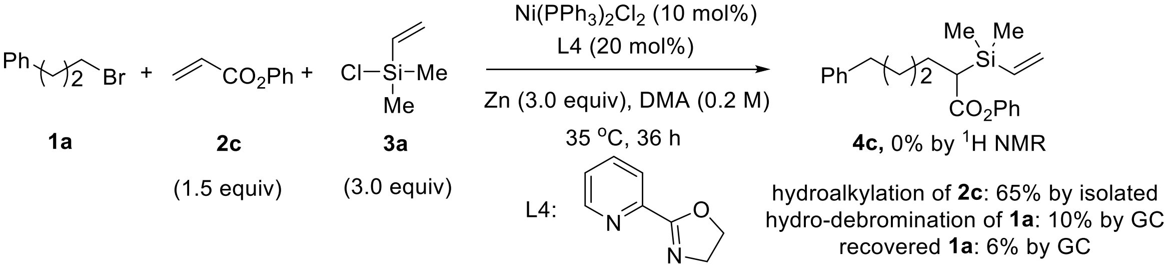

Prepared according to general procedure using (3-bromopropyl)benzene (79.2 mg, 0.4 mmol), phenyl acrylate (88.9 mg, 0.6 mmol), chlorodimethyl(vinyl)silane (144.8 mg, 1.2 mmol), $Ni(PPh_3)_2Cl_2$ (26.4 mg, 0.04 mmol), 2-(pyridin-2-yl)-4,5-dihydrooxazole (12.0 mg, 0.08 mmol), Zn (78.0 mg, 1.2 mmol), DMA (2.0 mL) at 35 °C for 36 h. In this reaction system, the yield of **4c** was determined by ^{1}H NMR analysis with dibromomethane (28.0 µL, 0.4 mmol) as an internal standard. Hydro-debromination of **1a**: 10% and recovered **1a**: 6% were determined by GC analysis with dodecane as an internal standard. Isolated hydroalkylation of **2c**: colorless oil, 69.3 mg, 65%.

Phenyl 6-phenylhexanoate

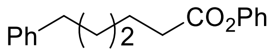

^{1}H NMR (400 MHz, $CDCl_3$) δ: 7.41 (t, J = 8.0 Hz, 2H), 7.33 (t, J = 7.6 Hz, 2H), 7.28-7.23 (m, 4H), 7.10 (d, J = 7.6 Hz, 2H), 2.70 (t, J = 7.8 Hz, 2H), 2.60 (t, J = 7.5 Hz, 2H), 1.84 (p, J = 7.6 Hz, 2H), 1.75 (p, J = 7.6 Hz, 2H), 1.51 (p, J = 7.7 Hz, 2H); ^{13}C NMR (100 MHz, $CDCl_3$) δ: 172.26, 150.84, 142.50, 129.47, 128.50, 128.40, 125.80, 121.67,

35.79, 34.38, 31.14, 28.74, 24.89; HRMS (ESI): $[M+H]^+$ calculated for $C_{18}H_{21}O_2^+$ = 269.1542, found: 269.1541.

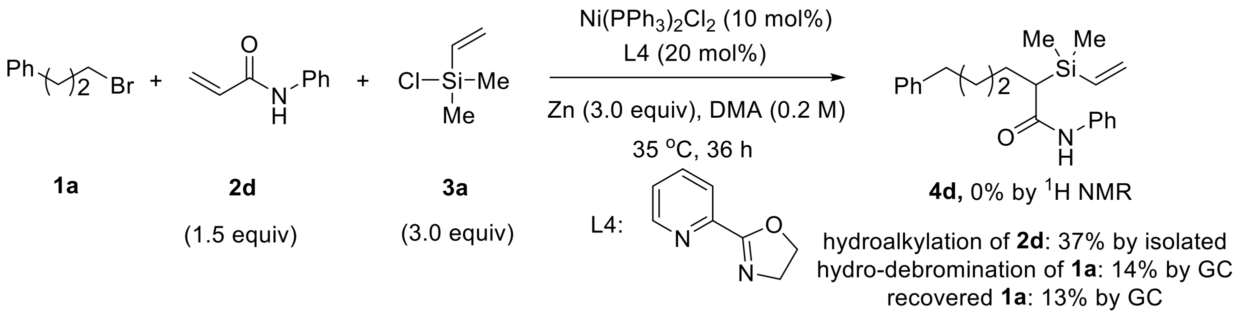

Prepared according to general procedure using (3-bromopropyl)benzene (79.2 mg, 0.4 mmol), *N*-phenylacrylamide (78.0 mg, 0.6 mmol), chlorodimethyl(vinyl)silane (144.8 mg, 1.2 mmol), $Ni(PPh_3)_2Cl_2$ (26.4 mg, 0.04 mmol), 2-(pyridin-2-yl)-4,5-dihydrooxazole (12.0 mg, 0.08 mmol), Zn (78.0 mg, 1.2 mmol), DMA (2.0 mL) at 35 °C for 36 h. In this reaction system, the yield of **4d** was determined by ^1H NMR analysis with dibromomethane (28.0 μL, 0.4 mmol) as an internal standard. Hydro-debromination of **1a**: 14% and recovered **1a**: 13% were determined by GC analysis with dodecane as an internal standard. Isolated hydroalkylation of **2d**: colorless oil, 39.5 mg, 37%.

***N*,6-diphenylhexanamide**

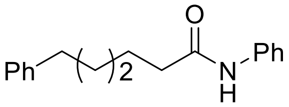

^1H NMR (400 MHz, $CDCl_3$) δ: 7.50 (d, *J* = 8.0 Hz, 2H), 7.35-7.24 (m, 4H), 7.20-7.16 (m, 3H), 7.10 (t, *J* = 7.5 Hz, 1H), 2.62 (t, *J* = 7.6 Hz, 2H), 2.34 (t, *J* = 7.5 Hz, 2H), 1.76 (p, *J* = 7.5 Hz, 2H), 1.67 (p, *J* = 7.6 Hz, 2H), 1.42 (p, *J* = 7.9 Hz, 2H); ^{13}C NMR (100 MHz, $CDCl_3$) δ: 171.61, 142.58, 138.10, 129.05, 128.50, 128.39, 125.79, 124.28, 120.00, 37.72, 35.82, 31.26, 28.94, 25.57; HRMS (ESI): $[M+H]^+$ calculated for $C_{18}H_{22}NO^+$ = 268.1701, found: 268.1701.

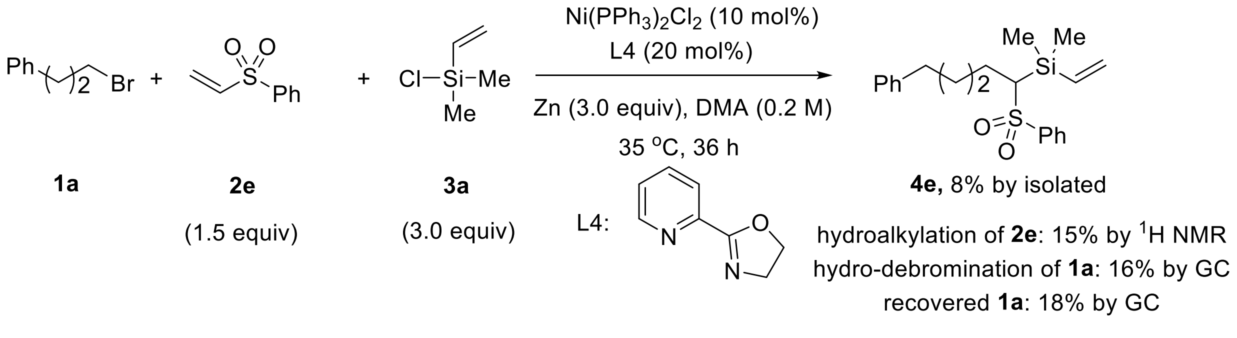

Prepared according to general procedure using (3-bromopropyl)benzene (79.2 mg, 0.4 mmol), (vinylsulfonyl)benzene (100.9 mg, 0.6 mmol), chlorodimethyl(vinyl)silane (144.8 mg, 1.2 mmol), $Ni(PPh_3)_2Cl_2$ (26.4 mg, 0.04 mmol), 2-(pyridin-2-yl)-4,5-dihydrooxazole (12.0 mg, 0.08 mmol), Zn (78.0 mg, 1.2 mmol), DMA (2.0 mL) at 35 °C for 36 h. The crude material was purified by flash chromatography (silica gel: 5% EtOAc in Petroleum ether) to provide the title compound as a pale yellow oil (11.2 mg, 8% yield). ^{1}H NMR (400 MHz, $CDCl_3$) δ: 7.85 (d, J = 7.3 Hz, 2H), 7.60-7.48 (m, 3H), 7.26-7.14 (m, 3H), 7.0 (d, J = 7.0 Hz, 2H), 6.23 (dd, J_1 = 20.2 Hz, J_2 = 14.7 Hz, 1H), 6.05 (dd, J_1 = 14.7 Hz, J_2 = 3.5 Hz, 1H), 5.77 (dd, J_1 = 20.2 Hz, J_2 = 3.5 Hz, 1H), 2.60 (t, J = 5.3 Hz, 1H), 2.40-2.32 (m, 2H), 1.69-1.64 (m, 2H), 1.36-1.28 (m, 2H), 1.19-1.11 (m, 1H), 0.93-0.84 (m, 1H), 0.39 (s, 3H), 0.38 (s, 3H); ^{13}C NMR (100 MHz, $CDCl_3$) δ: 142.08, 141.25, 136.42, 133.91, 133.10, 129.10, 128.40, 128.09, 125.89, 56.11, 35.36, 30.99, 28.59, 26.58, -2.29, -3.34; ^{29}Si NMR (79 MHz, $CDCl_3$) δ: -2.95; HRMS (ESI): $[M+H]^+$ calculated for $C_{21}H_{29}O_2SSi^+$ = 373.1658, found: 373.1658. In this reaction system, the isolated yield of **4e**: 8%. Hydroalkylation of **2e**: 15% was determined by ^{1}H NMR analysis with dibromomethane (28.0 μL, 0.4 mmol) as an internal standard. Hydro-debromination of **1a**: 16% and recovered **1a**: 18% were determined by GC analysis with dodecane as an internal standard.

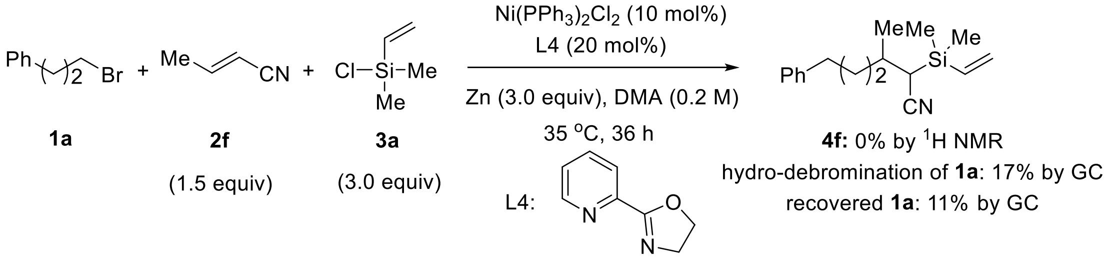

Prepared according to general procedure using (3-bromopropyl)benzene (79.2 mg, 0.4 mmol), (*E*)-but-2-enenitrile (40.2 mg, 0.6 mmol), chlorodimethyl(vinyl)silane (144.8 mg, 1.2 mmol), $Ni(PPh_3)_2Cl_2$ (26.4 mg, 0.04 mmol), 2-(pyridin-2-yl)-4,5-dihydrooxazole (12.0 mg, 0.08 mmol), Zn (78.0 mg, 1.2 mmol), DMA (2.0 mL) at 35 °C for 36 h. In this reaction system, the yield of **4f** was determined by ^{1}H NMR analysis with dibromomethane (28.0 μL, 0.4 mmol) as an internal standard. Hydro-debromination of **1a**: 17% and recovered **1a**: 11% were determined by GC analysis with dodecane as an internal standard.

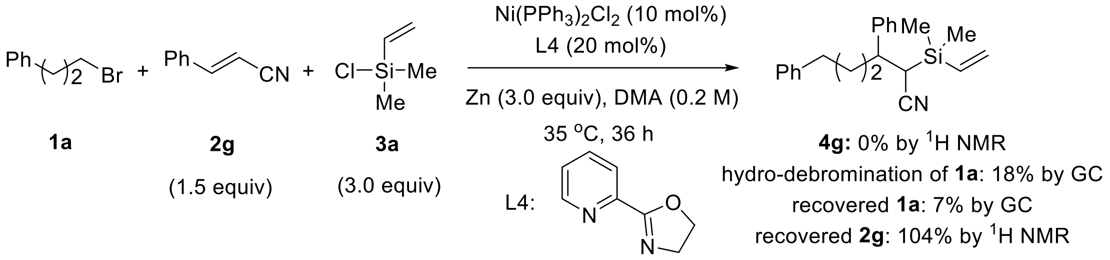

Prepared according to general procedure using (3-bromopropyl)benzene (79.2 mg, 0.4 mmol), cinnamonitrile (77.4 mg, 0.6 mmol), chlorodimethyl(vinyl)silane (144.8 mg, 1.2 mmol), $Ni(PPh_3)_2Cl_2$ (26.4 mg, 0.04 mmol), 2-(pyridin-2-yl)-4,5-dihydrooxazole (12.0 mg, 0.08 mmol), Zn (78.0 mg, 1.2 mmol), DMA (2.0 mL) at 35 °C for 36 h. In this reaction system, the yield of **4g** and recovered of **2g**: 104% were determined by ^{1}H NMR analysis with dibromomethane (28.0 μL, 0.4 mmol) as an internal standard. Hydro-debromination of **1a**: 18% and recovered **1a**: 7% were determined by GC analysis with dodecane as an internal standard.

3. Supplementary Notes

3.1 Synthesis of Substrates

3.1.1 Synthesis of alkyl bromides

The alkyl bromides used in this paper:

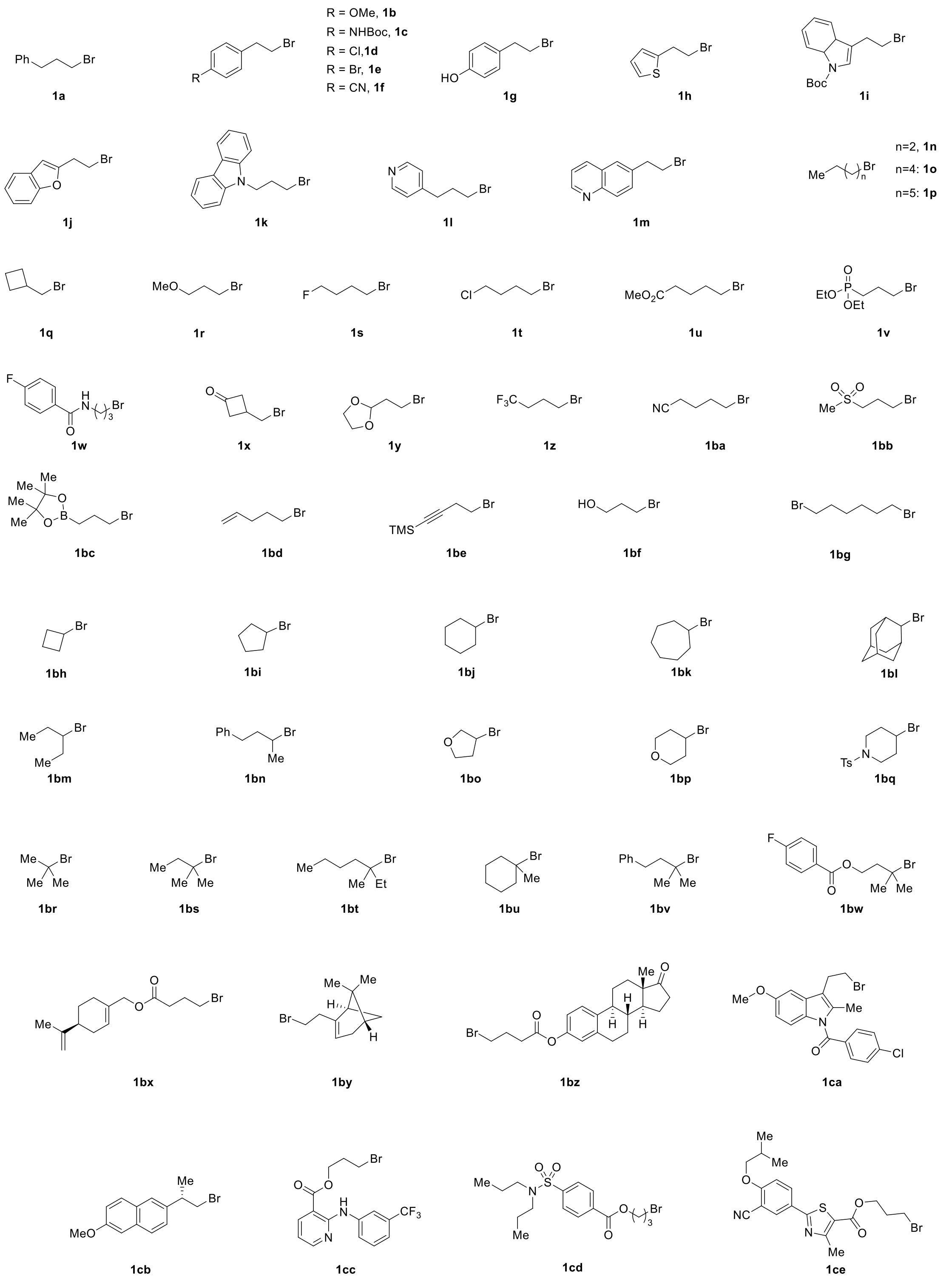

Alkyl bromides **1a, 1e-h, 1n-v, 1x-z, 1ba-bm, 1bo-bp, 1br-bs** and **1bu** are commercially available. **1b**[2], **1c**[3], **1i**[4], **1j**[5], **1k**[6], **1l**[7], **1m**[8], **1bn**[9], **1bq**[10], **1bt**[11], **1bu**[12], **1bv**[13],

1bw[14], **1by**[15], **1ca**[16], **1cd**[17], **1ce**[18] are known compounds, and they were synthesized according to the literature procedure.

The preparation of **1w, 1bx, 1bz, 1cb** and **1cc**, and their characterization data are provided as follows.

1-(2-bromoethyl)-4-methoxybenzene (1b)

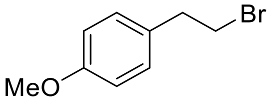

Cooled the solution of 2-(4-methoxyphenyl)ethan-1-ol (1.218 g, 8.0 mmol) in CH_2Cl_2 (60.0 mL) to 0 °C, added CBr_4 (3.980 g, 12.0 mmol) to the resulting solution followed by portion-wise addition of PPh_3 (3.148 g, 12.0 mmol), the reaction mixture stirred for 30 minutes at 0 °C and warmed to room temperature and stirred for 2 h. Removed half of the solvent in vacuo, added hexane to the mixture, filtered the resulting precipitate through Celite, purified by flash chromatography (silica gel: 5% EtOAc in Petroleum ether) to provide the title compound as a colorless oil (1.547 g, 90% yield). ^{1}H NMR (400 MHz, $CDCl_3$) δ: 7.15 (d, J = 8.3 Hz, 2H), 6.88 (d, J = 8.7 Hz, 2H), 3.81 (s, 3H), 3.55 (t, J = 7.7 Hz, 2H), 3.12 (t, J = 7.6 Hz, 2H). All data are in accordance with the literature[2].

Tert-butyl (4-(2-bromoethyl)phenyl)carbamate (1c)

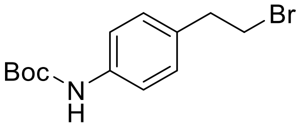

Cooled the solution of (4-(2-hydroxyethyl)phenyl)carbamate (0.948 g, 4.0 mmol) in CH_2Cl_2 (30.0 mL) to 0 °C, added CBr_4 (1.986 g, 6.0 mmol) to the resulting solution followed by portion-wise addition of PPh_3 (1.572 g, 6.0 mmol), the reaction mixture stirred for 30 minutes at 0 °C and warmed to room temperature and stirred for overnight. Removed half of the solvent in vacuo, added hexane to the mixture, filtered the resulting precipitate through Celite, purified by flash chromatography (silica gel: 20% EtOAc in Petroleum ether) to provide the title compound as a white solid (0.584 g, 49% yield), mp 95-96 °C. ^{1}H NMR (400 MHz, $CDCl_3$) δ: 7.31 (d, J = 8.3 Hz, 2H), 7.13 (d, J = 8.3 Hz, 2H), 6.46 (s, 1H), 3.52 (t, J = 7.7 Hz, 2H), 3.10 (t, J = 7.6 Hz, 2H), 1.51 (s, 9H). All data are in accordance with the literature[3].

Tert-butyl 3-(2-bromoethyl)-1*H*-indole-1-carboxylate (1i)

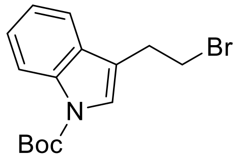

To a solution of 3-(2-bromoethyl)-1*H*-indole (1.0 g, 4.4 mmol), Et_3N (0.880 g, 8.8 mmol) and DMAP (0.120 g, 0.88 mmol) in CH_2Cl_2 (10.0 mL) at 0 °C, added $(Boc)_2O$ (1.030 g, 4.67 mmol) in CH_2Cl_2 (6.0 mL) over 30 minutes. After stirring for 1 h at room temperature, the reaction mixture was washed three times with water, dried over sodium sulfate and concentrated, purified by flash chromatography (silica gel: 10% EtOAc in Petroleum ether) to provide the title compound as a white solid (1.318 g, 91% yield), mp 71-73 °C. ^{1}H NMR (400 MHz, $CDCl_3$) δ: 8.14 (s, 1H), 7.54-7.44 (m, 2H), 7.36-7.29 (m, 1H), 7.26-7.21 (m, 1H), 3.63 (t, *J* = 7.6 Hz, 2H), 3.27 (t, *J* = 7.4 Hz, 2H), 1.66 (s, 9H). All data are in accordance with the literature[4].

2-(2-bromoethyl)benzofuran (1j)

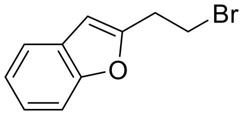

Cooled the solution of 2-(benzofuran-2-yl)ethan-1-ol (0.324 g, 2.0 mmol) in CH_2Cl_2 (15.0 mL) to 0 °C, added CBr_4 (1.016 g, 3.0 mmol) to the resulting solution followed by portion-wise addition of PPh_3 (0.787 g, 3.0 mmol), the reaction mixture stirred for 30 minutes at 0 °C and warmed to room temperature and stirred for 2 h. Removed half of the solvent in vacuo, added hexane to the mixture, filtered the resulting precipitate through Celite, purified by flash chromatography (silica gel: 20% EtOAc in Petroleum ether) to provide the title compound as a colorless oil (0.289 g, 65% yield). ^{1}H NMR (400 MHz, $CDCl_3$) δ: 7.65-7.51 (m, 2H), 7.39-7.28 (m, 2H), 6.61 (s, 1H), 3.75 (t, *J* = 7.2 Hz, 2H), 3.39 (t, *J* = 7.2 Hz, 2H). All data are in accordance with the literature[5].

9-(3-bromopropyl)-9H-carbazole (1k)

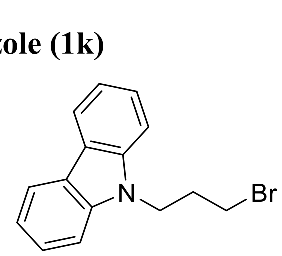

The 9*H*-carbazole (3.340 g, 20.0 mmol), 1,3-dibromopropane (6.1 mL, 60.0 mmol), NaOH (10.0 g, 25.0 mmol), tetrabutylammonium iodide (0.307 g, 0.83 mmol) were dissolved in H_2O (10.0 mL), The mixture was stirred at room temperature for 6 h and quenched by addition of ice, the crude product was diluted with CH_2Cl_2 and washed

with brine. The organic phase was dried anhydrous sodium sulfate and filtered, purified by flash chromatography (silica gel: 25% EtOAc in Petroleum ether) to provide the title compound as a white solid (1.830 g, 32% yield), mp 52-53 °C; ^{1}H NMR (400 MHz, $CDCl_3$) δ: 8.13 (d, J = 7.2 Hz, 2H), 7.50 (d, J = 3.6 Hz, 4H), 7.30-7.26 (m, 2H), 4.51 (t, J = 6.5 Hz, 2H), 3.40 (t, J = 6.1 Hz, 2H), 2.46 (q, J = 6.3 Hz, 2H). All data are in accordance with the literature[6].

4-(3-bromopropyl)pyridine (1l)

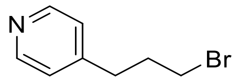

Cooled the solution of 3-(pyridin-4-yl)propan-1-ol (0.990 g, 7.0 mmol) in THF (10.0 mL) to 0 °C, added CBr_4 (3.553 g, 10.5 mmol) to the resulting solution followed by portion-wise addition of PPh_3 (2.754 g, 10.5 mmol), the reaction mixture stirred for 30 minutes at 0 °C and warmed to room temperature and stirred for overnight. Removed half of the solvent in vacuo, added hexane to the mixture, filtered the resulting precipitate through Celite, purified by flash chromatography (silica gel: 50% EtOAc and 3% Et_3N in Petroleum ether) to provide the title compound as a colorless oil (0.783 g, 56% yield). ^{1}H NMR (400 MHz, $CDCl_3$) δ: 8.52 (d, J = 6.0 Hz, 2H), 7.14 (d, J = 8.0 Hz, 2H), 3.40 (t, J = 6.4 Hz, 2H), 2.79 (t, J = 7.3 Hz, 2H), 2.22-2.15 (m, 2H). All data are in accordance with the literature[7].

6-(2-bromoethyl)quinoline (1m)

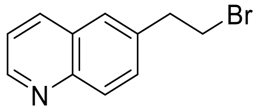

Cooled the solution of 2-(quinolin-6-yl)ethan-1-ol (0.519 g, 3.0 mmol) in THF (4.5 mL) to 0 °C, added CBr_4 (1.490 g, 4.5 mmol) to the resulting solution followed by portion-wise addition of PPh_3 (1.180 g, 4.5 mmol), the reaction mixture stirred for 30 minutes at 0 °C and warmed to room temperature and stirred for overnight. Removed half of the solvent in vacuo, added hexane to the mixture, filtered the resulting precipitate through Celite, purified by flash chromatography (silica gel: 50% EtOAc and 3% Et_3N in Petroleum ether) to provide the title compound as a yellow liquid (0.456 g, 63% yield). ^{1}H NMR (400 MHz, $CDCl_3$) δ: 8.90 (dd, J_1 = 4.2 Hz, J_2 = 1.7 Hz, 1H), 8.13 (d, J = 8.3 Hz, 1H), 8.07 (d, J = 8.6 Hz, 1H), 7.66 (s, 1H), 7.58 (dd, J_1 = 8.6 Hz, J_2 = 2.0 Hz, 1H), 7.40 (dd, J_1 = 8.3 Hz, J_2 = 4.2 Hz, 1H), 3.68 (t, J = 7.4 Hz, 2H), 3.36 (t, J = 7.4 Hz, 2H). All data are in accordance with the literature[8].

N-(3-bromopropyl)-4-fluorobenzamide (1w)

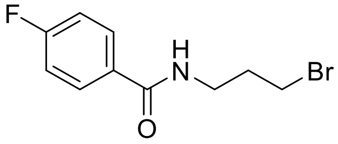

Cooled the solution of 3-Bromopropylamine hydrobromide (1.751 g, 8.0 mmol), Triethylamine (2.429 g, 24.0 mmol) in CH_2Cl_2 (16.0 mL) to 0 °C and added 4-Fluorobenzoyl chloride (1.522 g, 9.6 mmol), the reaction mixture warmed to room temperature and stirred for overnight, washed the mixture with brine and separated the organic layer, dried the resultant over anhydrous sodium sulfate, purified by flash chromatography (silica gel: 20% EtOAc in Petroleum ether) to provide the title compound as a white solid (0.972 g, 47% yield), mp 94-95 °C. ^1H NMR (400 MHz, $CDCl_3$) δ: 7.78 (dd, J_1 = 8.8 Hz, J_2 = 5.3 Hz, 2H), 7.12 (t, J = 8.6 Hz, 2H), 6.28 (s, 1H), 3.63 (q, J = 6.4 Hz, 2H), 3.51 (t, J = 6.4 Hz, 2H), 2.25-2.18 (m, 2H); ^{13}C NMR (100 MHz, $CDCl_3$) δ: 166.99, 163.49 (d, J = 250.6 Hz), 130.57 (d, J = 3.1 Hz), 129.42 (d, J = 6.6 Hz), 115.58 (d, J = 21.7 Hz), 38.80, 32.22, 31.11; ^{19}F NMR (376 MHz, $CDCl_3$) δ: -106.27; HRMS (ESI): $[M+H]^+$ calculated for $C_{10}H_{12}BrFNO^+$ = 260.0086, found: 260.0085.

(3-bromobutyl)benzene (1bn)

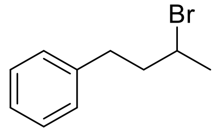

Added CBr_4 (2.710 g, 10.0 mmol) to a solution of 4-phenylbutan-2-ol (0.767 g, 5.0 mmol) and pyridine (0.400 g, 5.0 mmol) in toluene (10.0 mL) at 0 °C, the reaction mixture warmed to room temperature, then heated to 120 °C for 3 h. Washed the mixture with brine and separated the organic layer, dried the resultant over anhydrous sodium sulfate, purified by flash chromatography (silica gel: 5% EtOAc in Petroleum ether) to provide the title compound as a colorless oil (0.490 g, 46% yield). ^1H NMR (400 MHz, $CDCl_3$) δ: 7.39-7.35 (m, 2H), 7.30-7.25 (m, 3H), 4.19-4.11 (m, 1H), 2.98-2.91 (m, 1H), 2.87-2.79 (m, 1H), 2.27-2.18 (m, 1H), 2.17-2.08 (m, 1H), 1.81 (d, J = 6.7 Hz, 3H). All data are in accordance with the literature[9].

4-bromo-1-tosylpiperidine (1bq)

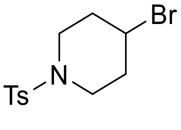

Added Et_3N (0.6 mL) to a solution of 4-bromopiperidine (0.490 g, 2.0 mmol) and *p*-

toluenesulfonyl chloride (0.420 g, 2.2 mmol) in CH_2Cl_2 (20.0 mL) at 0 °C, the reaction mixture warmed to room temperature and stirred for overnight. Diluted the reaction mixture with water and brine, dried the resultant over anhydrous magnesium chloride, purified by flash chromatography (silica gel: 50% DCM in Petroleum ether) to provide the title compound as a white solid (0.341 g, 54% yield), mp 134-136 °C. ^1H NMR (400 MHz, $CDCl_3$) δ: 7.65 (d, J = 8.3 Hz, 2H), 7.34 (d, J = 8.0 Hz, 2H), 4.25 (dt, J_1 = 6.7 Hz, J_2 = 3.2 Hz, 1H), 3.23-3.17 (m, 2H), 3.13-3.07 (m, 2H), 2.45 (s, 3H), 2.23-2.15 (m, 2H), 2.09-2.01 (m, 2H). All data are in accordance with the literature[10].

3-bromo-3-methylheptane (1bt)

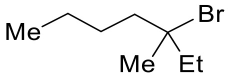

Added LiBr (0.274 g, 3.2 mmol) in 48 wt% aqueous HBr (2.1 mL) to a solution of 3-methylheptan-3-ol (0.273 g, 2.1 mmol) at 0 °C, the reaction mixture warmed to room temperature and stirred for overnight. Diluted the mixture with Et_2O, washed with water, saturated $NaHCO_3$ and brine. Dried over anhydrous magnesium chloride and concentrated to provide the title compound as a colorless oil (0.152 g, 37% yield). ^1H NMR (400 MHz, $CDCl_3$) δ: 1.92-1.75 (m, 4H), 1.70 (s, 3H), 1.49-1.40 (m, 2H), 1.34 (q, J = 8.0 Hz, 2H), 1.03 (t, J = 7.3 Hz, 3H), 0.93 (t, J = 7.2 Hz, 3H). All data are in accordance with the literature[11].

1-bromo-1-methylcyclohexane (1bu)

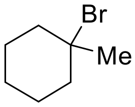

Added LiBr (1.302 g, 15.0 mmol) in 48 wt% aqueous HBr (10.0 mL) to a solution of 1-methylcyclohexan-1-ol (1.141g, 10.0 mmol) at 0 °C, the reaction mixture warmed to room temperature and stirred for overnight. Diluted the mixture with Et_2O, washed with water, saturated $NaHCO_3$ and brine. Dried over anhydrous magnesium chloride and concentrated to provide the title compound as a yellow liquid (0.740 g, 42% yield). ^1H NMR (400 MHz, $CDCl_3$) δ: 2.09 (dt, J_1 = 14.3 Hz, J_2 = 3.4 Hz, 2H), 1.83 (s, 3H), 1.76-1.57 (m, 5H), 1.50-1.43 (m, 2H), 1.26-1.17 (m, 1H). All data are in accordance with the literature[12].

(3-bromo-3-methylbutyl)benzene (1bv)

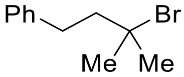

Added LiBr (1.300 g, 15.0 mmol) in 48 wt% aqueous HBr (10.0 mL) to a solution of

2-methyl-4-phenylbutan-2-ol (1.650 g, 10.0 mmol) at 0 ºC, the reaction mixture warmed to room temperature and stirred for overnight. Diluted the mixture with Et_2O, washed with water, saturated $NaHCO_3$ and brine. Dried over anhydrous magnesium chloride and concentrated to provide the title compound as a yellow oil (1.951 g, 86% yield). ^{1}H NMR (400 MHz, $CDCl_3$) δ: 7.32-7.28 (m, 2H), 7.23-7.19 (m, 3H), 2.88-2.84 (m, 2H), 2.11-2.07 (m, 2H), 1.83 (s, 6H). All data are in accordance with the literature[13].

3-bromo-3-methylbutyl 4-fluorobenzoate (1bw)

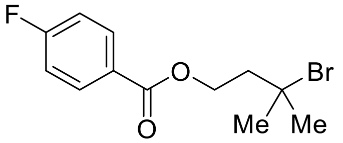

Added LiBr (1.106 g, 12.7 mmol) in 48 wt% aqueous HBr (8.5 mL) to a solution of 3-hydroxy-3-methylbutyl 4-fluorobenzoate (1.920 g, 8.49 mmol) at 0 ºC, the reaction mixture warmed to room temperature and stirred for overnight. Diluted the mixture with Et_2O, washed with water, saturated $NaHCO_3$ and brine. Dried over anhydrous magnesium chloride and concentrated to provide the title compound as a yellow liquid (2.210 g, 90% yield). ^{1}H NMR (400 MHz, $CDCl_3$) δ: 8.05 (dd, J_1 = 9.0 Hz, J_2 = 5.4 Hz, 2H), 7.11 (t, J = 8.7 Hz, 2H), 4.58 (t, J = 6.7 Hz, 2H), 2.30 (t, J = 6.8 Hz, 2H), 1.85 (s, 6H). All data are in accordance with the literature[14].

(*S*)-(4-(prop-1-en-2-yl)cyclohex-1-en-1-yl)methyl 4-bromobutanoate (1bx)

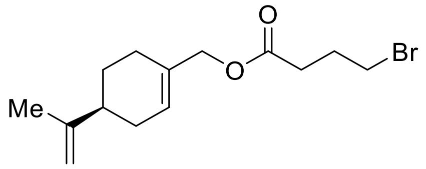

Cooled the solution of 4-Bromobutyryl chloride (1.854 g, 10.0 mmol), Triethylamine (1.39 mL, 10.0 mmol) in CH_2Cl_2 (15.0 mL) to 0 ºC and added (*S*)-(-)-Perillyl alcohol (0.85 mL, 5.4 mmol), the reaction mixture warmed to room temperature and stirred for overnight, washed the mixture with brine and separated the organic layer, dried the resultant over anhydrous sodium sulfate, purified by flash chromatography (silica gel: 25% DCM in Petroleum ether) to provide the title compound as a colorless oil (0.858 g, 53% yield). ^{1}H NMR (400 MHz, $CDCl_3$) δ: 5.76 (s, 1H), 4.72 (d, J = 8.1 Hz, 2H), 4.48 (s, 2H), 3.47 (t, J = 6.5 Hz, 2H), 2.53 (t, J = 7.2 Hz, 2H), 2.22-2.13 (m, 4H), 2.10-2.07 (m, 2H), 2.01-1.84 (m, 2H), 1.74 (s, 3H), 1.54-1.44 (m, 1H); ^{13}C NMR (100 MHz, $CDCl_3$) δ: 172.45, 149.53, 132.55, 126.03, 108.90, 68.65, 40.84, 32.78, 32.53, 30.50, 27.85, 27.34, 26.45, 20.81; HRMS (ESI): $[M+Na]^+$ calculated for $C_{14}H_{21}BrNaO_2^+$ =

323.0623, found: 323.0623.

(1*R*,5*S*)-2-(2-bromoethyl)-6,6-dimethylbicyclo[3.1.1]hept-2-ene (1by)

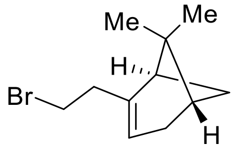

Cooled the solution of 2-((1*R*,5*S*)-6,6-dimethylbicyclo[3.1.1]hept-2-en-2-yl)ethan-1-ol (0.831 g, 5.0 mmol) in CH_2Cl_2 (35.0 mL) to 0 °C, added CBr_4 (2.538 g, 7.5 mmol) to the resulting solution followed by portion-wise addition of PPh_3 (1.967 g, 7.5 mmol), the reaction mixture stirred for 30 minutes at 0 °C and warmed to room temperature and stirred for 2 h. Removed half of the solvent in vacuo, added hexane to the mixture, filtered the resulting precipitate through Celite, purified by flash chromatography (silica gel: 5% EtOAc in Petroleum ether) to provide the title compound as a colorless oil (0.967 g, 85% yield). ^{1}H NMR (400 MHz, $CDCl_3$) δ: 5.32 (dt, J_1 = 2.9 Hz, J_2 = 1.4 Hz, 1H), 3.36 (dt, J_1 = 7.7 Hz, J_2 = 3.8 Hz, 2H), 2.52 (t, J = 7.5 Hz, 2H), 2.37 (dt, J_1 = 8.6 Hz, J_2 = 5.6 Hz, 1H), 2.29-2.17 (m, 2H), 2.10-2.06 (m, 1H), 2.01 (t, J = 8.0 Hz, 1H), 1.27 (s, 3H), 1.17 (d, J = 8.6 Hz, 1H), 0.84 (s, 3H). All data are in accordance with the literature[15].

(8*R*,9*S*,13*S*,14*S*)-13-methyl-17-oxo-7,8,9,11,12,13,14,15,16,17-decahydro-6*H*-cyclopenta[*a*]phenanthren-3-yl 4-bromobutanoate (1bz)

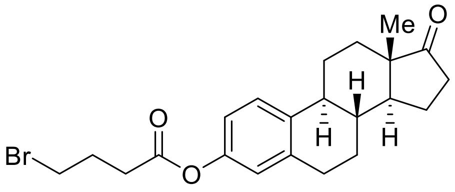

Cooled the solution of Estrone (0.811 g, 3.0 mmol), Triethylamine (0.77 mL, 5.50 mmol) in CH_2Cl_2 (7.5 mL) to 0 °C and added 4-Bromobutyryl chloride (0.62 mL, 5.50 mmol), the reaction mixture warmed to room temperature and stirred for overnight, washed the mixture with $NaHCO_3$ and brine, separated the organic layer, dried the resultant over anhydrous sodium sulfate, purified by flash chromatography (silica gel: 10% EtOAc in Petroleum ether) to provide the title compound as an orange solid (1.056 g, 84% yield), mp 116-117 °C. ^{1}H NMR (400 MHz, $CDCl_3$) δ: 7.28 (d, J = 8.5 Hz, 1H), 6.87-6.87 (m, 2H), 3.54 (t, J = 6.4 Hz, 2H), 2.91-2.89 (m, 2H), 2.75 (t, J = 7.2 Hz, 2H), 2.51 (dd, J_1 = 8.8 Hz, J_2 = 5.3 Hz, 1H), 2.43-2.38 (m, 1H), 2.28 (p, J = 6.8 Hz, 3H), 2.17-1.96 (m, 4H), 1.63-1.42 (m, 6H), 0.91 (s, 3H); ^{13}C NMR (100 MHz, $CDCl_3$) δ: 220.88, 171.49, 148.53, 138.17, 137.60, 126.54, 121.59, 118.77, 50.53, 48.05, 44.25, 38.10, 35.96,

32.65, 31.65, 29.50, 27.76, 26.43, 25.86, 21.69, 13.94; HRMS (ESI): $[M+H]^+$ calculated for $C_{22}H_{28}BrO_3^+$ = 419.1222, found: 419.1222.

(3-(2-bromoethyl)-5-methoxy-2-methyl-1*H*-indol-1-yl)(4-chlorophenyl)methanone (1ca)

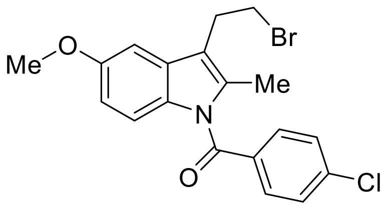

Cooled the solution of (4-chlorophenyl)(3-(2-hydroxyethyl)-5-methoxy-2-methyl-1*H*-indol-1-yl)methanone (0.686 g, 2.0 mmol) in CH_2Cl_2 (15.0 mL) to 0 °C, added CBr_4 (1.048 g, 4.0 mmol) to the resulting solution followed by portion-wise addition of PPh_3 (0.918 g, 4.0 mmol), the reaction mixture stirred for 30 minutes at 0 °C and warmed to room temperature and stirred for overnight. Removed half of the solvent in vacuo, added hexane to the mixture, filtered the resulting precipitate through Celite, purified by flash chromatography (silica gel: 10% EtOAc in Petroleum ether) to provide the title compound as a white solid (0.688 g, 85% yield), mp 92-93 °C. ^1H NMR (400 MHz, $CDCl_3$) δ: 7.65 (d, *J* = 7.3 Hz, 2H), 7.47 (d, *J* = 7.3 Hz, 2H), 6.89 (d, *J* = 7.9 Hz, 2H), 6.68 (d, *J* = 9.4 Hz, 1H), 3.85 (s, 3H), 3.57 (d, *J* = 7.6 Hz, 2H), 3.24 (d, *J* = 7.6 Hz, 2H), 3.26 (s, 3H). All data are in accordance with the literature[16].

(*S*)-2-(1-bromopropan-2-yl)-6-methoxynaphthalene (1cb)

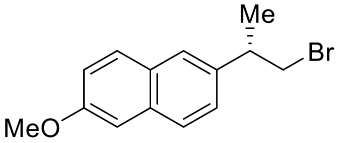

Cooled the solution of (*S*)-2-(6-methoxynaphthalen-2-yl)propan-1-ol (0.498 g, 2.3 mmol) to 0 °C, added CBr_4 (1.184 g, 3.5 mmol) to the resulting solution followed by portion-wise addition of PPh_3 (0.918 g, 3.5 mmol), the reaction mixture stirred for 30 minutes at 0 °C and warmed to room temperature and stirred for overnight. Removed half of the solvent in vacuo, added hexane to the mixture, filtered the resulting precipitate through Celite, purified by flash chromatography (silica gel: 10% EtOAc in Petroleum ether) to provide the title compound as a yellow solid (0.570 g, 89% yield), mp 60-62 °C. ^1H NMR (400 MHz, $CDCl_3$) δ: 7.71 (d, *J* = 8.7 Hz, 2H), 7.59 (s, 1H), 7.31 (d, *J* = 8.5 Hz, 1H), 7.16-7.12 (m, 2H), 3.92 (s, 3H), 3.68-3.64 (m, 1H), 3.57-3.53 (m, 1H), 3.27 (q, *J* = 7.1 Hz, 1H), 1.50 (d, *J* = 6.9 Hz, 3H); ^{13}C NMR (100 MHz, $CDCl_3$) δ: 157.69, 138.92, 133.78, 129.33, 129.08, 127.25, 125.98, 125.58, 119.11, 105.75,

55.43, 42.26, 40.16, 20.11; HRMS (ESI): $[M+H]^+$ calculated for $C_{14}H_{16}BrO^+$ = 279.0385, found: 279.0384.

3-bromopropyl 2-((3-(trifluoromethyl)phenyl)amino)nicotinate (1cc)

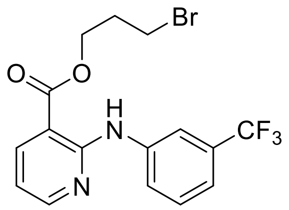

Mixed Niflumic acid (0.847 g, 3.0 mmol) and potassium carbonate (0.632 g, 4.57 mmol) in DMF (13.0 mL), added 1,3-Dibromopropane (0.46 mL, 4.57 mmol) to the mixture and stirred at the room temperature for 12 h, diluted the mixture with DCM, washed the mixture with water, separated the organic layer, dried the resultant over anhydrous sodium sulfate and concentrated the mixture under reduced pressure, purified by flash chromatography (silica gel: 10% EtOAc in Petroleum ether) to provide the title compound as a white solid (0.601 g, 50% yield), mp 47-48 °C. ^{1}H NMR (400 MHz, $CDCl_3$) δ: 10.34 (s, 1H), 8.42 (dd, J_1 = 4.7 Hz, J_2 = 1.9 Hz, 1H), 8.25 (dd, J_1 = 7.8 Hz, J_2 = 2.0 Hz,, 1H), 8.10 (s, 1H), 7.87 (d, J = 8.3 Hz, 1H), 7.43 (t, J = 7.9 Hz, 1H), 7.29 (d, J = 7.8 Hz, 1H), 6.79 (dd, J_1 = 7.8 Hz, J_2 = 4.7 Hz, 1H), 4.50 (t, J = 6.1 Hz, 2H), 3.55 (t, J = 6.5 Hz, 2H), 2.35 (p, J = 6.3 Hz, 2H); ^{13}C NMR (100 MHz, $CDCl_3$) δ: 167.41, 155.95, 153.42, 140.39, 140.26, 131.28 (q, J = 31.8 Hz), 129.34, 124.32 (q, J = 270.8 Hz), 123.68, 119.23 (q, J = 3.6 Hz), 117.31 (q, J = 3.7 Hz), 114.20, 107.33, 63.26, 31.72, 29.29; ^{19}F NMR (376 MHz, $CDCl_3$) δ: -62.47; HRMS (ESI): $[M+H]^+$ calculated for $C_{16}H_{15}BrF_3N_2O_2^+$ = 403.0269, found: 403.0268.

3-bromopropyl 4-(*N*,*N*-dipropylsulfamoyl)benzoate (1cd)

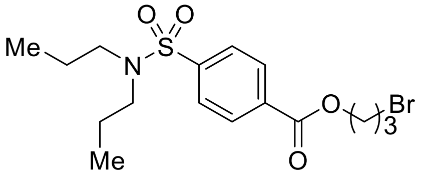

Mixed 4-(*N*,*N*-dipropylsulfamoyl)benzoic acid (1.141 g, 4.0 mmol) and K_2CO_3 (0.841 g, 6.1 mmol) in DMF (18.0 mL), added 1,3-dibromopropane (1.228 g, 6.1 mmol) to the mixture and stirred the mixture at room temperature for 12 h. Diluted the mixture with DCM, washed the mixture with water, separated the organic layer, dried the resultant over anhydrous sodium sulfate and concentrated the mixture under reduced pressure, purified by flash chromatography (silica gel: 20% EtOAc in Petroleum ether) to provide

the title compound as a white solid (1.095 g, 68% yield), mp 34-36 °C. ^{1}H NMR (400 MHz, $CDCl_3$) δ: 8.13 (d, *J* = 8.7 Hz, 2H), 7.86 (d, *J* = 8.3 Hz, 2H), 4.49 (t, *J* = 6.0 Hz, 2H), 3.54 (t, *J* = 6.5 Hz, 2H), 3.10-3.06 (m, 4H), 2.33 (p, *J* = 6.3 Hz, 2H), 1.57-1.48 (m, 4H), 0.85 (t, *J* = 7.4 Hz, 6H). All data are in accordance with the literature[17].

3-bromopropyl 2-(4-isobutoxyphenyl)-4-methylthiazole-5-carboxylate (1ce)

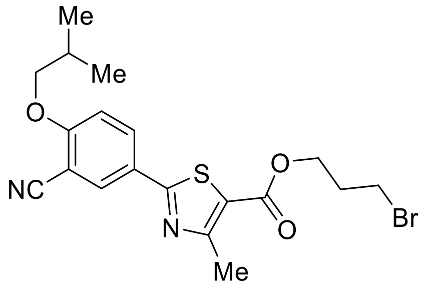

Mixed 2-(3-cyano-4-isobutoxyphenyl)-4-methylthiazole-5-carboxylic acid (0.949 g, 3.0 mmol) and K_2CO_3 (0.622 g, 4.5 mmol) in DMF (13.0 mL), added 1,3-dibromopropane (0.909 g, 4.5 mmol) to the mixture and stirred the mixture at room temperature for 12 h. Diluted the mixture with DCM, washed the mixture with water, separated the organic layer, dried the resultant over anhydrous sodium sulfate and concentrated the mixture under reduced pressure, purified by flash chromatography (silica gel: 20% EtOAc in Petroleum ether) to provide the title compound as a white solid (0.520 g, 40% yield), mp 122-124 °C. ^{1}H NMR (400 MHz, $CDCl_3$) δ: 8.18 (d, *J* = 2.3 Hz, 1H), 8.10 (dd, J_1 = 8.8 Hz, J_2 = 2.3 Hz, 1H), 7.01 (d, *J* = 8.9 Hz, 1H), 4.45 (t, *J* = 6.0 Hz, 2H), 3.90 (d, *J* = 6.5 Hz, 2H), 3.54 (t, *J* = 6.5 Hz, 2H), 2.77 (s, 3H), 2.31 (p, *J* = 6.3 Hz, 2H), 2.21 (dt, J_1 = 13.3 Hz, J_2 = 6.6 Hz, 1H), 1.10 (s, 3H), 1.08 (s, 3H). All data are in accordance with the literature[18].

3.1.2 Synthesis of vinyl chlorosilanes

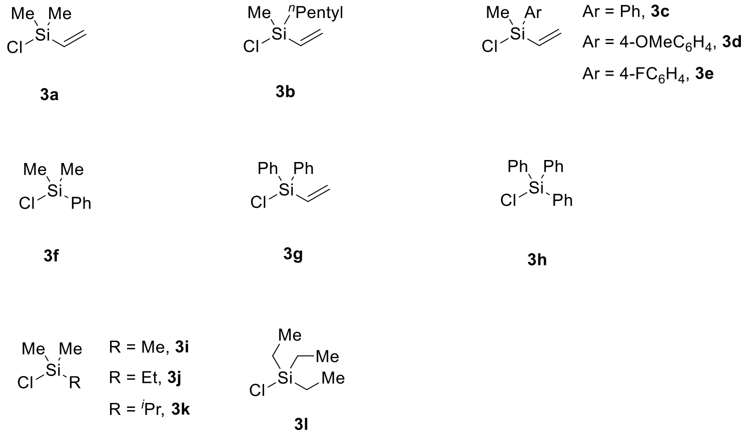

Chlorosilanes **3a, 3c, 3f-l** are commercially available. **3d and 3e** are known compounds,

and they were synthesized according to the literature procedure[19]. The preparation of **3b** and its characterization data is provided as follows.

Chloro(methyl)(pentyl)(vinyl)silane (3b)

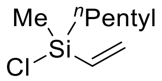

Pentylmagnesium bromide solution (20.0 mmol, 20.0 mL, 1.0 mol/L in THF) was added dropwise to the solution of dichloromethylvinylsilane (3.1 mL, 24.0 mmol) in THF (20.0 mL) at 0 °C and stirring continued for 12 h. The reaction mixture was cooled down to room temperature and filtered. The filtrate was concentrated by rotary evaporation. The residue was distilled under vacuum to afford the desired compound. Colorless oil (2.071 g, 59% yield). ^{1}H NMR (400 MHz, $CDCl_3$) δ: 6.22-6.06 (m, 2H), 5.92-5.86 (m, 1H), 1.46-1.39 (m, 2H), 1.35-1.29 (m, 4H), 0.90-0.86 (m, 5H), 0.46 (s, 3H); ^{13}C NMR (100 MHz, $CDCl_3$) δ: 135.35, 134.65, 35.27, 22.64, 22.37, 17.74, 14.07, -0.03; ^{29}Si NMR (79 MHz, $CDCl_3$) δ: -19.31; HRMS (ESI): $[M+H]^+$ calculated for $C_8H_{18}ClSi^+$ = 177.0866, found: 177.0858.

Chloro(4-methoxyphenyl)(methyl)(vinyl)silane (3d)

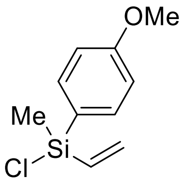

(4-methoxyphenyl)magnesium bromide solution (20.0 mmol, 20.0 mL, 1.0 mol/L in THF) was added dropwise to the solution of dichloromethylvinylsilane (3.1 mL, 24.0 mmol) in THF (20.0 mL) at 0 °C and stirring the mixture at room temperature for 12 h. The reaction mixture filtered and concentrated by rotary evaporation. The residue was distilled under vacuum to afford the desired compound. Colorless liquid (2.101 g, 47% yield). ^{1}H NMR (400 MHz, $CDCl_3$) δ: 7.58 (d, J = 8.6 Hz, 2H), 6.97 (d, J = 8.6 Hz, 2H), 6.39-6.19 (m, 2H), 6.00-5.94 (m, 1H), 3.84 (s, 3H), 0.75 (s, 3H). All data are in accordance with the literature[19].

Chloro(4-fluorophenyl)(methyl)(vinyl)silane (3e)

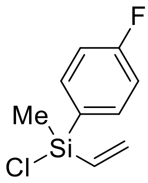

(4-fluorophenyl)magnesium bromide solution (20.0 mmol, 20.0 mL, 1.0 mol/L in THF)

was added dropwise to the solution of dichloromethylvinylsilane (3.1 mL, 24.0 mmol) in THF (20.0 mL) at 0 °C and stirring the mixture at room temperature for 12 h. The reaction mixture filtered and concentrated by rotary evaporation. The residue was distilled under vacuum to afford the desired compound. Colorless liquid (1.691 g, 42% yield). ^{1}H NMR (400 MHz, $CDCl_3$) δ: 7.62 (d, J_1 = 8.7 Hz, J_2 = 6.0 Hz, 2H), 7.14-7.09 (m, 2H), 6.36-6.20 (m, 2H), 6.00-5.94 (m, 1H), 0.75 (s, 3H). All data are in accordance with the literature[19].

3.2 Experimental Procedures and Characterization of Products

3.2.1 General Procedure A

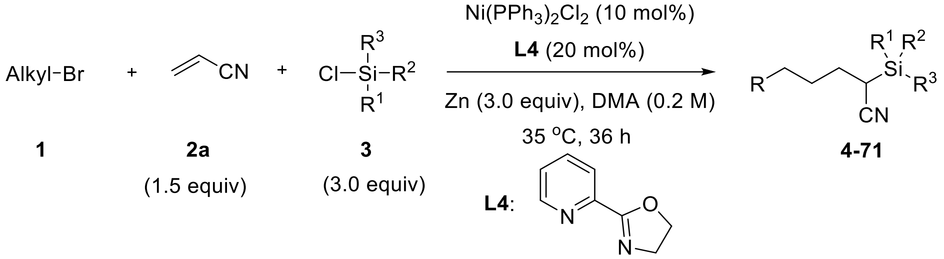

The procedure was conducted in a nitrogen-filled glove box. To a reaction vial equipped with a magnetic stir bar was added $Ni(PPh_3)_2Cl_2$ (26.4 mg, 0.04 mmol), 2-(pyridin-2-yl)-4,5-dihydrooxazole (12.0 mg, 0.08 mmol), Zn (78.0 mg, 1.2 mmol). A solution of **1** (0.4 mmol), **2a** (40.0 μL, 0.6 mmol) and chlorosilane **3** (1.2 mmol) in DMA (2.0 mL) was added. The reaction vial was sealed and removed from the glove box. The mixture was stirred at 35 °C for 36 h, subsequently quenched with water (10.0 mL) and extracted with dichloromethane (3 × 15.0 mL). The combined organic layers were washed with water, brine, dried over anhydrous Na_2SO_4, and concentrated under reduced pressure. The residue was purified by flash chromatography on silica gel to afford product **4-71**.

3.2.2 Characterization Data of Products

2-(dimethyl(vinyl)silyl)-6-phenylhexanenitrile (4)

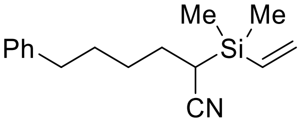

Prepared according to general procedure A using (3-bromopropyl)benzene (79.2 mg, 0.4 mmol), acrylonitrile (40.0 μL, 0.6 mmol), chlorodimethyl(vinyl)silane (144.8 mg, 1.2 mmol), $Ni(PPh_3)_2Cl_2$ (26.4 mg, 0.04 mmol), 2-(pyridin-2-yl)-4,5-dihydrooxazole (12.0 mg, 0.08 mmol), Zn (78.0 mg, 1.2 mmol), DMA (2.0 mL) at 35 °C for 36 h. The crude material was purified by flash chromatography (silica gel: 5% EtOAc in Petroleum ether) to provide the title compound as a colorless oil (74.8 mg, 73% yield). Prepared according to general procedure A using (3-iodopropyl)benzene (98.4 mg, 0.4 mmol), acrylonitrile (40.0 μL, 0.6 mmol), chlorodimethyl(vinyl)silane (144.8 mg, 1.2 mmol), $Ni(PPh_3)_2Cl_2$ (26.4 mg, 0.04 mmol), 2-(pyridin-2-yl)-4,5-dihydrooxazole (12.0 mg, 0.08 mmol), Zn (78.0 mg, 1.2 mmol), DMA (2.0 mL) at 35 °C for 36 h. The crude material was purified by flash chromatography (silica gel: 5% EtOAc in Petroleum

ether) to provide the title compound as a colorless oil (56.5 mg, 55% yield). ^{1}H NMR (400 MHz, $CDCl_3$) δ: 7.30-7.26 (m, 2H), 7.22-7.14 (m, 3H), 6.15-6.06 (m, 2H), 5.87-5.78 (m, 1H), 2.66-2.58 (m, 2H), 1.79 (dd, J_1 = 10.9 Hz, J_2 = 4.0 Hz, 1H), 1.70-1.48 (m, 6H), 0.26 (s, 6H); ^{13}C NMR (100 MHz, $CDCl_3$) δ: 142.32, 135.45, 134.17, 128.50, 125.95, 122.13, 35.81, 30.95, 29.69, 26.80, 18.41, -4.75, -5.10; ^{29}Si NMR (79 MHz, $CDCl_3$) δ: -1.58; HRMS (ESI): $[M+H]^+$ calculated for $C_{16}H_{24}NSi^+$ = 258.1678, found: 258.1679. All data are in accordance with the literature[20].

2-(dimethyl(vinyl)silyl)-5-(4-methoxyphenyl)pentanenitrile (5)

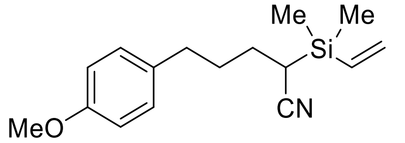

Prepared according to general procedure A using 1-(2-bromoethyl)-4-methoxybenzene (86.0 mg, 0.4 mmol), acrylonitrile (40.0 μL, 0.6 mmol), chlorodimethyl(vinyl)silane (144.8 mg, 1.2 mmol), $Ni(PPh_3)_2Cl_2$ (26.4 mg, 0.04 mmol), 2-(pyridin-2-yl)-4,5-dihydrooxazole (12.0 mg, 0.08 mmol), Zn (78.0 mg, 1.2 mmol), DMA (2.0 mL) at 35 °C for 36 h. The crude material was purified by flash chromatography (silica gel: 5% EtOAc in Petroleum ether) to provide the title compound as a colorless oil (83.1 mg, 76% yield). ^{1}H NMR (400 MHz, $CDCl_3$) δ: 7.08 (d, J = 8.6 Hz, 2H), 6.83 (d, J = 8.6 Hz, 2H), 6.14-6.07 (m, 2H), 5.87-5.78 (m, 1H), 3.79 (s, 3H), 2.65-2.52 (m, 2H), 2.01-1.90 (m, 1H), 1.83 (dd, J_1 = 10.8 Hz, J_2 = 4.5 Hz, 1H), 1.75-1.50 (m, 3H), 0.26 (s, 6H); ^{13}C NMR (100 MHz, $CDCl_3$) δ: 157.94, 135.42, 134.08, 133.58, 129.32, 122.01, 113.89, 55.34, 34.23, 31.77, 26.22, 18.19, -4.82, -5.15; ^{29}Si NMR (79 MHz, $CDCl_3$) δ: -1.57; HRMS (ESI): $[M+H]^+$ calculated for $C_{16}H_{24}NOSi^+$ = 274.1627, found: 274.1626.

Tert-butyl (4-(4-cyano-4-(dimethyl(vinyl)silyl)butyl)phenyl)carbamate (6)

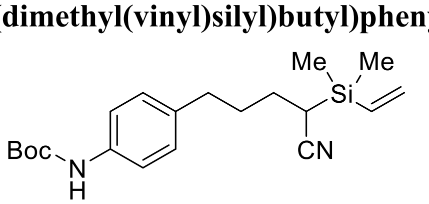

Prepared according to general procedure A using *tert*-butyl (4-(2-bromoethyl)phenyl)carbamate (120.1 mg, 0.4 mmol), acrylonitrile (40.0 μL, 0.6 mmol), chlorodimethyl(vinyl)silane (144.8 mg, 1.2 mmol), $Ni(PPh_3)_2Cl_2$ (26.4 mg, 0.04 mmol), 2-(pyridin-2-yl)-4,5-dihydrooxazole (12.0 mg, 0.08 mmol), Zn (78.0 mg, 1.2 mmol), DMA (2.0 mL) at 35 °C for 36 h. The crude material was purified by flash chromatography (silica gel: 20% EtOAc in Petroleum ether) to provide the title

compound as a white solid (73.1 mg, 51% yield), mp 96-97 °C. ^{1}H NMR (400 MHz, $CDCl_3$) δ: 7.26 (d, J = 8.3 Hz, 2H), 7.08 (d, J = 8.4 Hz, 2H), 6.41 (s, 1H), 6.14-6.06 (m, 2H), 5.86-5.78 (m, 1H), 2.64-2.54 (m, 2H), 1.99-1.90 (m, 1H), 1.82 (dd, J_1 = 10.7 Hz, J_2 = 4.5 Hz, 1H), 1.74-1.66 (m, 1H), 1.62-1.56 (m, 2H), 1.51 (s, 9H), 0.25 (s, 6H); ^{13}C NMR (100 MHz, $CDCl_3$) δ: 152.97, 136.39, 136.18, 135.47, 134.06, 128.90, 122.02, 118.81, 80.47, 34.45, 31.60, 28.45, 26.20, 18.22, -4.80, -5.13; ^{29}Si NMR (79 MHz, $CDCl_3$) δ: -1.57; HRMS (ESI): $[M+H]^+$ calculated for $C_{20}H_{31}N_2O_2Si^+$ = 359.2155, found:359.2156.

5-(4-chlorophenyl)-2-(dimethyl(vinyl)silyl)pentanenitrile (7)

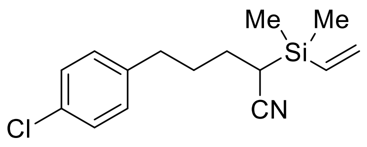

Prepared according to general procedure A using 1-(2-bromoethyl)-4-chlorobenzene (87.8 mg, 0.4 mmol), acrylonitrile (40.0 μL, 0.6 mmol), chlorodimethyl(vinyl)silane (144.8 mg, 1.2 mmol), $Ni(PPh_3)_2Cl_2$ (26.4 mg, 0.04 mmol), 2-(pyridin-2-yl)-4,5-dihydrooxazole (12.0 mg, 0.08 mmol), Zn (78.0 mg, 1.2 mmol), DMA (2.0 mL) at 35 °C for 36 h. The crude material was purified by flash chromatography (silica gel: 5% EtOAc in Petroleum ether) to provide the title compound as a colorless oil (78.9 mg, 71% yield). ^{1}H NMR (400 MHz, $CDCl_3$) δ: 7.24 (d, J = 8.8 Hz, 2H), 7.08 (d, J = 8.3 Hz, 2H), 6.14-6.05 (m, 2H), 5.86-5.77 (m, 1H), 2.67-2.53 (m, 2H), 2.00-1.90 (m, 1H), 1.81 (dd, J_1 = 10.9 Hz, J_2 = 4.5 Hz, 1H), 1.75-1.65 (m, 1H), 1.59-1.48 (m, 2H), 0.25 (s, 6H); ^{13}C NMR (100 MHz, $CDCl_3$) δ: 139.96, 135.57, 133.98, 131.79, 129.79, 128.62, 121.91, 34.53, 31.47, 26.23, 18.26, -4.80, -5.15; ^{29}Si NMR (79 MHz, $CDCl_3$) δ: -1.52; HRMS (ESI): $[M+H]^+$ calculated for $C_{15}H_{21}ClNSi^+$ = 278.1132, found: 278.1132.

5-(4-bromophenyl)-2-(dimethyl(vinyl)silyl)pentanenitrile (8)

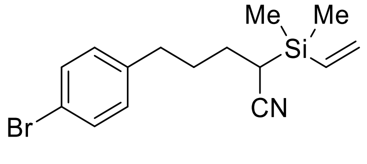

Prepared according to general procedure A using 1-bromo-4-(2-bromoethyl)benzene (105.5 mg, 0.4 mmol), acrylonitrile (40.0 μL, 0.6 mmol), chlorodimethyl(vinyl)silane (144.8 mg, 1.2 mmol), $Ni(PPh_3)_2Cl_2$ (26.4 mg, 0.04 mmol), 2-(pyridin-2-yl)-4,5-dihydrooxazole (12.0 mg, 0.08 mmol), Zn (78.0 mg, 1.2 mmol), DMA (2.0 mL) at 35 °C for 36 h. The crude material was purified by flash chromatography (silica gel: 5%

EtOAc in Petroleum ether) to provide the title compound as a colorless oil (82.9 mg, 65% yield). ^{1}H NMR (400 MHz, $CDCl_3$) δ: 7.40 (d, J = 8.4 Hz, 2H), 7.04 (d, J = 8.5 Hz, 2H), 6.17-6.06 (m, 2H) 5.87-5.78 (m, 1H), 2.67-2.52 (m, 2H), 2.03-1.90 (m, 1H), 1.82 (dd, J_1 = 11.0 Hz, J_2 = 4.4 Hz, 1H), 1.76-1.65 (m, 1H), 1.61-1.47 (m, 2H), 0.259 (s, 3H), 0.257 (s, 3H); ^{13}C NMR (100 MHz, $CDCl_3$) δ: 140.45, 135.53, 133.94, 131.53, 130.17, 121.86, 119.76, 34.55, 31.37, 26.19, 18.21, -4.82, -5.17; ^{29}Si NMR (79 MHz, $CDCl_3$) δ: -1.29; HRMS (ESI): $[M+H]^+$ calculated for $C_{15}H_{21}BrNSi^+$ = 322.0627, found: 322.0627.

4-(4-cyano-4-(dimethyl(vinyl)silyl)butyl)benzonitrile (9)

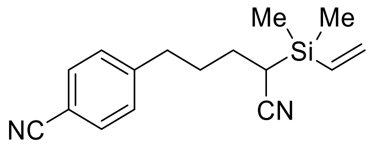

Prepared according to general procedure A using 4-(2-bromoethyl)benzonitrile (84.0 mg, 0.4 mmol), acrylonitrile (40.0 μL, 0.6 mmol), chlorodimethyl(vinyl)silane (144.8 mg, 1.2 mmol), $Ni(PPh_3)_2Cl_2$ (26.4 mg, 0.04 mmol), 2-(pyridin-2-yl)-4,5-dihydrooxazole (12.0 mg, 0.08 mmol), Zn (78.0 mg, 1.2 mmol), DMA (2.0 mL) at 35 °C for 36 h. The crude material was purified by flash chromatography (silica gel: 5% EtOAc in Petroleum ether) to provide the title compound as a colorless oil (79.1 mg, 74% yield). ^{1}H NMR (400 MHz, $CDCl_3$) δ: 7.55 (d, J = 7.6 Hz, 2H), 7.26 (d, J = 8.2 Hz, 2H), 6.13-6.03 (m, 2H), 5.85-5.76 (m, 1H), 2.74-2.63 (m, 2H), 2.03-1.92 (m, 1H), 1.82 (dd, J_1 = 10.9 Hz, J_2 = 4.6 Hz, 1H), 1.77-1.69 (m, 1H), 1.61-1.49 (m, 2H), 0.24 (s, 6H); ^{13}C NMR (100 MHz, $CDCl_3$) δ: 147.17, 135.65, 133.80, 132.34, 129.21, 121.74, 119.07, 109.98, 35.29, 31.08, 26.22, 18.23, -4.86, -5.23; ^{29}Si NMR (79 MHz, $CDCl_3$) δ: -1.45; HRMS (ESI): $[M+H]^+$ calculated for $C_{16}H_{21}N_2Si^+$ = 269.1474, found: 269.1474.

2-(dimethyl(vinyl)silyl)-5-(4-hydroxyphenyl)pentanenitrile (10)

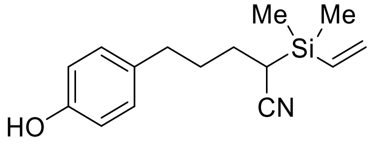

Prepared according to general procedure A using 4-(2-bromoethyl)phenol (80.4 mg, 0.4 mmol), acrylonitrile (40.0 μL, 0.6 mmol), chlorodimethyl(vinyl)silane (144.8 mg, 1.2 mmol), $Ni(PPh_3)_2Cl_2$ (26.4 mg, 0.04 mmol), 2-(pyridin-2-yl)-4,5-dihydrooxazole (12.0 mg, 0.08 mmol), Zn (78.0 mg, 1.2 mmol), DMA (2.0 mL) at 35 °C for 36 h. The crude

material was purified by flash chromatography (silica gel: 20% EtOAc in Petroleum ether) to provide the title compound as a yellow solid (25.5 mg, 25% yield), mp 72-74 °C. ^{1}H NMR (400 MHz, $CDCl_3$) δ: 7.02 (d, J = 8.4 Hz, 2H), 6.76 (d, J = 8.5 Hz, 2H), 6.15-6.06 (m, 2H), 5.87-5.78 (m, 1H), 5.22 (s, 1H), 2.63-2.49 (m, 2H), 2.00-1.89 (m, 1H), 1.83 (dd, J_1 = 10.8 Hz, J_2 = 4.5 Hz, 1H), 1.74-1.68 (m, 1H), 1.63-1.50 (m, 2H), 0.26 (s, 6H); ^{13}C NMR (100 MHz, $CDCl_3$) δ: 154.05, 135.54, 134.04, 133.54, 129.49, 122.12, 115.38, 34.29, 31.83, 26.22, 18.23, -4.79, -5.11; ^{29}Si NMR (79 MHz, $CDCl_3$) δ: -1.50; HRMS (ESI): $[M+H]^+$ calculated for $C_{15}H_{22}NOSi^+$ = 260.1471, found: 260.1472.

2-(dimethyl(vinyl)silyl)-5-(thiophen-2-yl)pentanenitrile (11)

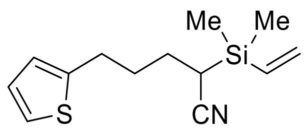

Prepared according to general procedure A using 2-(2-bromoethyl)thiophene (76.0 mg, 0.4 mmol), acrylonitrile (40.0 μL, 0.6 mmol), chlorodimethyl(vinyl)silane (144.8 mg, 1.2 mmol), $Ni(PPh_3)_2Cl_2$ (26.4 mg, 0.04 mmol), 2-(pyridin-2-yl)-4,5-dihydrooxazole (12.0 mg, 0.08 mmol), Zn (78.0 mg, 1.2 mmol), DMA (2.0 mL) at 35 °C for 36 h. The crude material was purified by flash chromatography (silica gel: 20% EtOAc in Petroleum ether) to provide the title compound as a colorless oil (80.7 mg, 81% yield). ^{1}H NMR (400 MHz, $CDCl_3$) δ: 7.12 (dd, J_1 = 5.1 Hz, J_2 = 1.2 Hz, 1H), 6.92 (dd, J_1 = 5.1 Hz, J_2 = 3.4 Hz, 1H), 6.79 (d, J = 3.4 Hz, 1H), 6.15-6.07 (m, 2H), 5.87-5.79 (m, 1H), 2.91-2.83 (m, 2H), 2.10-1.99 (m, 1H), 1.85-1.75 (m, 2H), 1.65-1.56 (m, 2H), 0.26 (s, 6H); ^{13}C NMR (100 MHz, $CDCl_3$) δ: 144.21, 135.57, 134.02, 126.92, 124.50, 123.31, 121.93, 31.80, 29.15, 26.09, 18.15, -4.79, -5.12; ^{29}Si NMR (79 MHz, $CDCl_3$) δ: -1.47; HRMS (ESI): $[M+H]^+$ calculated for $C_{13}H_{20}NSSi^+$ = 250.1086, found: 250.1084.

Tert-butyl 3-(4-cyano-4-(dimethyl(vinyl)silyl)butyl)indoline-1-carboxylate (12)

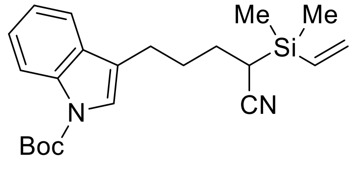

Prepared according to general procedure A using *tert*-butyl 3-(2-bromoethyl)-1*H*-indole-1-carboxylate (129.2 mg, 0.4 mmol), acrylonitrile (40.0 μL, 0.6 mmol), chlorodimethyl(vinyl)silane (144.8 mg, 1.2 mmol), $Ni(PPh_3)_2Cl_2$ (26.4 mg, 0.04 mmol), 2-(pyridin-2-yl)-4,5-dihydrooxazole (12.0 mg, 0.08 mmol), Zn (78.0 mg, 1.2 mmol),

DMA (2.0 mL) at 35 ºC for 36 h. The crude material was purified by flash chromatography (silica gel: 50% EtOAc in Petroleum ether) to provide the title compound as a colorless oil (98.1 mg, 65% yield). ^{1}H NMR (400 MHz, $CDCl_3$) δ: 8.13(s, 1H), 7.51 (d, J = 7.4 Hz, 1H), 7.32 (d, J_1 = 15.6 Hz, J_2 = 7.2 Hz, 2H), 7.24 (t, J = 7.5 Hz, 1H), 6.14-6.07 (m, 2H), 5.87-5.79 (m, 1H), 2.77-2.66 (m, 2H), 2.14-2.04 (m, 1H), 1.89-1.80 (m, 2H), 1.67 (s, 9H), 1.64-1.58 (m, 2H), 0.27 (s, 6H); ^{13}C NMR (100 MHz, $CDCl_3$) δ: 149.87, 135.67, 135.49, 134.06, 130.61, 124.44, 122.51, 122.46, 121.94, 120.23, 118.98, 115.38, 83.48, 29.41, 28.33, 26.59, 24.26, 18.20, -4.79, -5.14; ^{29}Si NMR (79 MHz, $CDCl_3$) δ: -1.49; HRMS (ESI): $[M+H]^+$ calculated for $C_{22}H_{31}N_2O_2Si^+$ = 383.2155, found: 383.2156.

5-(benzofuran-2-yl)-2-(dimethyl(vinyl)silyl)pentanenitrile (13)

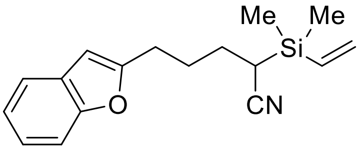

Prepared according to general procedure A using 2-(2-bromoethyl)benzofuran (89.6 mg, 0.4 mmol), acrylonitrile (40.0 μL, 0.6 mmol), chlorodimethyl(vinyl)silane (144.8 mg, 1.2 mmol), $Ni(PPh_3)_2Cl_2$ (26.4 mg, 0.04 mmol), 2-(pyridin-2-yl)-4,5-dihydrooxazole (12.0 mg, 0.08 mmol), Zn (78.0 mg, 1.2 mmol), DMA (2.0 mL) at 35 ºC for 36 h. The crude material was purified by flash chromatography (silica gel: 10% EtOAc in Petroleum ether) to provide the title compound as a colorless oil (65.5 mg, 58% yield). ^{1}H NMR (400 MHz, $CDCl_3$) δ: 7.49 (d, J = 6.9 Hz, 1H), 7.41 (d, J = 8.2 Hz, 1H), 7.24-7.17 (m, 2H), 6.40 (s, 1H), 6.15-6.07 (m, 2H), 5.88-5.79 (m, 1H), 2.82 (td, J_1 = 7.6 Hz, J_2 = 4.4 Hz, 2H), 2.18-2.07 (m, 1H), 1.92-1.85 (m, 2H), 1.70-1.59 (m, 2H), 0.27 (s, 6H); ^{13}C NMR (100 MHz, $CDCl_3$) δ: 158.31, 154.78, 135.61, 133.97, 128.92, 123.37, 121.87, 120.43, 110.84, 102.48, 102.42, 27.99, 27.71, 26.25, 18.21, -4.78, -5.15; ^{29}Si NMR (79 MHz, $CDCl_3$) δ: -1.43; HRMS (ESI): $[M+H]^+$ calculated for $C_{17}H_{22}NOSi^+$ = 284.1471, found: 284.1472.

6-(9*H*-carbazol-9-yl)-2-(dimethyl(vinyl)silyl)hexanenitrile (14)

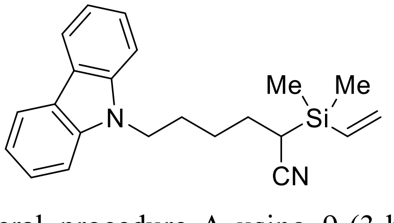

Prepared according to general procedure A using 9-(3-bromopropyl)-9*H*-carbazole

(115.2 mg, 0.4 mmol), acrylonitrile (40.0 μL, 0.6 mmol), chlorodimethyl(vinyl)silane (144.8 mg, 1.2 mmol), $Ni(PPh_3)_2Cl_2$ (26.4 mg, 0.04 mmol), 2-(pyridin-2-yl)-4,5-dihydrooxazole (12.0 mg, 0.08 mmol), Zn (78.0 mg, 1.2 mmol), DMA (2.0 mL) at 35 °C for 36 h. The crude material was purified by flash chromatography (silica gel: 50% EtOAc in Petroleum ether) to provide the title compound as a colorless oil (102.4 mg, 74% yield). ^{1}H NMR (400 MHz, $CDCl_3$) δ: 8.12 (d, J = 7.8 Hz, 2H), 7.48 (ddd, J_1 = 8.2 Hz, J_2 = 7.1 Hz, J_3 = 1.1 Hz, 2H), 7.41 (d, J = 8.2 Hz, 2H), 7.27-7.23 (m, 2H), 6.12-6.00 (m, 2H), 5.84-5.73 (m, 1H), 4.34 (t, J = 7.3 Hz, 2H), 1.97-1.86 (m, 2H), 1.80-1.70 (m, 2H), 1.58-1.42 (m, 3H), 0.21 (s, 6H); ^{13}C NMR (100 MHz, $CDCl_3$) δ: 140.38, 135.58, 133.86, 125.81, 122.95, 121.94, 120.51, 118.99, 108.68, 42.84, 28.48, 27.82, 26.67, 18.35, -4.88, -5.27; ^{29}Si NMR (79 MHz, $CDCl_3$) δ: -1.57; HRMS (ESI): $[M+H]^+$ calculated for $C_{22}H_{27}N_2Si^+$ = 347.1944, found: 347.1942.

2-(dimethyl(vinyl)silyl)-6-(pyridin-4-yl)hexanenitrile (15)

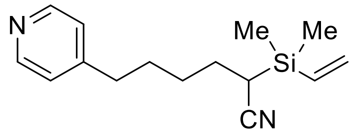

Prepared according to general procedure A using 4-(3-bromopropyl)pyridine (79.6 mg, 0.4 mmol), acrylonitrile (40.0 μL, 0.6 mmol), chlorodimethyl(vinyl)silane (144.8 mg, 1.2 mmol), $Ni(PPh_3)_2Cl_2$ (26.4 mg, 0.04 mmol), 2-(pyridin-2-yl)-4,5-dihydrooxazole (12.0 mg, 0.08 mmol), Zn (78.0 mg, 1.2 mmol), DMA (2.0 mL) at 35 °C for 36 h. The crude material was purified by flash chromatography (silica gel: 50% EtOAc in Petroleum ether) to provide the title compound as a colorless oil (40.7 mg, 39% yield). ^{1}H NMR (400 MHz, $CDCl_3$) δ: 8.47 (d, J = 5.1 Hz, 2H), 7.08 (d, J = 6.1 Hz, 2H), 6.13-6.05 (m, 2H), 5.85-5.77 (m, 1H), 2.63-2.58 (m, 2H), 1.78 (dd, J_1 = 11.0 Hz, J_2 = 4.1 Hz, 1H), 1.70-1.43 (m, 6H), 0.25 (s, 6H); ^{13}C NMR (100 MHz, $CDCl_3$) δ: 151.10, 149.81, 135.55, 133.95, 123.93, 121.96, 35.05, 29.74, 29.58, 26.62, 18.33, -4.84, -5.22; ^{29}Si NMR (79 MHz, $CDCl_3$) δ: -1.56; HRMS (ESI): $[M+H]^+$ calculated for $C_{15}H_{23}N_2Si^+$ = 259.1631, found: 259.1631.

2-(dimethyl(vinyl)silyl)-5-(quinolin-6-yl)pentanenitrile (16)

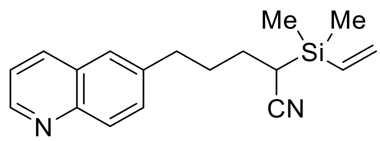

Prepared according to general procedure A using 6-(2-bromoethyl)quinoline (93.4 mg,

0.4 mmol), acrylonitrile (40.0 μL, 0.6 mmol), chlorodimethyl(vinyl)silane (144.8 mg, 1.2 mmol), $Ni(PPh_3)_2Cl_2$ (26.4 mg, 0.04 mmol), 2-(pyridin-2-yl)-4,5-dihydrooxazole (12.0 mg, 0.08 mmol), Zn (78.0 mg, 1.2 mmol), DMA (2.0 mL) at 35 °C for 36 h. The crude material was purified by flash chromatography (silica gel: 50% EtOAc in Petroleum ether) to provide the title compound as a colorless oil (46.5 mg, 40% yield). ^{1}H NMR (400 MHz, $CDCl_3$) δ: 8.87 (d, J = 5.9 Hz, 1H), 8.10 (d, J = 9.3 Hz, 1H), 8.03 (d, J = 8.6 Hz, 1H), 7.59-7.54 (m, 2H) 7.38 (dd, J_1 = 8.3 Hz, J_2 = 4.2 Hz, 1H), 6.14-6.05 (m, 2H), 5.86-5.78 (m, 1H), 2.89-2.77 (m, 2H), 2.16-2.05 (m, 1H), 1.89-1.81 (m, 2H), 1.67-1.56 (m, 2H), 0.26 (s, 6H); ^{13}C NMR (100 MHz, $CDCl_3$) δ: 149.89, 147.24, 139.92, 135.67, 135.54, 133.95, 130.85, 129.54, 128.40, 126.23, 121.90, 121.28, 35.08, 31.30, 26.31, 18.26, -4.82, -5.17; ^{29}Si NMR (79 MHz, $CDCl_3$): δ: -1.50; HRMS (ESI): $[M+H]^+$ calculated for $C_{18}H_{23}N_2Si^+$ = 295.1631, found: 295.1631.

2-(dimethyl(vinyl)silyl)heptanenitrile (17)

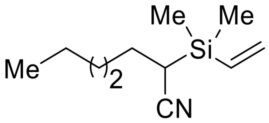

Prepared according to general procedure A using 1-bromobutane (54.4 mg, 0.4 mmol), acrylonitrile (40.0 μL, 0.6 mmol), chlorodimethyl(vinyl)silane (144.8 mg, 1.2 mmol), $Ni(PPh_3)_2Cl_2$ (26.4 mg, 0.04 mmol), 2-(pyridin-2-yl)-4,5-dihydrooxazole (12.0 mg, 0.08 mmol), Zn (78.0 mg, 1.2 mmol), DMA (2.0 mL) at 35 °C for 36 h. The crude material was purified by flash chromatography (silica gel: 5% EtOAc in Petroleum ether) to provide the title compound as a colorless oil (52.5 mg, 67% yield). ^{1}H NMR (400 MHz, $CDCl_3$) δ: 6.15-6.10 (m, 2H), 5.88-5.79 (m, 1H), 1.80 (dd, J_1 = 10.9 Hz, J_2 = 4.2 Hz, 1H), 1.68-1.61 (m, 1H), 1.54-1.25 (m, 7H), 0.89 (t, J = 6.9 Hz, 3H), 0.27 (s, 6H); ^{13}C NMR (100 MHz, $CDCl_3$) δ: 135.33, 134.20, 122.20, 31.20, 29.66, 26.76, 22.49, 18.36, 14.10, -4.80, -5.16; ^{29}Si NMR (79 MHz, $CDCl_3$) δ: -1.70; HRMS (ESI): $[M+H]^+$ calculated for $C_{11}H_{22}NSi^+$ = 196.1522, found: 196.1523.

2-(dimethyl(vinyl)silyl)nonanenitrile (18)

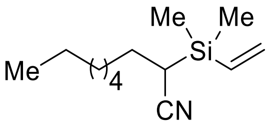

Prepared according to general procedure A using 1-bromohexane (65.6 mg, 0.4 mmol), acrylonitrile (40.0 μL, 0.6 mmol), chlorodimethyl(vinyl)silane (144.8 mg, 1.2 mmol),

$Ni(PPh_3)_2Cl_2$ (26.4 mg, 0.04 mmol), 2-(pyridin-2-yl)-4,5-dihydrooxazole (12.0 mg, 0.08 mmol), Zn (78.0 mg, 1.2 mmol), DMA (2.0 mL) at 35 °C for 36 h. The crude material was purified by flash chromatography (silica gel: 5% EtOAc in Petroleum ether) to provide the title compound as a colorless oil (61.0 mg, 68% yield). ^{1}H NMR (400 MHz, $CDCl_3$) δ: 6.17-6.09 (m, 2H), 5.87-5.78 (m, 1H), 1.79 (dd, J_1 = 10.8 Hz, J_2 = 4.3 Hz, 1H), 1.65-1.42 (m, 4H), 1.31-1.23 (m, 8H), 0.87 (t, J = 7.2 Hz, 3H), 0.25 (s, 6H); ^{13}C NMR (100 MHz, $CDCl_3$) δ: 135.31, 134.21, 122.18, 31.84, 29.98, 29.09, 28.99, 26.80, 22.69, 18.35, 14.16, -4.81, -5.16; ^{29}Si NMR (79 MHz, $CDCl_3$) δ: -1.69; HRMS (ESI): $[M+H]^+$ calculated for $C_{13}H_{26}NSi^+$ = 224.1835, found: 224.1836.

2-(dimethyl(vinyl)silyl)decanenitrile (19)

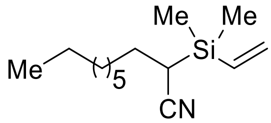

Prepared according to general procedure A using 1-bromoheptane (71.2 mg, 0.4 mmol), acrylonitrile (40.0 μL, 0.6 mmol), chlorodimethyl(vinyl)silane (144.8 mg, 1.2 mmol), $Ni(PPh_3)_2Cl_2$ (26.4 mg, 0.04 mmol), 2-(pyridin-2-yl)-4,5-dihydrooxazole (12.0 mg, 0.08 mmol), Zn (78.0 mg, 1.2 mmol), DMA (2.0 mL) at 35 °C for 36 h. The crude material was purified by flash chromatography (silica gel: 5% EtOAc in Petroleum ether) to provide the title compound as a colorless oil (71.5 mg, 75% yield). ^{1}H NMR (400 MHz, $CDCl_3$) δ: 6.19-6.08 (m, 2H), 5.88-5.79 (m, 1H), 1.80 (dd, J_1 = 10.9 Hz, J_2 = 4.3 Hz, 1H), 1.68-1.61 (m, 1H), 1.54-1.43 (m, 2H), 1.33-1.23 (m, 11H), 0.88 (t, J = 7.1 Hz, 3H), 0.27 (s, 6H); ^{13}C NMR (100 MHz, $CDCl_3$) δ: 135.35, 134.29, 122.23, 31.95, 30.03, 29.43, 29.35, 29.09, 26.87, 22.78, 18.42, 14.23, -4.75, -5.10; ^{29}Si NMR (79 MHz, $CDCl_3$) δ: -1.67; HRMS (ESI): $[M+H]^+$ calculated for $C_{14}H_{28}NSi^+$ = 238.1991, found: 238.1990.

4-cyclobutyl-2-(dimethyl(vinyl)silyl)butanenitrile (20)

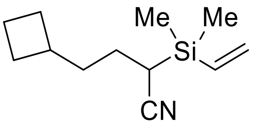

Prepared according to general procedure A using (bromomethyl)cyclobutane (59.2 mg, 0.4 mmol), acrylonitrile (40.0 μL, 0.6 mmol), chlorodimethyl(vinyl)silane (144.8 mg, 1.2 mmol), $Ni(PPh_3)_2Cl_2$ (26.4 mg, 0.04 mmol), 2-(pyridin-2-yl)-4,5-dihydrooxazole (12.0 mg, 0.08 mmol), Zn (78.0 mg, 1.2 mmol), DMA (2.0 mL) at 35 °C for 36 h. The

crude material was purified by flash chromatography (silica gel: 5% EtOAc in Petroleum ether) to provide the title compound as a colorless oil (69.6 mg, 84% yield). ^{1}H NMR (400 MHz, $CDCl_3$) δ: 6.14-6.07 (m, 2H), 5.87-5.78 (m, 1H), 2.25 (dt, J_1 = 15.4 Hz, J_2 = 7.6 Hz, 1H), 2.08-1.98 (m, 2H), 1.91-1.70 (m, 4H), 1.68-1.61 (m, 2H), 1.52-1.36 (m, 3H), 0.26 (s, 6H); ^{13}C NMR (100 MHz, $CDCl_3$) δ: 135.30, 134.17, 122.18, 37.05, 35.35, 28.36, 28.00, 24.43, 18.55, 18.29, -4.82, -5.17; ^{29}Si NMR (79 MHz, $CDCl_3$) δ: -1.69; HRMS (ESI): $[M+H]^+$ calculated for $C_{12}H_{22}NSi^+$ = 208.1522, found: 208.1523.

2-(dimethyl(vinyl)silyl)-6-methoxyhexanenitrile (21)

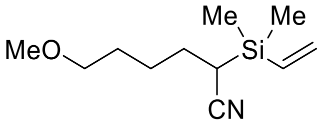

Prepared according to general procedure A using 1-bromo-3-methoxypropane (60.8 mg, 0.4 mmol), acrylonitrile (40.0 μL, 0.6 mmol), chlorodimethyl(vinyl)silane (144.8 mg, 1.2 mmol), $Ni(PPh_3)_2Cl_2$ (26.4 mg, 0.04 mmol), 2-(pyridin-2-yl)-4,5-dihydrooxazole (12.0 mg, 0.08 mmol), Zn (78.0 mg, 1.2 mmol), DMA (2.0 mL) at 35 ºC for 36 h. The crude material was purified by flash chromatography (silica gel: 5% EtOAc in Petroleum ether) to provide the title compound as a pale yellow oil (76.0 mg, 90% yield). ^{1}H NMR (400 MHz, $CDCl_3$) δ: 6.17-6.06 (m, 2H), 5.86-5.78 (m, 1H), 3.36 (t, J = 6.2 Hz, 2H), 3.31 (s, 3H), 1.80 (dd, J_1 = 10.7 Hz, J_2 = 4.3 Hz, 1H), 1.74-1.66 (m, 1H), 1.63-1.47 (m, 5H), 0.26 (s, 6H); ^{13}C NMR (100 MHz, $CDCl_3$) δ: 135.42, 134.11, 122.04, 72.44, 58.69, 29.04, 26.75, 26.70, 18.38, -4.82, -5.17; ^{29}Si NMR (79 MHz, $CDCl_3$) δ: -1.63; HRMS (ESI): $[M+H]^+$ calculated for $C_{11}H_{22}NOSi^+$ = 212.1471, found: 212.1473.

2-(dimethyl(vinyl)silyl)-7-fluoroheptanenitrile (22)

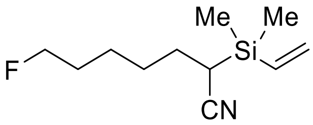

Prepared according to general procedure A using 1-bromo-4-fluorobutane (61.6 mg, 0.4 mmol), acrylonitrile (40.0 μL, 0.6 mmol), chlorodimethyl(vinyl)silane (144.8 mg, 1.2 mmol), $Ni(PPh_3)_2Cl_2$ (26.4 mg, 0.04 mmol), 2-(pyridin-2-yl)-4,5-dihydrooxazole (12.0 mg, 0.08 mmol), Zn (78.0 mg, 1.2 mmol), DMA (2.0 mL) at 35 ºC for 36 h. The crude material was purified by flash chromatography (silica gel: 5% EtOAc in Petroleum ether) to provide the title compound as a colorless oil (61.0 mg, 72% yield). ^{1}H NMR

(400 MHz, $CDCl_3$) δ: 6.16-6.08 (m, 2H), 5.88-5.80 (m, 1H), 4.50 (t, J = 6.0 Hz, 1H), 4.38 (t, J = 6.0 Hz, 1H), 1.81 (dd, J_1 = 10.9 Hz, J_2 = 4.2 Hz, 1H), 1.73-1.67 (m, 2H), 1.59-1.37 (m, 6H), 0.27 (s, 6H); ^{13}C NMR (100 MHz, $CDCl_3$) δ: 135.53, 134.10, 122.06, 84.01 (d, J = 163.7 Hz), 30.26 (d, J = 19.4 Hz), 29.63, 26.75, 24.78 (d, J = 5.1 Hz), 18.39, -4.77, -5.14; ^{29}Si NMR (79 MHz, $CDCl_3$) δ: -1.61; ^{19}F NMR (376 MHz, $CDCl_3$) δ: -218.42; HRMS (ESI): $[M+H]^+$ calculated for $C_{11}H_{21}NFSi^+$ = 214.1427, found: 214.1427.

7-chloro-2-(dimethyl(vinyl)silyl)heptanenitrile (23)

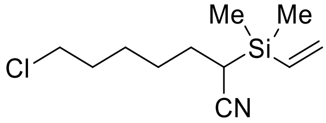

Prepared according to general procedure A using 1-bromo-4-chlorobutane (68.0 mg, 0.4 mmol), acrylonitrile (40.0 μL, 0.6 mmol), chlorodimethyl(vinyl)silane (144.8 mg, 1.2 mmol), $Ni(PPh_3)_2Cl_2$ (26.4 mg, 0.04 mmol), 2-(pyridin-2-yl)-4,5-dihydrooxazole (12.0 mg, 0.08 mmol), Zn (78.0 mg, 1.2 mmol), DMA (2.0 mL) at 35 ºC for 36 h. The crude material was purified by flash chromatography (silica gel: 5% EtOAc in Petroleum ether) to provide the title compound as a colorless oil (72.1 mg, 79% yield). ^1H NMR (400 MHz, $CDCl_3$) δ: 6.15-6.07 (m, 2H), 5.87-5.79 (m, 1H), 3.53 (t, J = 6.6 Hz, 2H), 1.83-1.74 (m, 3H), 1.70-1.64 (m, 1H), 1.56-1.40 (m, 5H), 0.26 (s, 6H); ^{13}C NMR (100 MHz, $CDCl_3$) δ: 135.51, 134.04, 121.99, 44.99, 32.33, 29.27, 26.67, 26.30, 18.35, -4.81, -5.17; ^{29}Si NMR (79 MHz, $CDCl_3$) δ: -1.57; HRMS (ESI): $[M+H]^+$ calculated for $C_{11}H_{21}NClSi^+$ = 230.1132, found: 230.1131.

Methyl 7-cyano-7-(dimethyl(vinyl)silyl)heptanoate (24)

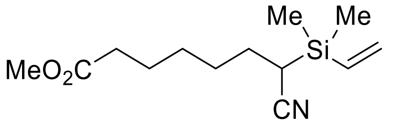

Prepared according to general procedure A using methyl 5-bromopentanoate (77.6 mg, 0.40 mmol), acrylonitrile (40.0 μL, 0.6 mmol), chlorodimethyl(vinyl)silane (144.8 mg, 1.2 mmol), $Ni(PPh_3)_2Cl_2$ (26.4 mg, 0.04 mmol), 2-(pyridin-2-yl)-4,5-dihydrooxazole (12.0 mg, 0.08 mmol), Zn (78.0 mg, 1.2 mmol), DMA (2.0 mL) at 35 ºC for 36 h. The crude material was purified by flash chromatography (silica gel: 5% EtOAc in Petroleum ether) to provide the title compound as a colorless oil (75.0 mg, 74% yield). ^1H NMR (400 MHz, $CDCl_3$) δ: 6.14-6.07 (m, 2H), 5.87-5.79 (m, 1H), 3.66 (s, 3H), 2.31

(t, $J = 7.4$ Hz, 2H), 1.80 (dd, $J_1 = 11.0$ Hz, $J_2 = 4.2$ Hz, 1H), 1.67-1.35 (m, 8H), 0.26 (s, 6H); ^{13}C NMR (100 MHz, $CDCl_3$) δ: 174.02, 135.34, 134.03, 121.93, 51.51, 33.88, 29.53, 28.40, 26.51, 24.58, 18.23, -4.91, -5.26; ^{29}Si NMR (79 MHz, $CDCl_3$) δ: -1.66; HRMS (ESI): $[M+H]^+$ calculated for $C_{13}H_{24}NO_2Si^+$ = 254.1576, found: 254.1575.

Diethyl (5-cyano-5-(dimethyl(vinyl)silyl)pentyl)phosphonate (25)

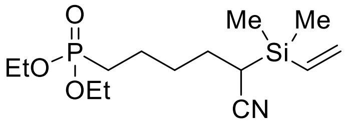

Prepared according to general procedure A using diethyl (3-bromopropyl)phosphonate (103.2 mg, 0.4 mmol), acrylonitrile (40.0 µL, 0.6 mmol), chlorodimethyl(vinyl)silane (144.8 mg, 1.2 mmol), $Ni(PPh_3)_2Cl_2$ (26.4 mg, 0.04 mmol), 2-(pyridin-2-yl)-4,5-dihydrooxazole (12.0 mg, 0.08 mmol), Zn (78.0 mg, 1.2 mmol), DMA (2.0 mL) at 35 °C for 36 h. The crude material was purified by flash chromatography (silica gel: 50% Petroleum ether in EtOAc) to provide the title compound as a colorless oil (73.6 mg, 58% yield). ^{1}H NMR (400 MHz, $CDCl_3$) δ: 6.15-6.06 (m, 2H), 5.87-5.79 (m, 1H), 4.13-4.04 (m, 4H), 1.82-1.70 (m, 6H), 1.58-1.49 (m, 3H), 1.32 (t, $J = 7.1$ Hz, 6H), 0.26 (s, 6H); ^{13}C NMR (100 MHz, $CDCl_3$) δ: 135.55, 133.95, 121.83, 61.59 (d, $J = 6.4$ Hz), 30.81 (d, $J = 16.9$ Hz), 26.40, 26.23, 24.83, 22.0 (d, $J = 5.0$ Hz), 18.24, 16.55 (d, $J = 6.0$ Hz), -4.84, -5.21; ^{29}Si NMR (79 MHz, $CDCl_3$) δ: -1.56; ^{31}P NMR (162 MHz, $CDCl_3$) δ: 32.36; HRMS (ESI): $[M+H]^+$ calculated for $C_{14}H_{29}NO_3PSi^+$ = 318.1654, found: 318.1654.

***N*-(5-cyano-5-(dimethyl(vinyl)silyl)pentyl)-4-fluorobenzamide (26)**

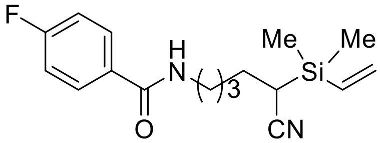

Prepared according to general procedure A using *N*-(3-bromopropyl)-4-fluorobenzamide (103.6 mg, 0.4 mmol), acrylonitrile (40.0 µL, 0.6 mmol), chlorodimethyl(vinyl)silane (144.8 mg, 1.2 mmol), $Ni(PPh_3)_2Cl_2$ (26.4 mg, 0.04 mmol), 2-(pyridin-2-yl)-4,5-dihydrooxazole (12.0 mg, 0.08 mmol), Zn (78.0 mg, 1.2 mmol), DMA (2.0 mL) at 35 °C for 36 h. The crude material was purified by flash chromatography (silica gel: 20% EtOAc in Petroleum ether) to provide the title compound as a colorless oil (56.4 mg, 44% yield). ^{1}H NMR (400 MHz, $CDCl_3$) δ: 7.78 (dd, $J_1 = 8.8$ Hz, $J_2 = 5.3$ Hz, 2H), 7.07 (t, $J = 8.6$ Hz, 2H), 6.46 (s, 1H), 6.13-6.05 (m,

2H), 5.86-5.77 (m, 1H), 3.41 (dt, J_1 = 13.3 Hz, J_2 = 6.0 Hz, 2H), 1.80 (dd, J_1 = 10.1 Hz, J_2 = 5.1 Hz, 1H), 1.70-1.48 (m, 6H), 0.25 (s, 6H); ^{13}C NMR (100 MHz, $CDCl_3$) δ: 166.68, 164.72 (d, J = 250.3 Hz), 135.61, 133.89, 130.87 (d, J = 3.1 Hz), 129.34 (d, J = 8.8 Hz), 122.06, 115.61 (d, J = 21.7 Hz), 39.82, 28.94, 27.32, 26.34, 18.35, -4.87, -5.20; ^{29}Si NMR (79 MHz, $CDCl_3$) δ: -1.52; ^{19}F NMR (376 MHz, $CDCl_3$) δ: -108.22; HRMS (ESI): $[M+H]^+$ calculated for $C_{17}H_{24}N_2OFSi^+$ = 319.1642, found: 319.1642.

2-(dimethyl(vinyl)silyl)-4-(3-oxocyclobutyl)butanenitrile (27)

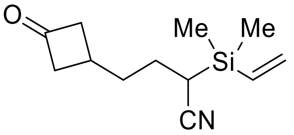

Prepared according to general procedure A using 3-(bromomethyl)cyclobutan-1-one (64.8 mg, 0.4 mmol), acrylonitrile (40.0 μL, 0.6 mmol), chlorodimethyl(vinyl)silane (144.8 mg, 1.2 mmol), $Ni(PPh_3)_2Cl_2$ (26.4 mg, 0.04 mmol), 2-(pyridin-2-yl)-4,5-dihydrooxazole (12.0 mg, 0.08 mmol), Zn (78.0 mg, 1.2 mmol), DMA (2.0 mL) at 35 °C for 36 h. The crude material was purified by flash chromatography (silica gel: 5% EtOAc in Petroleum ether) to provide the title compound as a colorless oil (55.8 mg, 63% yield). ^{1}H NMR (400 MHz, $CDCl_3$) δ: 6.16-6.07 (m, 2H), 5.88-5.79 (m, 1H), 3.21-3.10 (m, 2H), 2.73-2.65 (m, 2H), 2.38 (dt, J_1 = 16.1 Hz, J_2 = 8.0 Hz, 1H), 1.98-1.89 (m, 1H), 1.84-1.80 (m, 1H), 1.77-1.67 (m, 1H), 1.59-1.48 (m, 2H), 0.28 (s, 6H); ^{13}C NMR (100 MHz, $CDCl_3$) δ: 207.40, 135.78, 133.81, 121.79, 52.65, 52.49, 36.55, 25.57, 23.41, 18.37, -4.79, -5.19; ^{29}Si NMR (79 MHz, $CDCl_3$) δ: -1.42; HRMS (ESI): $[M+H]^+$ calculated for $C_{12}H_{20}NOSi^+$ = 222.1314, found: 222.1315.

2-(dimethyl(vinyl)silyl)-5-(1,3-dioxolan-2-yl)pentanenitrile (28)

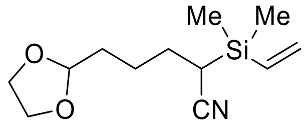

Prepared according to general procedure A using 2-(2-bromoethyl)-1,3-dioxolane (72.0 mg, 0.4 mmol), acrylonitrile (40.0 μL, 0.6 mmol), chlorodimethyl(vinyl)silane (144.8 mg, 1.2 mmol), $Ni(PPh_3)_2Cl_2$ (26.4 mg, 0.04 mmol), 2-(pyridin-2-yl)-4,5-dihydrooxazole (12.0 mg, 0.08 mmol), Zn (78.0 mg, 1.2 mmol), DMA (2.0 mL) at 35 °C for 36 h. The crude material was purified by flash chromatography (silica gel: 50% EtOAc in Petroleum ether) to provide the title compound as a colorless oil (19.4 mg, 20% yield). ^{1}H NMR (400 MHz, $CDCl_3$) δ: 6.15-6.07 (m, 2H), 5.88-5.79 (m, 1H), 4.85

(t, J = 4.5 Hz, 1H), 3.98-3.92 (m, 2H), 3.86-3.83 (m, 2H), 1.84-1.77 (m, 2H), 1.75-1.63 (m, 3H), 1.57-1.51 (m, 2H), 0.27 (s, 6H); ^{13}C NMR (100 MHz, $CDCl_3$) δ: 135.51, 134.10, 122.01, 104.21, 65.06, 65.01, 33.10, 26.77, 24.41, 18.44, -4.79, -5.14; ^{29}Si NMR (79 MHz, $CDCl_3$) δ: -1.60; HRMS (ESI): $[M+H]^+$ calculated for $C_{12}H_{22}NO_2Si^+$ = 240.1420, found: 240.1419.

2-(dimethyl(vinyl)silyl)-7,7,7-trifluoroheptanenitrile (29)

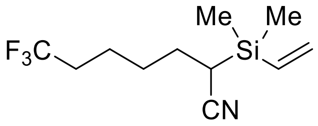

Prepared according to general procedure A using 4-bromo-1,1,1-trifluorobutane (76.0 mg, 0.4 mmol), acrylonitrile (40.0 µL, 0.6 mmol), chlorodimethyl(vinyl)silane (144.8 mg, 1.2 mmol), $Ni(PPh_3)_2Cl_2$ (26.4 mg, 0.04 mmol), 2-(pyridin-2-yl)-4,5-dihydrooxazole (12.0 mg, 0.08 mmol), Zn (78.0 mg, 1.2 mmol), DMA (2.0 mL) at 35 °C for 36 h. The crude material was purified by flash chromatography (silica gel: 5% EtOAc in Petroleum ether) to provide the title compound as a colorless oil (59.8 mg, 60% yield). ^{1}H NMR (400 MHz, $CDCl_3$) δ: 6.19-6.08 (m, 2H), 5.89-5.80 (m, 1H), 2.13-2.03 (m, 2H), 1.81 (dd, J_1 = 10.9 Hz, J_2 = 4.1 Hz, 1H), 1.76-1.68 (m, 1H), 1.63-1.48 (m, 5H), 0.28 (s, 6H); ^{13}C NMR (100 MHz, $CDCl_3$) δ: 135.70, 133.87, 127.10 (q, J = 275.0 Hz), 121.78, 33.61 (q, J = 29.0 Hz), 29.08, 26.53, 21.49 (q, J = 3.0 Hz), 18.32, -4.84, -5.23; ^{29}Si NMR (79 MHz, $CDCl_3$) δ: -1.48; ^{19}F NMR (376 MHz, $CDCl_3$) δ: -66.34; HRMS (ESI): $[M+H]^+$ calculated for $C_{11}H_{19}NF_3Si^+$ = 250.1239, found: 250.1238.

2-(dimethyl(vinyl)silyl)octanedinitrile (30)

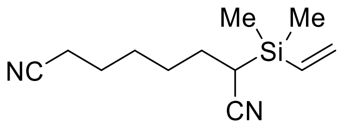

Prepared according to general procedure A using 5-bromopentanenitrile (67.9 mg, 0.4 mmol), acrylonitrile (40.0 µL, 0.6 mmol), chlorodimethyl(vinyl)silane (144.8 mg, 1.2 mmol), $Ni(PPh_3)_2Cl_2$ (26.4 mg, 0.04 mmol), 2-(pyridin-2-yl)-4,5-dihydrooxazole (12.0 mg, 0.08 mmol), Zn (78.0 mg, 1.2 mmol), DMA (2.0 mL) at 35 °C for 36 h. The crude material was purified by flash chromatography (silica gel: 5% EtOAc in Petroleum ether) to provide the title compound as a colorless oil (60.4 mg, 69% yield). ^{1}H NMR (400 MHz, $CDCl_3$) δ: 6.15-6.07 (m, 2H), 5.89-5.80 (m, 1H), 2.36 (t, J = 7.0 Hz, 2H), 1.81 (dd, J_1 = 10.9 Hz, J_2 = 4.3 Hz, 1H), 1.70-1.66 (m, 2H), 1.59-1.43 (m, 6H), 0.28 (s,

6H); ^{13}C NMR (100 MHz, $CDCl_3$) δ: 135.62, 133.93, 121.88, 119.66, 29.19, 28.06, 26.52, 25.18, 18.36, 17.20, -4.83, -5.21; ^{29}Si NMR (79 MHz, $CDCl_3$) δ: -1.53; HRMS (ESI): $[M+H]^+$ calculated for $C_{12}H_{21}N_2Si^+$ = 221.1474, found: 221.1474.

2-(dimethyl(vinyl)silyl)-6-(methylsulfonyl)hexanenitrile (31)

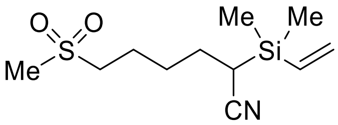

Prepared according to general procedure A using 1-bromo-3-(methylsulfonyl)propane (80.0 mg, 0.4 mmol), acrylonitrile (40.0 μL, 0.6 mmol), chlorodimethyl(vinyl)silane (144.8 mg, 1.2 mmol), $Ni(PPh_3)_2Cl_2$ (26.4 mg, 0.04 mmol), 2-(pyridin-2-yl)-4,5-dihydrooxazole (12.0 mg, 0.08 mmol), Zn (78.0 mg, 1.2 mmol), DMA (2.0 mL) at 35 °C for 36 h. The crude material was purified by flash chromatography (silica gel: 25% EtOAc in Petroleum ether) to provide the title compound as a colorless oil (72.9 mg, 70% yield). ^1H NMR (400 MHz, $CDCl_3$) δ: 6.16-6.06 (m, 2H), 5.89-5.79 (m, 1H), 3.02 (t, J = 7.9 Hz, 2H), 2.91 (s, 3H), 1.93-1.76 (m, 4H), 1.62-1.57 (m, 3H), 0.28 (s, 6H); ^{13}C NMR (100 MHz, $CDCl_3$) δ: 135.78, 133.68, 121.66, 54.35, 40.69, 28.66, 26.29, 21.71, 18.15, -4.90, -5.27; ^{29}Si NMR (79 MHz, $CDCl_3$) δ: -1.39; HRMS (ESI): $[M+H]^+$ calculated for $C_{11}H_{22}NO_2SSi^+$ = 260.1141, found: 260.1140.

2-(dimethyl(vinyl)silyl)-6-(4,4,5,5-tetramethyl-1,3,2-dioxaborolan-2-yl)hexanenitrile (32)

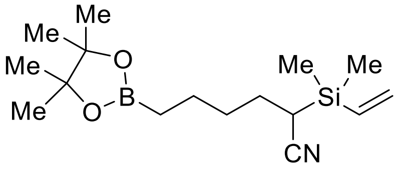

Prepared according to general procedure A using 2-(3-bromopropyl)-4,4,5,5-tetramethyl-1,3,2-dioxaborolane (99.2 mg, 0.4 mmol), acrylonitrile (40.0 μL, 0.6 mmol), chlorodimethyl(vinyl)silane (144.8 mg, 1.2 mmol), $Ni(PPh_3)_2Cl_2$ (26.4 mg, 0.04 mmol), 2-(pyridin-2-yl)-4,5-dihydrooxazole (12.0 mg, 0.08 mmol), Zn (78.0 mg, 1.2 mmol), DMA (2.0 mL) at 35 °C for 36 h. The crude material was purified by flash chromatography (silica gel: 10% EtOAc in Petroleum ether) to provide the title compound as a colorless oil (58.1 mg, 48% yield). ^1H NMR (400 MHz, $CDCl_3$) δ: 6.14-6.06 (m, 2H), 5.86-5.78 (m, 1H), 1.80 (dd, J_1 = 10.7 Hz, J_2 = 4.4 Hz, 1H), 1.66-1.39 (m, 6H), 1.67-1.24 (s, 12H), 0.78 (t, J = 7.4 Hz, 2H), 0.25 (s, 6H); ^{13}C NMR (100 MHz,

$CDCl_3$) δ: 135.32, 134.27, 122.16, 83.13, 32.51, 26.59, 24.95, 23.46, 18.26, -4.77, -5.12; ^{29}Si NMR (79 MHz, $CDCl_3$) δ: -1.67; HRMS (ESI): $[M+H]^+$ calculated for $C_{16}H_{31}NBO_2Si^+$ = 308.2217, found: 308.2217.

2-(dimethyl(vinyl)silyl)oct-7-enenitrile (33)

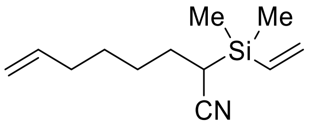

Prepared according to general procedure A using 5-bromopent-1-ene (59.1 mg, 0.4 mmol), acrylonitrile (40.0 μL, 0.6 mmol), chlorodimethyl(vinyl)silane (144.8 mg, 1.2 mmol), $Ni(PPh_3)_2Cl_2$ (26.4 mg, 0.04 mmol), 2-(pyridin-2-yl)-4,5-dihydrooxazole (12.0 mg, 0.08 mmol), Zn (78.0 mg, 1.2 mmol), DMA (2.0 mL) at 35 °C for 36 h. The crude material was purified by flash chromatography (silica gel: 3% EtOAc in Petroleum ether) to provide the title compound as a colorless oil (58.3 mg, 70% yield). ^{1}H NMR (400 MHz, $CDCl_3$) δ: 6.17-6.09 (m, 2H), 5.86-5.74 (m, 2H), 5.01-4.92 (m, 2H), 2.05 (q, J = 6.9 Hz, 2H), 1.79 (dd, J_1 = 10.9 Hz, J_2 = 4.2 Hz, 1H), 1.69-1.61 (m, 1H), 1.56-1.35 (m, 5H), 0.26 (s, 6H); ^{13}C NMR (100 MHz, $CDCl_3$) δ: 138.50, 135.35, 134.11, 122.06, 114.78, 33.47, 29.39, 28.22, 26.63, 18.30, -4.84, -5.20; ^{29}Si NMR (79 MHz, $CDCl_3$) δ: -1.63; HRMS (APCI): $[M+H]^+$ calculated for $C_{12}H_{22}NSi^+$ = 208.1522, found: 208.1513.

2-(dimethyl(vinyl)silyl)-6-(trimethylsilyl)hex-5-ynenitrile (34)

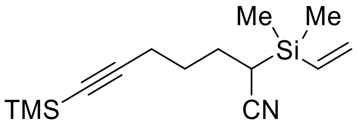

Prepared according to general procedure A using (4-bromobut-1-yn-1-yl)trimethylsilane (81.6 mg, 0.4 mmol), acrylonitrile (40.0 μL, 0.6 mmol), chlorodimethyl(vinyl)silane (144.8 mg, 1.2 mmol), $Ni(PPh_3)_2Cl_2$ (26.4 mg, 0.04 mmol), 2-(pyridin-2-yl)-4,5-dihydrooxazole (12.0 mg, 0.08 mmol), Zn (78.0 mg, 1.2 mmol), DMA (2.0 mL) at 35 °C for 36 h. The crude material was purified by flash chromatography (silica gel: 5% EtOAc in Petroleum ether) to provide the title compound as a colorless oil (68.3 mg, 65% yield). ^{1}H NMR (400 MHz, $CDCl_3$) δ: 6.19-6.08 (m, 2H), 5.89-5.81 (m, 1H), 2.30-2.27 (m, 2H), 1.87-1.84 (m, 2H), 1.68-1.61 (m, 3H), 0.28 (s, 6H), 0.14 (s, 9H); ^{13}C NMR (100 MHz, $CDCl_3$) δ: 135.51, 133.99, 121.78, 105.99, 85.70, 28.09, 25.53, 19.03, 17.67, 0.18, -4.86, -5.18; ^{29}Si NMR (79 MHz,

$CDCl_3$) δ: -1.45, -18.58; HRMS (ESI): $[M+H]^+$ calculated for $C_{14}H_{26}NSi_2^+$ = 264.1604, found: 264.1602.

2-(dimethyl(vinyl)silyl)-6-hydroxyhexanenitrile (35)

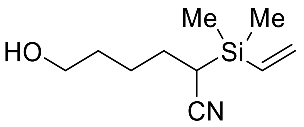

Prepared according to general procedure A using 3-bromopropan-1-ol (55.2 mg, 0.4 mmol), acrylonitrile (40.0 µL, 0.6 mmol), chlorodimethyl(vinyl)silane (144.8 mg, 1.2 mmol), $Ni(PPh_3)_2Cl_2$ (26.4 mg, 0.04 mmol), 2-(pyridin-2-yl)-4,5-dihydrooxazole (12.0 mg, 0.08 mmol), Zn (78.0 mg, 1.2 mmol), DMA (2.0 mL) at 35 °C for 36 h. The crude material was purified by flash chromatography (silica gel: 20% EtOAc in Petroleum ether) to provide the title compound as a colorless oil (21.8 mg, 28% yield). ^1H NMR (400 MHz, $CDCl_3$) δ: 6.19-6.08 (m, 2H), 5.88-5.80 (m, 1H), 3.66 (t, J = 6.3 Hz, 2H), 1.82 (dd, J_1 = 10.8 Hz, J_2 = 4.3 Hz, 1H), 1.78-1.70 (m, 1H), 1.63-1.50 (m, 5H), 0.27 (s, 6H); ^{13}C NMR (100 MHz, $CDCl_3$) δ: 135.54, 134.13, 122.07, 62.64, 32.08, 26.71, 26.36, 18.50, -4.76, -5.12; ^{29}Si NMR (79 MHz, $CDCl_3$) δ: -1.58; HRMS (ESI): $[M+H]^+$ calculated for $C_{10}H_{20}NOSi^+$ = 198.1314, found: 198.1315.

2,11-bis(dimethyl(vinyl)silyl)dodecanedinitrile (36)

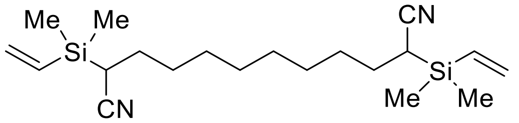

Prepared according to general procedure A using 1,6-dibromohexane (96.8 mg, 0.4 mmol), acrylonitrile (40.0 µL, 0.6 mmol), chlorodimethyl(vinyl)silane (144.8 mg, 1.2 mmol), $Ni(PPh_3)_2Cl_2$ (26.4 mg, 0.04 mmol), 2-(pyridin-2-yl)-4,5-dihydrooxazole (12.0 mg, 0.08 mmol), Zn (78.0 mg, 1.2 mmol), DMA (2.0 mL) at 35 °C for 36 h. The crude material was purified by flash chromatography (silica gel: 5% EtOAc in Petroleum ether) to provide the title compound as a pale yellow oil (76.4 mg, 53% yield). ^1H NMR (400 MHz, $CDCl_3$) δ: 6.14-6.07 (m, 4H), 5.87-5.78 (m, 2H), 1.80 (dd, J_1 = 10.9 Hz, J_2 = 4.2 Hz, 2H), 1.67-1.40 (m, 8H), 1.38-1.28 (m, 8H), 0.26 (s, 12H); ^{13}C NMR (100 MHz, $CDCl_3$) δ: 135.36, 134.18, 122.18, 29.95, 29.31, 28.95, 26.78, 18.35, -4.80, -5.15; ^{29}Si NMR (79 MHz, $CDCl_3$) δ: -1.67; HRMS (ESI): $[M+H]^+$ calculated for $C_{20}H_{37}N_2Si_2^+$ = 361.2495, found: 361.2497.

9-bromo-2-(dimethyl(vinyl)silyl)nonanenitrile (36')

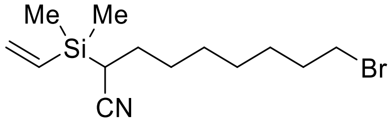

Prepared according to general procedure A using 1,6-dibromohexane (96.8 mg, 0.4 mmol), acrylonitrile (40.0 µL, 0.6 mmol), chlorodimethyl(vinyl)silane (144.8 mg, 1.2 mmol), $Ni(PPh_3)_2Cl_2$ (26.4 mg, 0.04 mmol), 2-(pyridin-2-yl)-4,5-dihydrooxazole (12.0 mg, 0.08 mmol), Zn (78.0 mg, 1.2 mmol), DMA (2.0 mL) at 35 °C for 36 h. The crude material was purified by flash chromatography (silica gel: 5% EtOAc in Petroleum ether) to provide the title compound as a pale yellow oil (28.9 mg, 24% yield). ^{1}H NMR (400 MHz, $CDCl_3$) δ: 6.15-6.08 (m, 2H), 5.88-5.79 (m, 1H), 3.40 (t, J = 6.8 Hz, 2H), 1.87-1.79 (m, 2H), 1.69-1.62 (m, 1H), 1.54-1.46 (m, 2H), 1.45-1.38 (m, 3H), 1.37-1.25 (m, 5H), 0.27 (s, 6H); ^{13}C NMR (100 MHz, $CDCl_3$) δ: 135.43, 134.17, 122.16, 34.05, 32.81, 29.91, 28.87, 28.59, 28.16, 26.77, 18.40, -4.77, -5.13; ^{29}Si NMR (79 MHz, $CDCl_3$) δ: -1.63; HRMS (ESI): $[M+H]^+$ calculated for $C_{13}H_{25}NBrSi^+$ = 302.0940, found: 302.0941.

3-cyclobutyl-2-(dimethyl(vinyl)silyl)propanenitrile (37)

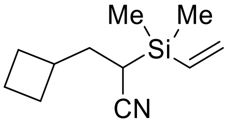

Prepared according to general procedure A using bromocyclobutane (53.6 mg, 0.4 mmol), acrylonitrile (40.0 µL, 0.6 mmol), chlorodimethyl(vinyl)silane (144.8 mg, 1.2 mmol), $Ni(PPh_3)_2Cl_2$ (26.4 mg, 0.04 mmol), 2-(pyridin-2-yl)-4,5-dihydrooxazole (12.0 mg, 0.08 mmol), Zn (78.0 mg, 1.2 mmol), DMA (2.0 mL) at 50 °C for 48 h. The crude material was purified by flash chromatography (silica gel: 5% EtOAc in Petroleum ether) to provide the title compound as a colorless oil (23.9 mg, 31% yield). ^{1}H NMR (400 MHz, $CDCl_3$) δ: 6.14-6.07 (m, 2H), 5.87-5.79 (m, 1H), 2.59-2.48 (m, 1H), 2.16-2.06 (m, 2H), 1.90-1.66 (m, 5H), 1.54-1.44 (m, 2H), 0.26 (s, 6H); ^{13}C NMR (100 MHz, $CDCl_3$) δ: 135.38, 134.19, 122.14, 36.07, 33.63, 28.24, 27.73, 18.21, 16.08, -4.84, -5.22; ^{29}Si NMR (79 MHz, $CDCl_3$) δ: -1.54; HRMS (ESI): $[M+H]^+$ calculated for $C_{11}H_{20}NSi^+$ = 194.1365, found: 194.1365.

3-cyclopentyl-2-(dimethyl(vinyl)silyl)propanenitrile (38)

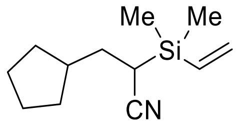

Prepared according to general procedure A using bromocyclopentane (59.2 mg, 0.4 mmol), acrylonitrile (40.0 μL, 0.6 mmol), chlorodimethyl(vinyl)silane (144.8 mg, 1.2 mmol), $Ni(PPh_3)_2Cl_2$ (26.4 mg, 0.04 mmol), 2-(pyridin-2-yl)-4,5-dihydrooxazole (12.0 mg, 0.08 mmol), Zn (78.0 mg, 1.2 mmol), DMA (2.0 mL) at 35 °C for 36 h. The crude material was purified by flash chromatography (silica gel: 5% EtOAc in Petroleum ether) to provide the title compound as a colorless oil (57.6 mg, 70% yield). Prepared according to general procedure A using iodocyclopentane (78.4 mg, 0.4 mmol), acrylonitrile (40.0 μL, 0.6 mmol), chlorodimethyl(vinyl)silane (144.8 mg, 1.2 mmol), $Ni(PPh_3)_2Cl_2$ (26.4 mg, 0.04 mmol), 2-(pyridin-2-yl)-4,5-dihydrooxazole (12.0 mg, 0.08 mmol), Zn (78.0 mg, 1.2 mmol), DMA (2.0 mL) at 35 °C for 36 h. The crude material was purified by flash chromatography (silica gel: 5% EtOAc in Petroleum ether) to provide the title compound as a colorless oil (44.7 mg, 54% yield). ^{1}H NMR (400 MHz, $CDCl_3$) δ: 6.18-6.07 (m, 2H), 5.88-5.79 (m, 1H), 2.12-2.02 (m, 1H), 1.88-1.81 (m, 3H), 1.74-1.67 (m, 1H), 1.63-1.53 (m, 4H), 1.33-1.26 (m, 1H), 1.19-1.10 (m, 1H), 1.03-0.94 (m, 1H), 0.26 (s, 6H); ^{13}C NMR (100 MHz, $CDCl_3$) δ: 135.35, 134.16, 122.33, 40.18, 32.87, 32.83, 31.70, 25.28, 25.08, 17.61, -4.88, -5.26; ^{29}Si NMR (79 MHz, $CDCl_3$) δ: -1.55; HRMS (ESI): $[M+H]^+$ calculated for $C_{12}H_{22}NSi^+$ = 208.1522, found: 208.1523.

3-cyclohexyl-2-(dimethyl(vinyl)silyl)propanenitrile (39)

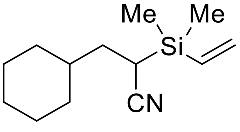

Prepared according to general procedure A using bromocyclohexane (64.8 mg, 0.4 mmol), acrylonitrile (40.0 μL, 0.6 mmol), chlorodimethyl(vinyl)silane (144.8 mg, 1.2 mmol), $Ni(PPh_3)_2Cl_2$ (26.4 mg, 0.04 mmol), 2-(pyridin-2-yl)-4,5-dihydrooxazole (12.0 mg, 0.08 mmol), Zn (78.0 mg, 1.2 mmol), DMA (2.0 mL) at 35 °C for 36 h. The crude material was purified by flash chromatography (silica gel: 5% EtOAc in Petroleum ether) to provide the title compound as a colorless oil (57.0 mg, 65% yield). ^{1}H NMR (400 MHz, $CDCl_3$) δ: 6.14-6.07 (m, 2H), 5.87-5.79 (m, 1H), 1.92-1.80 (m, 2H), 1.72-1.50 (m, 6H), 1.25-1.11 (m, 4H), 0.93-0.76 (m, 2H), 0.26 (s, 6H); ^{13}C NMR (100 MHz,

$CDCl_3$) δ: 135.39, 134.23, 122.36, 37.29, 34.07, 33.92, 31.68, 26.59, 26.24, 26.03, 15.37, -4.85, -5.22; ^{29}Si NMR (79 MHz, $CDCl_3$) δ: -1.22; HRMS (ESI): $[M+H]^+$ calculated for $C_{13}H_{24}NSi^+$ = 222.1678, found: 222.1677.

3-cycloheptyl-2-(dimethyl(vinyl)silyl)propanenitrile (40)

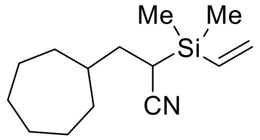

Prepared according to general procedure A using bromocycloheptane (70.4 mg, 0.4 mmol), acrylonitrile (40.0 μL, 0.6 mmol), chlorodimethyl(vinyl)silane (144.8 mg, 1.2 mmol), $Ni(PPh_3)_2Cl_2$ (26.4 mg, 0.04 mmol), 2-(pyridin-2-yl)-4,5-dihydrooxazole (12.0 mg, 0.08 mmol), Zn (78.0 mg, 1.2 mmol), DMA (2.0 mL) at 35 °C for 36 h. The crude material was purified by flash chromatography (silica gel: 5% EtOAc in Petroleum ether) to provide the title compound as a colorless oil (83.6 mg, 89% yield). ^{1}H NMR (400 MHz, $CDCl_3$) δ: 6.15-6.07 (m, 2H), 5.87-5.79 (m, 1H), 1.90 (dd, J_1 = 12.2 Hz, J_2 = 3.7 Hz, 1H), 1.79-1.69 (m, 2H), 1.64-1.52 (m, 6H), 1.50-1.42 (m, 4H), 1.29-1.23 (m, 2H), 1.10-1.02 (m, 1H), 0.26 (s, 6H); ^{13}C NMR (100 MHz, $CDCl_3$) δ: 135.35, 134.16, 122.28, 38.82, 35.47, 34.52, 32.49, 28.49, 28.37, 26.41, 26.12, 16.05, -4.89, -5.26; ^{29}Si NMR (79 MHz, $CDCl_3$) δ: -1.52; HRMS (ESI): $[M+H]^+$ calculated for $C_{14}H_{26}NSi^+$ = 236.1835, found: 236.1836.

3-((1*r*,3*r*,5*r*,7*r*)-adamantan-2-yl)-2-(dimethyl(vinyl)silyl)propanenitrile (41)

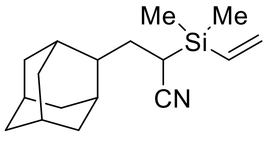

Prepared according to general procedure A using 2-bromoadamantane (85.6 mg, 0.4 mmol), acrylonitrile (40.0 μL, 0.6 mmol), chlorodimethyl(vinyl)silane (144.8 mg, 1.2 mmol), $Ni(PPh_3)_2Cl_2$ (26.4 mg, 0.04 mmol), 2-(pyridin-2-yl)-4,5-dihydrooxazole (12.0 mg, 0.08 mmol), Zn (78.0 mg, 1.2 mmol), DMA (2.0 mL) at 50 °C for 48 h. The crude material was purified by flash chromatography (silica gel: 5% EtOAc in Petroleum ether) to provide the title compound as a white solid (38.0 mg, 35% yield), mp 47-48 °C. ^{1}H NMR (400 MHz, $CDCl_3$) δ: 6.16-6.09 (m, 2H), 5.89-5.80 (m, 1H), 1.93-1.88 (m, 2H), 1.85-1.72 (m, 12H), 1.59-1.46 (m, 4H), 0.28 (s, 6H); ^{13}C NMR (100 MHz, $CDCl_3$) δ: 135.40, 134.24, 122.35, 43.80, 39.07, 38.90, 38.30, 33.09, 31.96, 31.35, 29.55, 29.39, 28.24, 28.06, 15.76, -4.78, -5.21; ^{29}Si NMR (79 MHz, $CDCl_3$) δ: -1.23; HRMS (ESI):

[M+H]$^+$ calculated for $C_{17}H_{28}NSi^+$ = 274.1991, found: 274.1991.

2-(dimethyl(vinyl)silyl)-4-ethylhexanenitrile (42)

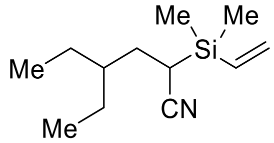

Prepared according to general procedure A using 3-bromopentane (60.0 mg, 0.4 mmol), acrylonitrile (40.0 μL, 0.6 mmol), chlorodimethyl(vinyl)silane (144.8 mg, 1.2 mmol), $Ni(PPh_3)_2Cl_2$ (26.4 mg, 0.04 mmol), 2-(pyridin-2-yl)-4,5-dihydrooxazole (12.0 mg, 0.08mmol), Zn (78.0 mg, 1.2 mmol), DMA (2.0 mL) at 35 °C for 36 h. The crude material was purified by flash chromatography (silica gel: 5% EtOAc in Petroleum ether) to provide the title compound as a pale yellow oil (57.0 mg, 67% yield). ^{1}H NMR (400 MHz, $CDCl_3$) δ: 6.15-6.08 (m, 2H), 5.88-5.80 (m, 1H), 1.86 (dd, J_1 = 12.1 Hz, J_2 = 3.6 Hz, 1H), 1.60-1.54 (m, 1H), 1.49-1.40 (m, 2H), 1.33-1.25 (m, 4H), 0.88 (t, J = 7.4 Hz, 3H), 0.82 (t, J = 7.4 Hz, 3H), 0.27 (s, 6H); ^{13}C NMR (100 MHz, $CDCl_3$) δ: 135.41, 134.14, 122.33, 39.92, 29.81, 25.54, 23.55, 15.71, 11.10, 9.85, -4.87, -5.27; ^{29}Si NMR (79 MHz, $CDCl_3$) δ: -1.18; HRMS (ESI): [M+H]$^+$ calculated for $C_{12}H_{24}NSi^+$ = 210.1678, found: 210.1677.

2-(dimethyl(vinyl)silyl)-4-methyl-6-phenylhexanenitrile (43)

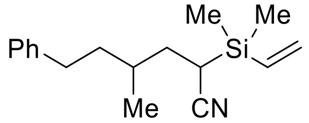

Prepared according to general procedure A using (3-bromobutyl)benzene (84.8 mg, 0.4 mmol), acrylonitrile (40.0 μL, 0.6 mmol), chlorodimethyl(vinyl)silane (144.8 mg, 1.2 mmol), $Ni(PPh_3)_2Cl_2$ (26.4 mg, 0.04 mmol), 2-(pyridin-2-yl)-4,5-dihydrooxazole (12.0 mg, 0.08 mmol), Zn (78.0 mg, 1.2 mmol), DMA (2.0 mL) at 35 °C for 36 h. The crude material was purified by flash chromatography (silica gel: 5% EtOAc in Petroleum ether) to provide the title compound as a pale yellow oil (83.1 mg, 77% yield, d.r.: 1.1:1). ^{1}H NMR (400 MHz, $CDCl_3$) δ: 7.32-7.27 (m, 2H), 7.21-7.19 (m, 3H), 6.16-6.09 (m, 2H), 5.88-5.81 (m, 1H), 2.74-2.53 (m, 2H), 1.94 (dd, J_1 = 12.4 Hz, J_2 = 3.6 Hz, 0.48H), 1.89 (dd, J_1 = 11.9 Hz, J_2 = 3.8 Hz, 0.52H), 1.79-1.52 (m, 3H), 1.40-1.30 (m, 1H), 1.21-1.15 (m, 1H), 1.06 (d, J = 6.6 Hz, 3H), 0.29 (s, 6H); ^{13}C NMR (100 MHz, $CDCl_3$) δ: 142.53, 135.51, 134.02, 128.49, 128.46, 128.39, 125.91, 125.85, 122.25, 122.01, 39.37, 37.06, 33.91, 33.64, 33.56, 33.22, 32.89, 32.64, 19.93, 18.27, 15.82, 15.74, -4.88, -5.26,

-5.31; ^{29}Si NMR (79 MHz, $CDCl_3$) δ: -1.08, -1.05; HRMS (ESI): $[M+H]^+$ calculated for $C_{17}H_{26}NSi^+$ = 272.1835, found: 272.1835.

2-(dimethyl(vinyl)silyl)-3-(tetrahydrofuran-3-yl)propanenitrile (44)

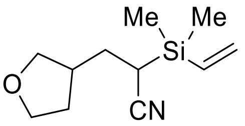

Prepared according to general procedure A using 3-bromotetrahydrofuran (60.0 mg, 0.4 mmol), acrylonitrile (40.0 µL, 0.6 mmol), chlorodimethyl(vinyl)silane (144.8 mg, 1.2 mmol), $Ni(PPh_3)_2Cl_2$ (26.4 mg, 0.04 mmol), 2-(pyridin-2-yl)-4,5-dihydrooxazole (12.0 mg, 0.08 mmol), Zn (78.0 mg, 1.2 mmol), DMA (2.0 mL) at 35 °C for 36 h. The crude material was purified by flash chromatography (silica gel: 20% EtOAc in Petroleum ether) to provide the title compound as a colorless oil (27.7 mg, 33% yield, d.r.: 1.1:1). ^{1}H NMR (400 MHz, $CDCl_3$) δ: 6.16-6.06 (m, 2H), 5.89-5.81 (m, 1H), 3.96-3.84 (m, 2H), 3.76 (dd, J_1 = 7.5 Hz, J_2 = 1.9 Hz, 1H), 3.41 (dd, J_1 = 8.5 Hz, J_2 = 6.6 Hz, 0.52H), 3.35 (dd, J_1 = 8.5 Hz, J_2 = 6.3 Hz, 0.48H), 2.55-2.43 (m, 1H), 2.20-2.06 (m, 1H), 1.85 (dd, J_1 = 12.3 Hz, J_2 = 3.6 Hz, 1H), 1.72-1.58 (m, 2H), 1.48-1.41 (m, 1H), 0.27(s, 6H); ^{13}C NMR (100 MHz, $CDCl_3$) δ: 135.87, 133.75, 133.73, 121.82, 121.75, 73.21, 72.34, 68.04, 67.93, 39.60, 39.48, 32.58, 31.51, 30.54, 30.42, 29.82, 17.80, 17.41, -4.88, -5.28; ^{29}Si NMR (79 MHz, $CDCl_3$) δ: -1.22; HRMS (ESI): $[M+H]^+$ calculated for $C_{11}H_{20}NOSi^+$ = 210.1314, found: 210.1313.

2-(dimethyl(vinyl)silyl)-3-(tetrahydro-2H-pyran-4-yl)propanenitrile (45)

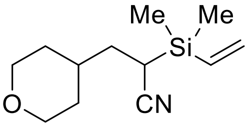

Prepared according to general procedure A using 4-bromotetrahydro-2*H*-pyran (65.6 mg, 0.4 mmol), acrylonitrile (40.0 µL, 0.6 mmol), chlorodimethyl(vinyl)silane (144.8 mg, 1.2 mmol), $Ni(PPh_3)_2Cl_2$ (26.4 mg, 0.04 mmol), 2-(pyridin-2-yl)-4,5-dihydrooxazole (12.0 mg, 0.08 mmol), Zn (78.0 mg, 1.2 mmol), DMA (2.0 mL) at 35 °C for 36 h. The crude material was purified by flash chromatography (silica gel: 20% EtOAc in Petroleum ether) to provide the title compound as a colorless oil (64.0 mg, 72% yield). ^{1}H NMR (400 MHz, $CDCl_3$) δ: 6.17-6.05 (m, 2H), 5.87-5.78 (m, 1H), 3.97-3.91 (m, 2H), 3.37 (tt, J_1 = 11.9 Hz, J_2 = 2.0 Hz, 2H), 1.90 (dd, J_1 = 12.5 Hz, J_2 = 3.7 Hz, 1H), 1.91-1.72 (m, 1H), 1.70-1.66 (m, 3H), 1.33-1.20 (m, 2H), 1.12 (qd, J_1 = 12.0

Hz, J_2 = 4.5 Hz, 1H), 0.26 (s, 6H); ^{13}C NMR (100 MHz, $CDCl_3$) δ: 135.64, 133.82, 121.84, 67.79, 34.67, 33.49, 33.40, 31.56, 14.95, -4.95, -5.32; ^{29}Si NMR (79 MHz, $CDCl_3$) δ: -1.09; HRMS (ESI): $[M+H]^+$ calculated for $C_{12}H_{22}NOSi^+$ = 224.1471, found: 224.1470.

2-(dimethyl(vinyl)silyl)-3-(1-tosylpiperidin-4-yl)propanenitrile (46)

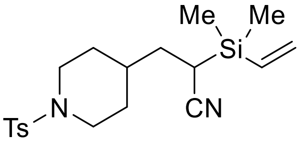

Prepared according to general procedure A using 4-bromo-1-tosylpiperidine (126.8 mg, 0.4 mmol), acrylonitrile (40.0 μL, 0.6 mmol), chlorodimethyl(vinyl)silane (144.8 mg, 1.2 mmol), $Ni(PPh_3)_2Cl_2$ (26.4 mg, 0.04 mmol), 2-(pyridin-2-yl)-4,5-dihydrooxazole (12.0 mg, 0.08mmol), Zn (78.0 mg, 1.2 mmol), DMA (2.0 mL) at 35 °C for 36 h. The crude material was purified by flash chromatography (silica gel: 5% EtOAc in Petroleum ether) to provide the title compound as a white solid (106.0 mg, 72% yield), mp 174-175 °C. ^{1}H NMR (400 MHz, $CDCl_3$) δ: 7.61 (d, J = 8.2 Hz, 2H), 7.31 (d, J = 8.0 Hz, 2H), 6.14-6.03 (m, 2H), 5.86-5.75 (m, 1H), 3.80-3.75 (m, 2H), 2.42 (s, 3H), 2.18 (dd, J_1 = 11.8 Hz, J_2 = 2.6 Hz, 2H), 1.88-1.80 (m, 2H), 1.70 (d, J = 12.4 Hz, 1H), 1.55 (td, J_1 = 13.3 Hz, J_2 = 3.6 Hz, 1H), 1.46-1.31 (m, 2H), 1.23-1.11 (m, 2H), 0.23 (s, 6H); ^{13}C NMR (100 MHz, $CDCl_3$) δ: 143.65, 135.78, 133.60, 132.74, 129.69, 127.75, 121.56, 46.37, 46.30, 34.83, 32.62, 31.99, 29.85, 21.57, 15.27, -5.01, -5.35; ^{29}Si NMR (79 MHz, $CDCl_3$) δ: -1.06; HRMS (ESI): $[M+H]^+$ calculated for $C_{19}H_{29}N_2O_2SSi^+$ = 377.1719, found: 377.1718.

2-(dimethyl(vinyl)silyl)-4,4-dimethylpentanenitrile (47)

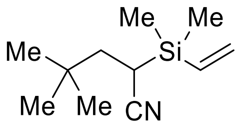

Prepared according to general procedure A using 2-bromo-2-methylpropane (54.4 mg, 0.4 mmol), acrylonitrile (40.0 μL, 0.6 mmol), chlorodimethyl(vinyl)silane (144.8 mg, 1.2 mmol), $Ni(PPh_3)_2Cl_2$ (26.4 mg, 0.04 mmol), 2-(pyridin-2-yl)-4,5-dihydrooxazole (12.0 mg, 0.08 mmol), Zn (78.0 mg, 1.2 mmol), DMA (2.0 mL) at 35 °C for 36 h. The crude material was purified by flash chromatography (silica gel: 5% EtOAc in Petroleum ether) to provide the title compound as a colorless oil (41.2 mg, 53% yield). ^{1}H NMR (400 MHz, $CDCl_3$) δ: 6.15-6.07 (m, 2H), 5.88-5.80 (m, 1H), 1.76 (dd, J_1 =

11.4 Hz, J_2 = 1.5 Hz, 1H), 1.54-1.51 (m, 1H), 1.35 (dd, J_1 = 14.1 Hz, J_2 = 1.4 Hz, 1H), 0.96 (s, 9H), 0.26 (s, 6H); ^{13}C NMR (100 MHz, $CDCl_3$) δ: 135.54, 134.05, 123.51, 40.42, 32.12, 29.07, 13.12, -4.91, -5.44; ^{29}Si NMR (79 MHz, $CDCl_3$) δ: -0.20; HRMS (ESI): $[M+H]^+$ calculated for $C_{11}H_{22}NSi^+$ = 196.1522, found: 196.1523.

2-(dimethyl(vinyl)silyl)-4,4-dimethylhexanenitrile (48)

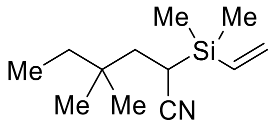

Prepared according to general procedure A using 2-bromo-2-methylbutane (60.0 mg, 0.4 mmol), acrylonitrile (40.0 μL, 0.6 mmol), chlorodimethyl(vinyl)silane (144.8 mg, 1.2 mmol), $Ni(PPh_3)_2Cl_2$ (26.4 mg, 0.04 mmol), 2-(pyridin-2-yl)-4,5-dihydrooxazole (12.0 mg, 0.08 mmol), Zn (78.0 mg, 1.2 mmol), DMA (2.0 mL) at 35 °C for 36 h. The crude material was purified by flash chromatography (silica gel: 5% EtOAc in Petroleum ether) to provide the title compound as a colorless oil (53.8 mg, 64% yield). ^{1}H NMR (400 MHz, $CDCl_3$) δ: 6.16-6.08 (m, 2H), 5.89-5.80 (m, 1H), 1.75 (dd, J_1 = 11.5 Hz, J_2 = 1.5 Hz, 1H), 1.61-1.50 (m, 2H), 1.36-1.29 (m, 2H), 0.914 (s, 3H), 0.906 (s, 3H), 0.82 (t, J = 7.5 Hz, 3H), 0.27 (s, 6H); ^{13}C NMR (100 MHz, $CDCl_3$) δ: 135.55, 134.10, 123.48, 38.00, 34.50, 33.72, 26.33, 26.23, 12.61, 8.44, -4.89, -5.44; ^{29}Si NMR (79 MHz, $CDCl_3$) δ: -0.08; HRMS (ESI): $[M+H]^+$ calculated for $C_{12}H_{24}NSi^+$ = 210.1678, found: 210.1677.

2-(dimethyl(vinyl)silyl)-4-ethyl-4-methyloctanenitrile (49)

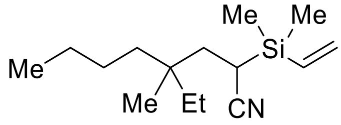

Prepared according to general procedure A using 3-bromo-3-methylheptane (76.8 mg, 0.4 mmol), acrylonitrile (40.0 μL, 0.6 mmol), chlorodimethyl(vinyl)silane (144.8 mg, 1.2 mmol), $Ni(PPh_3)_2Cl_2$ (26.4 mg, 0.04 mmol), 2-(pyridin-2-yl)-4,5-dihydrooxazole (12.0 mg, 0.08 mmol), Zn (78.0 mg, 1.2 mmol), DMA (2.0 mL) at 35 °C for 36 h. The crude material was purified by flash chromatography (silica gel: 5% EtOAc in Petroleum ether) to provide the title compound as a colorless oil (55.9 mg, 57% yield, d.r.: 1.1:1). ^{1}H NMR (400 MHz, $CDCl_3$) δ: 6.16-6.08 (m, 2H), 5.89-5.80 (m, 1H), 1.73 (s, 1H), 1.58-1.57 (m, 0.52H), 1.54-1.53 (m, 0.48H), 1.34-1.19 (m, 9H), 0.92-0.88 (m, 6H), 0.81-0.76 (m, 3H), 0.28 (s, 6H); ^{13}C NMR (100 MHz, $CDCl_3$) δ: 135.54, 134.20,

123.35, 38.17, 38.03, 36.65, 35.50, 35.43, 31.10, 30.89, 25.79, 25.73, 24.30, 24.21, 23.60, 14.28, 12.21, 8.07, 8.03, -4.84, -5.40; ^{29}Si NMR (79 MHz, $CDCl_3$) δ: -0.03; HRMS (ESI): $[M+H]^+$ calculated for $C_{15}H_{30}NSi^+$ = 252.2148, found: 252.2147.

2-(dimethyl(vinyl)silyl)-3-(1-methylcyclohexyl)propanenitrile (50)

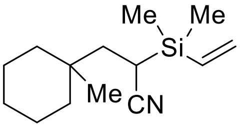

Prepared according to general procedure A using 1-bromo-1-methylcyclohexane (70.4 mg, 0.4 mmol), acrylonitrile (40.0 µL, 0.6 mmol), chlorodimethyl(vinyl)silane (144.8 mg, 1.2 mmol), $Ni(PPh_3)_2Cl_2$ (26.4 mg, 0.04 mmol), 2-(pyridin-2-yl)-4,5-dihydrooxazole (12.0 mg, 0.08 mmol), Zn (78.0 mg, 1.2 mmol), DMA (2.0 mL) at 35 °C for 36 h. The crude material was purified by flash chromatography (silica gel: 5% EtOAc in Petroleum ether) to provide the title compound as a colorless oil (35.0 mg, 37% yield). ^{1}H NMR (400 MHz, $CDCl_3$) δ: 6.16-6.08 (m, 2H), 5.89-5.80 (m, 1H), 1.79-1.78 (dd, J_1 = 11.4 Hz, J_2 = 1.3 Hz, 1H), 1.62-1.56 (m, 1H), 1.49-1.40 (m, 4H), 1.37-1.22 (m, 7H), 0.94 (s, 3H), 0.27 (s, 6H); ^{13}C NMR (100 MHz, $CDCl_3$) δ: 135.53, 134.19, 123.62, 37.53, 37.36, 34.32, 26.42, 22.08, 11.94, -4.85, -5.38; ^{29}Si NMR (79 MHz, $CDCl_3$) δ: -0.03; HRMS (ESI): $[M+H]^+$ calculated for $C_{14}H_{26}NSi^+$ = 236.1835, found: 236.1834.

2-(dimethyl(vinyl)silyl)-4,4-dimethyl-6-phenylhexanenitrile (51)

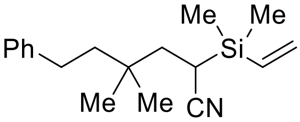

Prepared according to general procedure A using (3-bromo-3-methylbutyl)benzene (90.4 mg, 0.4 mmol), acrylonitrile (40.0 µL, 0.6 mmol), chlorodimethyl(vinyl)silane (144.8 mg, 1.2 mmol), $Ni(PPh_3)_2Cl_2$ (26.4 mg, 0.04 mmol), 2-(pyridin-2-yl)-4,5-dihydrooxazole (12.0 mg, 0.08 mmol), Zn (78.0 mg, 1.2 mmol), DMA (2.0 mL) at 35 °C for 36 h. The crude material was purified by flash chromatography (silica gel: 5% EtOAc in Petroleum ether) to provide the title compound as a colorless oil (90.1 mg, 79% yield). ^{1}H NMR (400 MHz, $CDCl_3$) δ: 7.30-7.26 (m, 2H), 7.20-7.16 (m, 3H), 6.17-6.09 (m, 2H), 5.90-5.81 (m, 1H), 2.61 (td, J_1 = 12.9 Hz, J_2 = 5.2 Hz, 1H), 2.51 (td, J_1 = 12.8 Hz, J_2 = 5.0 Hz, 1H), 1.80 (dd, J_1 = 11.5 Hz, J_2 = 1.5 Hz, 1H), 1.66-1.53 (m, 3H), 1.43-1.37 (m, 1H), 1.03 (s, 3H), 1.02 (s, 3H), 0.29 (s, 6H); ^{13}C NMR (100 MHz, $CDCl_3$)

δ: 142.86, 135.69, 133.98, 128.49, 128.42, 125.83, 123.31, 43.82, 38.32, 34.69, 30.66, 26.86, 26.67, 12.65, -4.88, -5.47; ^{29}Si NMR (79 MHz, $CDCl_3$) δ: 0.05; HRMS (ESI): $[M+H]^+$ calculated for $C_{18}H_{28}NSi^+$ = 286.1991, found: 286.1991.

2-(dimethyl(vinyl)silyl)-4,4-dimethyl-6-phenylhexanenitrile (52)

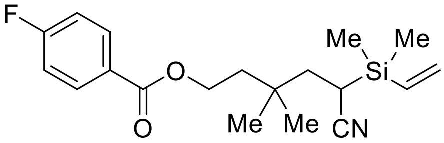

Prepared according to general procedure A using 3-bromo-3-methylbutyl 4-fluorobenzoate (115.2 mg, 0.4 mmol), acrylonitrile (40.0 μL, 0.6 mmol), chlorodimethyl(vinyl)silane (144.8 mg, 1.2 mmol), $Ni(PPh_3)_2Cl_2$ (26.4 mg, 0.04 mmol), 2-(pyridin-2-yl)-4,5-dihydrooxazole (12.0 mg, 0.08 mmol), Zn (78.0 mg, 1.2 mmol), DMA (2.0 mL) at 35 °C for 36 h. The crude material was purified by flash chromatography (silica gel: 5% EtOAc in Petroleum ether) to provide the title compound as a colorless oil (79.9 mg, 58% yield). ^{1}H NMR (400 MHz, $CDCl_3$) δ: 8.04 (dd, J_1 = 8.8 Hz, J_2 = 5.5 Hz, 2H), 7.11 (t, J = 8.6 Hz, 2H), 6.16-6.07 (m, 2H), 5.89-5.80 (m, 1H), 4.36 (t, J = 7.3 Hz, 2H), 1.85-1.73 (m, 3H), 1.68-1.61 (m, 1H), 1.45-1.41 (m, 1H), 1.07 (s, 3H), 1.04 (s, 3H), 0.28 (t, J = 2.0 Hz, 6H); ^{13}C NMR (100 MHz, $CDCl_3$) δ: 165.80 (d, J = 252.5 Hz), 165.66, 135.80, 133.76, 132.15 (d, J = 9.3 Hz), 126.59 (d, J = 3.0 Hz), 123.10, 115.57 (d, J = 21.7 Hz), 62.02, 39.65, 38.95, 34.02, 26.75, 26.73, 12.66, -4.95, -5.56; ^{29}Si NMR (79 MHz, $CDCl_3$) δ: 0.12; ^{19}F NMR (376 MHz, $CDCl_3$) δ: -105.57; HRMS (ESI): $[M+H]^+$ calculated for $C_{19}H_{27}NFO_2Si^+$ = 348.1795, found: 348.1793.

((*S*)-4-(prop-1-en-2-yl)cyclohex-1-en-1-yl)methyl 6-cyano-6-(dimethyl(vinyl)silyl)hexanoate (53)

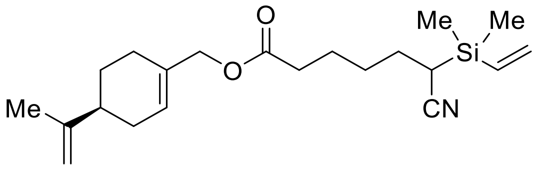

Prepared according to general procedure A using (*S*)-(4-(prop-1-en-2-yl)cyclohex-1-en-1-yl)methyl 4-bromobutanoate (120.0 mg, 0.4 mmol), acrylonitrile (40.0 μL, 0.6 mmol), chlorodimethyl(vinyl)silane (144.8 mg, 1.2 mmol), $Ni(PPh_3)_2Cl_2$ (26.4 mg, 0.04 mmol), 2-(pyridin-2-yl)-4,5-dihydrooxazole (12.0 mg, 0.08 mmol), Zn (78.0 mg, 1.2 mmol), DMA (2.0 mL) at 35 °C for 36 h. The crude material was purified by flash chromatography (silica gel: 10% EtOAc in Petroleum ether) to provide the title

compound as a colorless oil (52.9 mg, 37% yield). ^{1}H NMR (400 MHz, $CDCl_3$) δ: 6.16-6.07 (m, 2H), 5.88-5.79 (m, 1H), 5.75 (s, 1H), 4.72 (d, J = 7.5 Hz, 2H), 4.47 (s, 2H), 2.35 (t, J = 7.0 Hz, 2H), 2.19-2.07 (m, 4H), 1.89-1.79 (m, 2H), 1.74 (s, 3H), 1.70-1.58 (m, 4H), 1.55-1.46 (m, 4H), 0.27 (s, 6H); ^{13}C NMR (100 MHz, $CDCl_3$) δ: 173.34, 149.64, 135.50, 134.02, 132.69, 125.91, 121.87, 108.89, 68.50, 40.88, 34.01, 30.53, 29.46, 27.37, 26.53, 26.48, 24.32, 20.83, 18.28, -4.83, -5.19; ^{29}Si NMR (79 MHz, $CDCl_3$) δ: -1.57; HRMS (ESI): $[M+H]^+$ calculated for $C_{21}H_{34}NO_2Si^+$ = 360.2359, found: 360.2358.

2-(dimethyl(vinyl)silyl)-5-((1*R*,5*S*)-6,6-dimethylbicyclo[3.1.1]hept-2-en-2-yl)pentanenitrile (54)

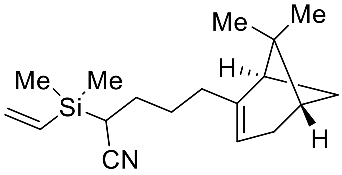

Prepared according to general procedure A using (1*R*,5*S*)-2-(2-bromoethyl)-6,6-dimethylbicyclo[3.1.1]hept-2-ene (91.2 mg, 0.4 mmol), acrylonitrile (40.0 μL, 0.6 mmol), chlorodimethyl(vinyl)silane (144.8 mg, 1.2 mmol), $Ni(PPh_3)_2Cl_2$ (26.4 mg, 0.04 mmol), 2-(pyridin-2-yl)-4,5-dihydrooxazole (12.0 mg, 0.08 mmol), Zn (78.0 mg, 1.2 mmol), DMA (2.0 mL) at 35 °C for 36 h. The crude material was purified by flash chromatography (silica gel: 5% EtOAc in Petroleum ether) to provide the title compound as a colorless oil (64.6 mg, 56% yield). ^{1}H NMR (400 MHz, $CDCl_3$) δ: 6.14-6.07 (m, 2H), 5.87-5.79 (m, 1H), 5.19 (s, 1H), 2.36-2.32 (m, 1H), 2.26-2.15 (m, 2H), 2.09-2.06 (m, 1H), 2.01-1.94 (m, 3H), 1.81 (dd, J_1 = 10.0 Hz, J_2 = 3.7 Hz, 1H), 1.76-1.67 (m, 1H), 1.54-1.45 (m, 3H), 1.26 (s, 3H), 1.11 (dd, J_1 = 8.5 Hz, J_2 = 1.8 Hz, 1H), 0.82 (d, J = 3.6 Hz, 3H), 0.26 (s, 6H); ^{13}C NMR (100 MHz, $CDCl_3$) δ: 147.53, 135.34, 134.18, 122.11, 116.73, 45.70, 40.90, 38.02, 36.16, 35.97, 31.76, 31.35, 27.52, 26.40, 21.27, 18.08, -4.81, -5.16; ^{29}Si NMR (79 MHz, $CDCl_3$) δ: -1.62; HRMS (ESI): $[M+H]^+$ calculated for $C_{18}H_{30}NSi^+$ = 288.2148, found: 288.2149.

(8*S*,9*R*,13*R*,14*R*)-13-methyl-17-oxo-7,8,9,11,12,13,14,15,16,17-decahydro-6*H*-cyclopenta[a]phenanthren-3-yl 6-cyano-6-(dimethyl(vinyl)silyl)hexanoate (55)

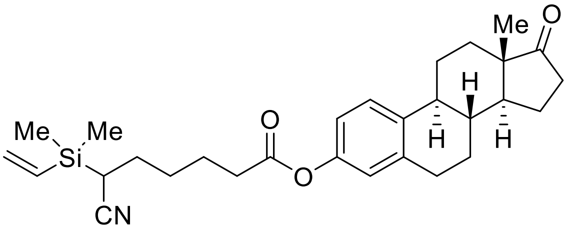

Prepared according to general procedure A using (8*S*,9*R*,13*R*,14*R*)-13-methyl-17-oxo-7,8,9,11,12,13,14,15,16,17-decahydro-6*H*-cyclopenta[*a*]phenanthren-3-yl 4-bromobutanoate (83.6 mg, 0.2 mmol), acrylonitrile (20.0 µL, 0.3 mmol), chlorodimethyl(vinyl)silane (72.4 mg, 0.6 mmol), $Ni(PPh_3)_2Cl_2$ (13.2 mg, 0.02 mmol), 2-(pyridin-2-yl)-4,5-dihydrooxazole (6.0 mg, 0.04 mmol), Zn (39.0 mg, 0.6 mmol), DMA (1.0 mL) at 35 °C for 36 h. The crude material was purified by flash chromatography (silica gel: 5% EtOAc in Petroleum ether) to provide the title compound as a colorless solid (50.2 mg, 53% yield), mp 110-112 °C. ^{1}H NMR (400 MHz, $CDCl_3$) δ: 7.28 (d, *J* = 8.5 Hz, 1H), 6.86-6.80 (m, 2H), 6.17-6.08 (m, 2H), 5.89-5.80 (m, 1H), 2.91 (dd, J_1 = 8.5 Hz, J_2 = 3.7 Hz, 2H), 2.58-2.47 (m, 3H), 2.43-2.37 (m, 1H), 2.29 (td, J_1 = 11.0 Hz, J_2 = 4.2 Hz, 1H), 2.19-1.95 (m, 4H), 1.86-1.72 (m, 4H), 1.63-1.47 (m, 9H), 0.91 (s, 3H), 0.28 (s, 6H); ^{13}C NMR (100 MHz, $CDCl_3$) δ: 220.92, 172.25, 148.63, 138.14, 137.48, 135.60, 134.00, 126.51, 121.90, 121.66, 118.82, 50.52, 48.05, 44.25, 38.09, 35.96, 34.12, 31.65, 29.49, 29.46, 26.57, 26.44, 25.85, 24.34, 21.69, 18.35, 13.93, -4.79, -5.15; ^{29}Si NMR (79 MHz, $CDCl_3$) δ: -1.51; HRMS (ESI): $[M+H]^+$ calculated for $C_{29}H_{40}NO_3Si^+$ = 478.2777, found: 478.2776.

5-(1-(4-chlorobenzoyl)-5-methoxy-2-methyl-1*H*-indol-3-yl)-2-(dimethyl(vinyl)silyl)pentanenitrile (56)

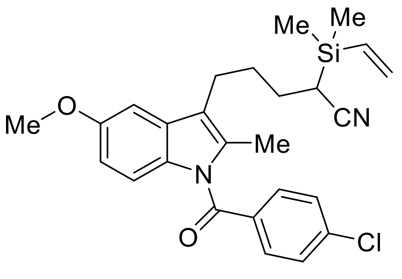

Prepared according to general procedure A using (3-(2-bromoethyl)-5-methoxy-2-methyl-1*H*-indol-1-yl)(4-chlorophenyl)methanone (162.0 mg, 0.4 mmol), acrylonitrile (40.0 µL, 0.6 mmol), chlorodimethyl(vinyl)silane (144.8 mg, 1.2 mmol), $Ni(PPh_3)_2Cl_2$ (26.4 mg, 0.04 mmol), 2-(pyridin-2-yl)-4,5-dihydrooxazole (12.0 mg, 0.08 mmol), Zn

(78.0 mg, 1.2 mmol), DMA (2.0 mL) at 35 °C for 36 h. The crude material was purified by flash chromatography (silica gel: 10% EtOAc in Petroleum ether) to provide the title compound as a yellow oil (129.4 mg, 70% yield). ^{1}H NMR (400 MHz, $CDCl_3$) δ: 7.64 (d, J = 8.4 Hz, 2H), 7.46 (d, J = 8.9 Hz, 2H), 6.92-6.87 (m, 2H), 6.66 (dd, J_1 = 9.0 Hz, J_2 = 2.5 Hz, 1H), 6.15-6.07 (m, 2H), 5.87-5.79 (m, 1H), 3.84 (s, 3H), 2.68 (q, J = 7.9 Hz, 2H), 2.32 (s, 3H), 2.03-1.93 (m, 1H), 1.90-1.86 (m, 1H), 1.80-1.58 (m, 3H), 0.27 (s, 6H); ^{13}C NMR (100 MHz, $CDCl_3$) δ: 168.38, 156.02, 139.12, 135.59, 134.27, 134.09, 133.99, 131.18, 131.07, 131.05, 129.18, 121.86, 119.13, 115.11, 111.27, 101.37, 55.87, 29.87, 26.58, 23.41, 18.20, 13.45, -4.78, -5.13; ^{29}Si NMR (79 MHz, $CDCl_3$) δ: -1.49; HRMS (ESI): $[M+H]^+$ calculated for $C_{26}H_{30}N_2O_2ClSi^+$ = 465.1765, found: 465.1765.

(5*S*)-2-(dimethyl(vinyl)silyl)-5-(6-methoxynaphthalen-2-yl)hexanenitrile (57)

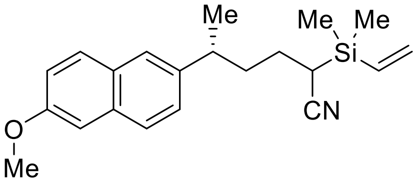

Prepared according to general procedure A using (*S*)-2-(1-bromopropan-2-yl)-6-methoxynaphthalene (111.2 mg, 0.4 mmol), acrylonitrile (40.0 μL, 0.6 mmol), chlorodimethyl(vinyl)silane (144.8 mg, 1.2 mmol), $Ni(PPh_3)_2Cl_2$ (26.4 mg, 0.04 mmol), 2-(pyridin-2-yl)-4,5-dihydrooxazole (12.0 mg, 0.08 mmol), Zn (78.0 mg, 1.2 mmol), DMA (2.0 mL) at 35 °C for 36 h. The crude material was purified by flash chromatography (silica gel: 5% EtOAc in Petroleum ether) to provide the title compound as a colorless oil (112.5 mg, 83% yield, d.r.: 1:1). ^{1}H NMR (400 MHz, $CDCl_3$) δ: 7.71-7.67 (m, 2H), 7.53 (s, 1H), 7.30 (dd, J_1 = 4.5 Hz, J_2 = 1.8 Hz, 1H), 7.16-7.12 (m, 2H), 6.09-6.00 (m, 2H), 5.81-5.77 (m, 0.5H), 5.76-5.71 (m, 0.5H), 3.92 (s, 3H), 2.85 (dd, J_1 = 13.6 Hz, J_2 = 6.9 Hz, 1H), 2.07-1.96 (m, 1H), 1.85-1.70 (m, 2H), 1.61-1.51 (m, 1H), 1.45-1.41 (m, 1H), 1.32 (d, J = 3.9 Hz, 3H), 0.20 (d, J = 1.1 Hz, 3H), 0.17 (s, 3H); ^{13}C NMR (100 MHz, $CDCl_3$) δ: 157.33, 141.96, 141.37, 135.36, 135.30, 134.05, 133.37, 133.33, 129.12, 129.07, 127.21, 127.11, 126.06, 125.92, 125.27, 125.06, 122.17, 122.06, 118.84, 105.68, 55.37, 39.68, 39.09, 38.52, 37.74, 25.14, 24.72, 22.94, 22.28, 18.53, 18.02, -4.84, -5.13, -5.17; ^{29}Si NMR (79 MHz, $CDCl_3$) δ: -1.64, -1.69; HRMS (ESI): $[M+H]^+$ calculated for $C_{21}H_{28}NOSi^+$ = 338.1940, found: 338.1940.

5-cyano-5-(dimethyl(vinyl)silyl)pentyl 2-((3-(trifluoromethyl)phenyl)amino)nicotinate (58)

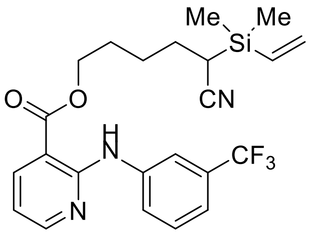

Prepared according to general procedure A using 3-bromopropyl 2-((3-(trifluoromethyl)phenyl)amino)nicotinate (160.8 mg, 0.4 mmol), acrylonitrile (40.0 μL, 0.6 mmol), chlorodimethyl(vinyl)silane (144.8 mg, 1.2 mmol), $Ni(PPh_3)_2Cl_2$ (26.4 mg, 0.04 mmol), 2-(pyridin-2-yl)-4,5-dihydrooxazole (12.0 mg, 0.08 mmol), Zn (78.0 mg, 1.2 mmol), DMA (2.0 mL) at 35 °C for 36 h. The crude material was purified by flash chromatography (silica gel: 15% EtOAc in Petroleum ether) to provide the title compound as a colorless oil (121.8 mg, 66% yield). ^{1}H NMR (400 MHz, $CDCl_3$) δ: 10.39 (s, 1H), 8.42 (dd, J_1 = 4.8 Hz, J_2 = 1.9 Hz, 1H), 8.27 (dd, J_1 = 7.8 Hz, J_2 = 1.9 Hz, 1H), 8.08 (s, 1H), 7.88 (d, J = 8.2 Hz, 1H), 7.44 (t, J = 7.9 Hz, 1H), 7.29 (d, J = 8.3 Hz, 1H), 6.80 (dd, J_1 = 7.8 Hz, J_2 = 4.8 Hz, 1H), 6.16-6.08 (m, 2H), 5.89-5.80 (m, 1H), 4.36 (td, J_1 = 6.3 Hz, J_2 = 2.6 Hz, 2H), 1.88-1.83 (m, 3H), 1.67-1.55 (m, 4H), 0.29 (s, 6H); ^{13}C NMR (100 MHz, $CDCl_3$) δ: 167.52, 155.82, 153.13, 140.44, 140.24, 135.62, 133.87, 131.14 (q, J = 32.0 Hz), 129.27, 124.28 (q, J = 270.0 Hz), 123.49, 121.81, 118.99 (q, J = 5.0 Hz), 117.06 (q, J = 4.0 Hz), 114.18, 107.54, 65.02, 28.07, 26.57, 26.48, 18.42, -4.87, -5.24; ^{29}Si NMR (79 MHz, $CDCl_3$) δ: -1.47; ^{19}F NMR (376 MHz, $CDCl_3$) δ: -62.46; HRMS (ESI): $[M+H]^+$ calculated for $C_{23}H_{27}N_3O_2F_3Si^+$ = 462.1825, found: 462.1825.

5-cyano-5-(dimethyl(vinyl)silyl)pentyl 4-(*N*,*N*-dibutylsulfamoyl)benzoate (59)

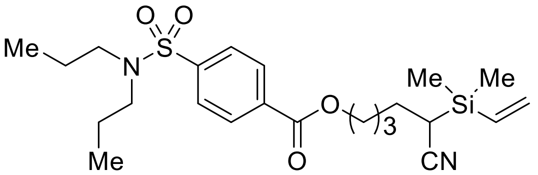

Prepared according to general procedure A using 3-bromopropyl 4-(*N*,*N*-dipropylsulfamoyl)benzoate (81.0 mg, 0.2 mmol), acrylonitrile (20.0 μL, 0.3 mmol), chlorodimethyl(vinyl)silane (72.4 mg, 0.6 mmol), $Ni(PPh_3)_2Cl_2$ (13.2 mg, 0.02 mmol), 2-(pyridin-2-yl)-4,5-dihydrooxazole (6.0 mg, 0.04 mmol), Zn (39.0 mg, 0.6 mmol), DMA (1.0 mL) at 35 °C for 36 h. The crude material was purified by flash chromatography (silica gel: 20% EtOAc in Petroleum ether) to provide the title

compound as a colorless oil (74.6 mg, 80% yield). ^{1}H NMR (400 MHz, $CDCl_3$) δ: 8.15 (d, J = 8.7 Hz, 2H), 7.87 (d, J = 8.7 Hz, 2H), 6.16-6.08 (m, 2H), 5.88-5.80 (m, 1H), 4.36 (td, J_1 = 6.4 Hz, J_2 = 2.3 Hz, 2H), 3.12-3.08 (m, 4H), 1.88-1.77 (m, 4H), 1.60-1.50 (m, 7H), 0.87 (t, J = 7.4 Hz, 6H), 0.28 (s, 6H); ^{13}C NMR (100 MHz, $CDCl_3$) δ: 165.30, 144.29, 135.60, 133.88, 133.60, 130.26, 127.08, 121.81, 65.22, 50.01, 28.10, 26.55, 26.47, 22.00, 18.39, 11.22, -4.85, -5.22; ^{29}Si NMR (79 MHz, $CDCl_3$) δ: -1.48; HRMS (ESI): $[M+H]^+$ calculated for $C_{23}H_{37}N_2O_4SSi^+$ = 465.2243, found: 465.2242.

5-cyano-5-(dimethyl(vinyl)silyl)pentyl 2-(3-cyano-4-isobutoxyphenyl)-4-methylthiazole-5-carboxylate (60)

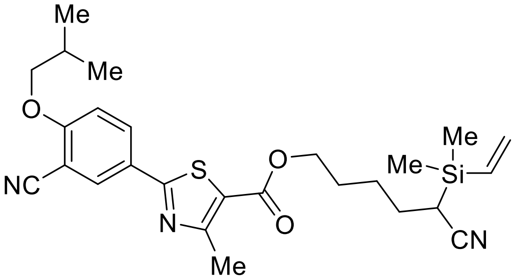

Prepared according to general procedure A using 3-bromopropyl 2-(3-cyano-4-isobutoxyphenyl)-4-methylthiazole-5-carboxylate (174.4 mg, 0.4 mmol), acrylonitrile (40.0 μL, 0.6 mmol), chlorodimethyl(vinyl)silane (144.8 mg, 1.2 mmol), $Ni(PPh_3)_2Cl_2$ (26.4 mg, 0.04 mmol), 2-(pyridin-2-yl)-4,5-dihydrooxazole (12.0 mg, 0.08 mmol), Zn (78.0 mg, 1.2 mmol), DMA (2.0 mL) at 35 °C for 36 h. The crude material was purified by flash chromatography (silica gel: 15% EtOAc in Petroleum ether) to provide the title compound as a white solid (64.1 mg, 32% yield), mp 92-94 °C. ^{1}H NMR (400 MHz, $CDCl_3$) δ: 8.17 (d, J = 1.5 Hz, 1H), 8.08 (dd, J_1 = 8.9 Hz, J_2 = 1.4 Hz, 1H), 7.00 (d, J = 8.9 Hz, 1H), 6.16-6.08 (m, 2H), 5.88-5.80 (m, 1H), 4.34-4.27 (m, 2H), 3.89 (d, J = 6.5 Hz, 2H), 2.75 (s, 3H), 2.20 (dt, J_1 = 13.4 Hz, J_2 = 6.7 Hz, 1H), 1.86-1.73 (m, 4H), 1.65-1.55 (m, 3H), 1.09 (s, 3H), 1.07 (s, 3H), 0.28 (s, 6H); ^{13}C NMR (100 MHz, $CDCl_3$) δ: 167.32, 162.57, 162.04, 161.34, 135.59, 133.92, 132.66, 132.10, 126.02, 121.78, 121.69, 115.45, 112.72, 103.01, 75.76, 64.90, 28.21, 28.13, 26.54, 26.49, 19.11, 18.42, 17.55, -4.83, -5.19; ^{29}Si NMR (79 MHz, $CDCl_3$) δ: -1.49; HRMS (ESI): $[M+H]^+$ calculated for $C_{26}H_{34}N_3O_3SSi^+$ = 496.2090, found: 496.2090.

2-(methyl(propyl)(vinyl)silyl)-6-phenylhexanenitrile (61)

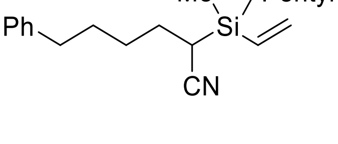

Prepared according to general procedure A using (3-bromopropyl)benzene (61.2 mg, 0.3 mmol), acrylonitrile (30.0 μL, 0.45 mmol), chloro(methyl)(pentyl)(vinyl)silane (159.1 mg, 0.9 mmol), $Ni(PPh_3)_2Cl_2$ (19.8 mg, 0.03 mmol), 2-(pyridin-2-yl)-4,5-dihydrooxazole (9.0 mg, 0.06 mmol), Zn (58.5 mg, 0.9 mmol), DMA (1.5 mL) at 35 °C for 36 h. The crude material was purified by flash chromatography (silica gel: 5% EtOAc in Petroleum ether) to provide the title compound as a colorless oil (72.0 mg, 77% yield, d.r.: 1.1:1). ^1H NMR (400 MHz, $CDCl_3$) δ: 7.33-7.27 (m, 2H), 7.21-7.16 (m, 3H), 6.18-6.05 (m, 2H), 5.87-5.80 (m, 1H), 2.68-2.60 (m, 2H), 1.84 (dd, J_1 = 3.8 Hz, J_2 = 1.7 Hz, 0.48H), 1.81 (dd, J_1 = 4.0 Hz, J_2 = 1.7 Hz, 0.52H), 1.75-1.56 (m, 4H), 1.57-1.45 (m, 2H), 1.37-1.28 (m, 6H), 0.90 (t, J = 6.9 Hz, 3H), 0.83-0.73 (m, 2H), 0.26 (s, 3H); ^{13}C NMR (100 MHz, $CDCl_3$) δ: 142.26, 135.66, 135.63, 133.32, 128.45, 128.42, 125.89, 122.15, 122.11, 35.75, 35.62, 30.87, 29.64, 26.78, 26.71, 23.00, 22.27, 17.38, 17.24, 14.02, 12.16, 11.90, -6.86, -7.14; ^{29}Si NMR (79 MHz, $CDCl_3$) δ: -1.29, -1.50; HRMS (ESI): $[M+H]^+$ calculated for $C_{20}H_{32}NSi^+$ = 314.2304, found: 314.2303.

2-(methyl(phenyl)(vinyl)silyl)-6-phenylhexanenitrile (62)

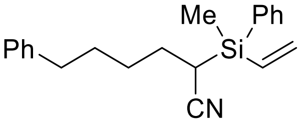

Prepared according to general procedure A using (3-bromopropyl)benzene (79.2 mg, 0.4 mmol), acrylonitrile (40.0 μL, 0.6 mmol), chloro(methyl)(phenyl)(vinyl)silane (218.4 mg, 1.2 mmol), $Ni(PPh_3)_2Cl_2$ (26.4 mg, 0.04 mmol), 2-(pyridin-2-yl)-4,5-dihydrooxazole (12.0 mg, 0.08 mmol), Zn (78.0 mg, 1.2 mmol), DMA (2.0 mL) at 35 °C for 36 h. The crude material was purified by flash chromatography (silica gel: 5% EtOAc in Petroleum ether) to provide the title compound as a colorless oil (75.2 mg, 59% yield, d.r.: 1.1:1). ^1H NMR (400 MHz, $CDCl_3$) δ: 7.58-7.55 (m, 2H), 7.46-7.38 (m, 3H), 7.28-7.25 (m, 2H), 7.19-7.11 (m, 3H), 6.41-6.24 (m, 2H), 5.96 (dd, J_1 = 9.0 Hz, J_2 = 4.1 Hz, 1H), 2.59-2.54 (m, 2H), 2.05 (dd, J_1 = 3.9 Hz, J_2 = 2.7 Hz, 0.48H), 2.02 (dd, J_1 = 4.0 Hz, J_2 = 2.4 Hz, 0.52H), 1.71-1.58 (m, 3H), 1.53-1.37 (m, 3H), 0.55 (s, 3H); ^{13}C NMR (100 MHz, $CDCl_3$) δ: 142.20, 137.20, 136.89, 134.47, 134.44, 132.72, 132.51, 132.11, 131.76, 130.43, 128.43, 128.40, 128.33, 125.87, 121.87, 35.67, 30.77, 30.74, 29.49, 29.45, 26.86, 26.79, 18.08, 17.92, -6.46, -6.67; ^{29}Si NMR (79 MHz, $CDCl_3$) δ: -8.60, -8.72; HRMS (ESI): $[M+H]^+$ calculated for $C_{21}H_{26}NSi^+$ = 320.1835, found: 320.1835.

2-((4-methoxyphenyl)(methyl)(vinyl)silyl)-6-phenylhexanenitrile (63)

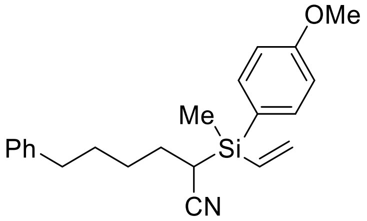

Prepared according to general procedure A using (3-bromopropyl)benzene (79.2 mg, 0.4 mmol), acrylonitrile (40.0 μL, 0.6 mmol), chloro(4-methoxyphenyl)(methyl)(vinyl)silane (254.4 mg, 1.2 mmol), $Ni(PPh_3)_2Cl_2$ (26.4 mg, 0.04 mmol), 2-(pyridin-2-yl)-4,5-dihydrooxazole (12.0 mg, 0.08 mmol), Zn (78.0 mg, 1.2 mmol), DMA (2.0 mL) at 35 ºC for 36 h. The crude material was purified by flash chromatography (silica gel: 5% EtOAc in Petroleum ether) to provide the title compound as a colorless oil (90.8 mg, 65% yield, d.r.: 1.3:1). ^{1}H NMR (400 MHz, $CDCl_3$) δ: 7.51-7.47 (m, 2H), 7.29-7.25 (m, 2H), 7.19-7.12 (m, 3H), 6.95 (d, J = 8.6 Hz, 2H), 6.40-6.22 (m, 2H), 5.94 (dd, J_1 = 8.5 Hz, J_2 = 4.0 Hz, 0.57H), 5.89 (dd, J_1 = 9.2 Hz, J_2 = 4.0 Hz, 0.43H), 3.83 (s, 3H), 2.66-2.55 (m, 2H), 2.01 (dd, J_1 = 3.8 Hz, J_2 = 2.4 Hz, 1H), 1.72-1.58 (m, 4H), 1.49-1.40 (m, 2H), 0.53 (s, 3H); ^{13}C NMR (100 MHz, $CDCl_3$) δ: 161.46, 142.22, 136.87, 136.59, 136.04, 136.01, 132.46, 132.16, 128.42, 128.38, 125.84, 123.28, 123.07, 122.03, 114.12, 55.13, 35.67, 30.77, 30.75, 29.49, 29.46, 26.84, 26.79, 18.28, 18.13, -6.31, -6.47; ^{29}Si NMR (79 MHz, $CDCl_3$) δ: -8.92, -9.05; HRMS (ESI): $[M+H]^+$ calculated for $C_{22}H_{28}NOSi^+$ = 350.1940, found: 350.1940.

2-((4-fluorophenyl)(methyl)(vinyl)silyl)-6-phenylhexanenitrile (64)

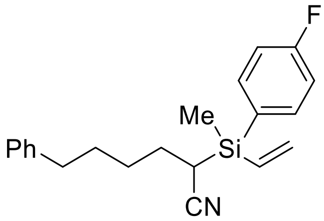

Prepared according to general procedure A using (3-bromopropyl)benzene (79.2 mg, 0.4 mmol), acrylonitrile (40.0 μL, 0.6 mmol), chloro(4-fluorophenyl)(methyl)(vinyl)silane (240.0 mg, 1.2 mmol), $Ni(PPh_3)_2Cl_2$ (26.4 mg, 0.04 mmol), 2-(pyridin-2-yl)-4,5-dihydrooxazole (12.0 mg, 0.08 mmol), Zn (78.0 mg, 1.2 mmol), DMA (2.0 mL) at 35 ºC for 36 h. The crude material was purified by flash chromatography (silica gel: 5% EtOAc in Petroleum ether) to provide the title compound as a colorless oil (97.2 mg, 72% yield, d.r.: 1.2:1). ^{1}H NMR (400 MHz, $CDCl_3$) δ: 7.58-7.53 (m, 2H), 7.29-7.25 (m, 2H), 7.18 (d, J = 6.9 Hz, 1H), 7.15-7.08 (m,

4H), 6.36-6.24 (m, 2H), 5.97-5.94 (m, 0.57H), 5.92-5.89 (m, 0.43H), 2.61-2.54 (m, 2H), 2.03 (d, J = 3.6 Hz, 1H), 1.69-1.56 (m, 3H), 1.53-1.38 (m, 3H), 0.55 (s, 3H); ^{13}C NMR (100 MHz, $CDCl_3$) δ: 164.54 (d, J = 250.0 Hz), 142.18, 137.31 (d, J = 21.0 Hz), 136.60 (d, J = 7.7 Hz), 136.58 (d, J = 7.7 Hz), 131.73 (d, J = 18.0 Hz), 128.44, 128.29 (d, J = 3.8 Hz), 128.11 (d, J = 3.9 Hz), 125.92, 121.74, 115.69 (d, J = 20.1 Hz), 115.68 (d, J = 19.8 Hz), 35.69, 30.78, 30.76, 29.48, 29.46, 26.85, 26.81, 18.15, 18.05, -6.40, -6.44; ^{29}Si NMR (79 MHz, $CDCl_3$) δ: -8.63, -8.69; ^{19}F NMR (376 MHz, $CDCl_3$) δ: -109.25; HRMS (ESI): $[M+H]^+$ calculated for $C_{21}H_{25}NFSi^+$ = 338.1740, found: 338.1740.

2-(dimethyl(phenyl)silyl)-6-phenylhexanenitrile (65)

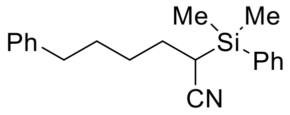

Prepared according to general procedure A using (3-bromopropyl)benzene (79.2 mg, 0.4 mmol), acrylonitrile (40.0 μL, 0.6 mmol), chlorodimethyl(phenyl)silane (204.0 mg, 1.2 mmol), $Ni(PPh_3)_2Cl_2$ (26.4 mg, 0.04 mmol), 2-(pyridin-2-yl)-4,5-dihydrooxazole (12.0 mg, 0.08 mmol), Zn (78.0 mg, 1.2 mmol), DMA (2.0 mL) at 35 ºC for 36 h. The crude material was purified by flash chromatography (silica gel: 5% EtOAc in Petroleum ether) to provide the title compound as a colorless oil (58.6 mg, 48% yield). ^{1}H NMR (400 MHz, $CDCl_3$) δ: 7.54 (dd, J_1 = 7.8 Hz, J_2 = 1.7 Hz, 2H), 7.45-7.37 (m, 3H), 7.30-7.27 (m, 1H), 7.23 (d, J = 7.5 Hz, 1H), 7.19-7.11 (m, 3H), 2.56 (td, J_1 = 7.9 Hz, J_2 = 2.7 Hz, 2H), 1.93 (dd, J_1 = 11.3 Hz, J_2 = 3.9 Hz, 1H), 1.70-1.57 (m, 3H), 1.52-1.36 (m, 3H), 0.48 (s, 6H); ^{13}C NMR (100 MHz, $CDCl_3$) δ: 142.21, 134.18, 133.95, 130.27, 128.42, 128.39, 128.27, 125.85, 122.12, 35.66, 30.76, 29.50, 26.72, 18.84, -4.42, -5.07; ^{29}Si NMR (79 MHz, $CDCl_3$) δ: -0.50; HRMS (ESI): $[M+H]^+$ calculated for $C_{20}H_{26}NSi^+$ = 308.1835, found: 308.1833.

2-(diphenyl(vinyl)silyl)-6-phenylhexanenitrile (66)

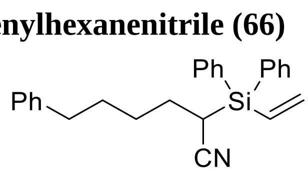

Prepared according to general procedure A using (3-bromopropyl)benzene (79.2 mg, 0.4 mmol), acrylonitrile (40.0 μL, 0.6 mmol), chlorodiphenyl(vinyl)silane (292.9 mg, 1.2 mmol), $Ni(PPh_3)_2Cl_2$ (26.4 mg, 0.04 mmol), 2-(pyridin-2-yl)-4,5-dihydrooxazole (12.0 mg, 0.08 mmol), Zn (78.0 mg, 1.2 mmol), DMA (2.0 mL) at 35 ºC for 72 h. The crude material was purified by flash chromatography (silica gel: 5% EtOAc in Petroleum ether) to provide the title compound as a colorless oil (90.7 mg, 60% yield).

^{1}H NMR (400 MHz, $CDCl_3$) δ: 7.63-7.57 (m, 4H), 7.50-7.40 (m, 6H), 7.28-7.24 (m, 2H), 7.19-7.11 (m, 3H), 6.57-6.36 (m, 2H), 5.94-5.88 (m, 1H), 2.57 (t, J = 7.5 Hz, 2H), 2.37 (dd, J_1 = 11.0 Hz, J_2 = 4.0 Hz, 1H), 1.76-1.57 (m, 5H), 1.51-1.44 (m, 1H); ^{13}C NMR (100 MHz, $CDCl_3$) δ: 142.17, 139.17, 135.62, 135.57, 130.72, 130.64, 130.35, 128.44, 128.40, 128.38, 125.87, 121.80, 35.64, 30.69, 29.47, 27.06, 17.18; ^{29}Si NMR (79 MHz, $CDCl_3$) δ: -14.48; HRMS (ESI): $[M+H]^+$ calculated for $C_{26}H_{28}NSi^+$ = 382.1991, found: 382.1992.

6-phenyl-2-(triphenylsilyl)hexanenitrile (67)

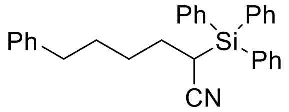

Prepared according to general procedure A using (3-bromopropyl)benzene (79.2 mg, 0.4 mmol), acrylonitrile (40.0 μL, 0.6 mmol), chlorotriphenylsilane (352.9 mg, 1.2 mmol), $Ni(PPh_3)_2Cl_2$ (26.4 mg, 0.04 mmol), 2-(pyridin-2-yl)-4,5-dihydrooxazole (12.0 mg, 0.08 mmol), Zn (78.0 mg, 1.2 mmol), DMA (2.0 mL) at 35 °C for 72 h. The crude material was purified by flash chromatography (silica gel: 5% EtOAc in Petroleum ether) to provide the title compound as a colorless oil (109.0 mg, 63% yield). ^{1}H NMR (400 MHz, $CDCl_3$) δ: 7.69-7.64 (m, 6H), 7.53-7.42 (m, 9H), 7.30-7.26 (m, 2H), 7.22-7.17 (m, 1H), 7.16-7.12 (m, 2H), 2.64-2.57 (m, 3H), 1.82-1.50 (m, 6H); ^{13}C NMR (100 MHz, $CDCl_3$) δ: 142.16, 135.95, 131.08, 130.64, 128.43, 128.41, 125.87, 122.03, 35.63, 30.64, 29.54, 27.31, 17.20; ^{29}Si NMR (79 MHz, $CDCl_3$) δ: -11.79; HRMS (ESI): $[M+H]^+$ calculated for $C_{30}H_{30}NSi^+$ = 432.2148, found: 432.2148.

6-phenyl-2-(trimethylsilyl)hexanenitrile (68)

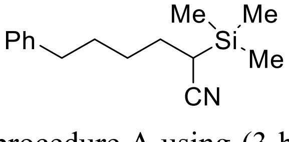

Prepared according to general procedure A using (3-bromopropyl)benzene (79.2 mg, 0.4 mmol), acrylonitrile (40.0 μL, 0.6 mmol), chlorotrimethylsilane (129.6 mg, 1.2 mmol), $Ni(PPh_3)_2Cl_2$ (26.4 mg, 0.04 mmol), 2-(pyridin-2-yl)-4,5-dihydrooxazole (12.0 mg, 0.08 mmol), Zn (78.0 mg, 1.2 mmol), DMA (2.0 mL) at 35 °C for 36 h. The crude material was purified by flash chromatography (silica gel: 5% EtOAc in Petroleum ether) to provide the title compound as a colorless oil (80.9 mg, 82% yield). ^{1}H NMR (400 MHz, $CDCl_3$) δ: 7.30-7.26 (m, 2H), 7.22-7.17 (m, 3H), 2.63 (td, J_1 = 7.7 Hz, J_2 = 3.7 Hz, 2H), 1.74 (dd, J_1 = 11.1 Hz, J_2 = 4.0 Hz, 1H), 1.71-1.57 (m, 4H), 1.53-1.43 (m, 2H), 0.18 (s, 9H); ^{13}C NMR (100 MHz, $CDCl_3$) δ: 142.27, 128.46, 128.43, 125.89,

122.33, 35.77, 30.92, 29.70, 26.65, 18.94, -3.12; ^{29}Si NMR (79 MHz, $CDCl_3$) δ: 7.45; HRMS (ESI): $[M+H]^+$ calculated for $C_{15}H_{24}NSi^+$ = 246.1678, found: 246.1678.

2-(ethyldimethylsilyl)-6-phenylhexanenitrile (69)

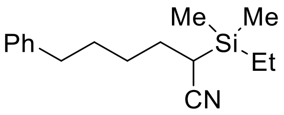

Prepared according to general procedure A using (3-bromopropyl)benzene (79.2 mg, 0.4 mmol), acrylonitrile (40.0 μL, 0.6 mmol), chloro(ethyl)dimethylsilane (146.4 mg, 1.2 mmol), $Ni(PPh_3)_2Cl_2$ (26.4 mg, 0.04 mmol), 2-(pyridin-2-yl)-4,5-dihydrooxazole (12.0 mg, 0.08 mmol), Zn (78.0 mg, 1.2 mmol), DMA (2.0 mL) at 35 ºC for 36 h. The crude material was purified by flash chromatography (silica gel: 5% EtOAc in Petroleum ether) to provide the title compound as a colorless oil (65.6 mg, 63% yield). ^{1}H NMR (400 MHz, $CDCl_3$) δ: 7.30-7.26 (m, 2H), 7.22-7.16 (m, 3H), 2.67-2.59 (m, 2H), 1.77 (dd, J_1 = 11.3 Hz, J_2 = 3.8 Hz, 1H), 1.73-1.56 (m, 4H), 1.52-1.43 (m, 2H), 0.98 (t, J = 7.9 Hz, 3H), 0.75-0.62 (m, 2H), 0.15 (s, 3H), 0.14 (s, 3H); ^{13}C NMR (100 MHz, $CDCl_3$) δ: 142.32, 128.49, 128.47, 125.93, 122.42, 35.81, 30.95, 29.77, 26.70, 17.64, 7.10, 5.47, -5.18, -5.25; ^{29}Si NMR (79 MHz, $CDCl_3$) δ: 9.17; HRMS (ESI): $[M+H]^+$ calculated for $C_{16}H_{26}NSi^+$ = 260.1835, found: 260.1833.

2-(isopropyldimethylsilyl)-6-phenylhexanenitrile (70)

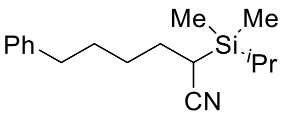

Prepared according to general procedure A using (3-bromopropyl)benzene (79.2 mg, 0.4 mmol), acrylonitrile (40.0 μL, 0.6 mmol), chloro(isopropyl)dimethylsilane (163.3 mg, 1.2 mmol), $Ni(PPh_3)_2Cl_2$ (26.4 mg, 0.04 mmol), 2-(pyridin-2-yl)-4,5-dihydrooxazole (12.0 mg, 0.08 mmol), Zn (78.0 mg, 1.2 mmol), DMA (2.0 mL) at 35 ºC for 36 h. The crude material was purified by flash chromatography (silica gel: 5% EtOAc in Petroleum ether) to provide the title compound as a colorless oil (63.2 mg, 58% yield). ^{1}H NMR (400 MHz, $CDCl_3$) δ: 7.30-7.27 (m, 2H), 7.21-7.15 (m, 3H), 2.64 (dd, J_1 = 7.9 Hz, J_2 = 3.9 Hz, 2H), 1.81 (dd, J_1 = 11.5 Hz, J_2 = 3.7 Hz, 1H), 1.74-1.54 (m, 5H), 1.52-1.44 (m, 2H), 1.02 (s, 3H), 1.00 (s, 3H), 0.14 (s, 3H), 0.10 (s, 3H); ^{13}C NMR (100 MHz, $CDCl_3$) δ: 142.28, 128.47, 128.44, 125.91, 122.46, 35.79, 30.92, 29.76, 26.80, 17.44, 17.38, 16.66, 12.20, -6.78, -6.92; ^{29}Si NMR (79 MHz, $CDCl_3$) δ: 10.60; HRMS (ESI): $[M+H]^+$ calculated for $C_{17}H_{28}NSi^+$ = 274.1991, found: 274.1992.

6-phenyl-2-(triethylsilyl)hexanenitrile (71)

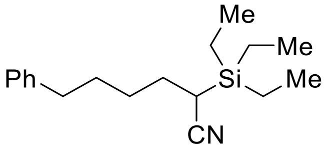

Prepared according to general procedure A using (3-bromopropyl)benzene (79.2 mg, 0.4 mmol), acrylonitrile (40.0 μL, 0.6 mmol), chlorotriethylsilane (180.8 mg, 1.2 mmol), $Ni(PPh_3)_2Cl_2$ (26.4 mg, 0.04 mmol), 2-(pyridin-2-yl)-4,5-dihydrooxazole (12.0 mg, 0.08 mmol), Zn (78.0 mg, 1.2 mmol), DMA (2.0 mL) at 50 °C for 36 h. The crude material was purified by flash chromatography (silica gel: 5% EtOAc in Petroleum ether) to provide the title compound as a colorless oil (21.9 mg, 19% yield). ^{1}H NMR (400 MHz, $CDCl_3$) δ: 7.30-7.26 (m, 2H), 7.22-7.17 (m, 3H), 2.63 (td, J_1 = 7.2 Hz, J_2 = 6.6 Hz, J_3 = 3.7 Hz, 2H), 1.82 (dd, J_1 = 11.7 Hz, J_2 = 3.6 Hz, 1H), 1.76-1.59 (m, 4H), 1.50-1.43 (m, 2H), 1.00 (d, J = 7.9 Hz, 9H), 0.71 (qd, J_1 = 8.4 Hz, J_2 = 7.9 Hz, J_3 = 3.1 Hz, 6H); ^{13}C NMR (100 MHz, $CDCl_3$) δ: 142.33, 128.50, 128.47, 125.94, 122.57, 35.83, 30.93, 29.90, 26.81, 15.31, 7.30, 2.43; ^{29}Si NMR (79 MHz, $CDCl_3$) δ: 10.48; HRMS (ESI): $[M+H]^+$ calculated for $C_{18}H_{30}NSi^+$ = 288.2148, found: 288.2148.

3.3 Gram-scale Reactions and Synthetic Applications

3.3.1 Gram-scale reaction of 4

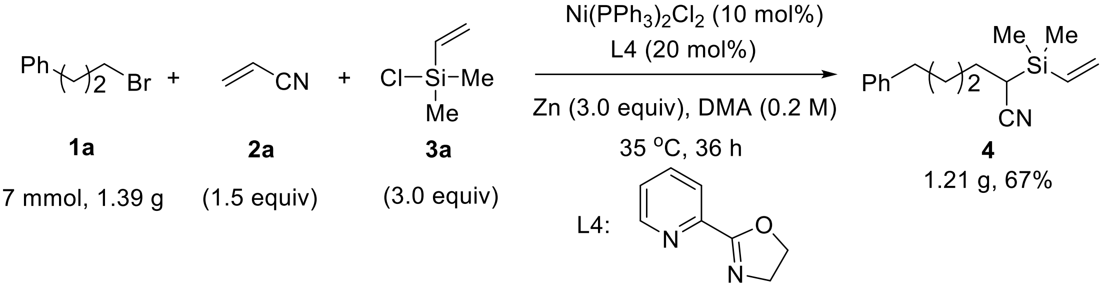

Prepared according to general procedure A using (3-bromopropyl)benzene (1.39 g, 7.0 mmol), acrylonitrile (0.70 mL, 10.5 mmol), chlorodimethyl(vinyl)silane (2.534 g, 21.0 mmol), $Ni(PPh_3)_2Cl_2$ (0.462 g, 0.7 mmol), 2-(pyridin-2-yl)-4,5-dihydrooxazole (0.210 g, 1.4 mmol), Zn (1.365 mg, 21 mmol), DMA (35.0 mL) at 35 °C for 36 h. The crude material was purified by flash chromatography (silica gel: 5% EtOAc in Petroleum ether) to provide the title compound as a colorless oil (1.21 g, 67% yield).

3.3.2 Synthetic transformations of 4

Procedure for synthesis of 72

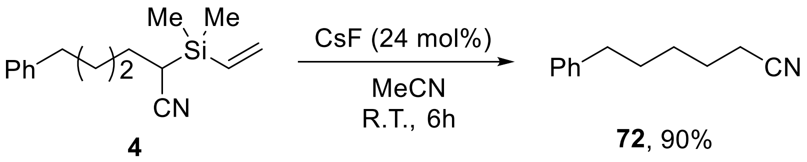

To a solution of 2-(dimethyl(vinyl)silyl)-6-phenylhexanenitrile (51.4 mg, 0.2 mmol) in MeCN (4.3 mL), solid CsF (7.1 mg, 0.048 mmol) was added. The resulting mixture was stirred at room temperature for 6 h, then diluted with aqueous saturated NH_4Cl solution and extracted with EtOAc. The organic extracts were washed with brine, dried over Na_2SO_4, filtered and concentrated. The crude material was purified by flash chromatography (silica gel: 3% EtOAc in Petroleum ether) to provide the compound as a colorless oil (31.1 mg, 90% yield). ^{1}H NMR (400 MHz, $CDCl_3$) δ: 7.30 (t, J = 7.3 Hz, 2H), 7.22-7.18 (m, 3H), 2.65 (t, J = 7.6 Hz, 2H), 2.33 (t, J = 7.1 Hz, 2H), 1.68 (dq, J_1 = 15.4, J_2 = 7.6 Hz, 4H), 1.54-1.46 (m, 2H); ^{13}C NMR (100 MHz, $CDCl_3$) δ: 142.08, 128.44, 125.94, 119.87, 35.64, 30.70, 28.33, 25.35, 17.16; HRMS (ESI): $[M+H]^+$ calculated for $C_{12}H_{16}N^+$ = 174.1283, found: 174.1283.

Procedure for synthesis of 73

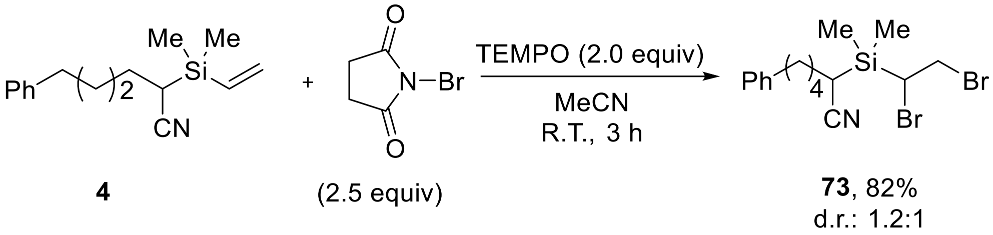

To a MeCN solution (0.4 mL) of 2-(dimethyl(vinyl)silyl)-6-phenylhexanenitrile (51.4 mg, 0.2 mmol), was added N-bromosuccinimide (90.0 mg, 0.5 mmol) and TEMPO (62.0 mg, 0.4 mmol). After the mixture was stirred for 3 h, it was concentrated in vaco. The crude material was purified by flash chromatography (silica gel: 2% Acetone in Petroleum ether) to provide the title compound as a colorless oil (69.5 mg, 83% yield, d.r.:1.2:1). ^{1}H NMR (400 MHz, $CDCl_3$) δ: 7.31 (t, J = 7.3 Hz, 2H), 7.21 (t, J = 8.4 Hz, 3H), 3.94 (td, J_1 = 10.8, J_2 = 5.8 Hz, 1H), 3.85-3.77 (m, 1H), 3.72 (dd, J_1 = 8.7, J_2 = 5.8 Hz, 0.45 H), 3.65 (dd, J_1 = 9.1, J_2 = 5.8 Hz, 0.55 H), 2.70-2.62 (m, 2H), 2.23-2.18 (m, 1H), 1.77-1.61 (m, 4H), 1.57-1.52 (m, 2H), 0.42 (s, 3H), 0.38 (s, 3H); ^{13}C NMR (100 MHz, $CDCl_3$) δ: 141.85, 128.27, 125.78, 121.00, 120.89, 38.12, 37.87, 35.49, 35.47, 34.34, 34.30, 30.56, 30.49, 29.34, 29.28, 26.33, 17.53, 17.27, -5.66, -5.77, -5.88; ^{29}Si

NMR (79 MHz, $CDCl_3$) δ: 9.06, 8.94; HRMS (ESI): $[M+H]^+$ calculated for $C_{16}H_{24}Br_2NSi^+$ = 416.0045, found: 416.0045.

Procedure for synthesis of 74

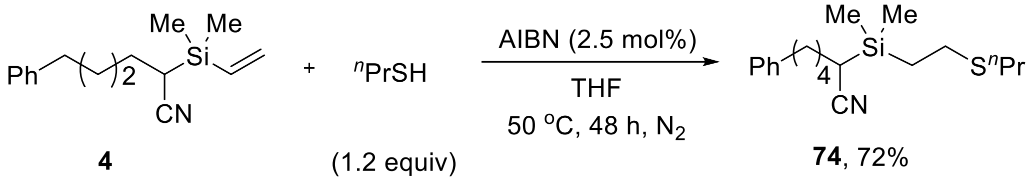

To a super dehydrated THF (1.0 mL) solution of AIBN (1.6 mg, 0.01 mmol) under N_2, were added 2-(dimethyl(vinyl)silyl)-6-phenylhexanenitrile (0.103 g, 0.4 mmol) and propane-1-thiol (36.6 mg, 0.48 mmol). The mixture was stirred at 50 °C for 48 h and then concentrated. The crude material was purified by flash chromatography (silica gel: 5% EtOAc in Petroleum ether) to provide the compound as a colorless oil (46.4 mg, 72% yield). ^{1}H NMR (400 MHz, $CDCl_3$) δ: 7.30-7.26 (m, 2H), 7.22-7.16 (m, 3H), 2.66-2.57 (m, 4H), 2.51 (t, J = 7.3 Hz, 2H), 1.84 (dd, J_1 = 11.3, J_2 = 3.8 Hz, 1H), 1.74-1.66 (m, 2H), 1.63-1.56 (m, 4H), 1.52-1.44 (m, 2H), 1.11-1.03 (m, 2H), 0.99 (t, J = 7.4 Hz, 3H), 0.21 (s, 3H), 0.20 (s, 3H); ^{13}C NMR (100 MHz, $CDCl_3$) δ: 142.18, 128.42, 125.88, 122.03, 35.72, 34.10, 30.85, 29.66, 26.80, 26.61, 22.85, 17.98, 14.26, 13.62, -4.71; ^{29}Si NMR (79 MHz, $CDCl_3$) δ: 7.41; HRMS (ESI): $[M+H]^+$ calculated for $C_{19}H_{32}NSSi^+$ = 334.2025, found: 334.2024.

3.3.3 Surface modification of glass slide[21]

Glass slide (1.5 × 1.5 cm^2) was submerged in 60 % HNO_3, and the solution was sonicated for 5 minutes. Then, the slide was treated in Piranha solution (H_2SO_4 : H_2O_2 = 7 : 3, 10 mL) at room temperature for 30 minutes. After washing with distilled water and methanol and drying in vacuum, glass slide was treated with a solution of alkylsilane **4** (2 mmol), $[(COE)_2IrCl]_2$ (22.4 mg, 5 mol%), and DMA·HCl (12.3 mg, 10 mol%) in DCM (1.0 mL). The solution was shaken for 12 hours. The glass slide was washed with DCM and methanol. After drying in vacuum, it was used for the measurement of the contact angle of the glass surface. The contact angle changed from 31° for the bare surface to 71° for the immobilized surface.

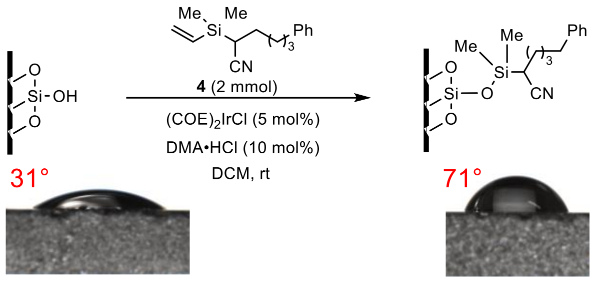

Supplementary Fig. 1. Surface modification of glass slide. Left the contact angle before surface modification. **Right** the contact angle after surface modification.

3.3.4 Gram-scale reaction of 68

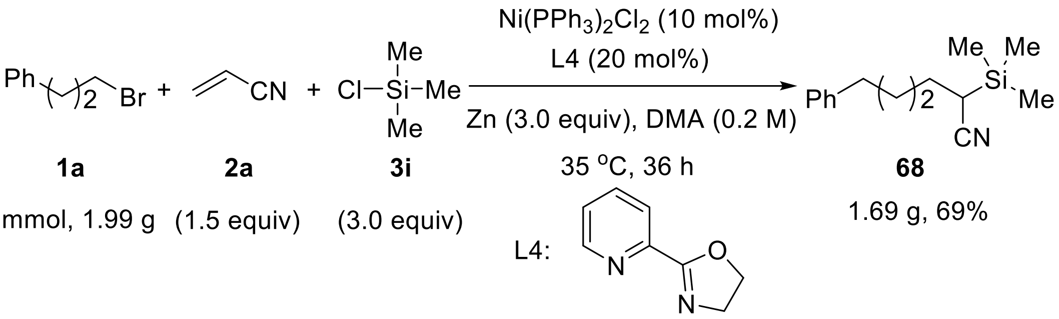

Prepared according to general procedure A using (3-bromopropyl)benzene (1.99 g, 10 mmol), acrylonitrile (1.0 mL, 15 mmol), chlorotrimethylsilane (3.260 g, 30 mmol), $Ni(PPh_3)_2Cl_2$ (0.660 g, 1.0 mmol), 2-(pyridin-2-yl)-4,5-dihydrooxazole (0.300 g, 2.0 mmol), Zn (1.950 g, 30 mmol), DMA (50.0 mL) at 35 °C for 36 h. The crude material was purified by flash chromatography (silica gel: 5% EtOAc in Petroleum ether) to provide the title compound as a colorless oil (1.69 g, 69% yield).

3.3.5 Synthetic transformations of 68

Procedure for synthesis of 75

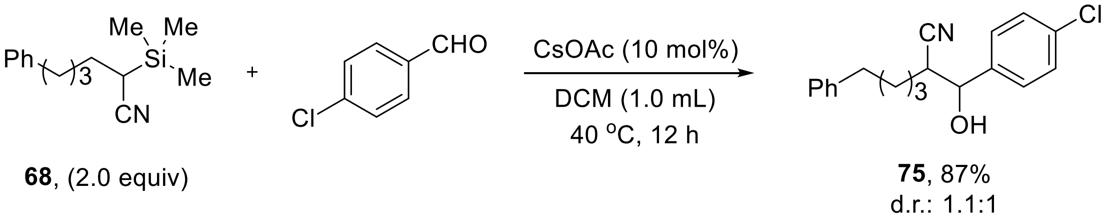

To a mixture of 4-chlorobenzaldehyde (28.1 mg, 0.2 mmol) and 6-phenyl-2-(trimethylsilyl)hexanenitrile (98.1 mg, 0.4 mmol) in DMF (1.0 mL), was added CsOAc (3.8 mg, 0.02 mmol) at 0 °C. The resulting mixture was stirred at 40 °C for 12 h, then diluted with aqueous saturated NaCl solution and extracted with DCM. The organic extracts were washed with brine, dried over Na_2SO_4, filtered and concentrated. The

crude material was purified by flash chromatography (silica gel: 15% EtOAc in Petroleum ether) affording the title compound as a colorless oil (54.9 mg, 87% yield, d.r.: 1.1:1). ^{1}H NMR (400 MHz, $CDCl_3$) δ: 7.38-7.26 (m, 6H), 7.20-7.13 (m, 3H), 4.80 (d, J = 5.8 Hz, 0.53 H), 4.75 (d, J = 5.8 Hz, 0.47 H), 2.77 (dt, J_1 = 10.2, J_2 = 5.0 Hz, 1H), 2.63-2.58 (m, 2H), 1.66-1.57 (m, 4H), 1.66-1.57 (m, 2H); ^{13}C NMR (100 MHz, $CDCl_3$) δ: 142.08, 142.02, 138.89, 138.52, 134.68, 134.66, 129.09, 128.98, 128.46, 127.91, 127.66, 125.98, 125.96, 119.97, 73.37, 73.09, 41.12, 40.49, 35.66, 35.62, 30.96, 30.90, 28.88, 27.76, 26.84; HRMS (ESI): $[M+Na]^+$ calculated for $C_{19}H_{20}ClNNaO^+$ = 336.1131, found: 336.1131.

Procedure for synthesis of 76

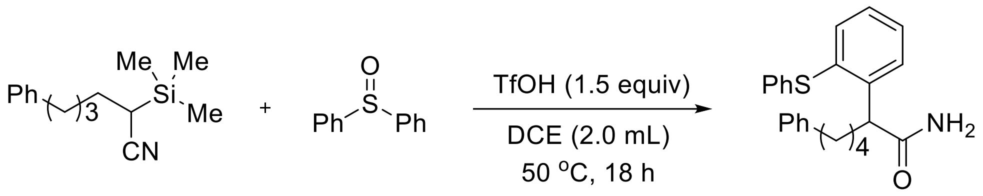

68, (2.0 equiv) **76**, 77%

To a mixture of sulfinyldibenzene (40.5 mg, 0.2 mmol) and 6-phenyl-2-(trimethylsilyl)hexanenitrile (98.1 mg, 0.4 mmol) in DCE (2.0 mL), was added TfOH (45.0 mg, 0.3 mmol) at 0 °C stirring for 10 min. After that, the mixture was warmed to 50 °C for 18 h. To the mixture was added saturated aqueous $NaHCO_3$ solution (5.0 mL). The mixture was then extracted with DCM (5.0 mL×3). The combined extracts were dried over Na_2SO_4, concentrated under reduced pressure. The resulting residue was purified by silica gel chromatography (silica gel: 30% EtOAc in Petroleum ether) affording the title compound as a white solid (57.8 mg, 77% yield), mp 87-88 °C. ^{1}H NMR (400 MHz, $CDCl_3$) δ: 7.57 (dd, J_1 = 7.8, J_2 = 1.2 Hz, 1H), 7.47 (dd, J_1 = 7.7, J_2 = 1.3 Hz, 1H), 7.37 (td, J_1 = 8.9, J_2 = 7.6, J_3 = 1.3 Hz, 1H), 7.28-7.22 (m, 5H), 7.20-7.11 (m, 6H), 5.64 (s, 1H), 5.27 (s, 1H), 4.14 (t, J = 7.4 Hz, 1H), 2.52 (t, J = 7.8 Hz, 2H), 2.19-2.10 (m, 1H), 1.71-1.64 (m, 1H), 1.60-1.51 (m, 2H), 1.35-1.30 (m, 1H), 1.22-1.12 (m, 1H); ^{13}C NMR (100 MHz, $CDCl_3$) δ: 175.54, 142.74, 142.65, 137.02, 135.22, 132.79, 129.58, 129.42, 129.07, 128.46, 128.36, 128.31, 128.04, 126.66, 125.69, 48.14, 35.74, 32.95, 31.34, 27.32; HRMS (ESI): $[M+H]^+$ calculated for $C_{24}H_{26}NOS^+$ = 376.1735, found: 376.1760.

Procedure for synthesis of 77

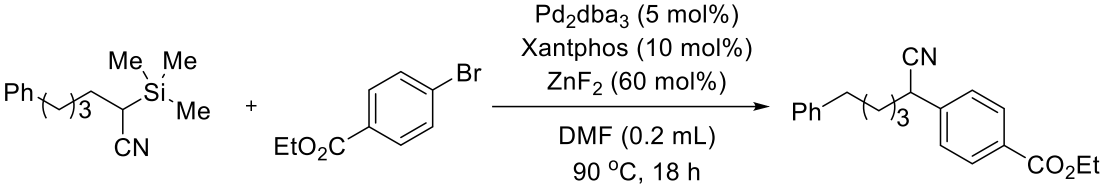

68, (1.5 equiv) **77**, 72%

To a solution of Ethyl 4-bromobenzoate (45.8 mg, 0.2 mmol), 6-phenyl-2-(trimethylsilyl)hexanenitrile (74.0 mg, 0.3 mmol), Pd_2dba_3 (9.0 mg, 0.01 mmol), Xantphos (11.5 mg, 0.02 mmol), and ZnF_2 (12.4 mg, 0.12 mmol) in DMF (0.2 mL) under nitrogen. The resulting mixture was stirred at 90 °C for 18 h, then diluted with aqueous saturated NH_4Cl solution and extracted with DCM. The organic extracts were washed with brine, dried over Na_2SO_4, filtered and concentrated. The crude material was purified by flash chromatography (silica gel: 3% DCM and 30% EtOAc in Petroleum ether) to provide the title compound as a colorless oil (46.4 mg, 72% yield). ^{1}H NMR (400 MHz, $CDCl_3$) δ: 8.06 (d, J = 8.4 Hz, 2H), 7.39 (d, J = 8.3 Hz, 2H), 7.29 (d, J = 7.2 Hz, 2H), 7.23-7.12 (m, 3H), 4.39 (q, J = 7.1 Hz, 2H), 3.83 (dd, J_1 = 8.5 Hz, J_2 = 6.2 Hz, 1H), 2.61 (t, J = 7.6 Hz, 2H), 2.01-1.84 (m, 2H), 1.70-1.61 (m, 2H), 1.59-1.48 (m, 2H), 1.41 (t, J = 7.1 Hz, 3H); ^{13}C NMR (100 MHz, $CDCl_3$) δ: 166.06, 141.97, 140.75, 130.44, 128.49, 128.44, 127.38, 126.00, 120.32, 61.30, 37.40, 35.70, 35.64, 30.86, 26.70, 14.42; HRMS (ESI): $[M+H]^+$ calculated for $C_{21}H_{24}NO_2^+$ = 322.1807, found: 322.1807.

3.4 Mechanistic Studies

3.4.1 Radical-trapping experiment

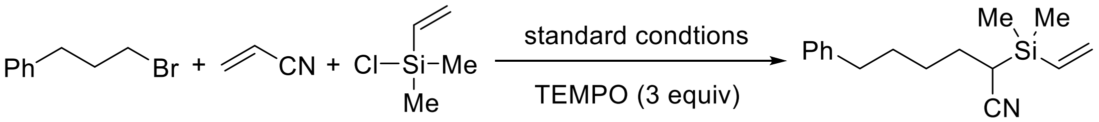

1a **2a** **3a** **4,** 0% yield

Prepared according to general procedure A using (3-bromopropyl)benzene (39.6 mg, 0.2 mmol), acrylonitrile (20.0 μL, 0.3 mmol), chlorodimethyl(vinyl)silane (72.4 mg, 0.6 mmol), $Ni(PPh_3)_2Cl_2$ (13.2 mg, 0.02 mmol), 2-(pyridin-2-yl)-4,5-dihydrooxazole (6.0 mg, 0.04 mmol), Zn (39.0 mg, 0.6 mmol), TEMPO (93.8 mg, 0.6 mmol), DMA (1.0 mL) at 35 °C for 36 h, subsequently quenched with water (10.0 mL) and extracted with dichloromethane (3 × 15.0 mL). The combined organic layers were washed with water, brine, dried over anhydrous Na_2SO_4, and concentrated under reduced pressure.

In this reaction system, no target product was detected by ^{1}H NMR analysis with dibromomethane (14.0 μL, 0.2 mmol) as an internal standard.

3.4.2 Radical-clock experiment

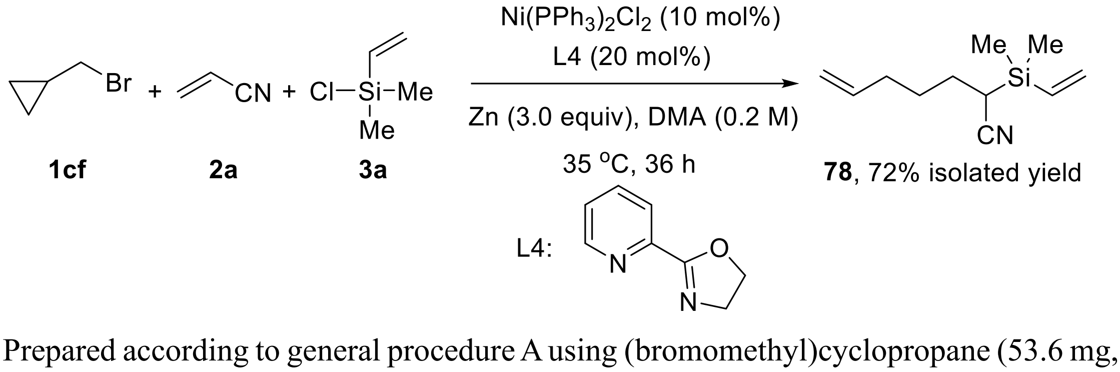

Prepared according to general procedure A using (bromomethyl)cyclopropane (53.6 mg, 0.4 mmol), acrylonitrile (40.0 μL, 0.6 mmol), chlorodimethyl(vinyl)silane (144.8 mg, 1.2 mmol), $Ni(PPh_3)_2Cl_2$ (26.4 mg, 0.04 mmol), 2-(pyridin-2-yl)-4,5-dihydrooxazole (12.0 mg, 0.08 mmol), Zn (78.0 mg, 1.2 mmol), DMA (2.0 mL) at 35 °C for 36 h. The crude material was purified by flash chromatography (silica gel: 5% EtOAc in Petroleum ether) to provide the title compound as a colorless oil (56.0 mg, 72% yield). ^{1}H NMR (400 MHz, $CDCl_3$) δ: 6.14-6.07 (m, 2H), 5.87-5.72 (m, 2H), 5.04-4.96 (m, 2H), 2.08 (dt, J_1 = 14.1 Hz, J_2 = 6.7 Hz, 2H), 1.81 (dd, J_1 = 10.7 Hz, J_2 = 4.4 Hz, 1H), 1.78-1.71 (m, 1H), 1.57-1.47 (m, 3H), 0.26 (s, 6H); ^{13}C NMR (100 MHz, $CDCl_3$) δ: 137.85, 135.41, 134.10, 122.04, 115.30, 32.99, 28.98, 26.13, 18.20, -4.82, -5.16; ^{29}Si NMR (79 MHz, $CDCl_3$) δ: -1.57; HRMS (ESI): $[M+H]^+$ calculated for $C_{11}H_{20}NSi^+$ = 194.1365, found: 194.1365.

3.4.3 Control experiments

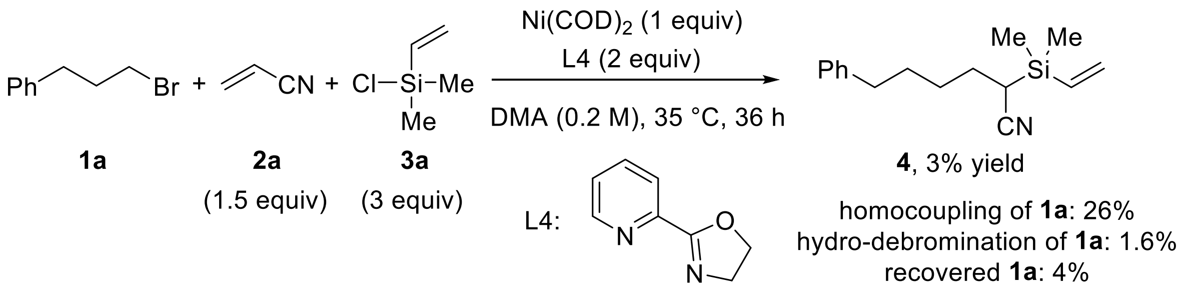

Prepared according to general procedure A using (3-bromopropyl)benzene (19.8 mg, 0.1 mmol), acrylonitrile (10.0 μL, 0.15 mmol), chlorodimethyl(vinyl)silane (36.2 mg, 0.3 mmol), $Ni(COD)_2$ (28.0 mg, 0.1 mmol), 2-(pyridin-2-yl)-4,5-dihydrooxazole (29.6 mg, 0.2 mmol), Zn (19.5 mg, 0.3 mmol), DMA (0.5 mL) at 35 °C for 36 h, subsequently quenched with water (10.0 mL) and extracted with dichloromethane (3 × 15.0 mL). The

combined organic layers were washed with water, brine, dried over anhydrous Na_2SO_4, and concentrated under reduced pressure. the yield of **4** was determined by ^{1}H NMR analysis with dibromomethane (7.0 μL, 0.1 mmol) as an internal standard. Homocoupling of **1a**: 26%, hydro-debromination of **1a**: 1.6% and recovered **1a**: 4% were determined by GC analysis with dodecane as an internal standard.

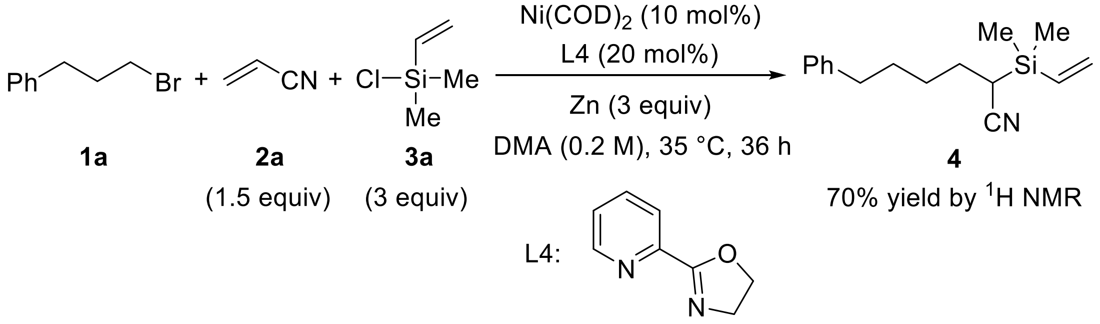

Prepared according to general procedure A using (3-bromopropyl)benzene (39.6 mg, 0.2 mmol), acrylonitrile (20.0 μL, 0.3 mmol), chlorodimethyl(vinyl)silane (72.4 mg, 0.6 mmol), $Ni(COD)_2$ (5.6 mg, 0.02 mmol), 2-(pyridin-2-yl)-4,5-dihydrooxazole (6.0 mg, 0.04 mmol), Zn (39.0 mg, 0.6 mmol), DMA (1.0 mL) at 35 °C for 36 h, subsequently quenched with water (10.0 mL) and extracted with dichloromethane (3 × 15.0 mL). The combined organic layers were washed with water, brine, dried over anhydrous Na_2SO_4, and concentrated under reduced pressure. In this reaction system, the yield of **4** was determined by ^{1}H NMR analysis with dibromomethane (14.0 μL, 0.2 mmol) as an internal standard.

3.5 Copies of NMR Spectra

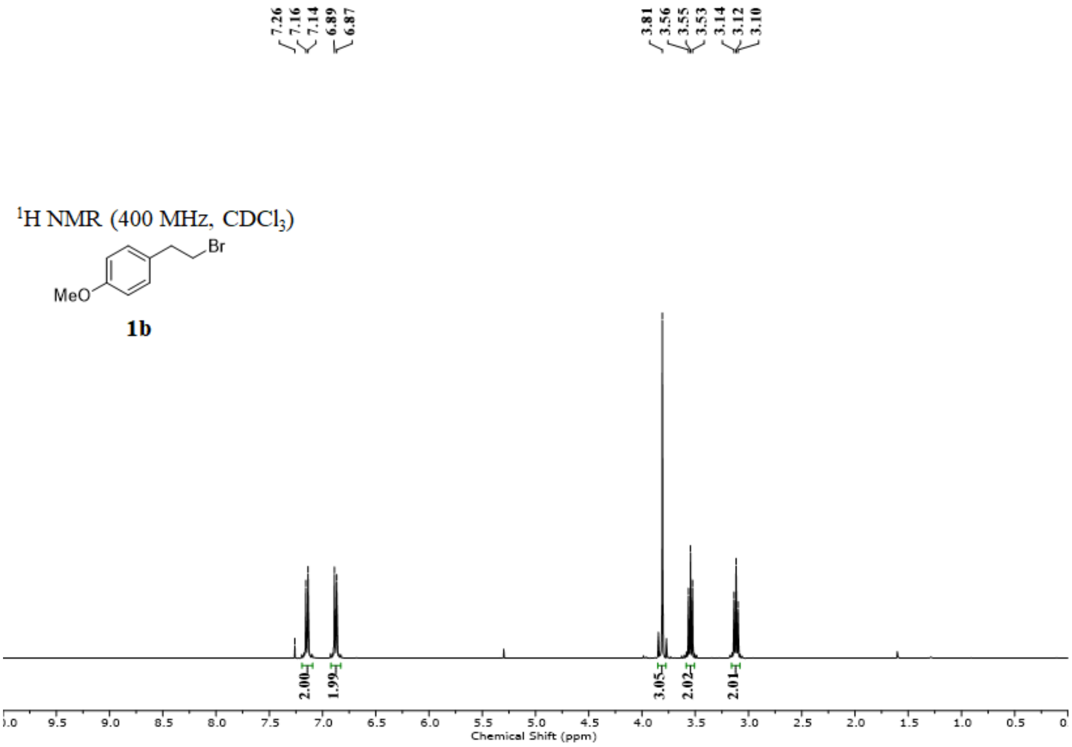

Supplementary Fig. 2. ^{1}H NMR of compound 1b. The sample has been recorded in 400 MHz, $CDCl_3$ at 25 °C

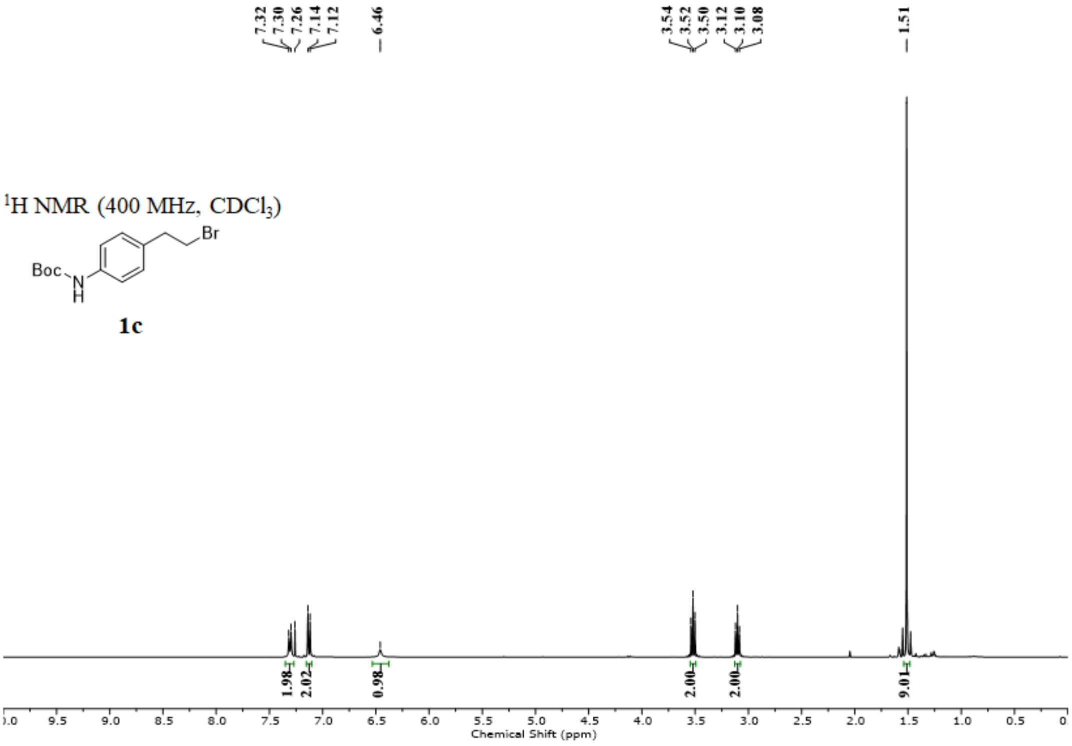

Supplementary Fig. 3. ^{1}H NMR of compound 1c. The sample has been recorded in 400 MHz, $CDCl_3$ at 25 °C

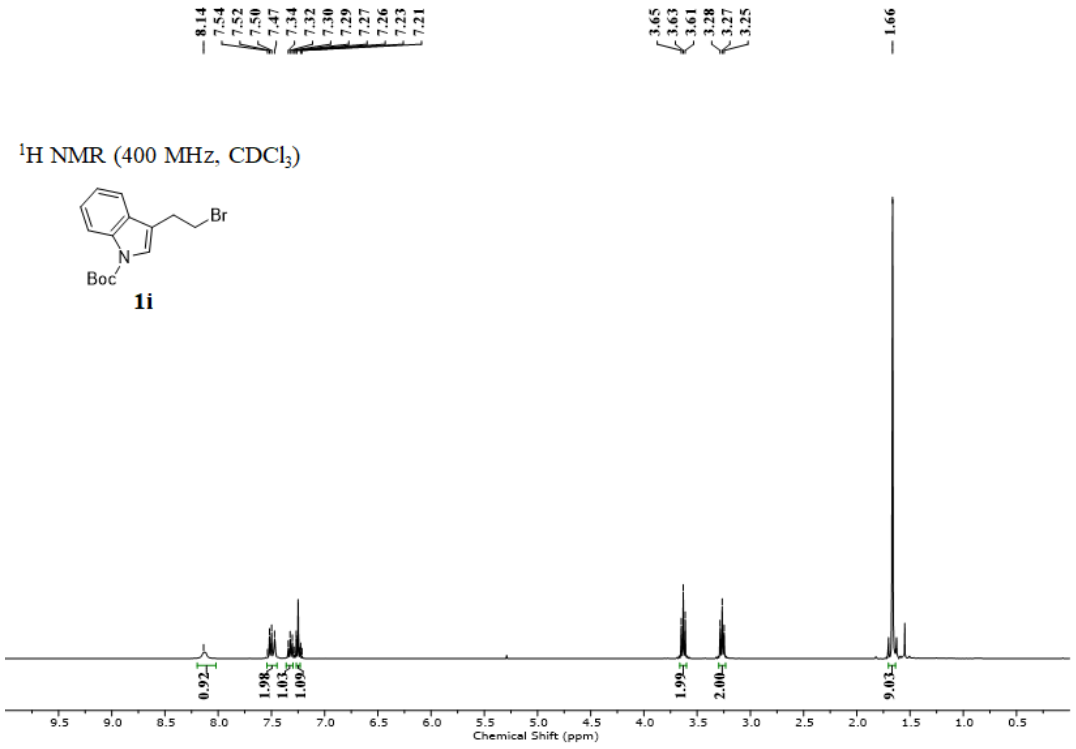

Supplementary Fig. 4. ^{1}H NMR of compound 1i. The sample has been recorded in 400 MHz, $CDCl_3$ at 25 °C

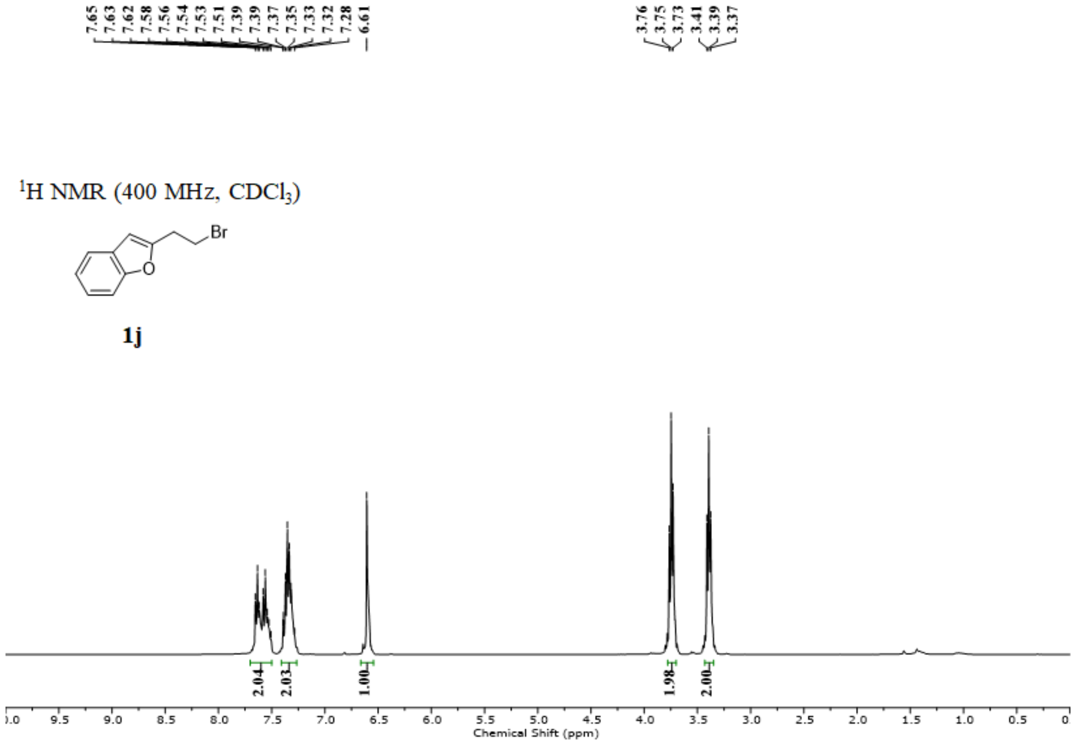

Supplementary Fig. 5. ^{1}H NMR of compound 1b. The sample has been recorded in 400 MHz, $CDCl_3$ at 25 °C

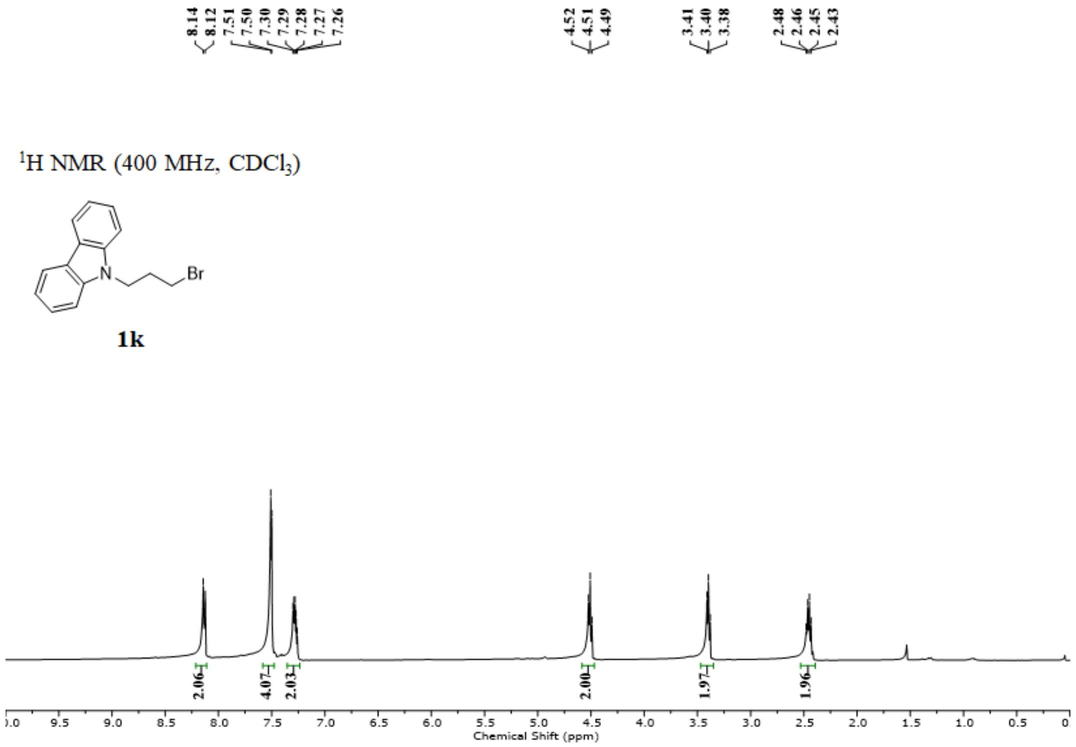

Supplementary Fig. 6. ^{1}H NMR of compound 1k. The sample has been recorded in 400 MHz, $CDCl_3$ at 25 °C

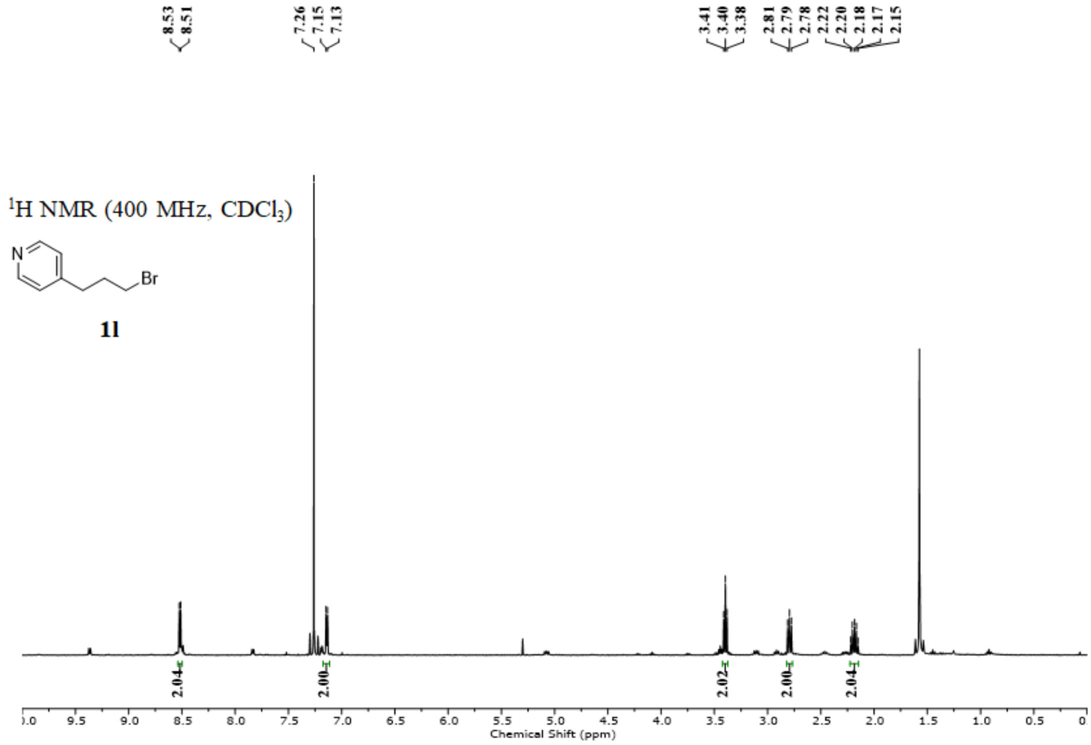

Supplementary Fig. 7. ^{1}H NMR of compound 1l. The sample has been recorded in 400 MHz, $CDCl_3$ at 25 °C

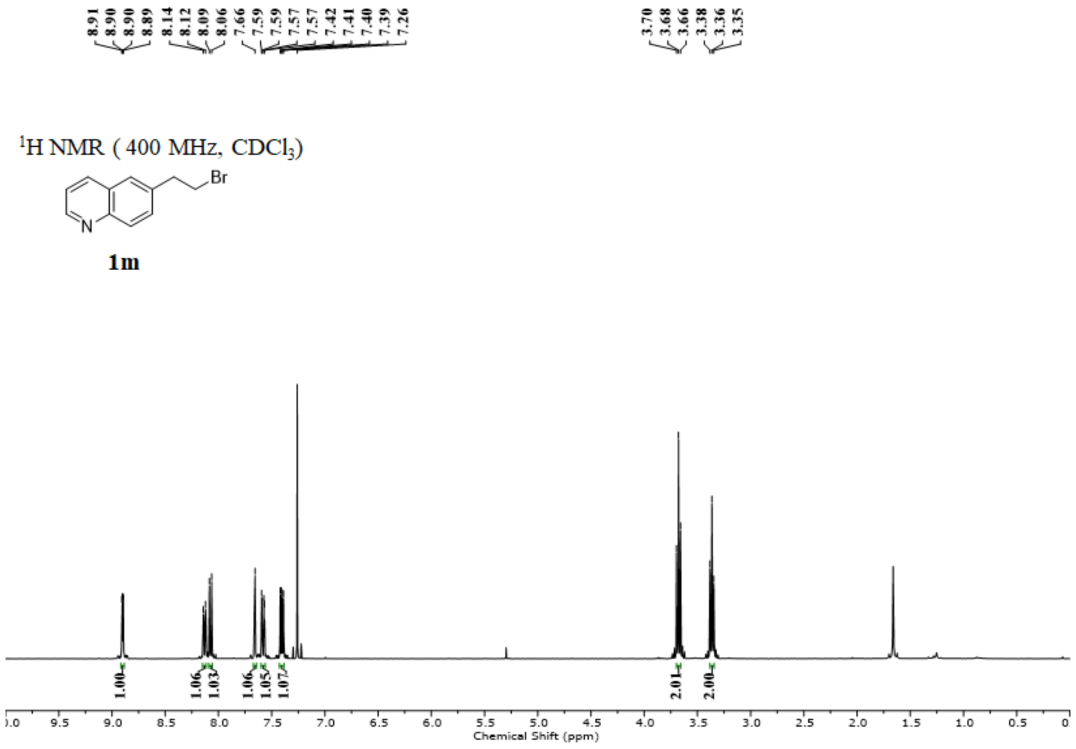

Supplementary Fig. 8. ^{1}H NMR of compound 1m. The sample has been recorded in 400 MHz, $CDCl_3$ at 25 °C

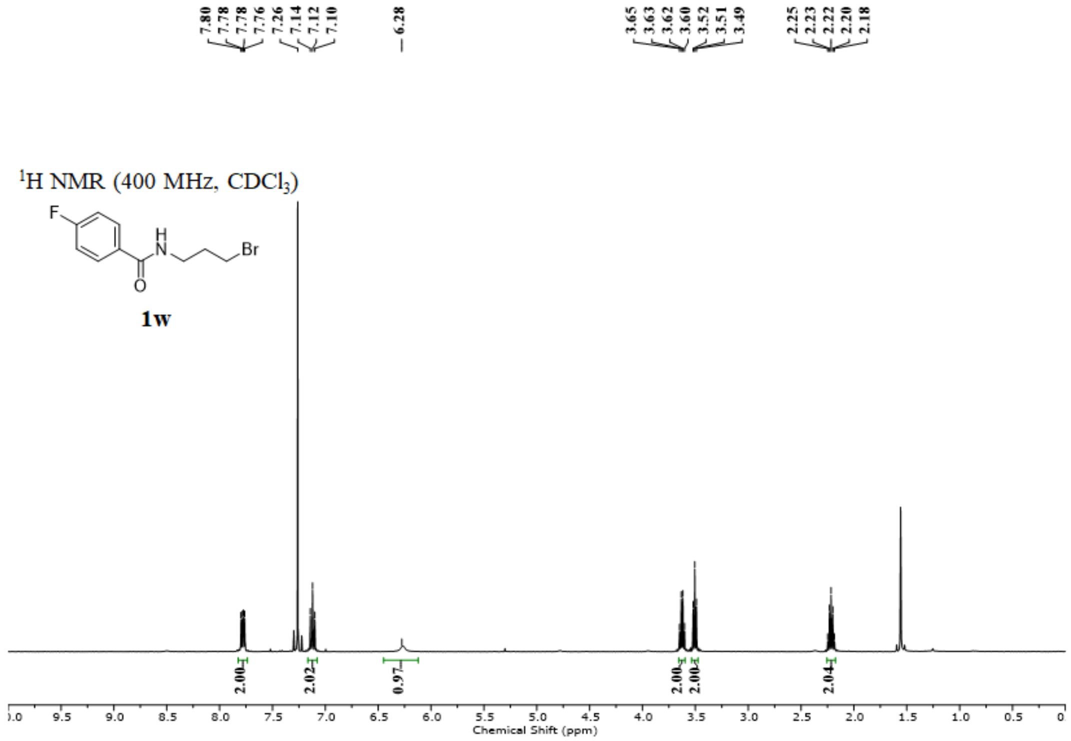

Supplementary Fig. 9. ^{1}H NMR of compound 1w. The sample has been recorded in 400 MHz, $CDCl_3$ at 25 °C

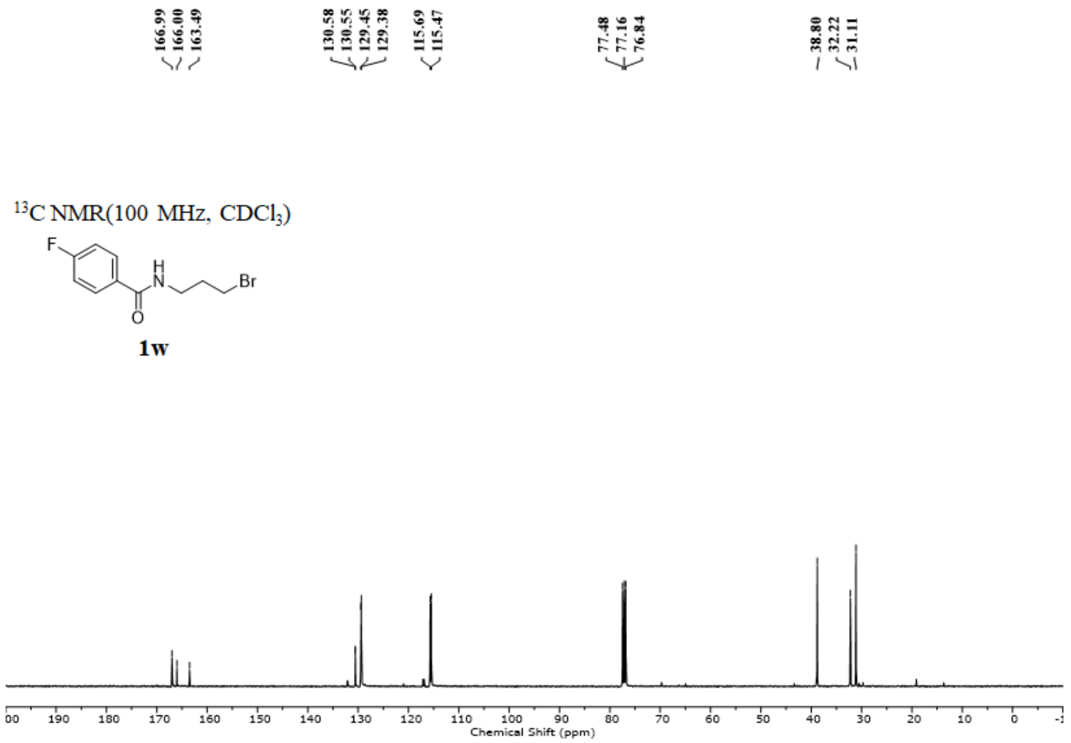

Supplementary Fig. 10. ^{13}C NMR of compound 1w. The sample has been recorded in 100 MHz, CDCl3 at 25 °C

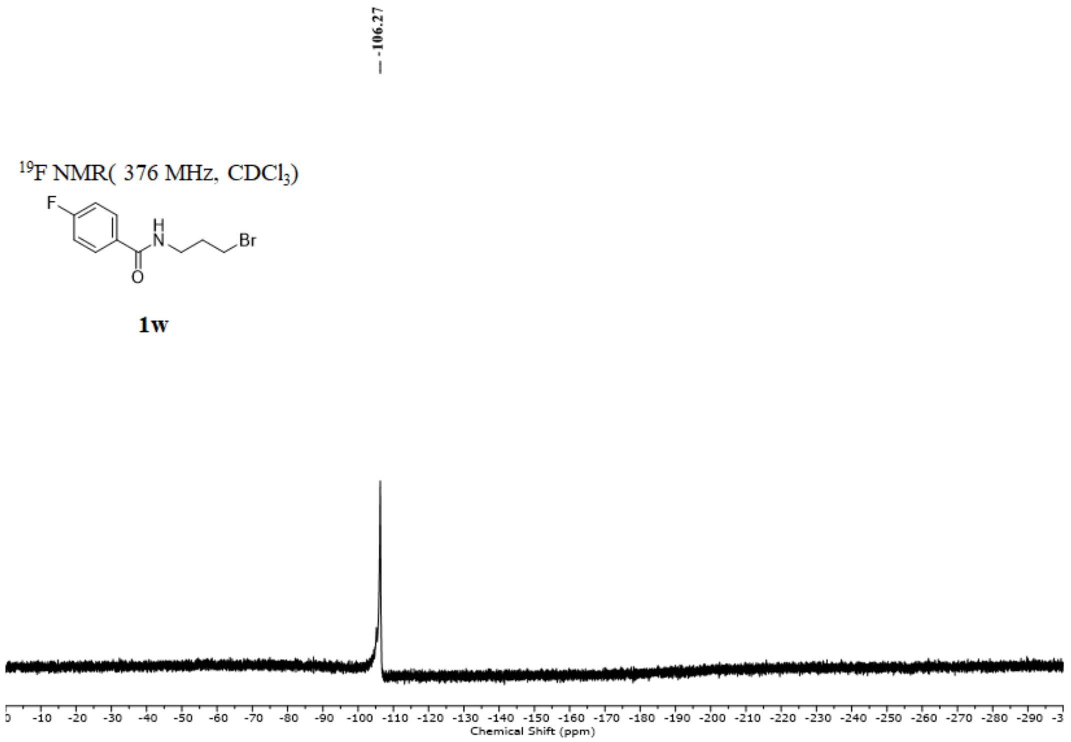

Supplementary Fig. 11. ^{19}F NMR of compound 1w. The sample has been recorded in 376 MHz, CDCl3 at 25 °C

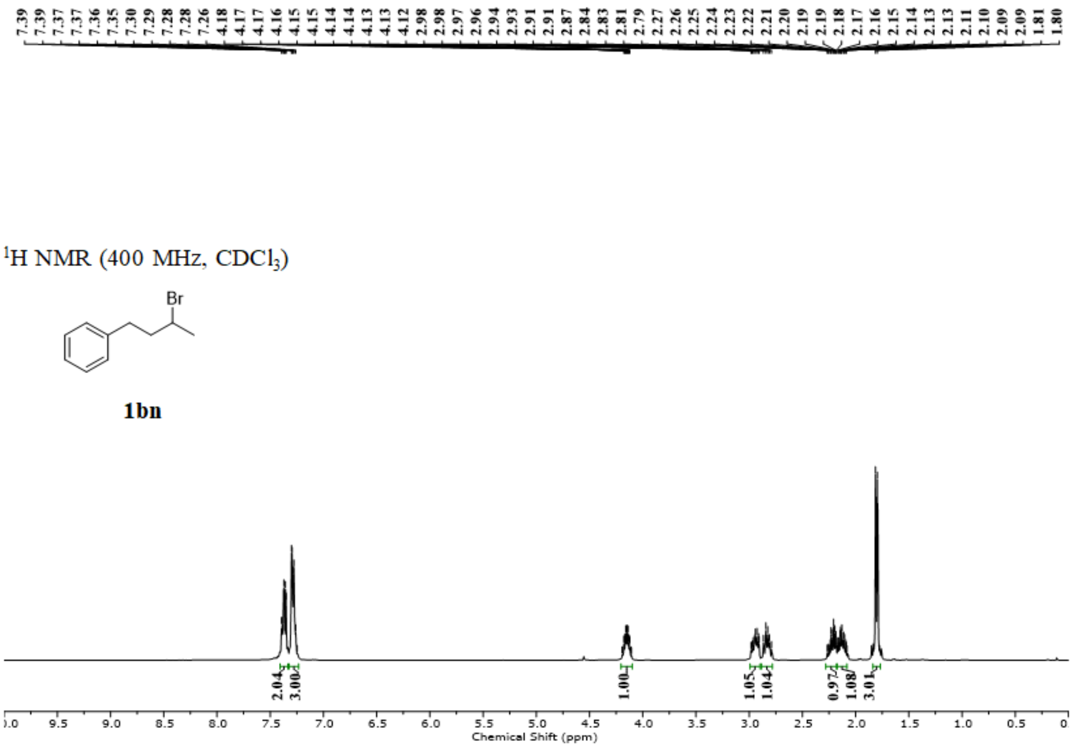

Supplementary Fig. 12. ^{1}H NMR of compound 1bn. The sample has been recorded in 400 MHz, $CDCl_3$ at 25 °C

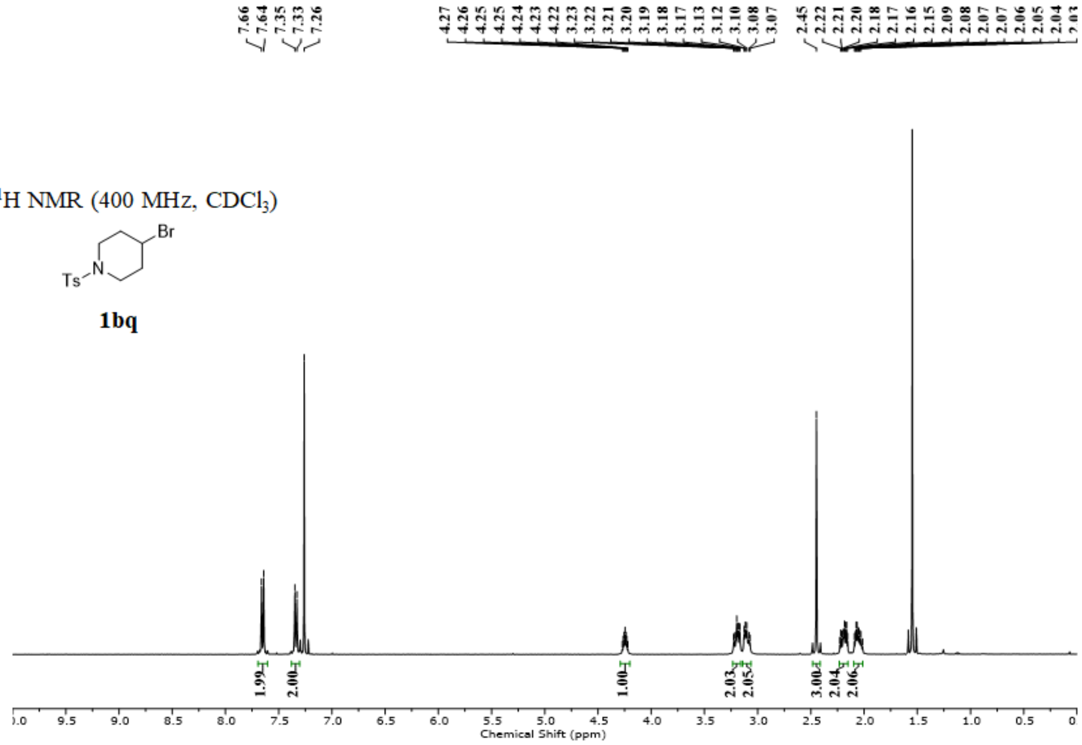

Supplementary Fig. 13. ^{1}H NMR of compound 1bq. The sample has been recorded in 400 MHz, $CDCl_3$ at 25 °C

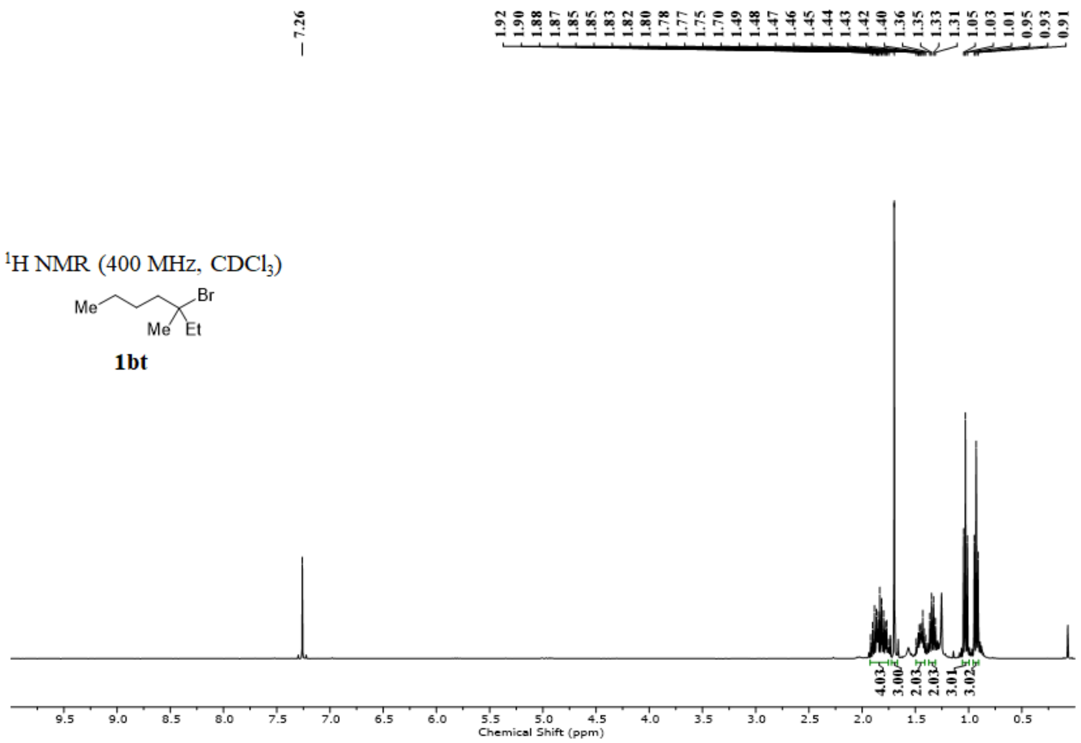

Supplementary Fig. 14. **^{1}H NMR of compound 1t.** The sample has been recorded in 400 MHz, $CDCl_3$ at 25 °C

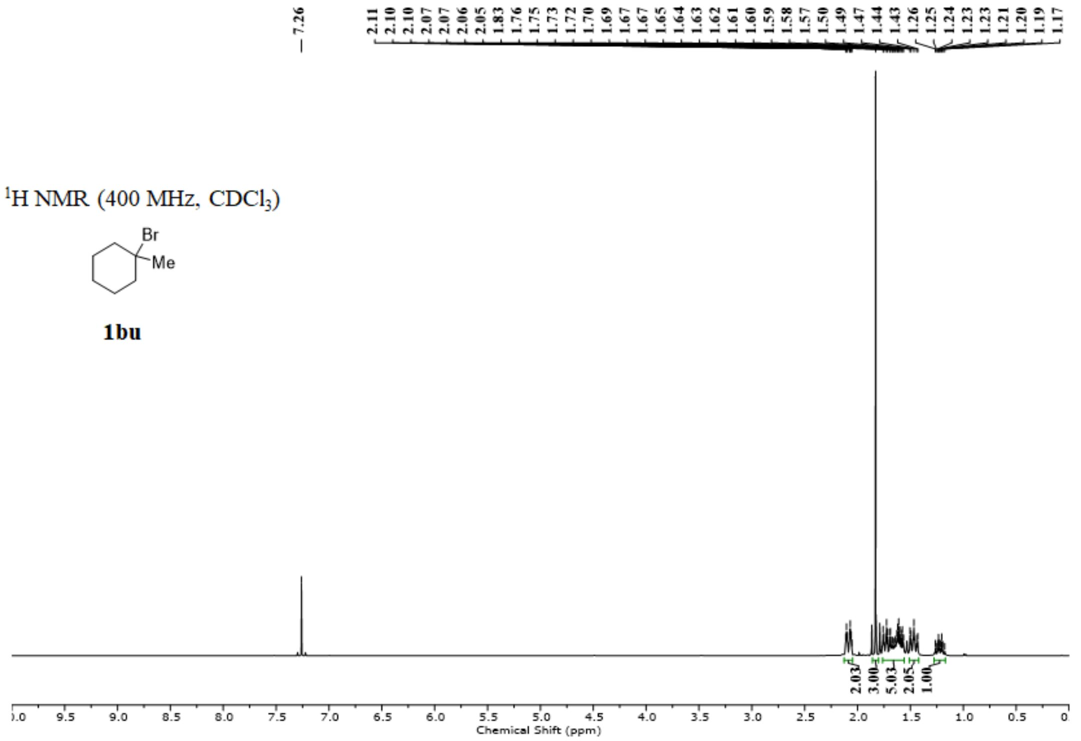

Supplementary Fig. 15. ^{1}H NMR of compound 1bu. The sample has been recorded in 400 MHz, $CDCl_3$ at 25 °C

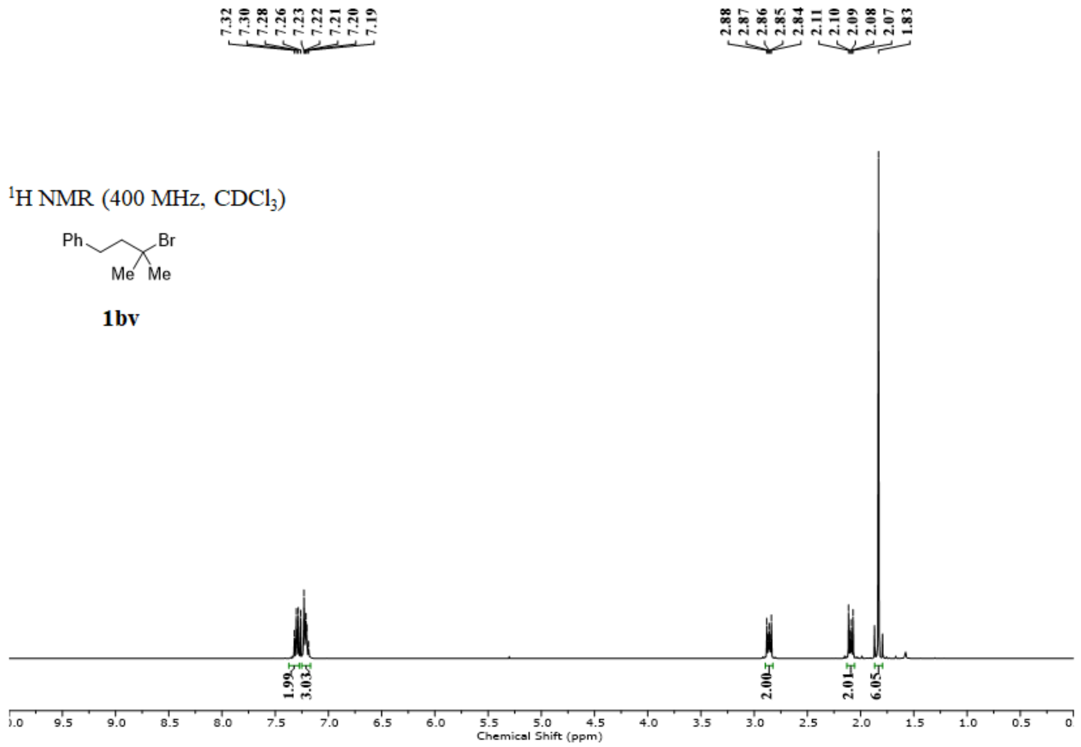

Supplementary Fig. 16. ^{1}H NMR of compound 1bv. The sample has been recorded in 400 MHz, $CDCl_3$ at 25 °C

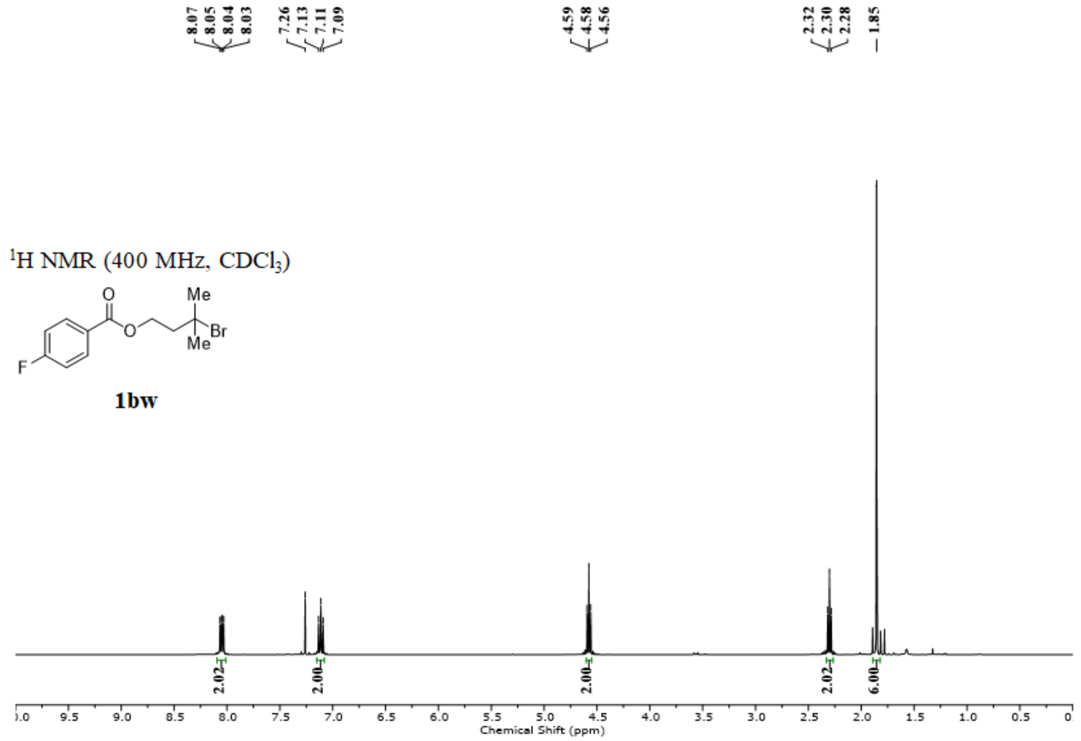

Supplementary Fig. 17. ^{1}H NMR of compound 1bw. The sample has been recorded in 400 MHz, $CDCl_3$ at 25 °C

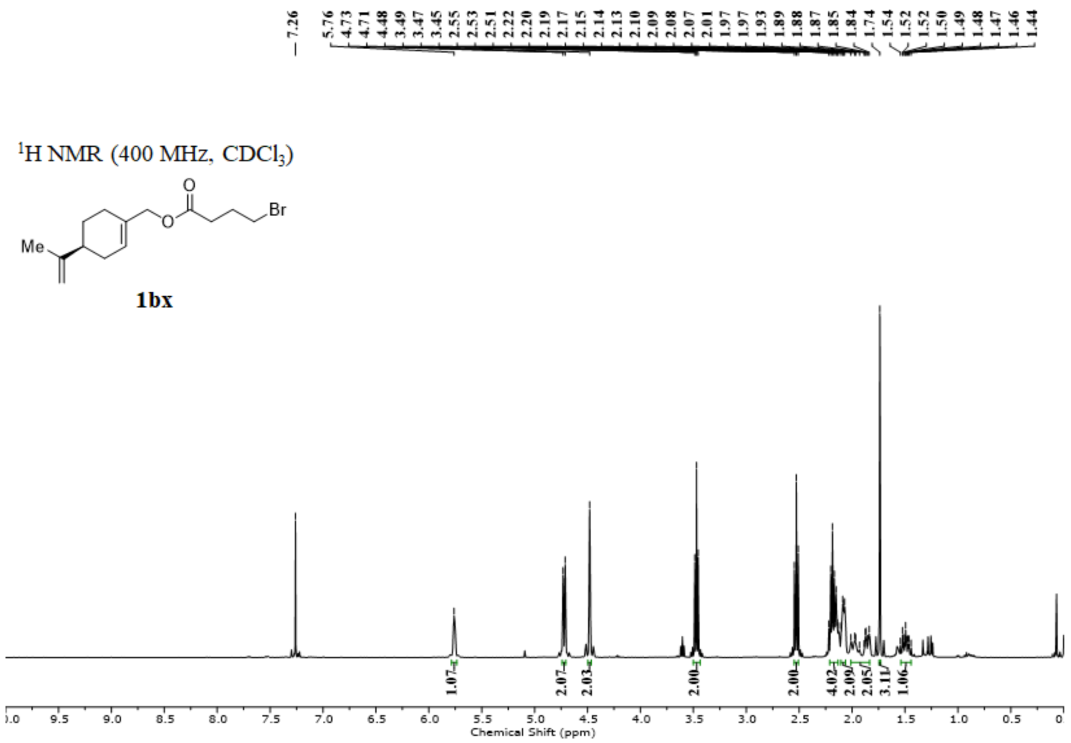

Supplementary Fig. 18. ^{1}H NMR of compound 1bx. The sample has been recorded in 400 MHz, $CDCl_3$ at 25 °C

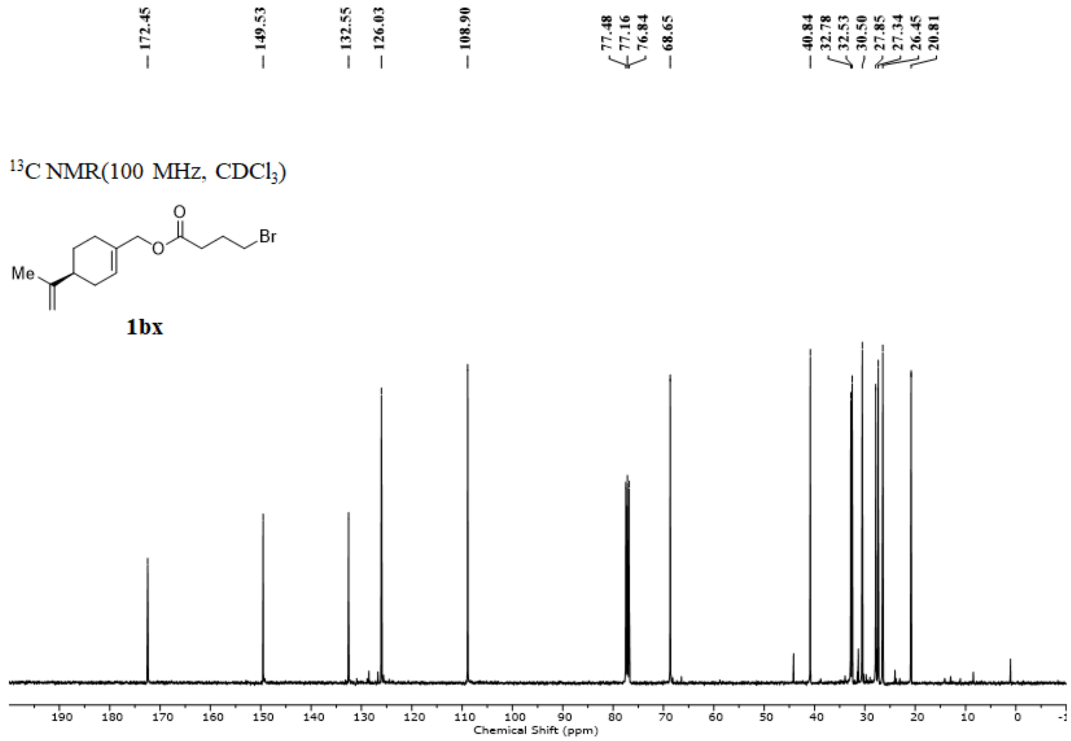

Supplementary Fig. 19. ^{13}C NMR of compound 1bx. The sample has been recorded in 100 MHz, $CDCl_3$ at 25 °C

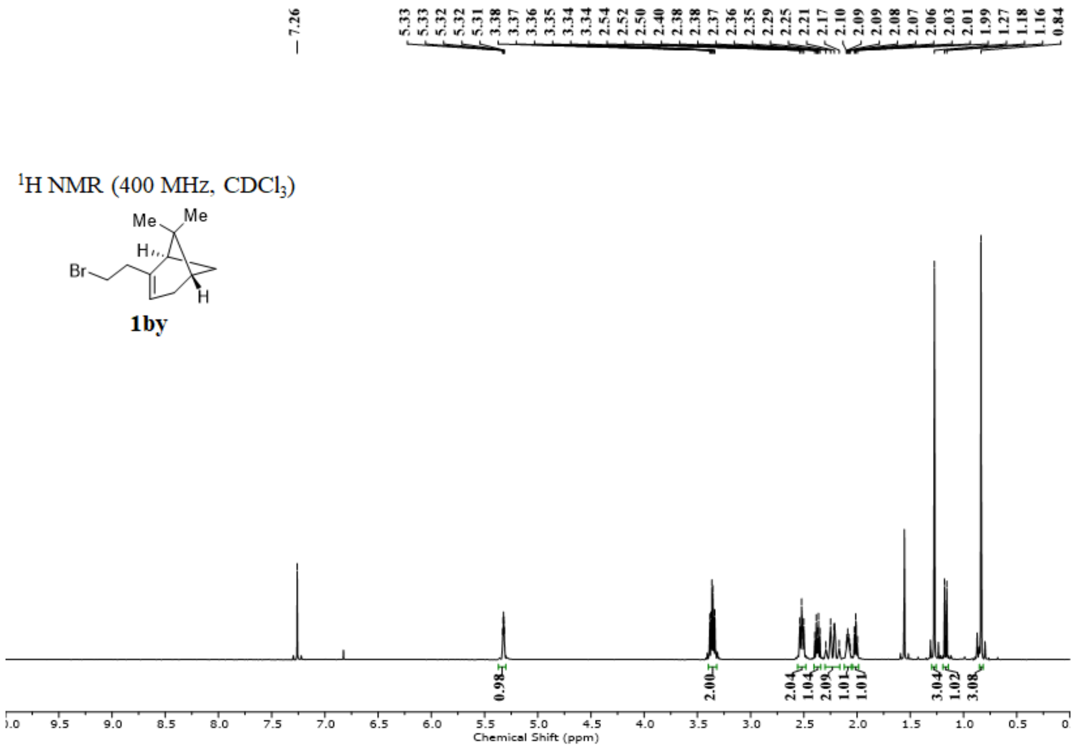

Supplementary Fig. 20. ^{1}H NMR of compound 1by. The sample has been recorded in 400 MHz, $CDCl_3$ at 25 °C

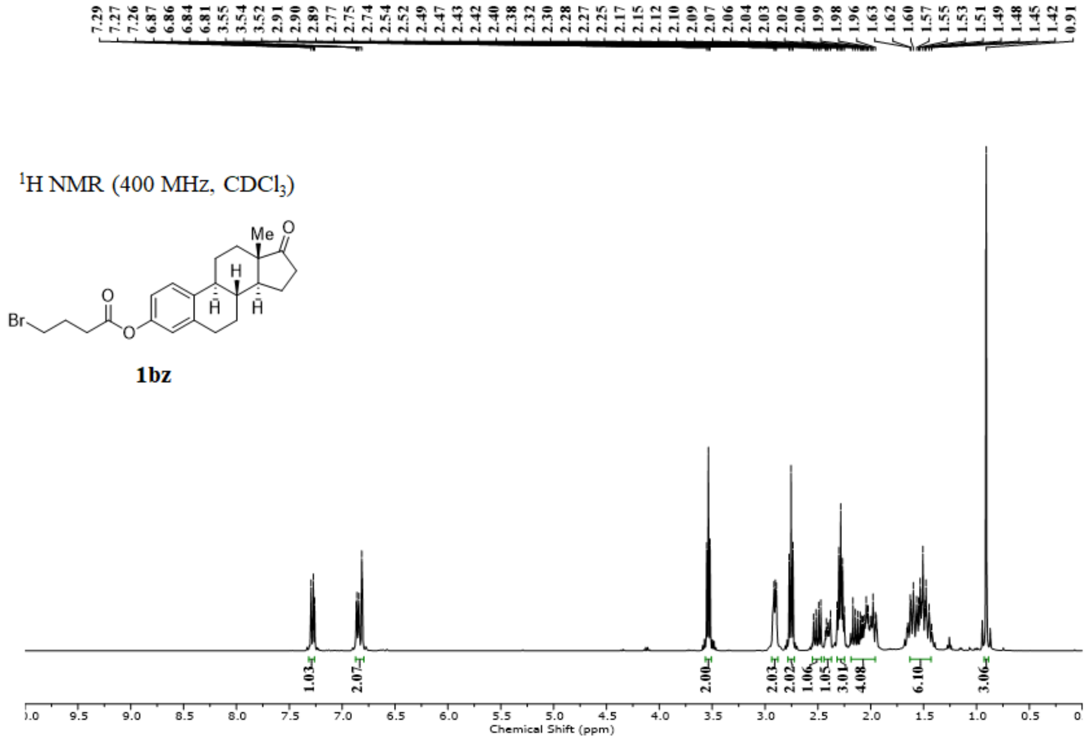

Supplementary Fig. 21. ^{1}H NMR of compound 1bz. The sample has been recorded in 400 MHz, $CDCl_3$ at 25 °C

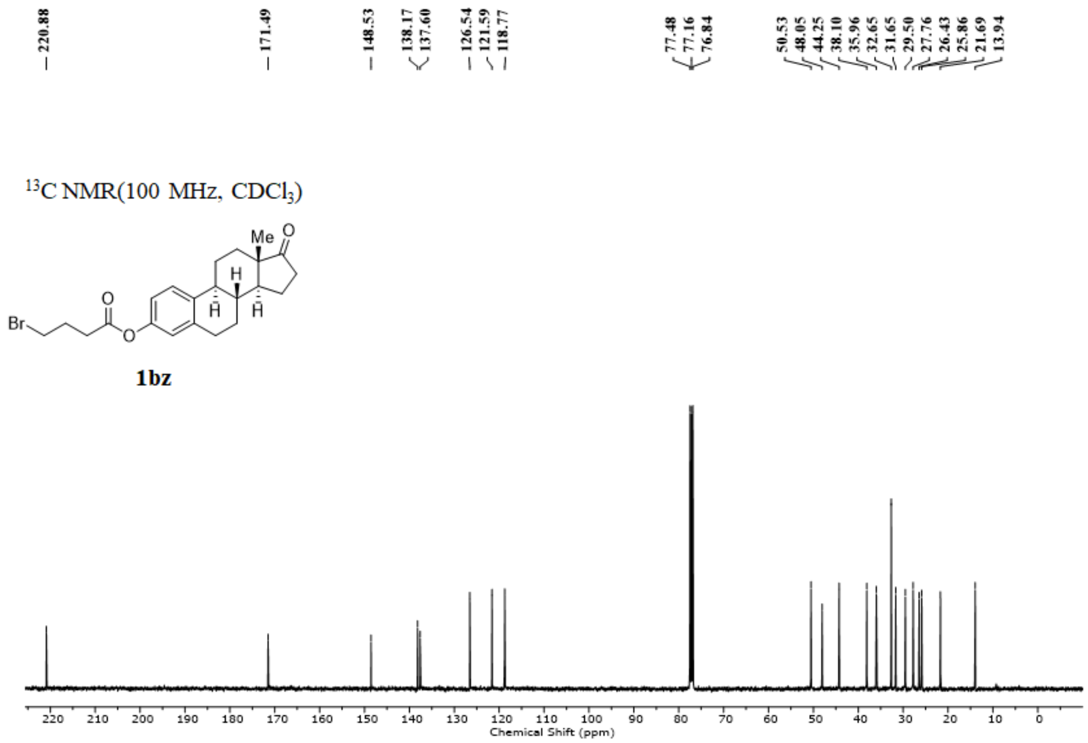

Supplementary Fig. 22. ^{13}C NMR of compound 1bz. The sample has been recorded in 100 MHz, $CDCl_3$ at 25 °C

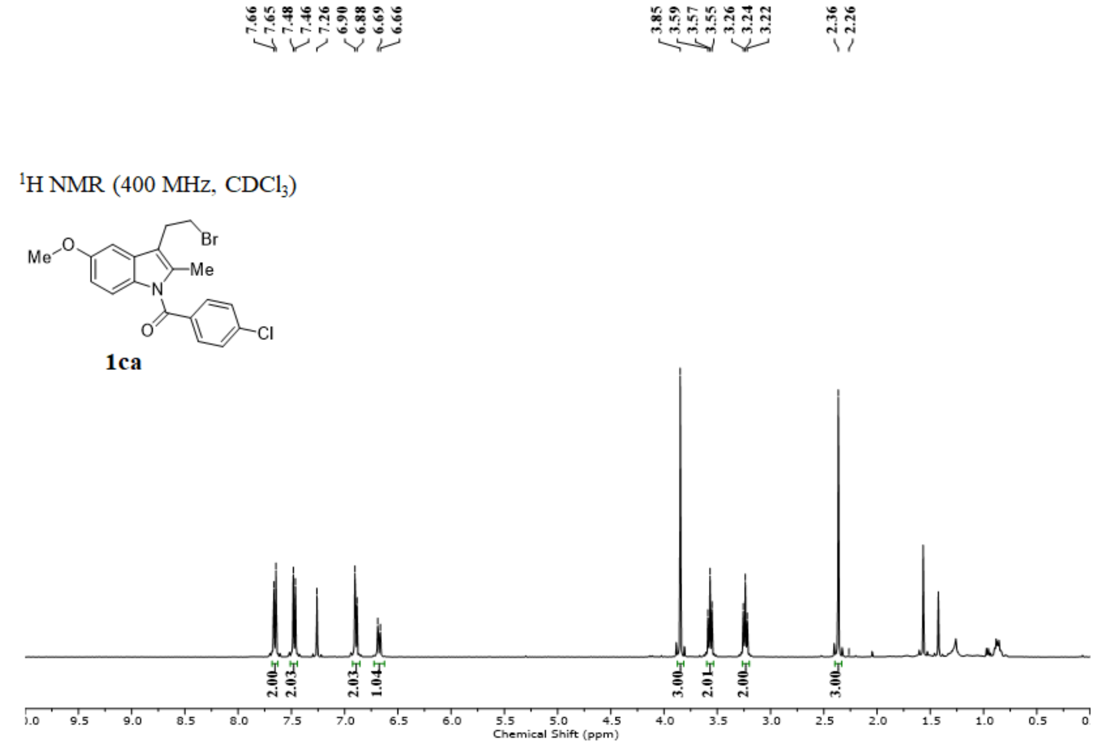

Supplementary Fig. 23. ^{1}H NMR of compound 1ca. The sample has been recorded in 400 MHz, $CDCl_3$ at 25 °C

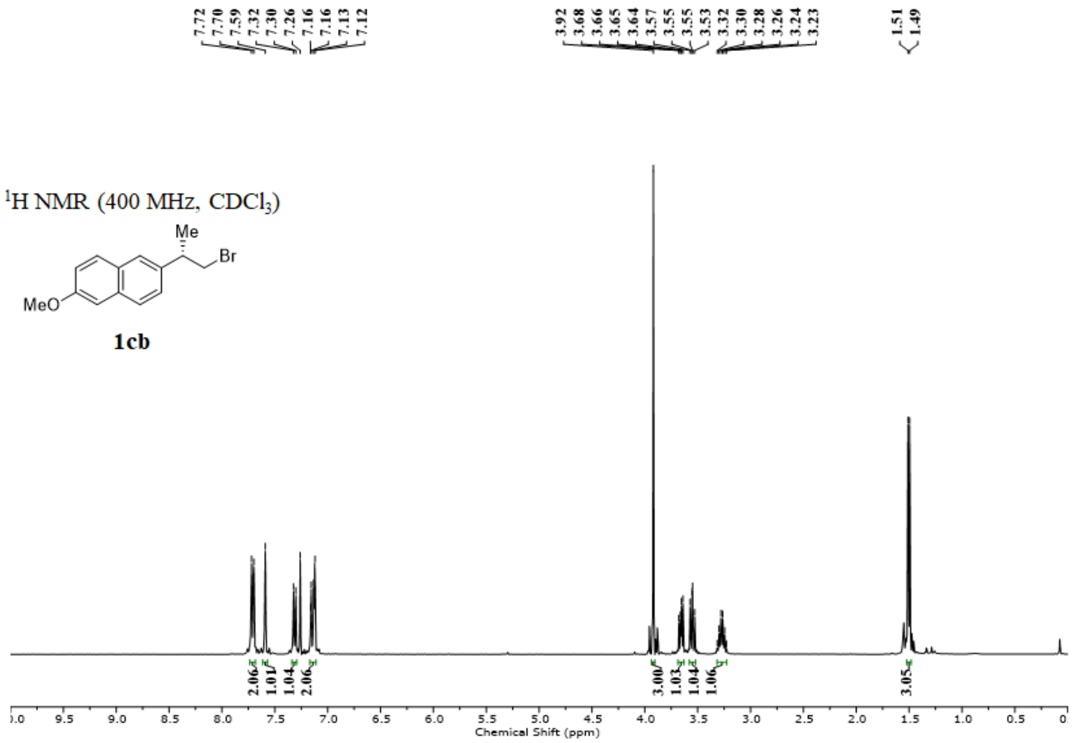

Supplementary Fig. 24. ^{1}H NMR of compound 1cb. The sample has been recorded in 400 MHz, $CDCl_3$ at 25 °C

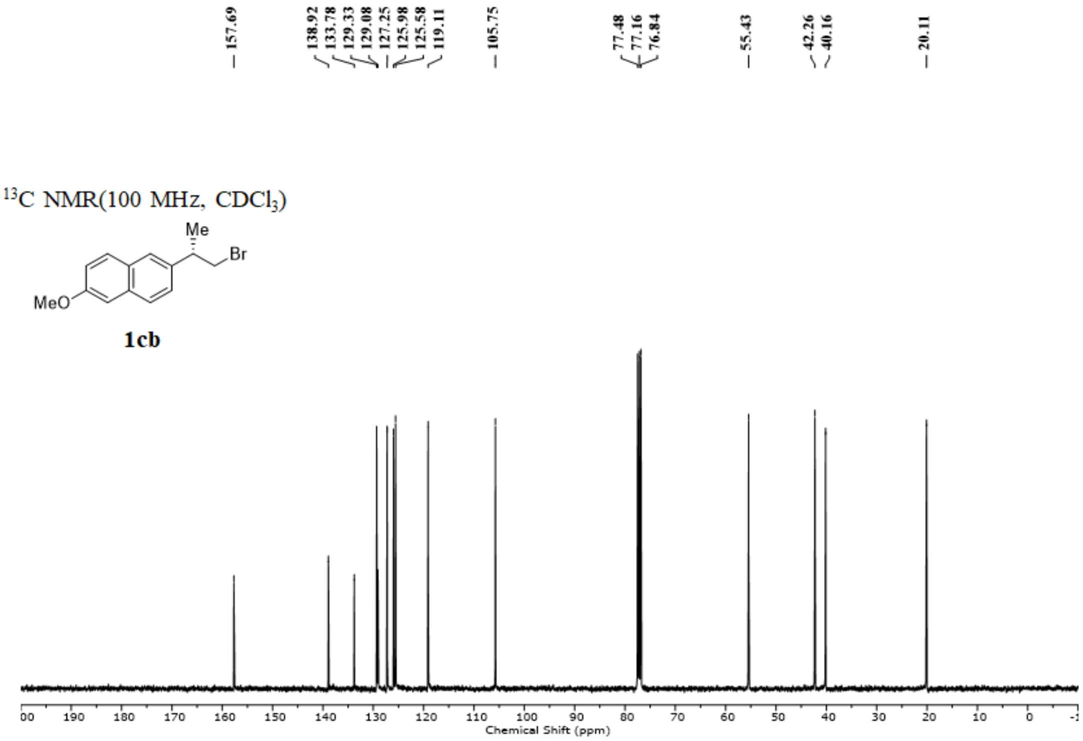

Supplementary Fig. 25. ^{13}C NMR of compound 1cb. The sample has been recorded in 100 MHz, $CDCl_3$ at 25 °C

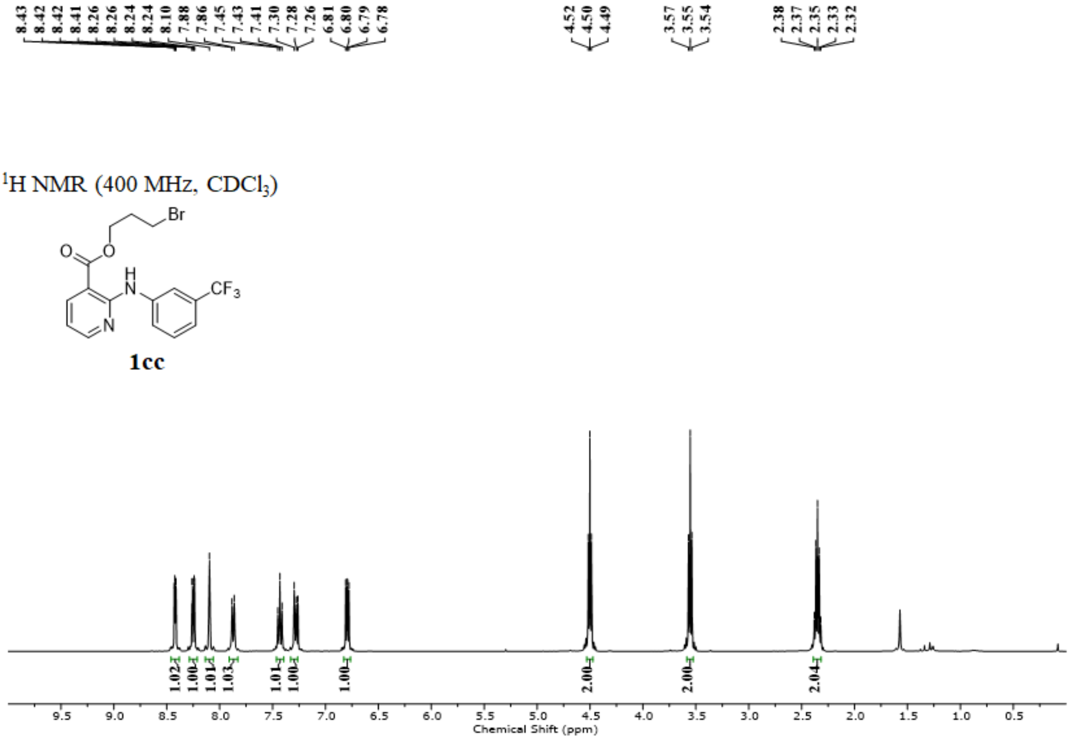

Supplementary Fig. 26. ^{1}H NMR of compound 1cc. The sample has been recorded in 400 MHz, $CDCl_3$ at 25 °C

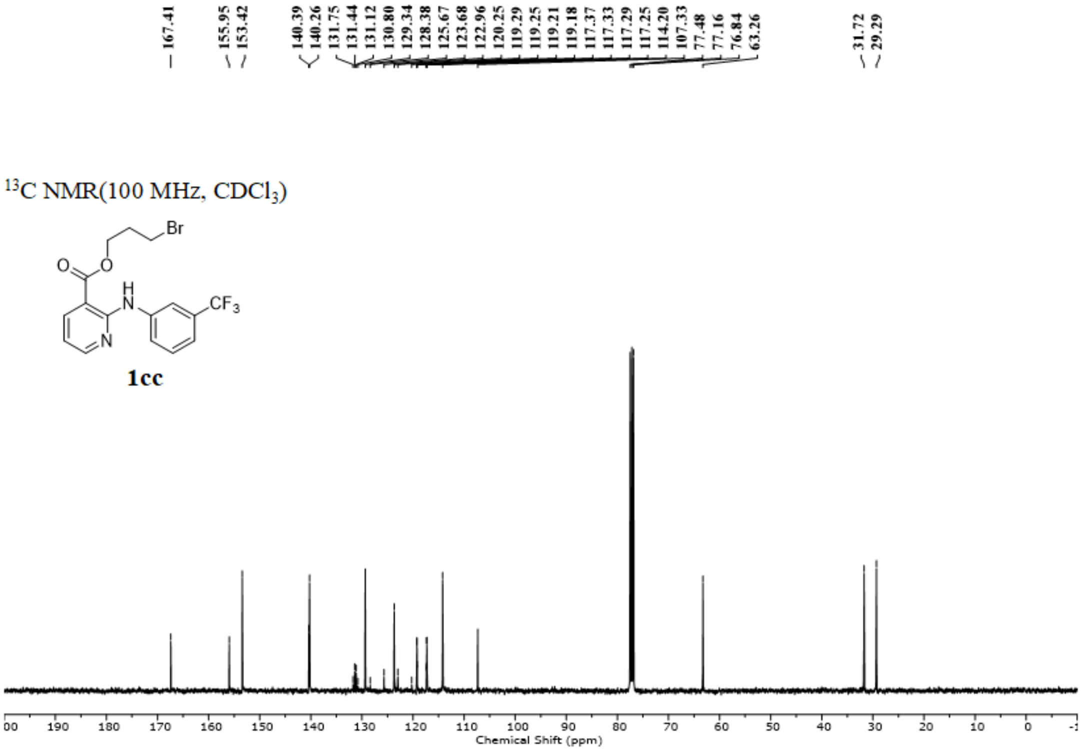

Supplementary Fig. 27. ^{13}C NMR of compound 1cc. The sample has been recorded in 100 MHz, $CDCl_3$ at 25 °C

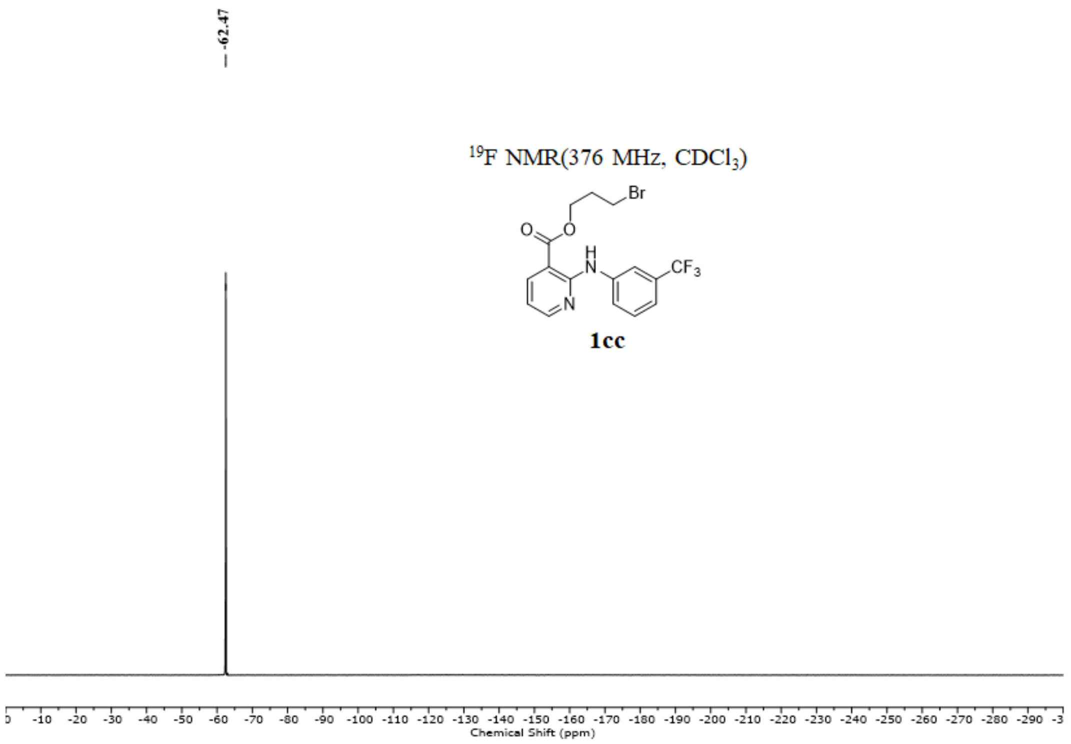

Supplementary Fig. 28. ^{19}F NMR of compound 1cc. The sample has been recorded in 376 MHz, $CDCl_3$ at 25 °C

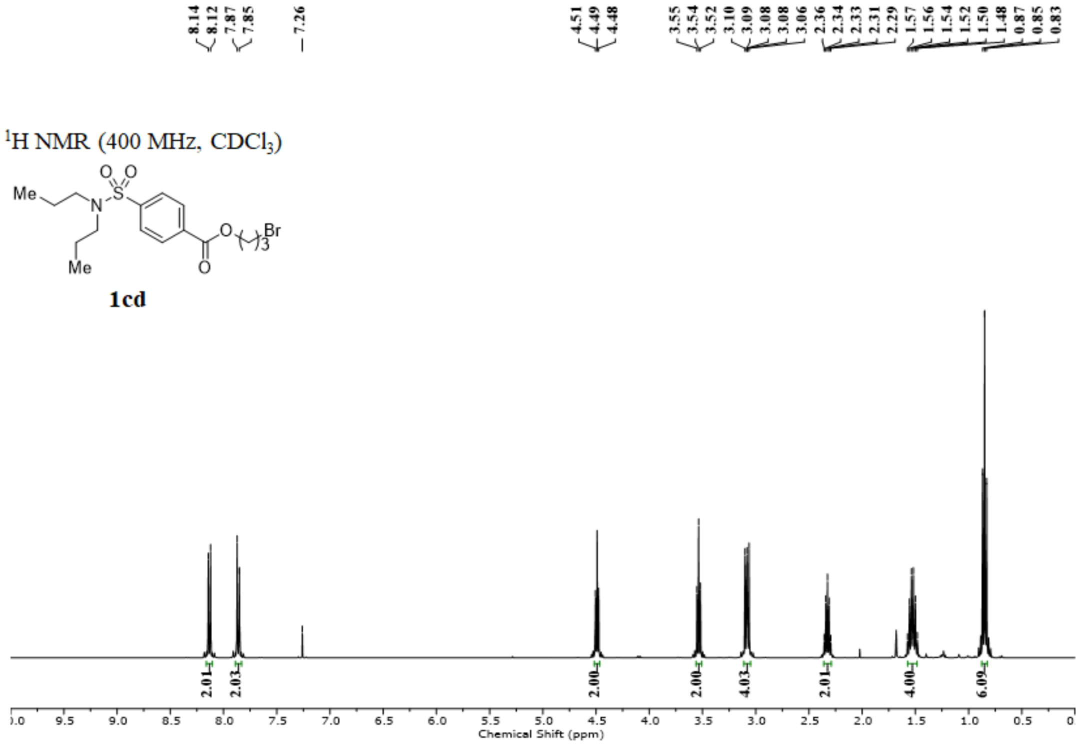

Supplementary Fig. 29. ^{1}H NMR of compound 1cd. The sample has been recorded in 400 MHz, $CDCl_3$ at 25 °C

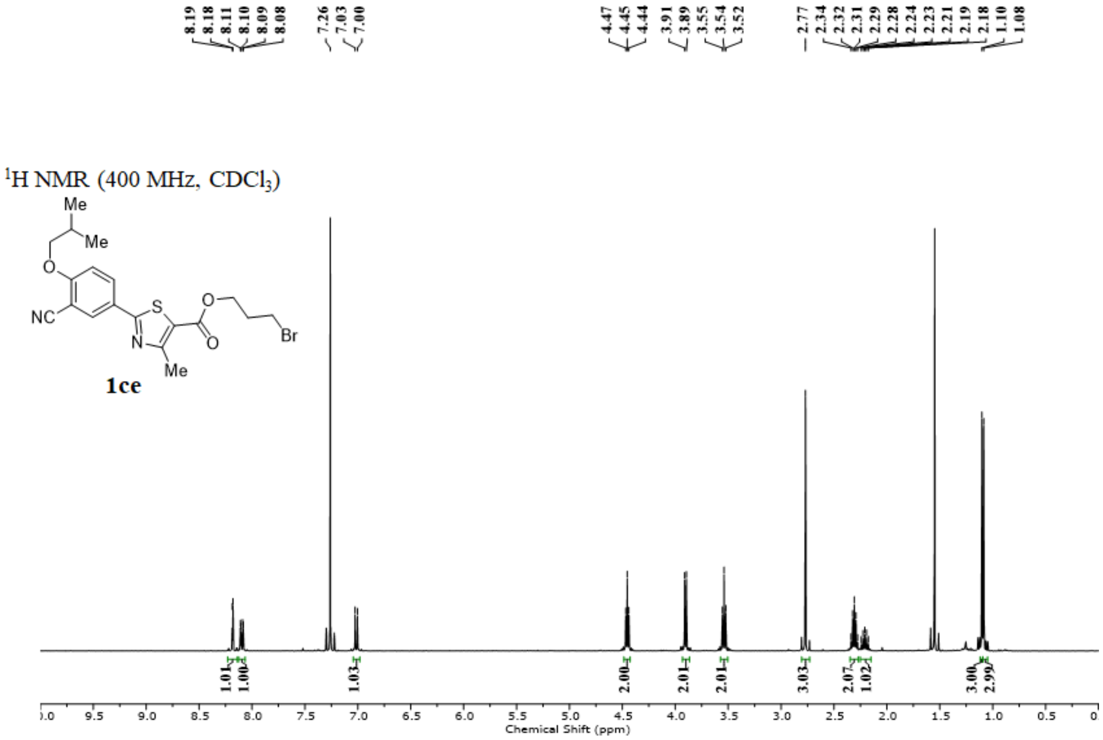

Supplementary Fig. 30. ^{1}H NMR of compound 1ce. The sample has been recorded in 400 MHz, $CDCl_3$ at 25 °C

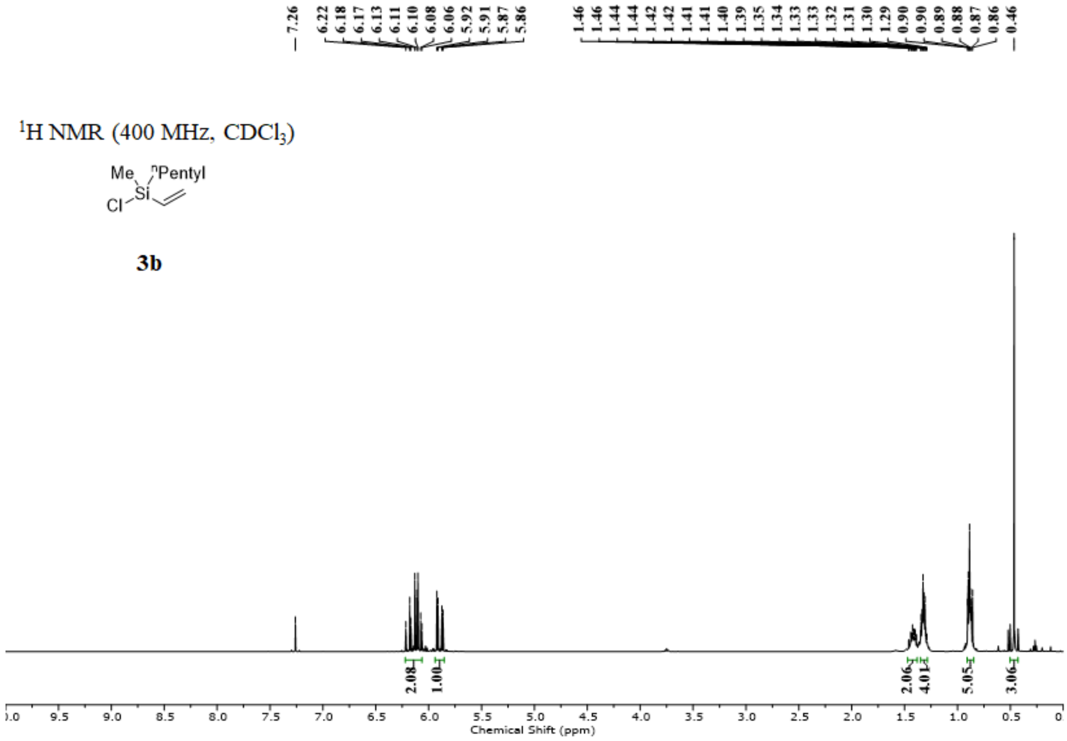

Supplementary Fig. 31. ^{1}H NMR of compound 3b. The sample has been recorded in 400 MHz, $CDCl_3$ at 25 °C

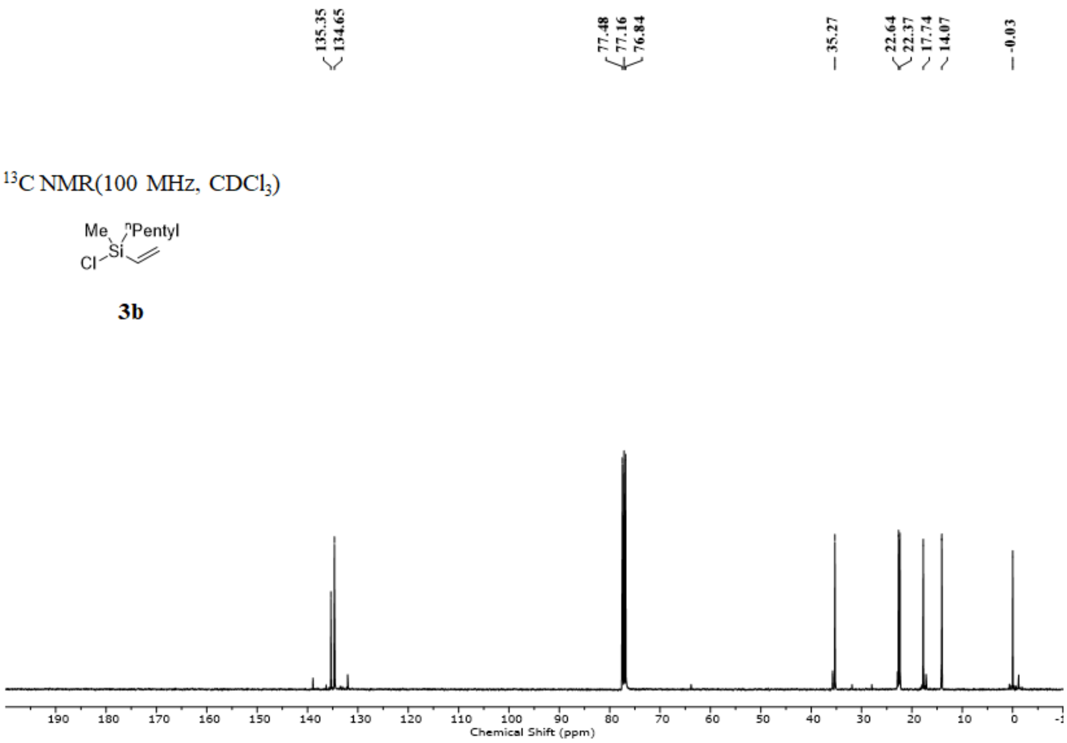

Supplementary Fig. 32. ^{13}C NMR of compound 3b. The sample has been recorded in 100 MHz, $CDCl_3$ at 25 °C

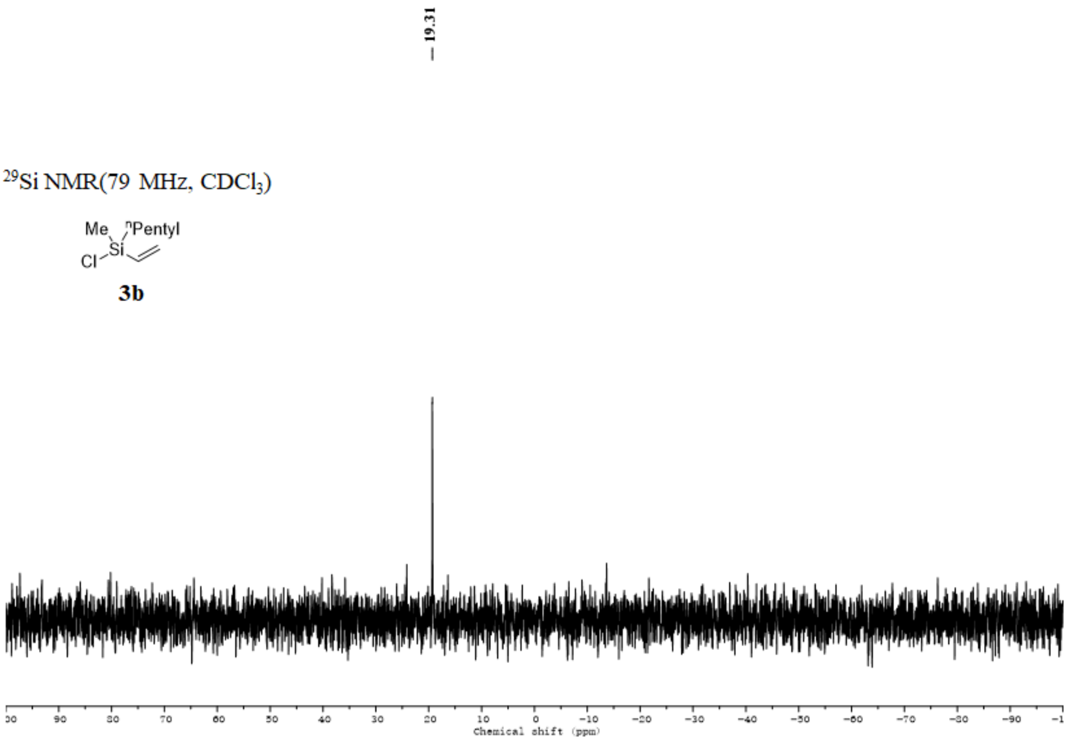

Supplementary Fig. 33. ^{29}Si NMR of compound 3b. The sample has been recorded in 79 MHz, $CDCl_3$ at 25 °C

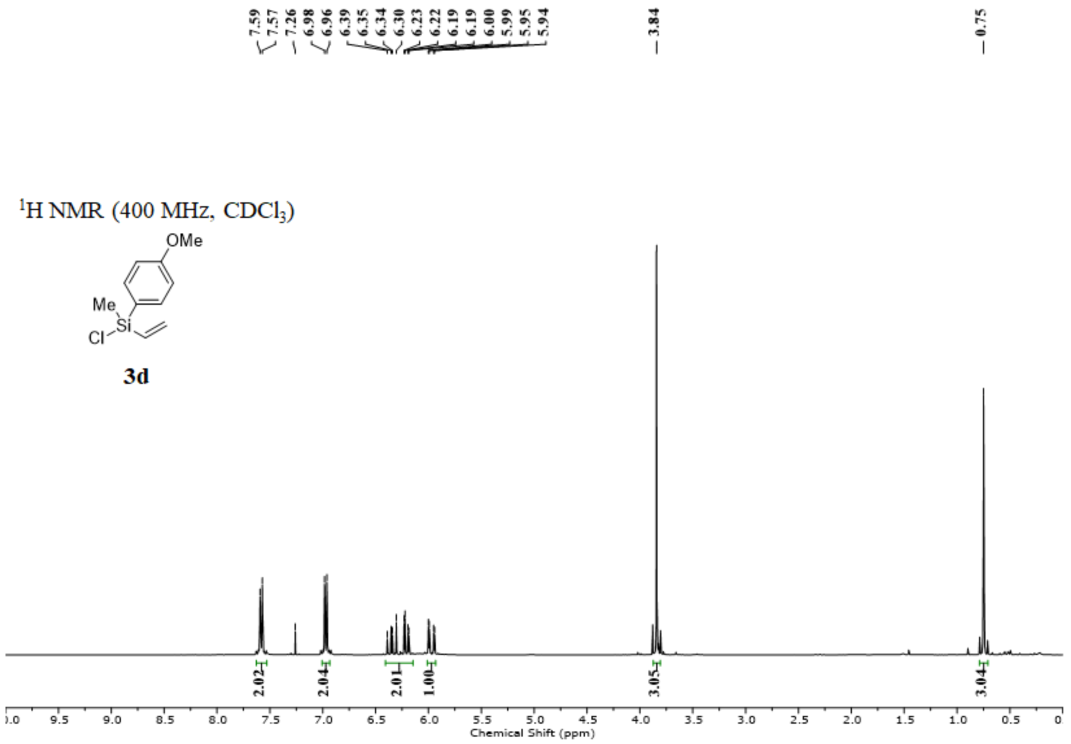

Supplementary Fig. 34. ¹H NMR of compound 3d. The sample has been recorded in 400 MHz, $CDCl_3$ at 25 °C

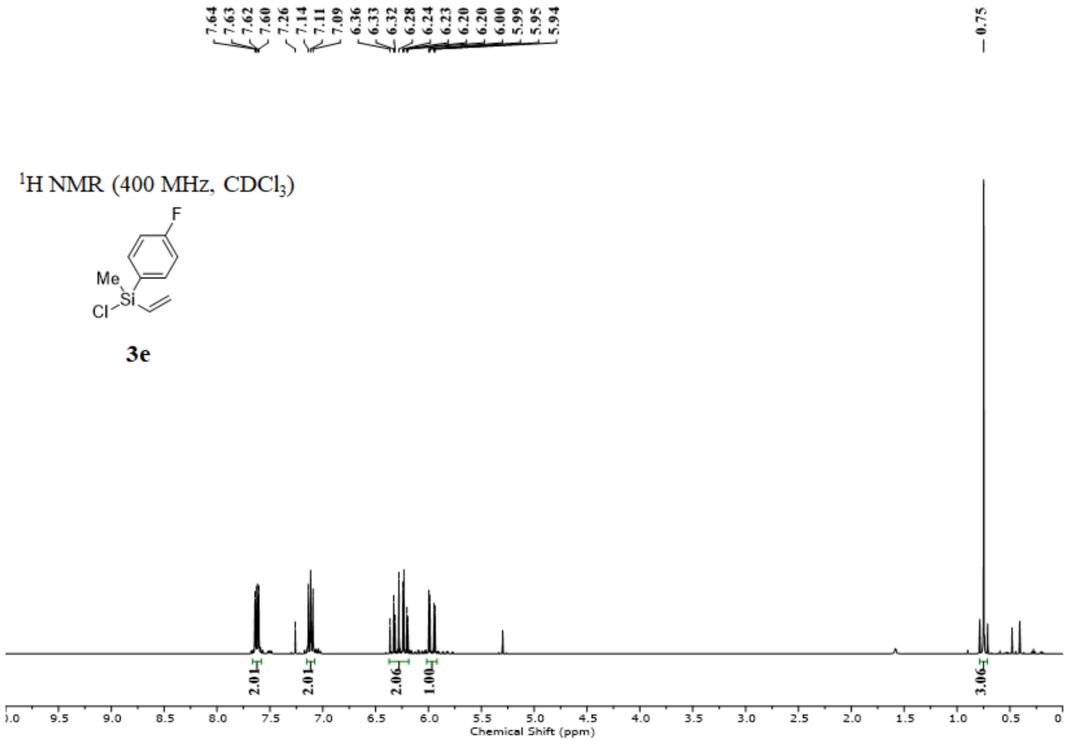

Supplementary Fig. 35. ¹H NMR of compound 3e. The sample has been recorded in 400 MHz, $CDCl_3$ at 25 °C

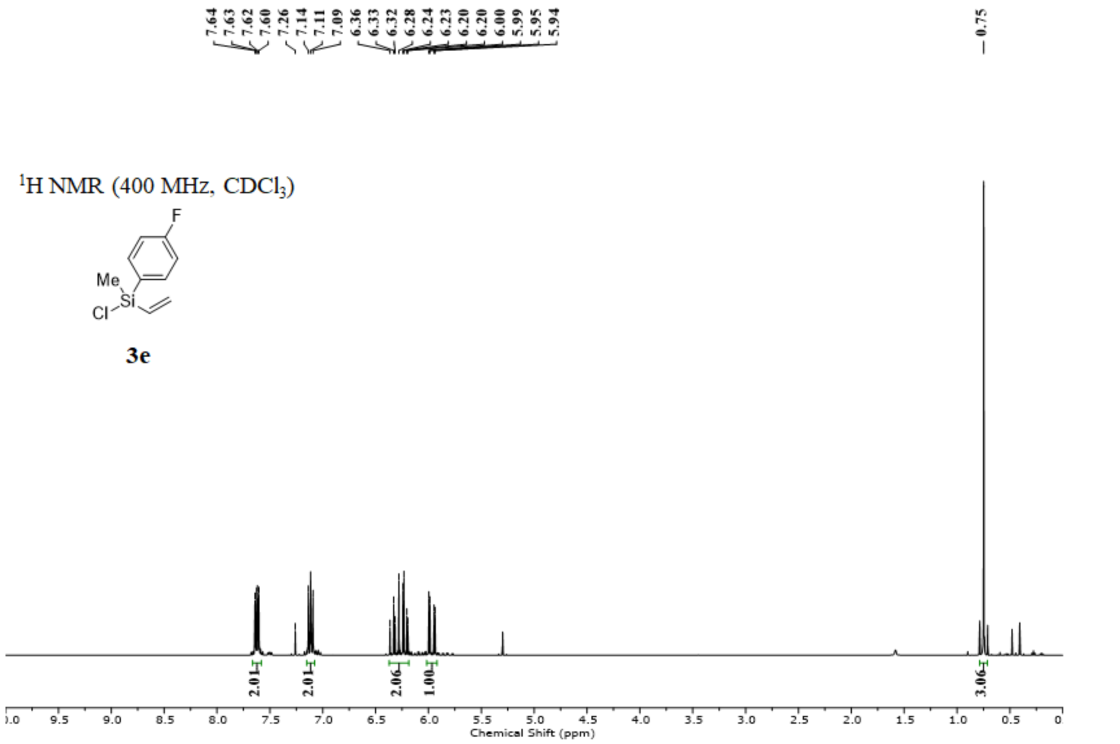

Supplementary Fig. 36. ^{1}H NMR of compound 3e. The sample has been recorded in 400 MHz, $CDCl_3$ at 25 °C

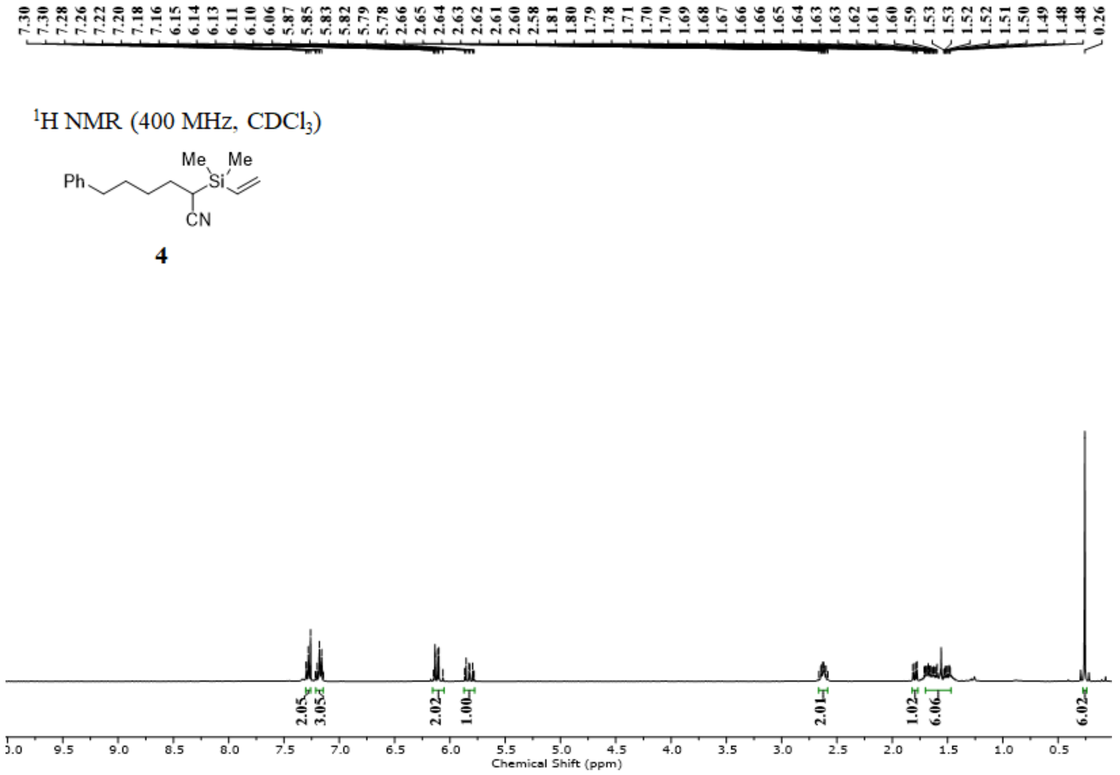

Supplementary Fig. 37. ^{1}H NMR of compound 4. The sample has been recorded in 400 MHz, $CDCl_3$ at 25 °C

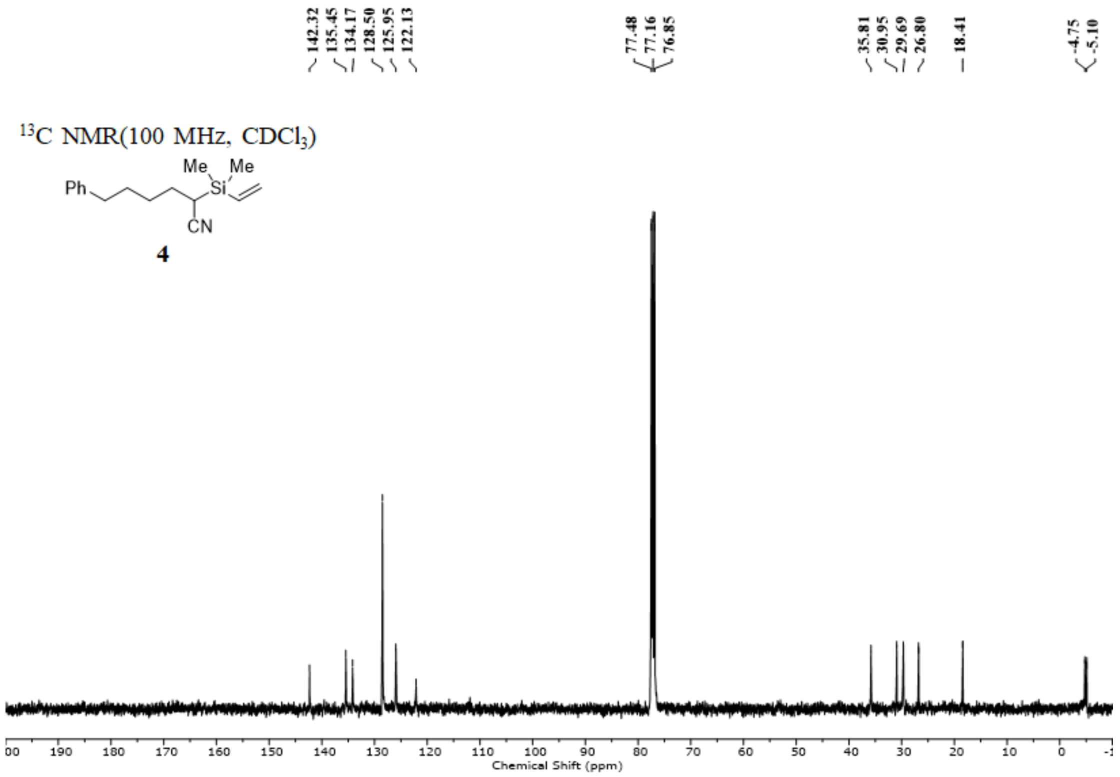

Supplementary Fig. 38. ^{13}C NMR of compound 4. The sample has been recorded in 100 MHz, $CDCl_3$ at 25 °C

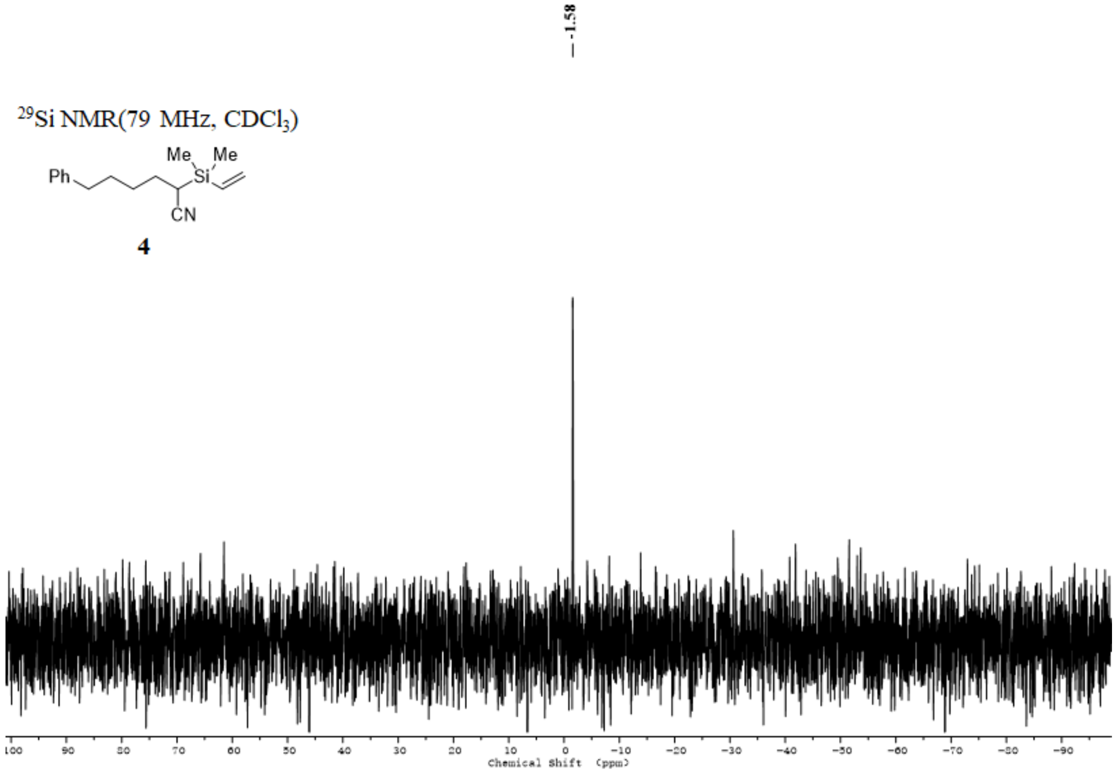

Supplementary Fig. 39. ^{29}Si NMR of compound 4. The sample has been recorded in 79 MHz, $CDCl_3$ at 25 °C

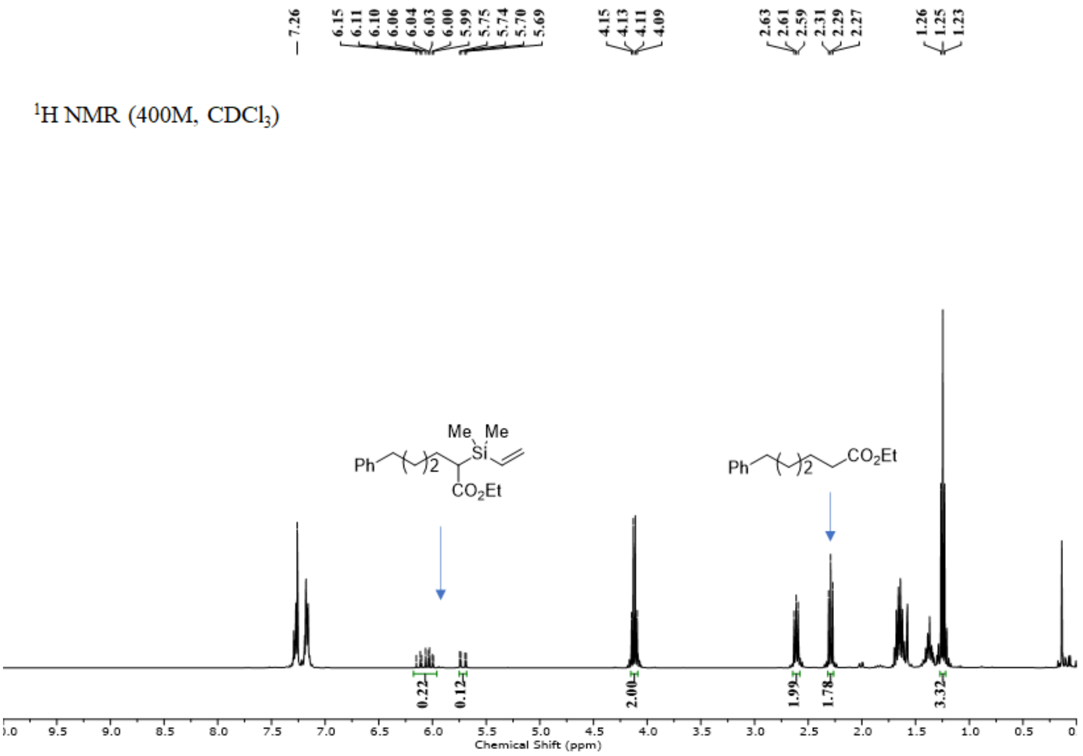

Supplementary Fig. 40. ^{1}H NMR of compound 4b. The sample has been recorded in 400 MHz, $CDCl_3$ at 25 °C

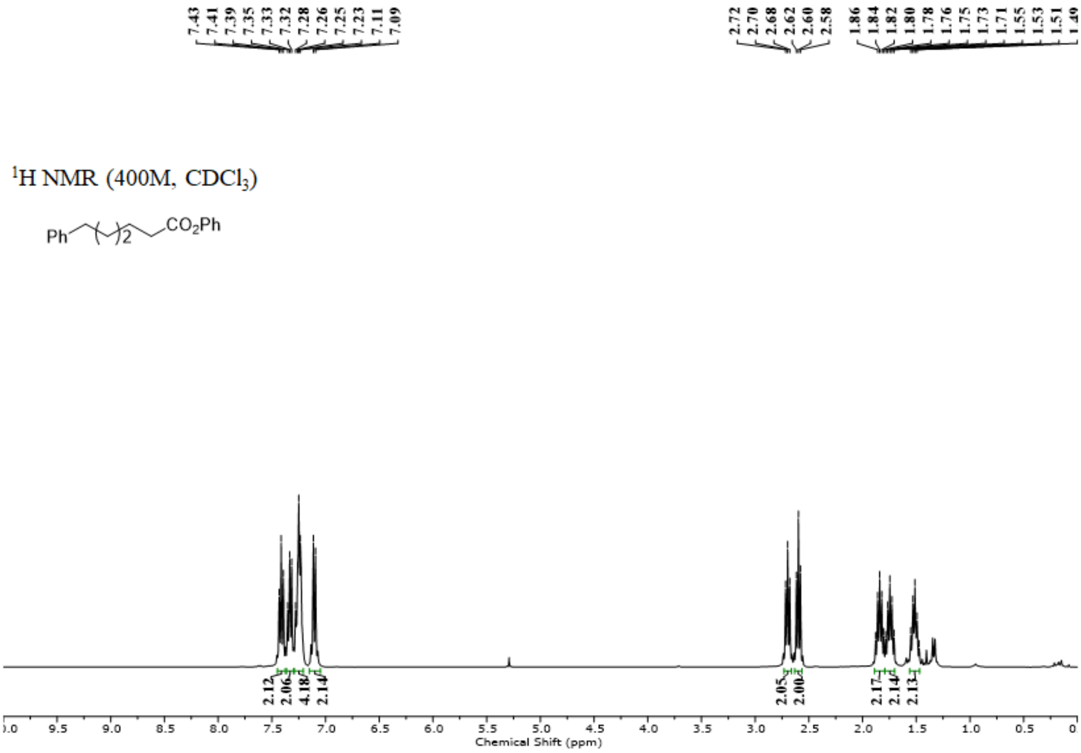

Supplementary Fig. 41. ^{1}H NMR of compound 4c. The sample has been recorded in 400 MHz, $CDCl_3$ at 25 °C

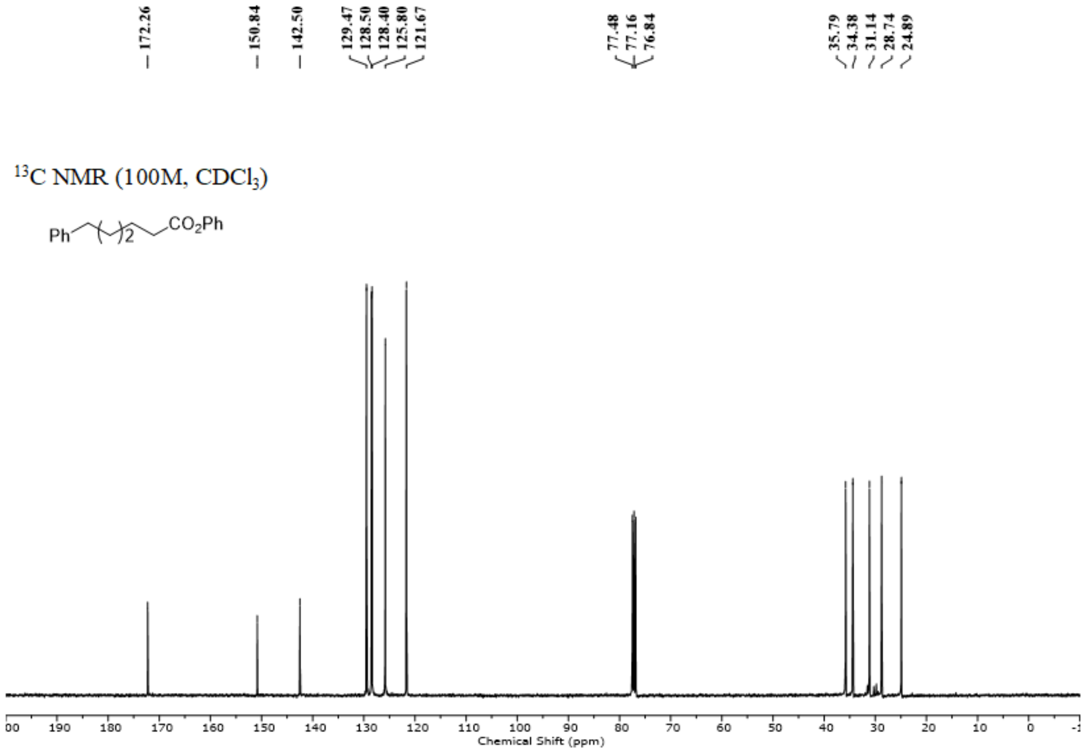

Supplementary Fig. 42. ^1H NMR of compound 4c. The sample has been recorded in 100 MHz, $CDCl_3$ at 25 °C

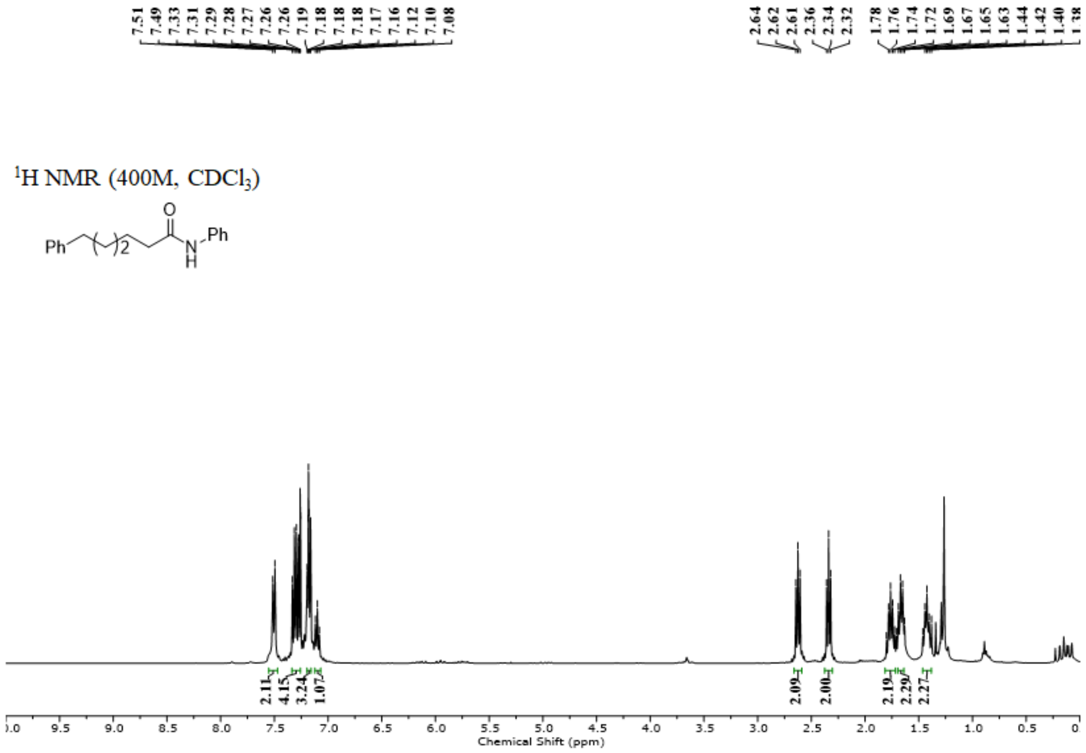

Supplementary Fig. 43. ^1H NMR of compound 4d. The sample has been recorded in 400 MHz, $CDCl_3$ at 25 °C

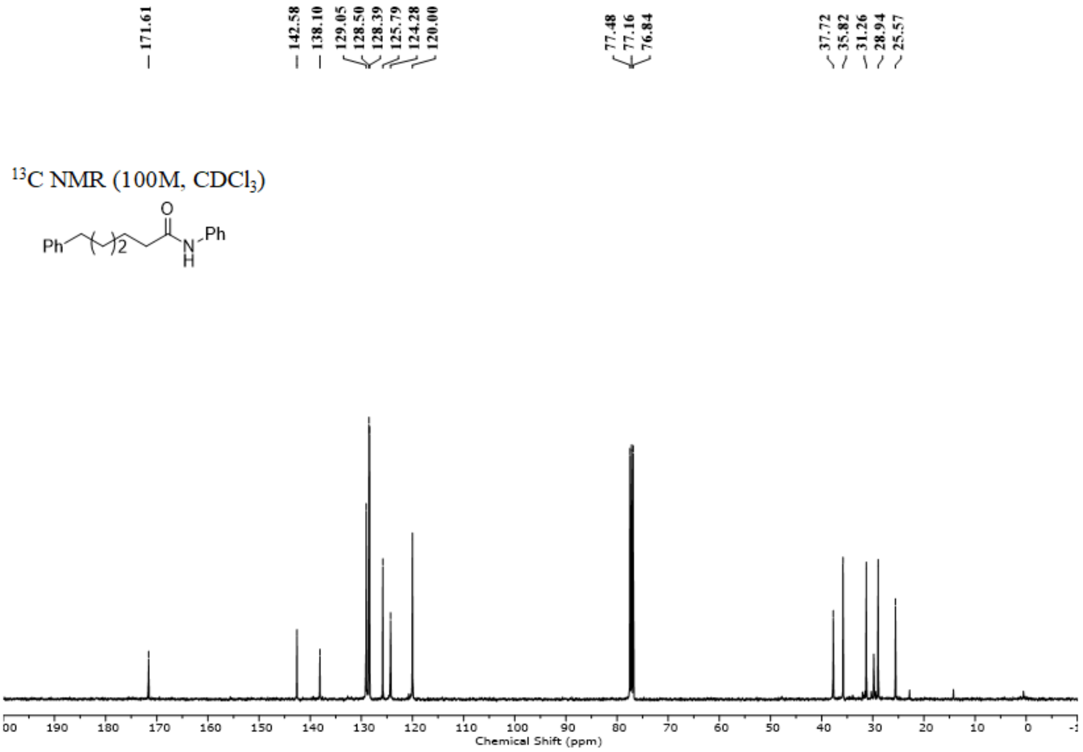

Supplementary Fig. 44. ^{1}H NMR of compound 4d. The sample has been recorded in 100 MHz, $CDCl_3$ at 25 °C

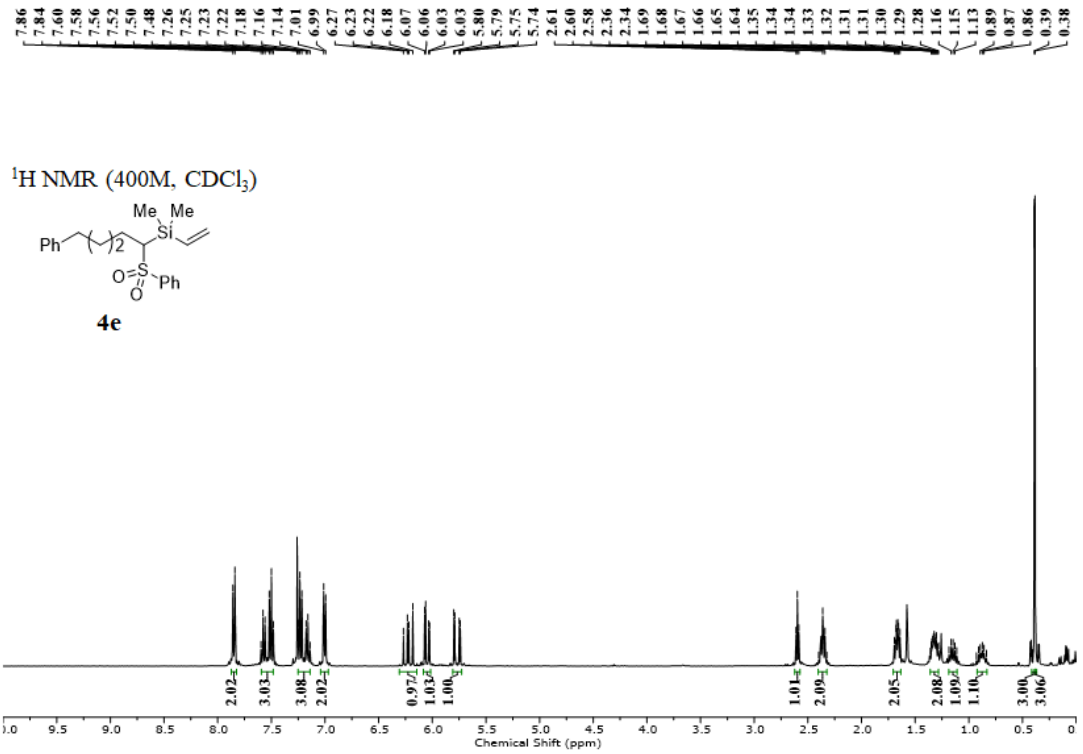

Supplementary Fig. 45. ^{1}H NMR of compound 4e. The sample has been recorded in 400 MHz, $CDCl_3$ at 25 °C

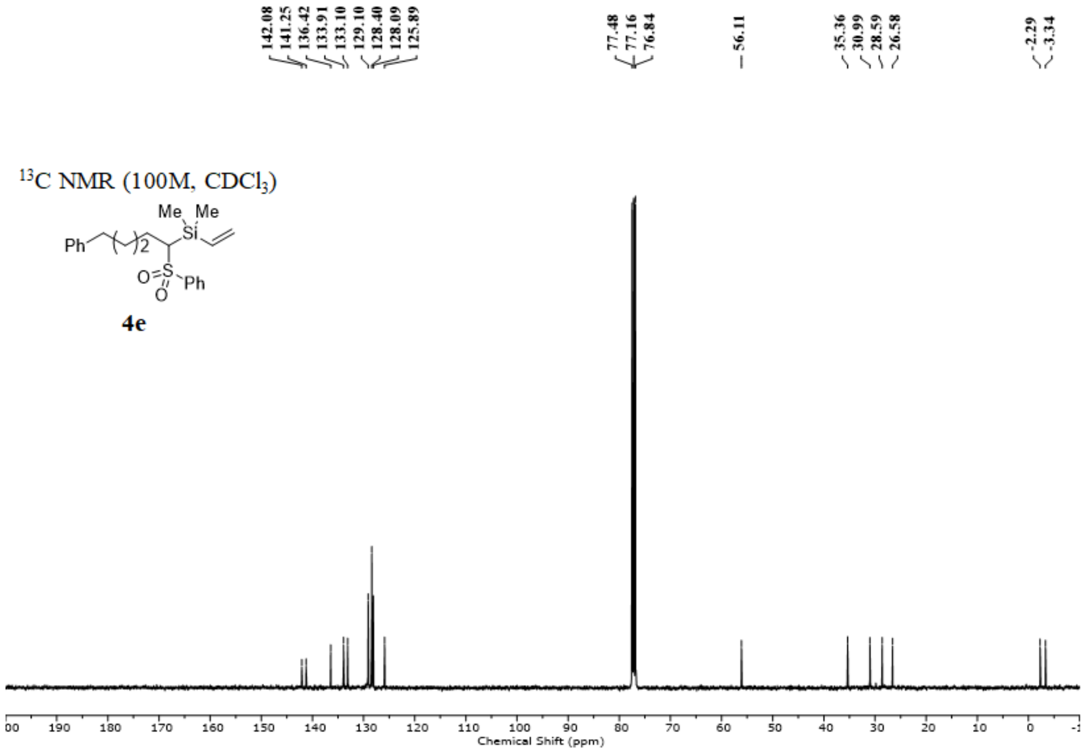

Supplementary Fig. 46. ^{13}C NMR of compound 4e. The sample has been recorded in 100 MHz, $CDCl_3$ at 25 °C

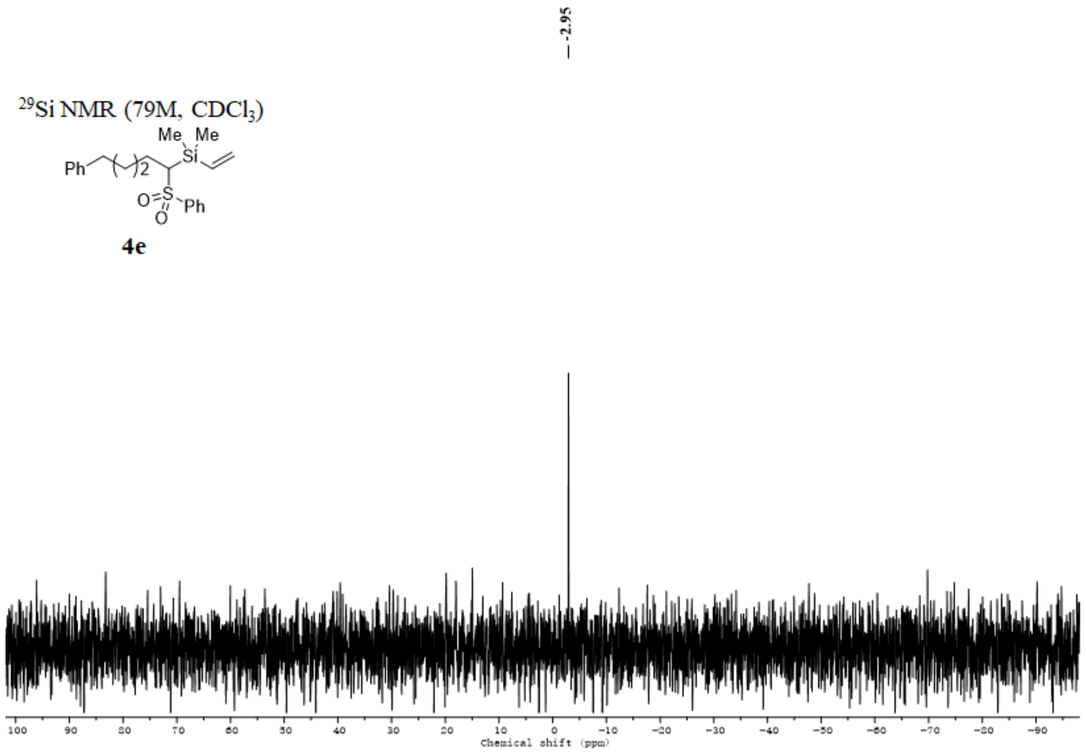

Supplementary Fig. 47. ^{29}Si NMR of compound 4e. The sample has been recorded in 79 MHz, $CDCl_3$ at 25 °C

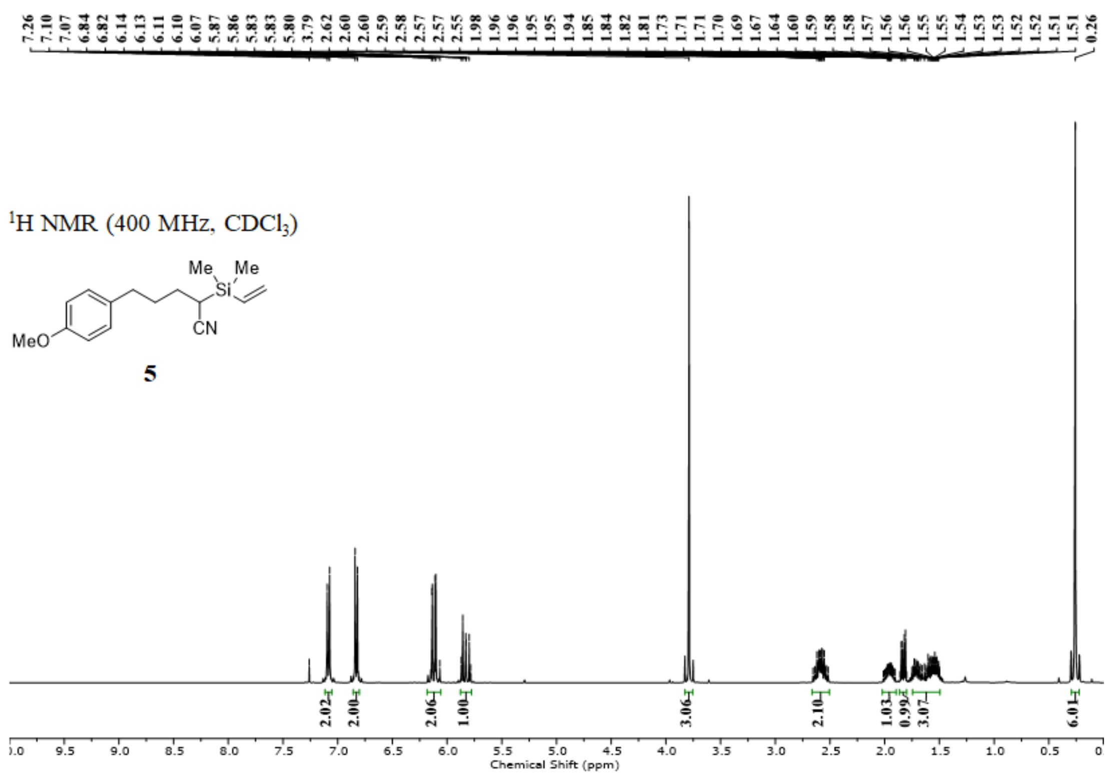

Supplementary Fig. 48. ^{1}H NMR of compound 5. The sample has been recorded in 400 MHz, $CDCl_3$ at 25 °C

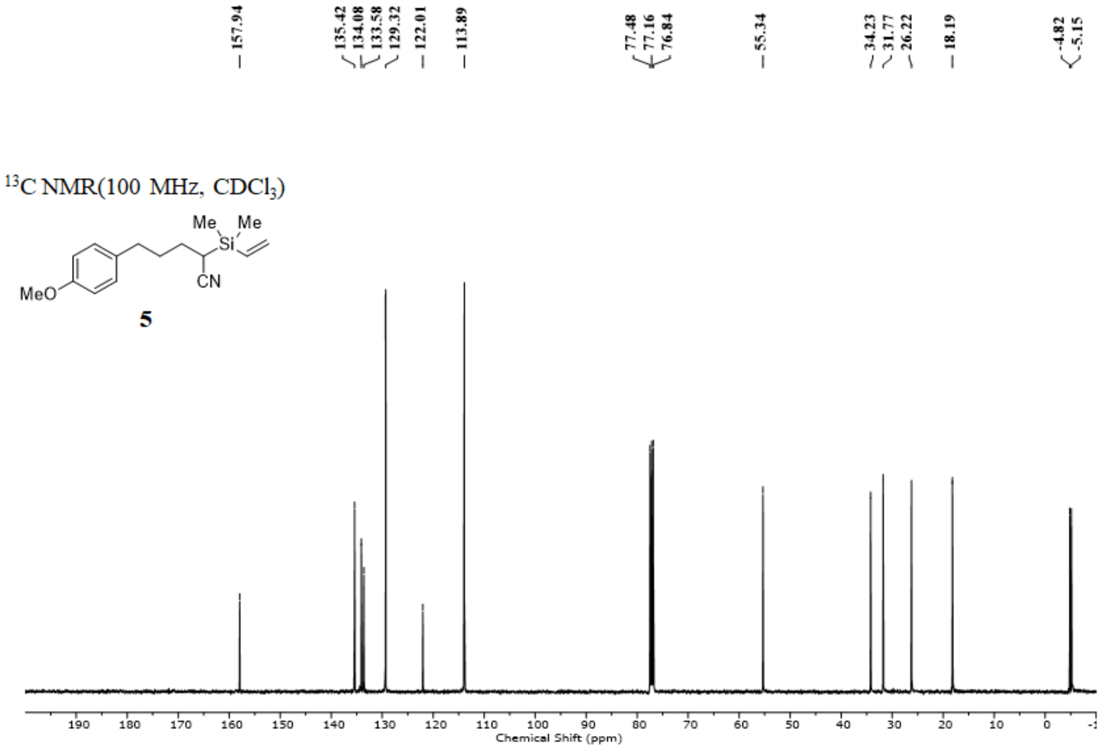

Supplementary Fig. 49. ^{13}C NMR of compound 5. The sample has been recorded in 100 MHz, $CDCl_3$ at 25 °C

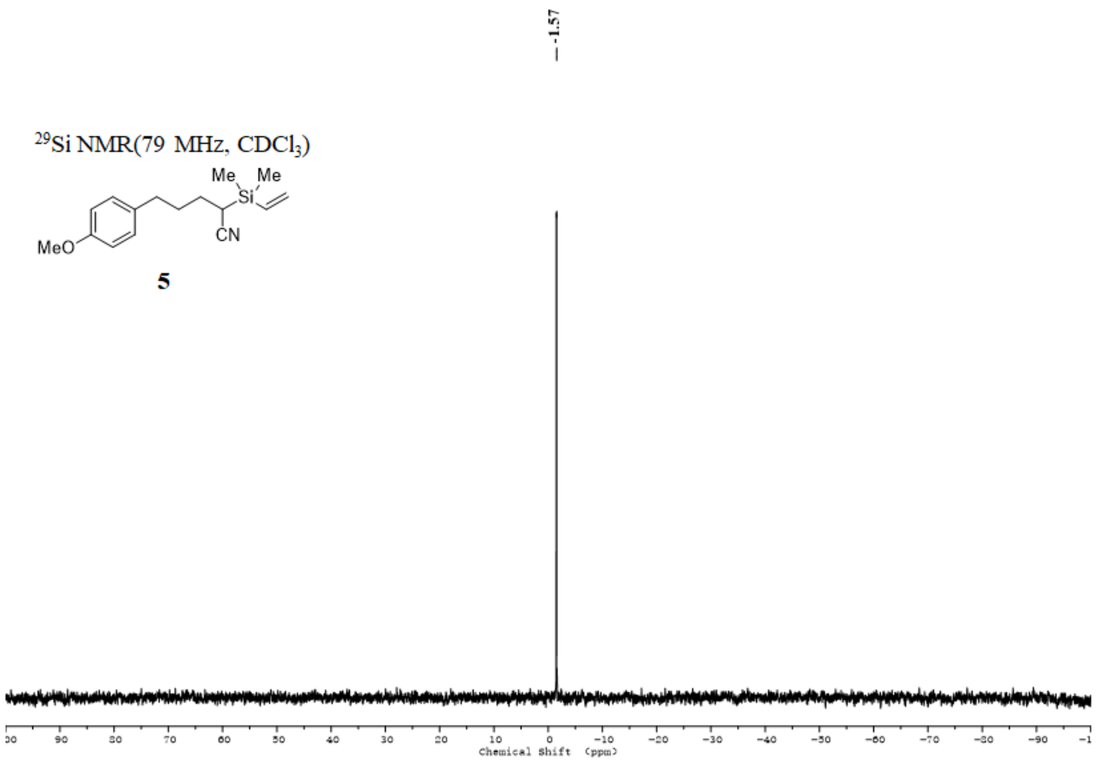

Supplementary Fig. 50. ^{29}Si NMR of compound 5. The sample has been recorded in 79 MHz, CDCl$_3$ at 25 °C

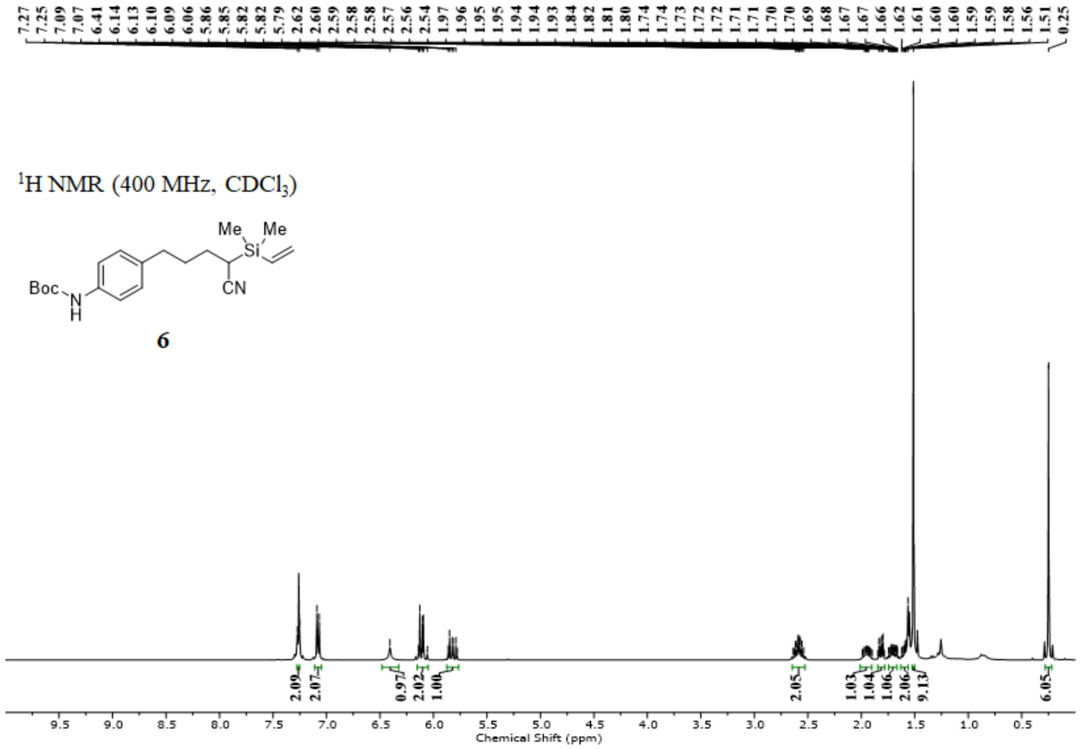

Supplementary Fig. 51. ^{1}H NMR of compound 6. The sample has been recorded in 400 MHz, CDCl$_3$ at 25 °C

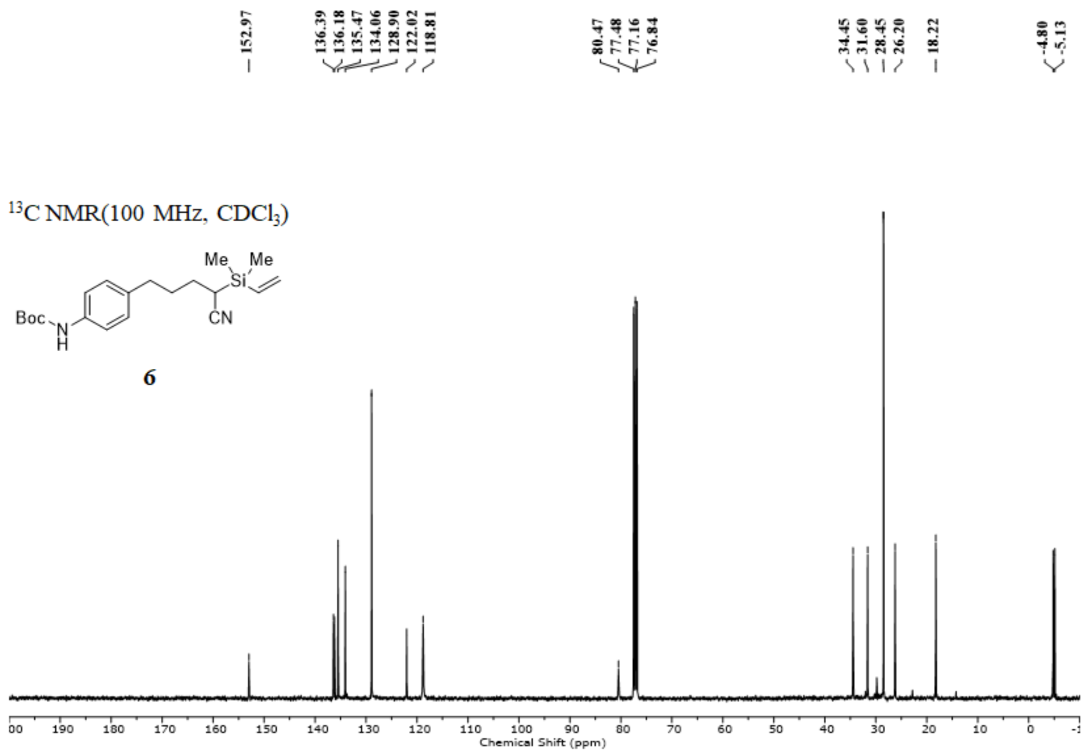

Supplementary Fig. 52. ^{13}C NMR of compound 6. The sample has been recorded in 100 MHz, $CDCl_3$ at 25 °C

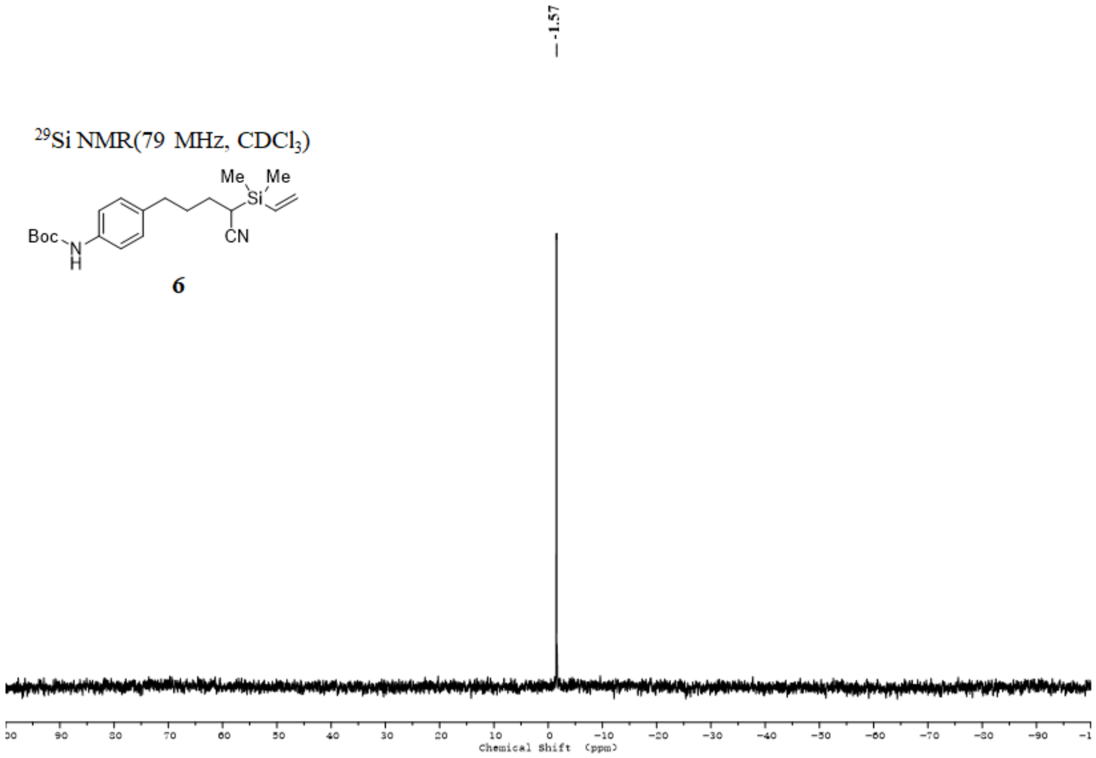

Supplementary Fig. 53. ^{29}Si NMR of compound 7. The sample has been recorded in 79 MHz, $CDCl_3$ at 25 °C

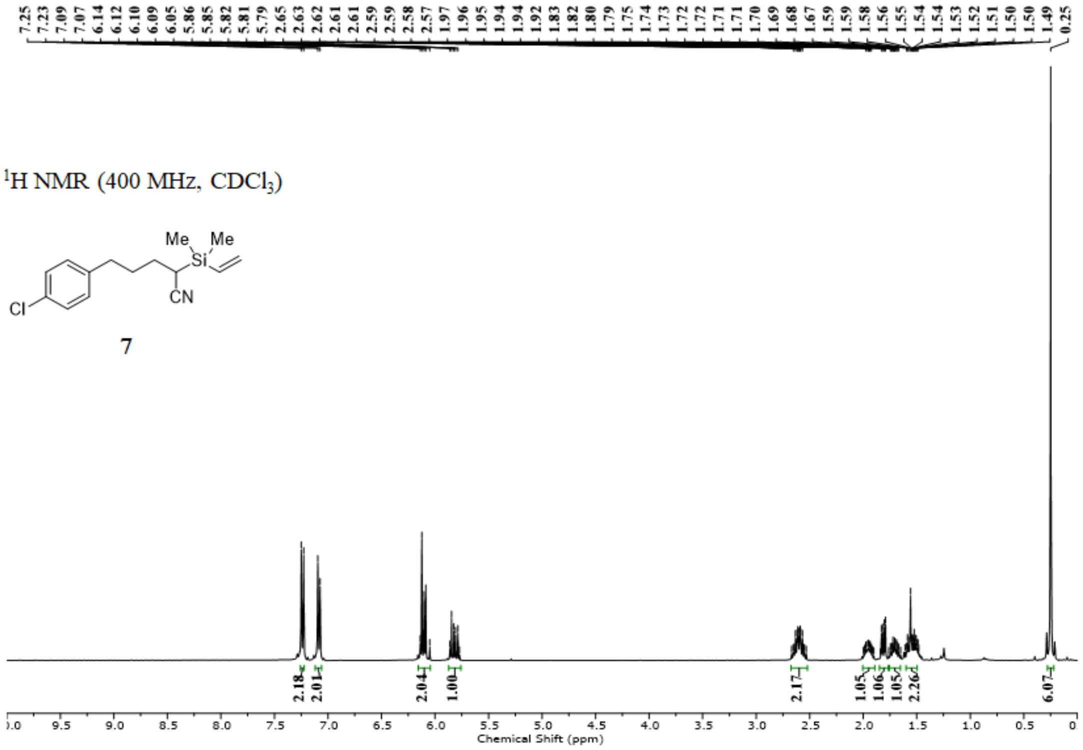

Supplementary Fig. 54. ^{1}H NMR of compound 7. The sample has been recorded in 400 MHz, $CDCl_3$ at 25 °C

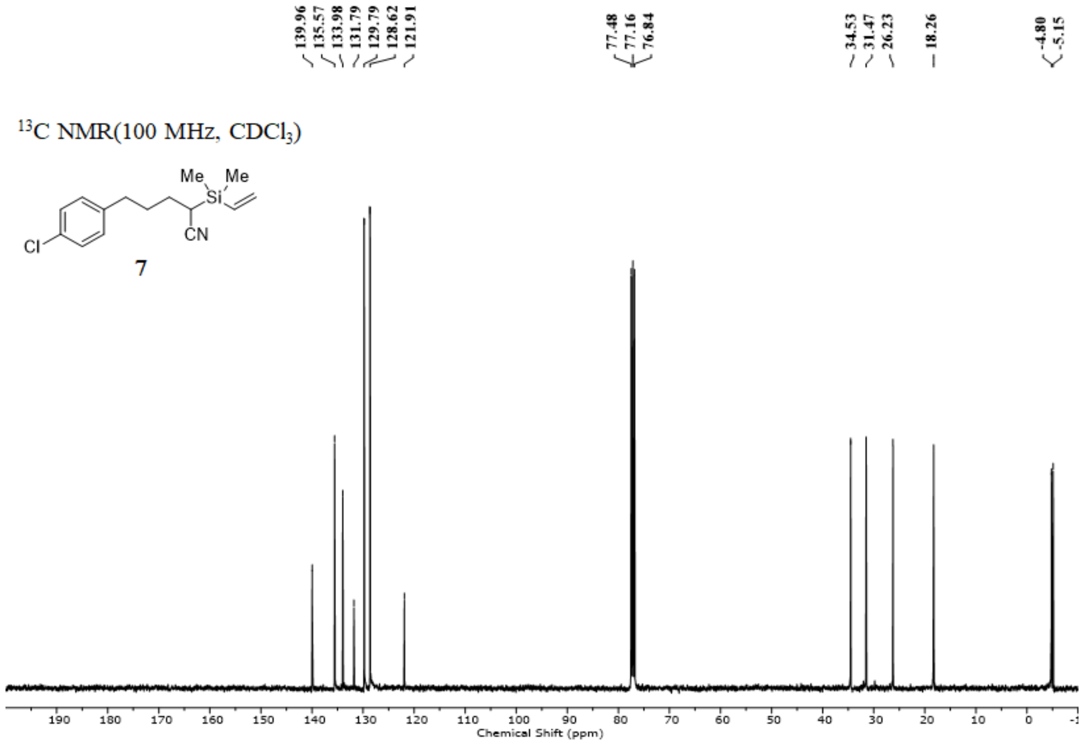

Supplementary Fig. 55. ^{13}C NMR of compound 7. The sample has been recorded in 100 MHz, $CDCl_3$ at 25 °C

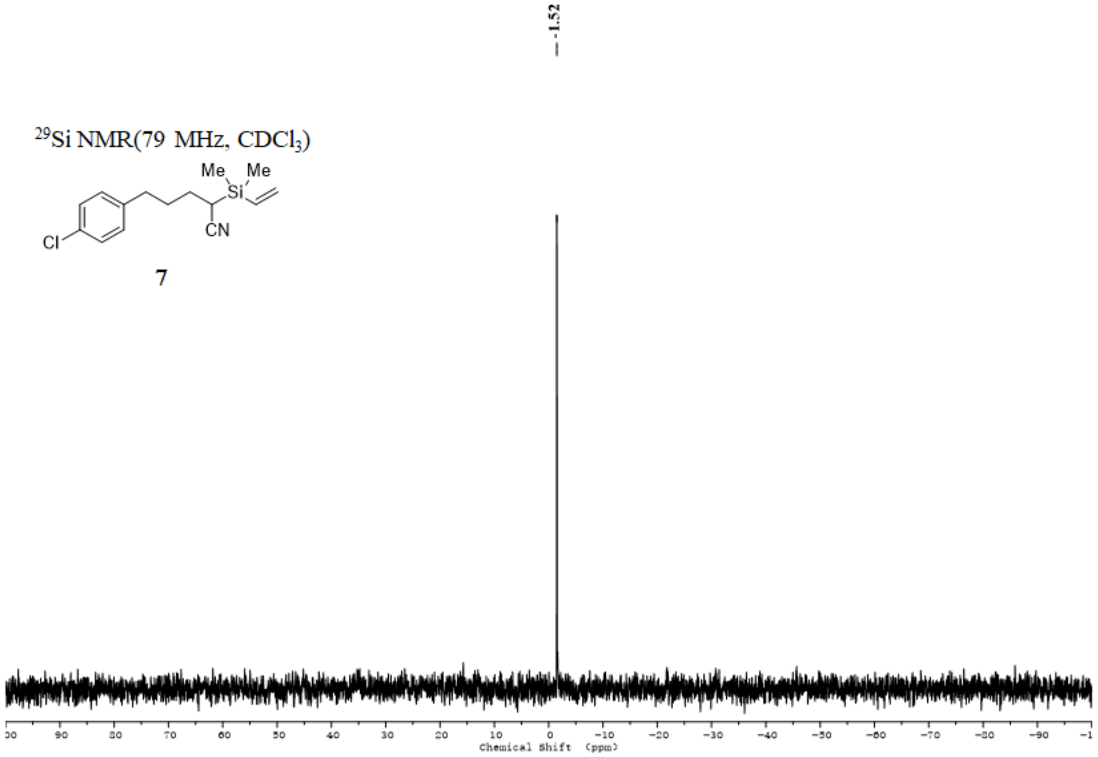

Supplementary Fig. 56. ^{29}Si NMR of compound 7. The sample has been recorded in 79 MHz, $CDCl_3$ at 25 °C

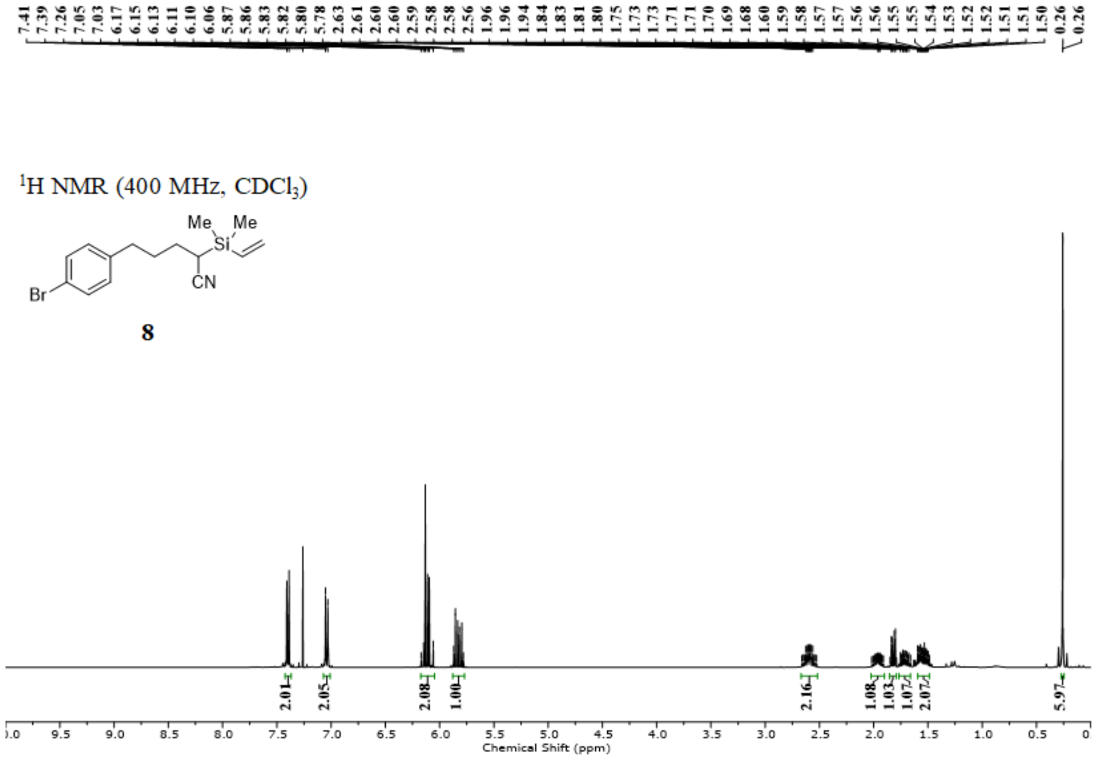

Supplementary Fig. 57. ^{1}H NMR of compound 8. The sample has been recorded in 400 MHz, $CDCl_3$ at 25 °C

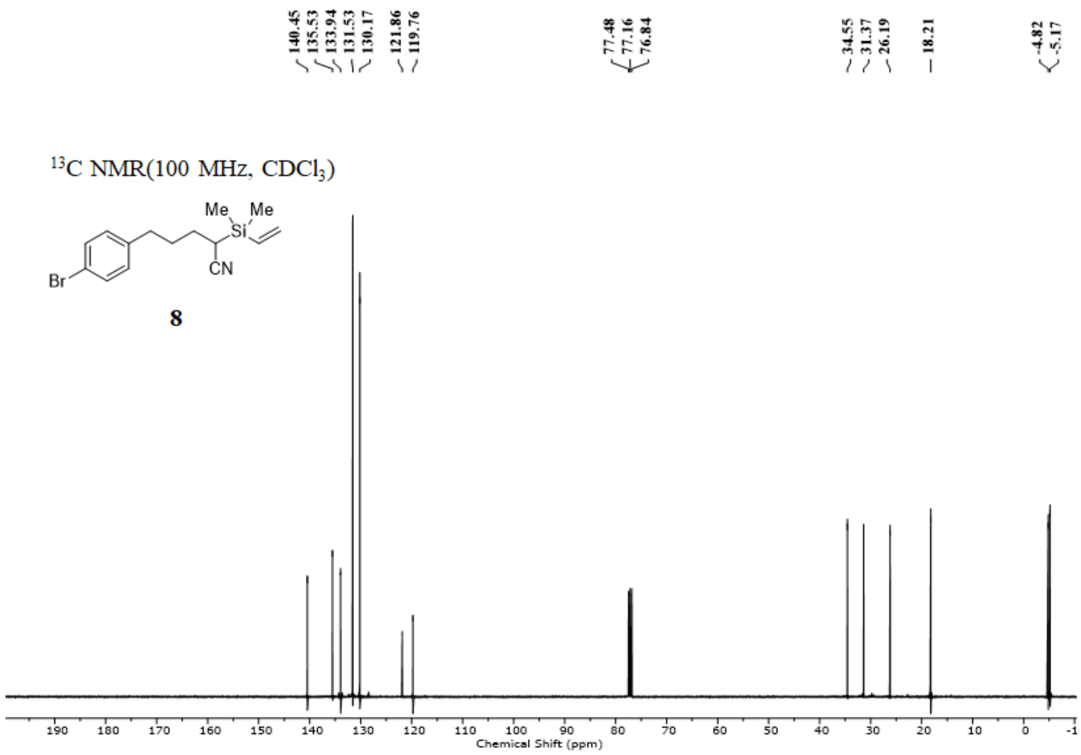

Supplementary Fig. 58. ^{13}C NMR of compound 8 The sample has been recorded in 100 MHz, $CDCl_3$ at 25 °C

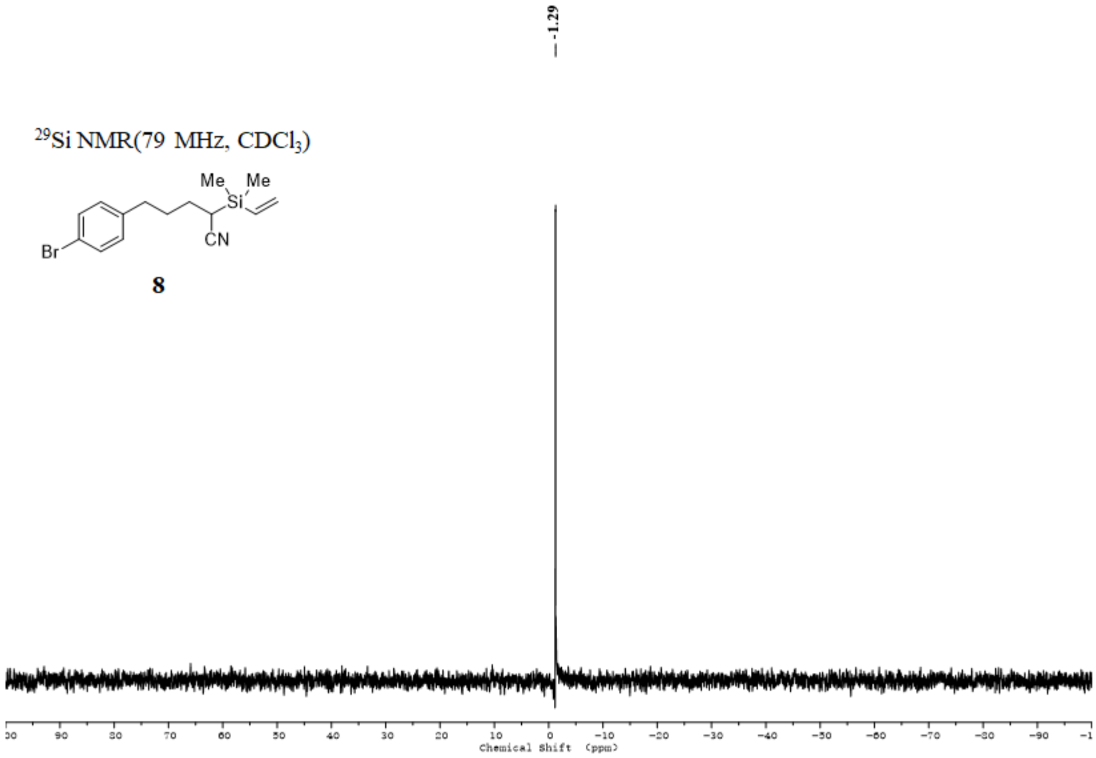

Supplementary Fig. 59. ^{29}Si NMR of compound 8. The sample has been recorded in 79 MHz, $CDCl_3$ at 25 °C

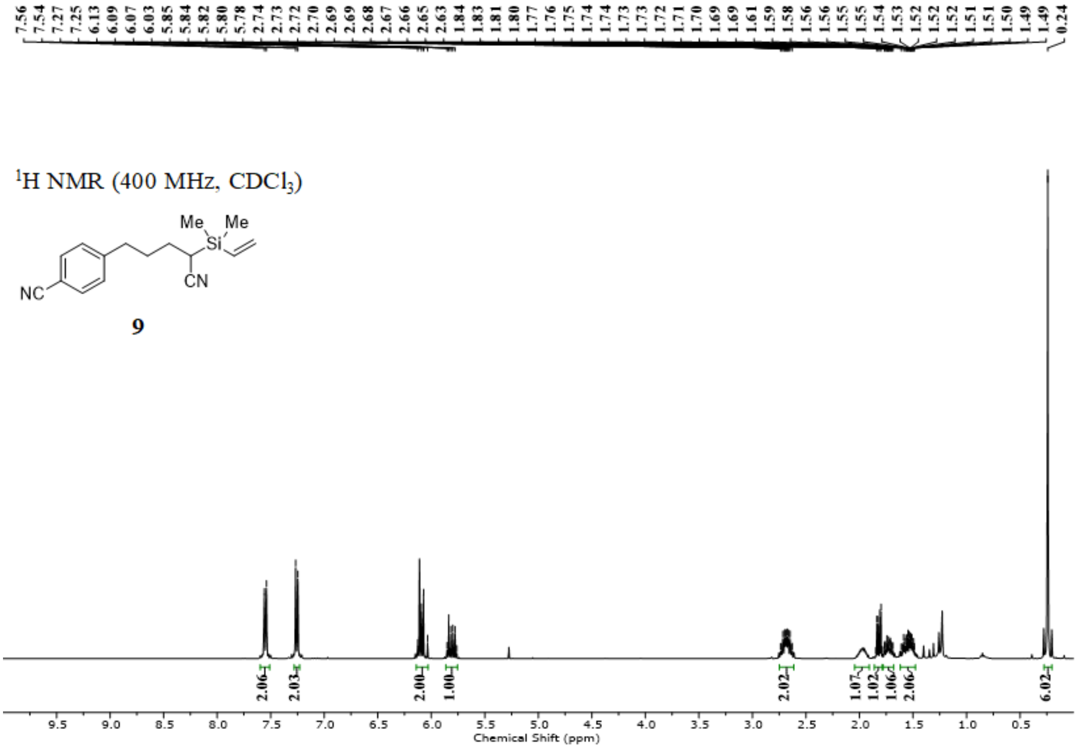

Supplementary Fig. 60. ^{1}H NMR of compound 9. The sample has been recorded in 400 MHz, $CDCl_3$ at 25 °C

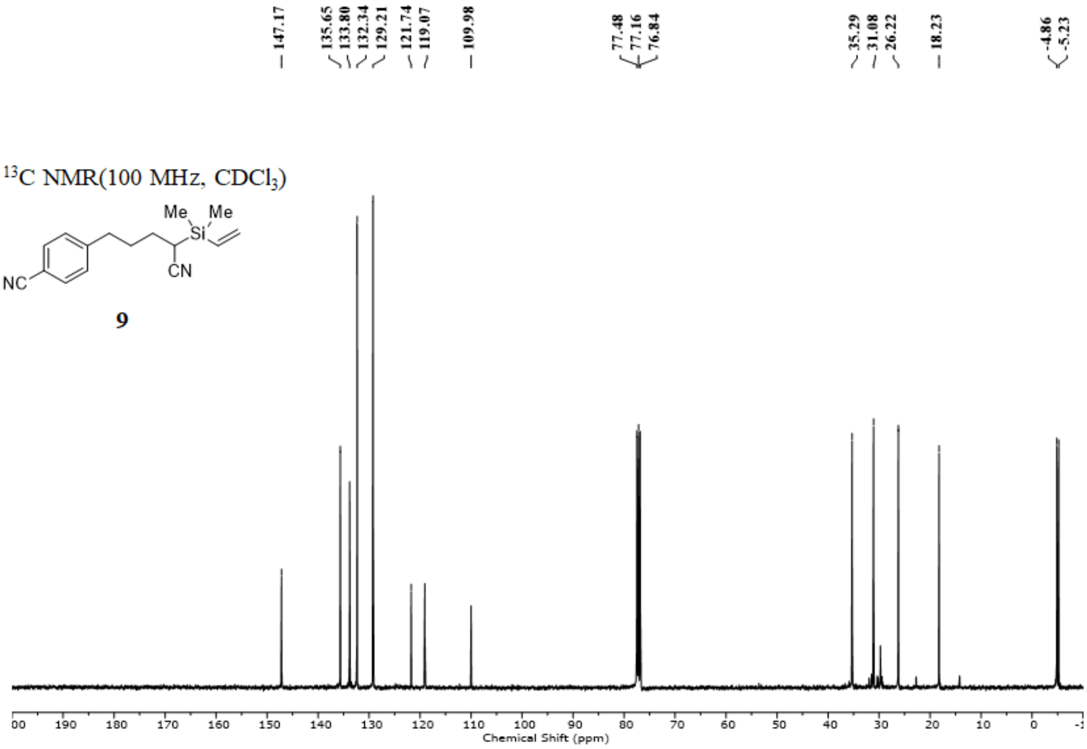

Supplementary Fig. 61. ^{13}C NMR of compound 9. The sample has been recorded in 100 MHz, $CDCl_3$ at 25 °C

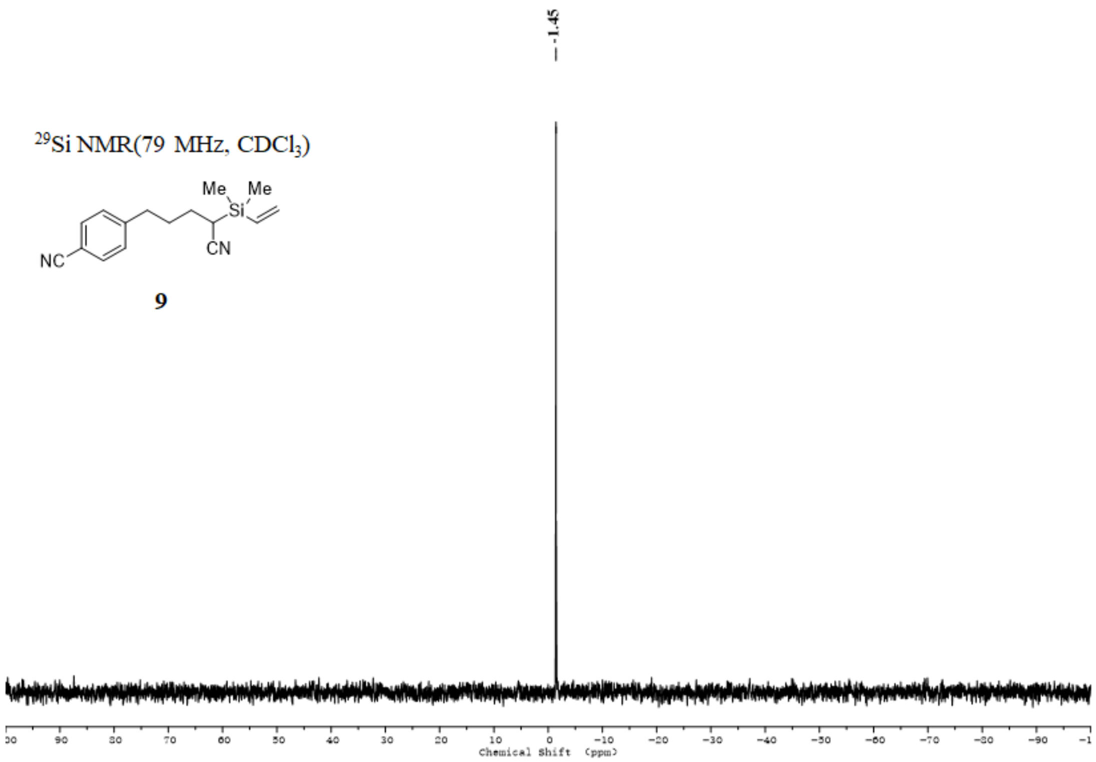

Supplementary Fig. 62. ^{29}Si NMR of compound 9. The sample has been recorded in 79 MHz, $CDCl_3$ at 25 °C

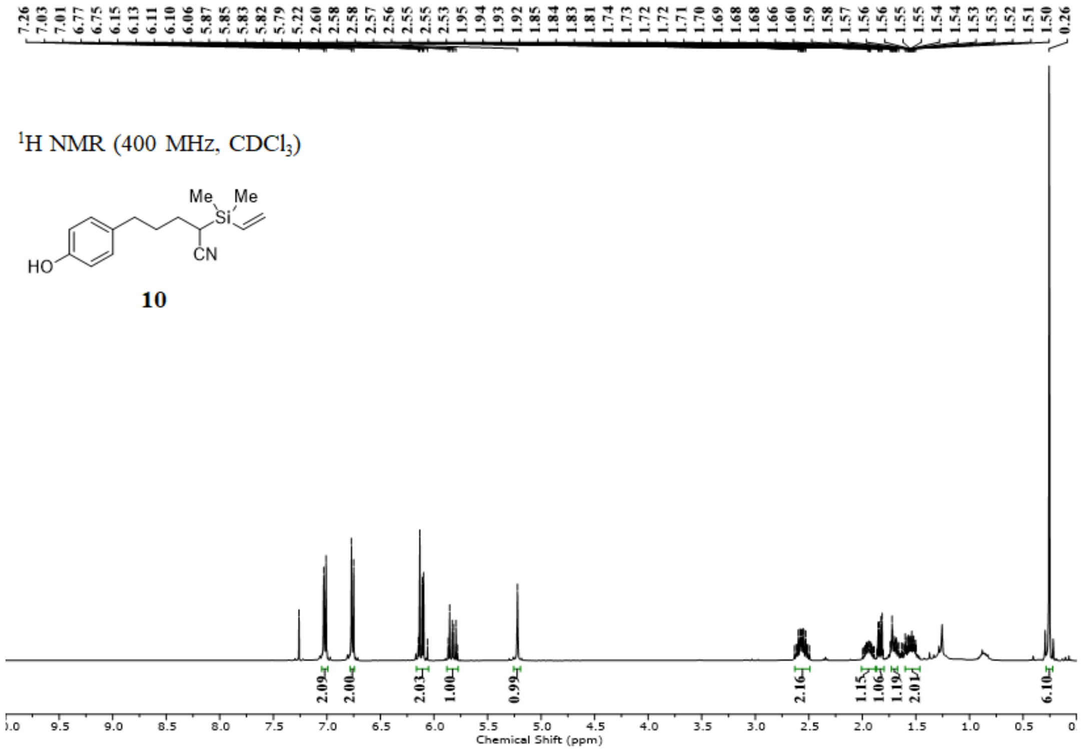

Supplementary Fig. 63. ^{1}H NMR of compound 10. The sample has been recorded in 400 MHz, $CDCl_3$ at 25 °C

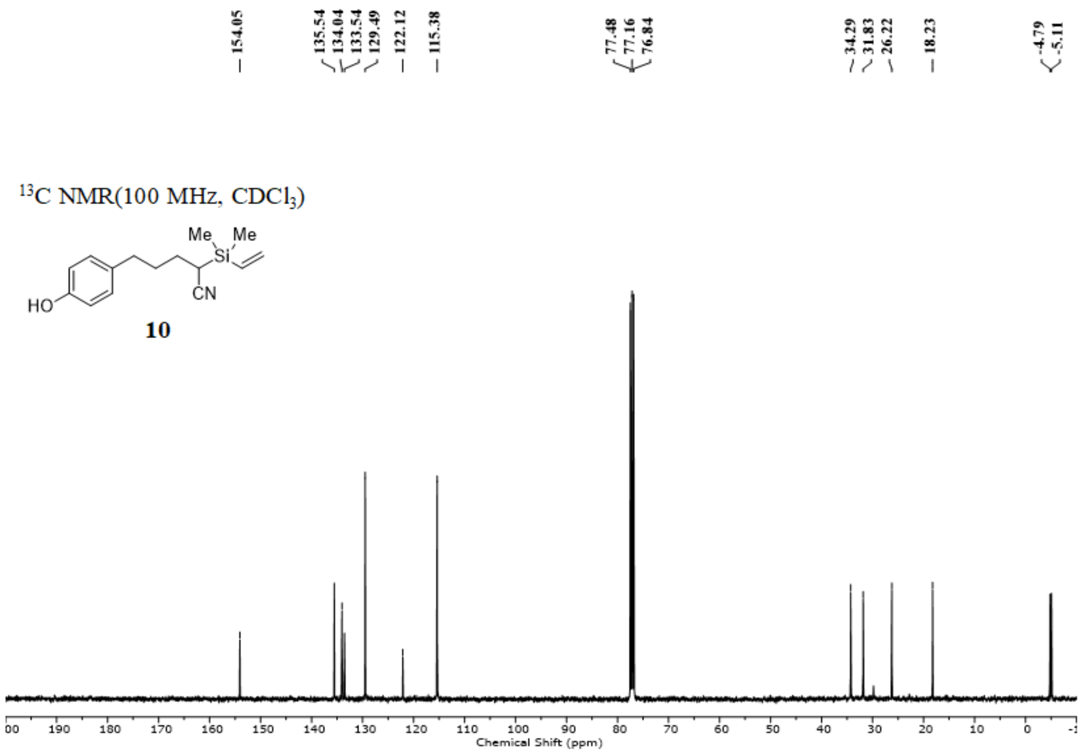

Supplementary Fig. 64. ^{13}C NMR of compound 10. The sample has been recorded in 100 MHz, $CDCl_3$ at 25 °C

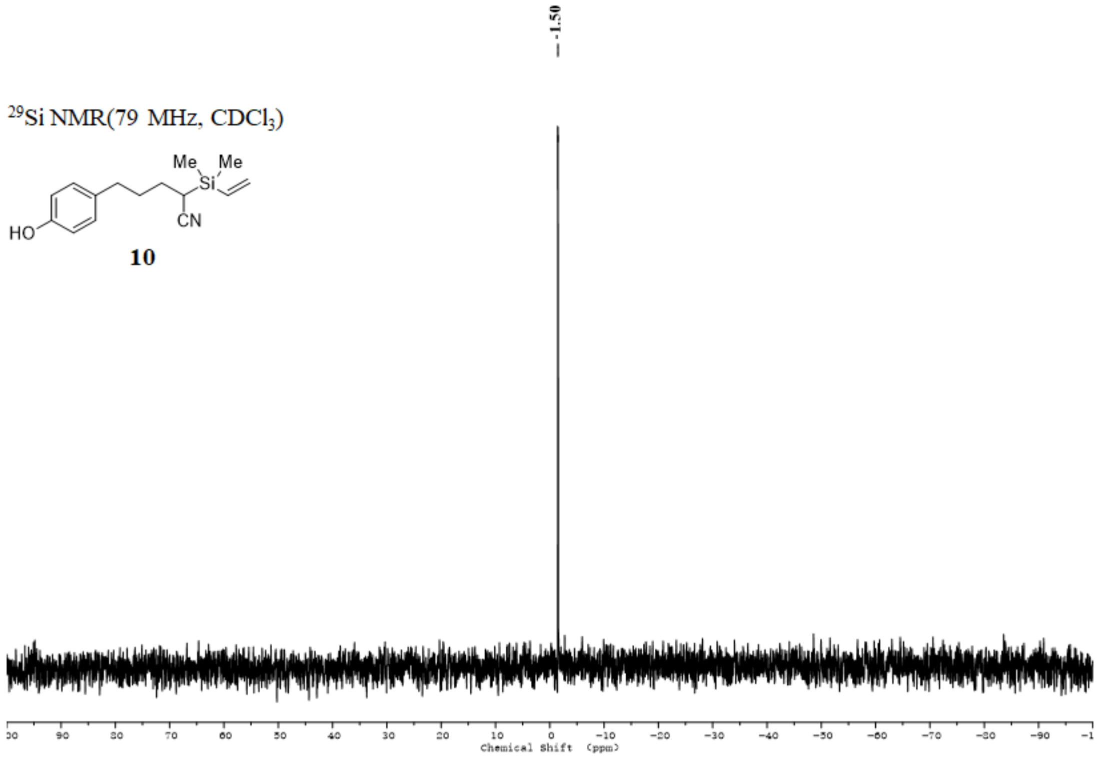

Supplementary Fig. 65. ^{29}Si NMR of compound 10. The sample has been recorded in 79 MHz, $CDCl_3$ at 25 °C

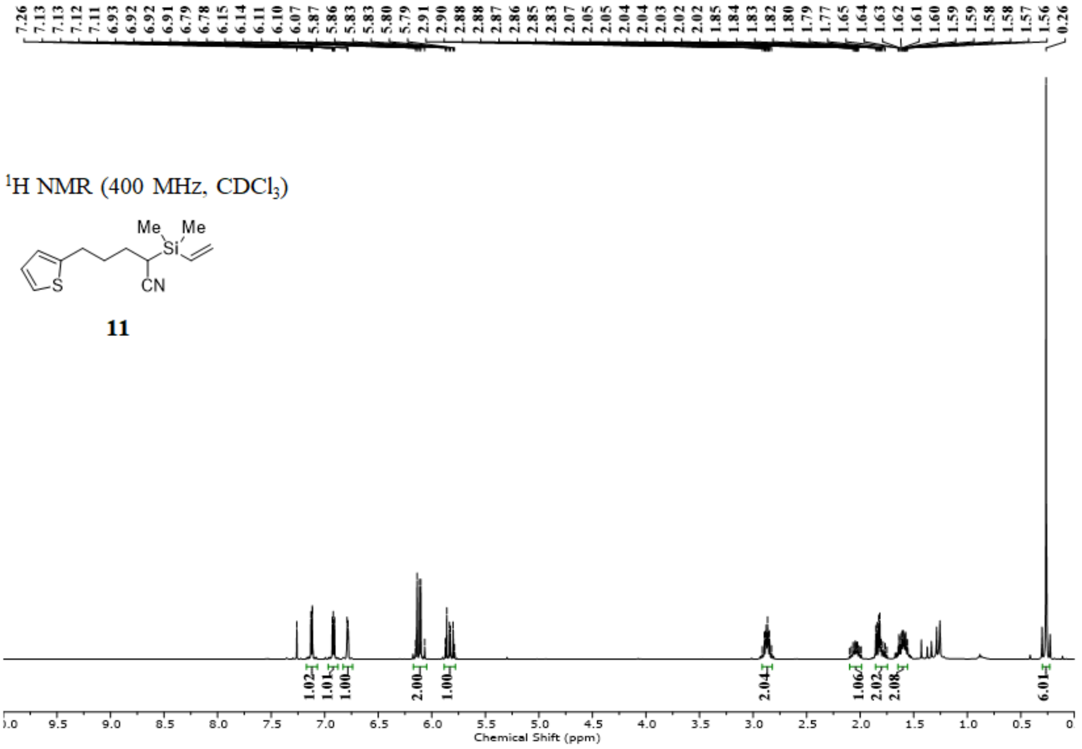

Supplementary Fig. 66. ^{1}H NMR of compound 11. The sample has been recorded in 400 MHz, $CDCl_3$ at 25 °C

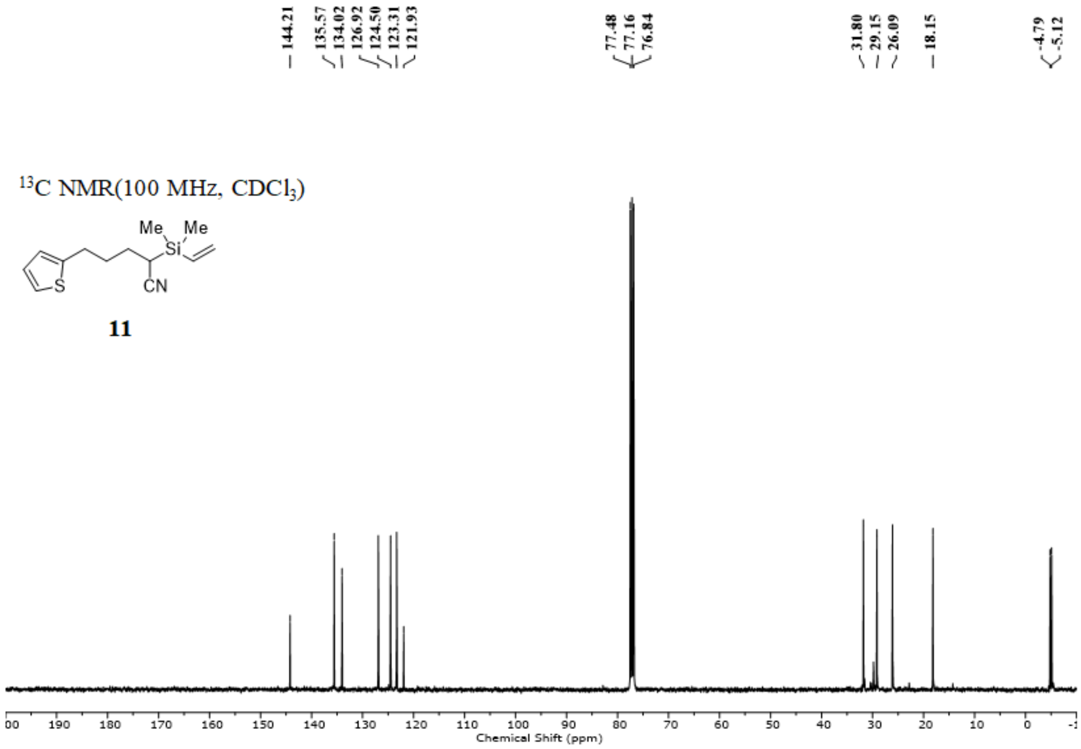

Supplementary Fig. 67. ^{13}C NMR of compound 11. The sample has been recorded in 100 MHz, $CDCl_3$ at 25 °C

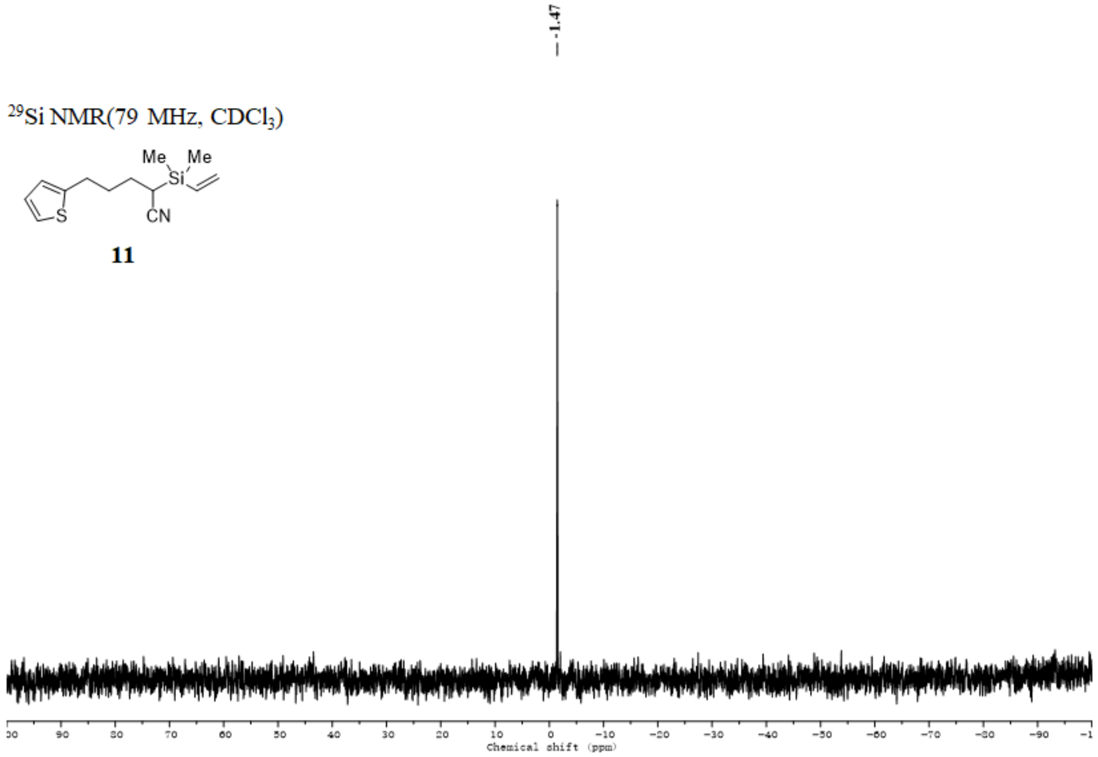

Supplementary Fig. 68. ^{29}Si NMR of compound 11. The sample has been recorded in 79 MHz, CDCl3 at 25 °C

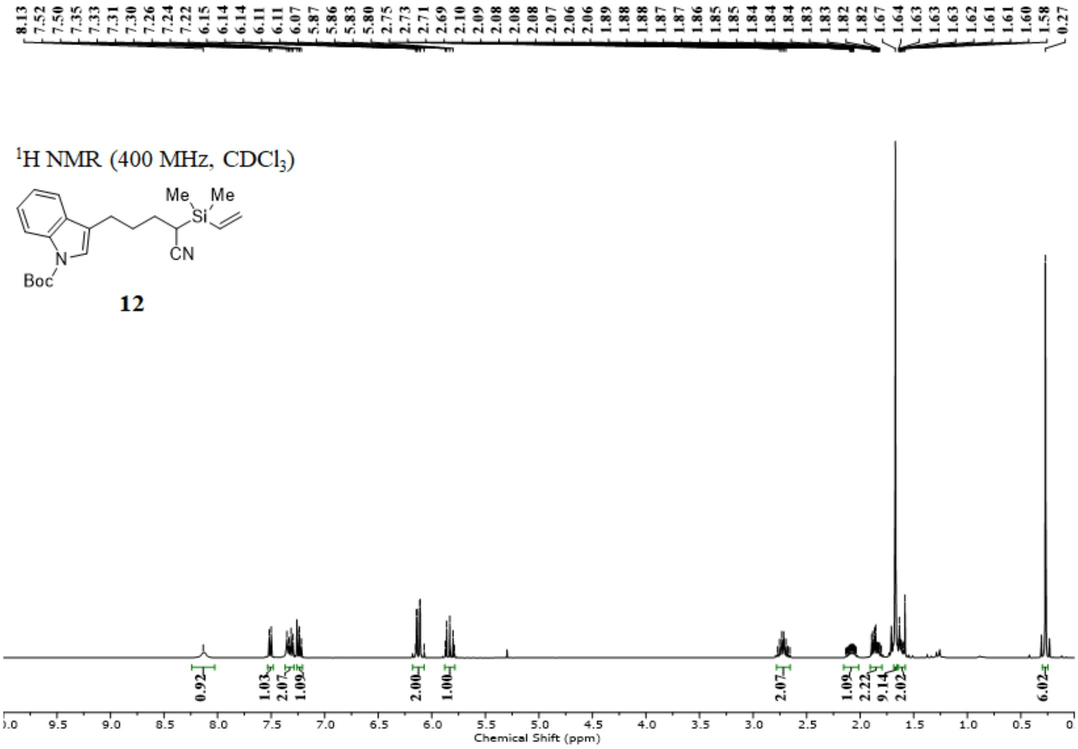

Supplementary Fig. 69. ^{1}H NMR of compound 12. The sample has been recorded in 400 MHz, CDCl3 at 25 °C

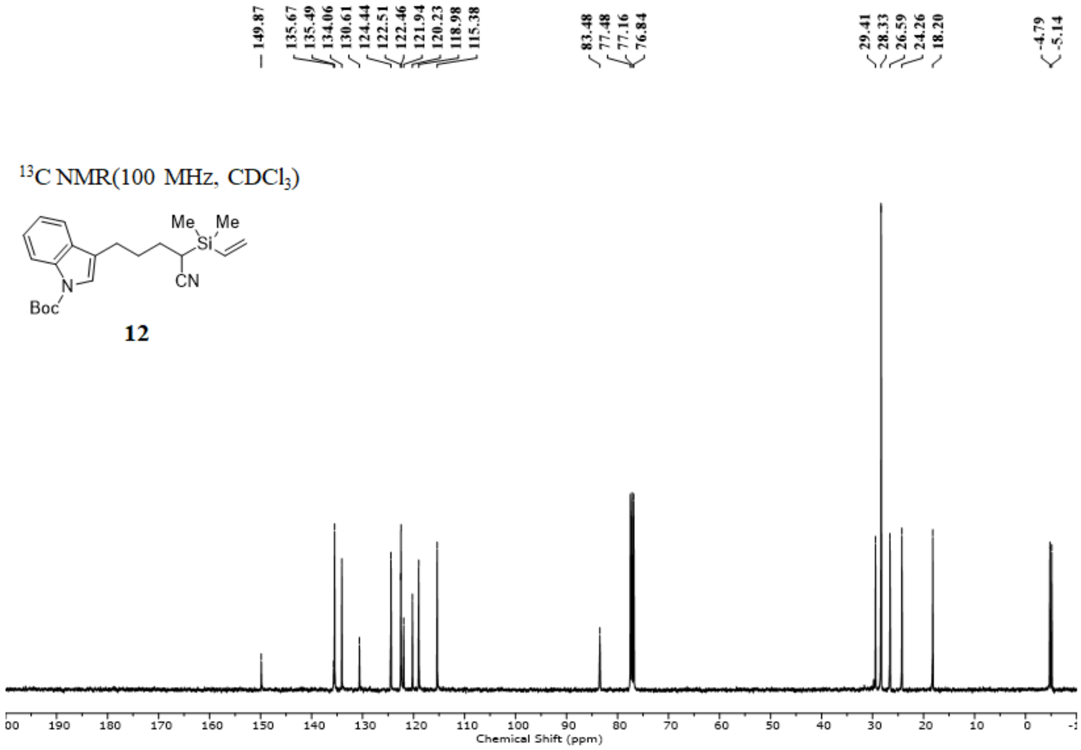

Supplementary Fig. 70. ^{13}C NMR of compound 12. The sample has been recorded in 100 MHz, $CDCl_3$ at 25 °C

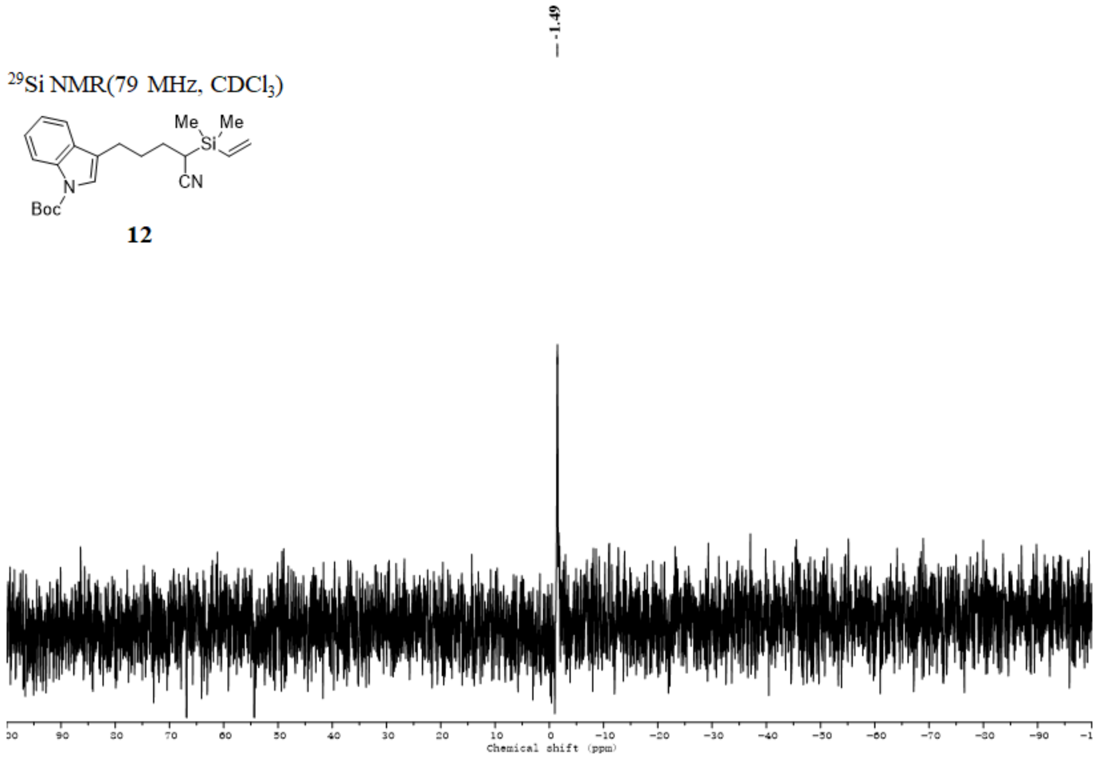

Supplementary Fig. 71. ^{29}Si NMR of compound 12. The sample has been recorded in 79 MHz, $CDCl_3$ at 25 °C

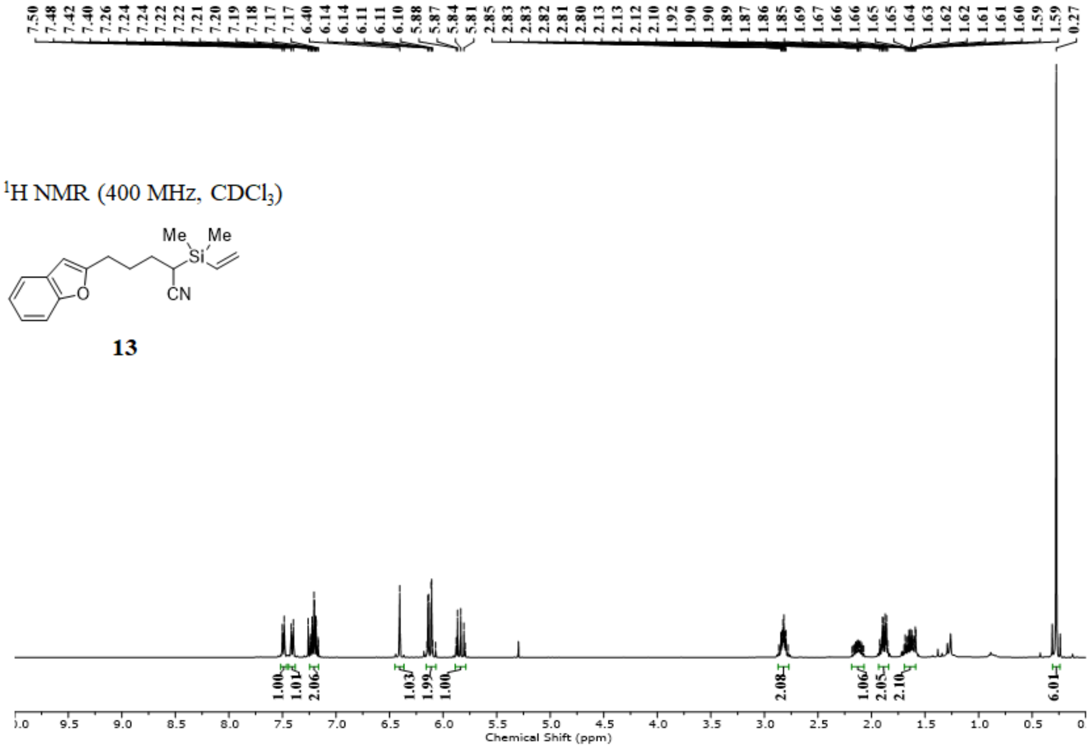

Supplementary Fig. 72. ^{1}H NMR of compound 13. The sample has been recorded in 400 MHz, $CDCl_3$ at 25 °C

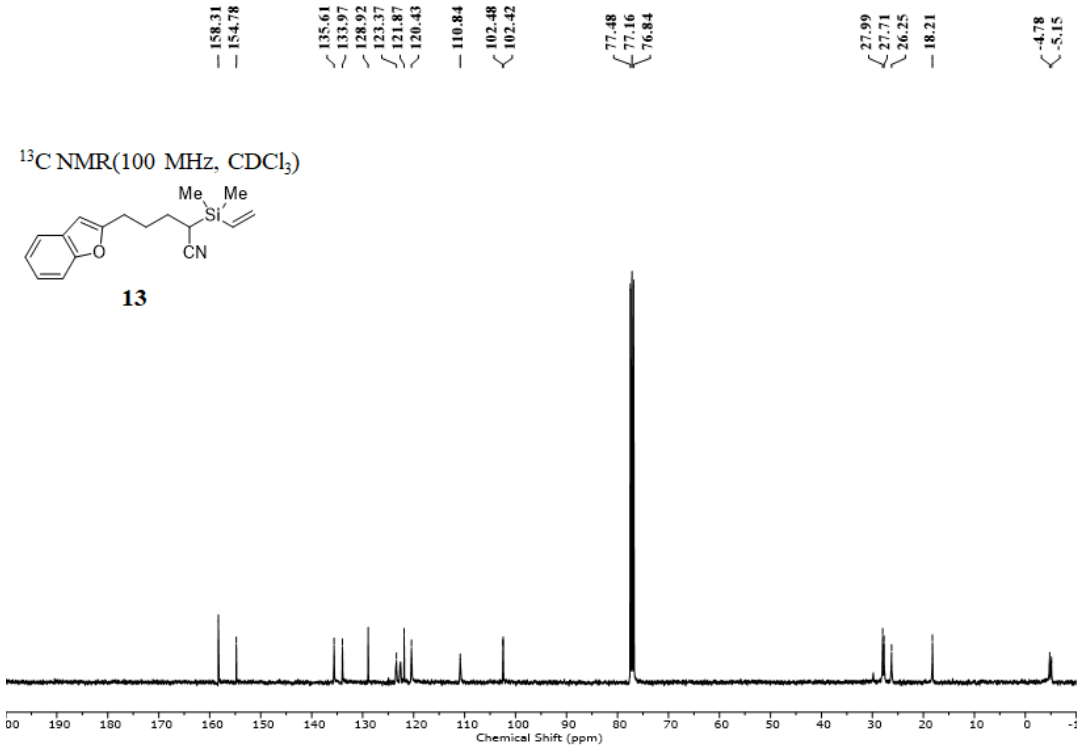

Supplementary Fig. 73. ^{13}C NMR of compound 14. The sample has been recorded in 100 MHz, $CDCl_3$ at 25 °C

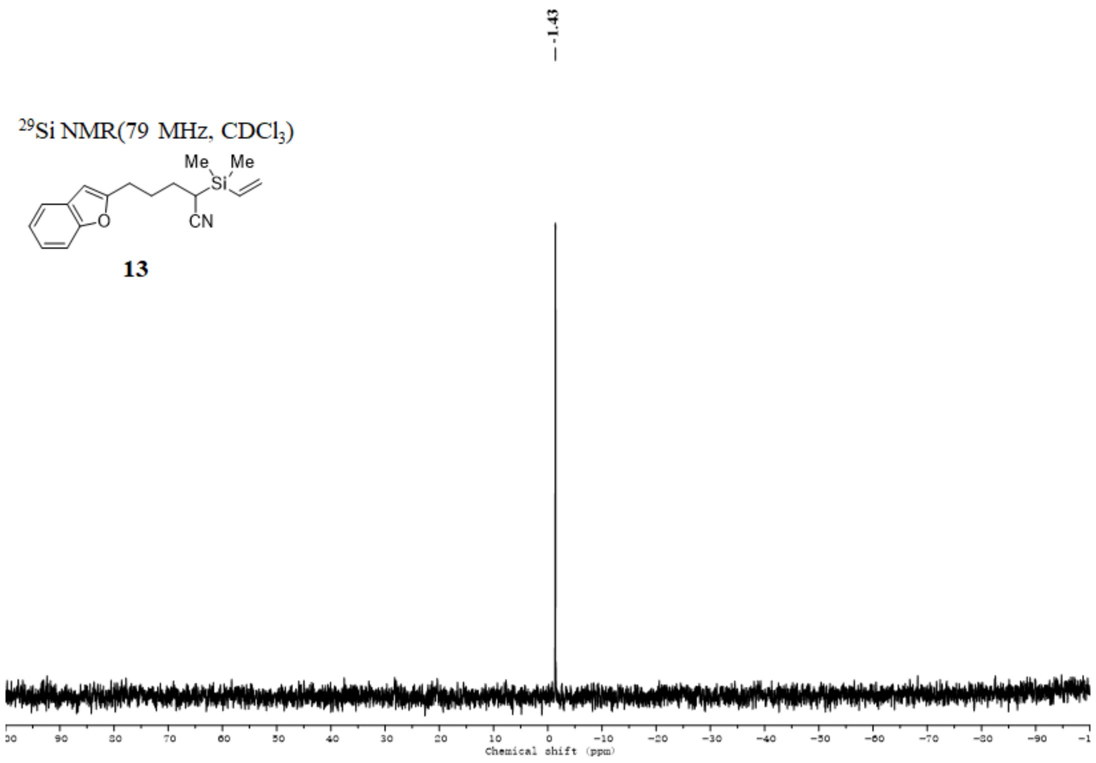

Supplementary Fig. 74. ^{29}Si NMR of compound 13. The sample has been recorded in 79 MHz, $CDCl_3$ at 25 °C

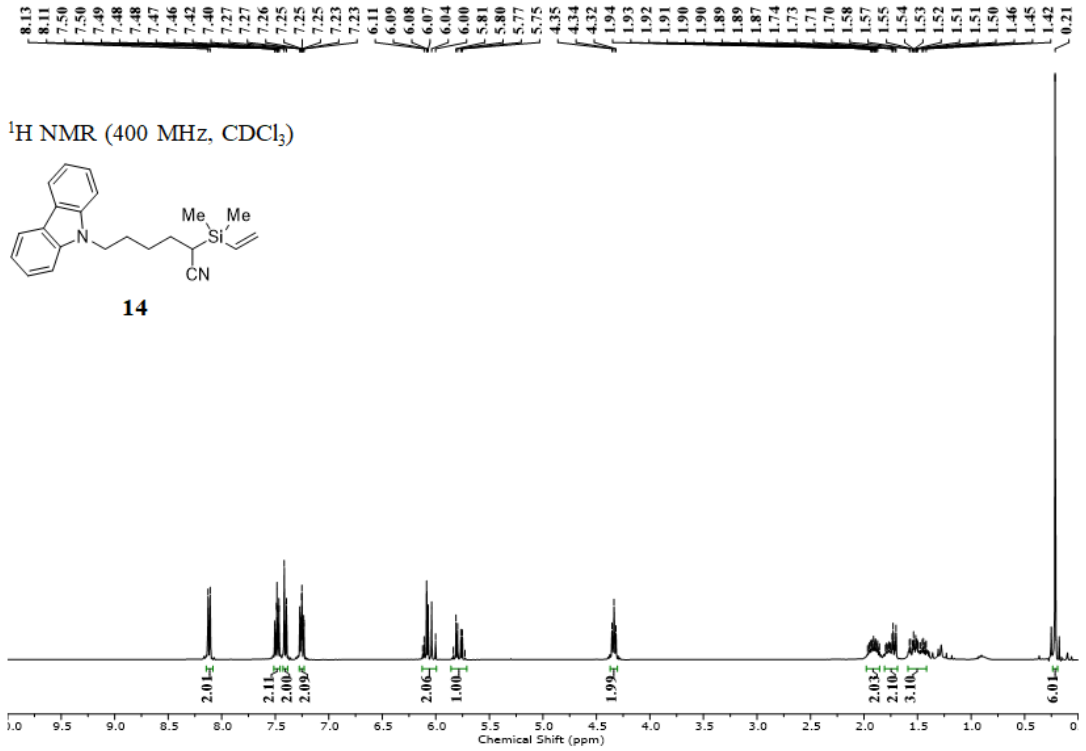

Supplementary Fig. 75. ^{1}H NMR of compound 14. The sample has been recorded in 400 MHz, $CDCl_3$ at 25 °C

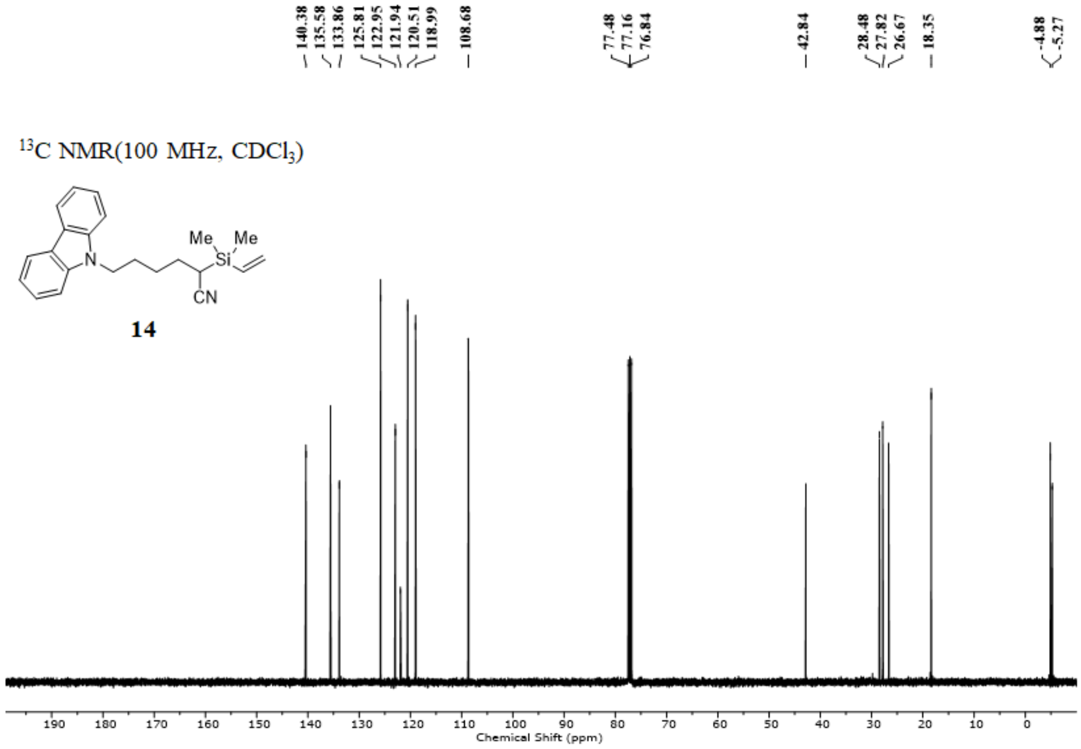

Supplementary Fig. 76. ^{13}C NMR of compound 14. The sample has been recorded in 100 MHz, $CDCl_3$ at 25 °C

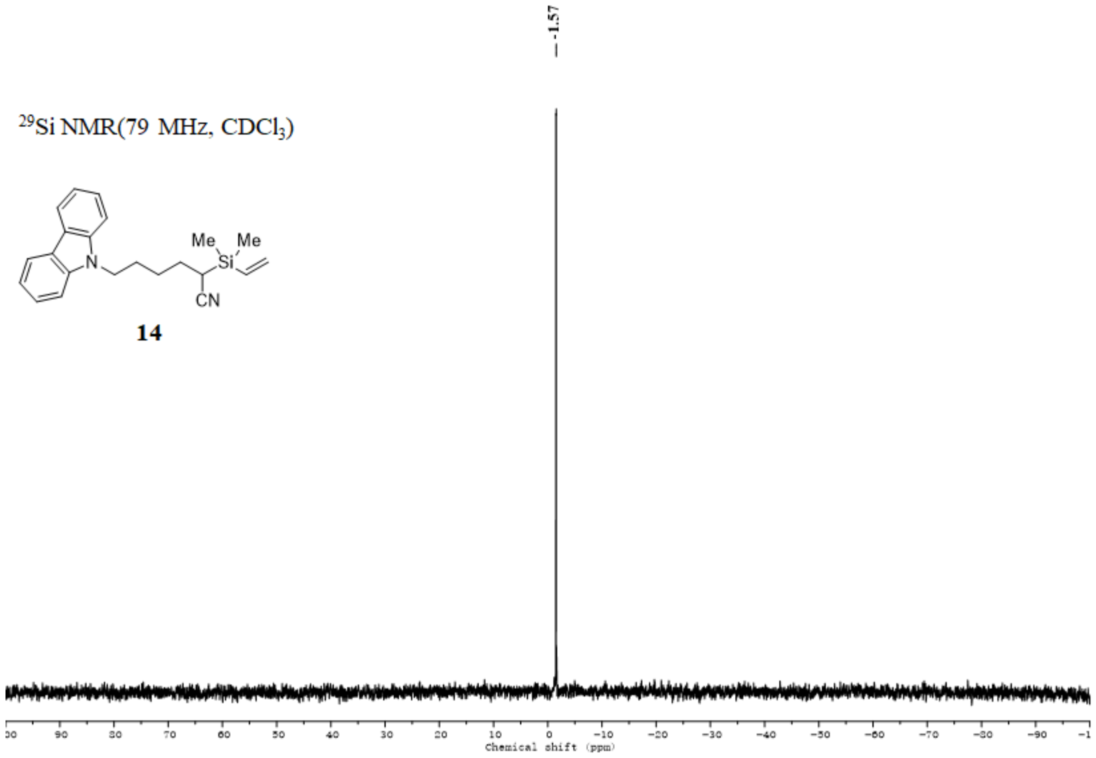

Supplementary Fig. 77. ^{29}Si NMR of compound 14. The sample has been recorded in 79 MHz, $CDCl_3$ at 25 °C

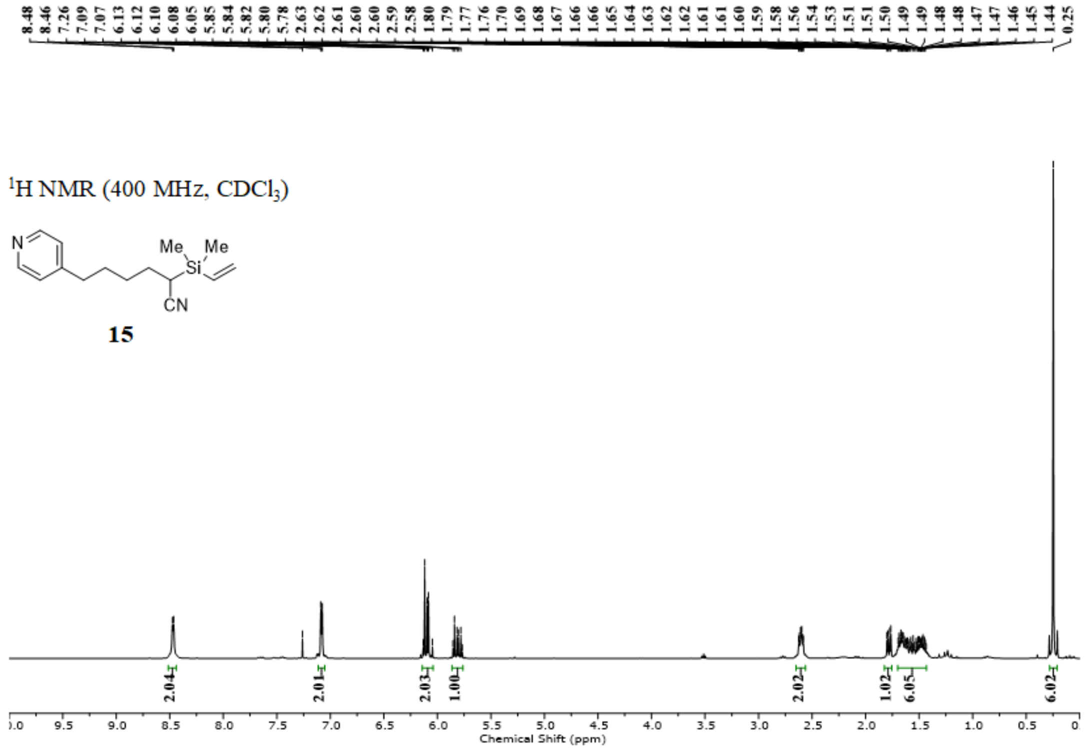

Supplementary Fig. 78. ^{1}H NMR of compound 15. The sample has been recorded in 400 MHz, $CDCl_3$ at 25 °C

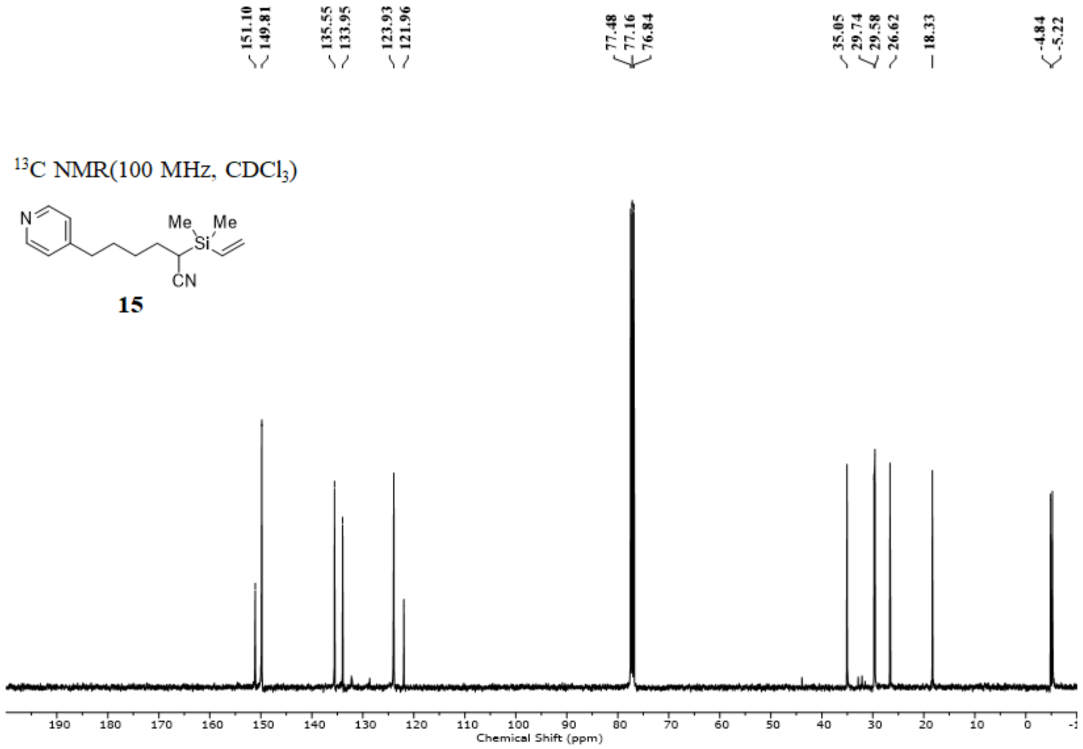

Supplementary Fig. 79. ^{13}C NMR of compound 15. The sample has been recorded in 100 MHz, $CDCl_3$ at 25 °C

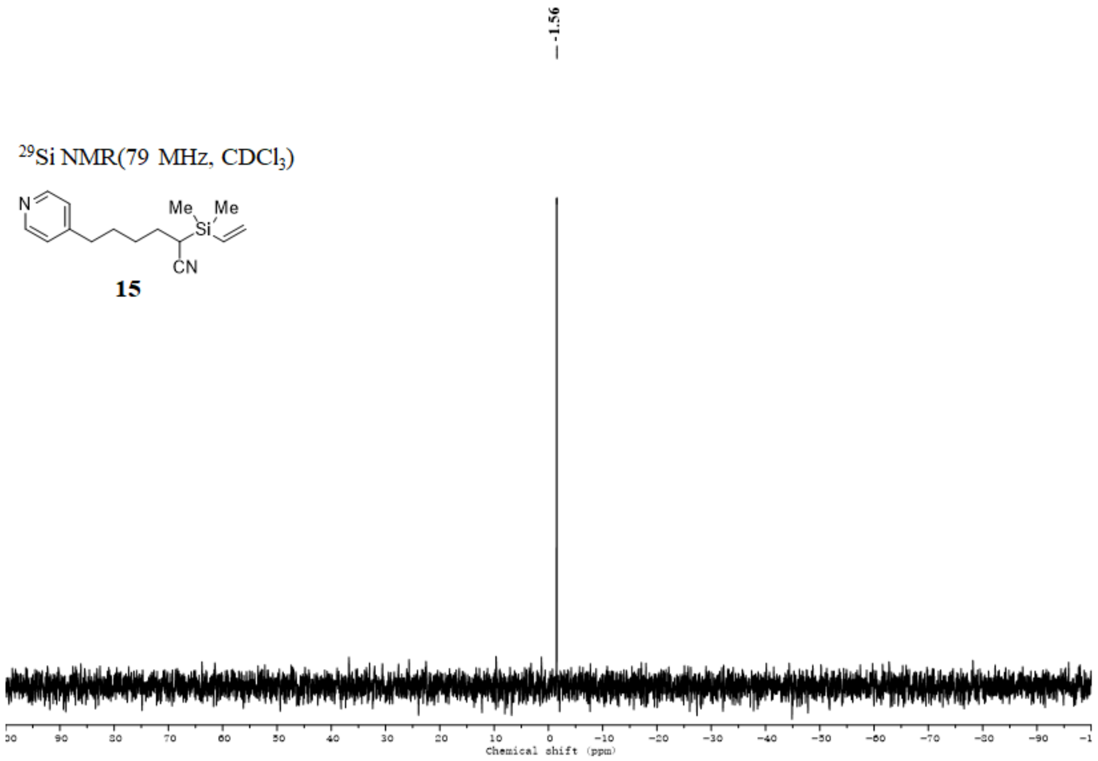

Supplementary Fig. 80. ^{29}Si NMR of compound 15. The sample has been recorded in 79 MHz, $CDCl_3$ at 25 °C

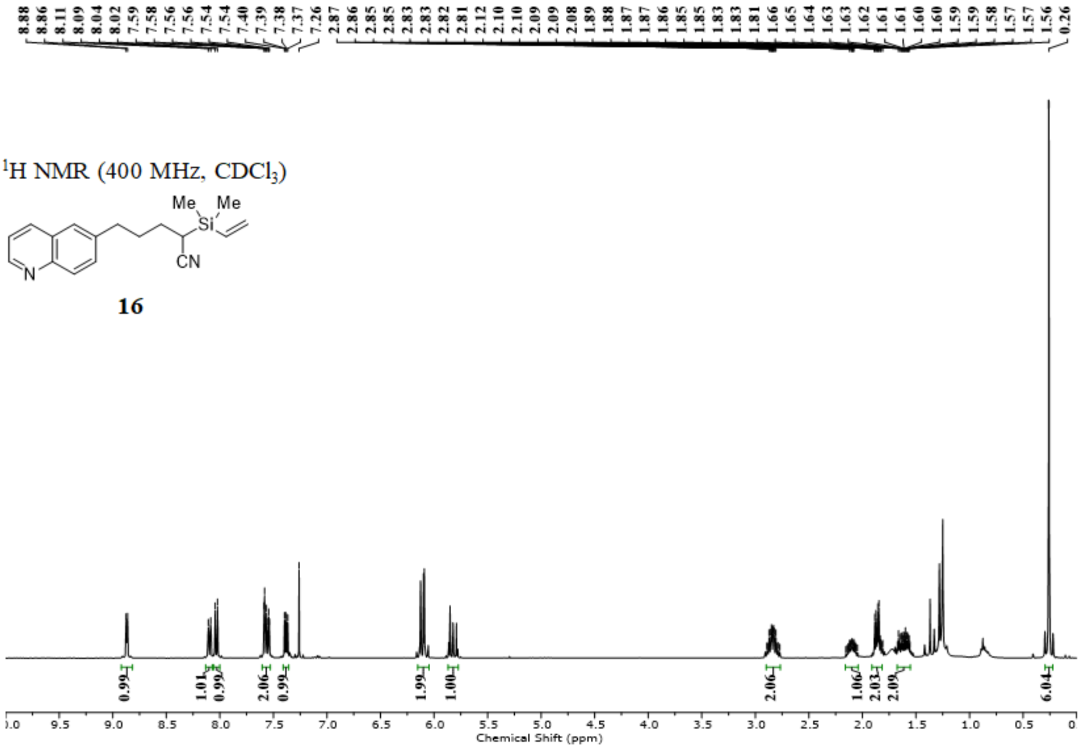

Supplementary Fig. 81. ^{1}H NMR of compound 16 The sample has been recorded in 400 MHz, $CDCl_3$ at 25 °C

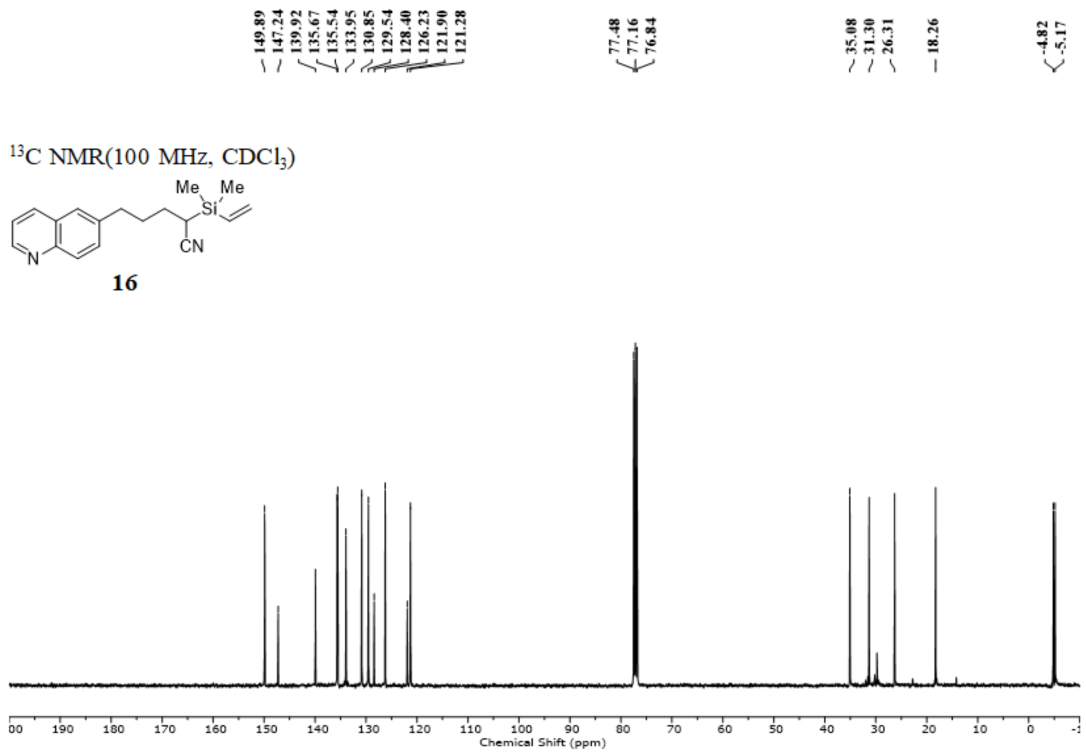

Supplementary Fig. 82. ^{13}C NMR of compound 16. The sample has been recorded in 100 MHz, $CDCl_3$ at 25 °C

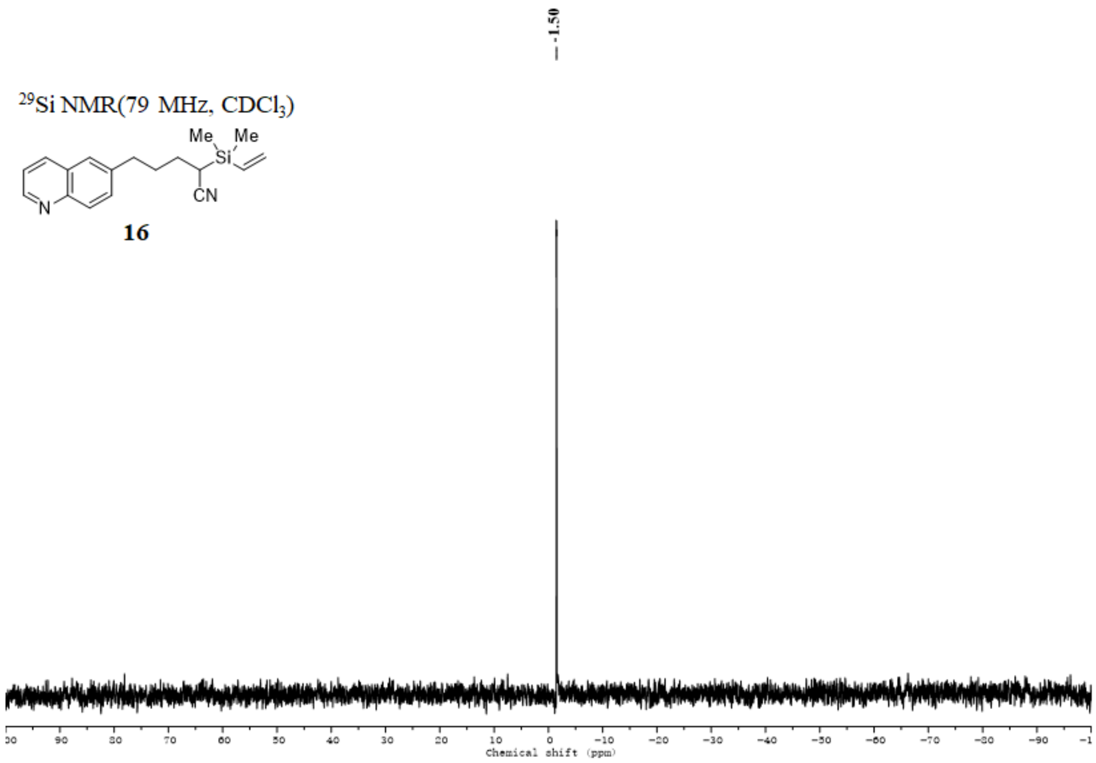

Supplementary Fig. 83. ^{29}Si NMR of compound 16. The sample has been recorded in 79 MHz, $CDCl_3$ at 25 °C

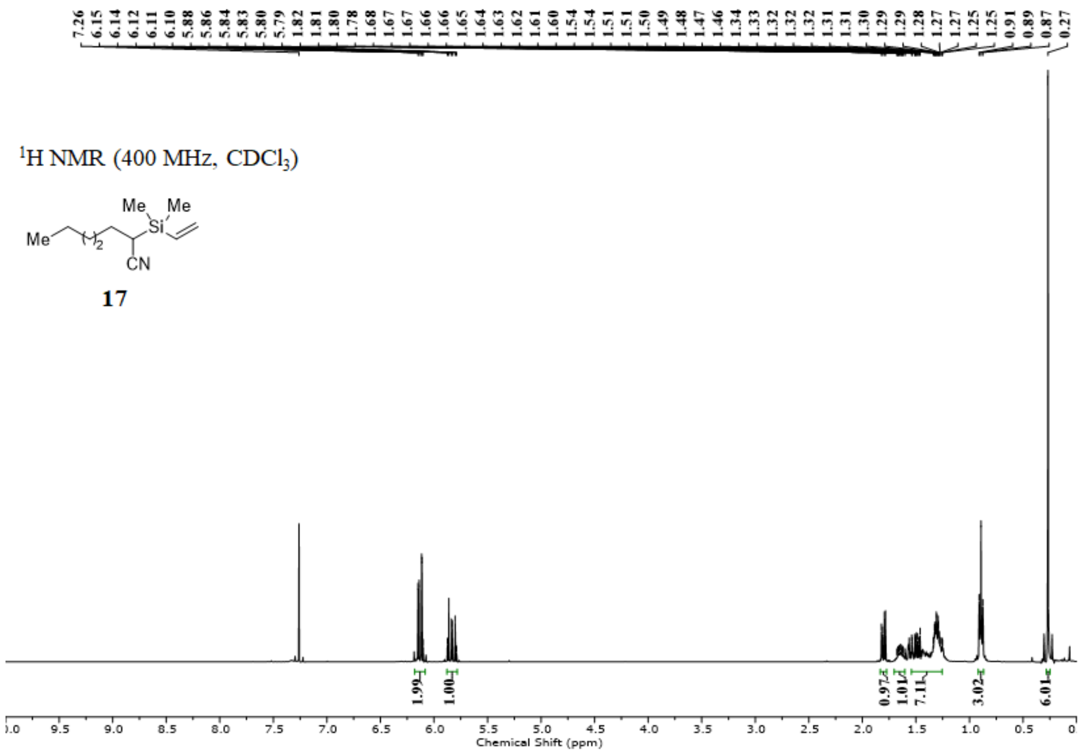

Supplementary Fig. 84. ^{1}H NMR of compound 17. The sample has been recorded in 400 MHz, $CDCl_3$ at 25 °C

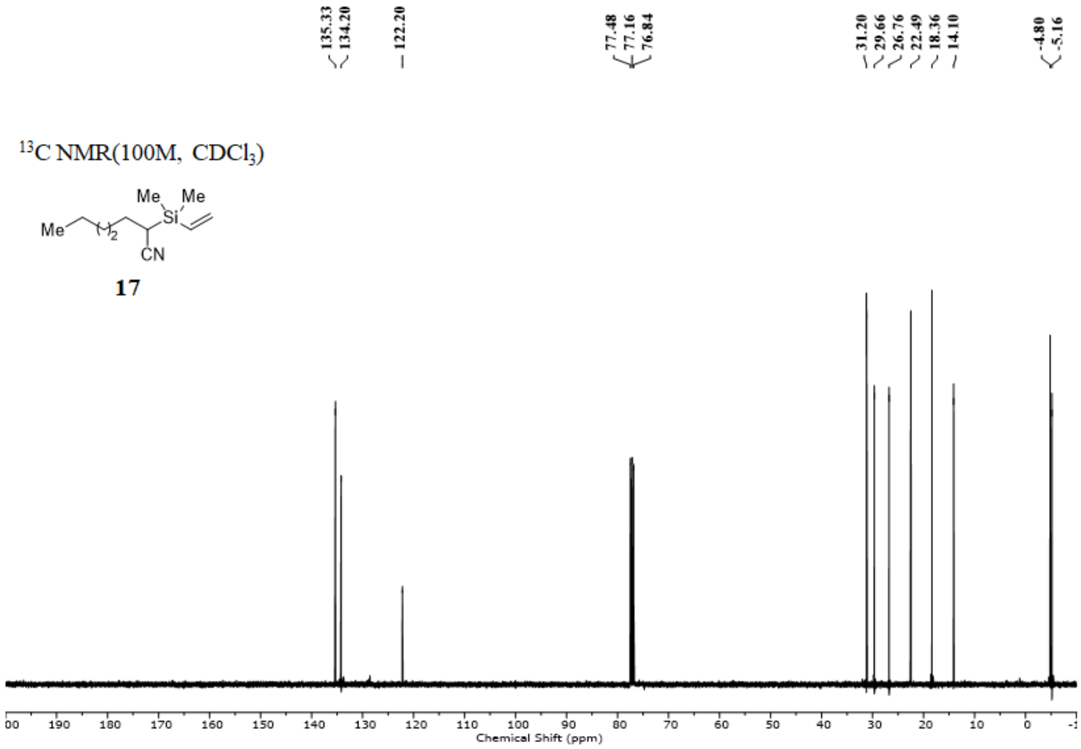

Supplementary Fig. 85. ^{13}C NMR of compound 17. The sample has been recorded in 100 MHz, $CDCl_3$ at 25 °C

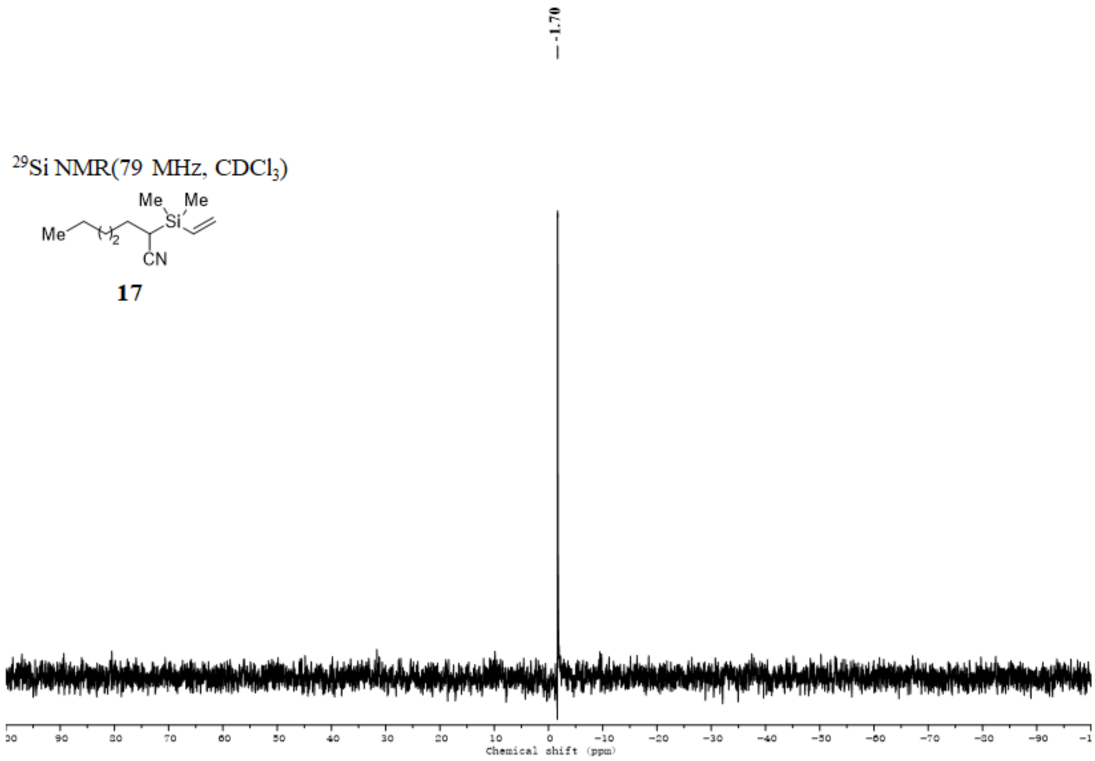

Supplementary Fig. 86. ^{29}Si NMR of compound 17. The sample has been recorded in 79 MHz, $CDCl_3$ at 25 °C

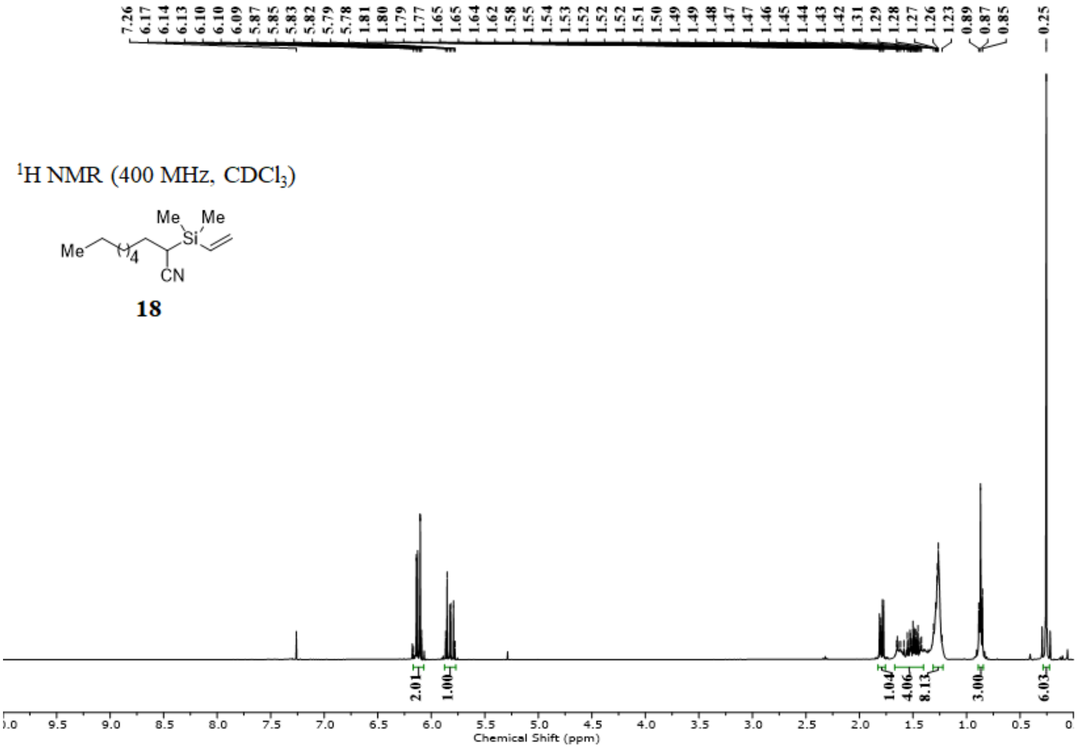

Supplementary Fig. 87. ^{1}H NMR of compound 18. The sample has been recorded in 400 MHz, $CDCl_3$ at 25 °C

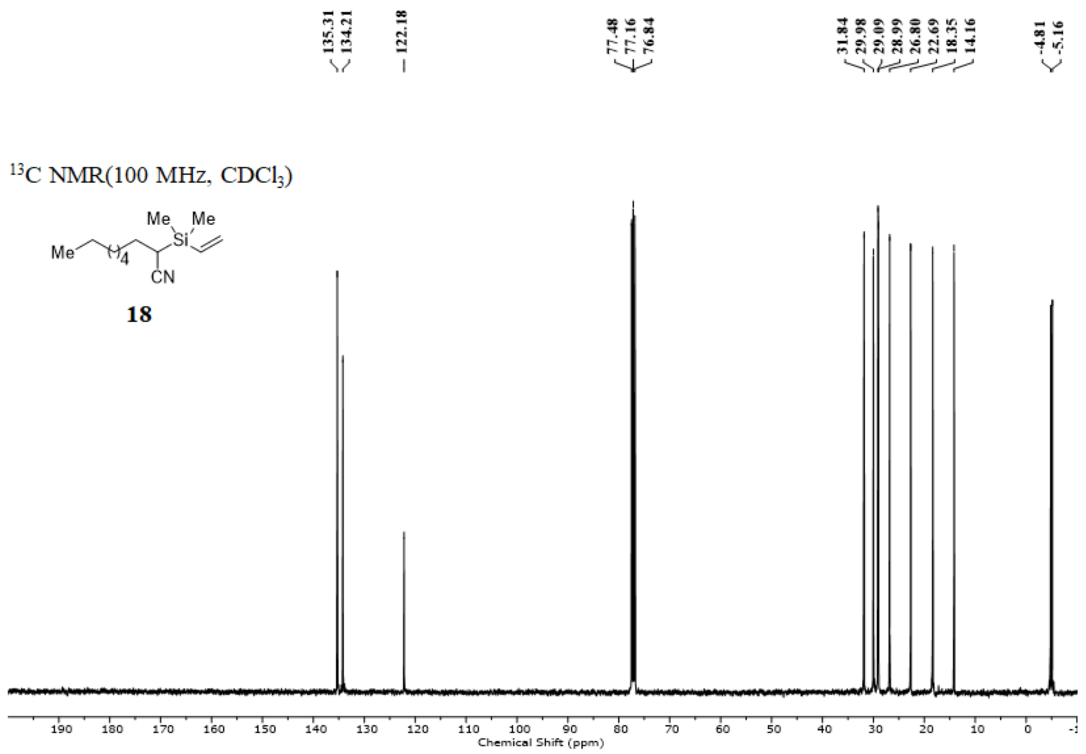

Supplementary Fig. 88. ^{13}C NMR of compound 18. The sample has been recorded in 100 MHz, $CDCl_3$ at 25 °C

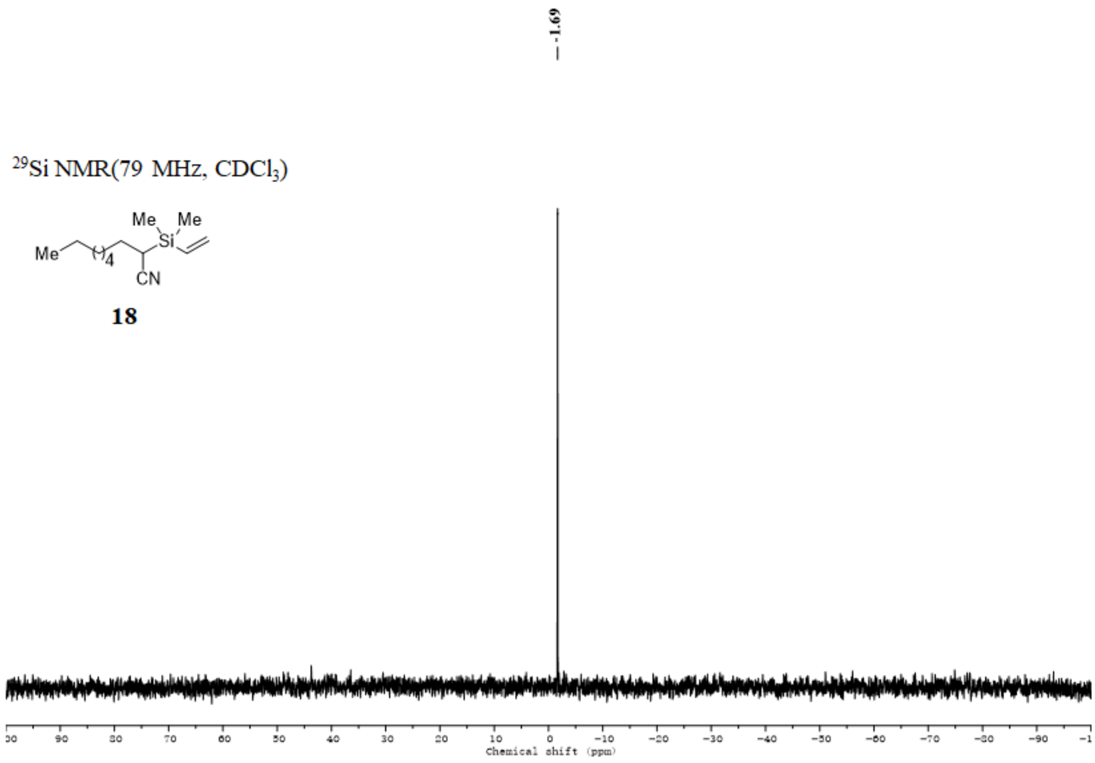

Supplementary Fig. 89. ^{29}Si NMR of compound 18. The sample has been recorded in 79 MHz, $CDCl_3$ at 25 °C

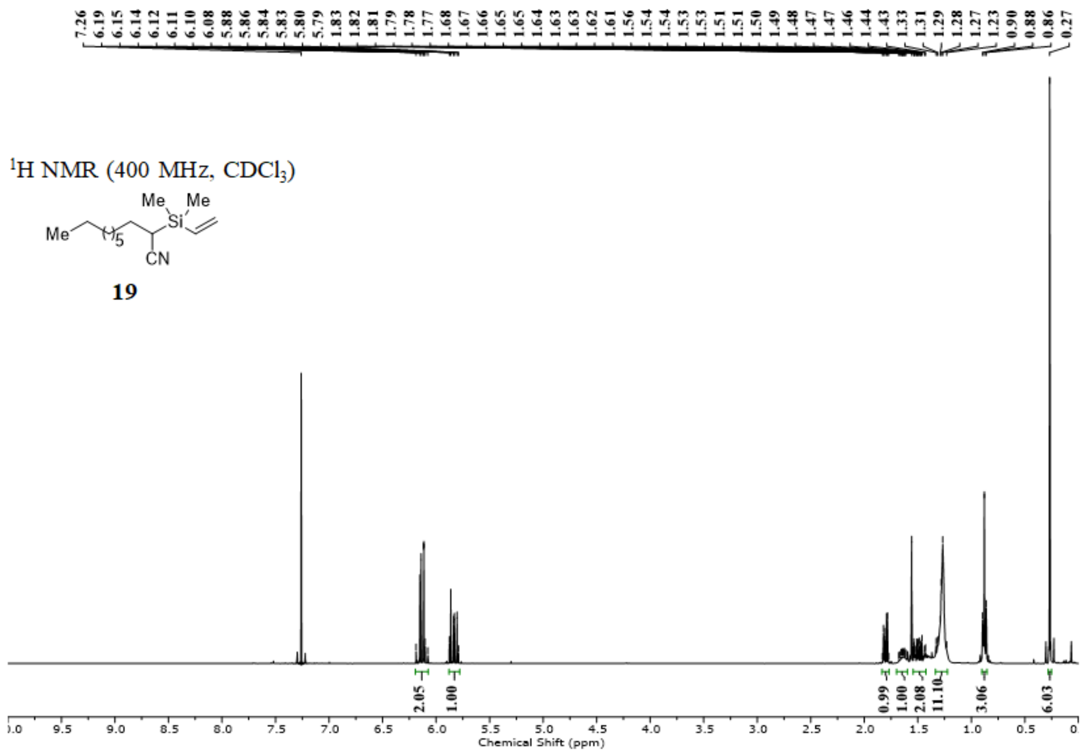

Supplementary Fig. 90. ^{1}H NMR of compound 19. The sample has been recorded in 400 MHz, $CDCl_3$ at 25 °C

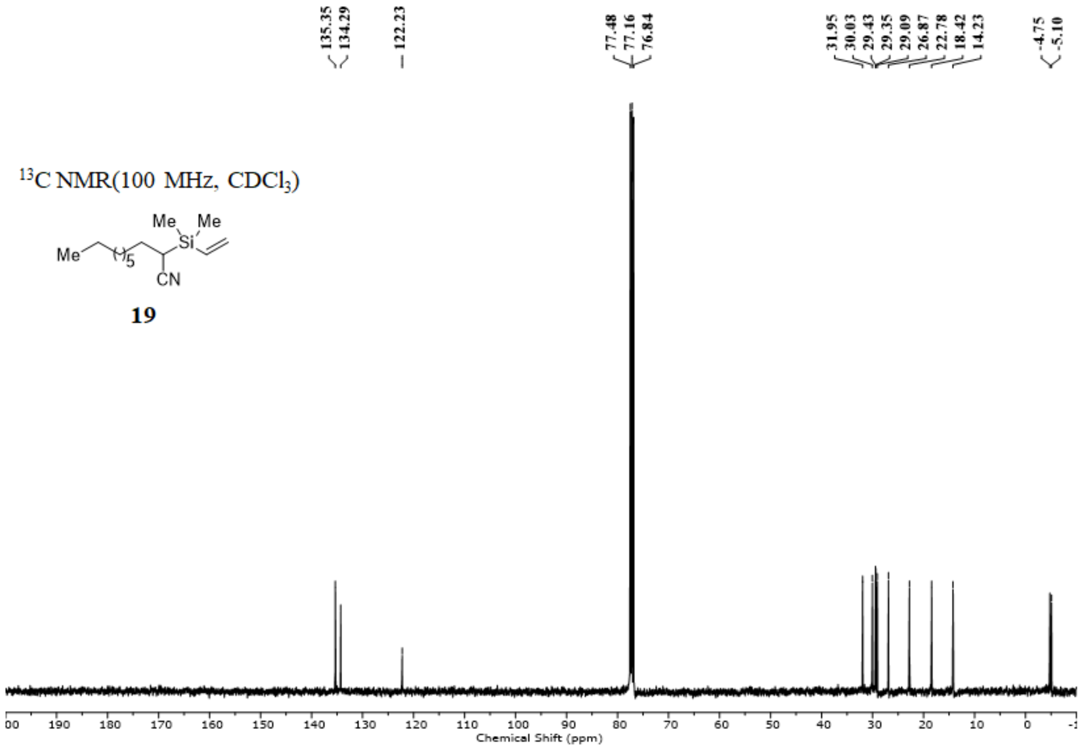

Supplementary Fig. 91. ^{13}C NMR of compound 19. The sample has been recorded in 100 MHz, $CDCl_3$ at 25 °C

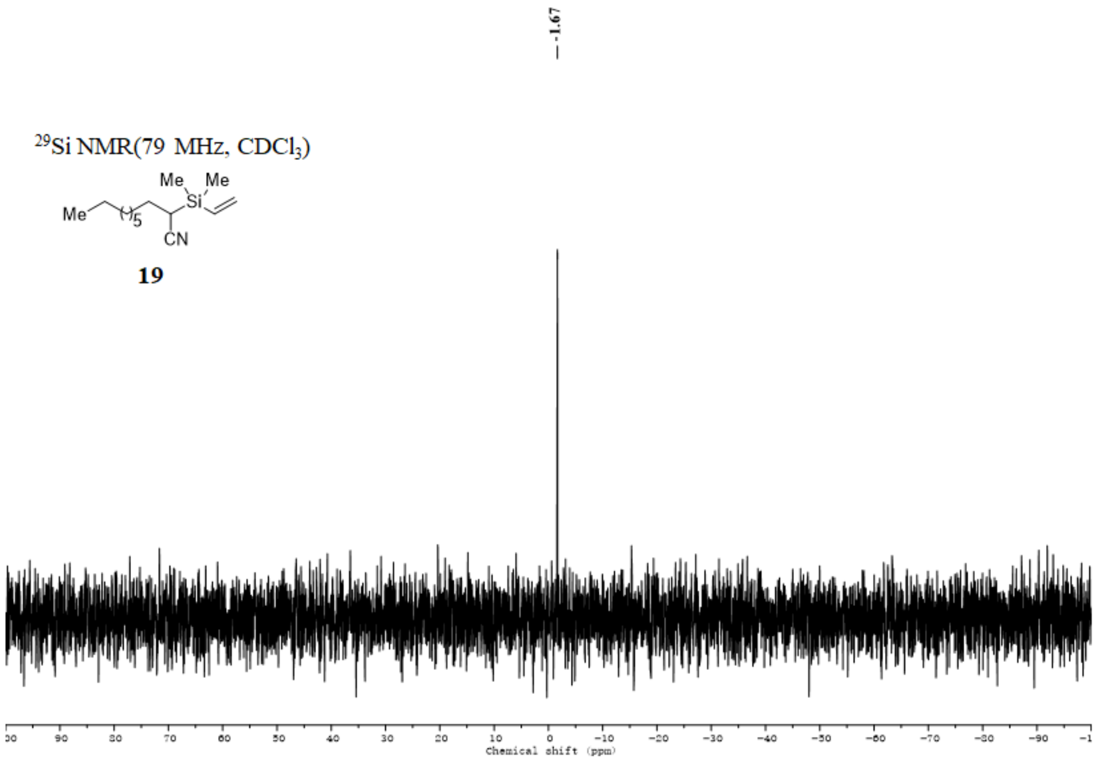

Supplementary Fig. 92. ^{29}Si NMR of compound 19. The sample has been recorded in 79 MHz, $CDCl_3$ at 25 °C

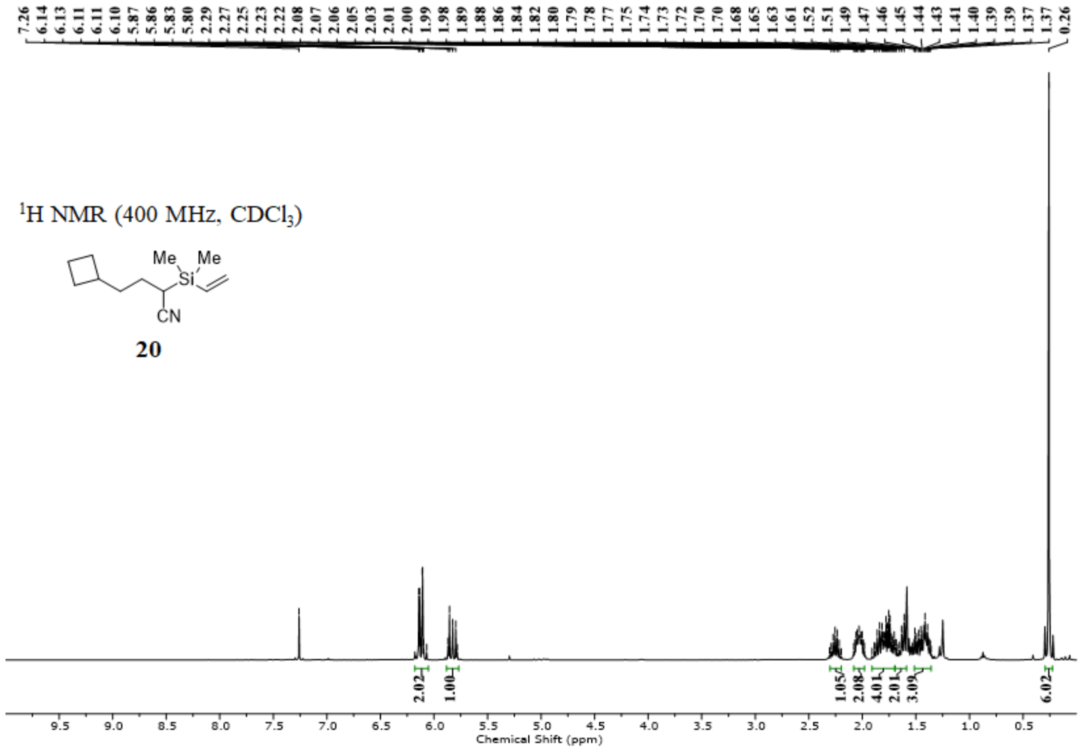

Supplementary Fig. 93. ^{1}H NMR of compound 20. The sample has been recorded in 400 MHz, $CDCl_3$ at 25 °C

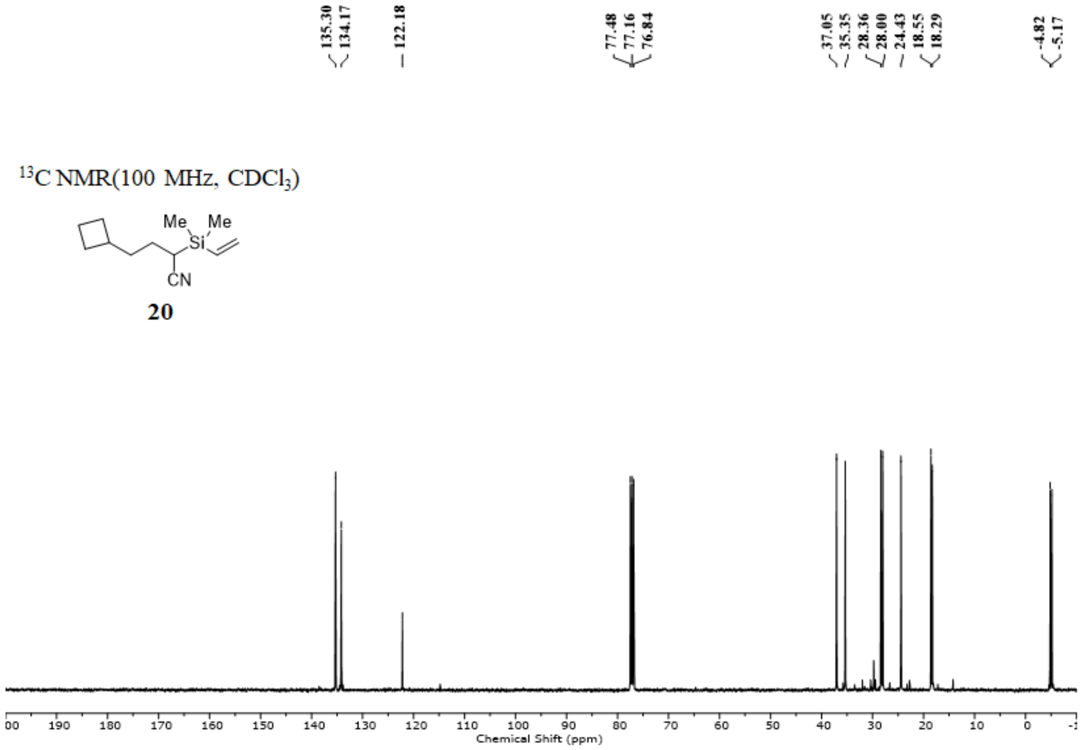

Supplementary Fig. 94. ^{13}C NMR of compound 20. The sample has been recorded in 100 MHz, $CDCl_3$ at 25 °C

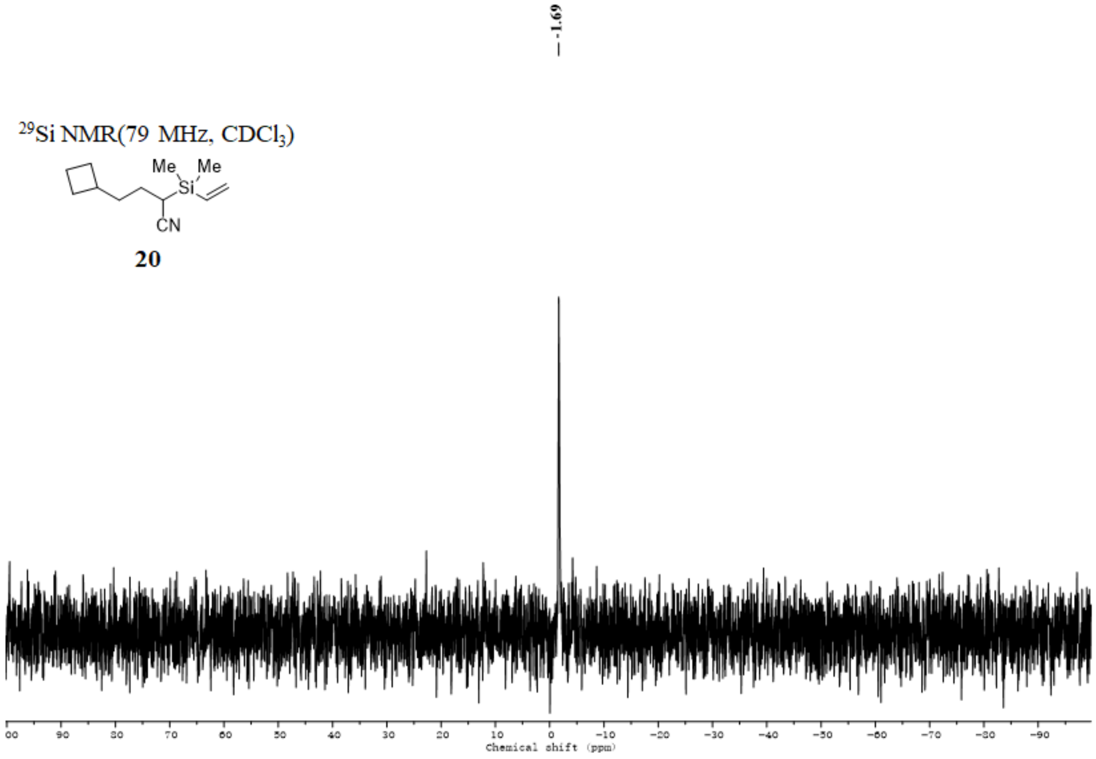

Supplementary Fig. 95. ^{29}Si NMR of compound 20. The sample has been recorded in 79 MHz, $CDCl_3$ at 25 °C

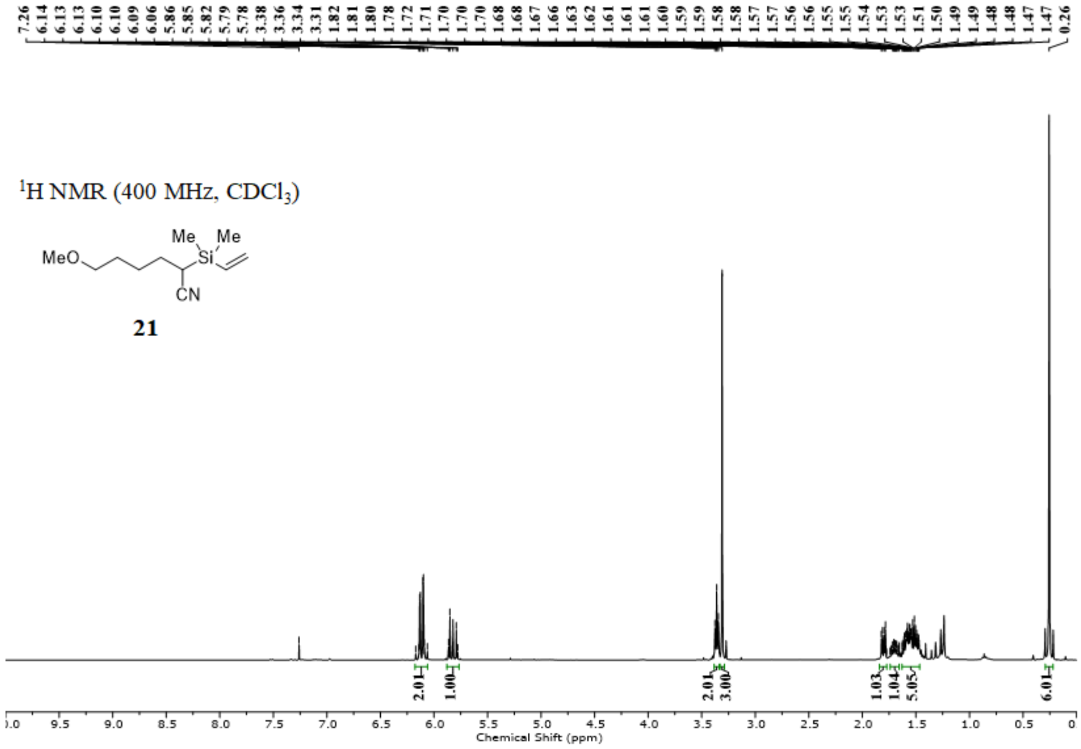

Supplementary Fig. 96. ^{1}H NMR of compound 21. The sample has been recorded in 400 MHz, $CDCl_3$ at 25 °C

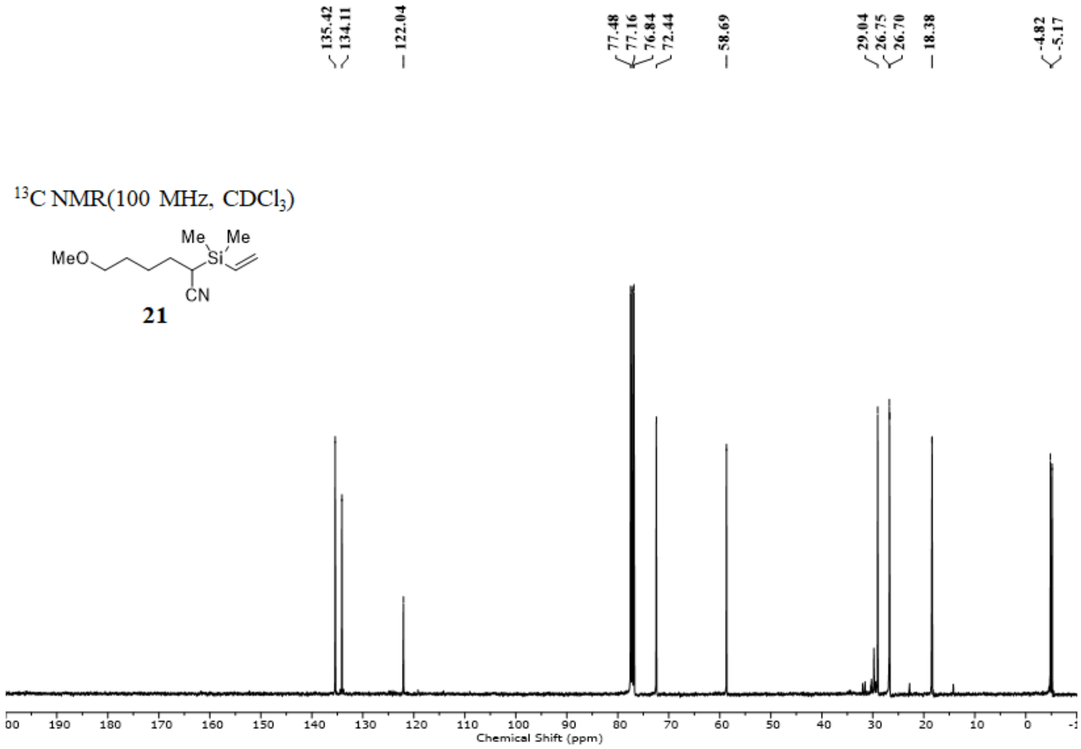

Supplementary Fig. 97. ^{13}C NMR of compound 21. The sample has been recorded in 100 MHz, $CDCl_3$ at 25 °C

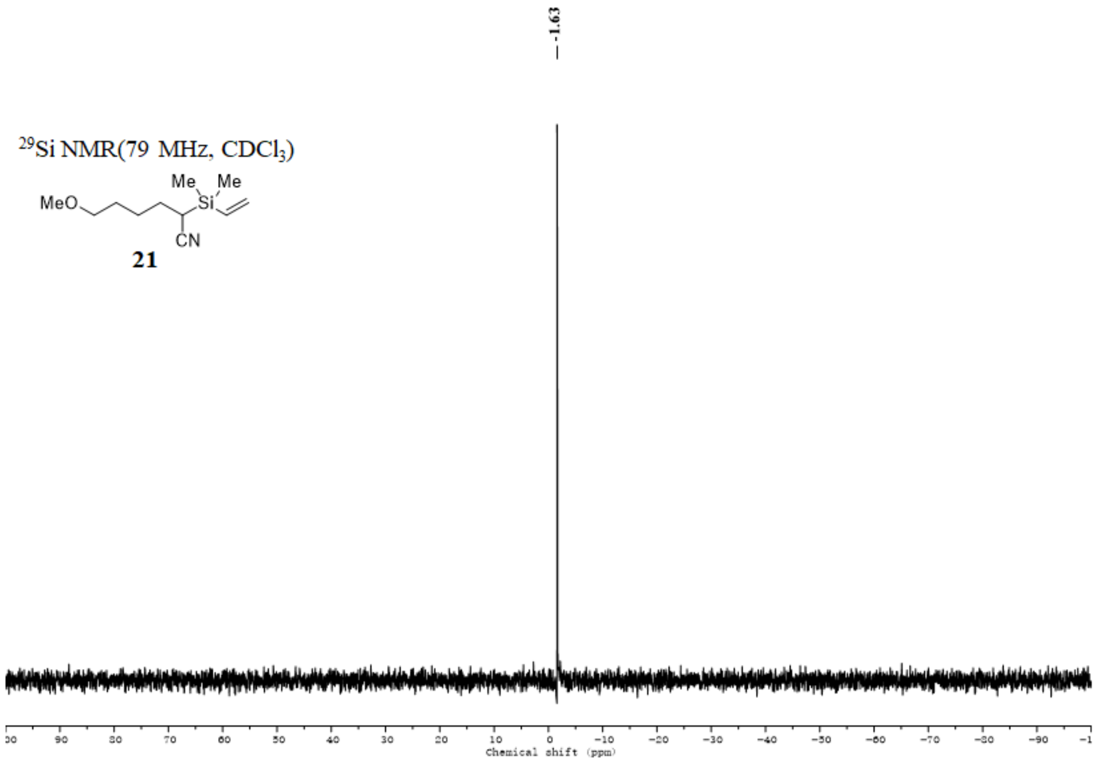

Supplementary Fig. 98. ^{29}Si NMR of compound 21. The sample has been recorded in 79 MHz, $CDCl_3$ at 25 °C

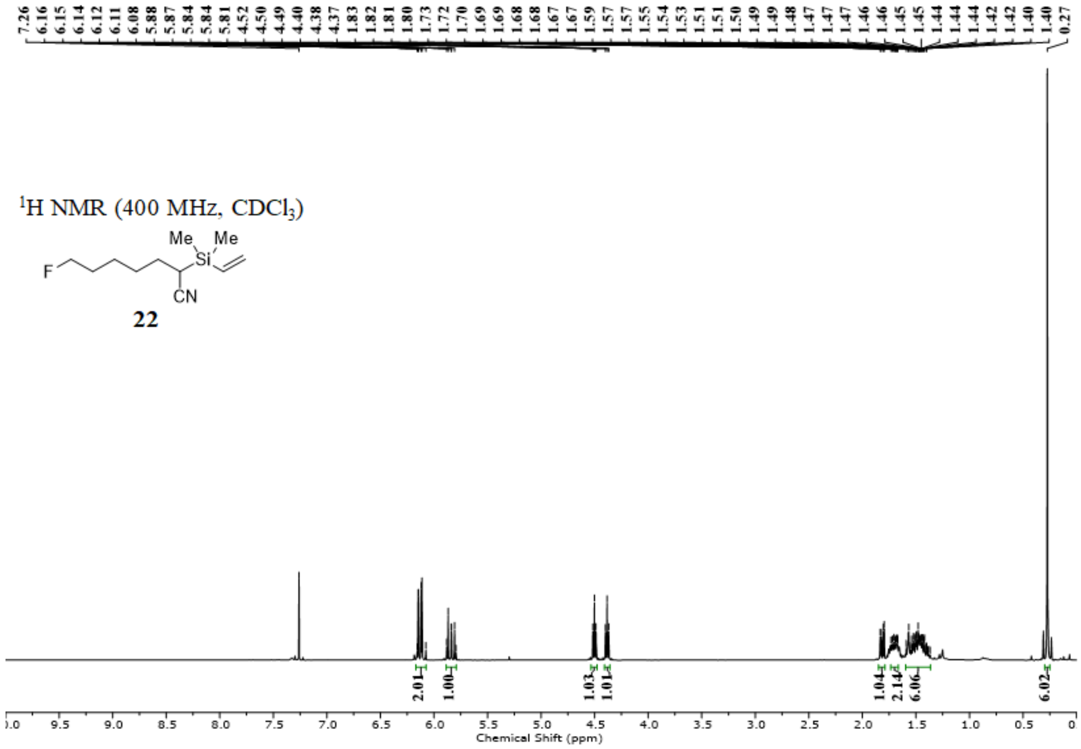

Supplementary Fig. 99. ^{1}H NMR of compound 22. The sample has been recorded in 400 MHz, $CDCl_3$ at 25 °C

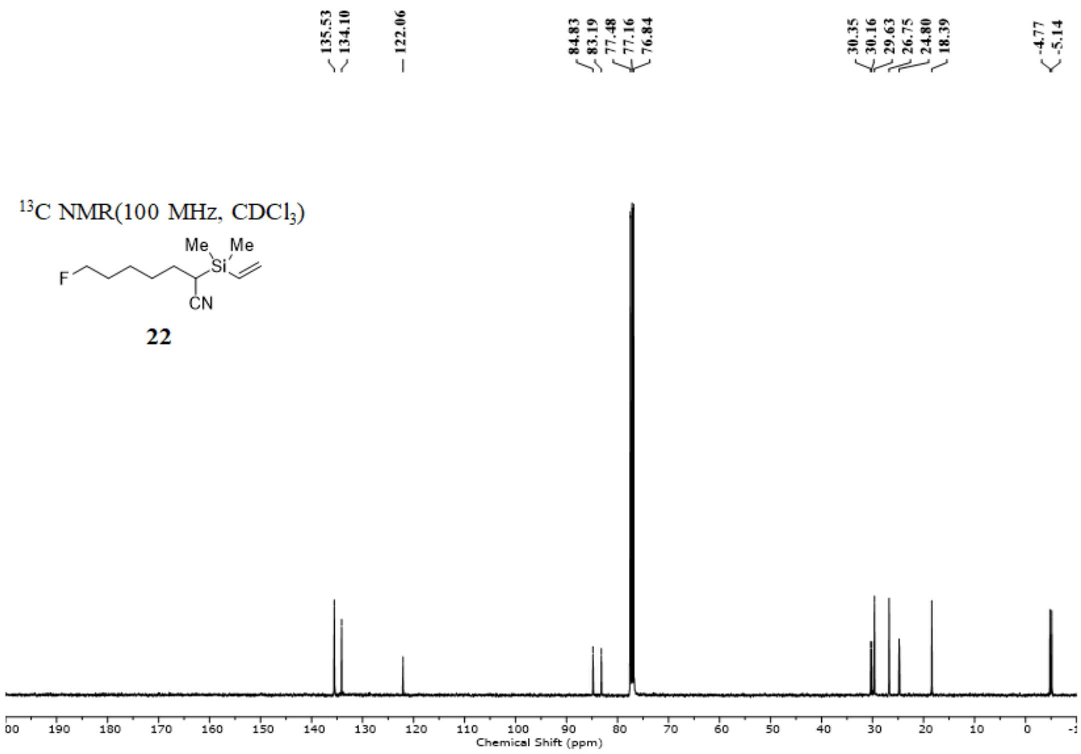

Supplementary Fig. 100. ^{13}C NMR of compound 22. The sample has been recorded in 100 MHz, $CDCl_3$ at 25 °C

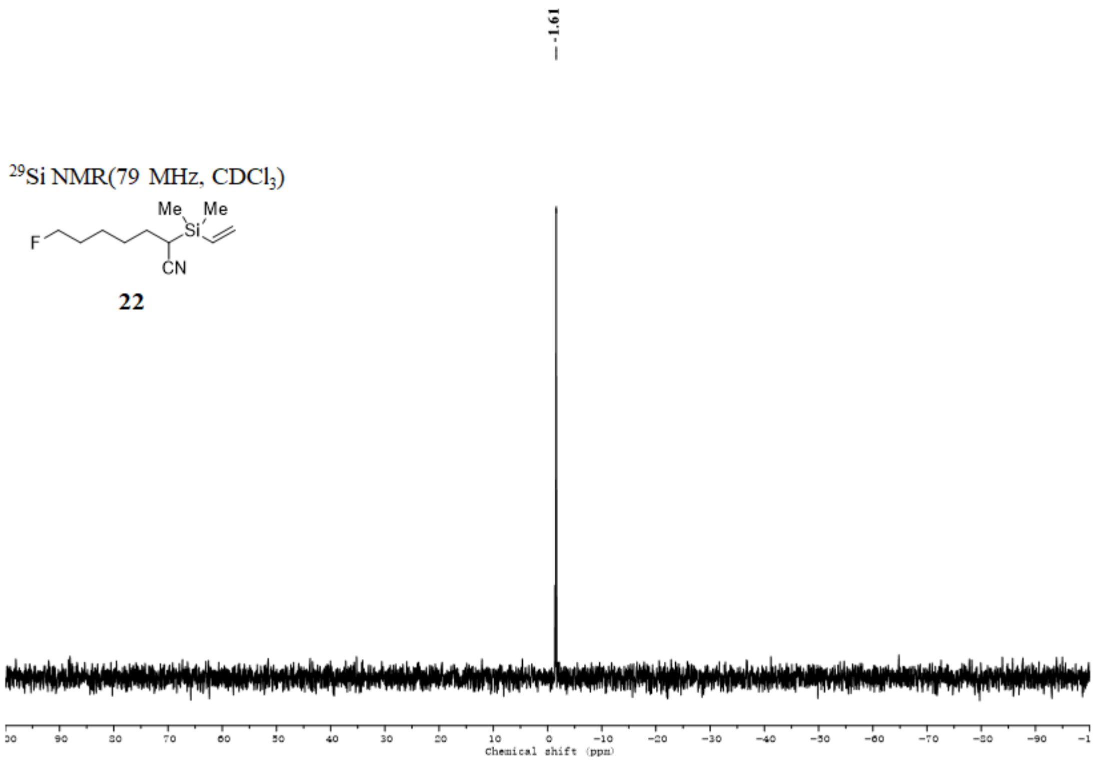

Supplementary Fig. 101. ^{29}Si NMR of compound 22. The sample has been recorded in 79 MHz, $CDCl_3$ at 25 °C

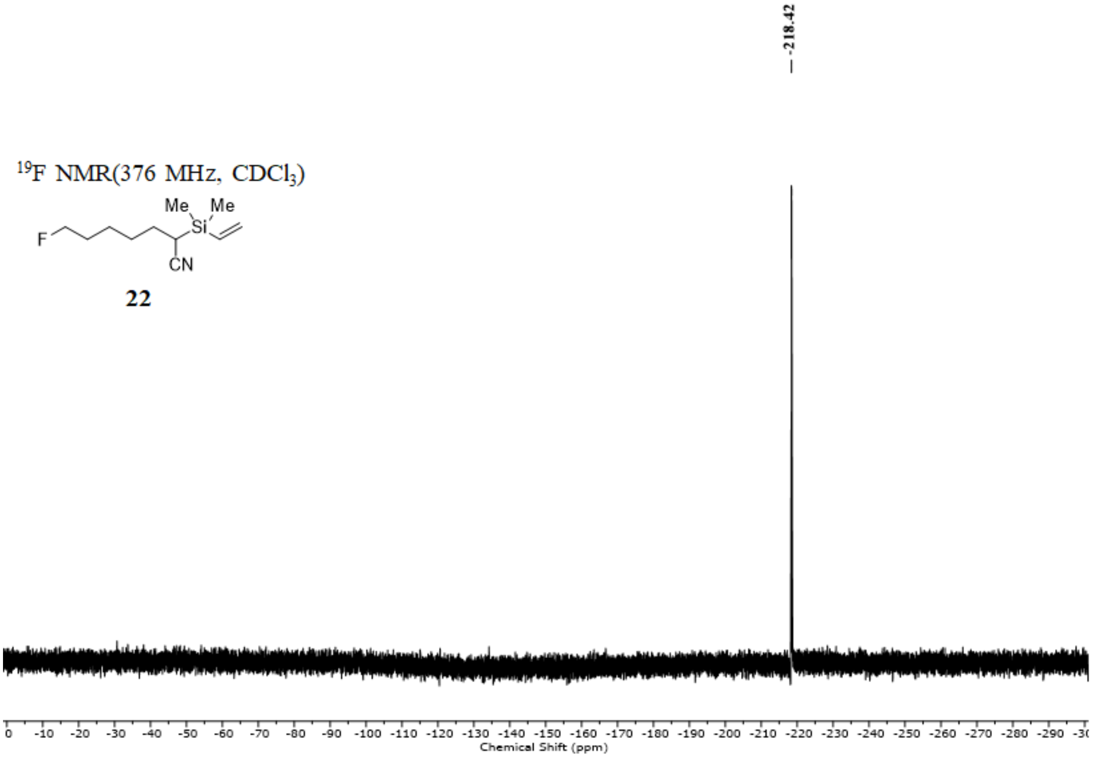

Supplementary Fig. 102. ^{19}F NMR of compound 22. The sample has been recorded in 376 MHz, $CDCl_3$ at 25 °C

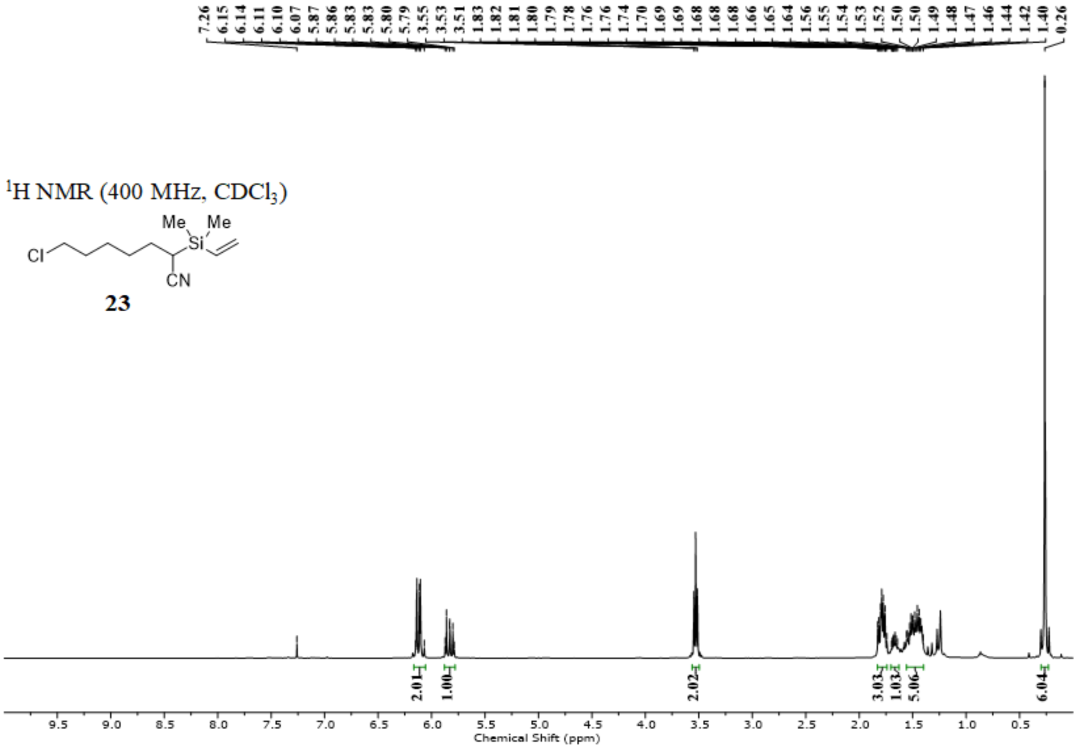

Supplementary Fig. 103. ^{1}H NMR of compound 23. The sample has been recorded in 400 MHz, $CDCl_3$ at 25 °C

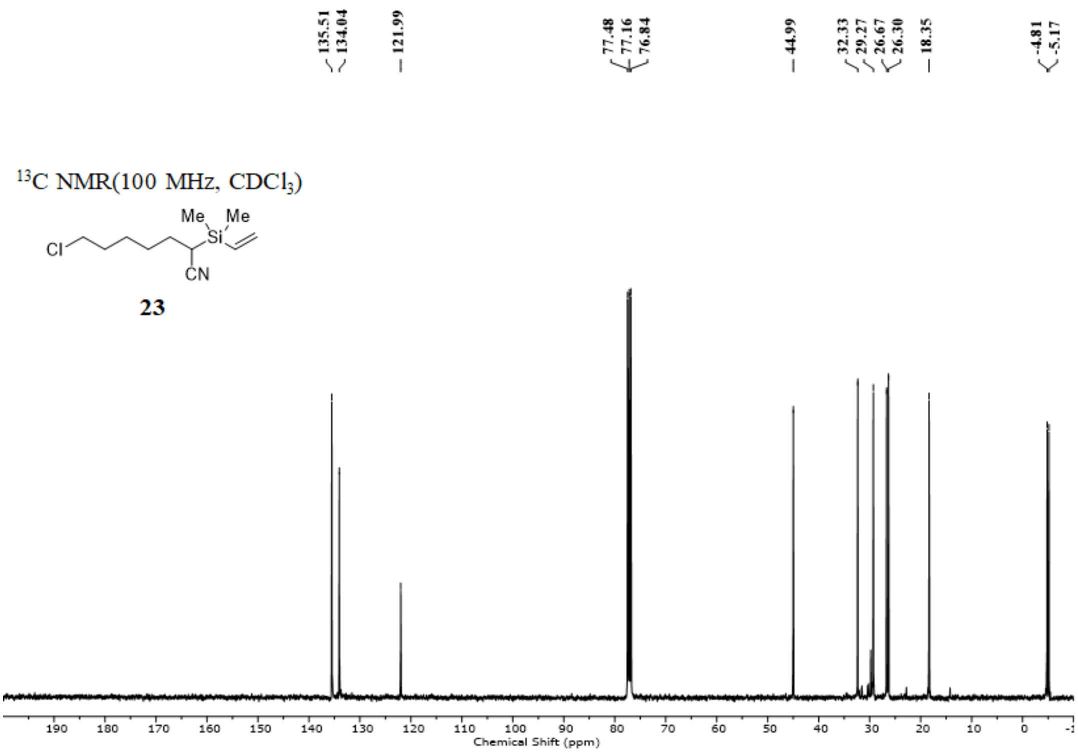

Supplementary Fig. 104. ^{13}C NMR of compound 23. The sample has been recorded in 100 MHz, $CDCl_3$ at 25 °C

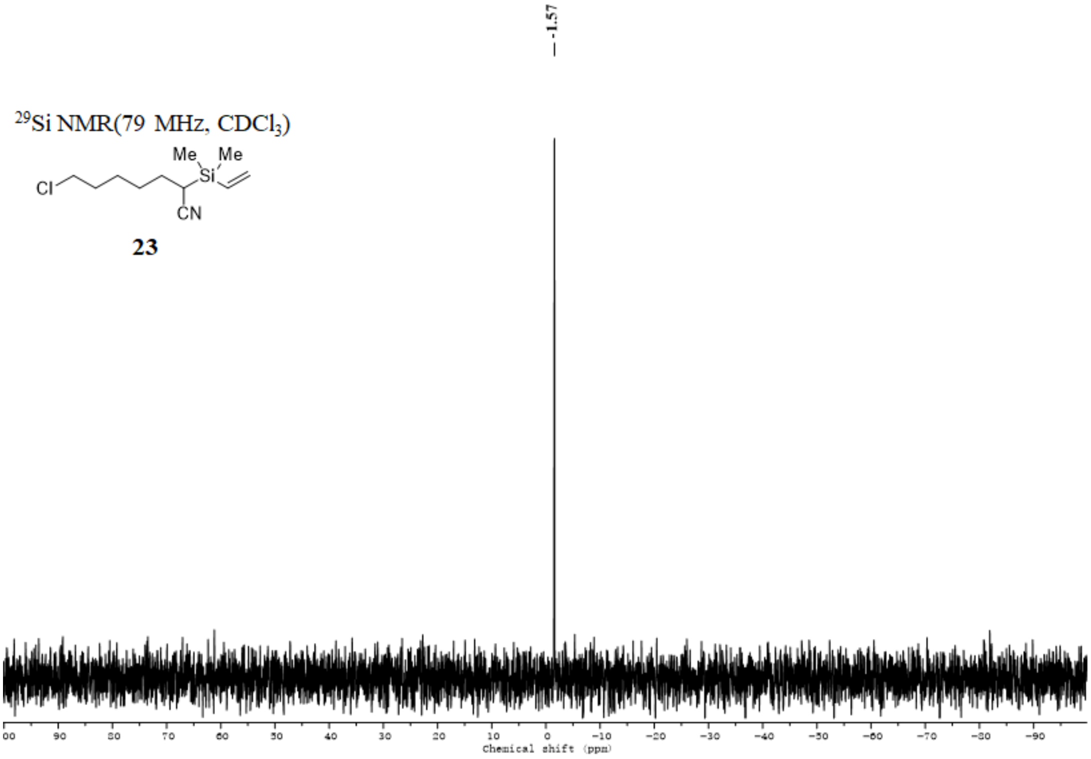

Supplementary Fig. 105. ^{29}Si NMR of compound 23. The sample has been recorded in 79 MHz, $CDCl_3$ at 25 °C

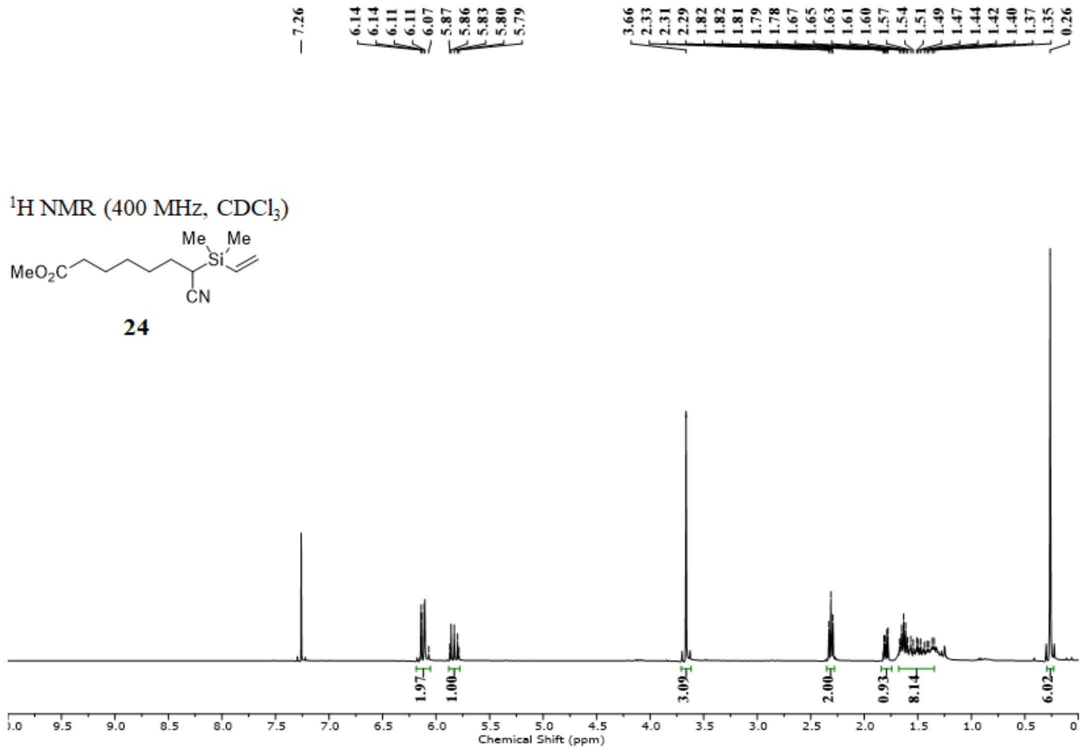

Supplementary Fig. 106. ^{1}H NMR of compound 24. The sample has been recorded in 400 MHz, CDCl3 at 25 °C

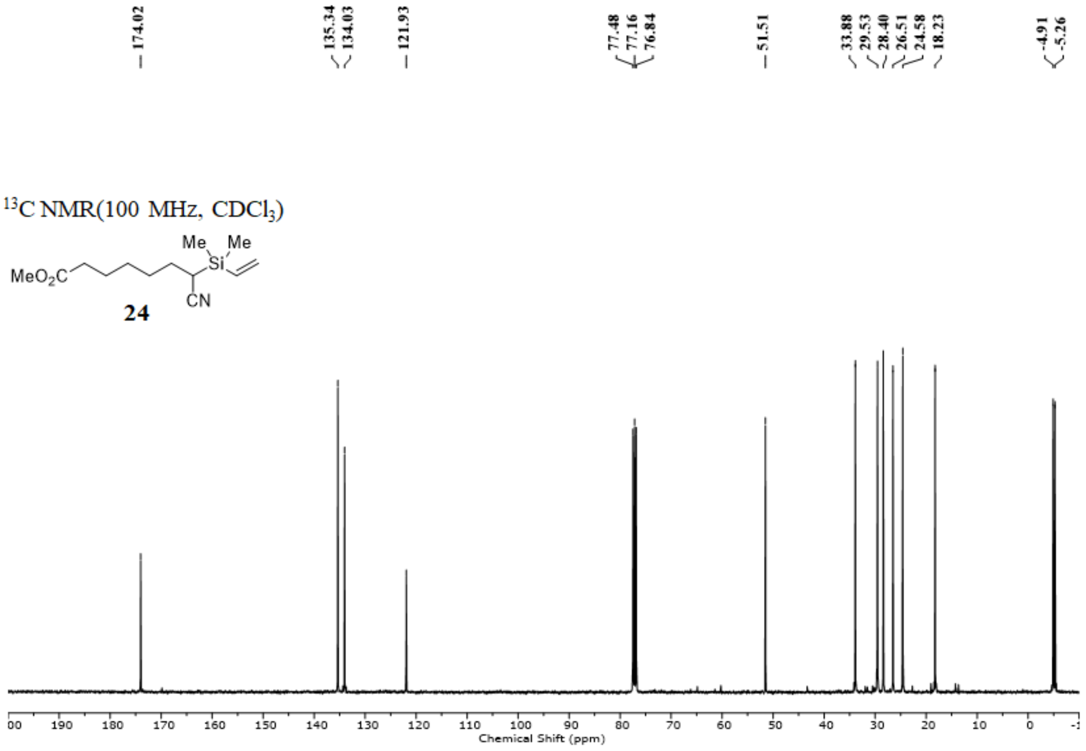

Supplementary Fig. 107. ^{13}C NMR of compound 24. The sample has been recorded in 100 MHz, CDCl3 at 25 °C

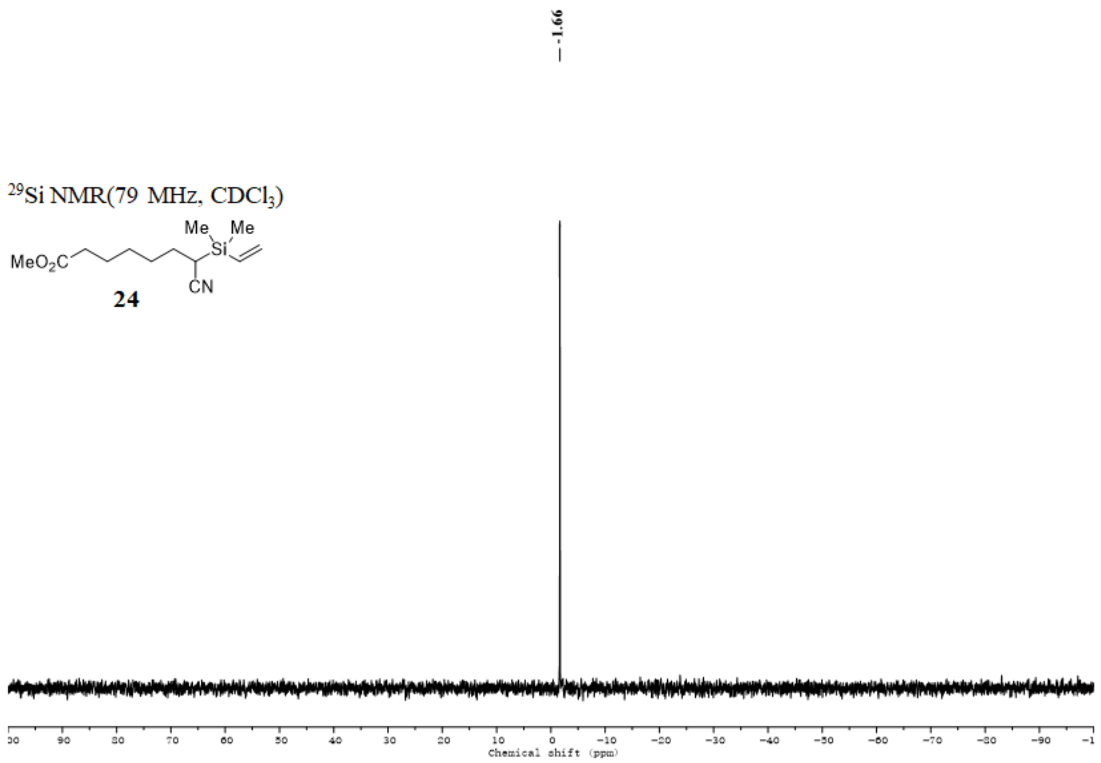

Supplementary Fig. 108. ^{29}Si NMR of compound 24. The sample has been recorded in 79 MHz, $CDCl_3$ at 25 °C

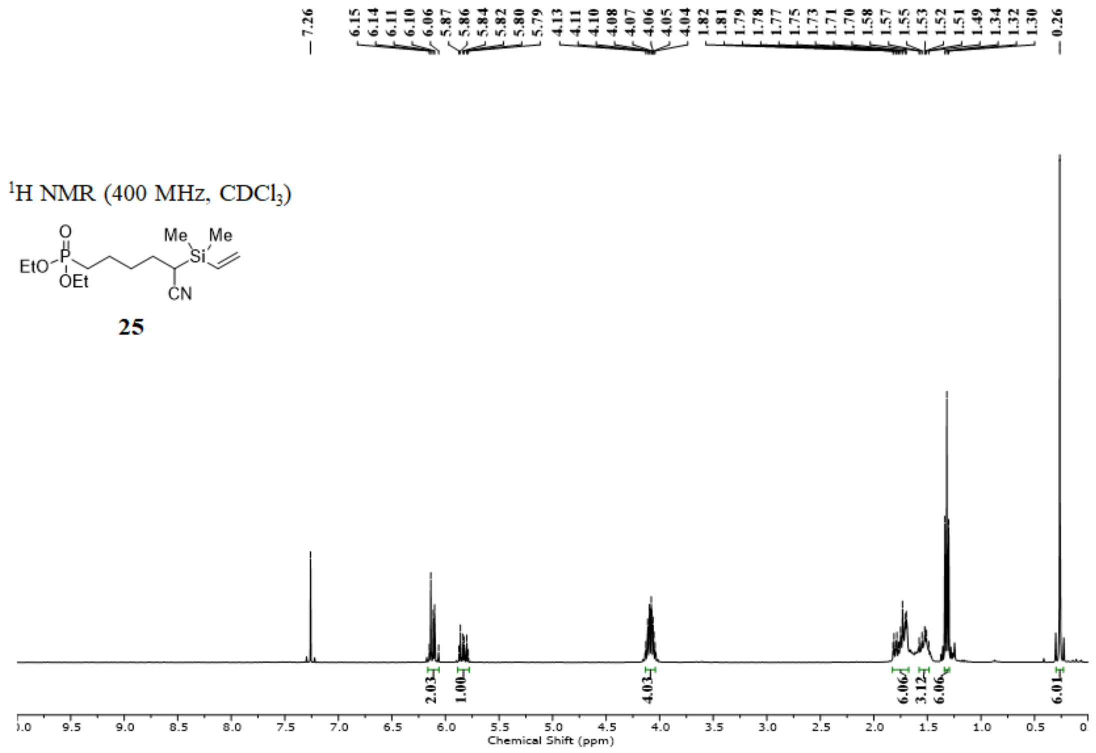

Supplementary Fig. 109. ^{1}H NMR of compound 25. The sample has been recorded in 400 MHz, $CDCl_3$ at 25 °C

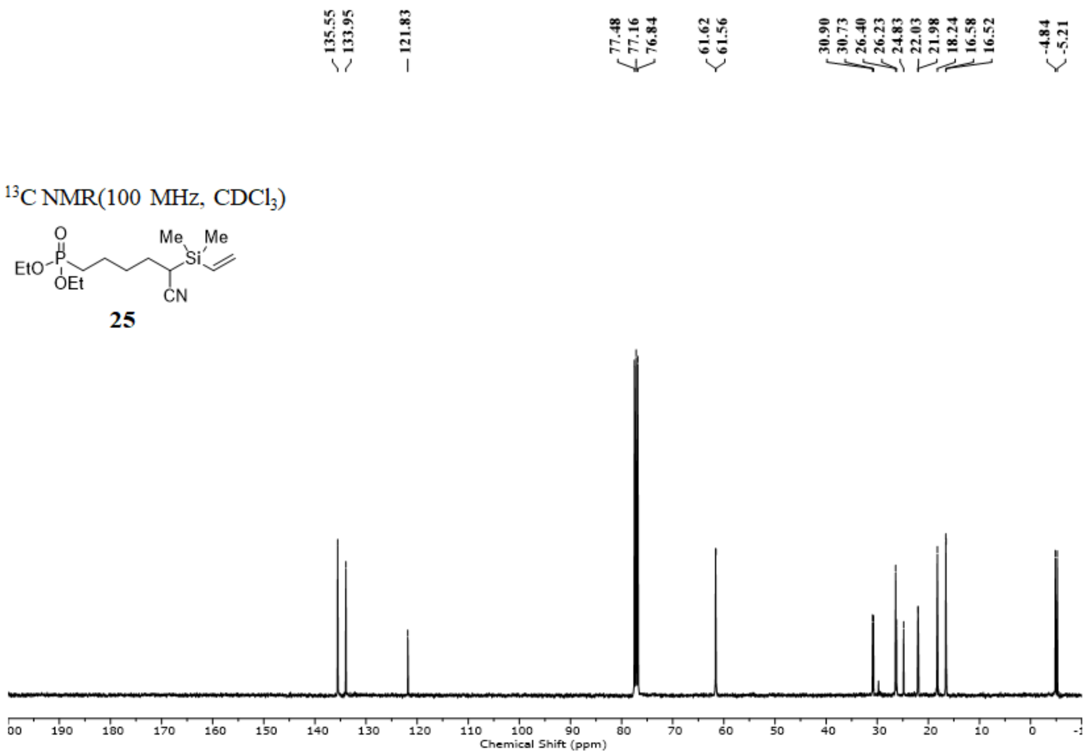

Supplementary Fig. 110. ^{13}C NMR of compound 25. The sample has been recorded in 100 MHz, $CDCl_3$ at 25 °C

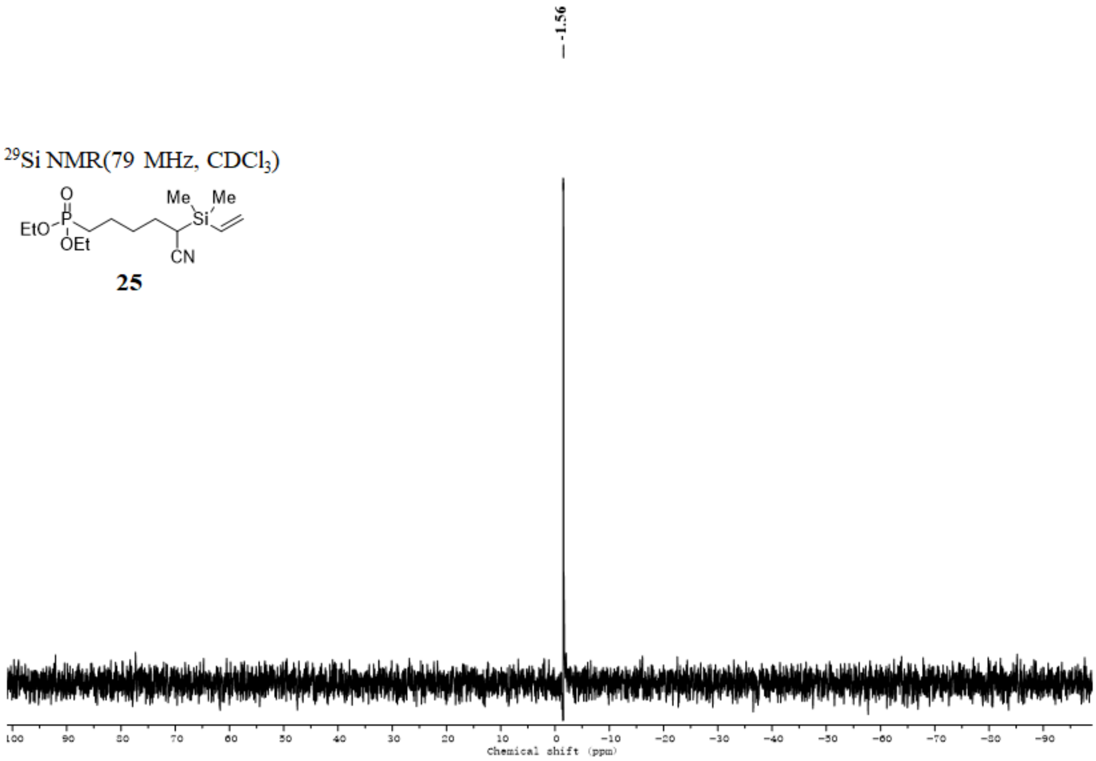

Supplementary Fig. 111. ^{29}Si NMR of compound 25. The sample has been recorded in 79 MHz, $CDCl_3$ at 25 °C

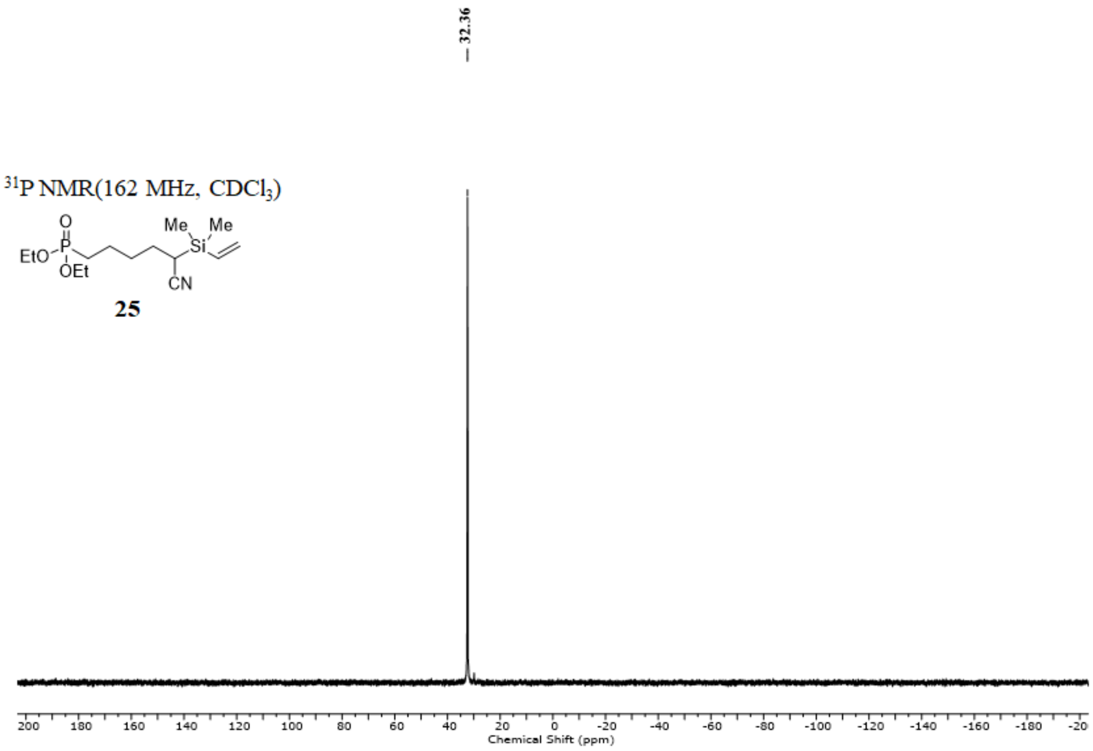

Supplementary Fig. 112. ^{31}P NMR of compound 25. The sample has been recorded in 162 MHz, $CDCl_3$ at 25 °C

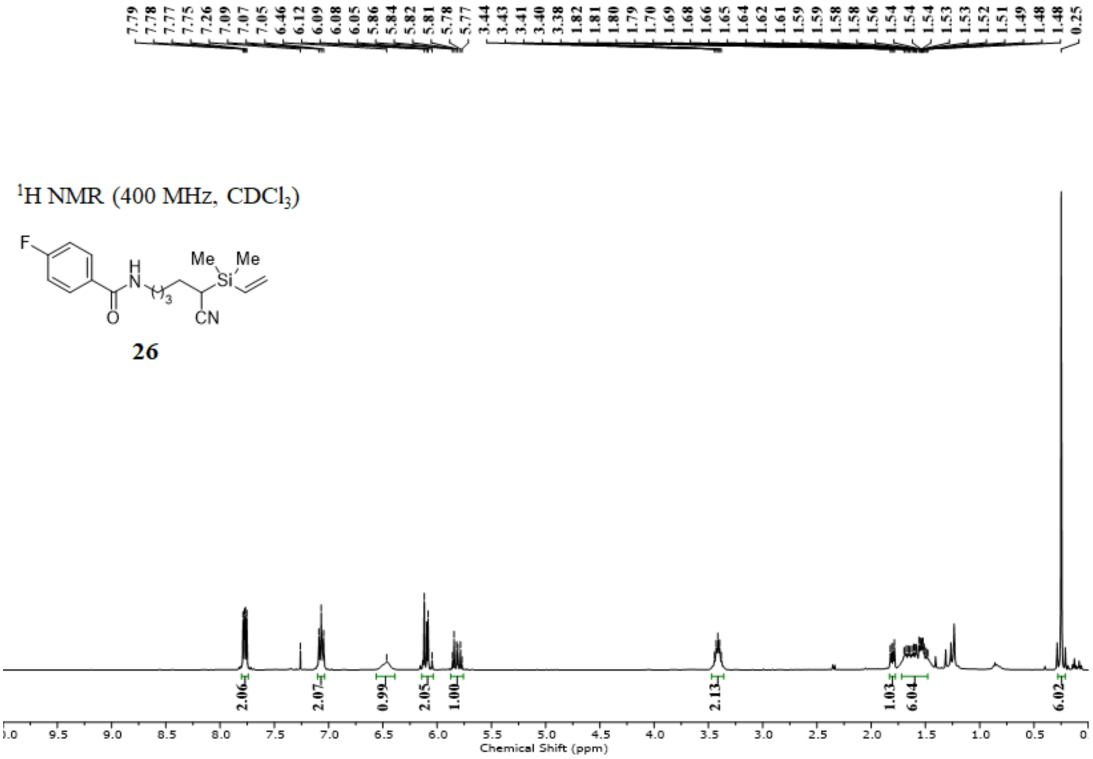

Supplementary Fig. 113. ^{1}H NMR of compound 26. The sample has been recorded in 400 MHz, $CDCl_3$ at 25 °C

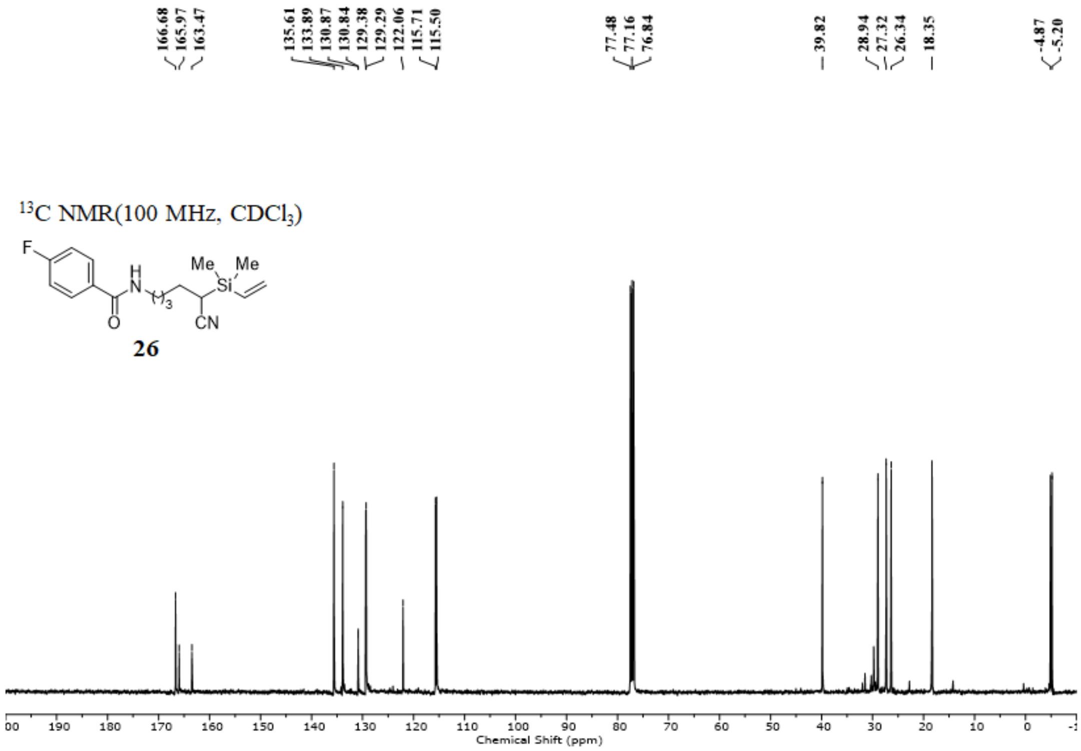

Supplementary Fig. 114. ^{13}C NMR of compound 26. The sample has been recorded in 100 MHz, $CDCl_3$ at 25 °C

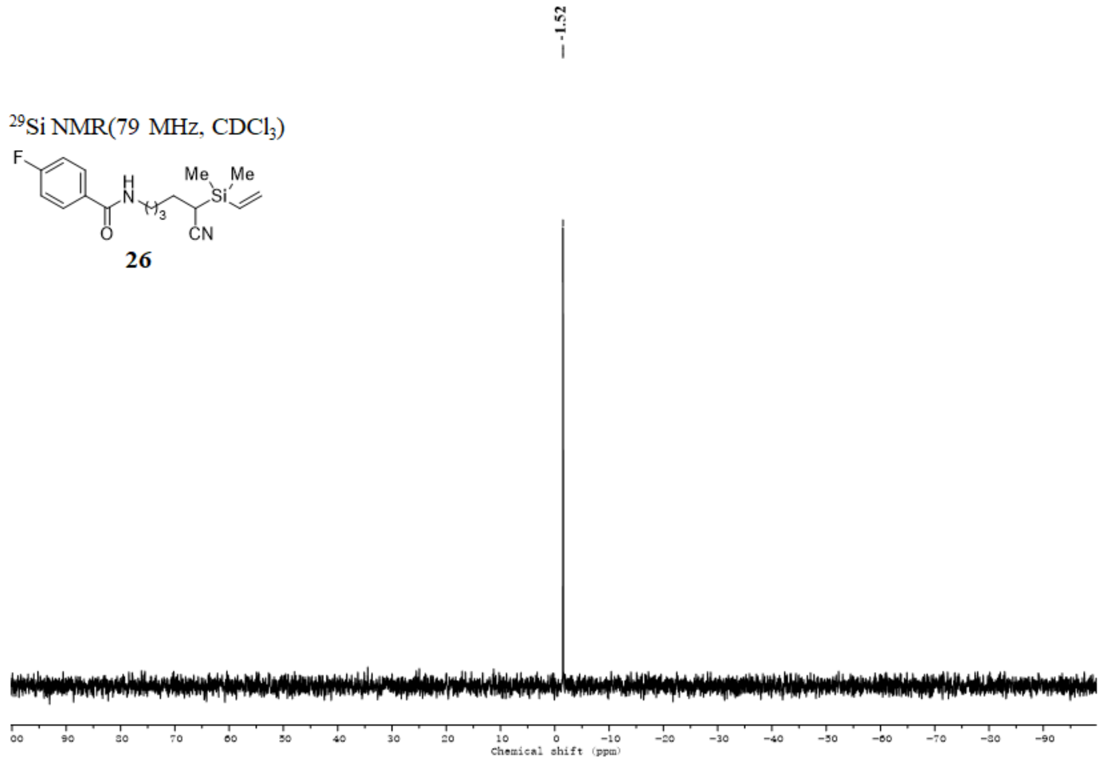

Supplementary Fig. 115. ^{29}Si NMR of compound 26. The sample has been recorded in 79 MHz, $CDCl_3$ at 25 °C

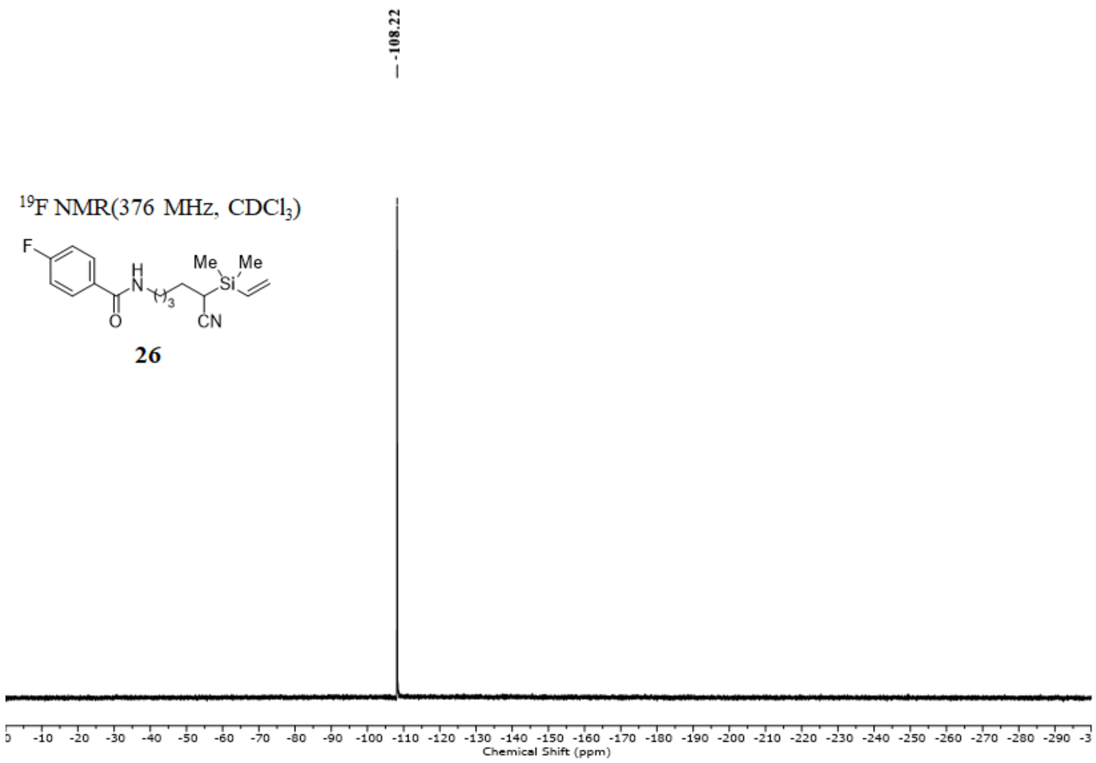

Supplementary Fig. 116. ^{19}F NMR of compound 26. The sample has been recorded in 376 MHz, $CDCl_3$ at 25 °C

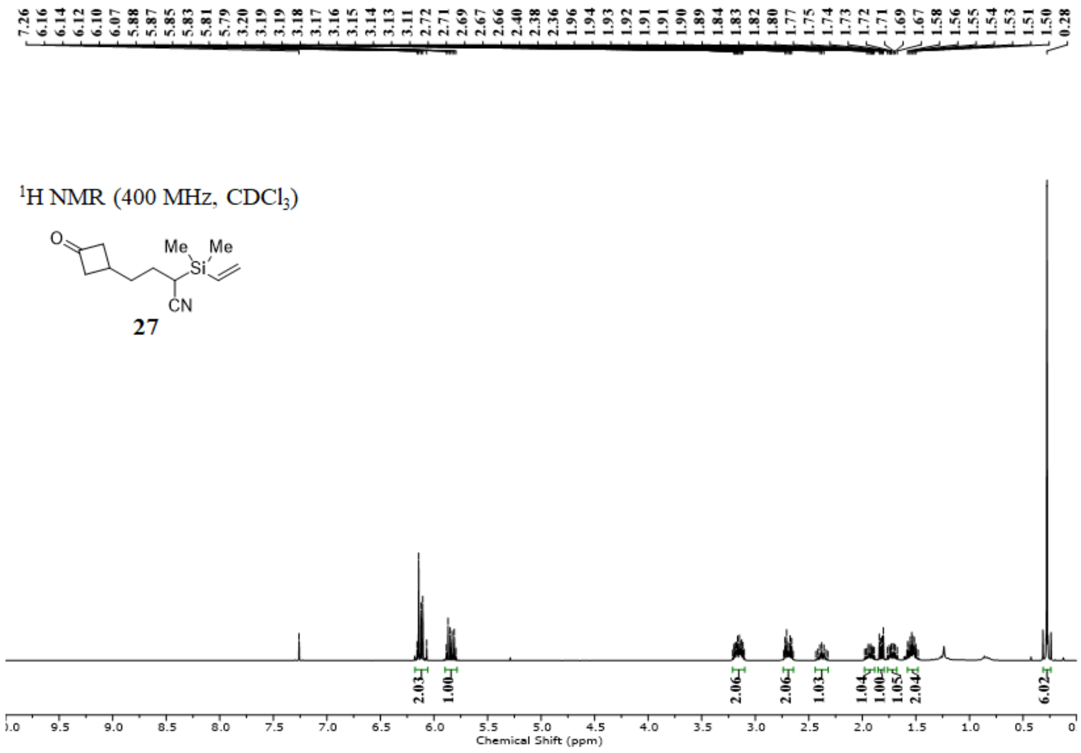

Supplementary Fig. 117. ^{1}H NMR of compound 27. The sample has been recorded in 400 MHz, $CDCl_3$ at 25 °C

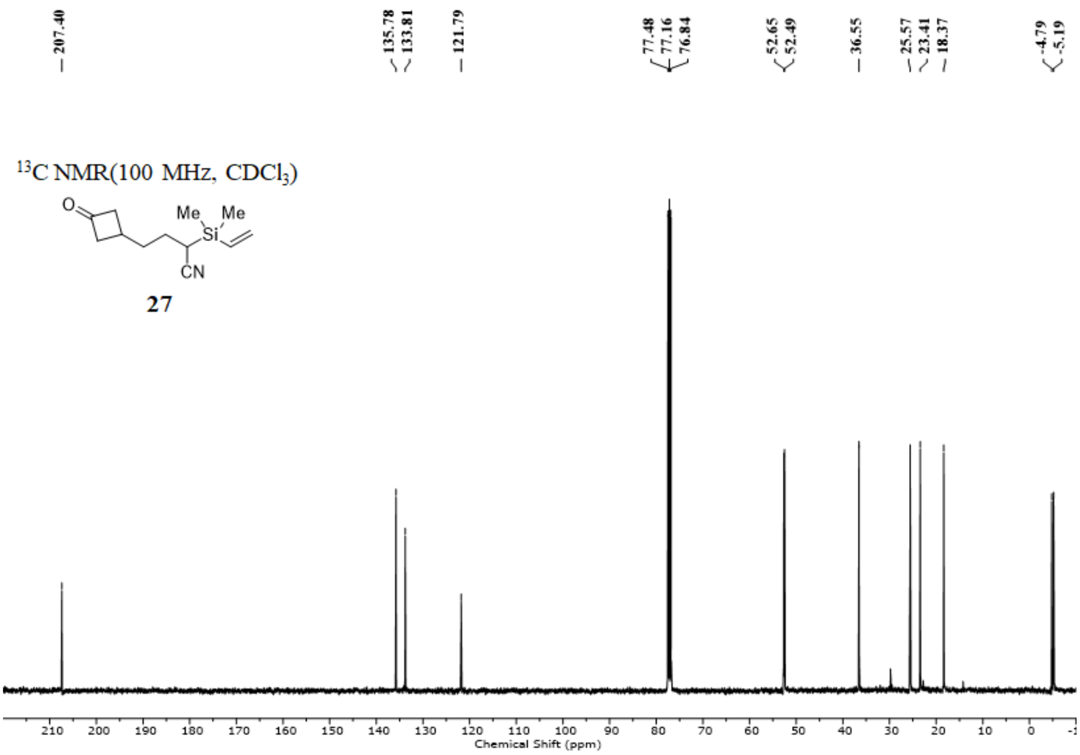

Supplementary Fig. 118. ^{13}C NMR of compound 27. The sample has been recorded in 100 MHz, $CDCl_3$ at 25 °C

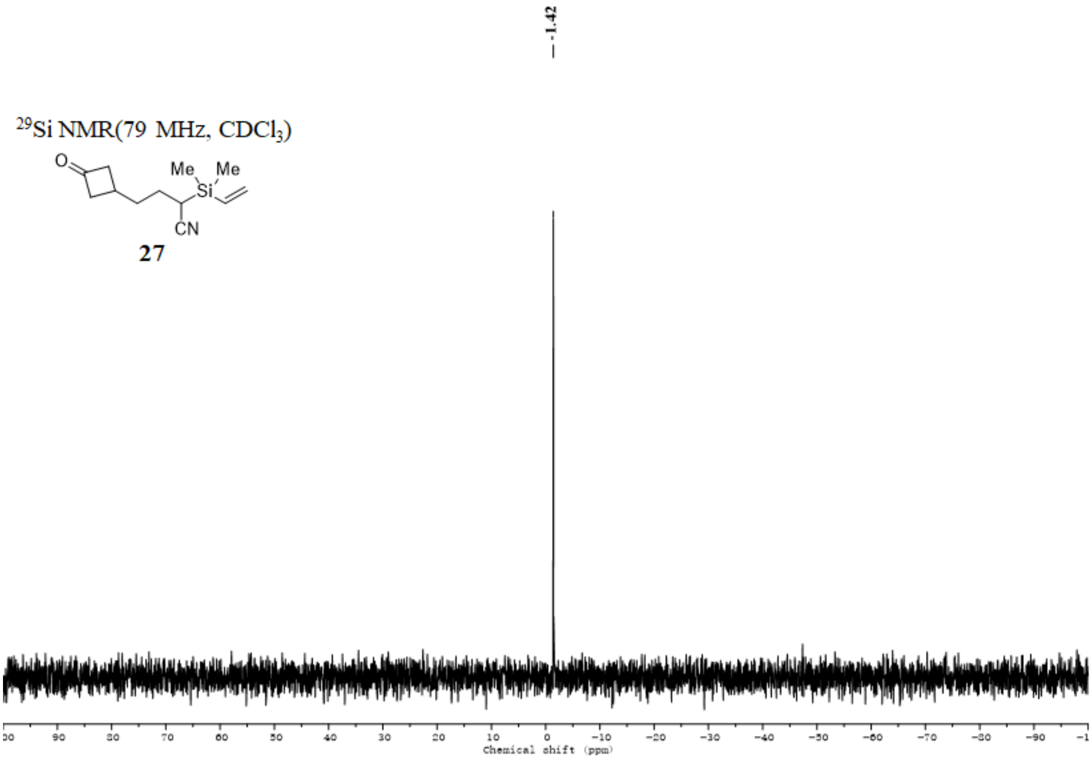

Supplementary Fig. 119. ^{29}Si NMR of compound 27. The sample has been recorded in 79 MHz, $CDCl_3$ at 25 °C

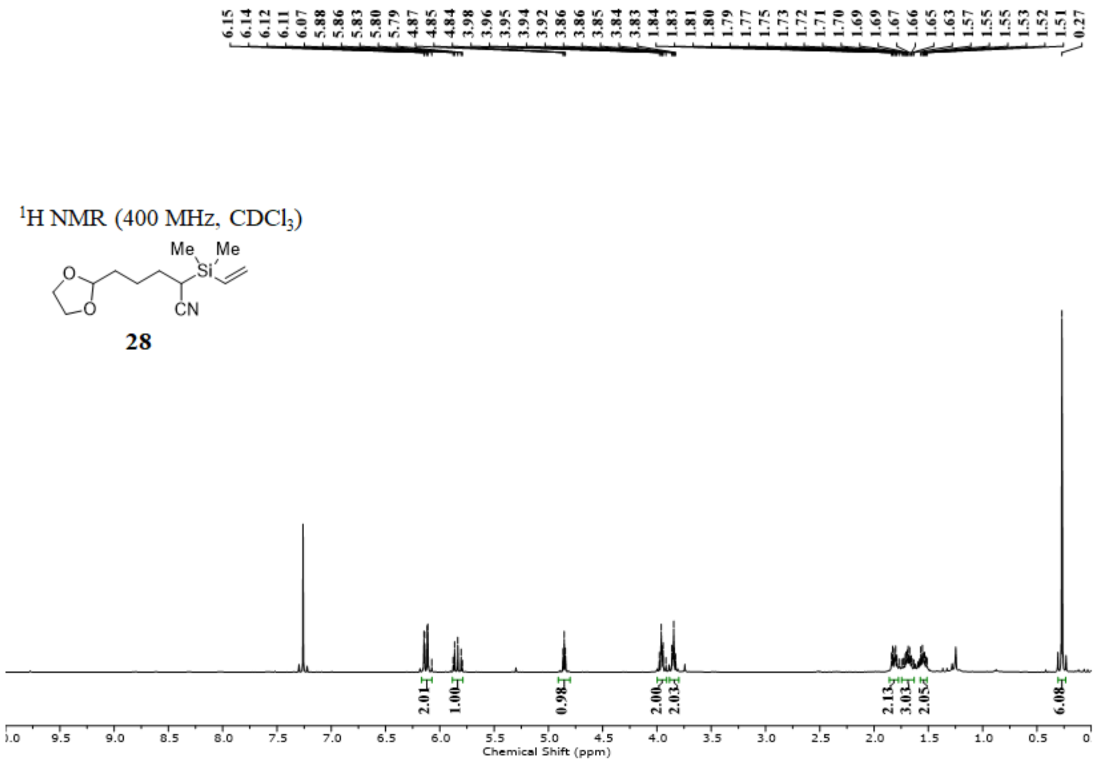

Supplementary Fig. 120. ^{1}H NMR of compound 28. The sample has been recorded in 400 MHz, $CDCl_3$ at 25 °C

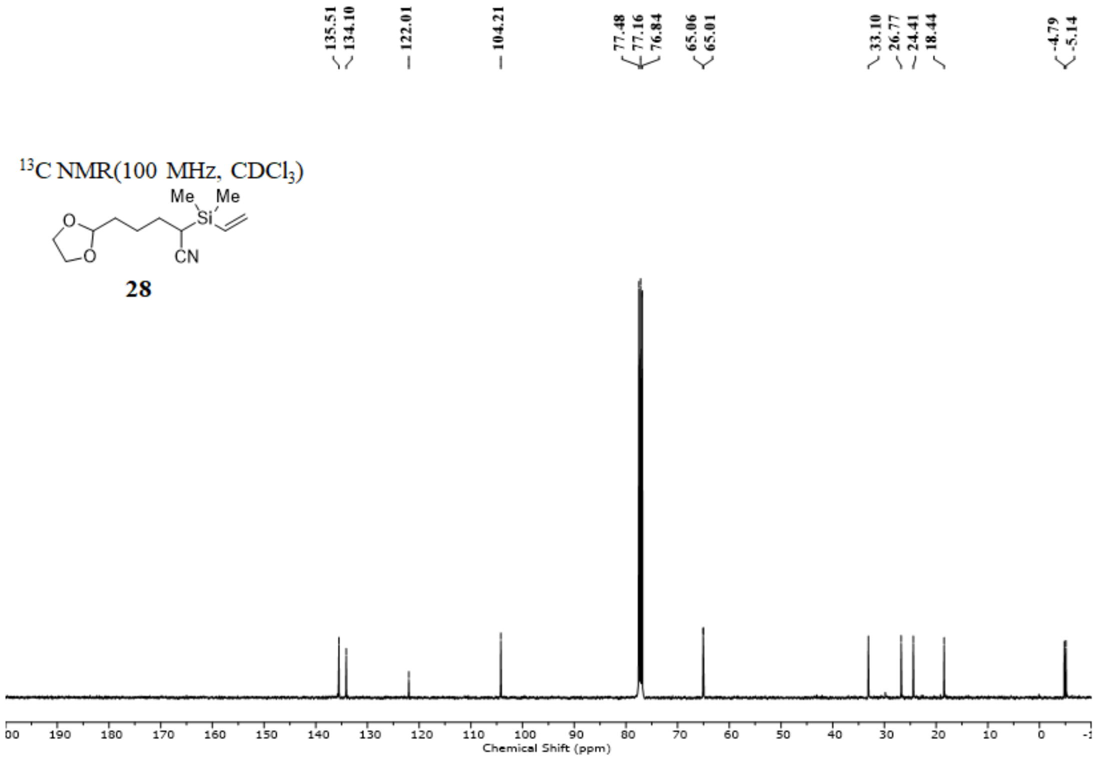

Supplementary Fig. 121. ^{13}C NMR of compound 28. The sample has been recorded in 100 MHz, $CDCl_3$ at 25 °C

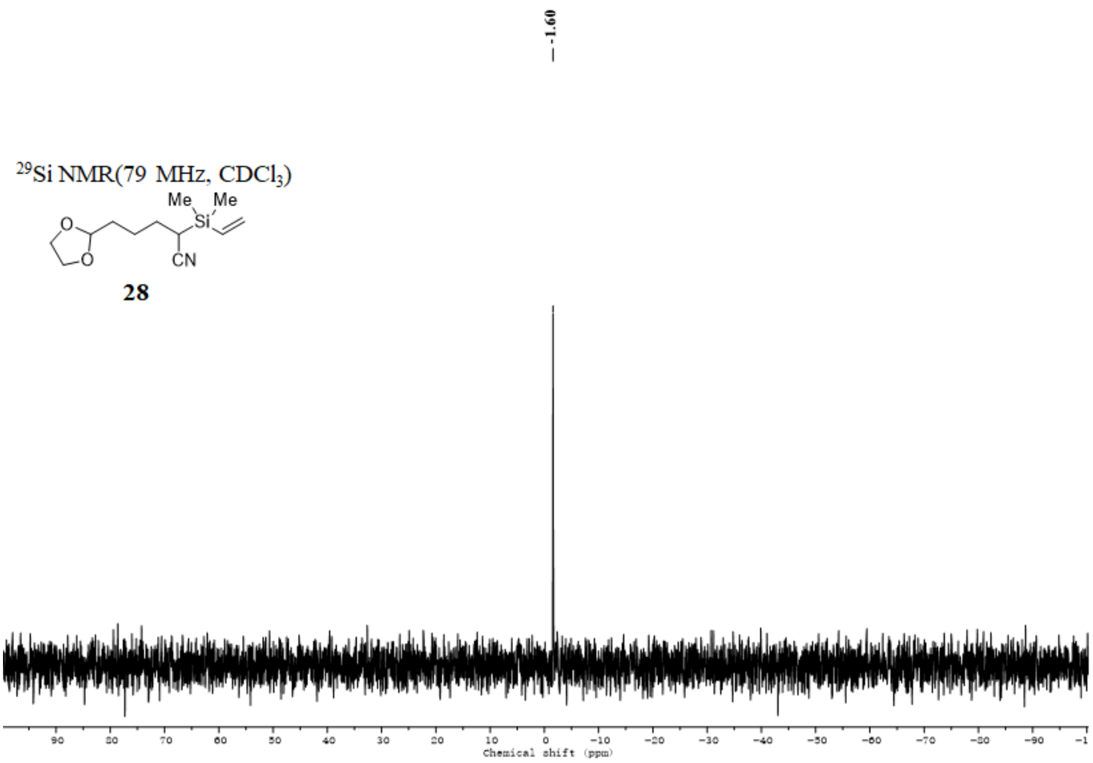

Supplementary Fig. 122. ^{29}Si NMR of compound 28. The sample has been recorded in 79 MHz, $CDCl_3$ at 25 °C

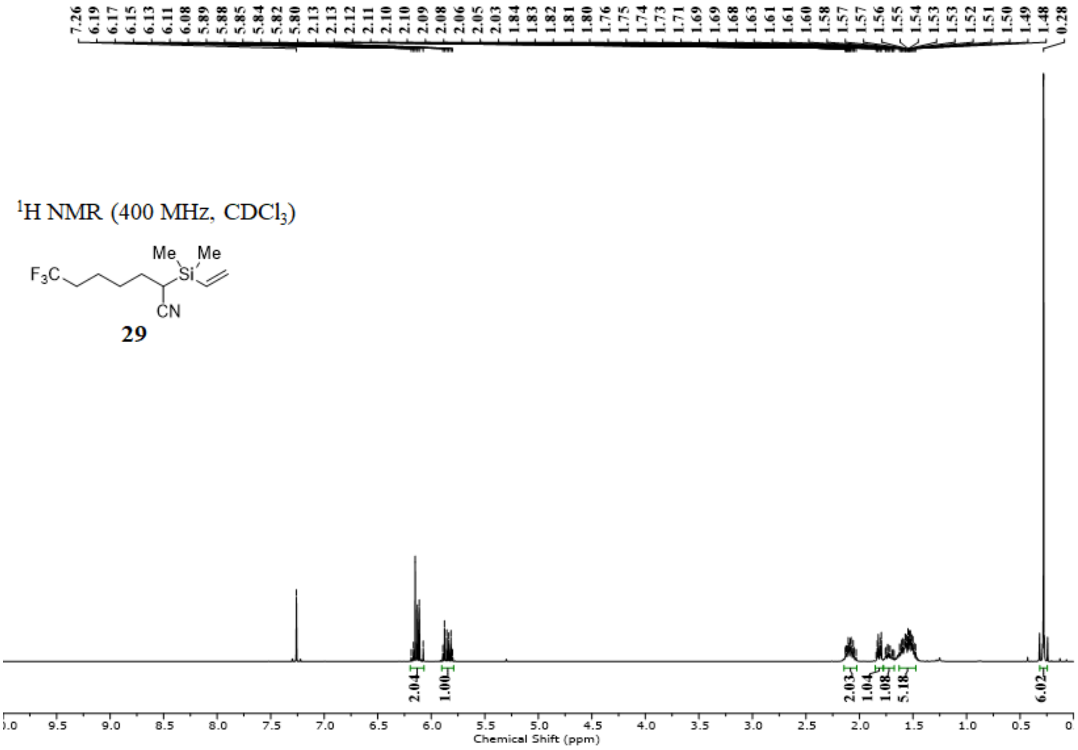

Supplementary Fig. 123. ^{1}H NMR of compound 29. The sample has been recorded in 400 MHz, $CDCl_3$ at 25 °C

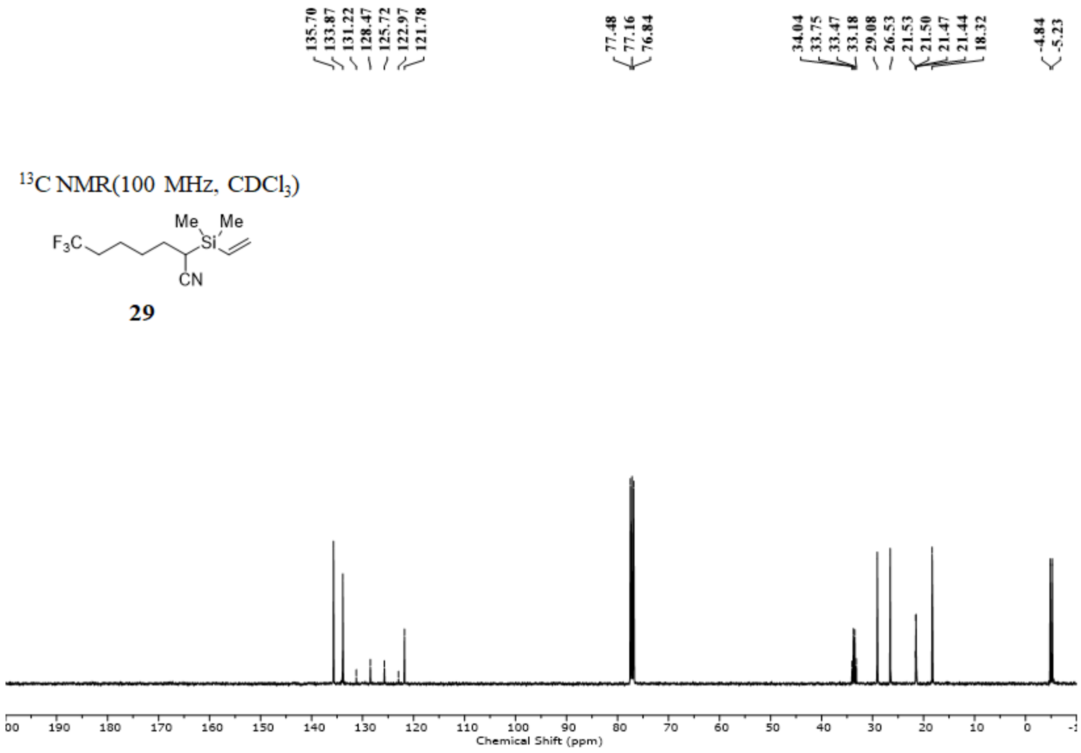

Supplementary Fig. 124. ^{13}C NMR of compound 29. The sample has been recorded in 100 MHz, $CDCl_3$ at 25 °C

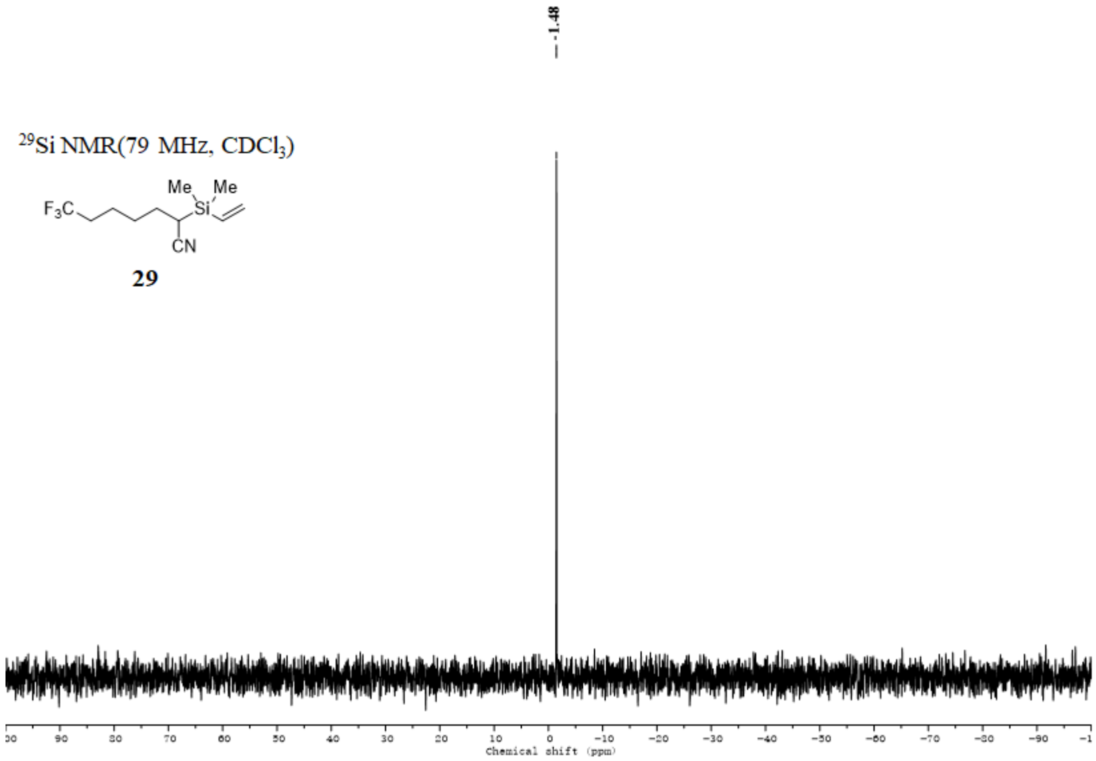

Supplementary Fig. 125. ^{29}Si NMR of compound 29. The sample has been recorded in 79 MHz, $CDCl_3$ at 25 °C

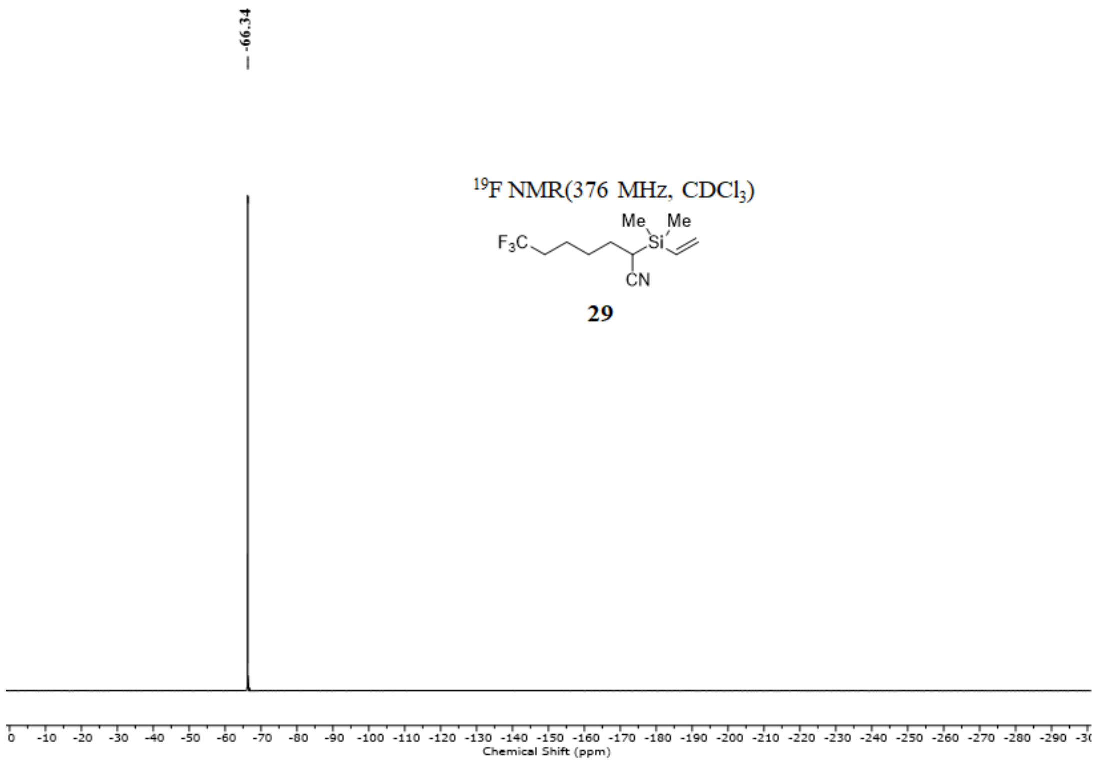

Supplementary Fig. 126. ^{19}F NMR of compound 29. The sample has been recorded in 376 MHz, $CDCl_3$ at 25 °C

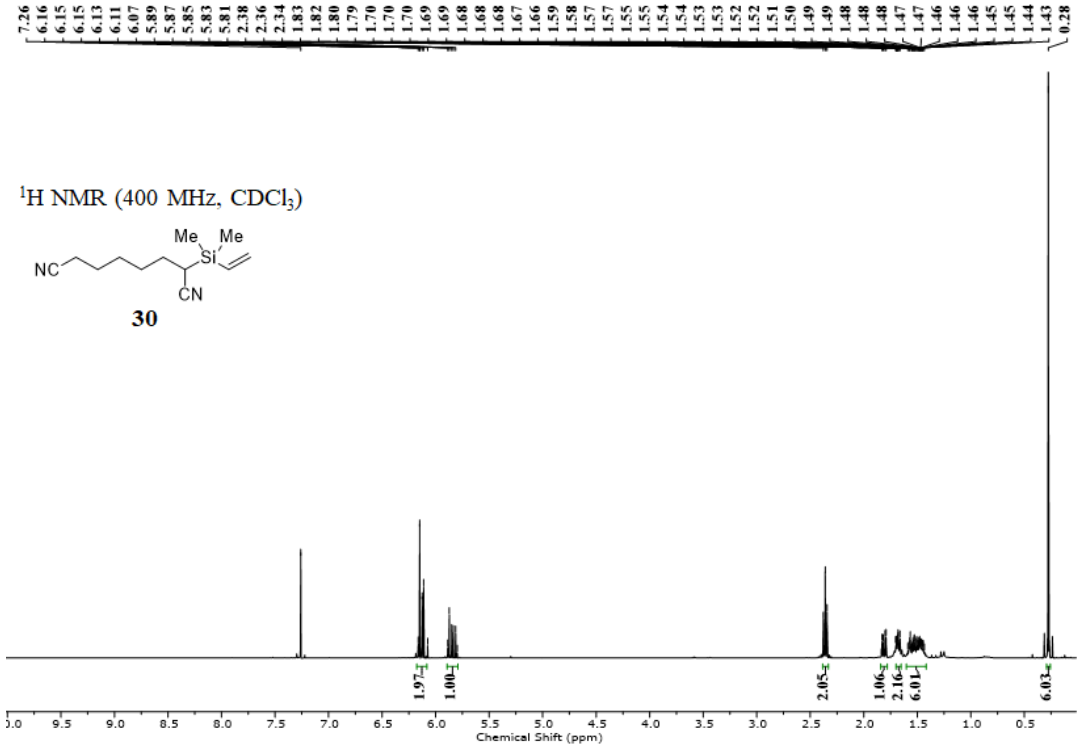

Supplementary Fig. 127. ^{1}H NMR of compound 30. The sample has been recorded in 400 MHz, $CDCl_3$ at 25 °C

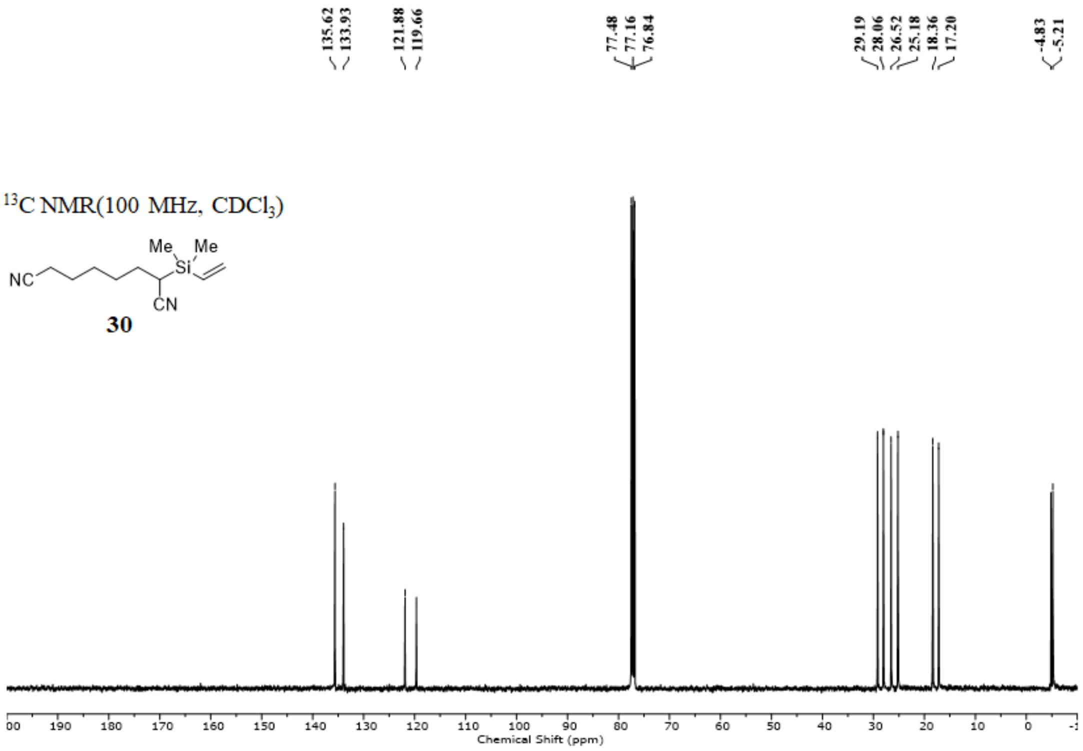

Supplementary Fig. 128. ^{13}C NMR of compound 30. The sample has been recorded in 100 MHz, $CDCl_3$ at 25 °C

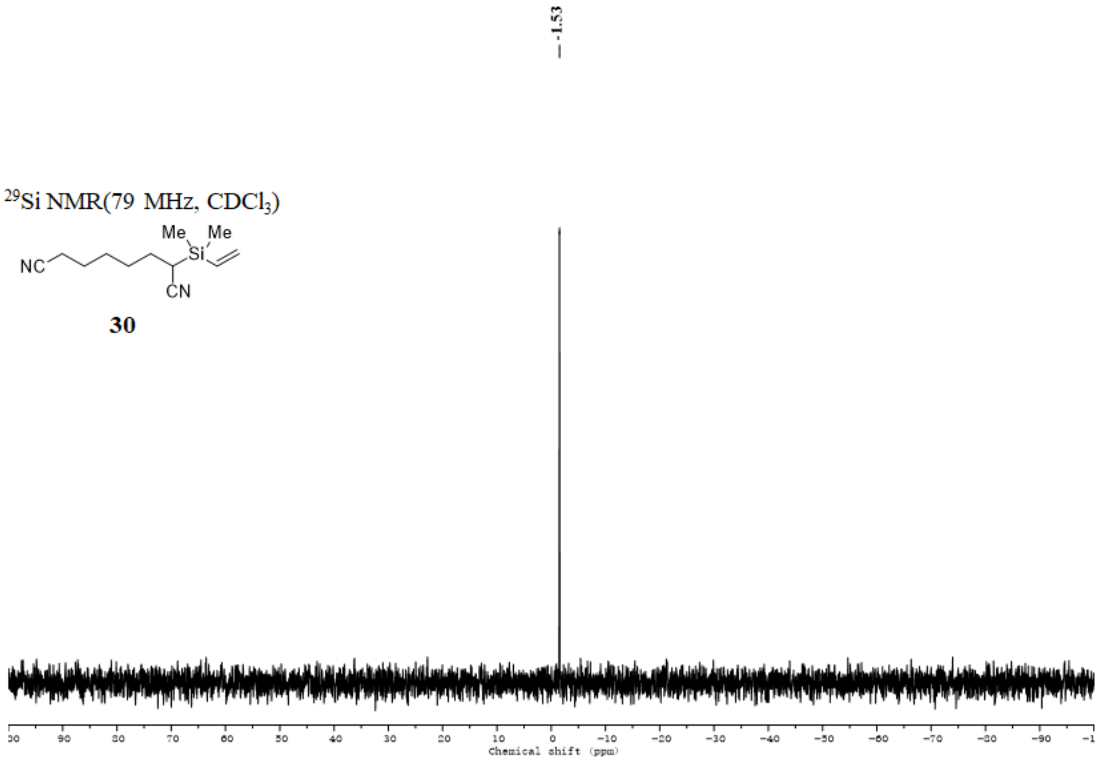

Supplementary Fig. 129. ^{29}Si NMR of compound 30. The sample has been recorded in 79 MHz, $CDCl_3$ at 25 °C

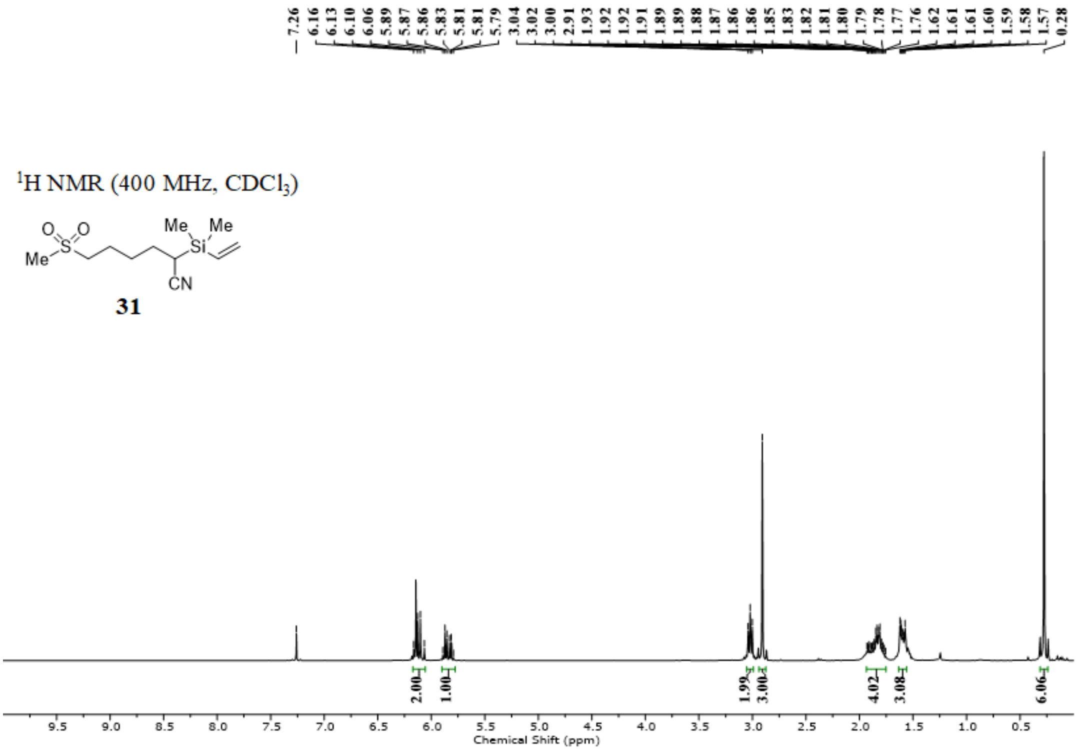

Supplementary Fig. 130. ^{1}H NMR of compound 31. The sample has been recorded in 400 MHz, $CDCl_3$ at 25 °C

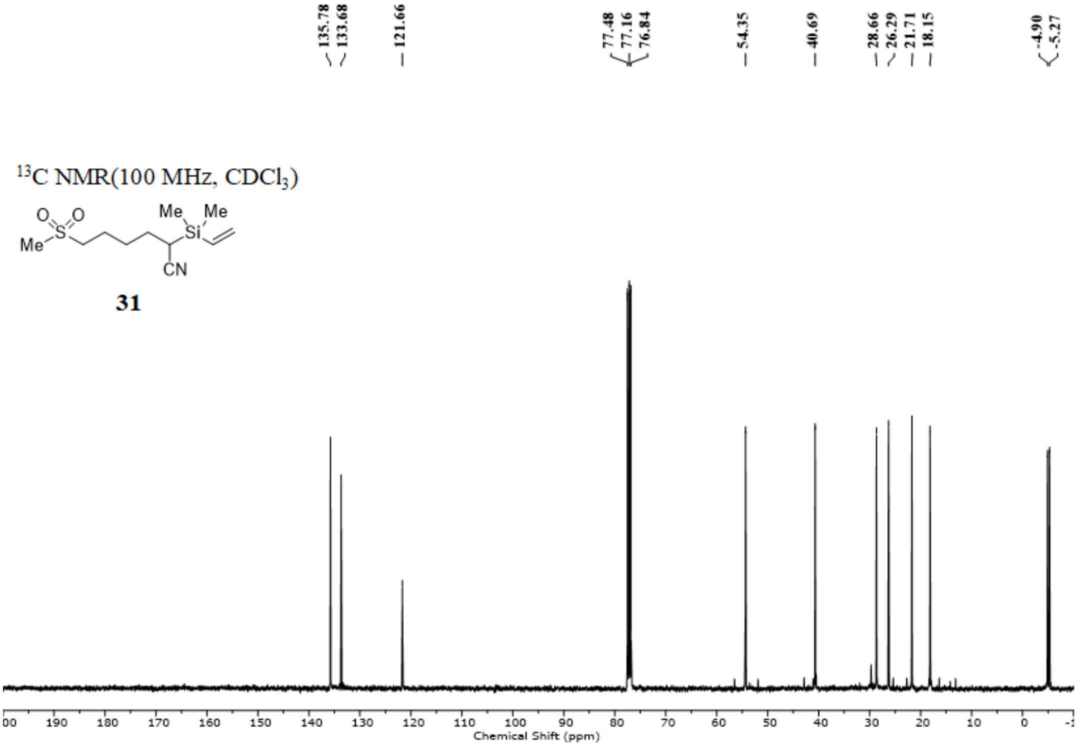

Supplementary Fig. 131. ^{13}C NMR of compound 31. The sample has been recorded in 100 MHz, $CDCl_3$ at 25 °C

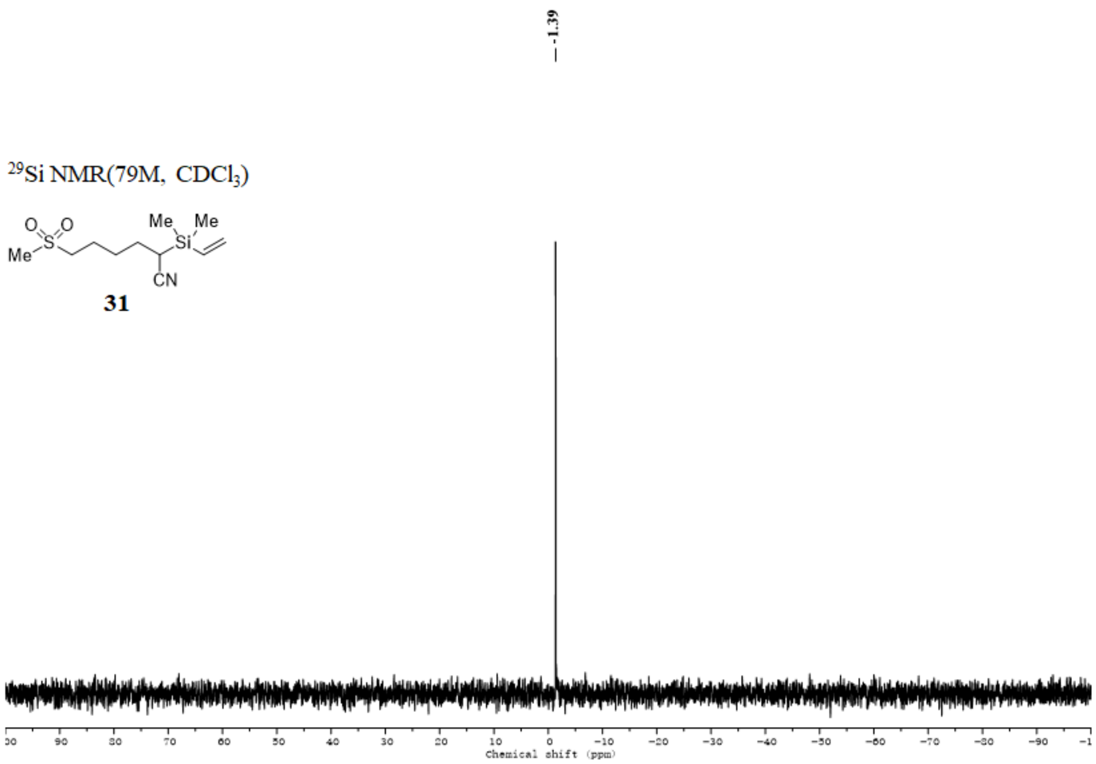

Supplementary Fig. 132. ^{29}Si NMR of compound 31. The sample has been recorded in 79 MHz, $CDCl_3$ at 25 °C

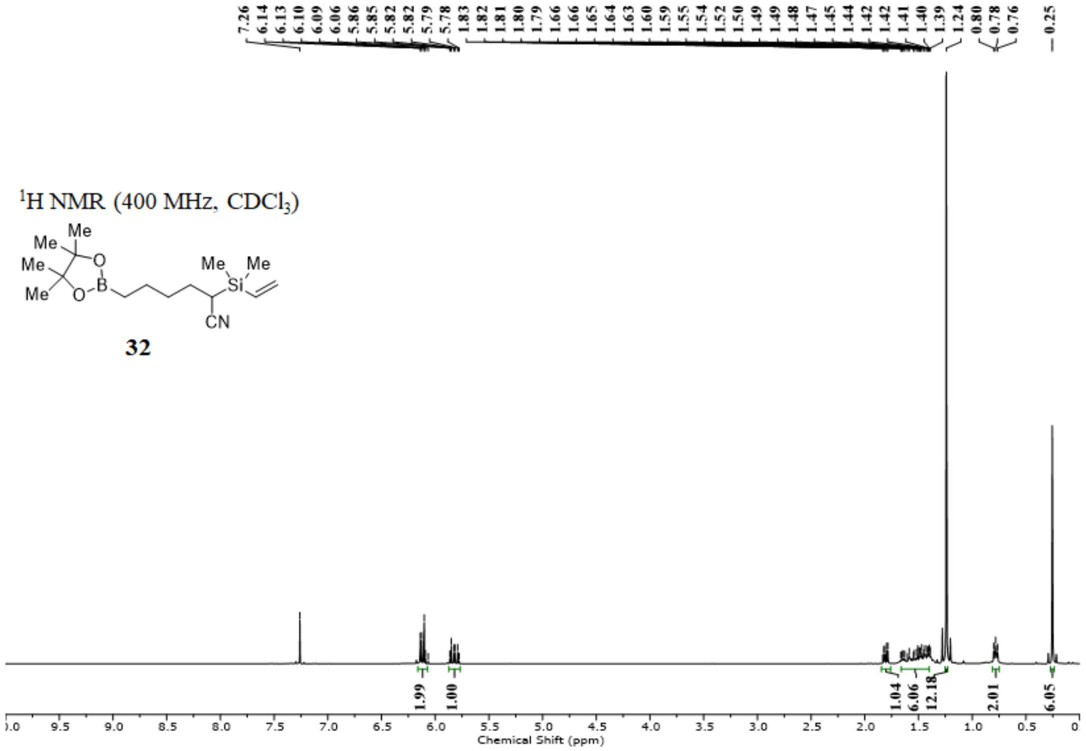

Supplementary Fig. 133. ^{1}H NMR of compound 32. The sample has been recorded in 400 MHz, $CDCl_3$ at 25 °C

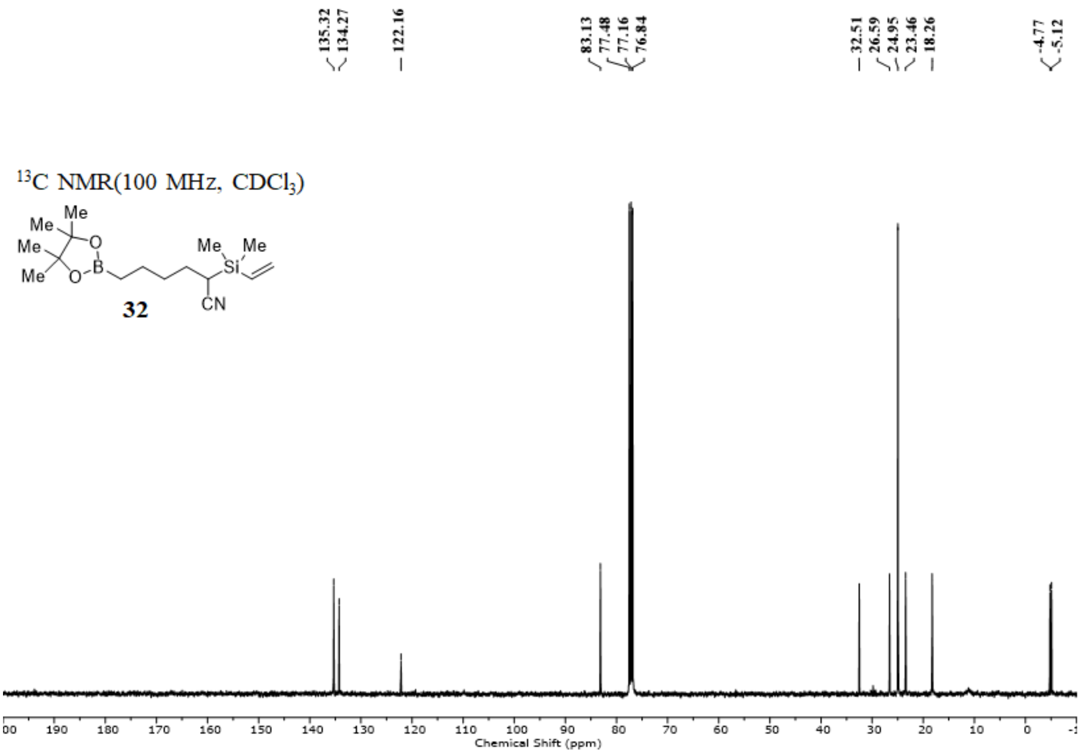

Supplementary Fig. 134. ^{13}C NMR of compound 32. The sample has been recorded in 100 MHz, $CDCl_3$ at 25 °C

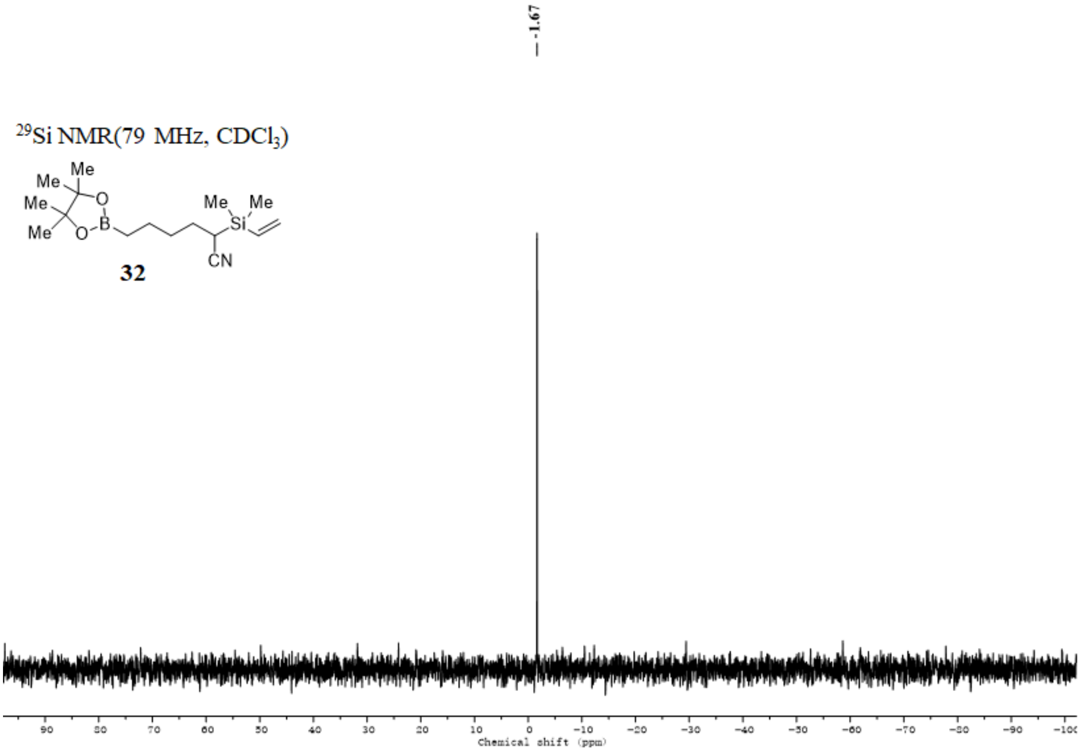

Supplementary Fig. 135. ^{29}Si NMR of compound 32. The sample has been recorded in 79 MHz, $CDCl_3$ at 25 °C

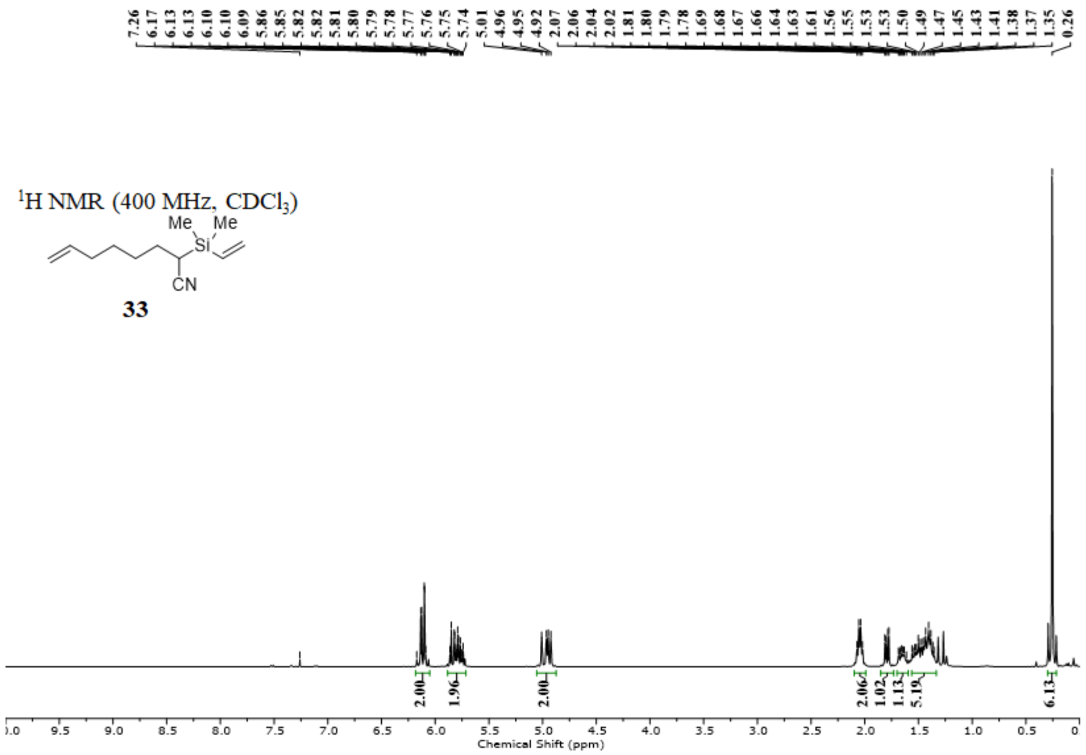

Supplementary Fig. 136. ^{1}H NMR of compound 33. The sample has been recorded in 400 MHz, $CDCl_3$ at 25 °C

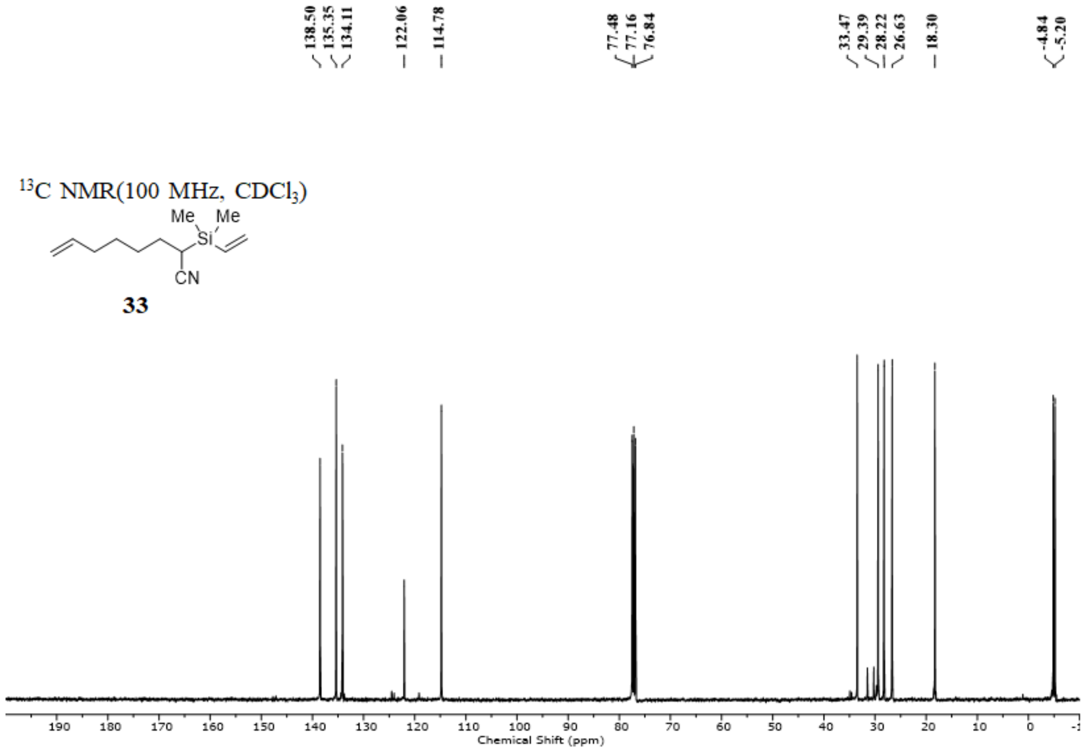

Supplementary Fig. 137. ^{13}C NMR of compound 33. The sample has been recorded in 100 MHz, $CDCl_3$ at 25 °C

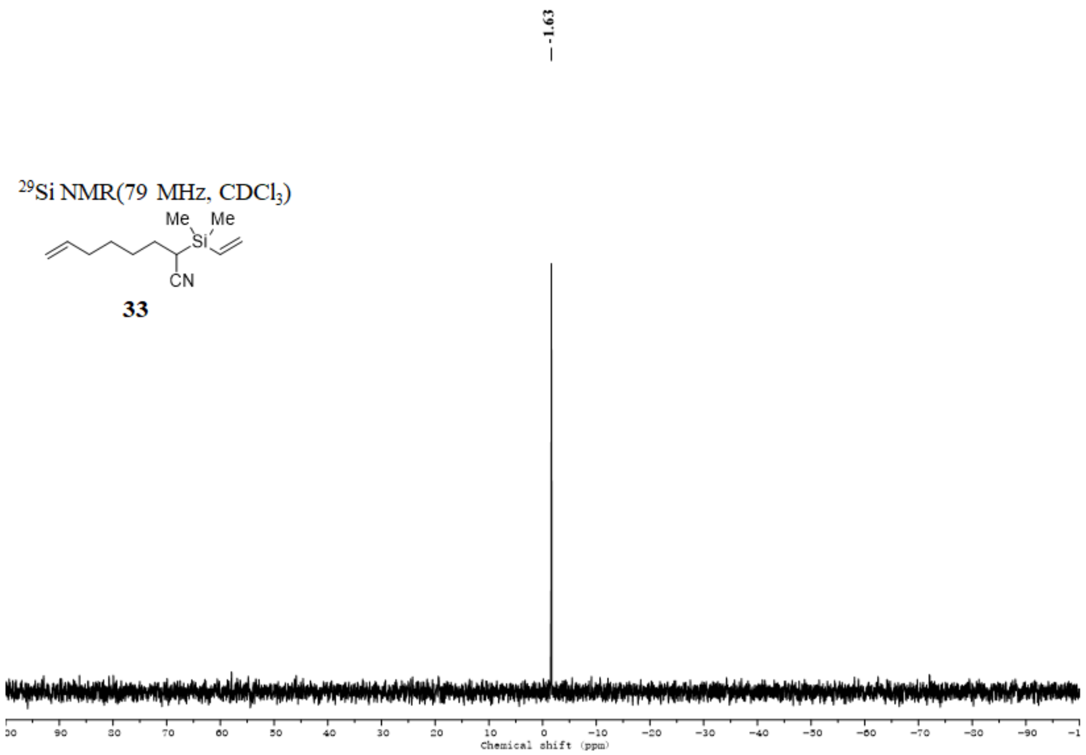

Supplementary Fig. 138. ^{29}Si NMR of compound 33. The sample has been recorded in 79 MHz, $CDCl_3$ at 25 °C

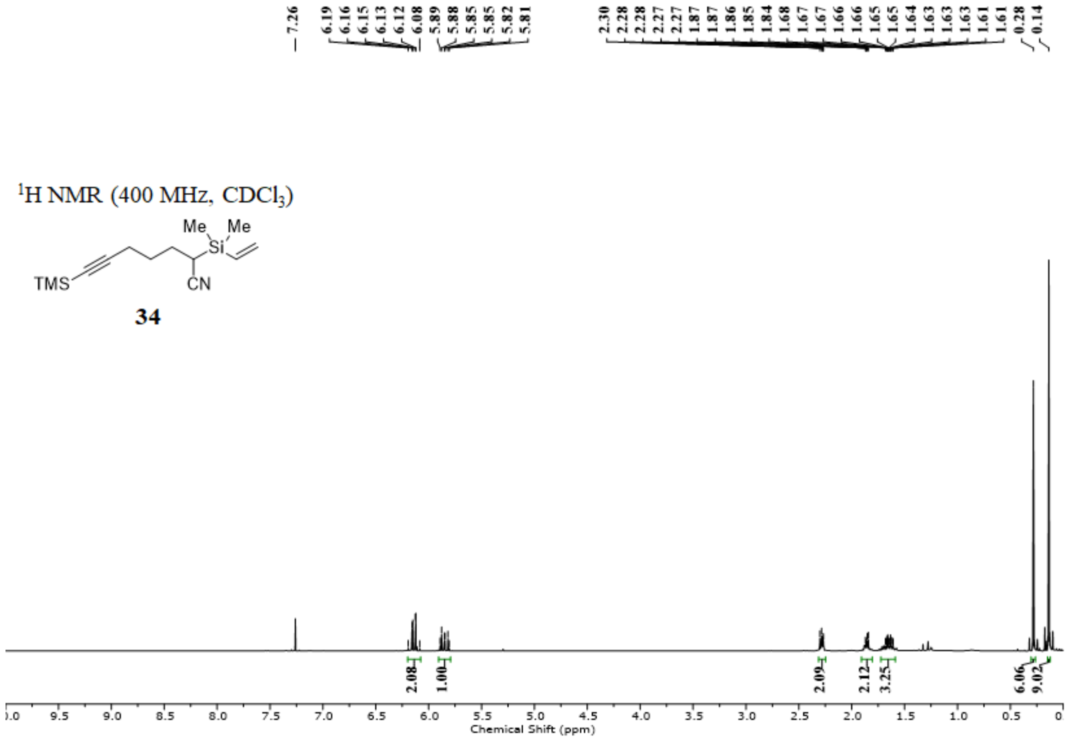

Supplementary Fig. 139. ^{1}H NMR of compound 34. The sample has been recorded in 400 MHz, $CDCl_3$ at 25 °C

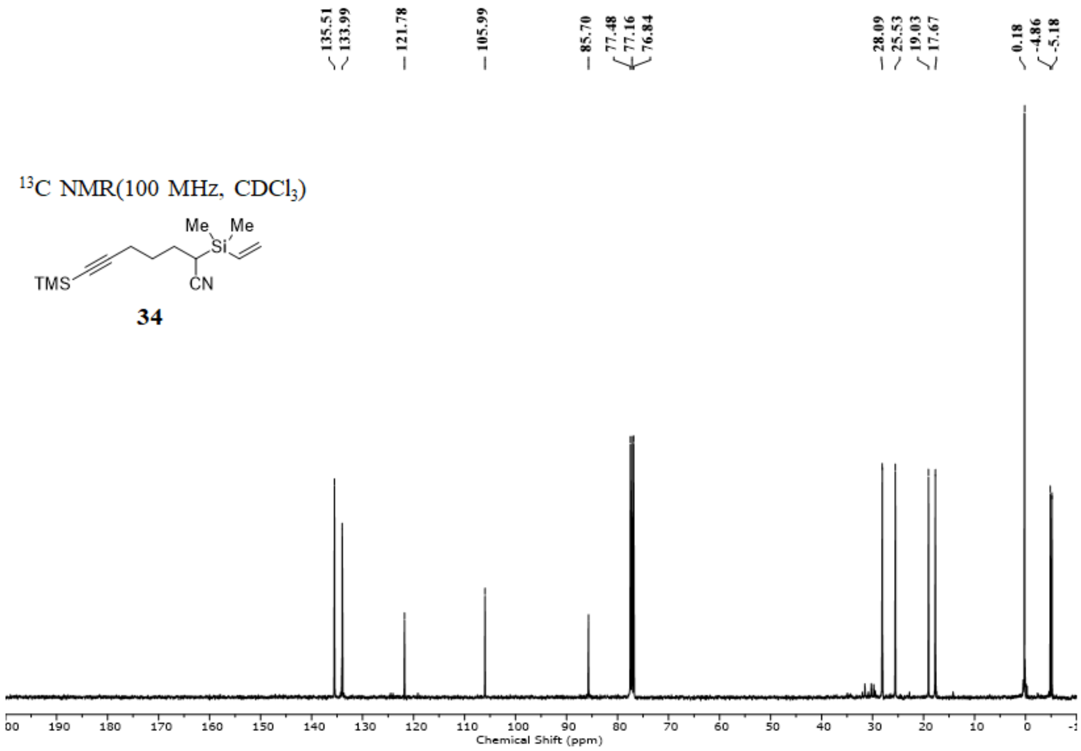

Supplementary Fig. 140. ^{13}C NMR of compound 34. The sample has been recorded in 100 MHz, $CDCl_3$ at 25 °C

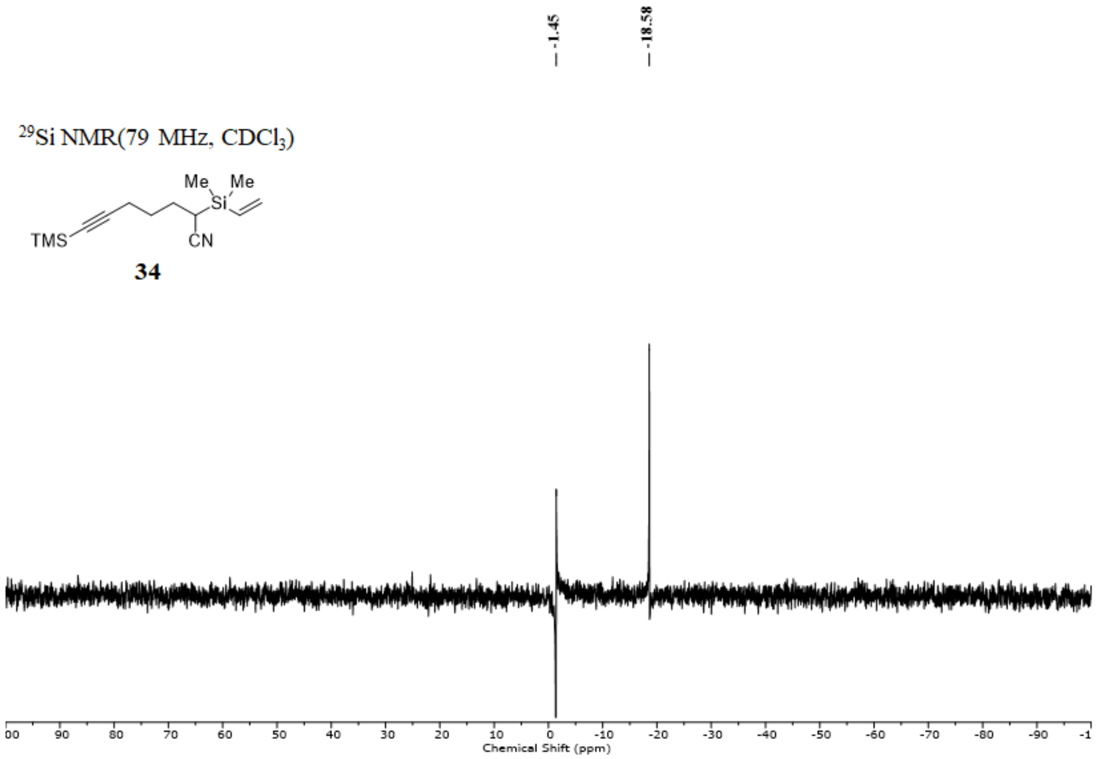

Supplementary Fig. 141. ^{29}Si NMR of compound 34. The sample has been recorded in 79 MHz, $CDCl_3$ at 25 °C

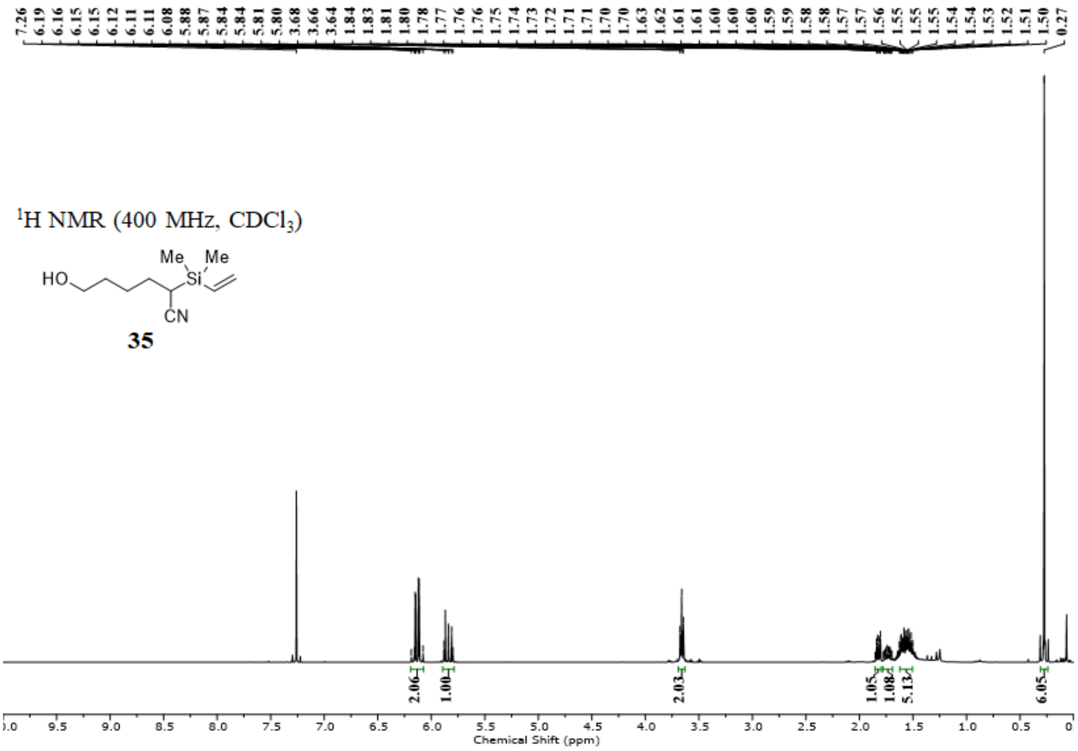

Supplementary Fig. 142. ^{1}H NMR of compound 35. The sample has been recorded in 400 MHz, $CDCl_3$ at 25 °C

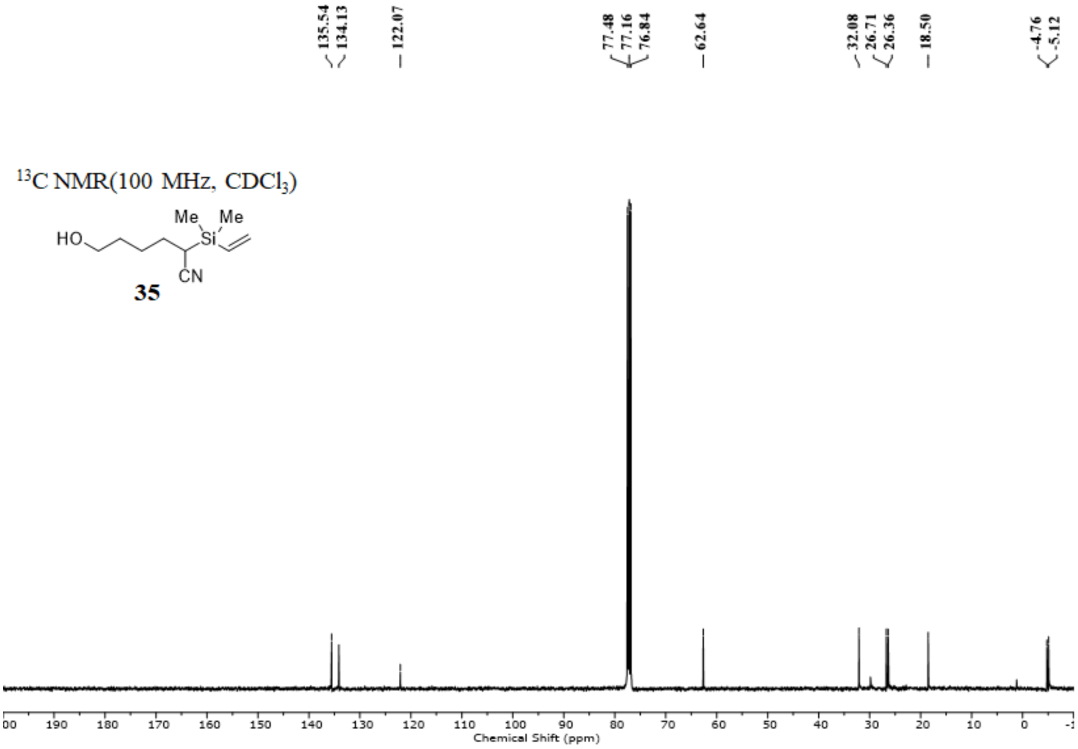

Supplementary Fig. 143. ^{13}C NMR of compound 35. The sample has been recorded in 100 MHz, $CDCl_3$ at 25 °C

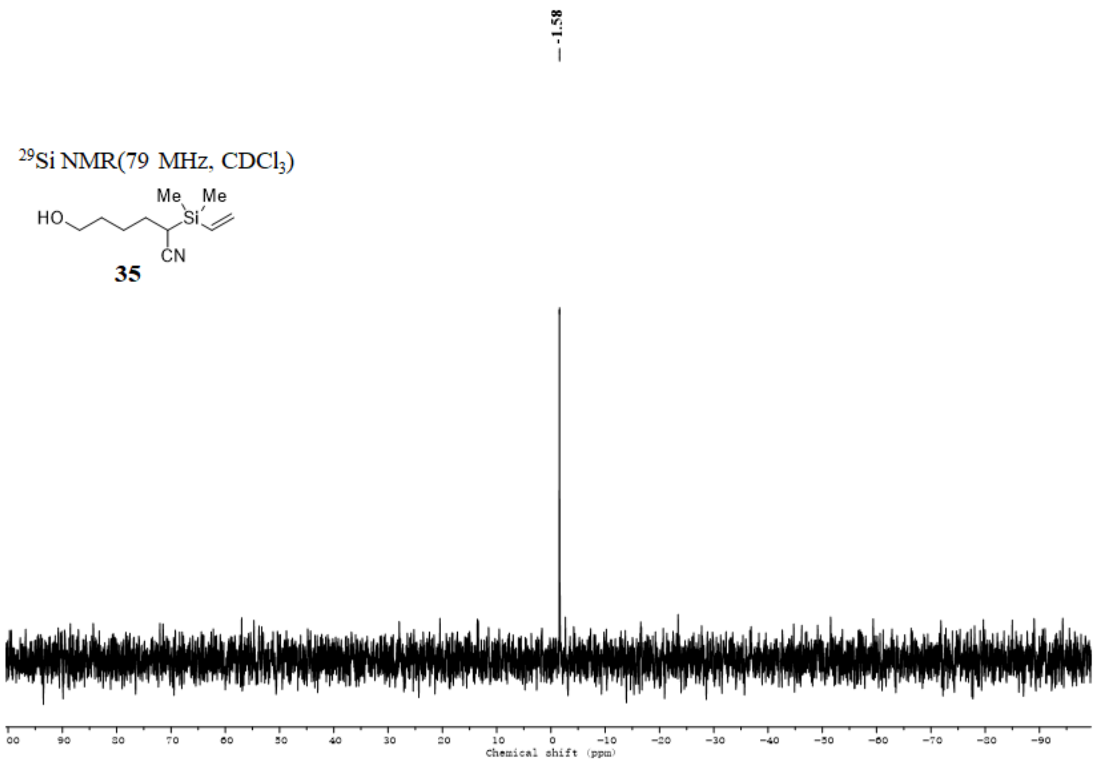

Supplementary Fig. 144. ^{29}Si NMR of compound 35. The sample has been recorded in 79 MHz, $CDCl_3$ at 25 °C

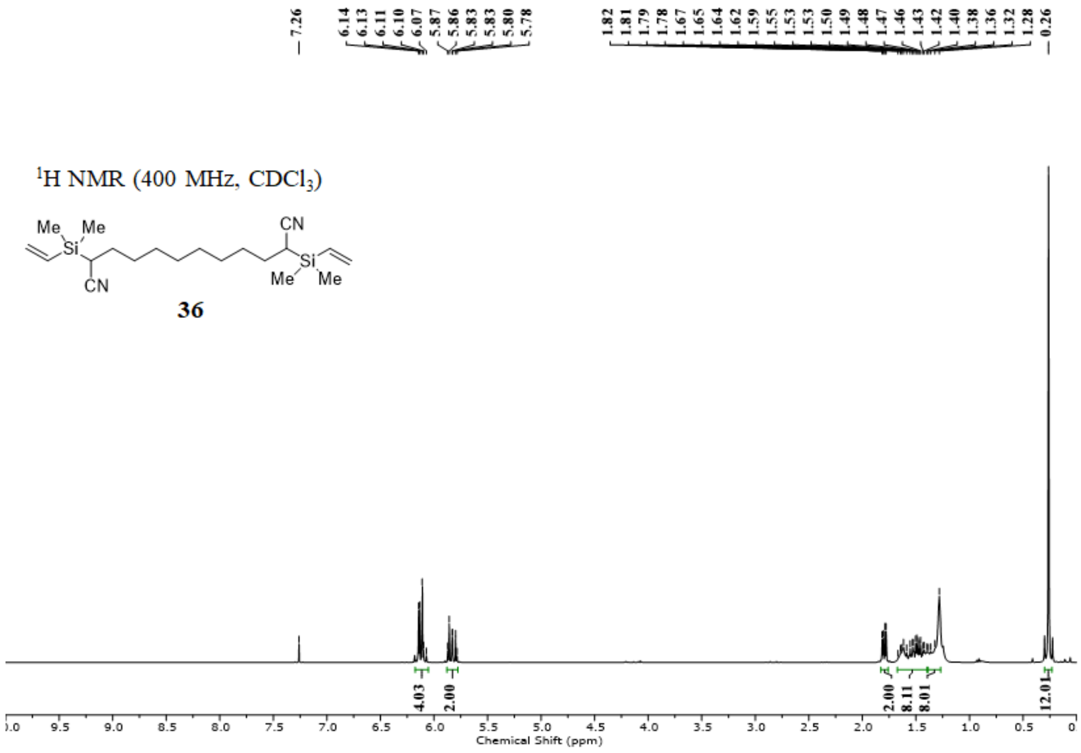

Supplementary Fig. 145. ^{1}H NMR of compound 36. The sample has been recorded in 400 MHz, $CDCl_3$ at 25 °C

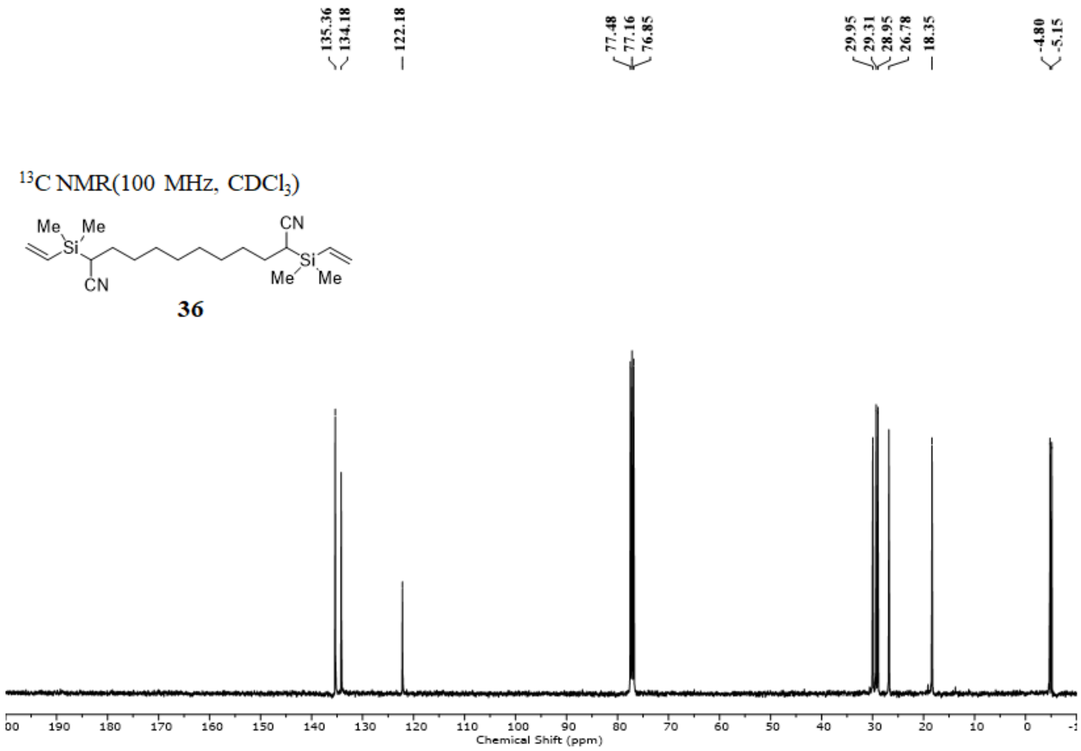

Supplementary Fig. 146. ^{13}C NMR of compound 36. The sample has been recorded in 100 MHz, $CDCl_3$ at 25 °C

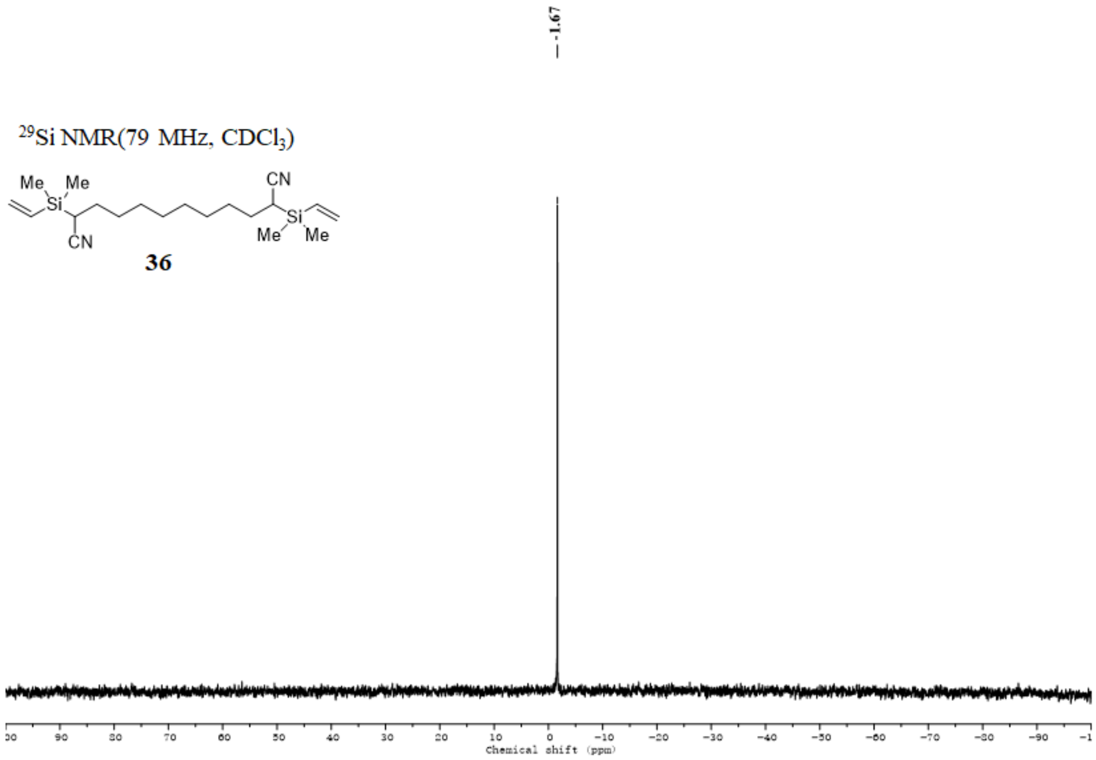

Supplementary Fig. 147. ^{29}Si NMR of compound 36. The sample has been recorded in 79 MHz, $CDCl_3$ at 25 °C

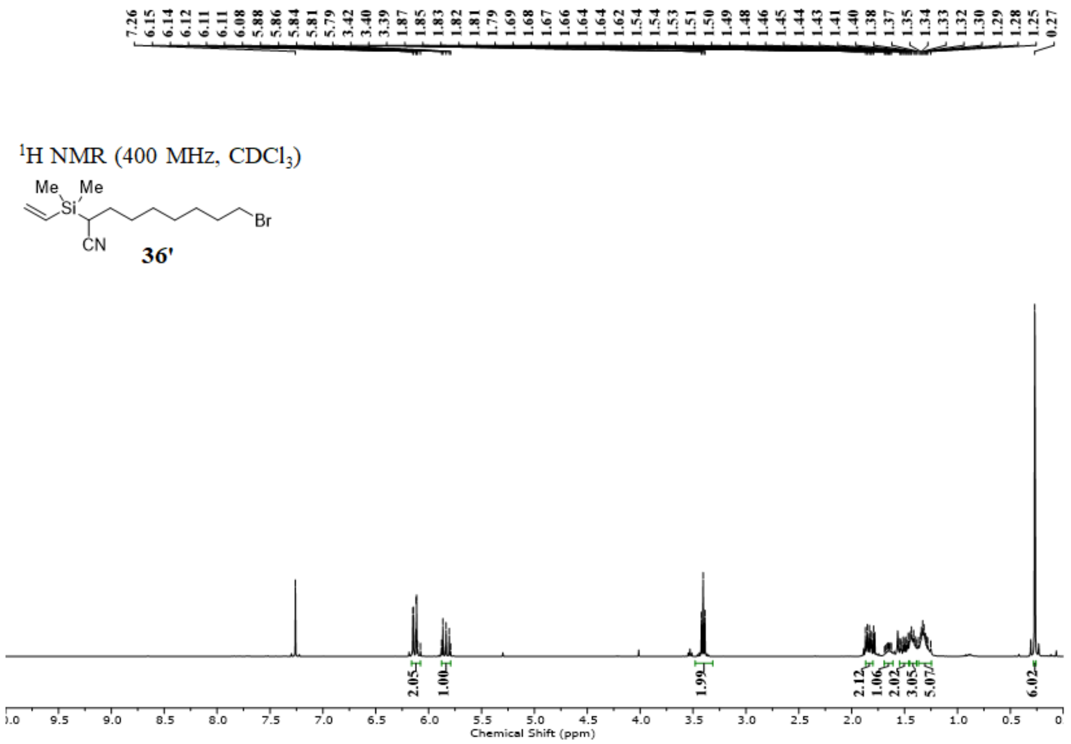

Supplementary Fig. 148. ^{1}H NMR of compound 36'. The sample has been recorded in 400 MHz, $CDCl_3$ at 25 °C

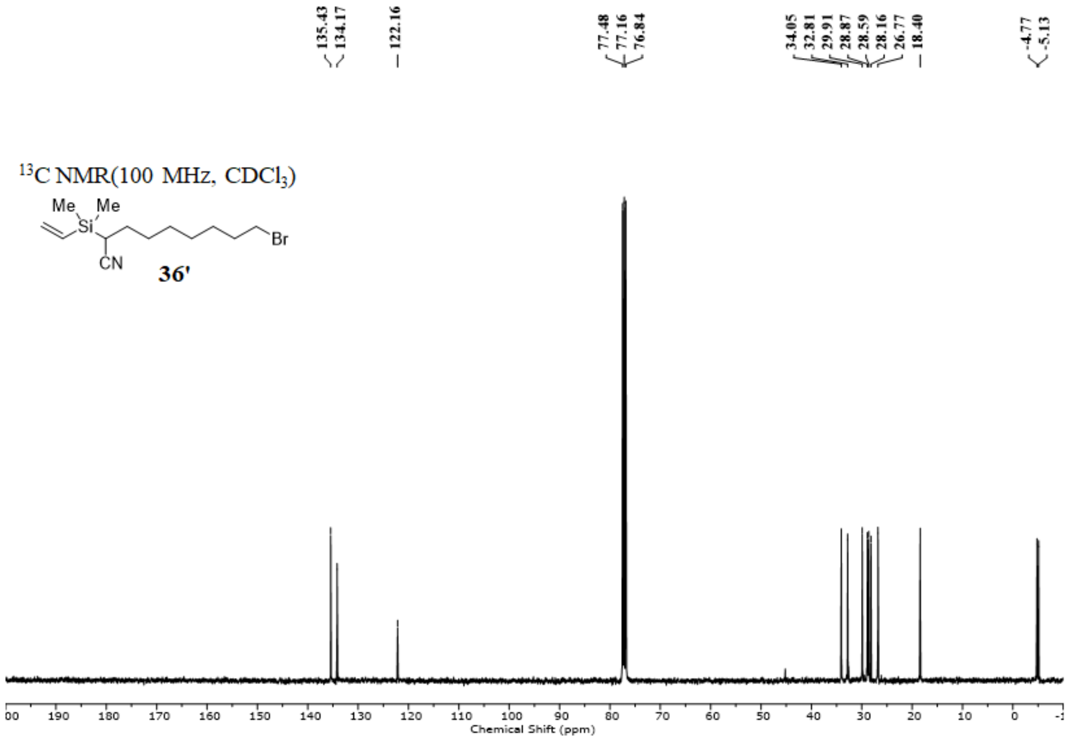

Supplementary Fig. 149. ^{13}C NMR of compound 36'. The sample has been recorded in 100 MHz, $CDCl_3$ at 25 °C

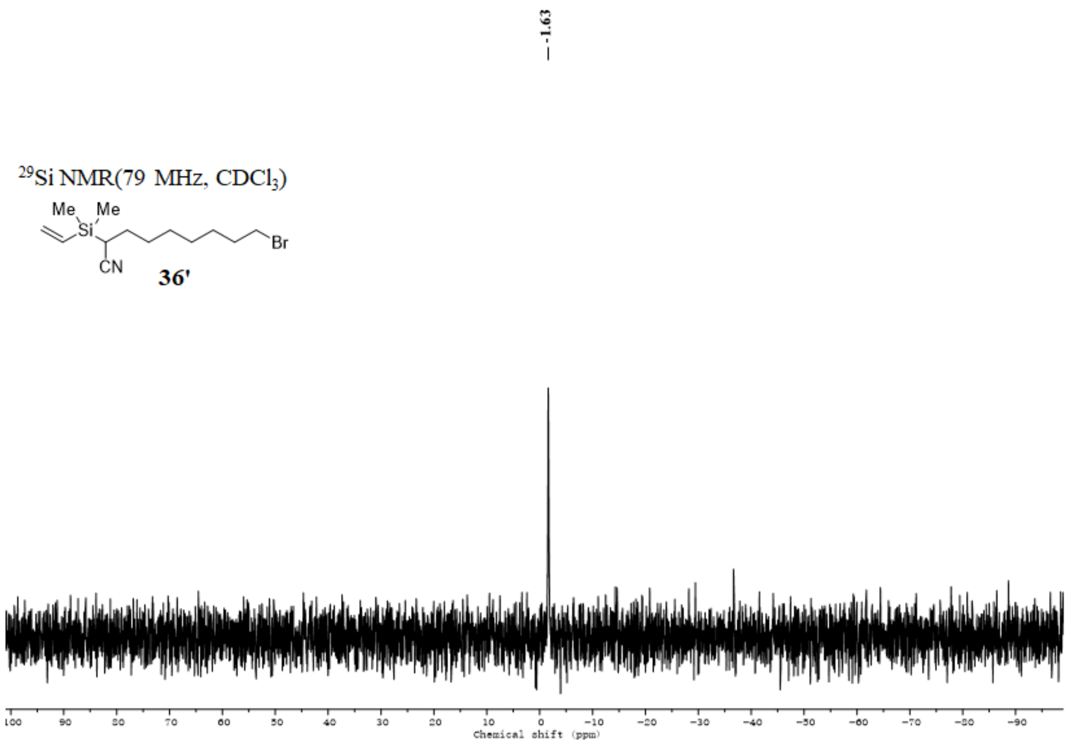

Supplementary Fig. 150. ^{29}Si NMR of compound 36'. The sample has been recorded in 79 MHz, $CDCl_3$ at 25 °C

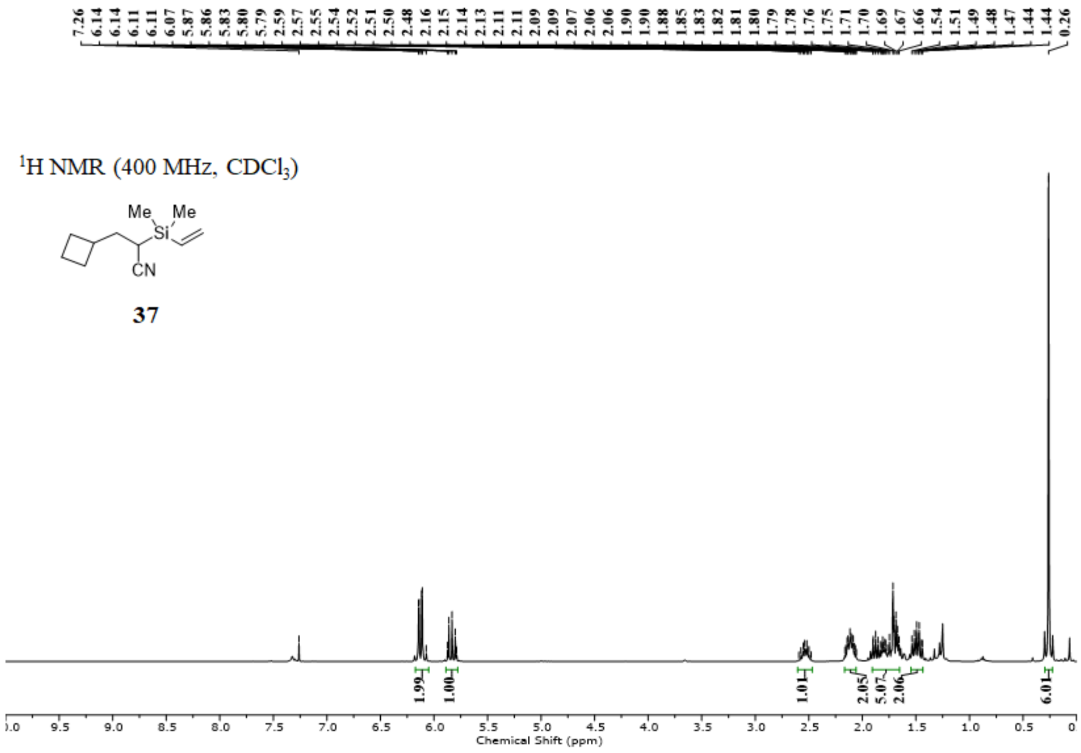

Supplementary Fig. 151. ^{1}H NMR of compound 37. The sample has been recorded in 400 MHz, $CDCl_3$ at 25 °C

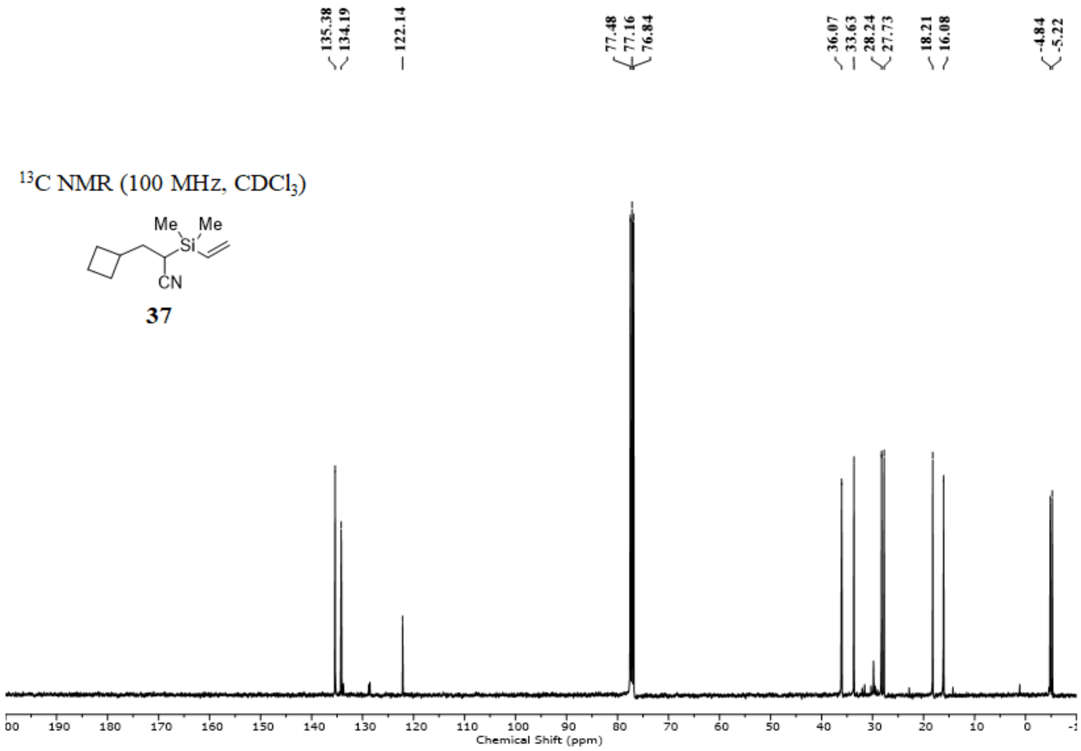

Supplementary Fig. 152. ^{13}C NMR of compound 37. The sample has been recorded in 100 MHz, $CDCl_3$ at 25 °C

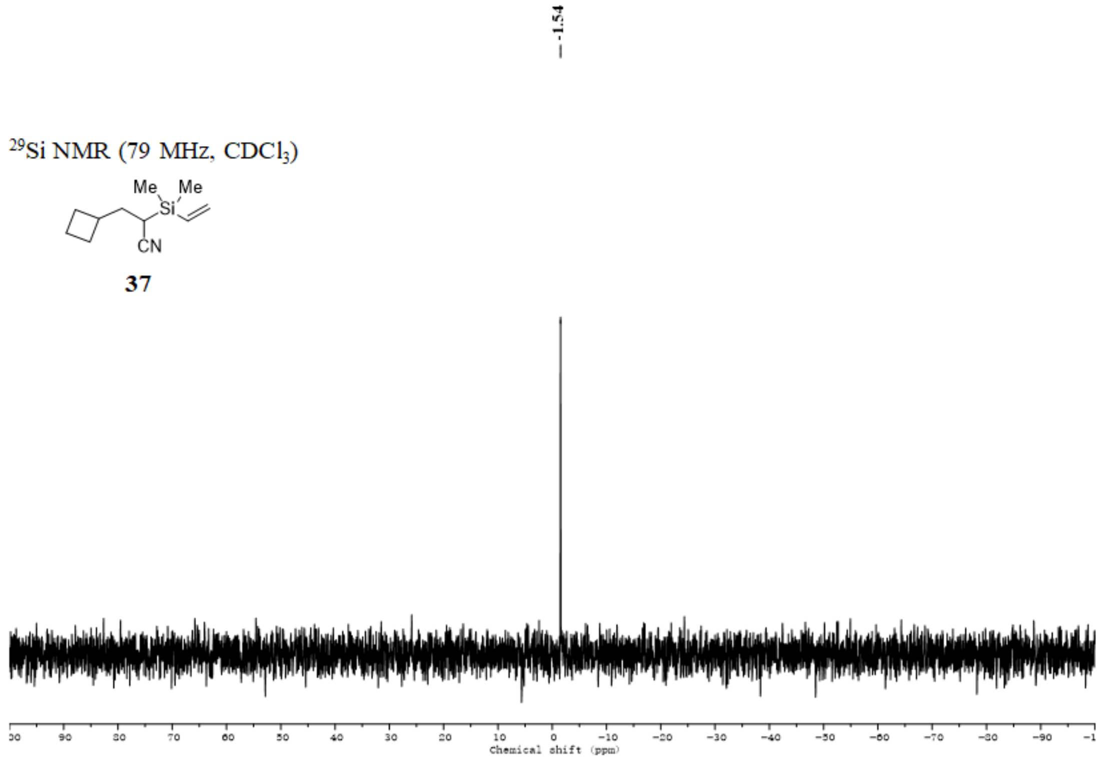

Supplementary Fig. 153. ^{29}Si NMR of compound 37. The sample has been recorded in 79 MHz, $CDCl_3$ at 25 °C

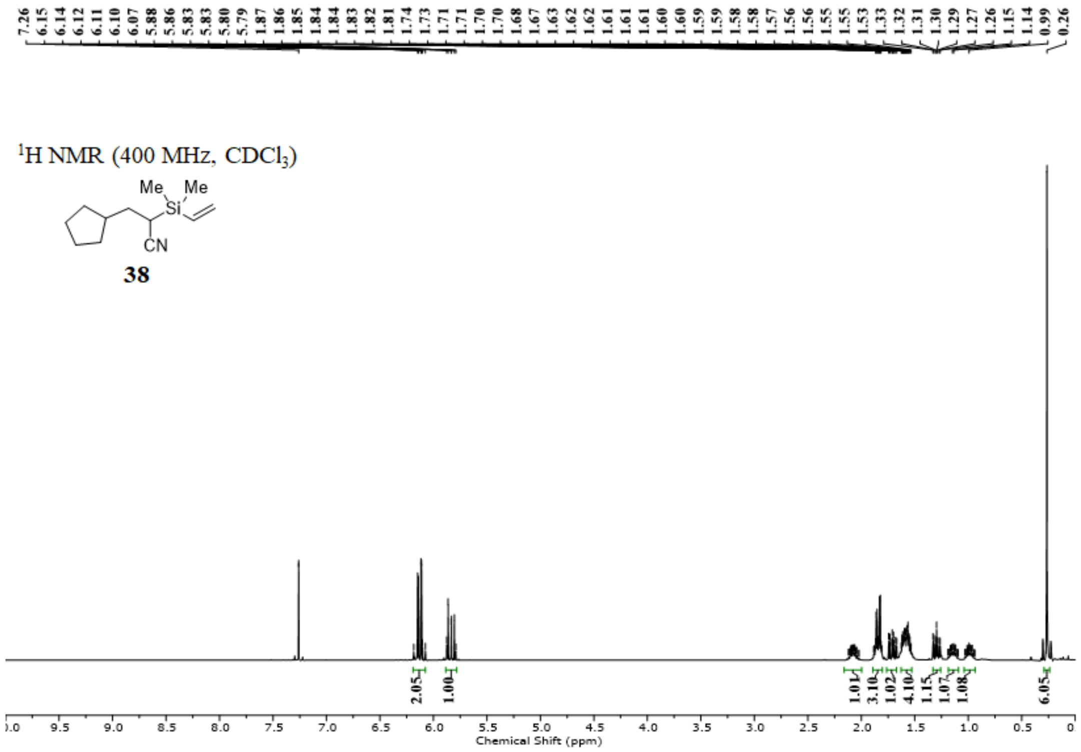

Supplementary Fig. 154. ^{1}H NMR of compound 38. The sample has been recorded in 400 MHz, $CDCl_3$ at 25 °C

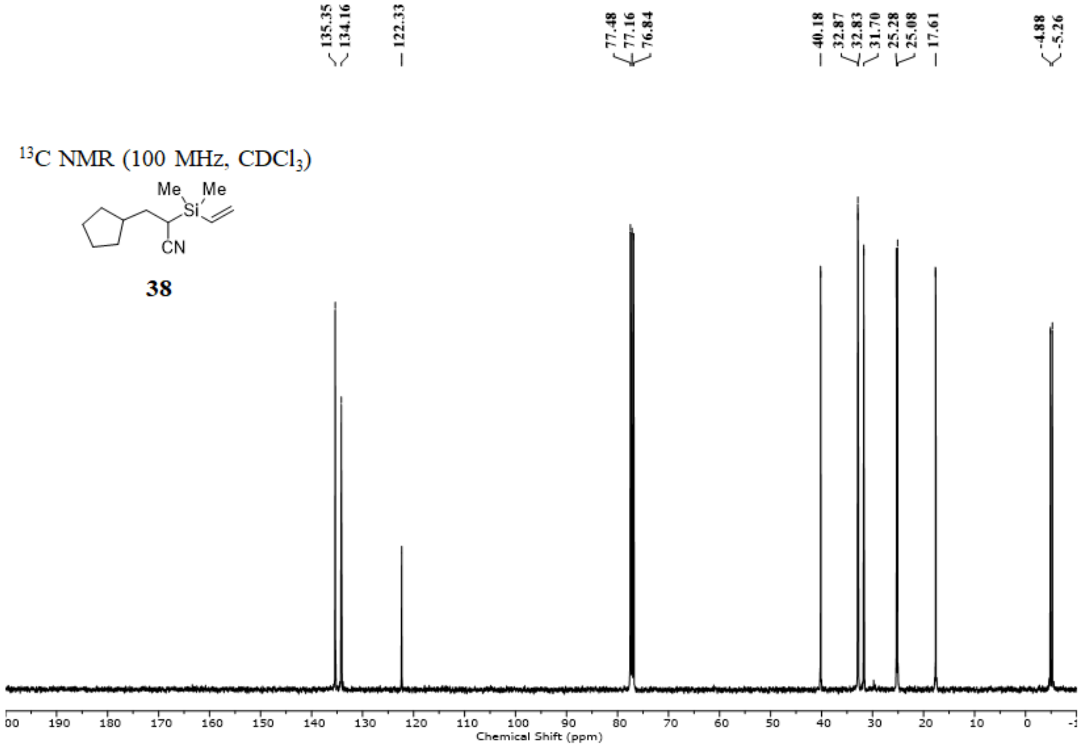

Supplementary Fig. 155. ^{13}C NMR of compound 38. The sample has been recorded in 100 MHz, $CDCl_3$ at 25 °C

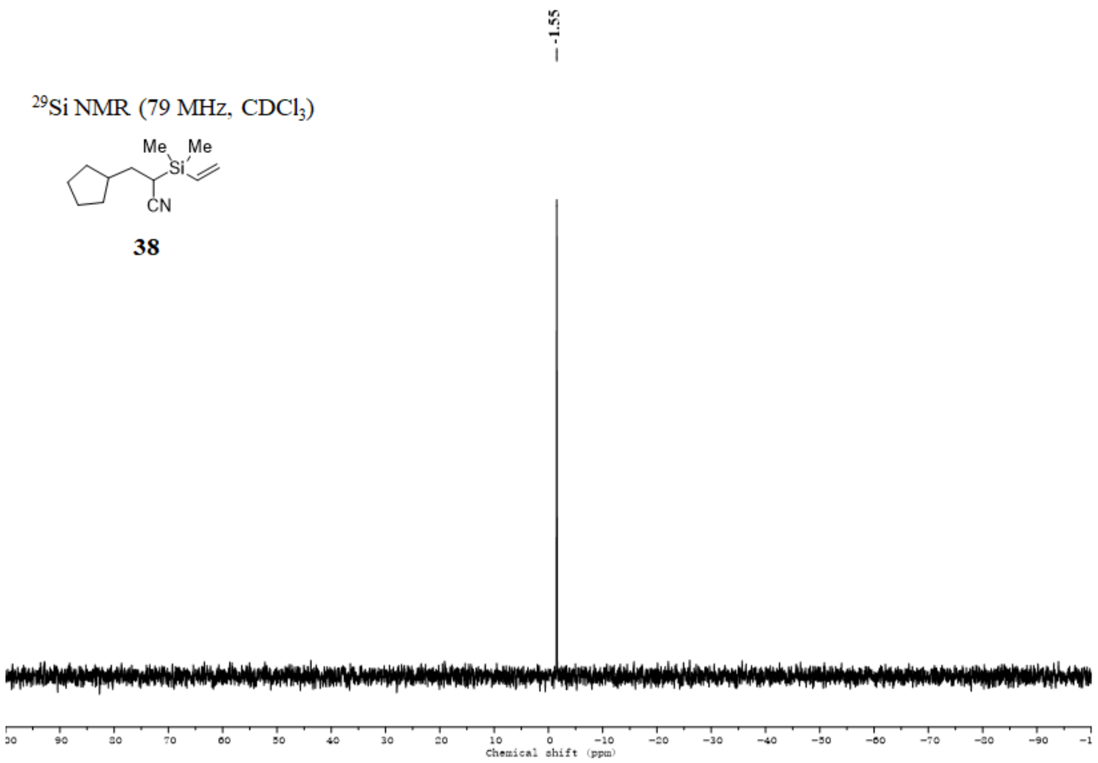

Supplementary Fig. 156. ^{29}Si NMR of compound 38. The sample has been recorded in 79 MHz, $CDCl_3$ at 25 °C

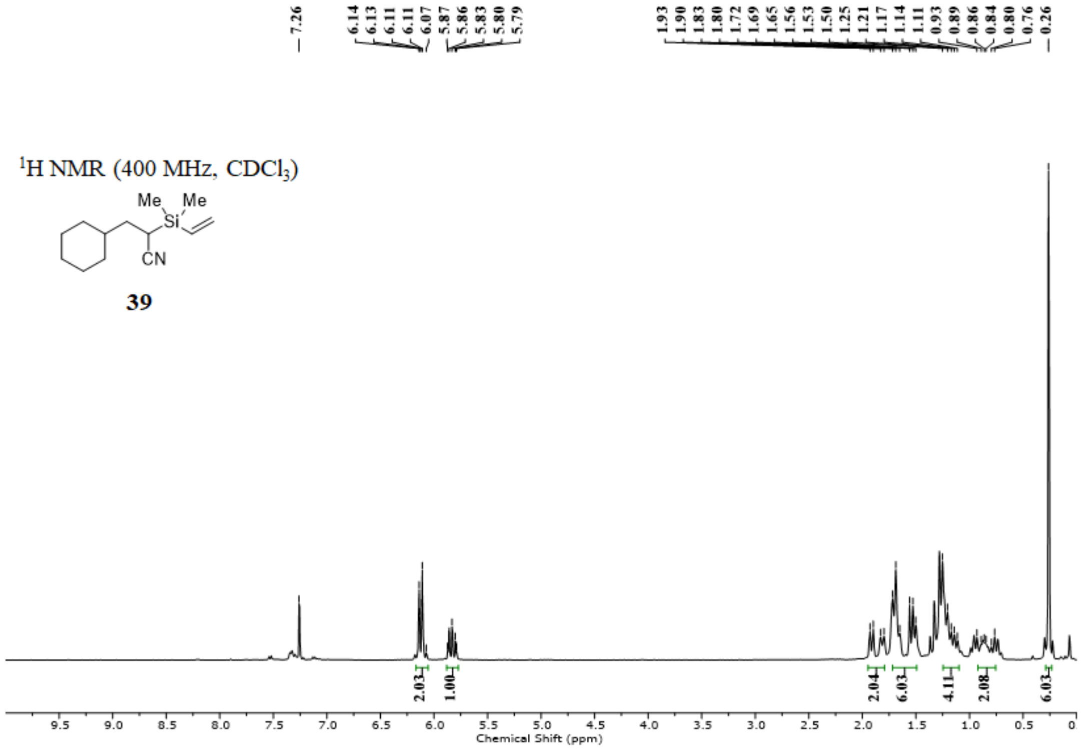

Supplementary Fig. 157. ^{1}H NMR of compound 39. The sample has been recorded in 400 MHz, $CDCl_3$ at 25 °C

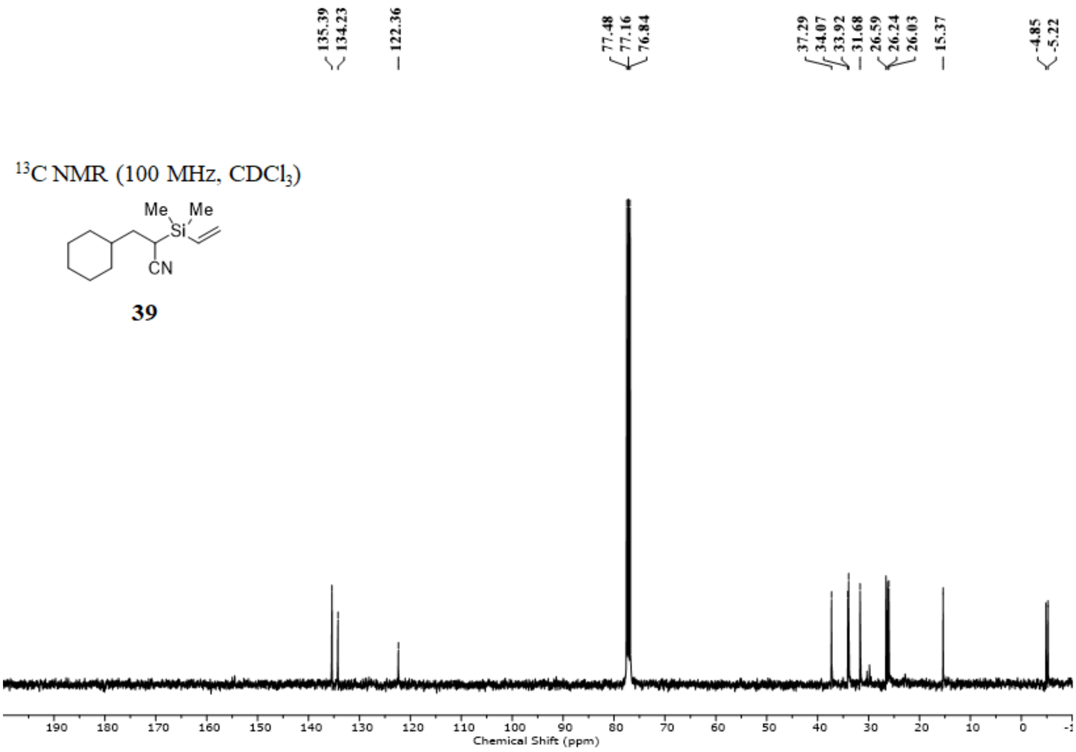

Supplementary Fig. 158. ^{13}C NMR of compound 39. The sample has been recorded in 100 MHz, $CDCl_3$ at 25 °C

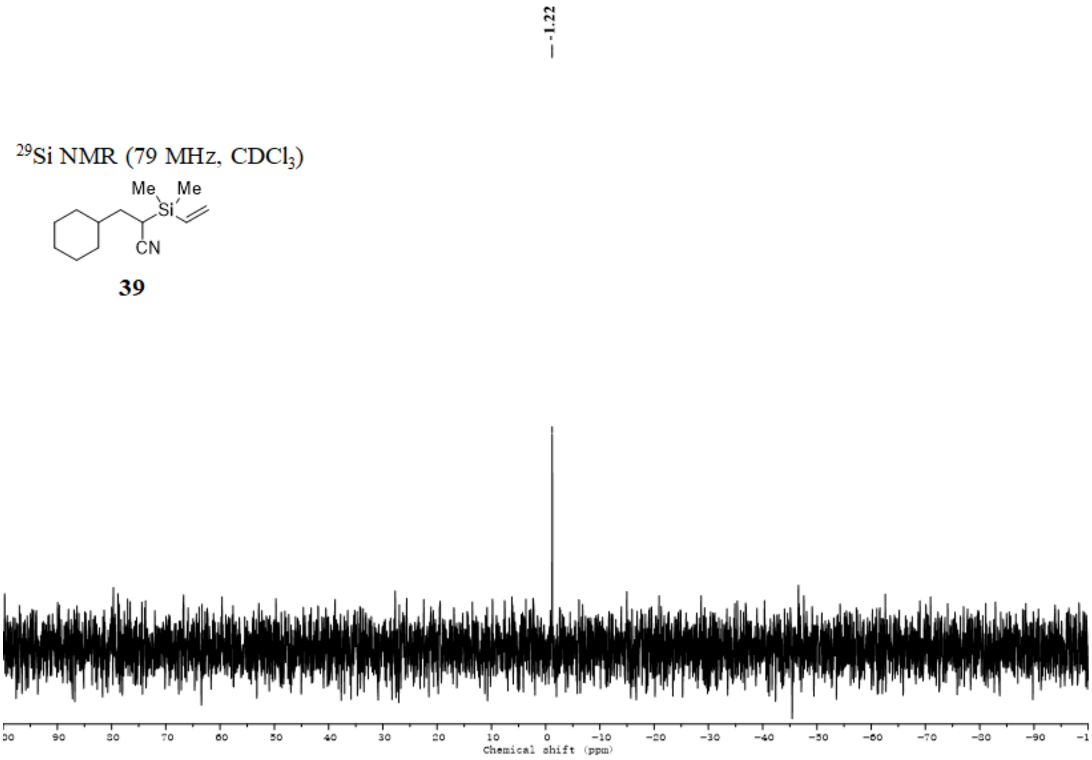

Supplementary Fig. 159. ^{29}Si NMR of compound 39. The sample has been recorded in 79 MHz, $CDCl_3$ at 25 °C

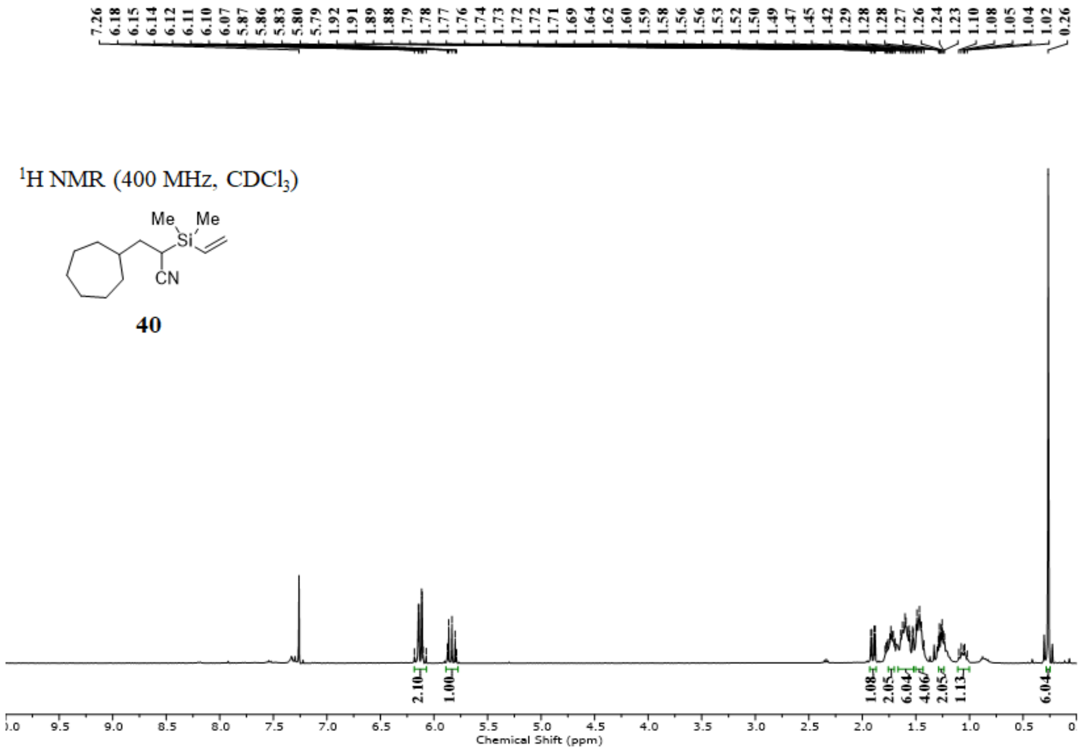

Supplementary Fig. 160. ^{1}H NMR of compound 40. The sample has been recorded in 400 MHz, $CDCl_3$ at 25 °C

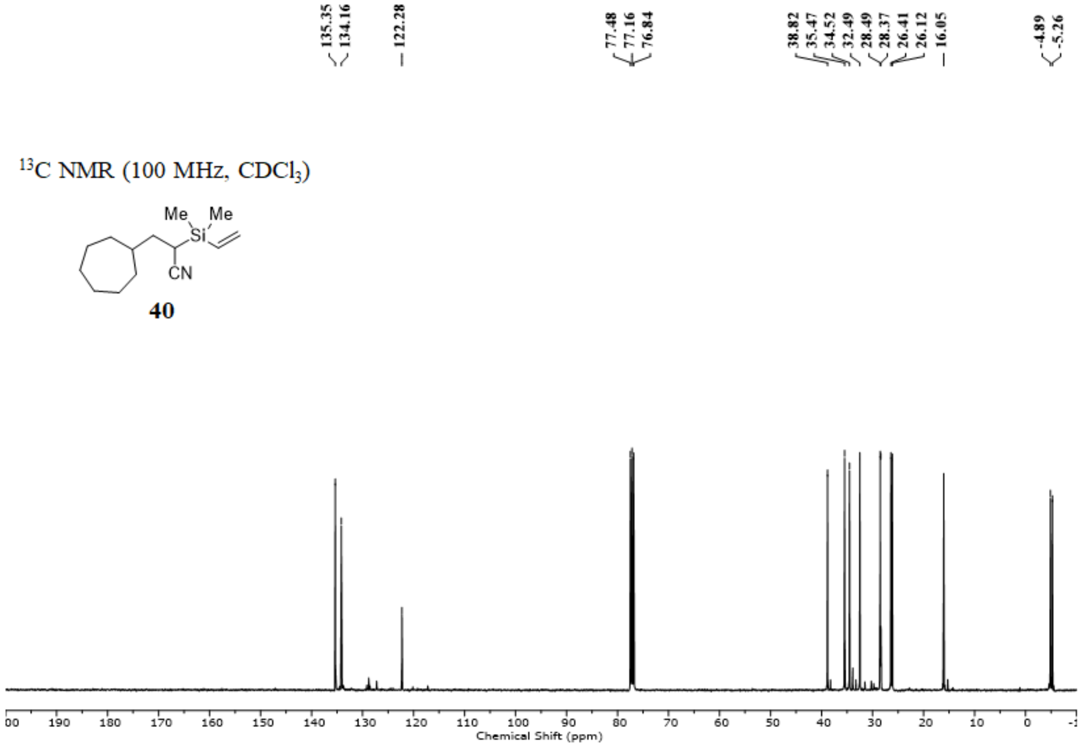

Supplementary Fig. 161. ^{13}C NMR of compound 40. The sample has been recorded in 100 MHz, $CDCl_3$ at 25 °C

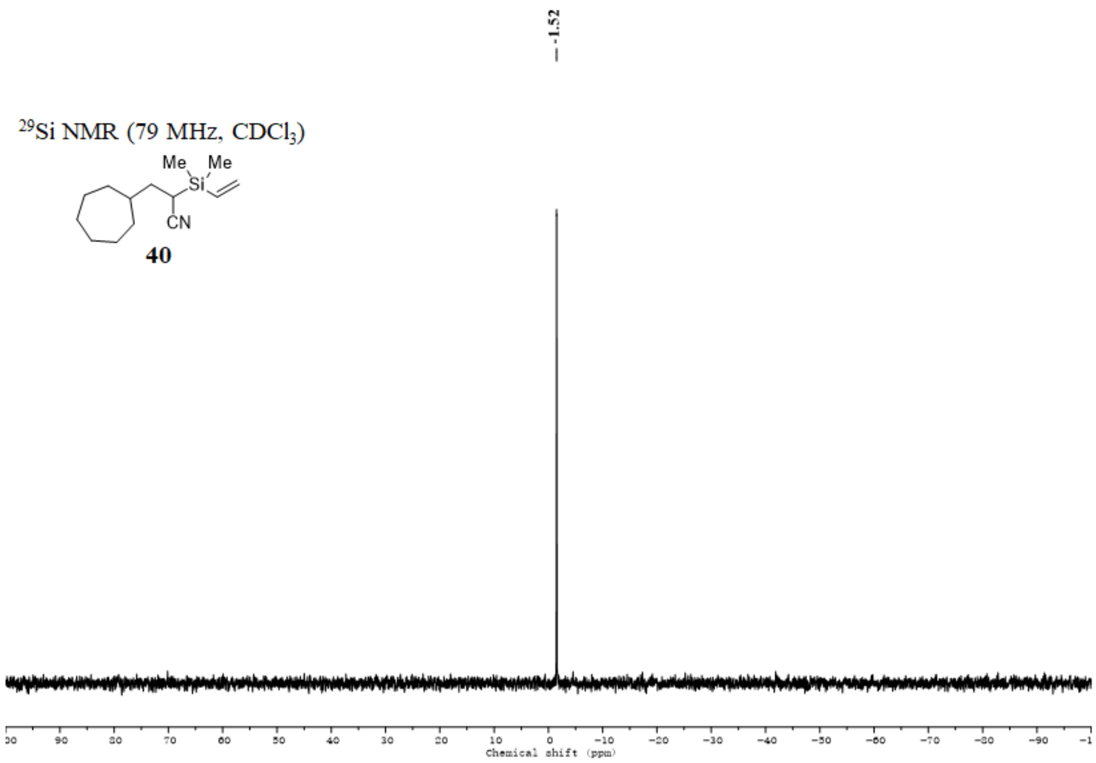

Supplementary Fig. 162. ^{29}Si NMR of compound 40. The sample has been recorded in 79 MHz, $CDCl_3$ at 25 °C

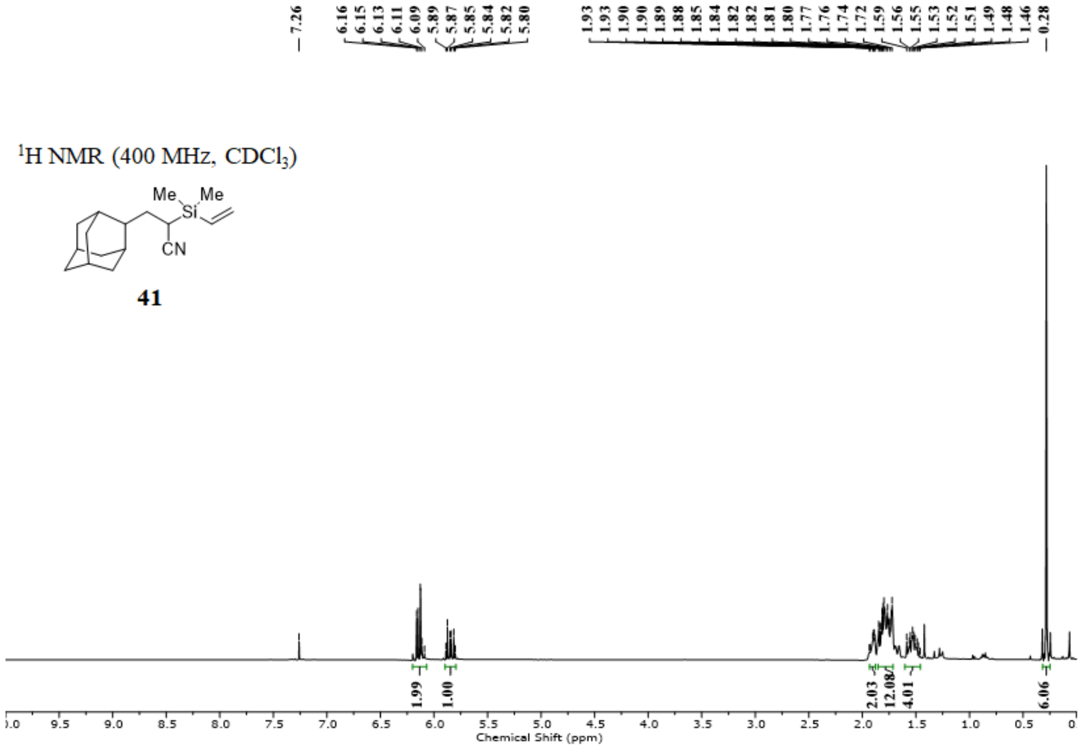

Supplementary Fig. 163. ^{1}H NMR of compound 41. The sample has been recorded in 400 MHz, $CDCl_3$ at 25 °C

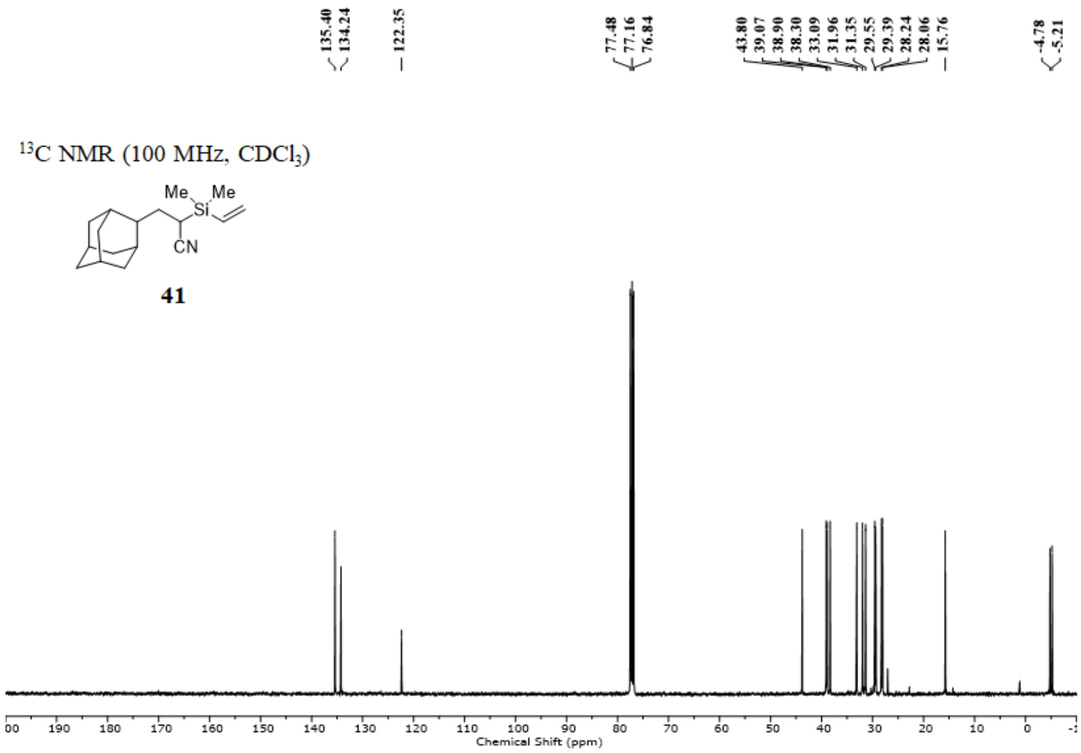

Supplementary Fig. 164. ^{13}C NMR of compound 41. The sample has been recorded in 100 MHz, $CDCl_3$ at 25 °C

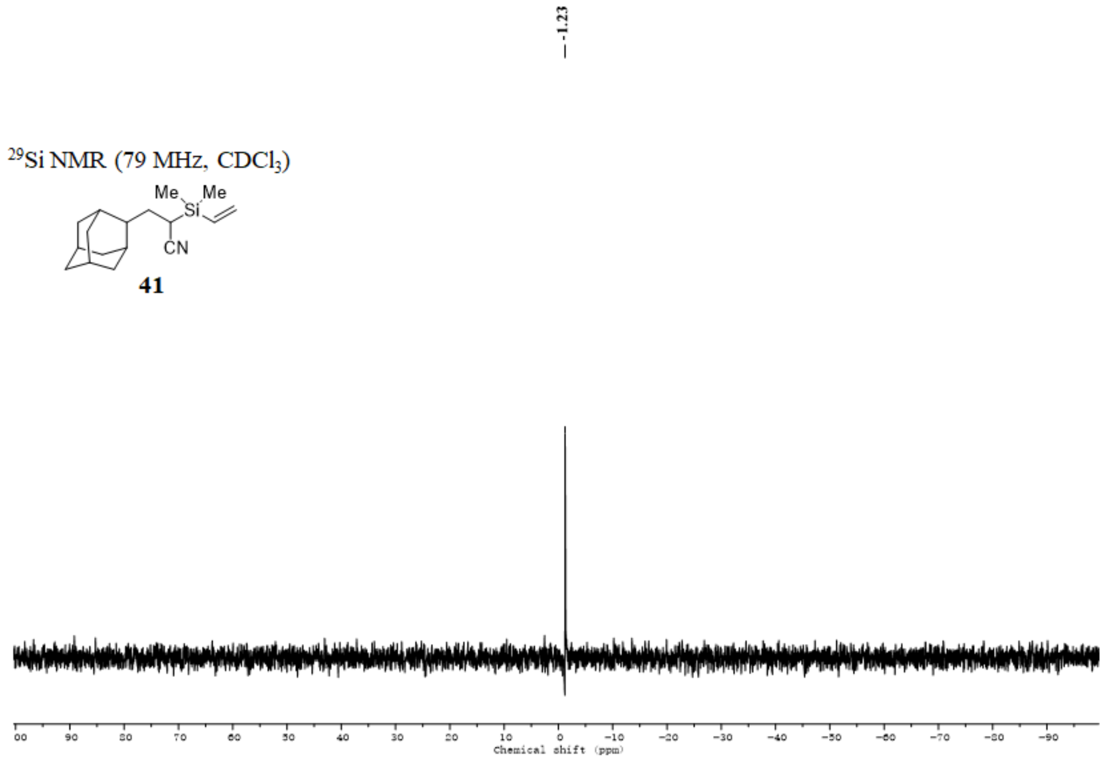

Supplementary Fig. 165. ^{29}Si NMR of compound 41. The sample has been recorded in 79 MHz, $CDCl_3$ at 25 °C

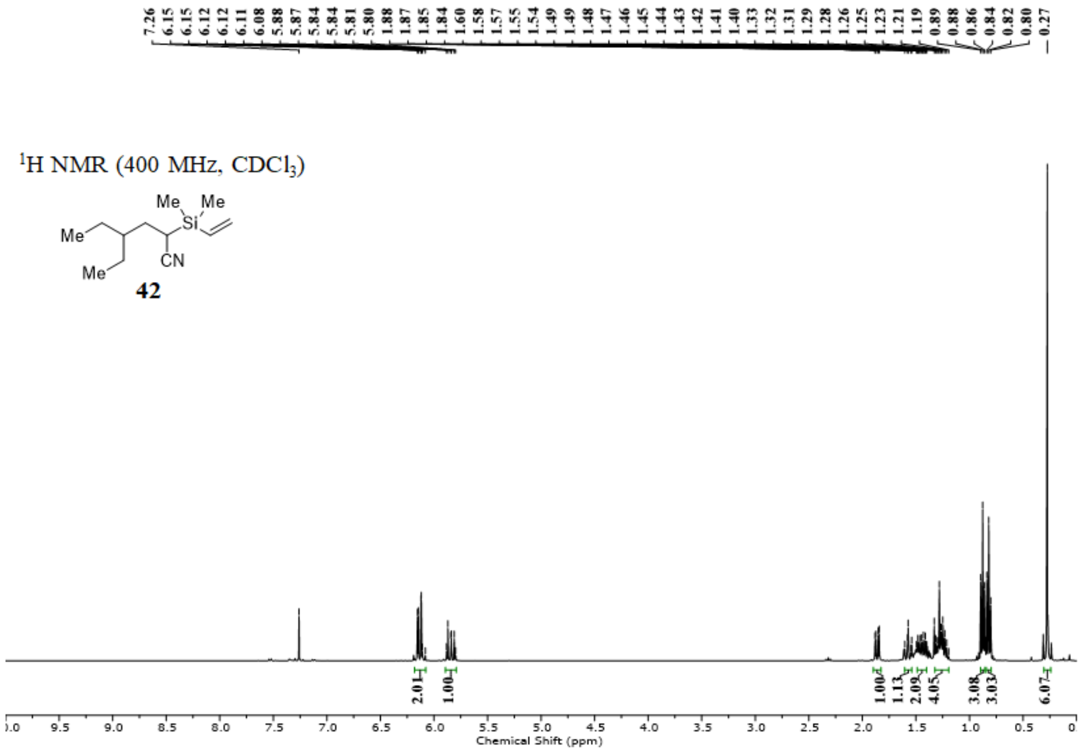

Supplementary Fig. 166. ^{1}H NMR of compound 42. The sample has been recorded in 400 MHz, $CDCl_3$ at 25 °C

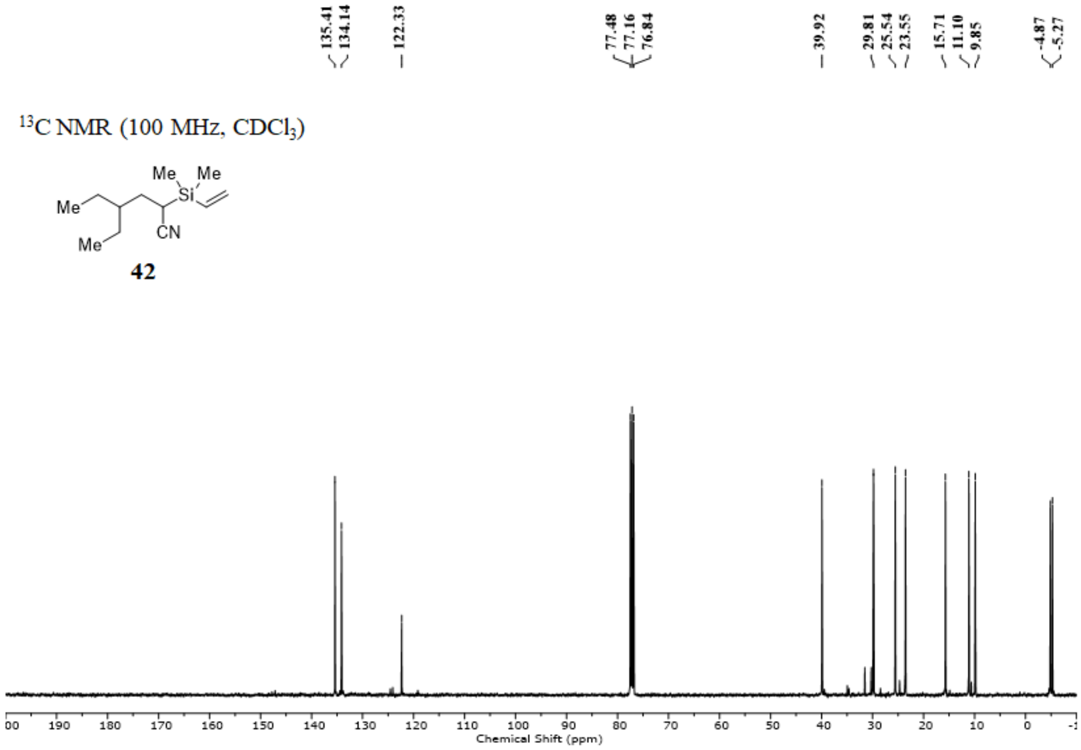

Supplementary Fig. 167. ^{13}C NMR of compound 42. The sample has been recorded in 100 MHz, $CDCl_3$ at 25 °C

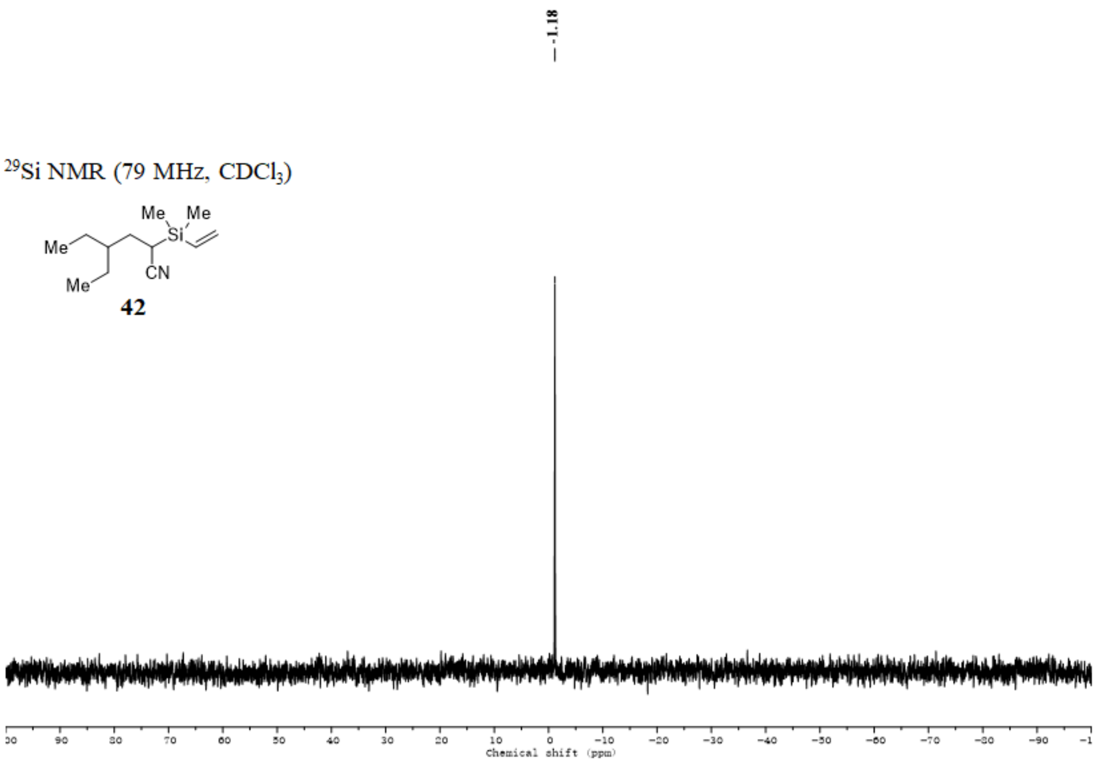

Supplementary Fig. 168. ^{29}Si NMR of compound 42. The sample has been recorded in 79 MHz, $CDCl_3$ at 25 °C

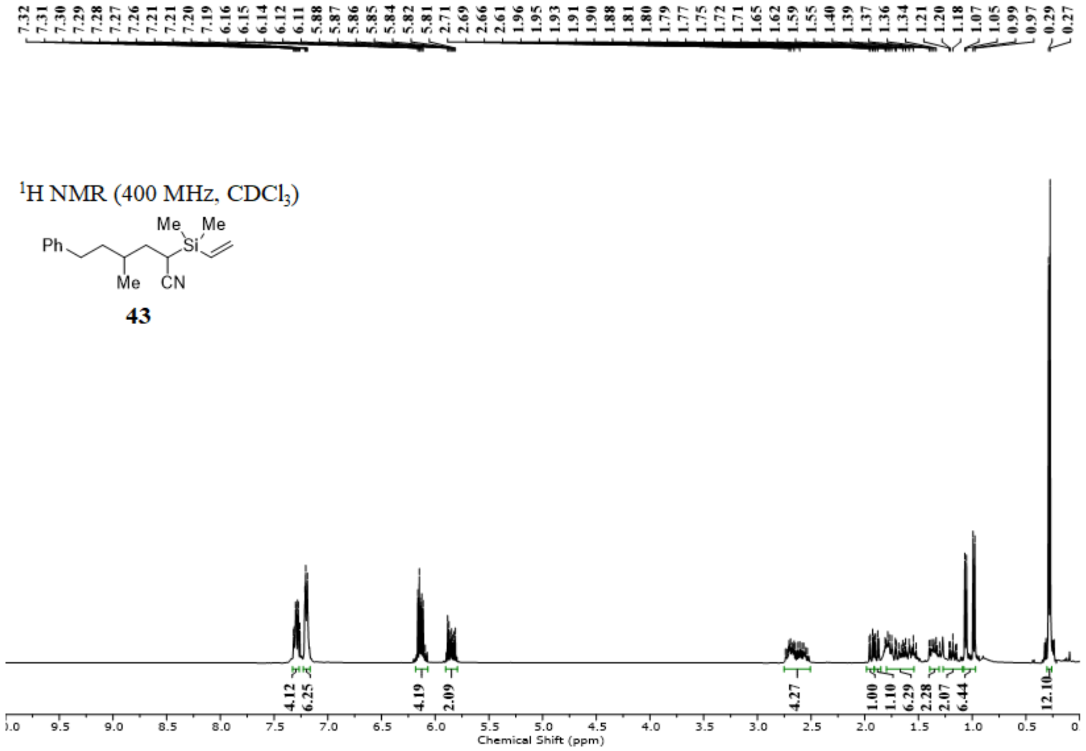

Supplementary Fig. 169. ^{1}H NMR of compound 43. The sample has been recorded in 400 MHz, $CDCl_3$ at 25 °C

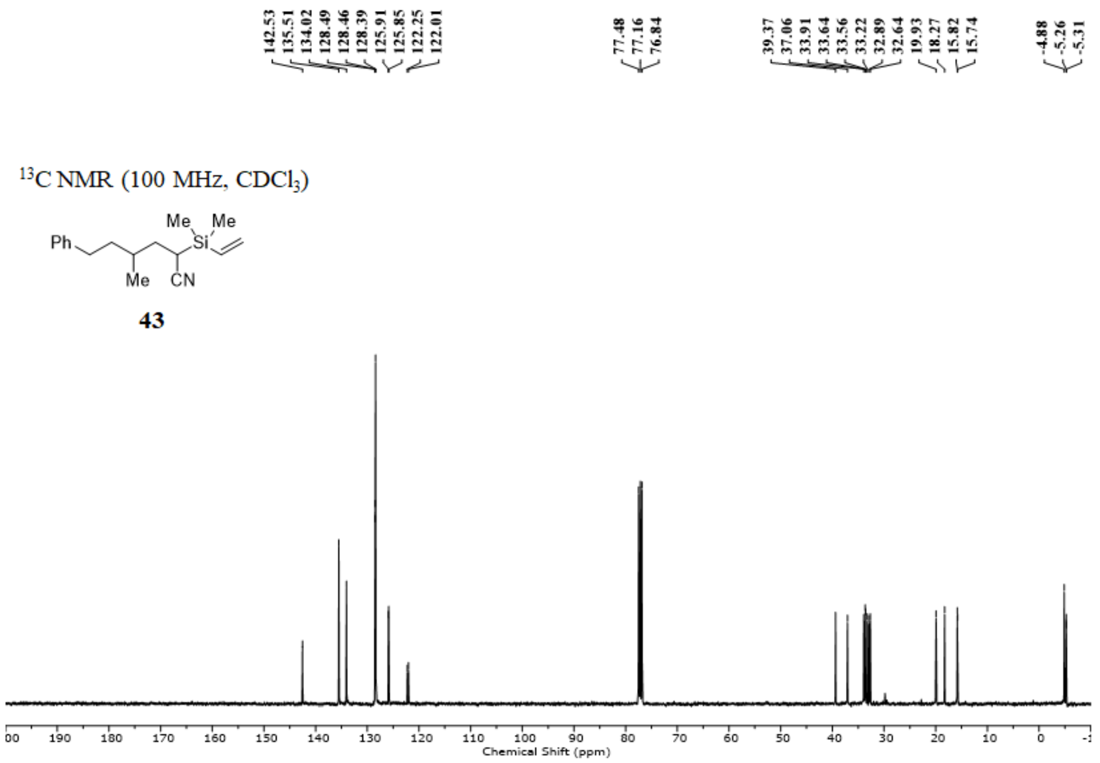

Supplementary Fig. 170. ^{13}C NMR of compound 43. The sample has been recorded in 100 MHz, $CDCl_3$ at 25 °C

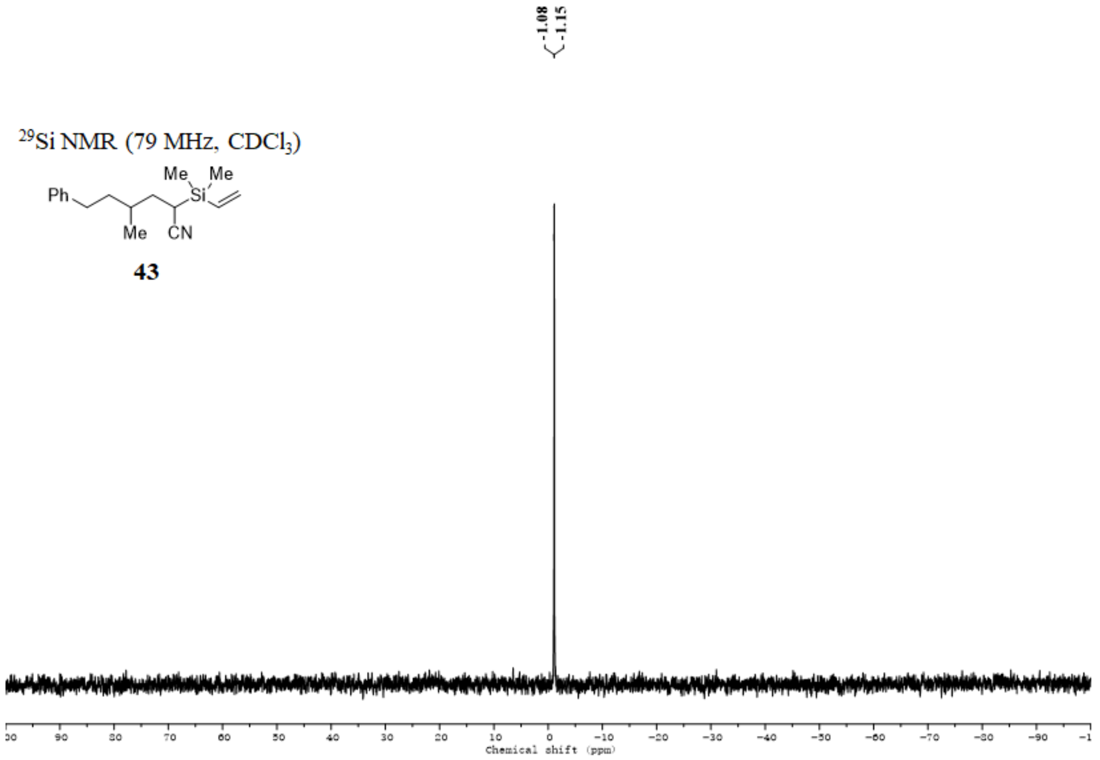

Supplementary Fig. 171. ^{29}Si NMR of compound 43. The sample has been recorded in 79 MHz, $CDCl_3$ at 25 °C

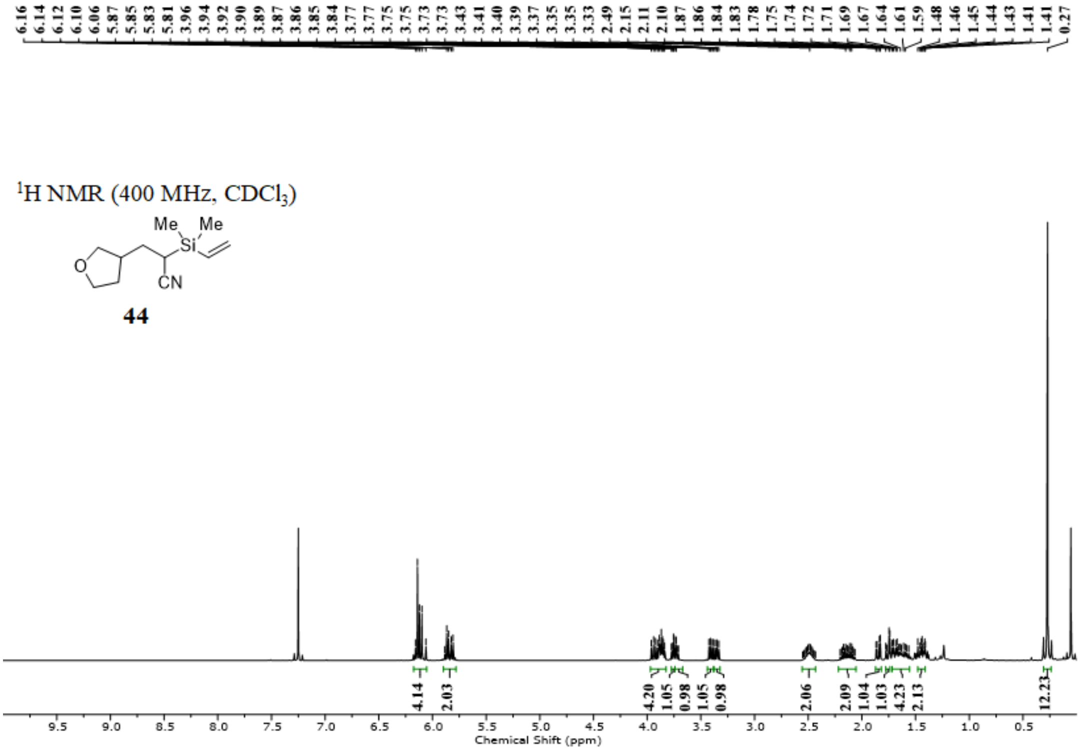

Supplementary Fig. 172. ^{1}H NMR of compound 44. The sample has been recorded in 400 MHz, $CDCl_3$ at 25 °C

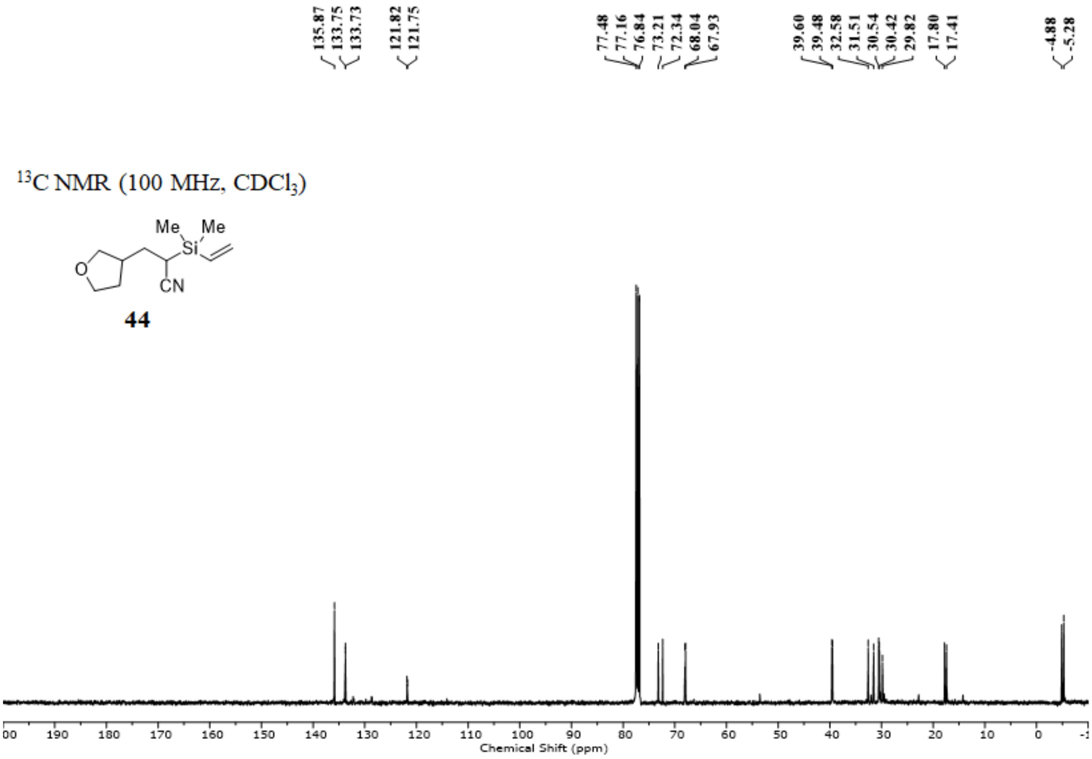

Supplementary Fig. 173. ^{13}C NMR of compound 44. The sample has been recorded in 100 MHz, $CDCl_3$ at 25 °C

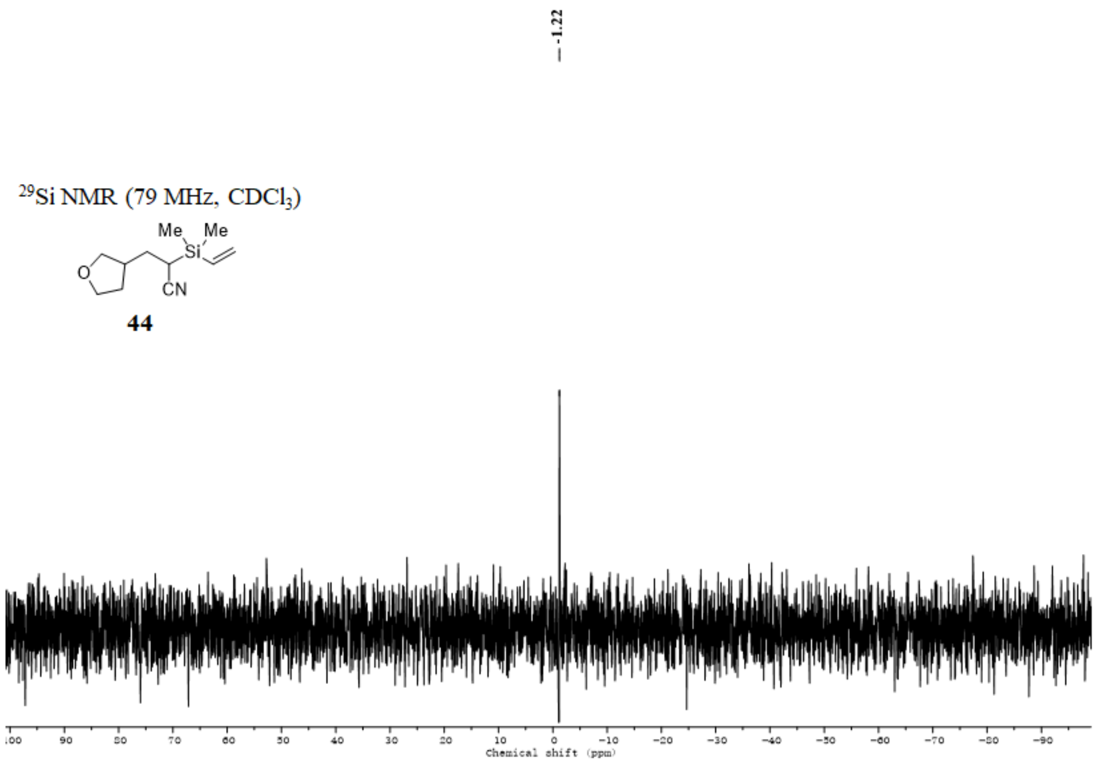

Supplementary Fig. 174. ^{29}Si NMR of compound 44. The sample has been recorded in 79 MHz, $CDCl_3$ at 25 °C

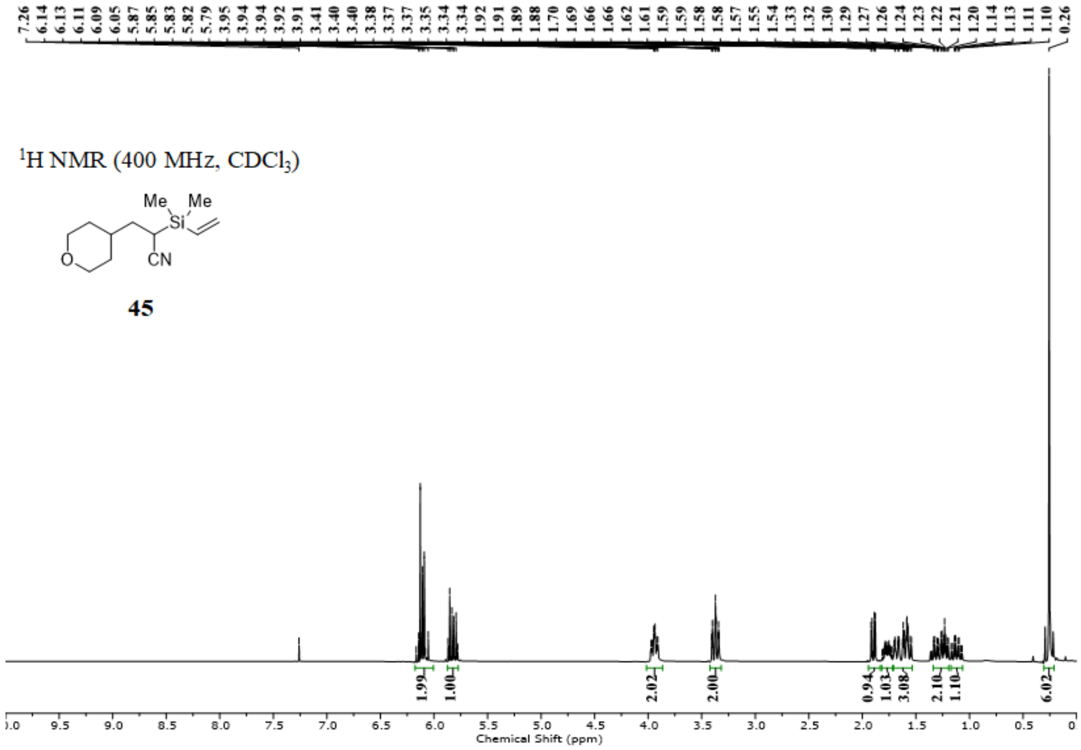

Supplementary Fig. 175. ^{1}H NMR of compound 45. The sample has been recorded in 400 MHz, $CDCl_3$ at 25 °C

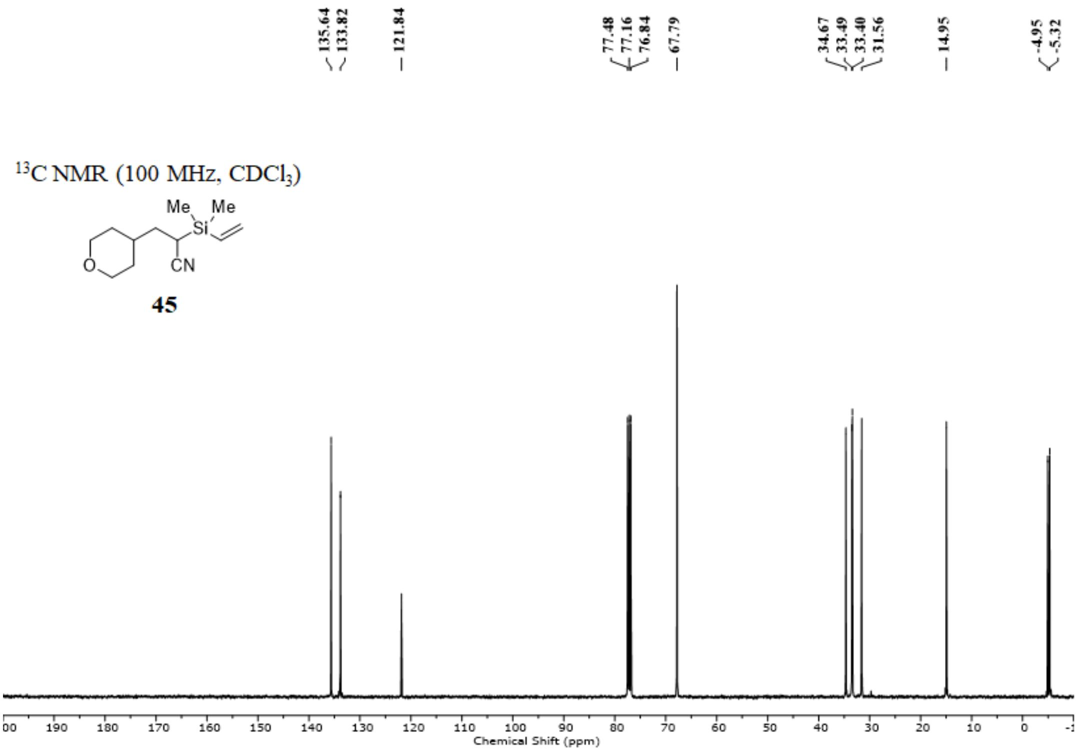

Supplementary Fig. 176. ^{13}C NMR of compound 45. The sample has been recorded in 100 MHz, $CDCl_3$ at 25 °C

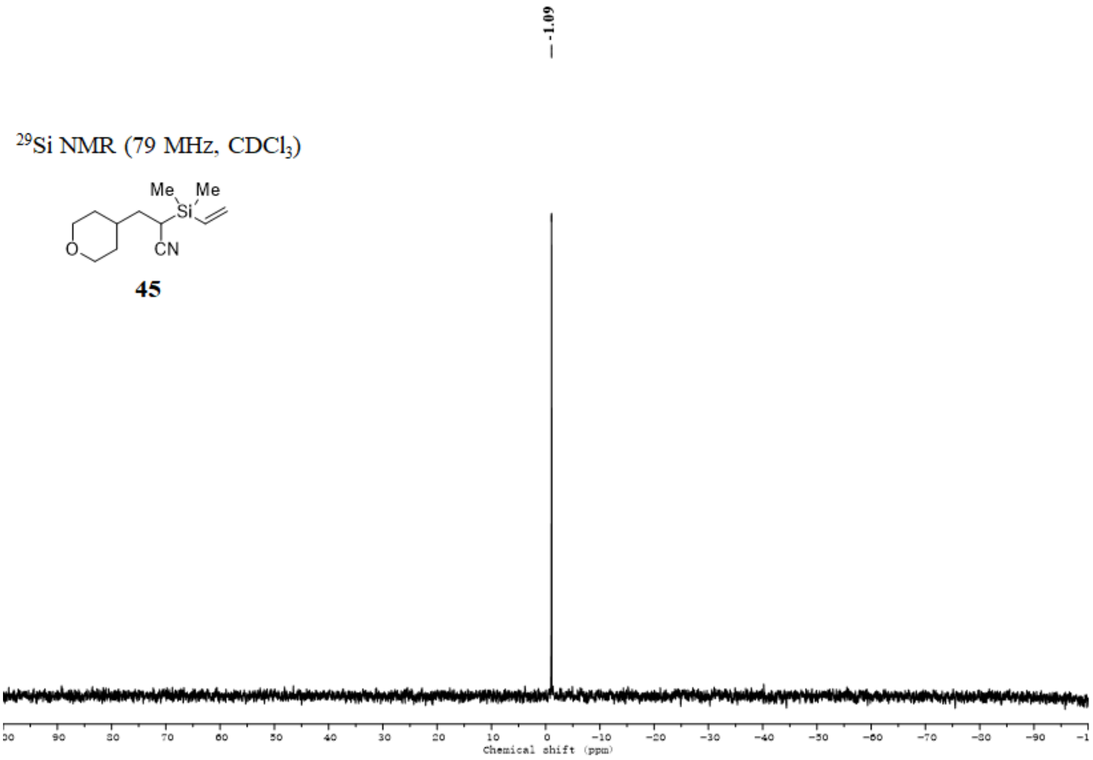

Supplementary Fig. 177. ^{29}Si NMR of compound 45. The sample has been recorded in 79 MHz, $CDCl_3$ at 25 °C

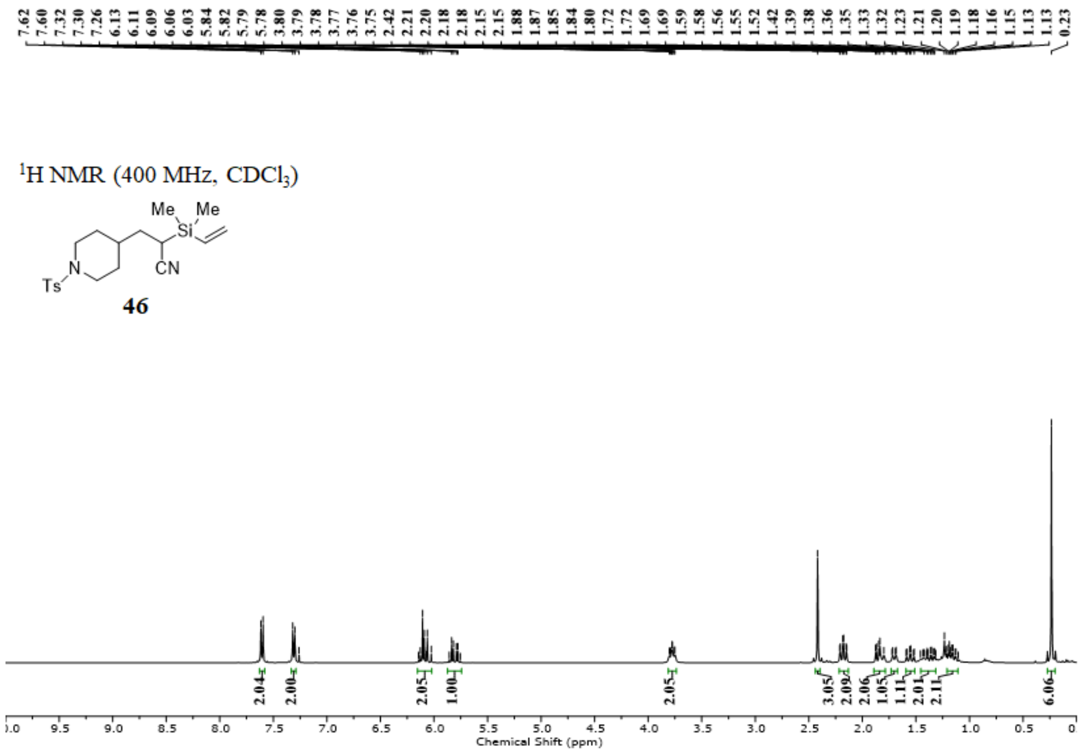

Supplementary Fig. 178. ^{1}H NMR of compound 46. The sample has been recorded in 400 MHz, $CDCl_3$ at 25 °C

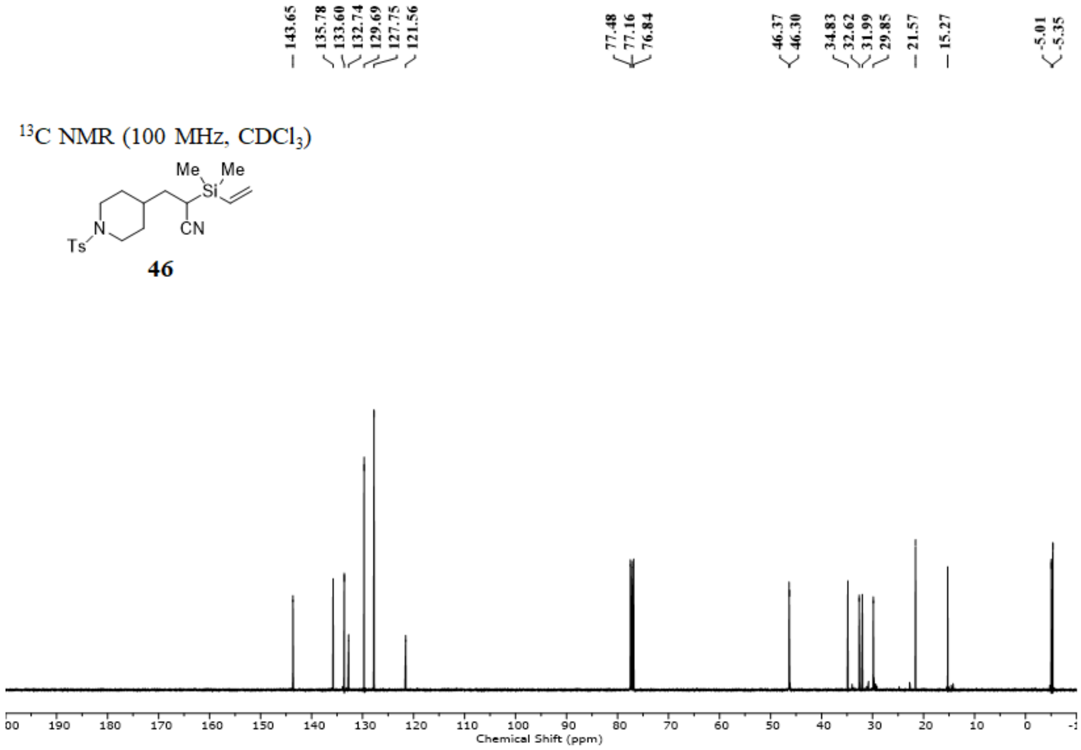

Supplementary Fig. 179. ^{13}C NMR of compound 46. The sample has been recorded in 100 MHz, $CDCl_3$ at 25 °C

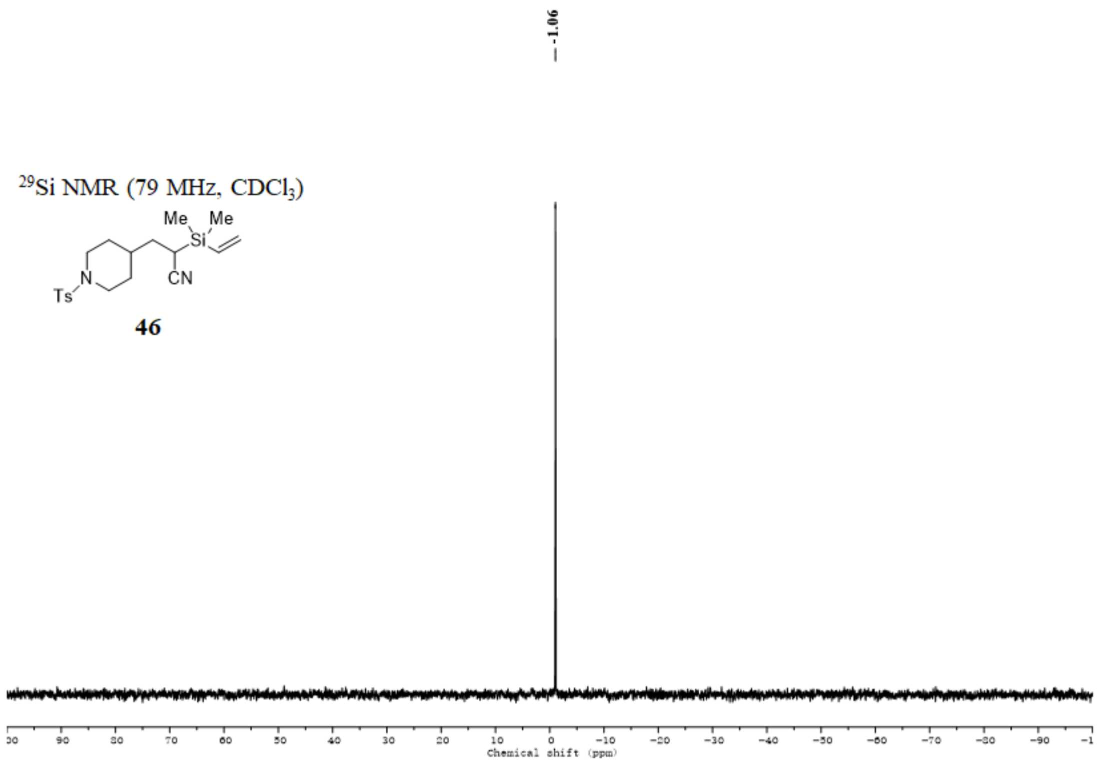

Supplementary Fig. 180. ^{29}Si NMR of compound 46. The sample has been recorded in 79 MHz, $CDCl_3$ at 25 °C

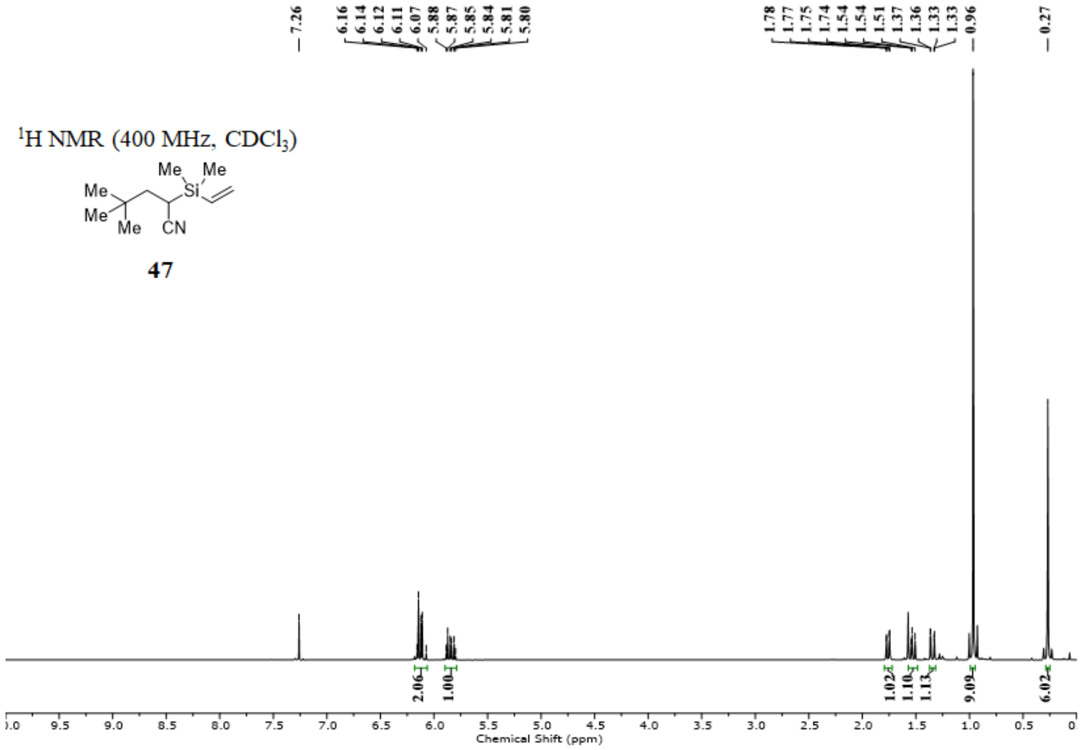

Supplementary Fig. 181. ^{1}H NMR of compound 47. The sample has been recorded in 400 MHz, $CDCl_3$ at 25 °C

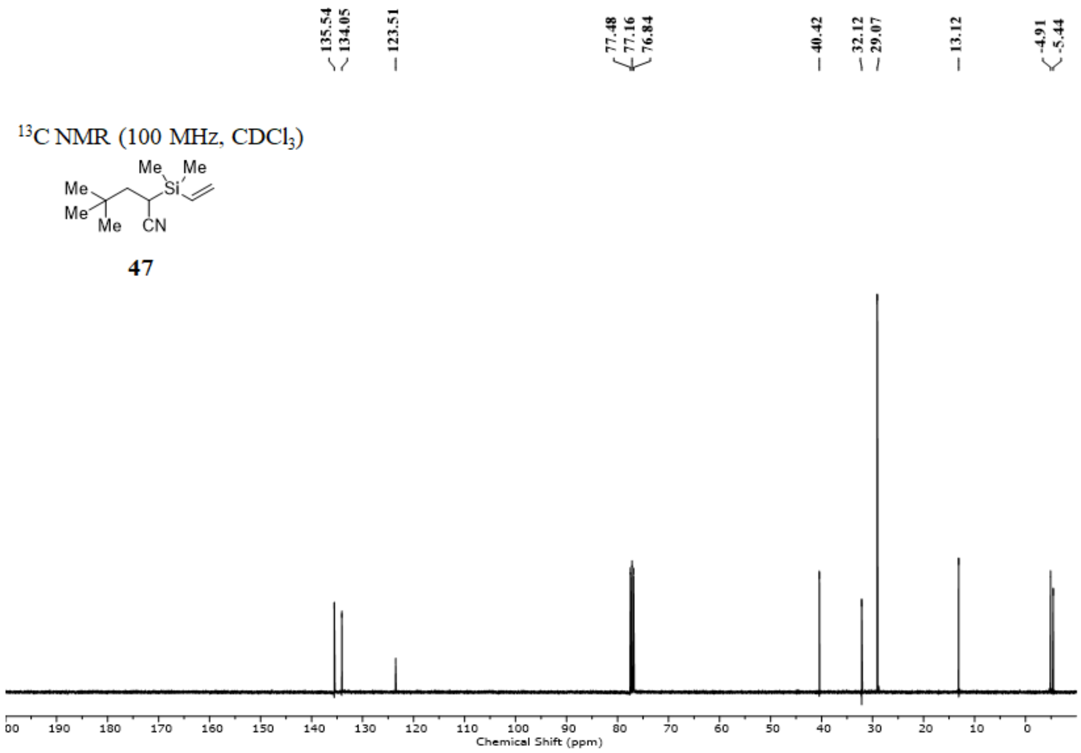

Supplementary Fig. 182. ^{13}C NMR of compound 47. The sample has been recorded in 100 MHz, $CDCl_3$ at 25 °C

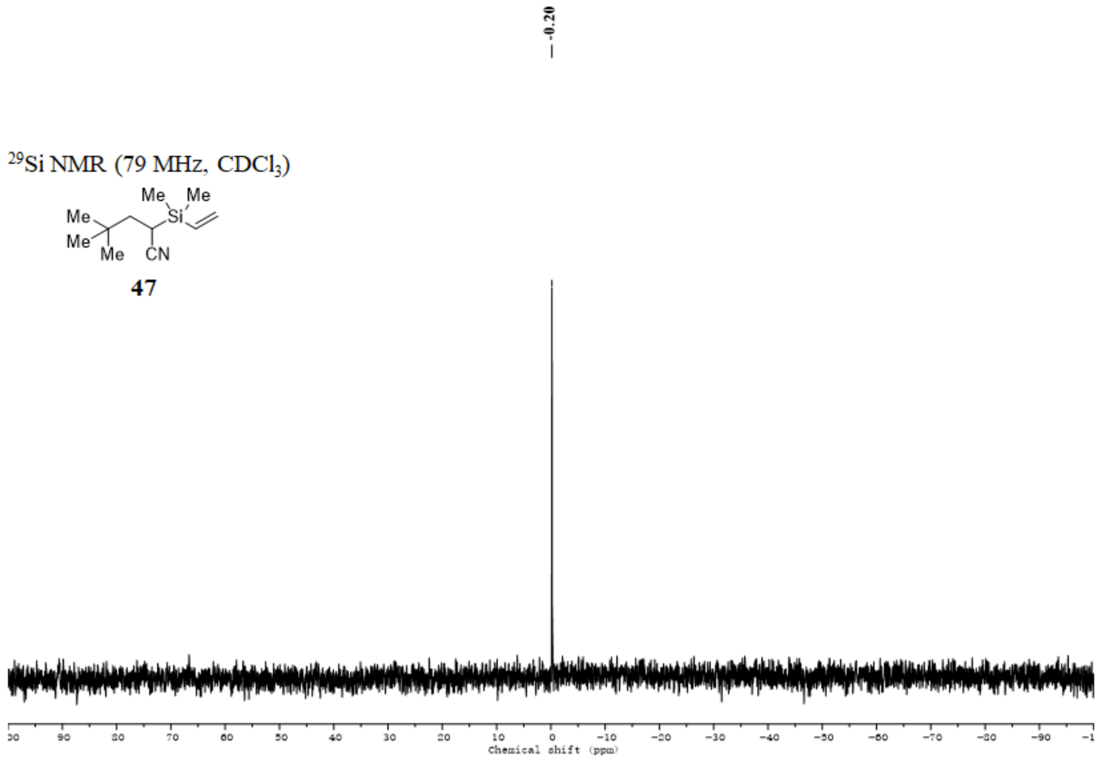

Supplementary Fig. 183. ^{29}Si NMR of compound 47. The sample has been recorded in 79 MHz, $CDCl_3$ at 25 °C

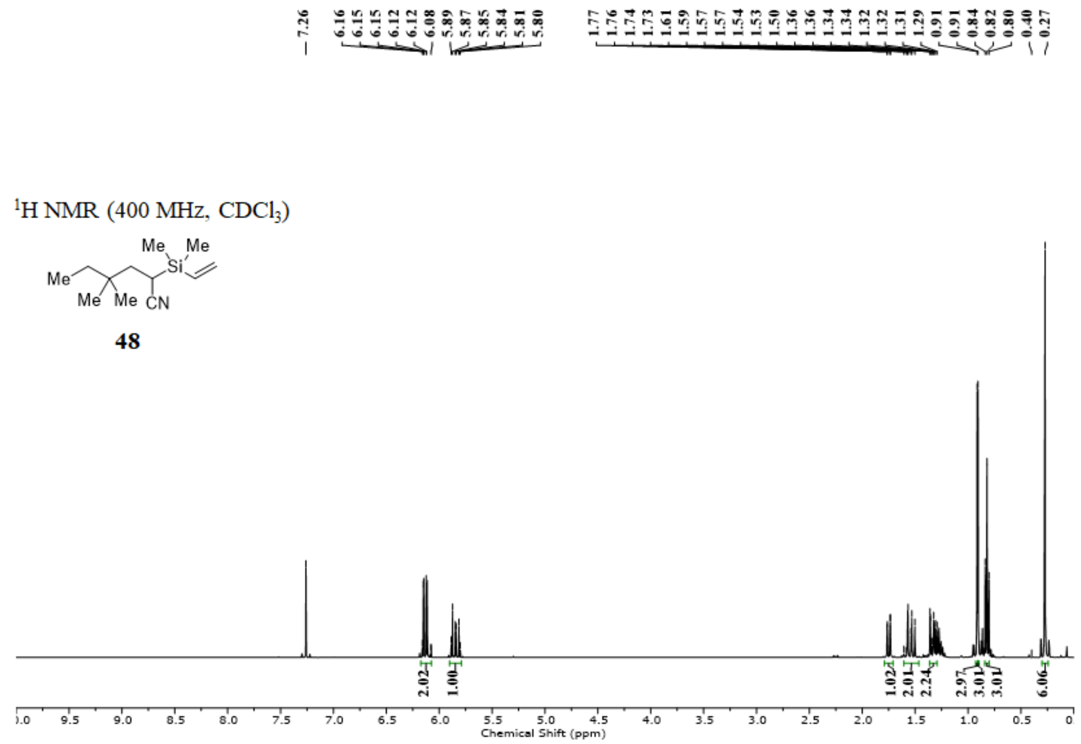

Supplementary Fig. 184. ^{1}H NMR of compound 48. The sample has been recorded in 400 MHz, $CDCl_3$ at 25 °C

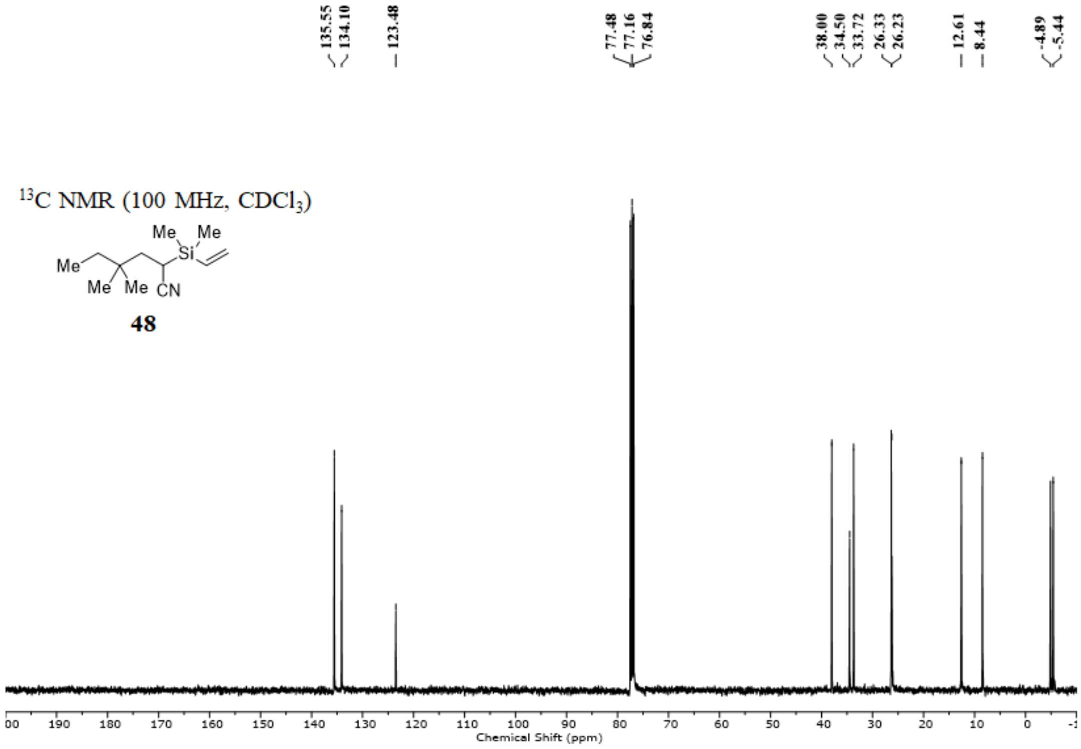

Supplementary Fig. 185. ^{13}C NMR of compound 48. The sample has been recorded in 100 MHz, $CDCl_3$ at 25 °C

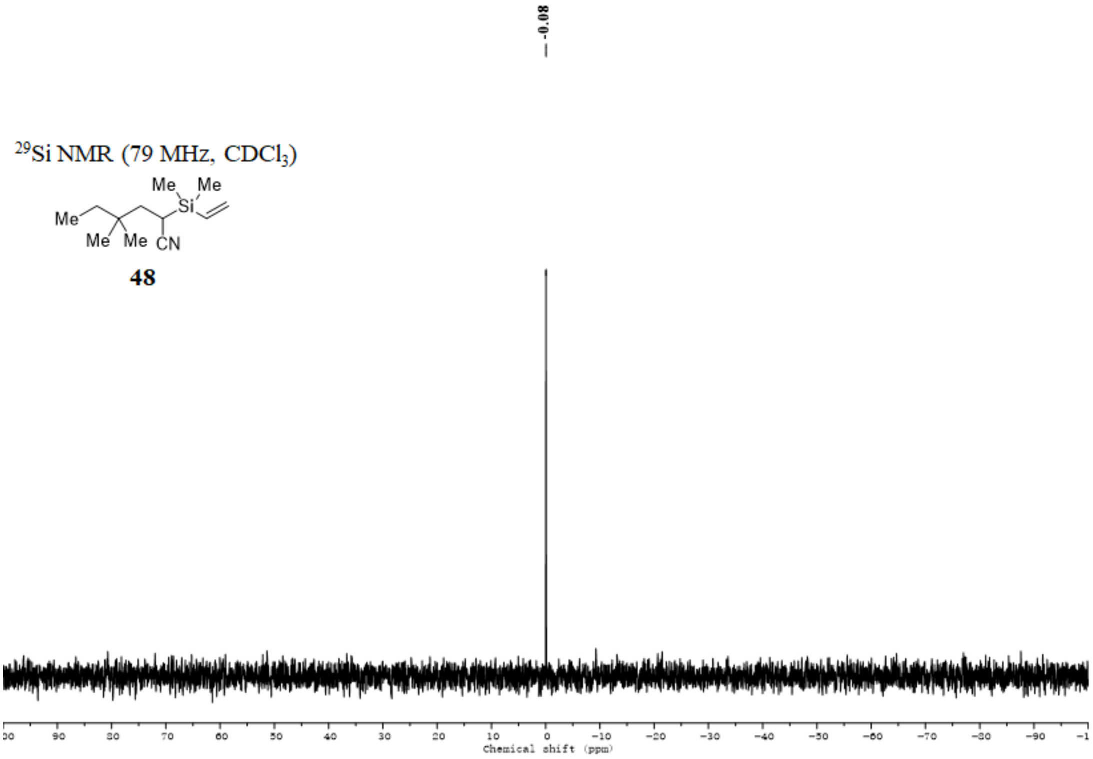

Supplementary Fig. 186. ^{29}Si NMR of compound 48. The sample has been recorded in 79 MHz, $CDCl_3$ at 25 °C

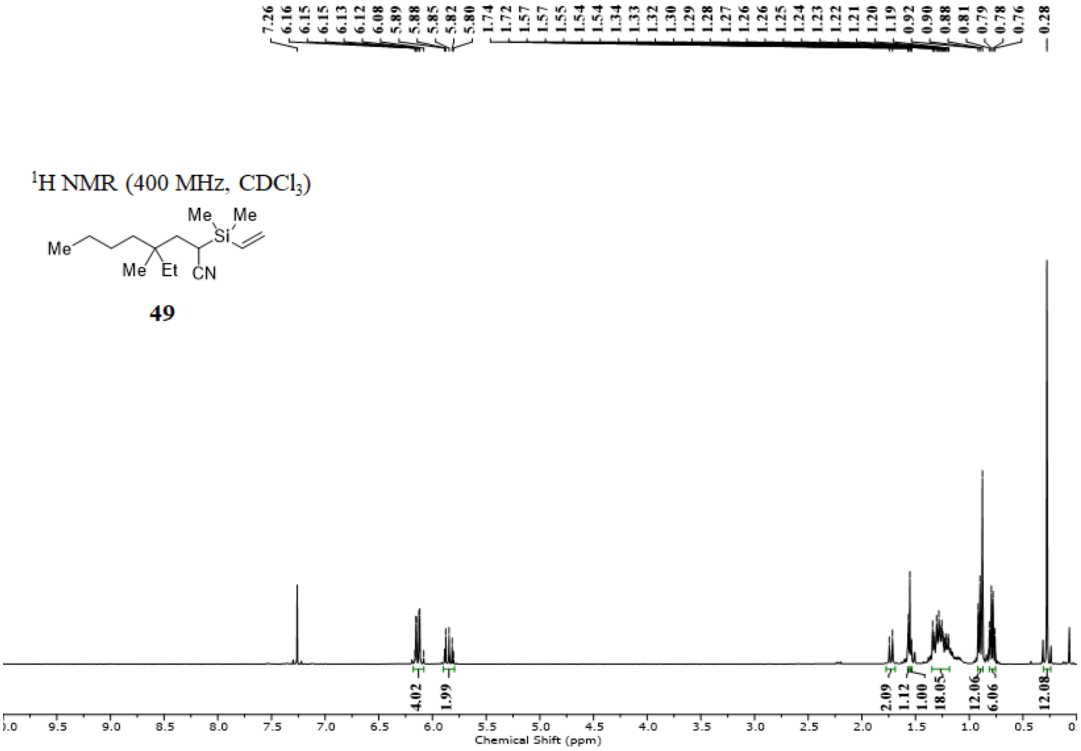

Supplementary Fig. 187. ^{1}H NMR of compound 49. The sample has been recorded in 400 MHz, $CDCl_3$ at 25 °C

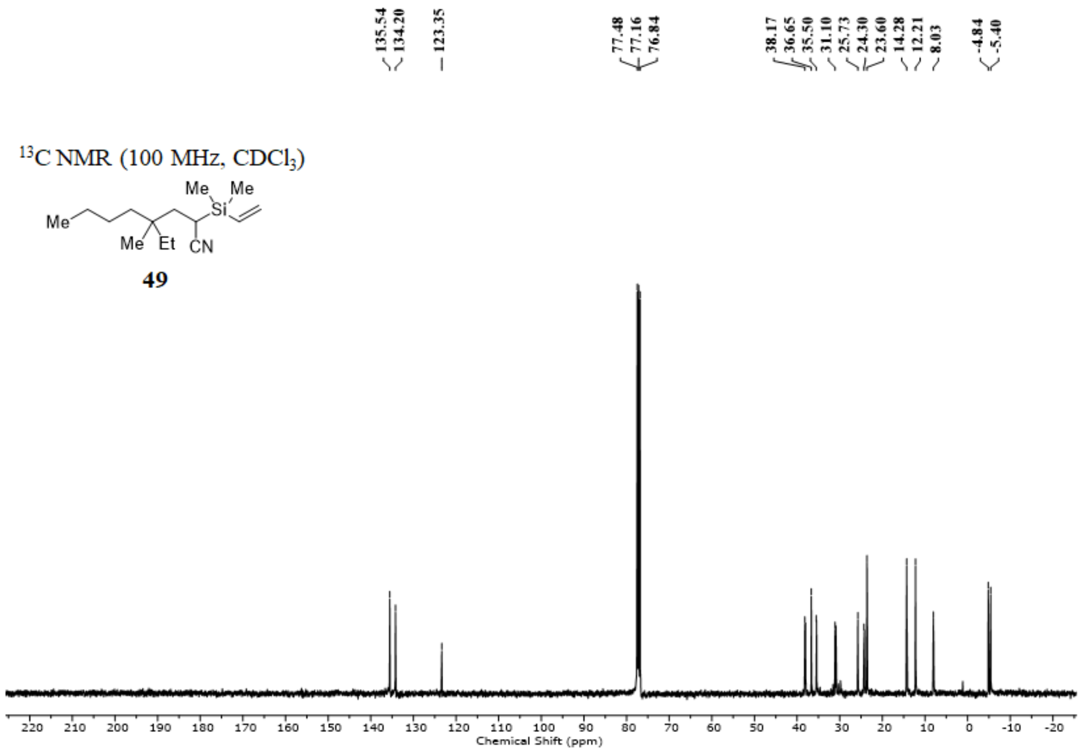

Supplementary Fig. 188. ^{13}C NMR of compound 49. The sample has been recorded in 100 MHz, $CDCl_3$ at 25 °C

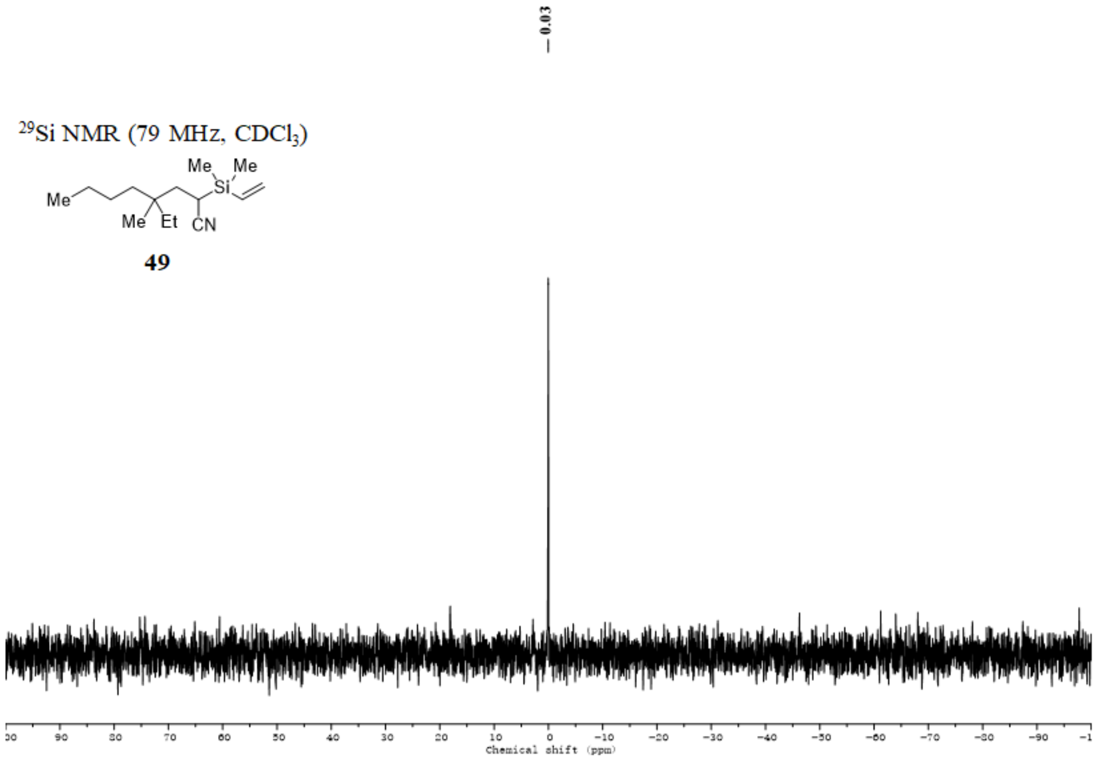

Supplementary Fig. 189. ^{29}Si NMR of compound 49. The sample has been recorded in 79 MHz, $CDCl_3$ at 25 °C

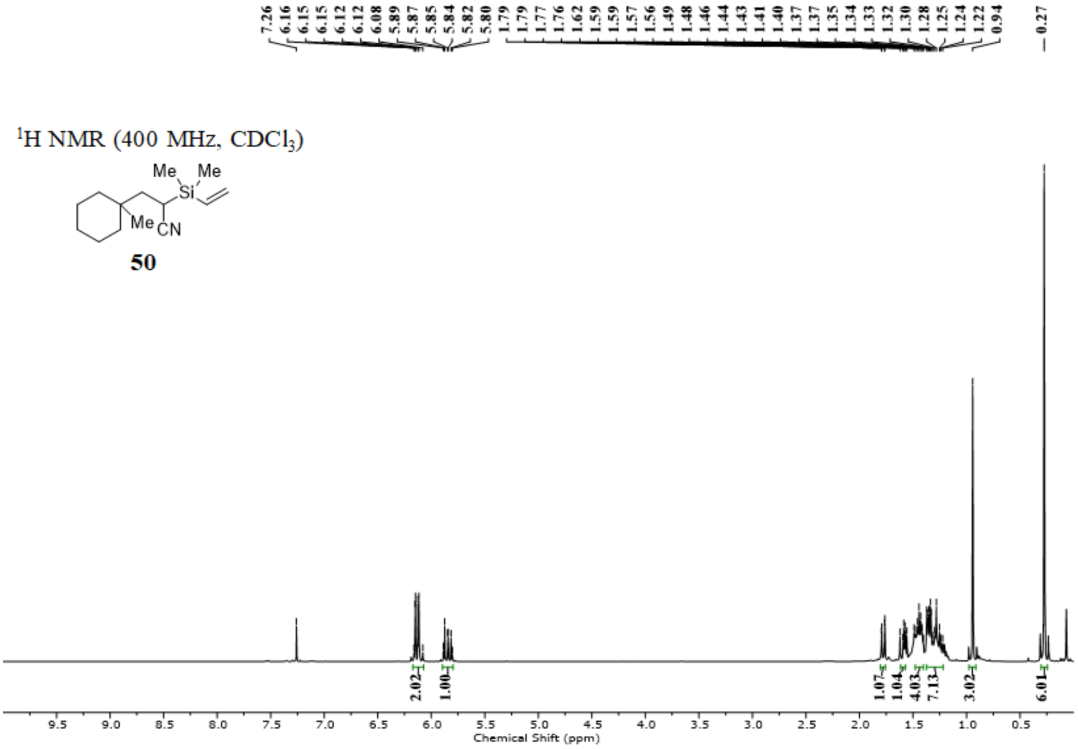

Supplementary Fig. 190. ^{1}H NMR of compound 50. The sample has been recorded in 400 MHz, $CDCl_3$ at 25 °C

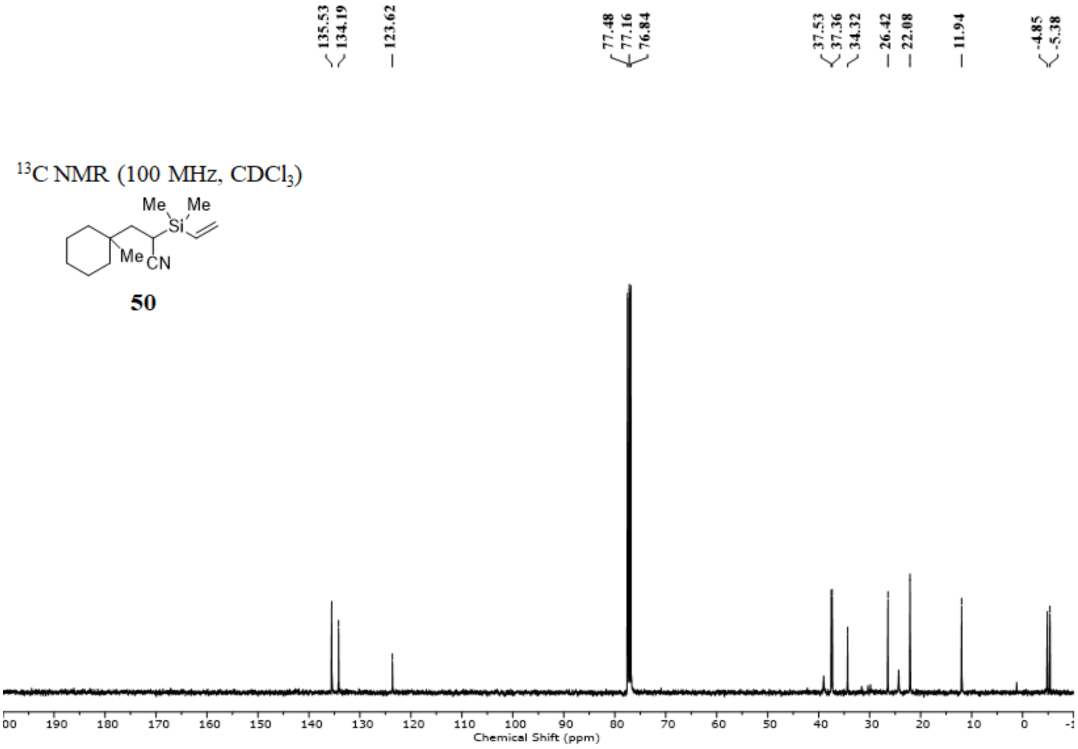

Supplementary Fig. 191. ^{13}C NMR of compound 50. The sample has been recorded in 100 MHz, $CDCl_3$ at 25 °C

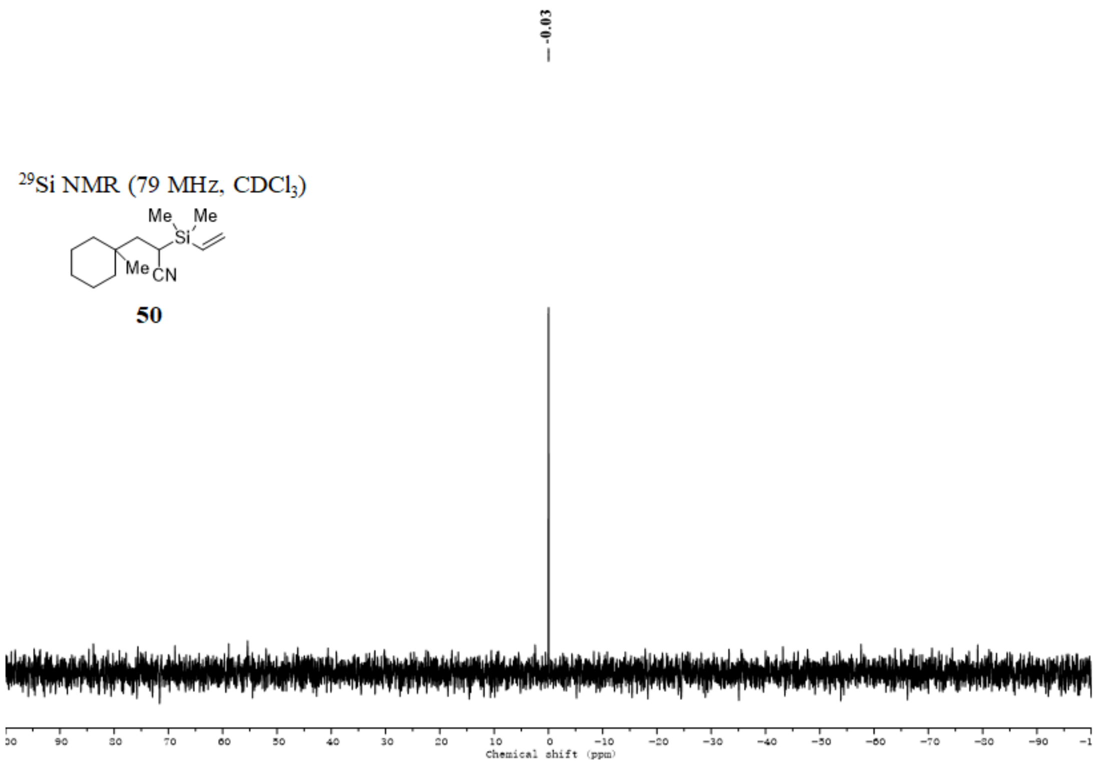

Supplementary Fig. 192. ^{29}Si NMR of compound 50. The sample has been recorded in 79 MHz, $CDCl_3$ at 25 °C

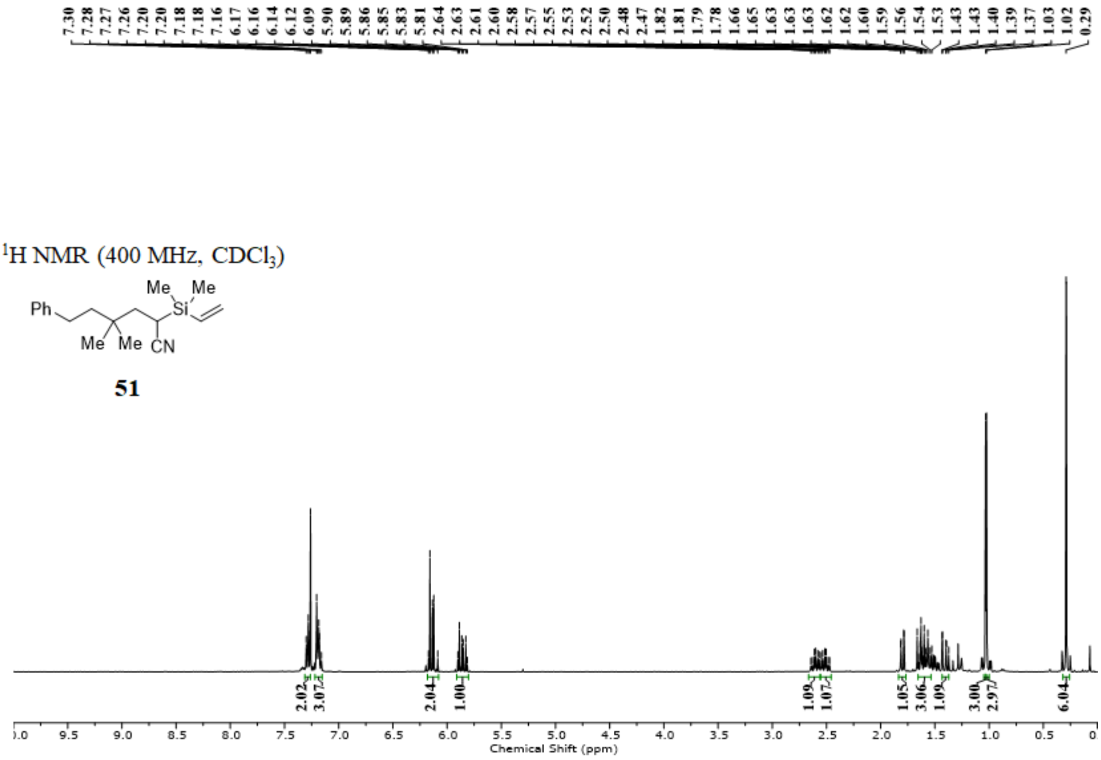

Supplementary Fig. 193. ^{1}H NMR of compound 51. The sample has been recorded in 400 MHz, $CDCl_3$ at 25 °C

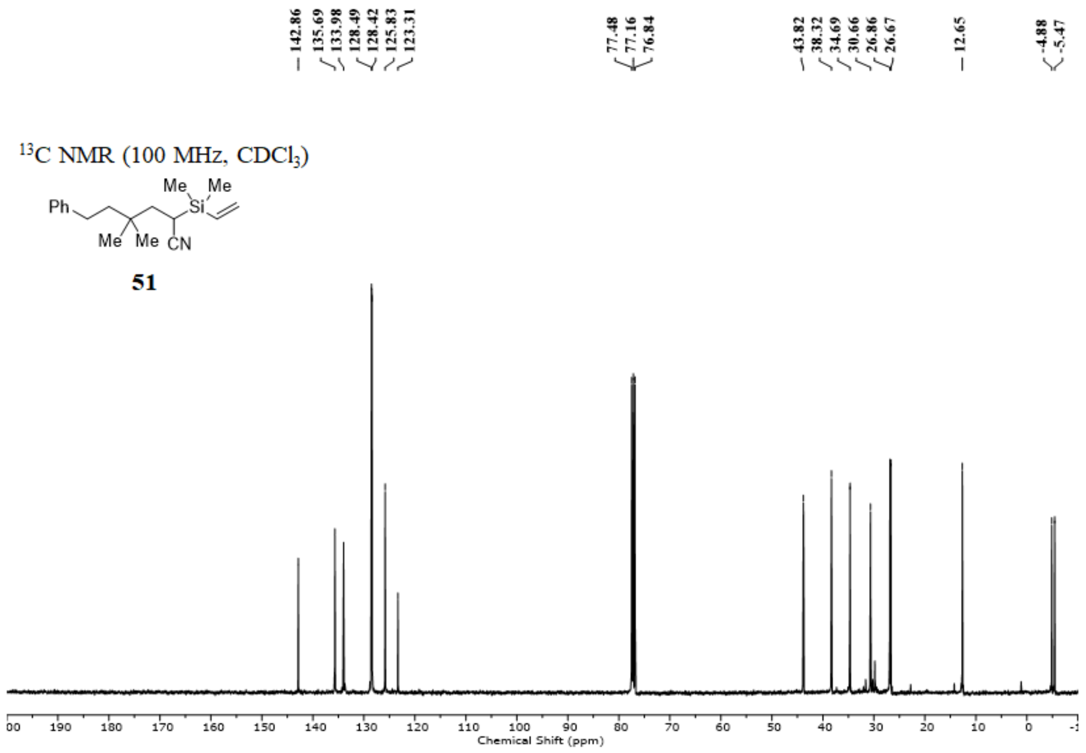

Supplementary Fig. 194. ^{13}C NMR of compound 51. The sample has been recorded in 100 MHz, $CDCl_3$ at 25 °C

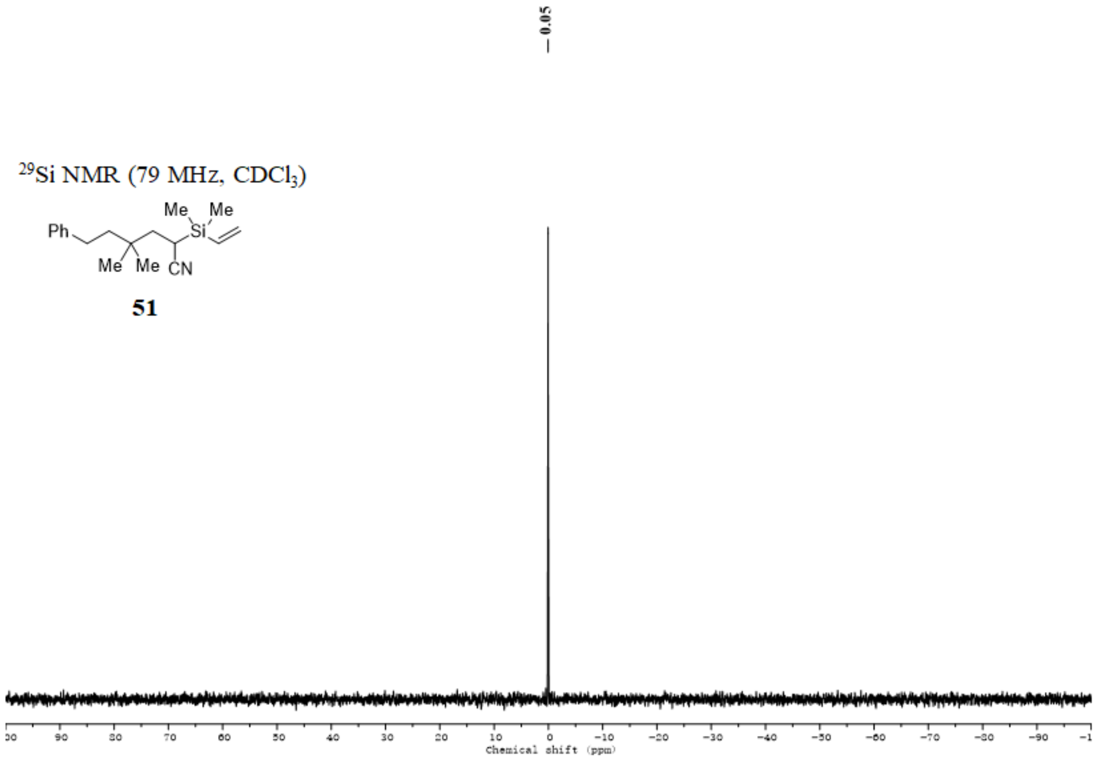

Supplementary Fig. 195. ^{29}Si NMR of compound 51. The sample has been recorded in 79 MHz, $CDCl_3$ at 25 °C

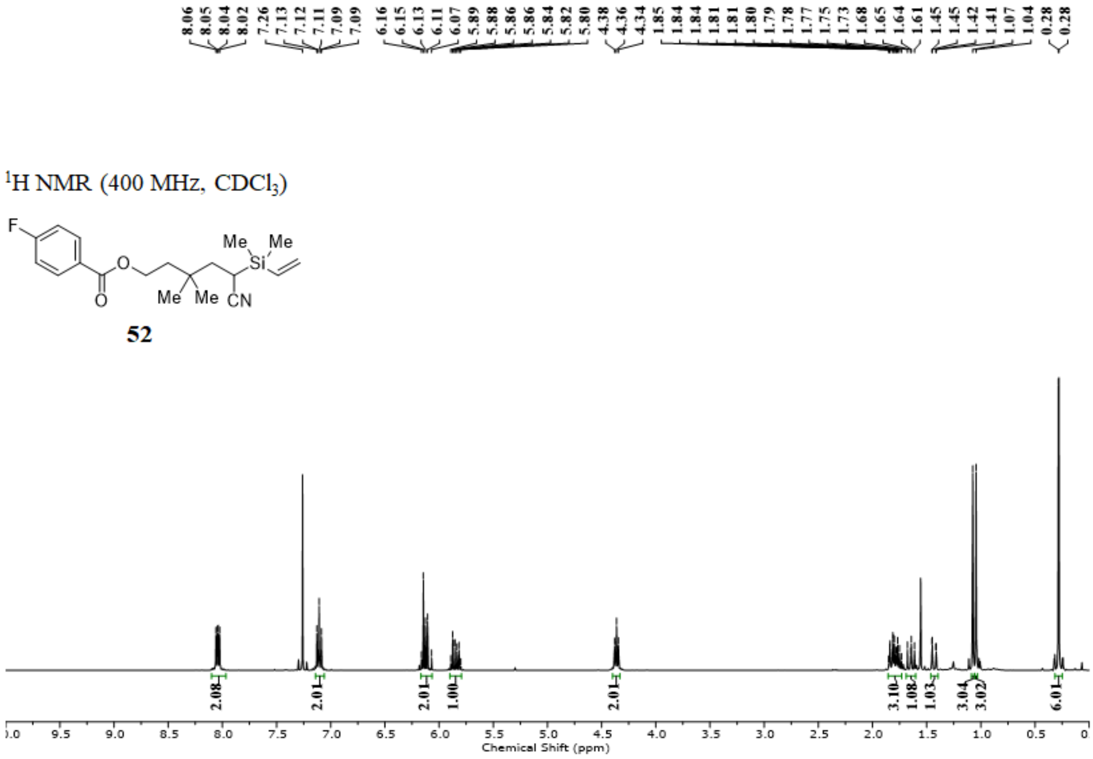

Supplementary Fig. 196. ^{1}H NMR of compound 52. The sample has been recorded in 400 MHz, $CDCl_3$ at 25 °C

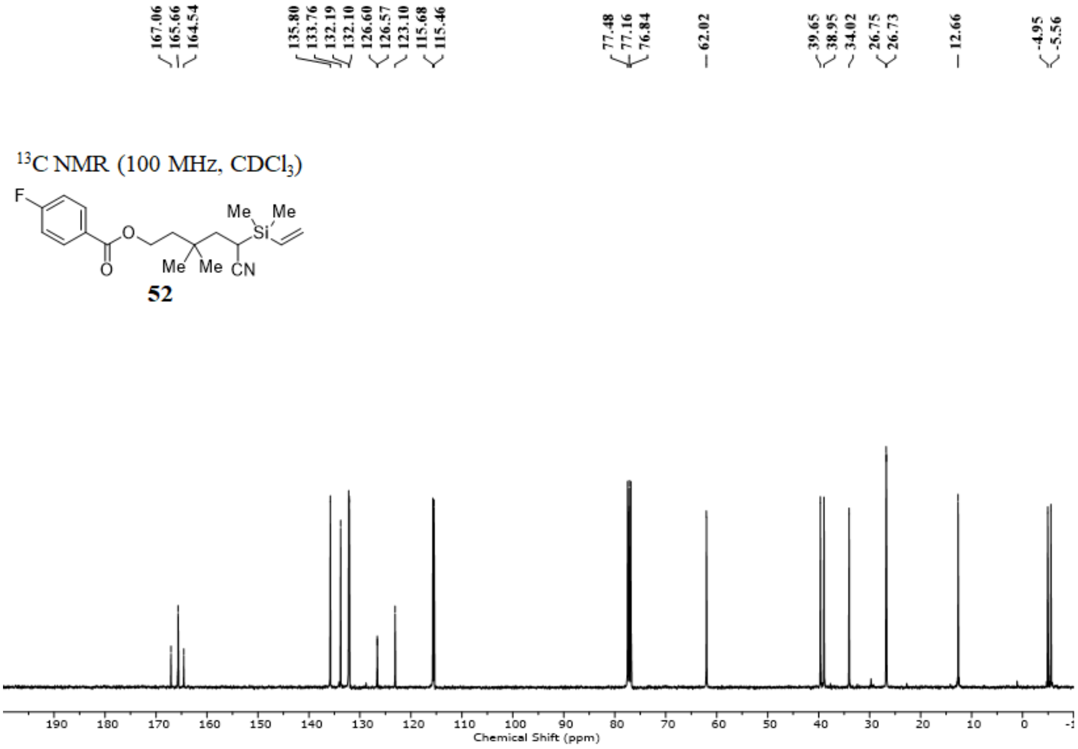

Supplementary Fig. 197. ^{13}C NMR of compound 52. The sample has been recorded in 100 MHz, $CDCl_3$ at 25 °C

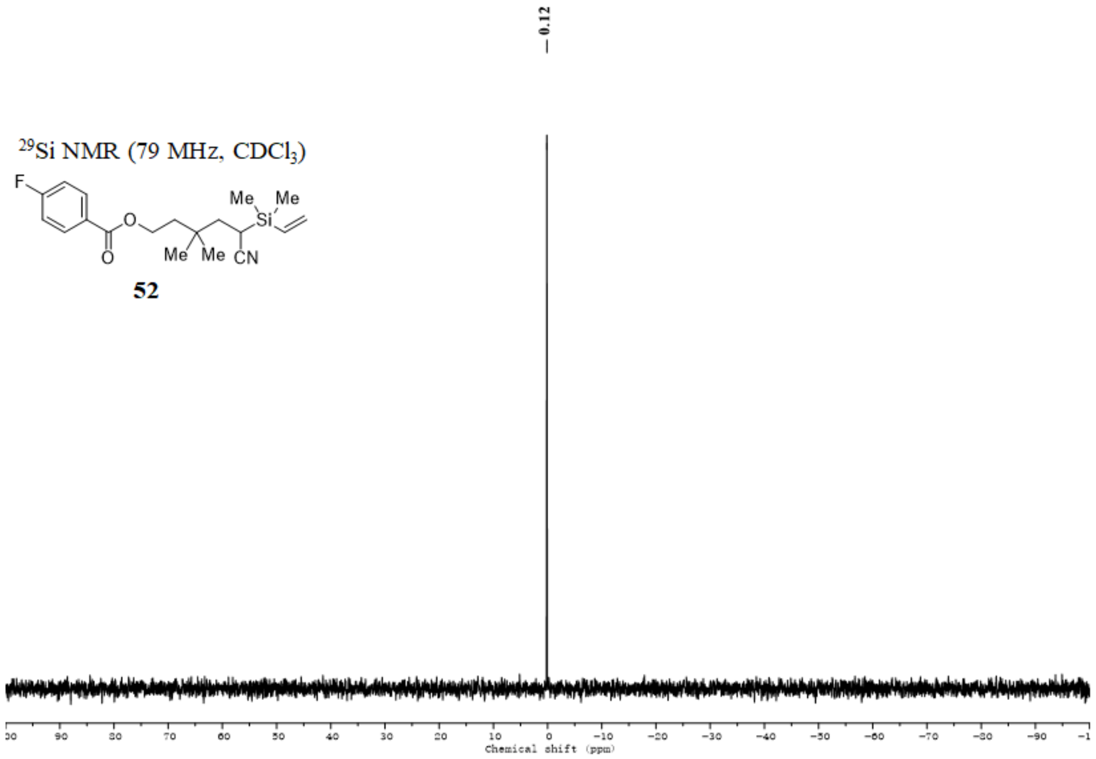

Supplementary Fig. 198. ^{29}Si NMR of compound 52. The sample has been recorded in 79 MHz, $CDCl_3$ at 25 °C

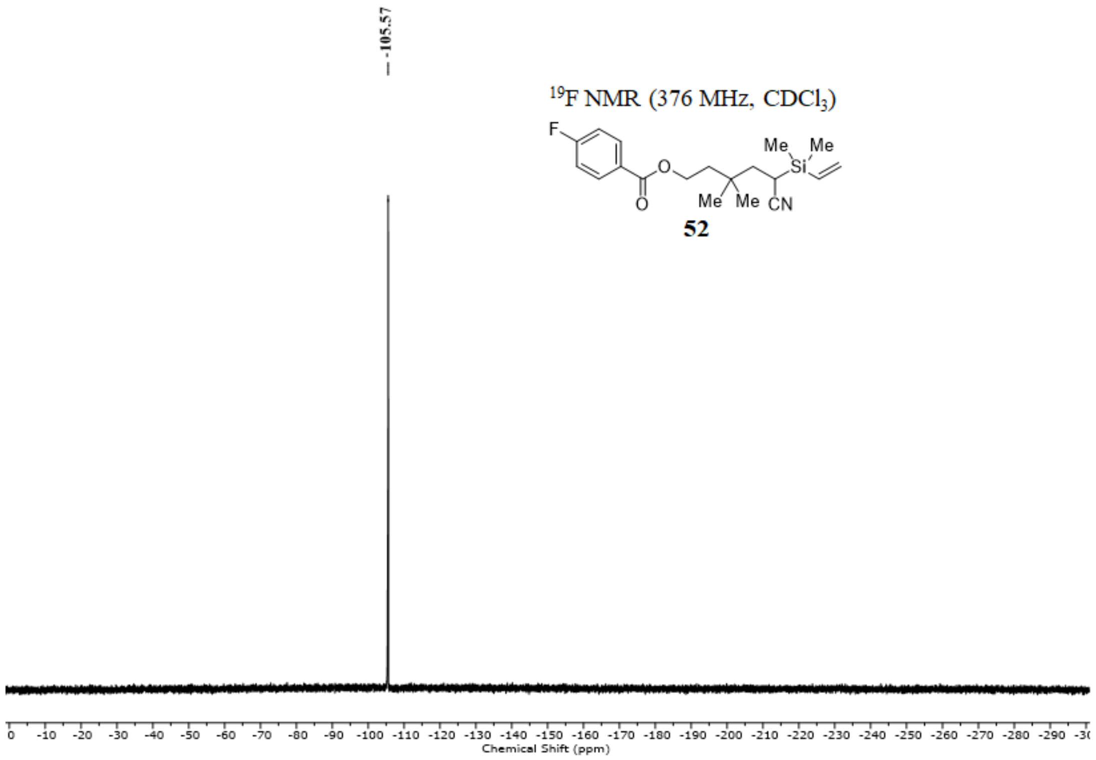

Supplementary Fig. 199. ^{19}F NMR of compound 52. The sample has been recorded in 376 MHz, $CDCl_3$ at 25 °C

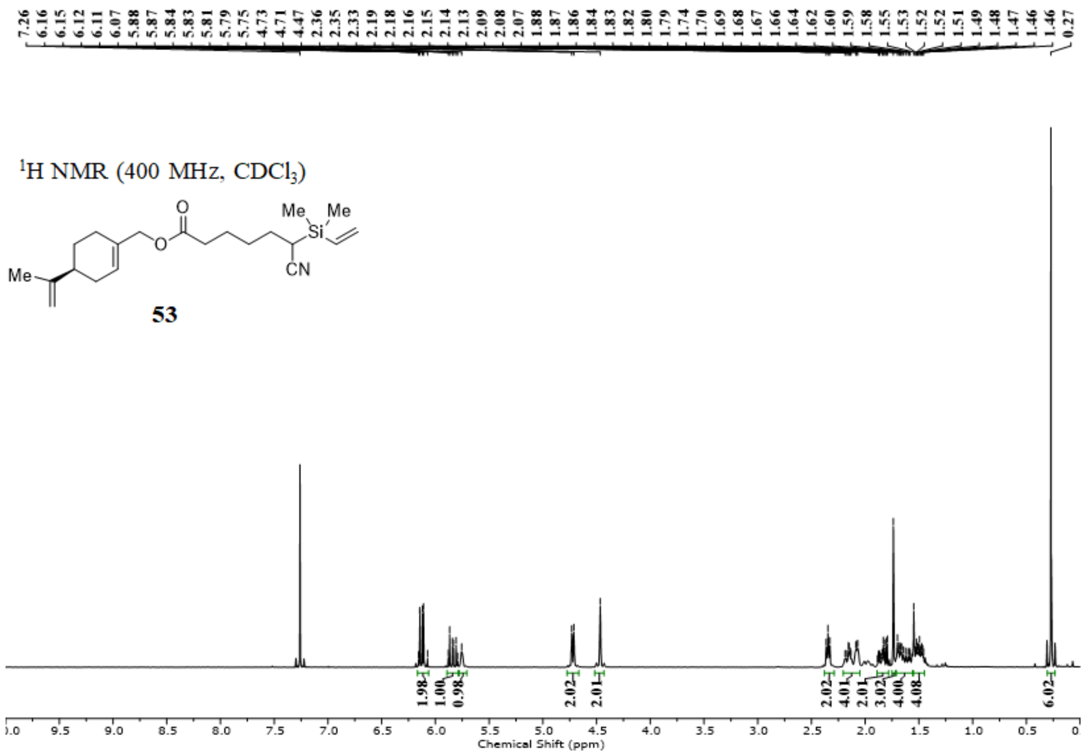

Supplementary Fig. 200. ^{1}**H NMR of compound 53.** The sample has been recorded in 400 MHz, $CDCl_3$ at 25 °C

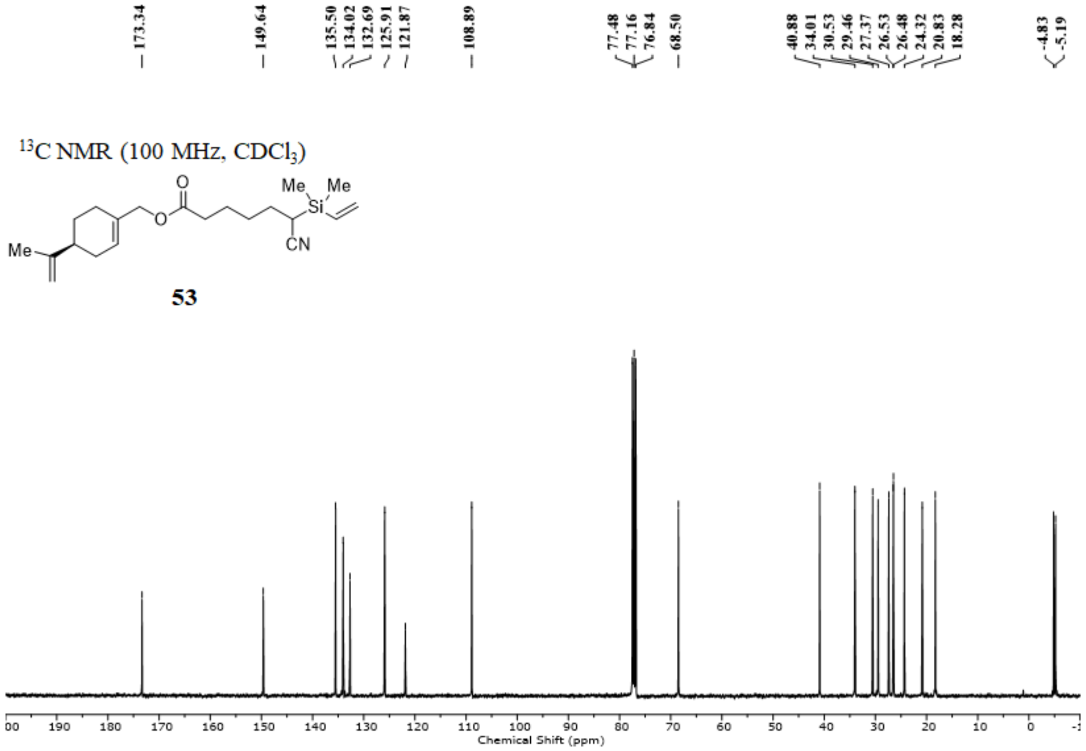

Supplementary Fig. 201. ^{13}**C NMR of compound 53.** The sample has been recorded in 100 MHz, $CDCl_3$ at 25 °C

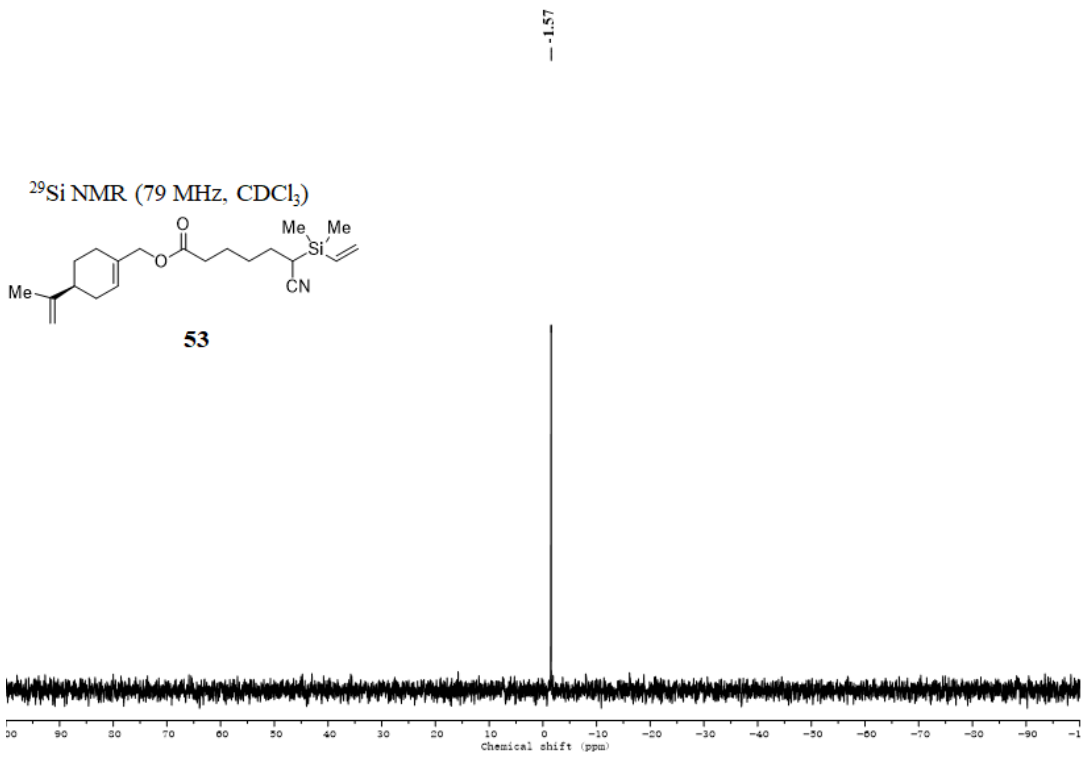

Supplementary Fig. 202. ^{29}Si NMR of compound 53. The sample has been recorded in 79 MHz, $CDCl_3$ at 25 °C

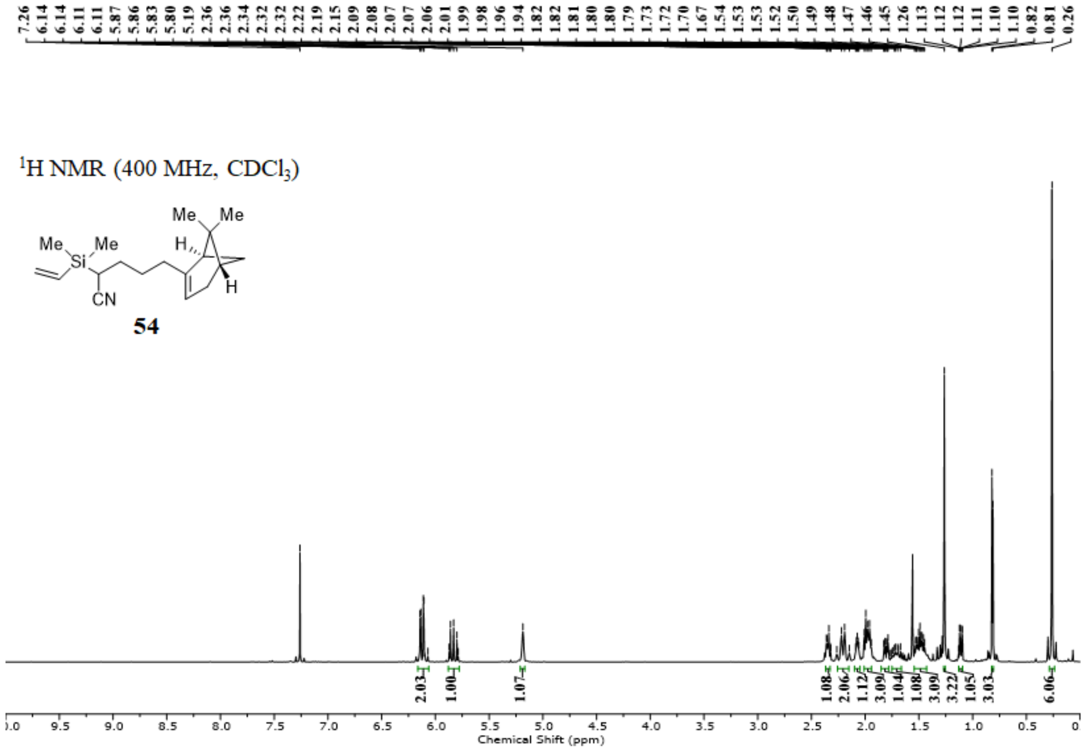

Supplementary Fig. 203. ^{1}H NMR of compound 54. The sample has been recorded in 400 MHz, $CDCl_3$ at 25 °C

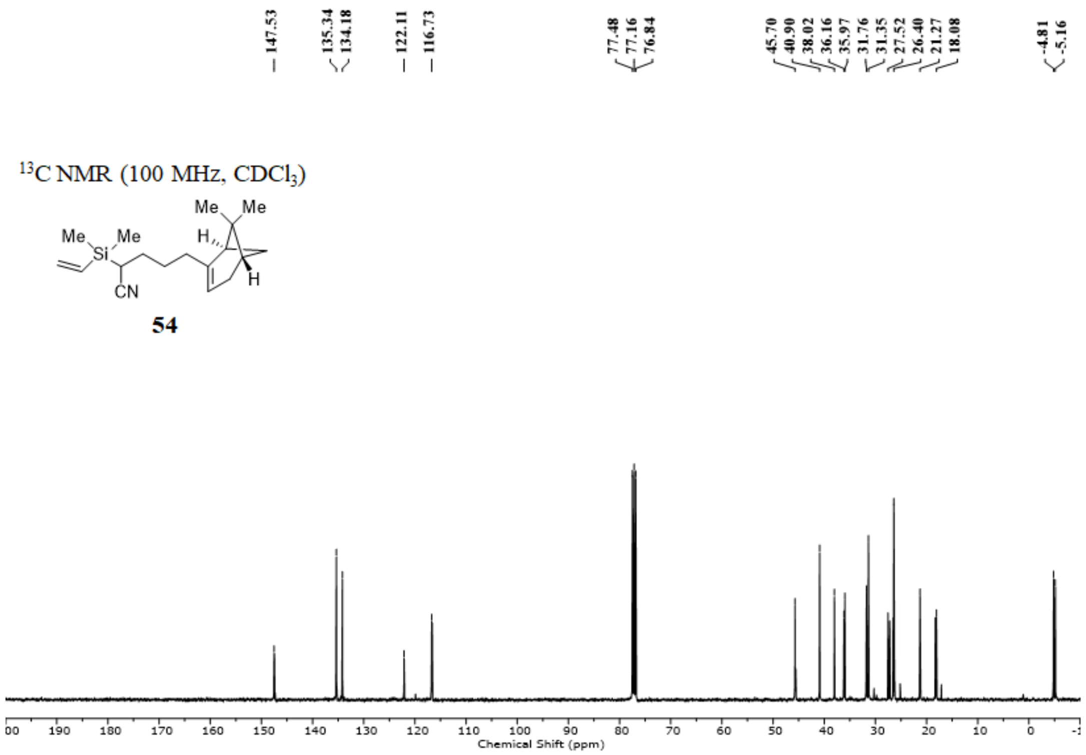

Supplementary Fig. 204. ^{13}C NMR of compound 54. The sample has been recorded in 100 MHz, $CDCl_3$ at 25 °C

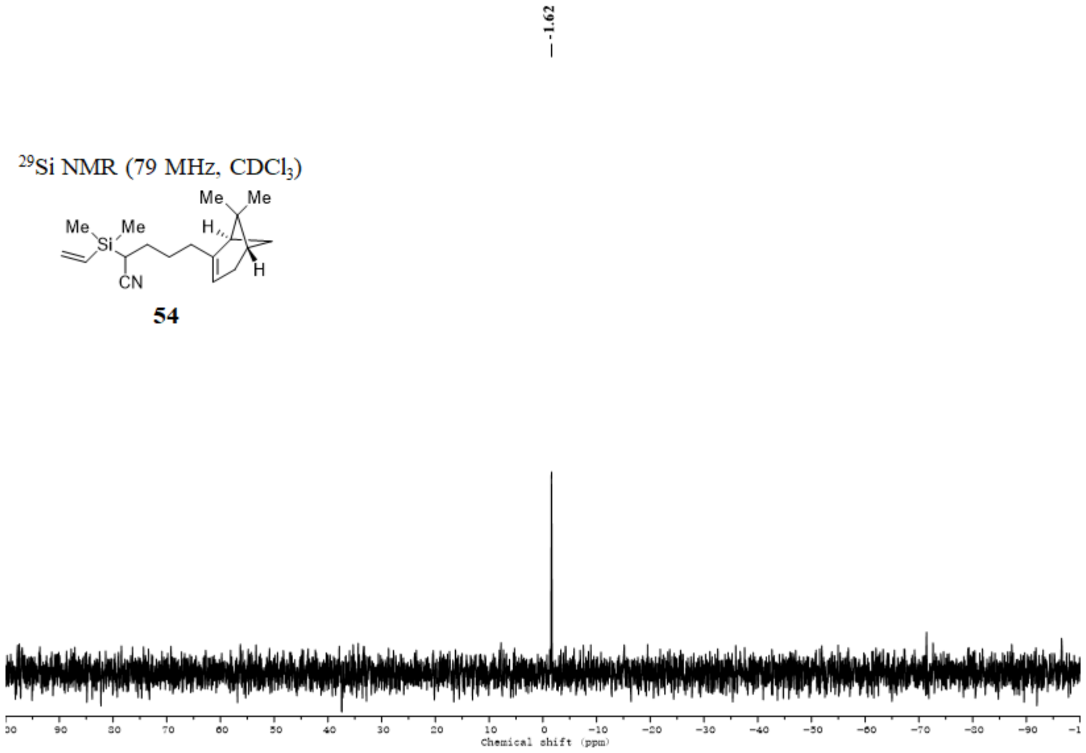

Supplementary Fig. 205. ^{29}Si NMR of compound 54. The sample has been recorded in 79 MHz, $CDCl_3$ at 25 °C

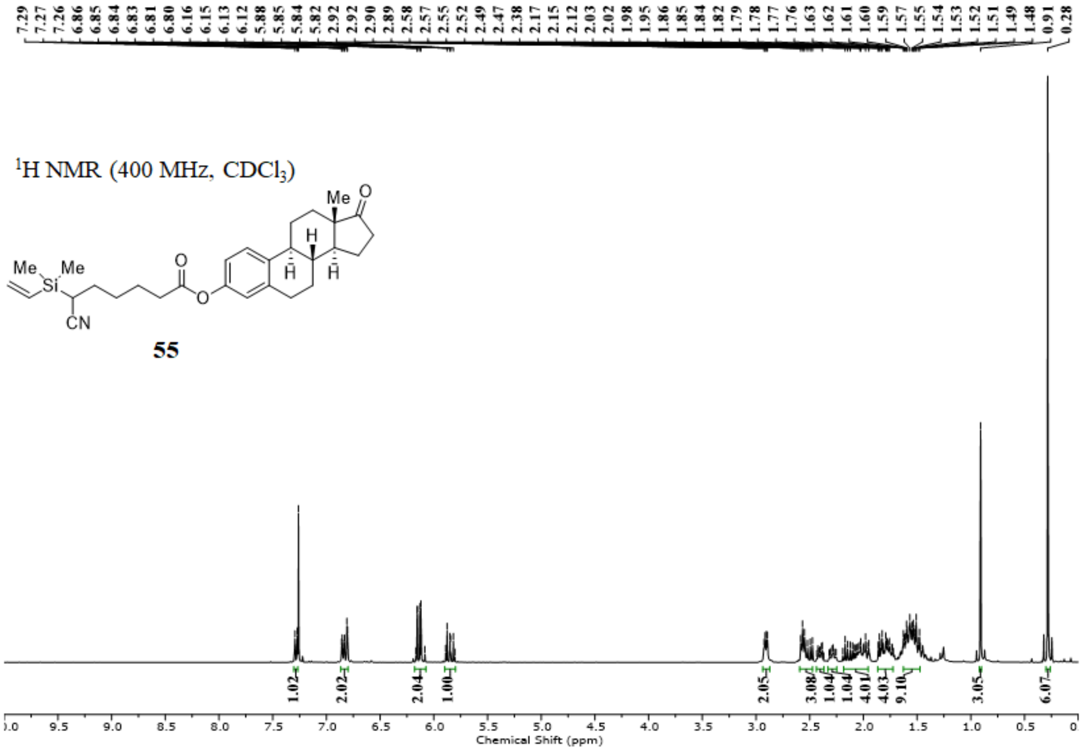

Supplementary Fig. 206. ^{1}H NMR of compound 55. The sample has been recorded in 400 MHz, $CDCl_3$ at 25 °C

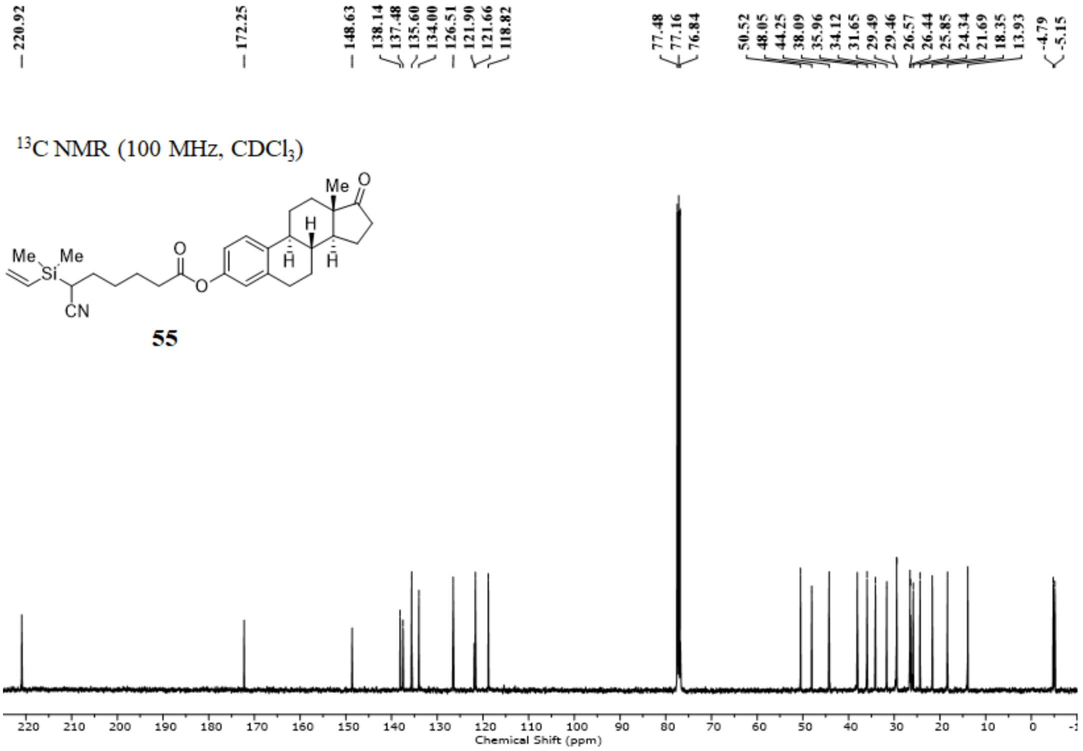

Supplementary Fig. 207. ^{13}C NMR of compound 55. The sample has been recorded in 100 MHz, $CDCl_3$ at 25 °C

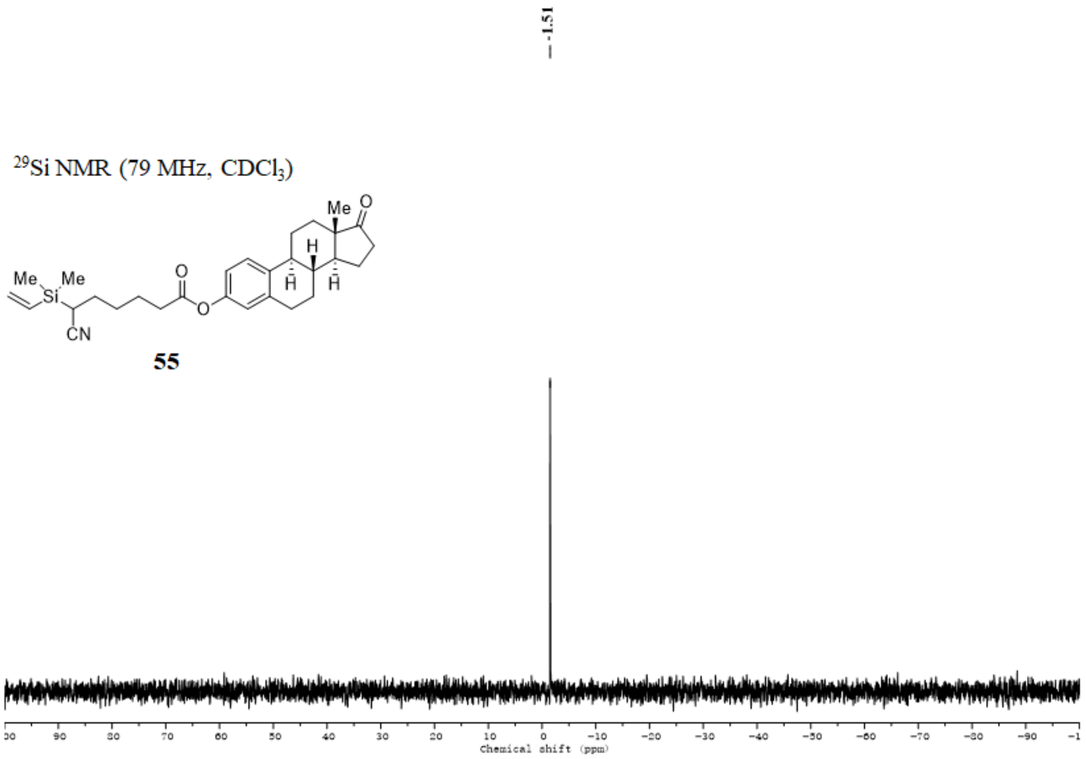

Supplementary Fig. 208. ^{29}Si NMR of compound 55. The sample has been recorded in 79 MHz, $CDCl_3$ at 25 °C

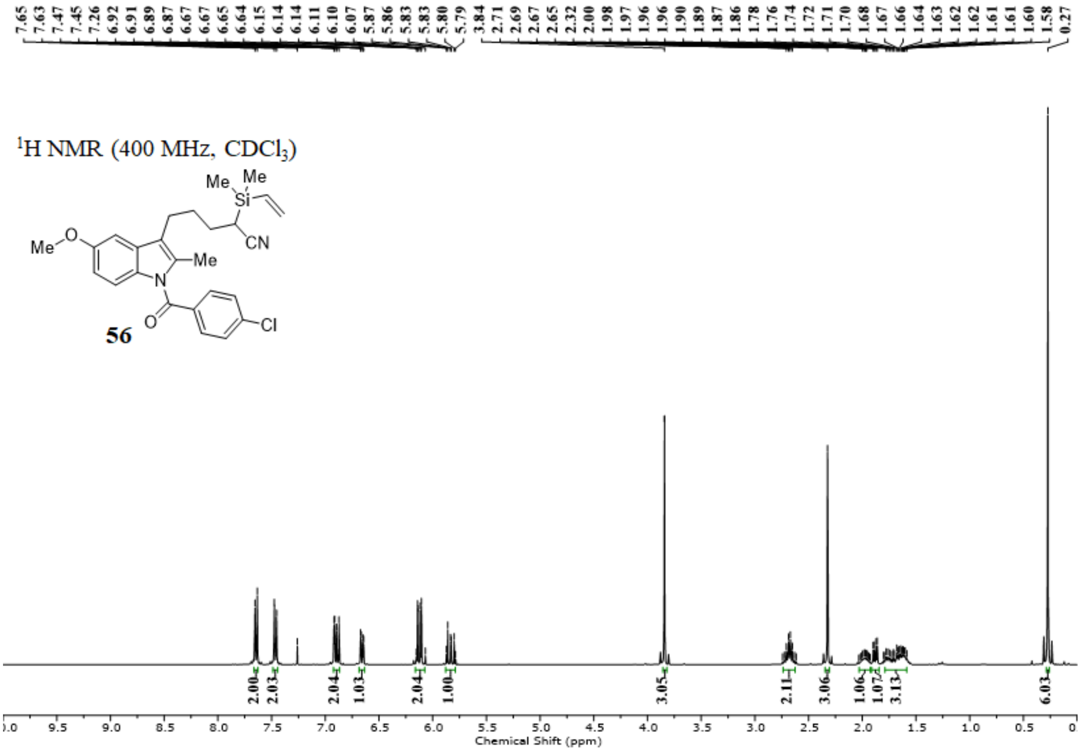

Supplementary Fig. 209. ^{1}H NMR of compound 56. The sample has been recorded in 400 MHz, $CDCl_3$ at 25 °C

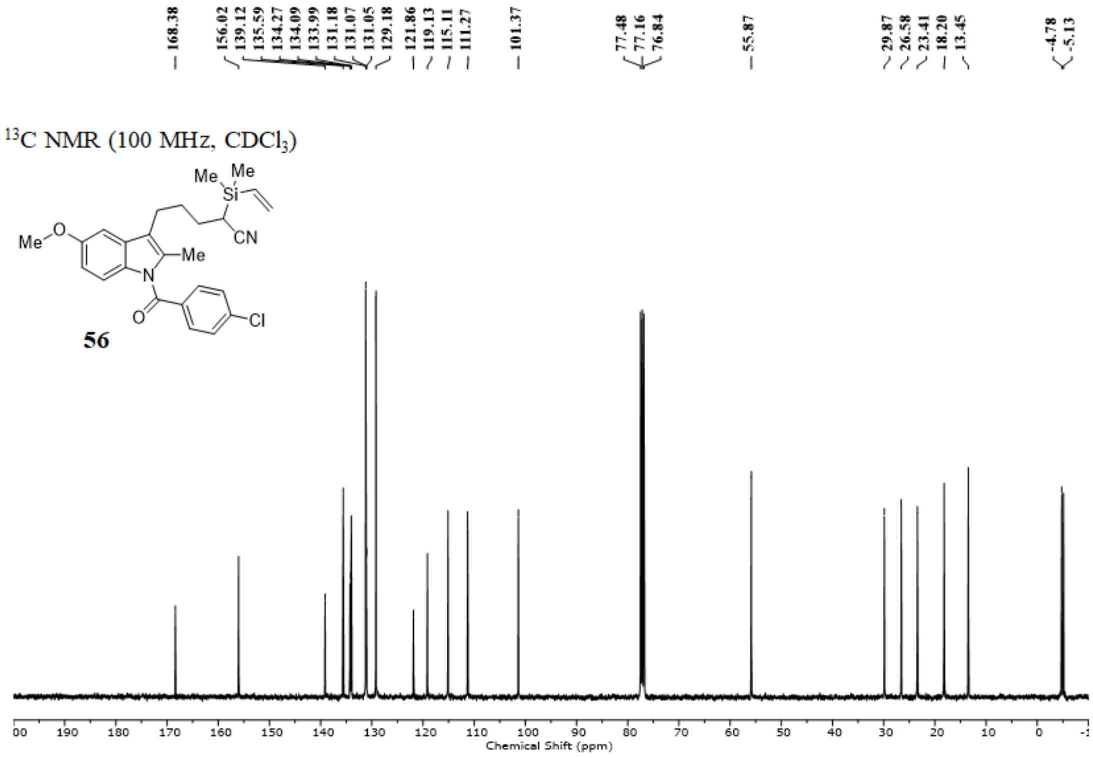

Supplementary Fig. 210. ^{13}C NMR of compound 56. The sample has been recorded in 100 MHz, $CDCl_3$ at 25 °C

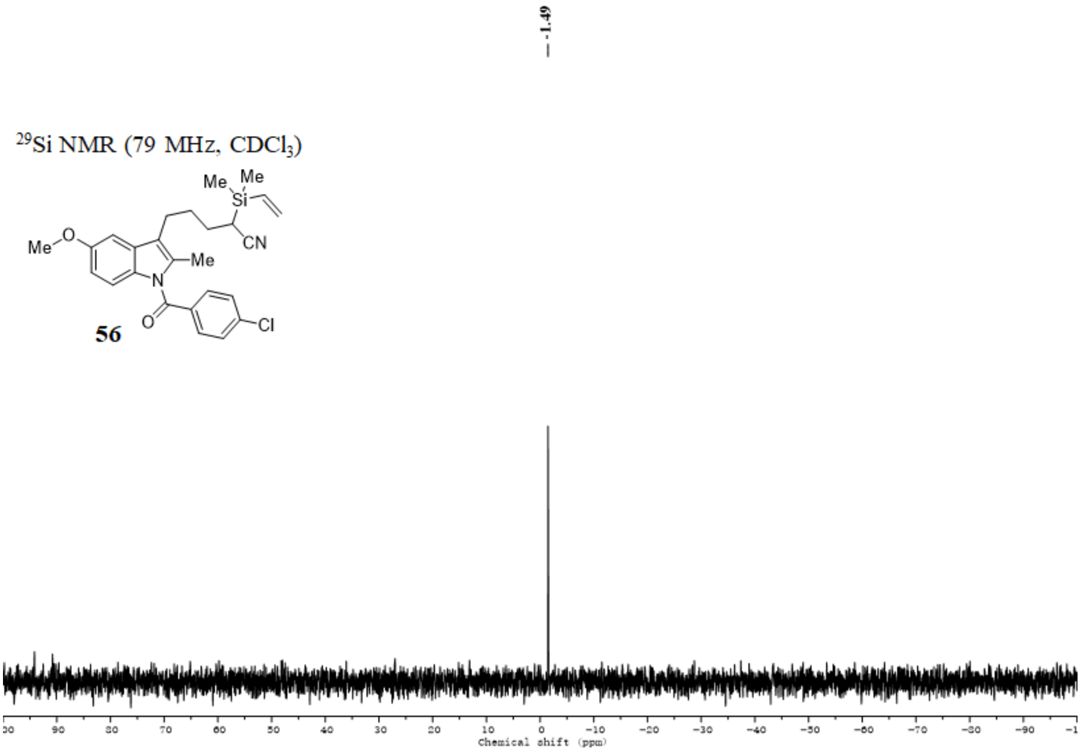

Supplementary Fig. 211. ^{29}Si NMR of compound 56. The sample has been recorded in 79 MHz, $CDCl_3$ at 25 °C

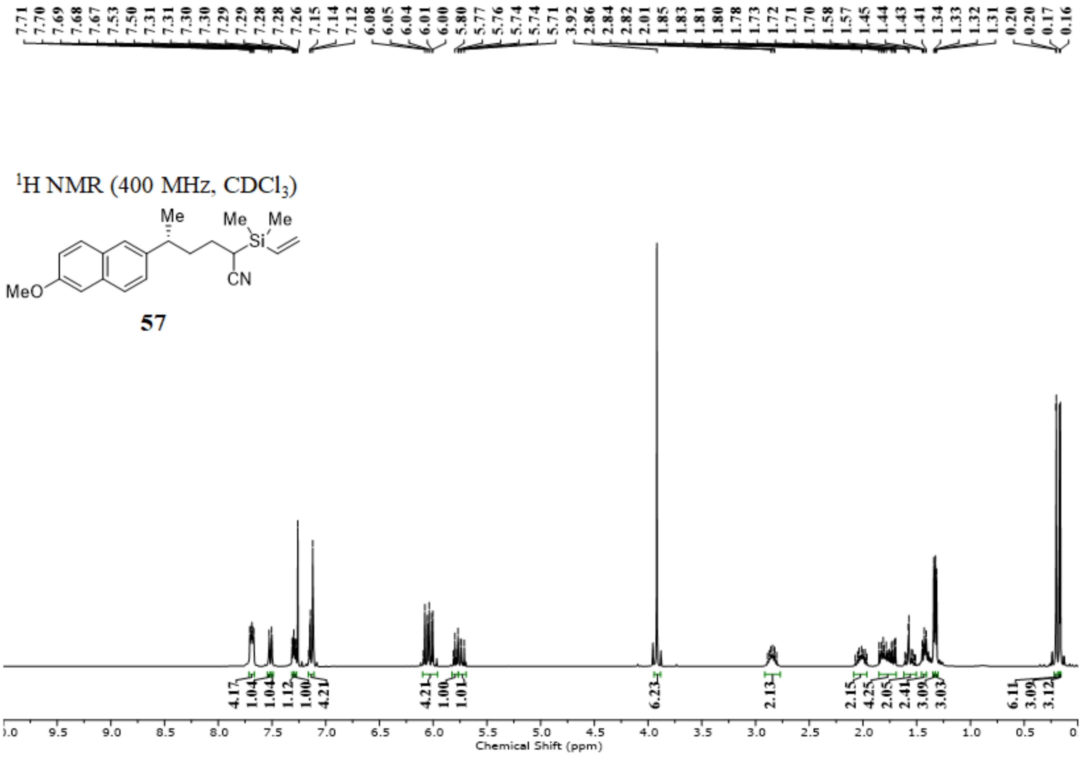

Supplementary Fig. 212. ^{1}H NMR of compound 57. The sample has been recorded in 400 MHz, $CDCl_3$ at 25 °C

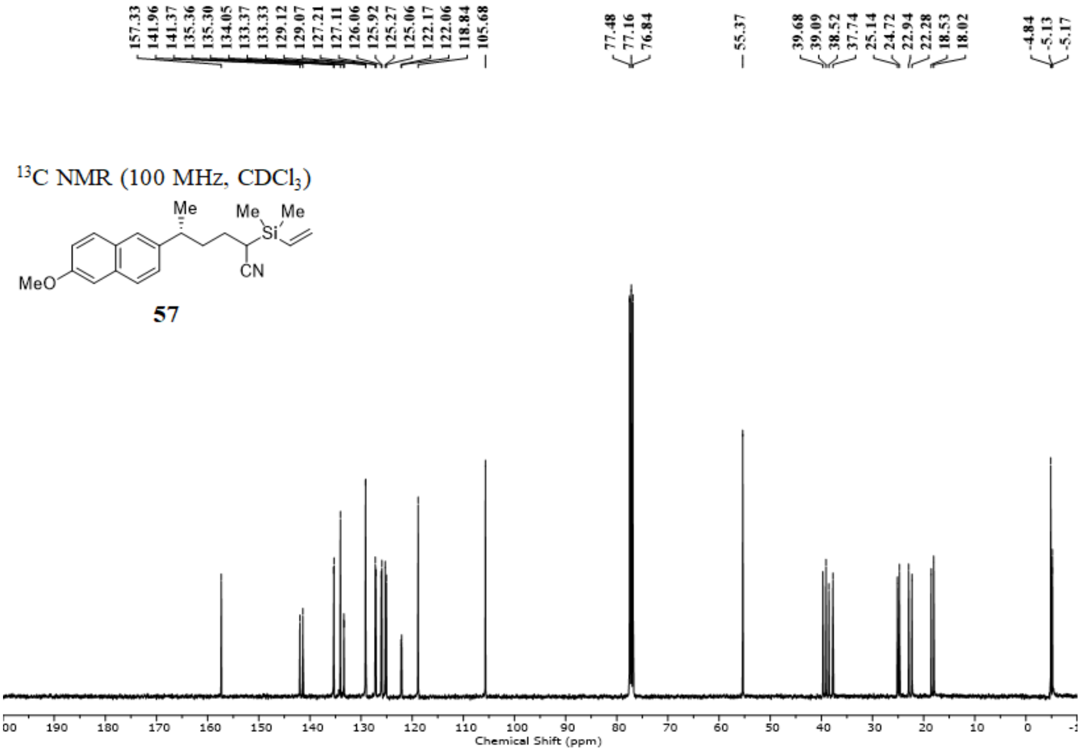

Supplementary Fig. 213. ^{13}C NMR of compound 57. The sample has been recorded in 100 MHz, $CDCl_3$ at 25 °C

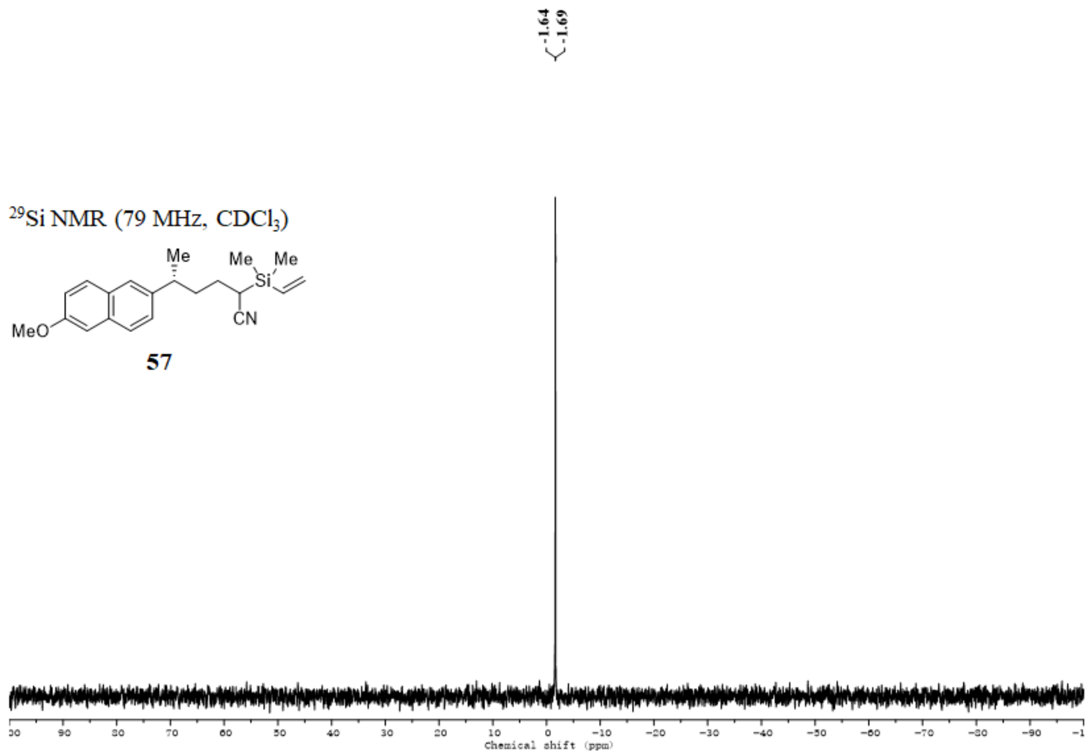

Supplementary Fig. 214. ^{29}Si NMR of compound 57. The sample has been recorded in 79 MHz, $CDCl_3$ at 25 °C

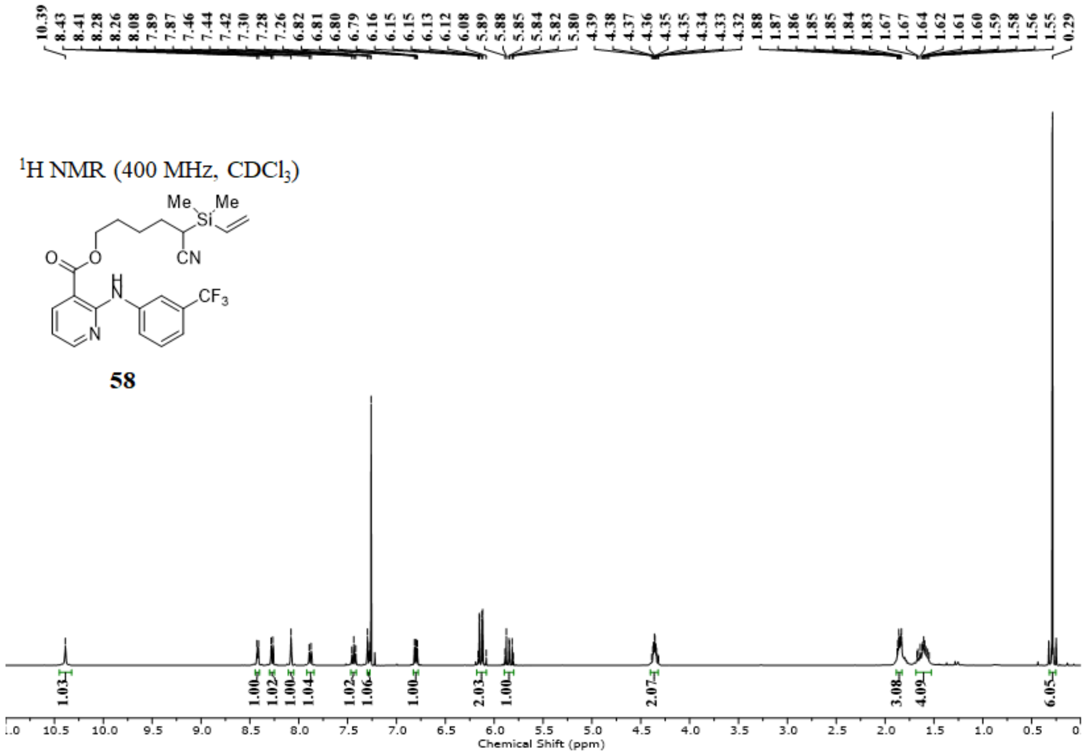

Supplementary Fig. 215. ^{1}H NMR of compound 58. The sample has been recorded in 400 MHz, $CDCl_3$ at 25 °C

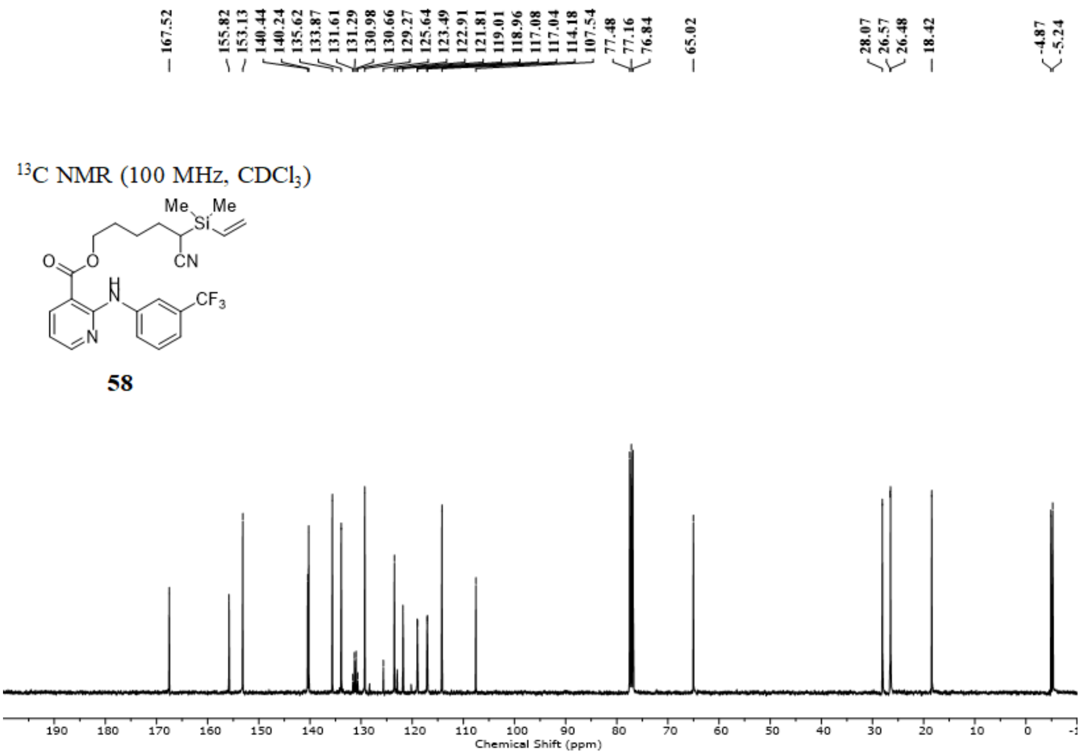

Supplementary Fig. 216. ^{13}C NMR of compound 58. The sample has been recorded in 100 MHz, $CDCl_3$ at 25 °C

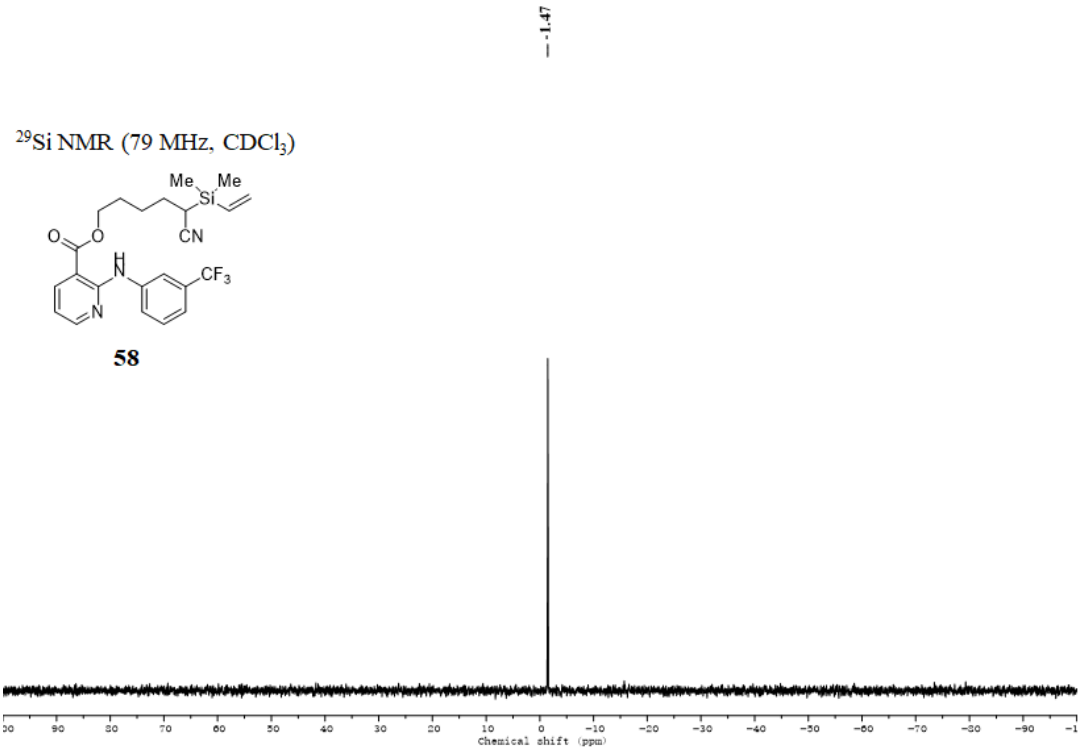

Supplementary Fig. 217. ^{29}Si NMR of compound 58. The sample has been recorded in 79 MHz, $CDCl_3$ at 25 °C

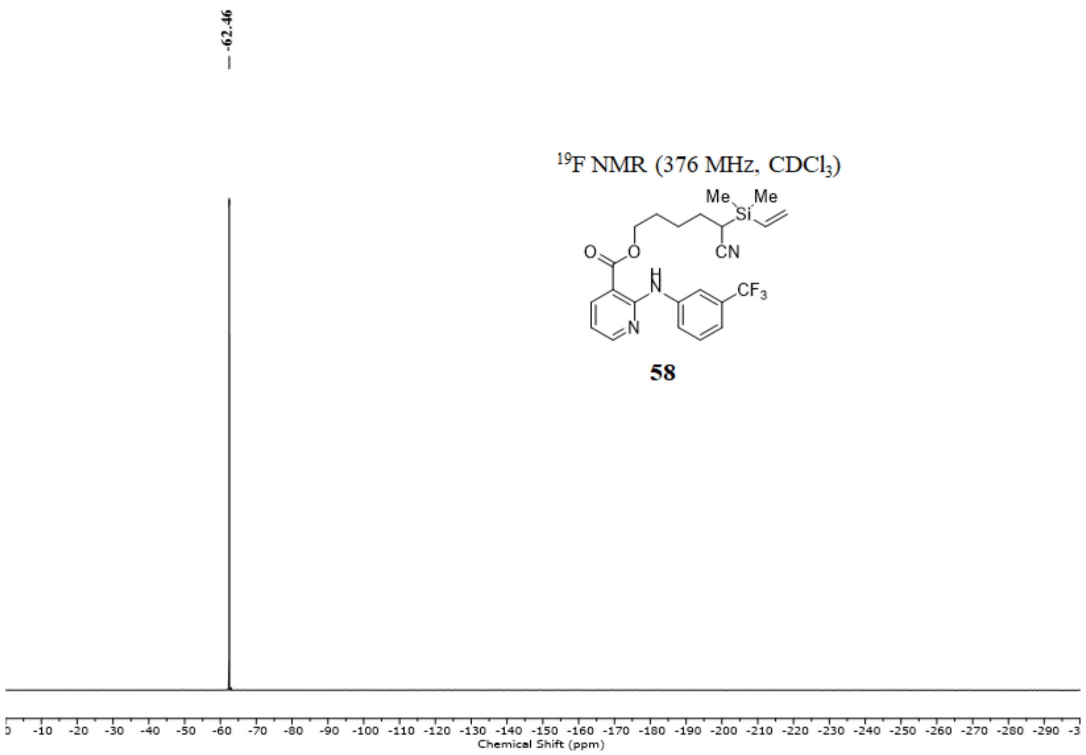

Supplementary Fig. 218. ^{19}F NMR of compound 58. The sample has been recorded in 376 MHz, CDCl$_3$ at 25 °C

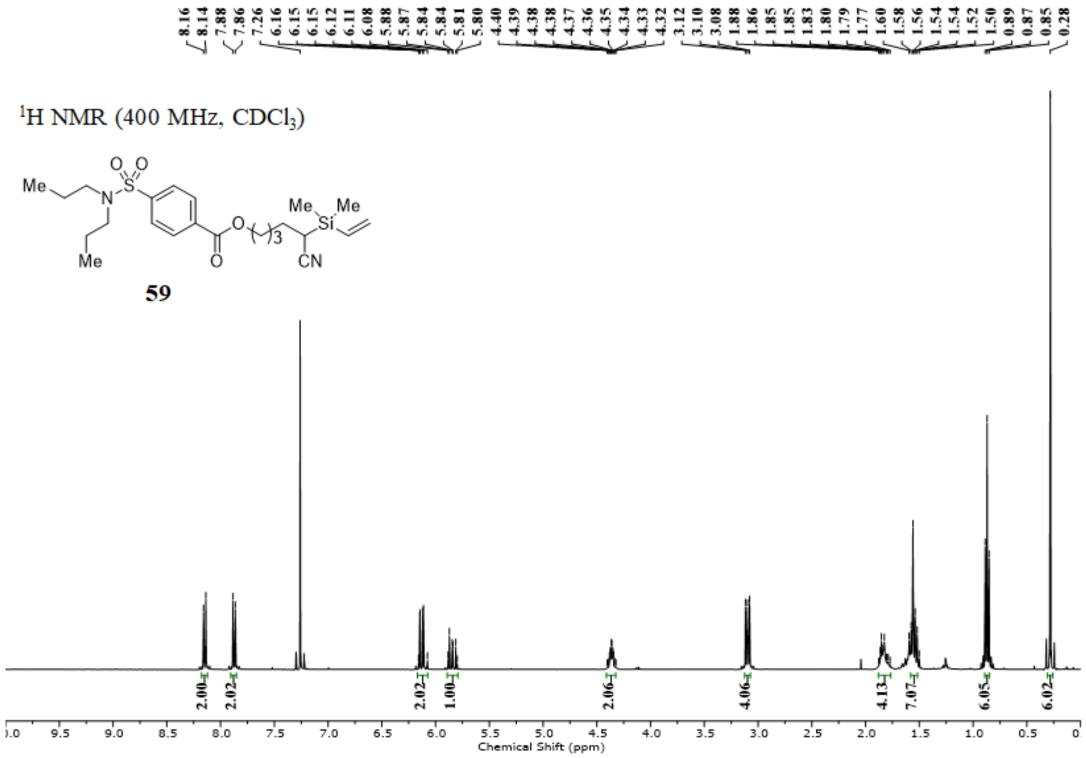

Supplementary Fig. 219. ^{1}H NMR of compound 59. The sample has been recorded in 400 MHz, CDCl$_3$ at 25 °C

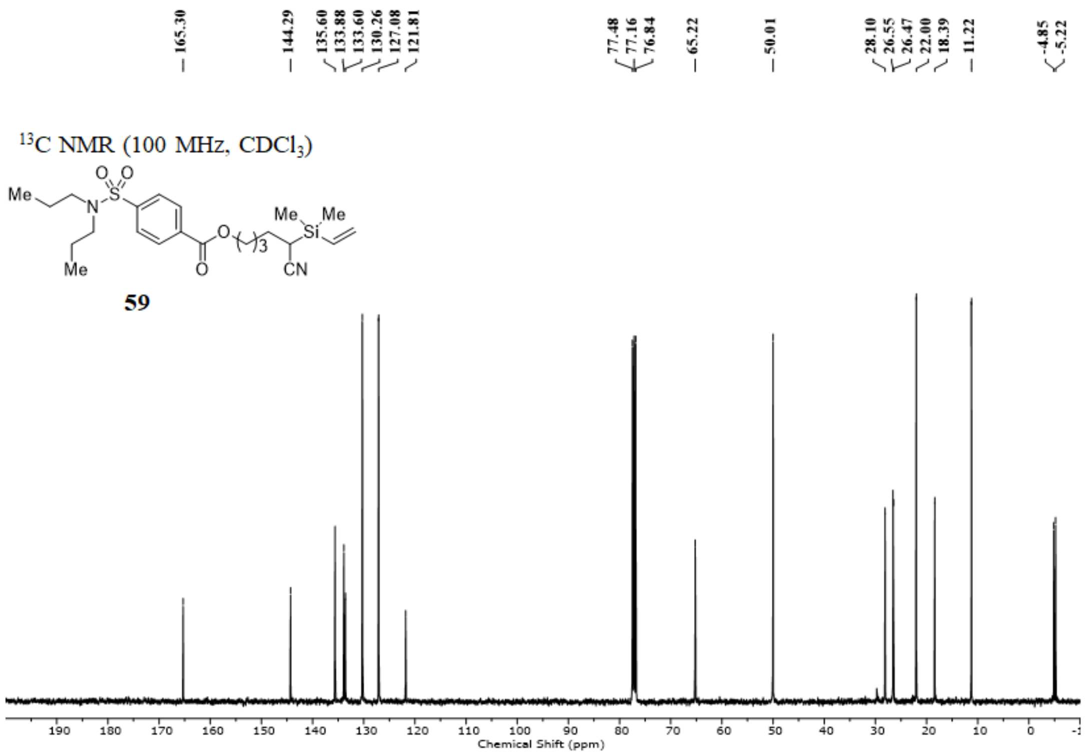

Supplementary Fig. 220. ^{13}C NMR of compound 59. The sample has been recorded in 100 MHz, $CDCl_3$ at 25 °C

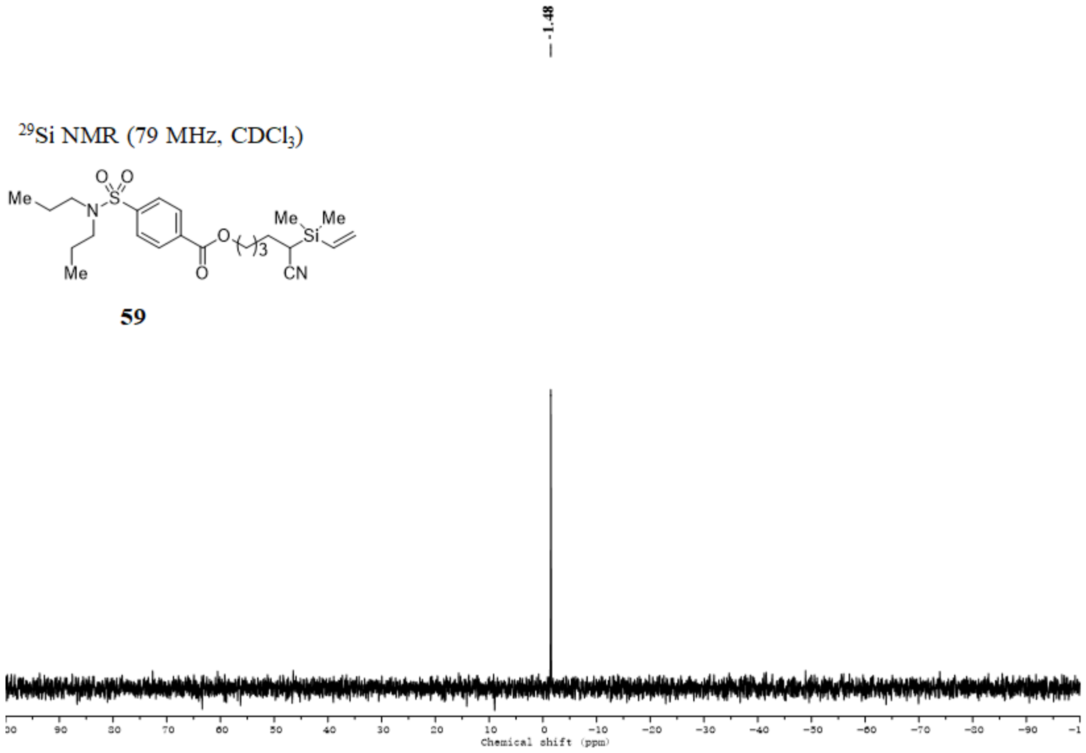

Supplementary Fig. 221. ^{29}Si NMR of compound 59. The sample has been recorded in 79 MHz, $CDCl_3$ at 25 °C

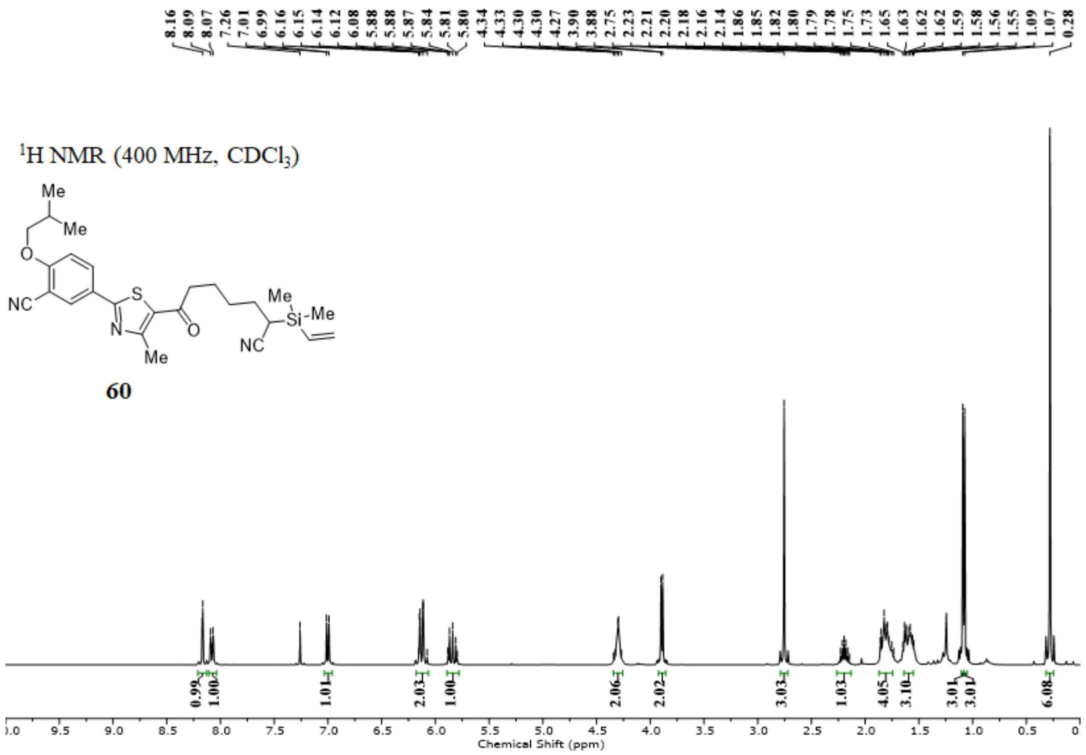

Supplementary Fig. 222. ^{1}H NMR of compound 60. The sample has been recorded in 400 MHz, $CDCl_3$ at 25 °C

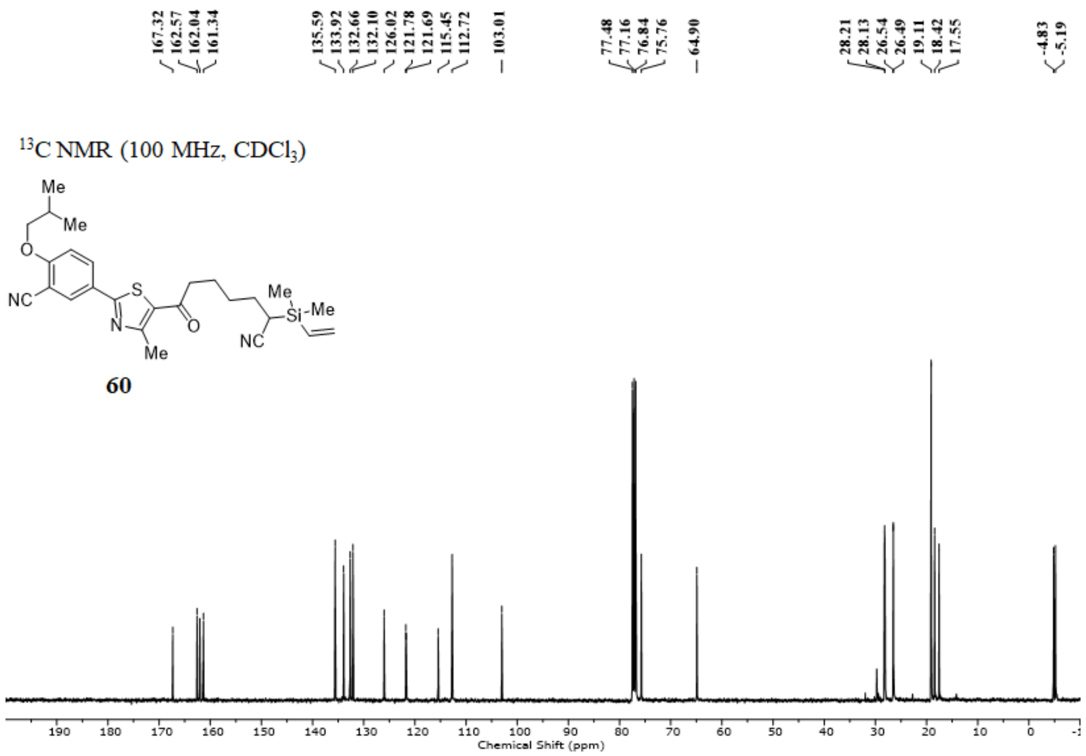

Supplementary Fig. 223. ^{13}C NMR of compound 60. The sample has been recorded in 100 MHz, $CDCl_3$ at 25 °C

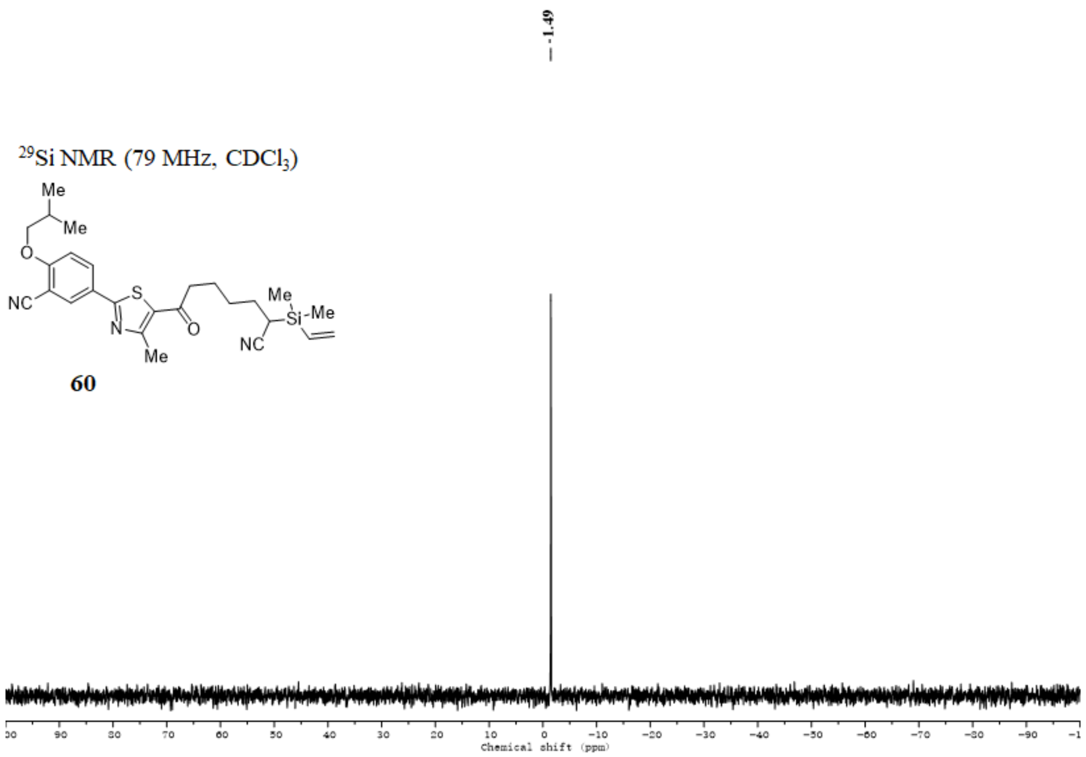

Supplementary Fig. 224. ^{29}Si NMR of compound 60. The sample has been recorded in 79 MHz, $CDCl_3$ at 25 °C

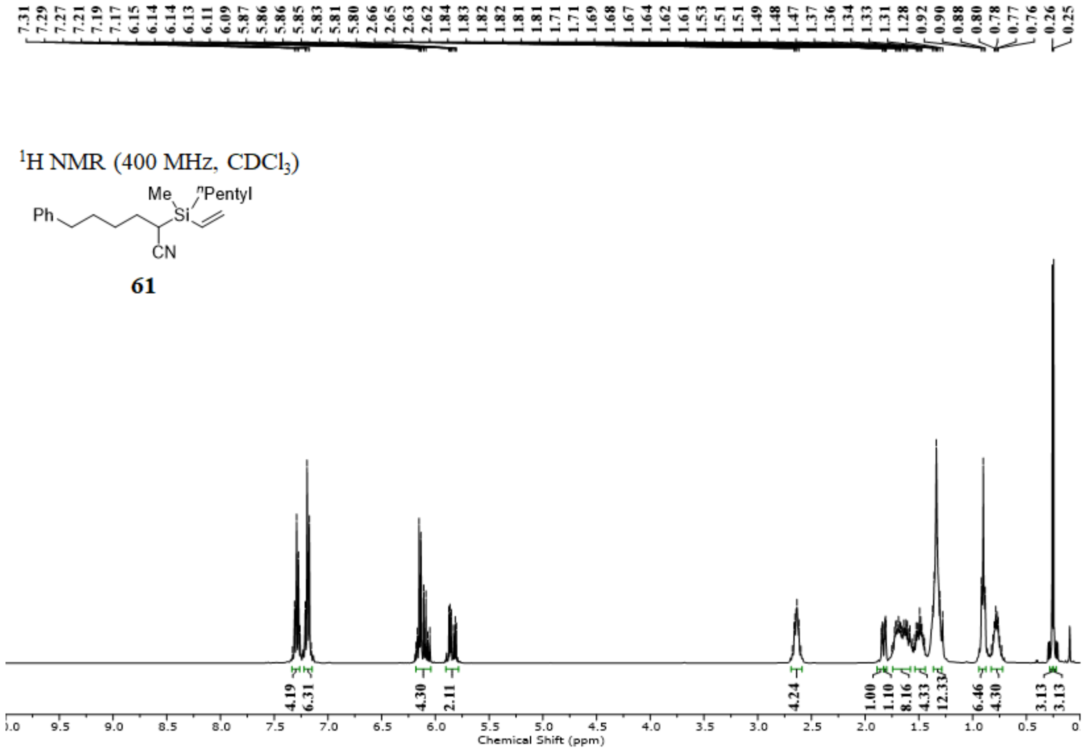

Supplementary Fig. 225. ^{1}H NMR of compound 61. The sample has been recorded in 400 MHz, $CDCl_3$ at 25 °C

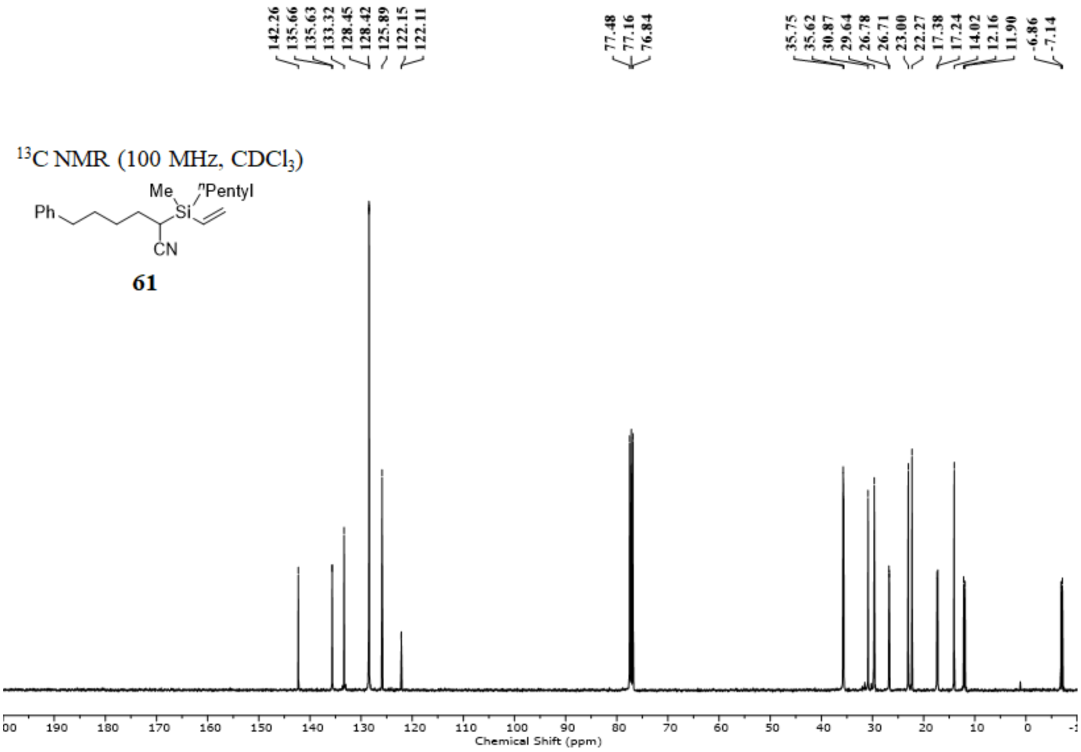

Supplementary Fig. 226. ^{13}C NMR of compound 61. The sample has been recorded in 100 MHz, $CDCl_3$ at 25 °C

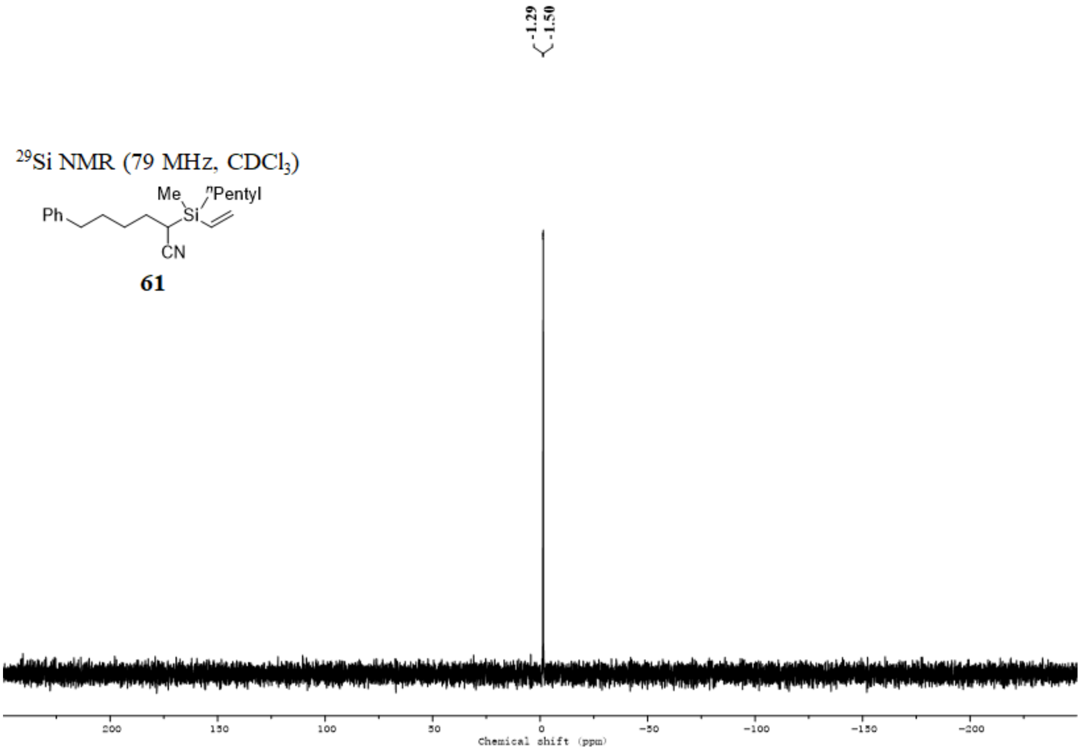

Supplementary Fig. 227. ^{29}Si NMR of compound 61. The sample has been recorded in 79 MHz, $CDCl_3$ at 25 °C

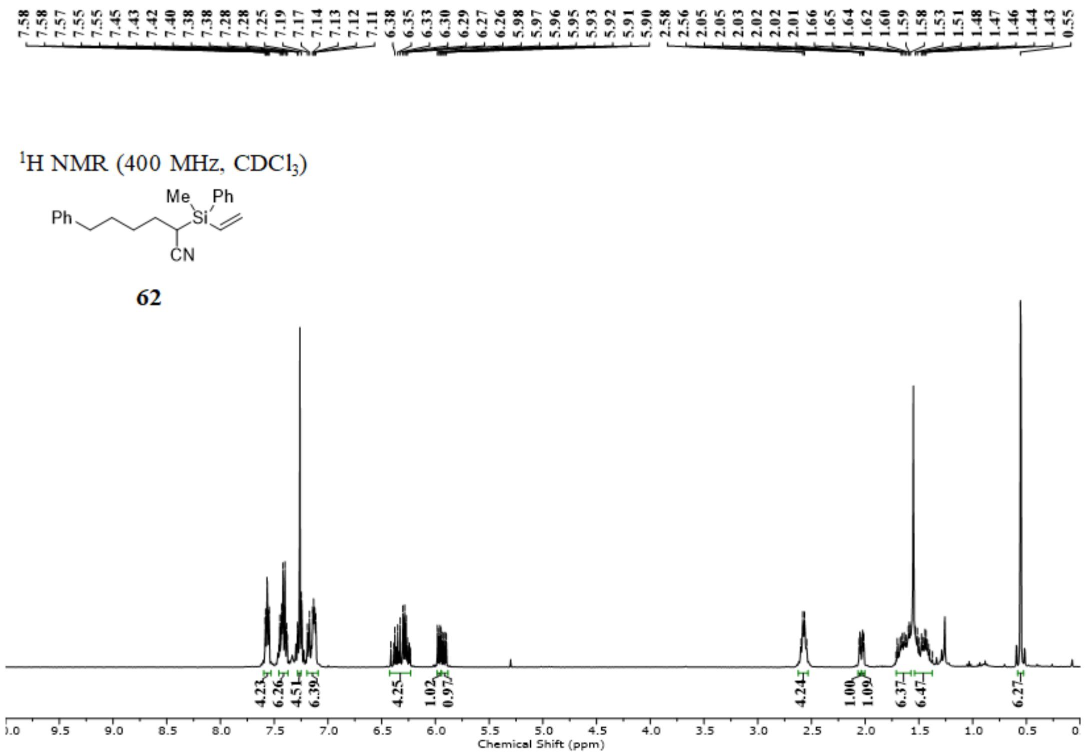

Supplementary Fig. 228. ^{1}H NMR of compound 62. The sample has been recorded in 400 MHz, $CDCl_3$ at 25 °C

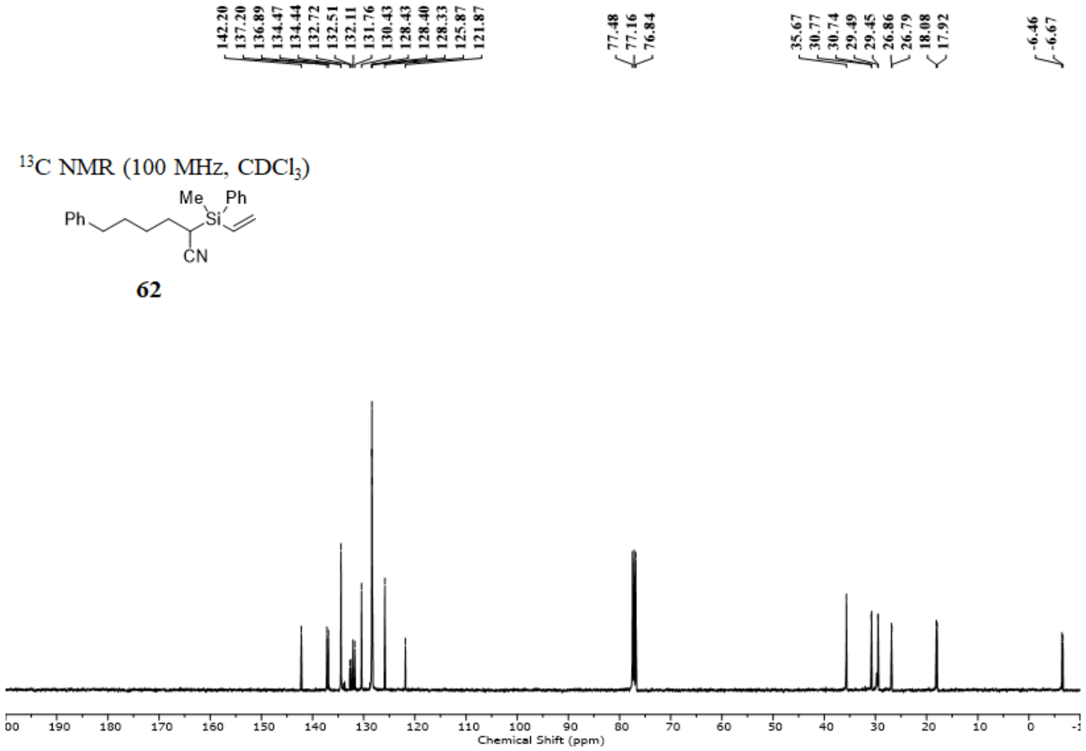

Supplementary Fig. 229. ^{13}C NMR of compound 62. The sample has been recorded in 100 MHz, $CDCl_3$ at 25 °C

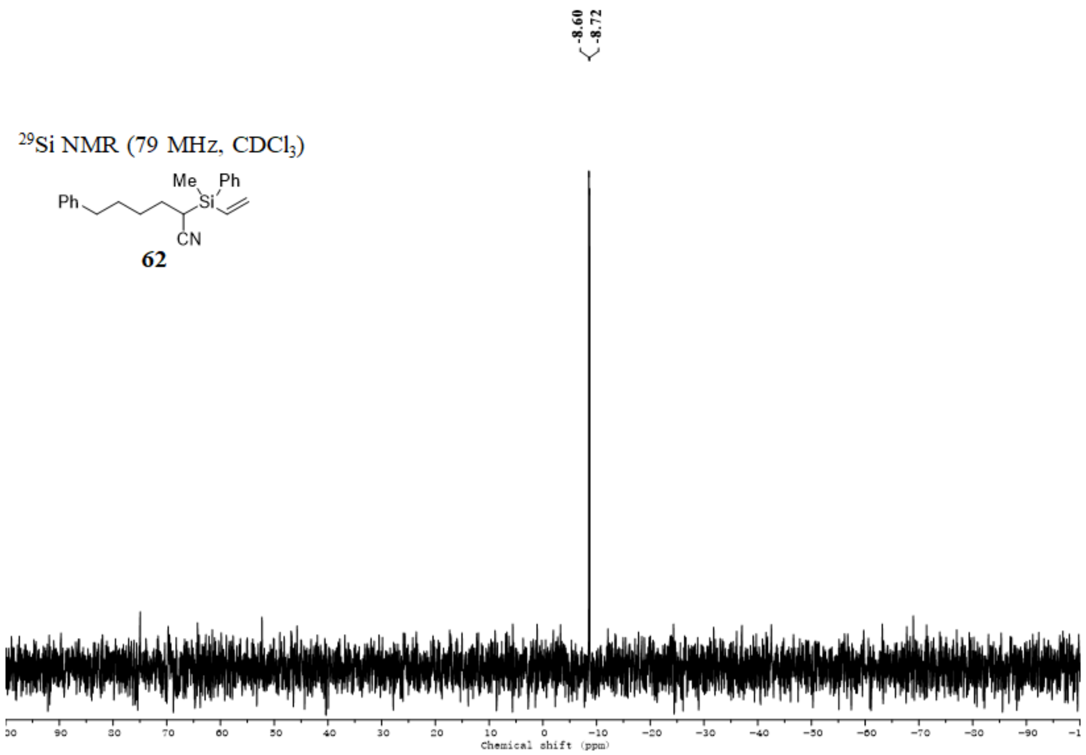

Supplementary Fig. 230. ^{29}Si NMR of compound 62. The sample has been recorded in 79 MHz, $CDCl_3$ at 25 °C

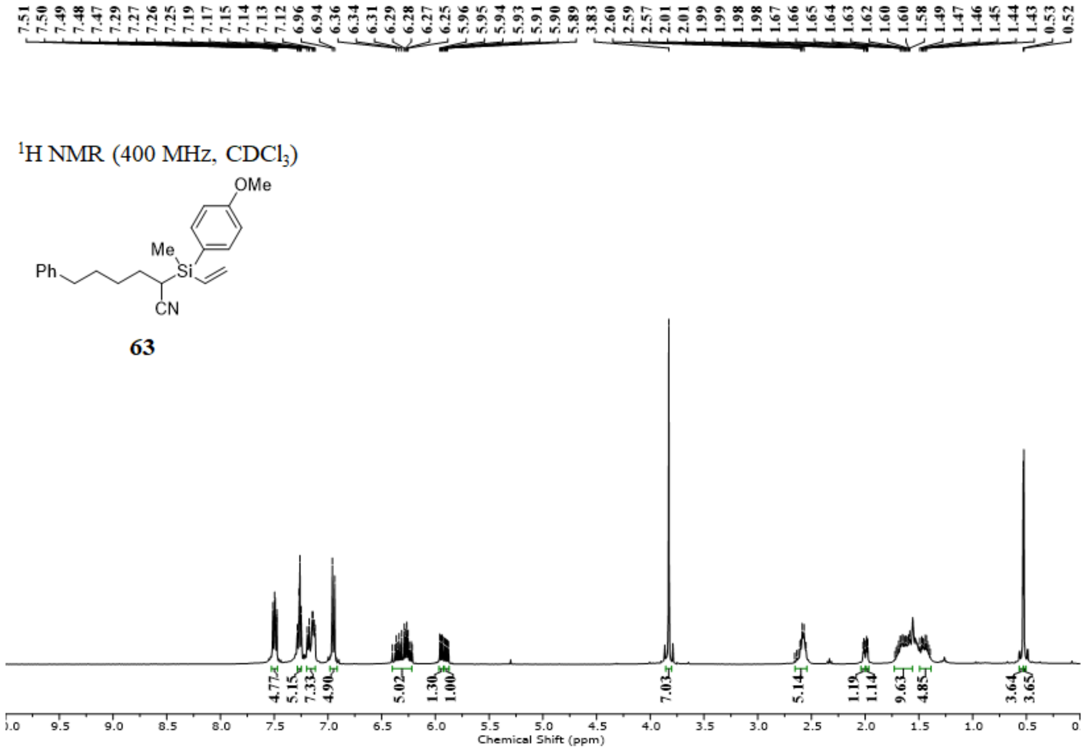

Supplementary Fig. 231. ^{1}H NMR of compound 63. The sample has been recorded in 400 MHz, $CDCl_3$ at 25 °C

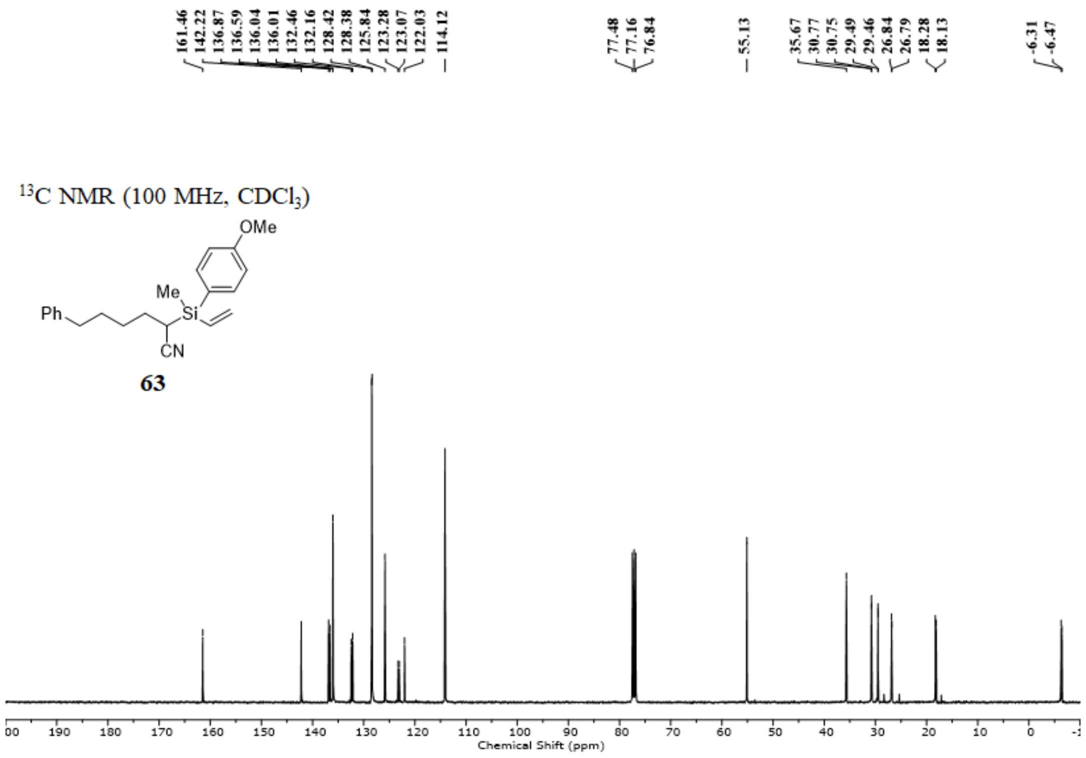

Supplementary Fig. 232. ^{13}C NMR of compound 63. The sample has been recorded in 100 MHz, $CDCl_3$ at 25 °C

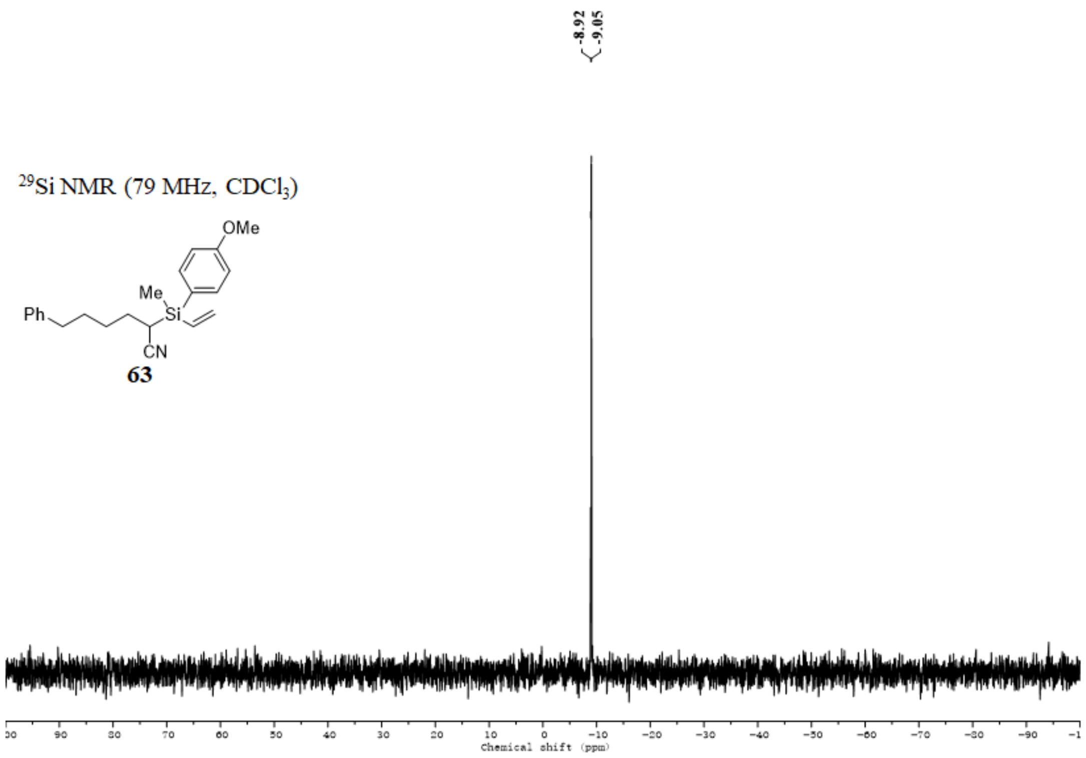

Supplementary Fig. 233. ^{29}Si NMR of compound 63. The sample has been recorded in 79 MHz, $CDCl_3$ at 25 °C

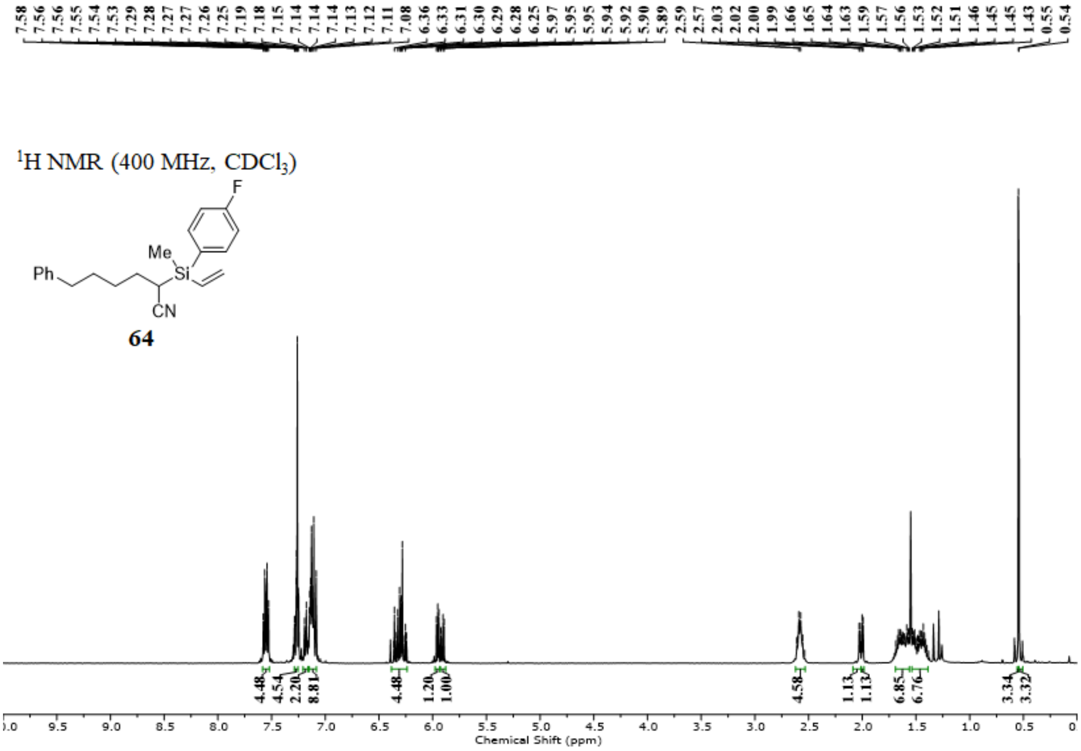

Supplementary Fig. 234. ^{1}H NMR of compound 64. The sample has been recorded in 400 MHz, $CDCl_3$ at 25 °C

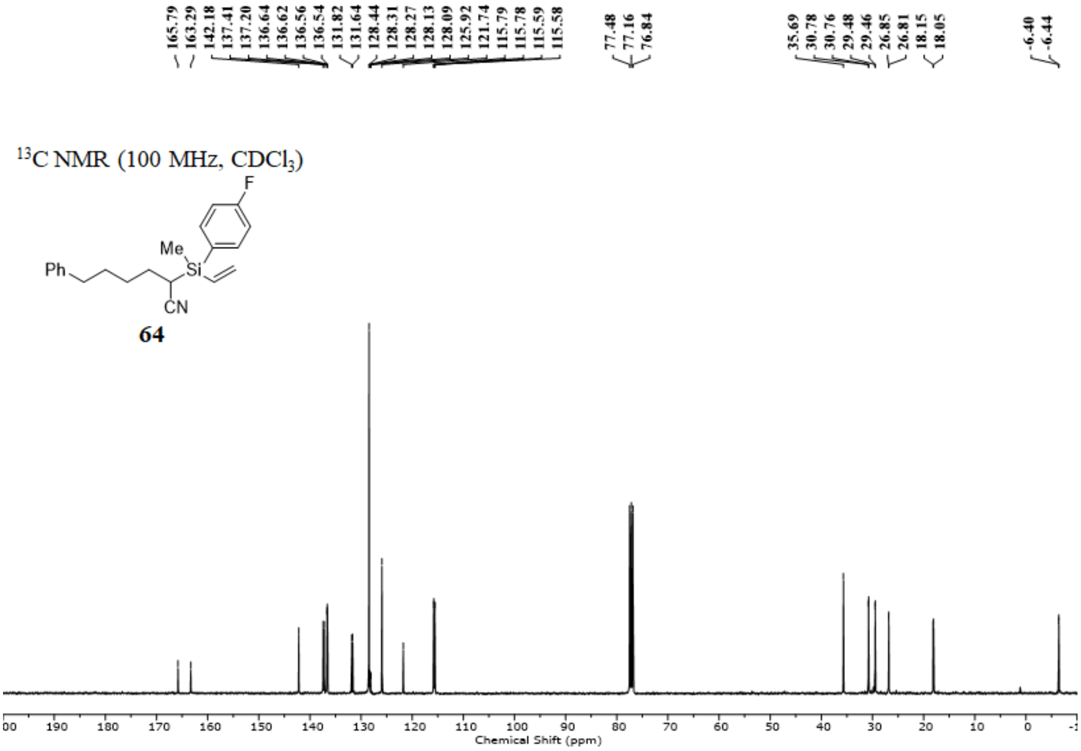

Supplementary Fig. 235. ^{13}C NMR of compound 64. The sample has been recorded in 100 MHz, $CDCl_3$ at 25 °C

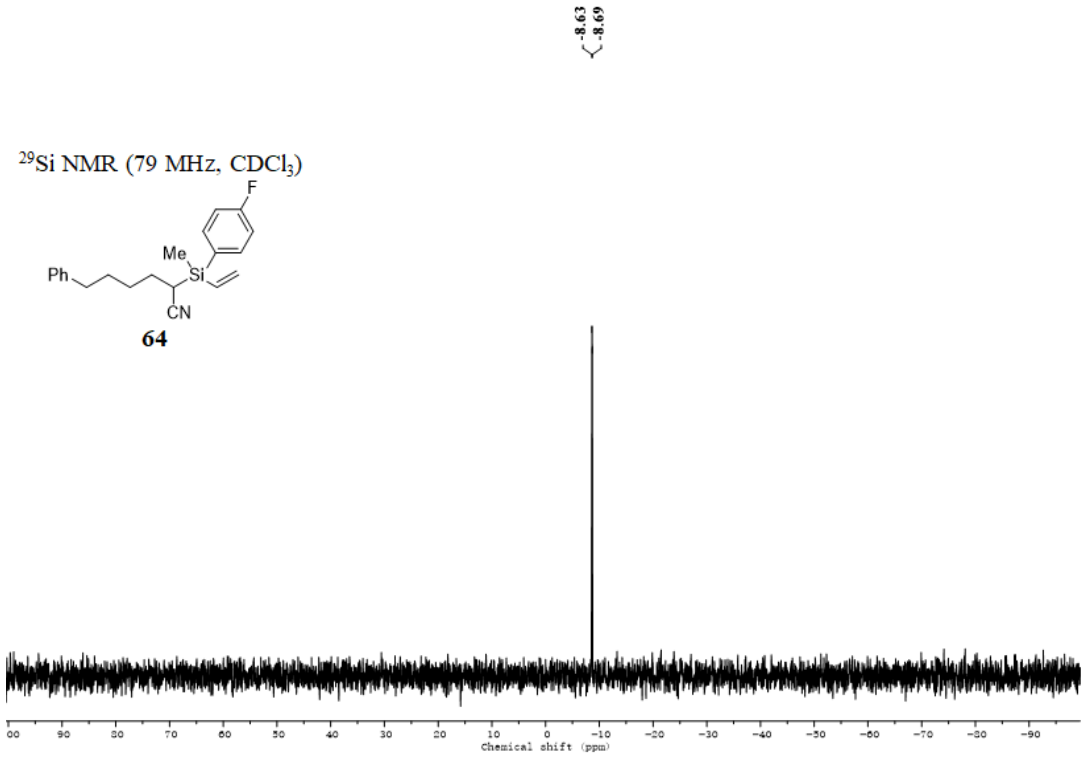

Supplementary Fig. 236. ^{29}Si NMR of compound 64. The sample has been recorded in 79 MHz, $CDCl_3$ at 25 °C

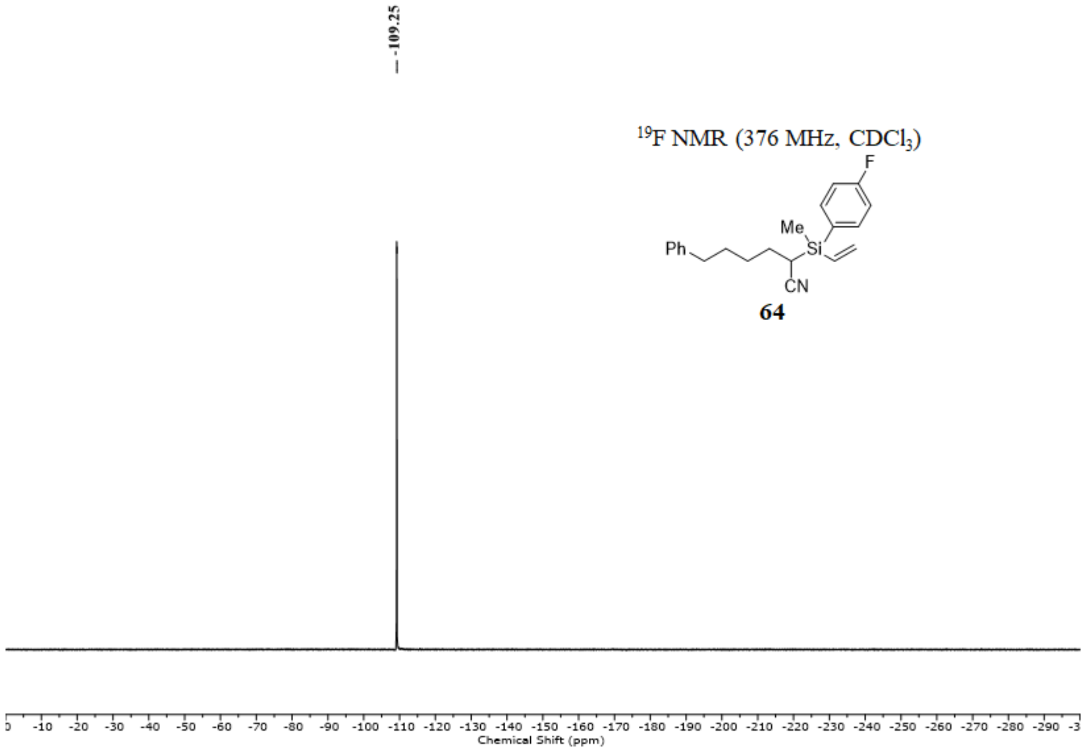

Supplementary Fig. 237. ^{19}F NMR of compound 64. The sample has been recorded in 376 MHz, $CDCl_3$ at 25 °C

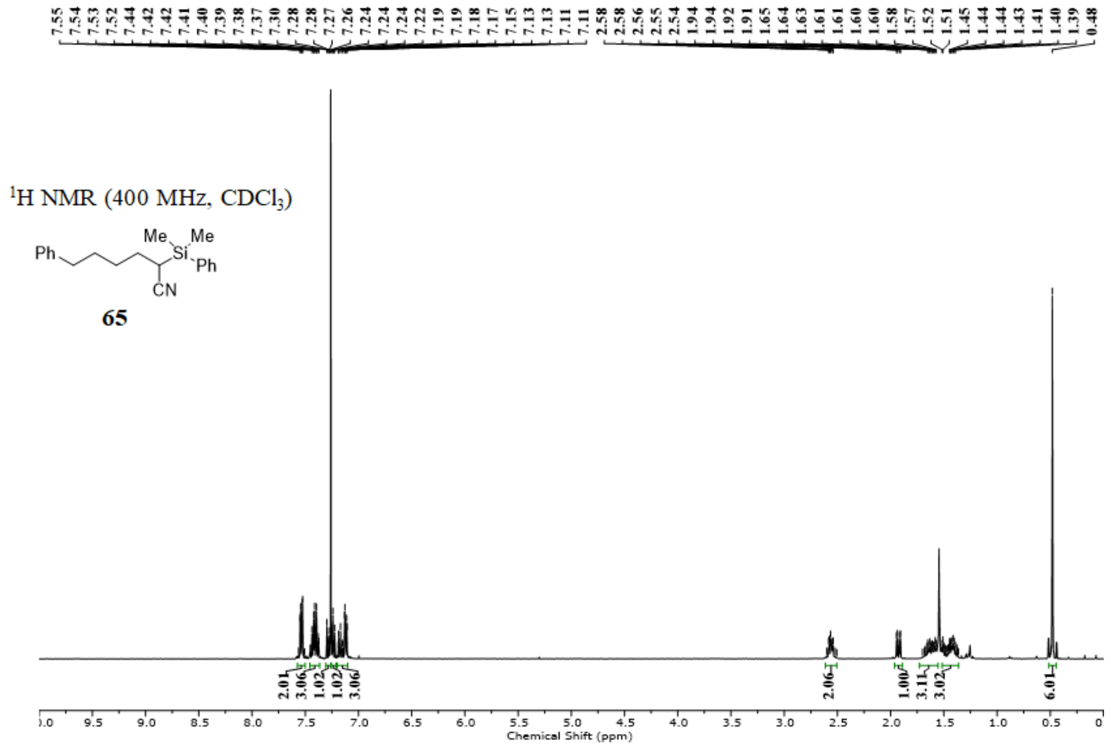

Supplementary Fig. 238. ^{1}H NMR of compound 65. The sample has been recorded in 400 MHz, $CDCl_3$ at 25 °C

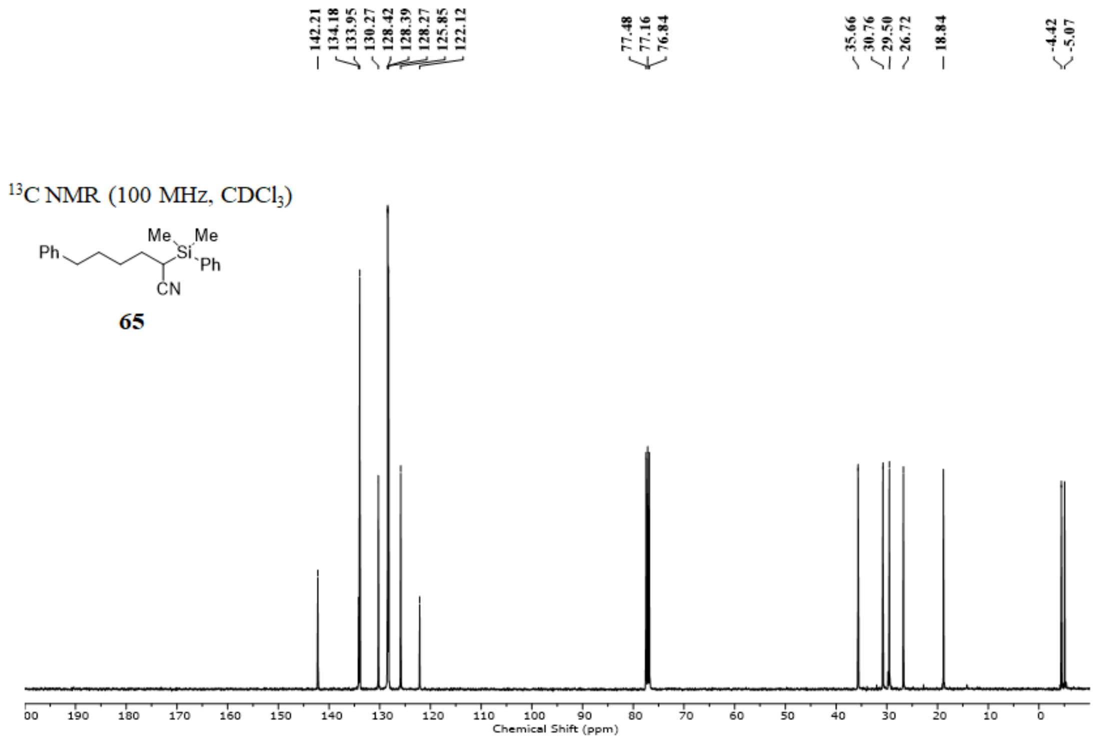

Supplementary Fig. 239. ^{13}C NMR of compound 65. The sample has been recorded in 100 MHz, $CDCl_3$ at 25 °C

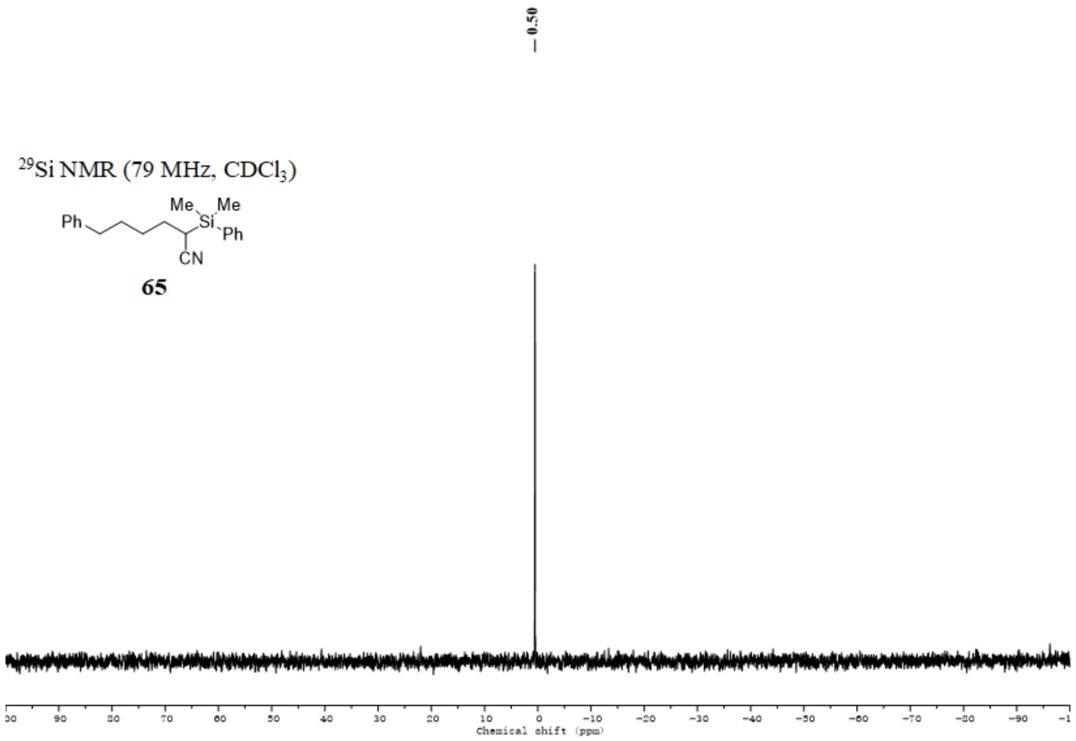

Supplementary Fig. 240. ^{29}Si NMR of compound 65. The sample has been recorded in 79 MHz, $CDCl_3$ at 25 °C

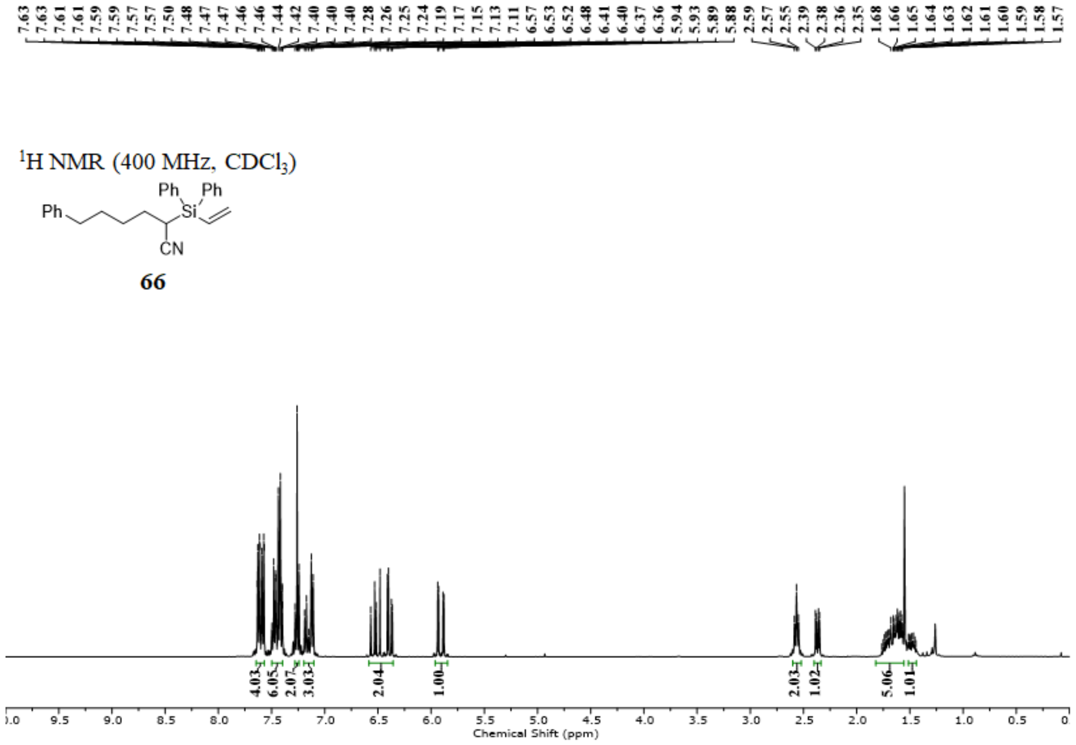

Supplementary Fig. 241. ^{1}H NMR of compound 66. The sample has been recorded in 400 MHz, $CDCl_3$ at 25 °C

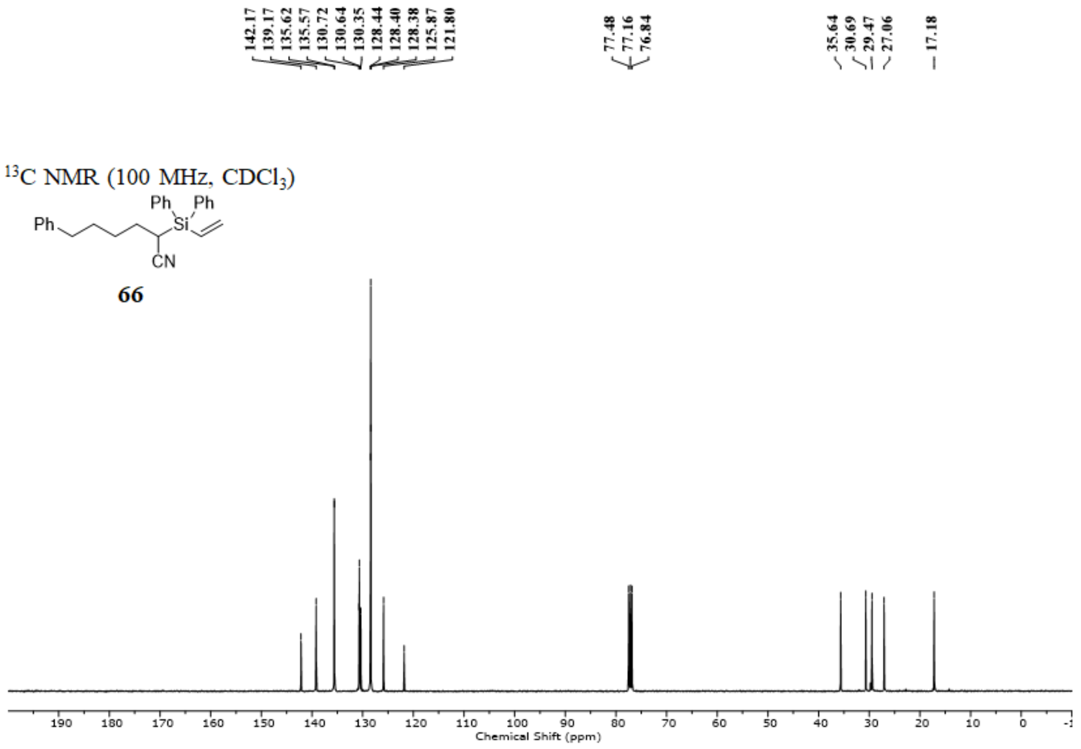

Supplementary Fig. 242. ^{13}C NMR of compound 66. The sample has been recorded in 100 MHz, $CDCl_3$ at 25 °C

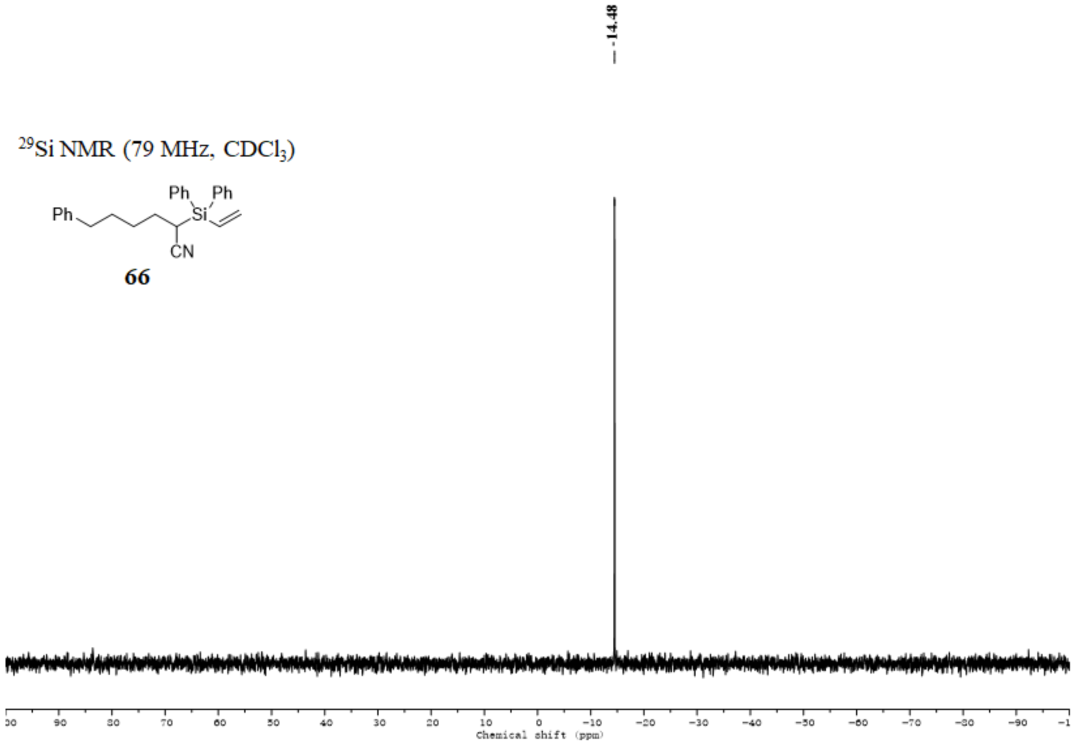

Supplementary Fig. 243. ^{29}Si NMR of compound 66. The sample has been recorded in 79 MHz, $CDCl_3$ at 25 °C

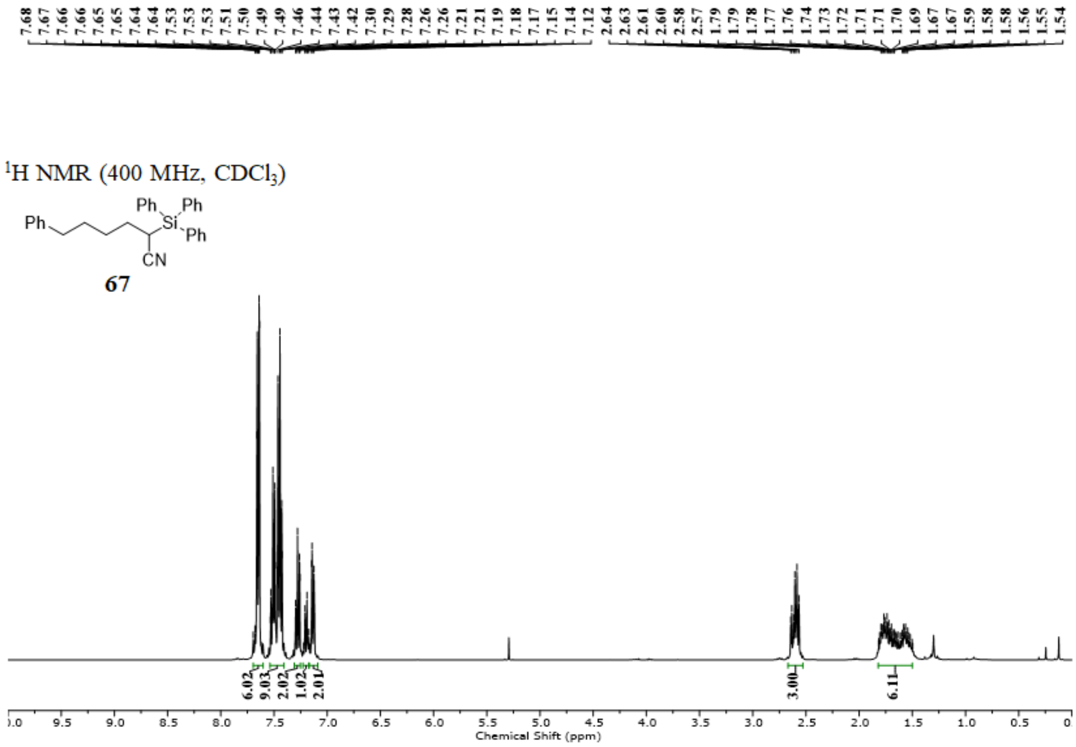

Supplementary Fig. 244. ^{1}H NMR of compound 67. The sample has been recorded in 400 MHz, $CDCl_3$ at 25 °C

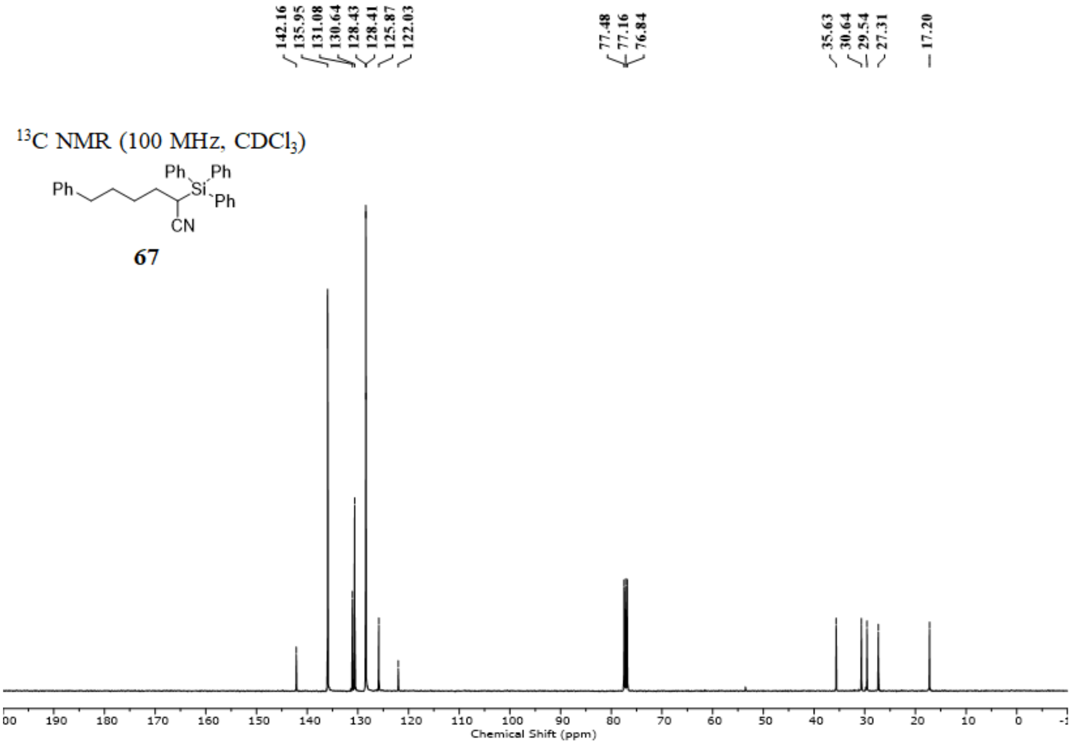

Supplementary Fig. 245. ^{13}C NMR of compound 67. The sample has been recorded in 100 MHz, $CDCl_3$ at 25 °C

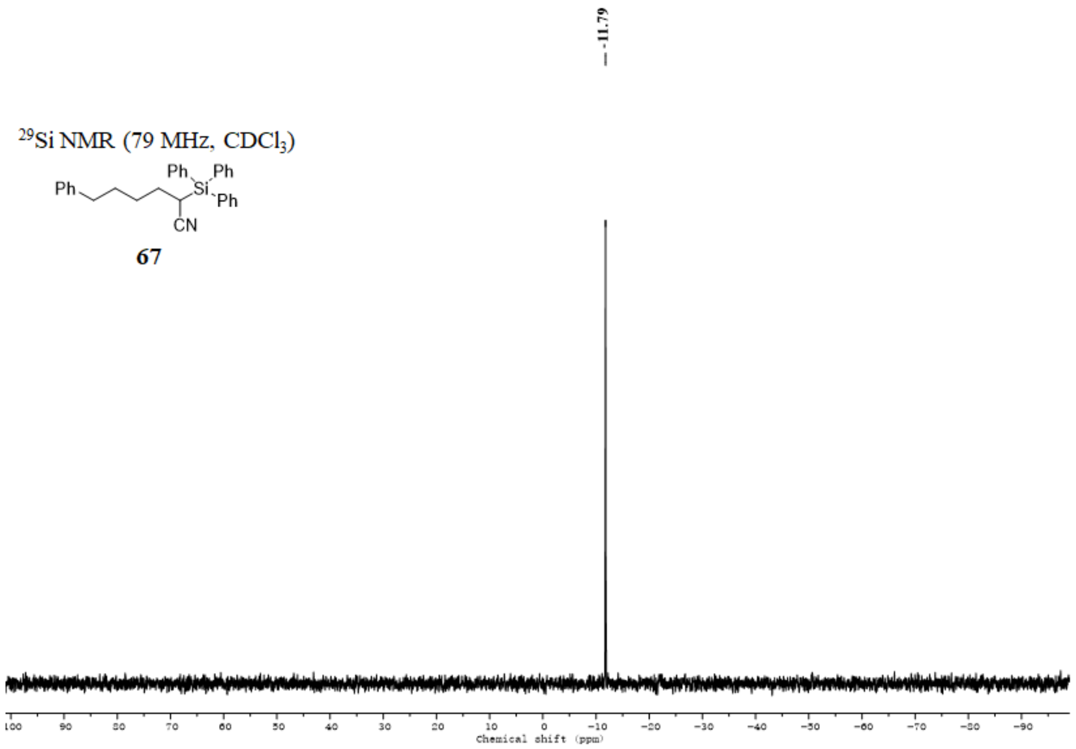

Supplementary Fig. 246. ^{29}Si NMR of compound 67. The sample has been recorded in 79 MHz, $CDCl_3$ at 25 °C

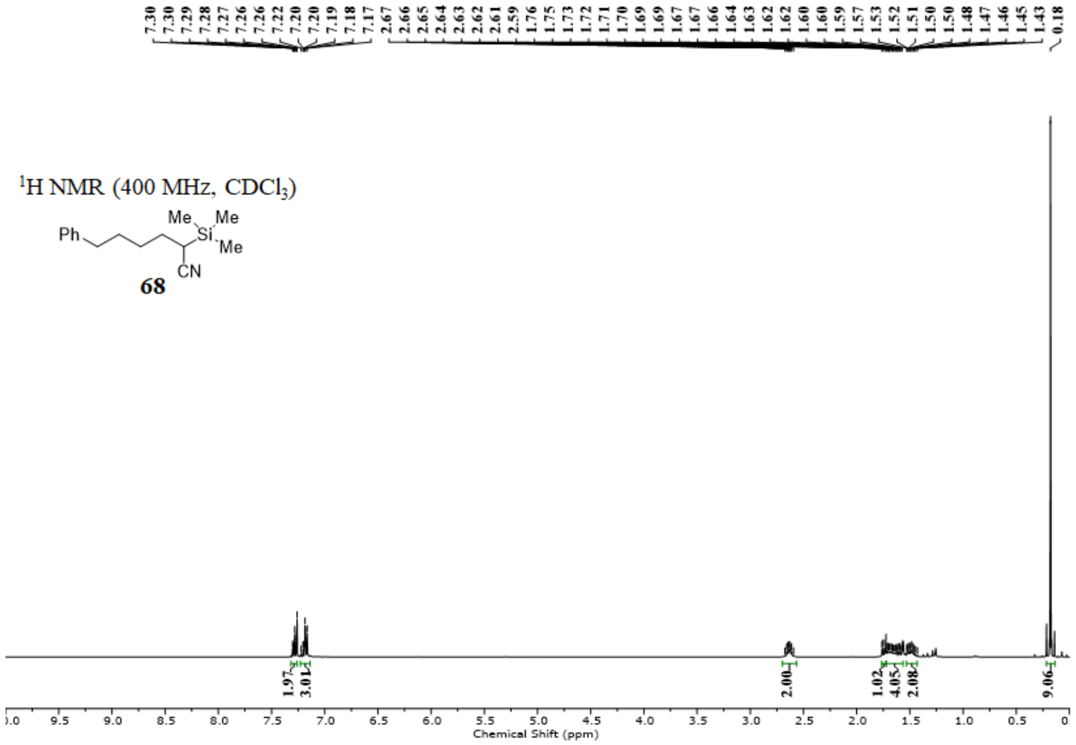

Supplementary Fig. 247. ^{1}H NMR of compound 68. The sample has been recorded in 400 MHz, $CDCl_3$ at 25 °C

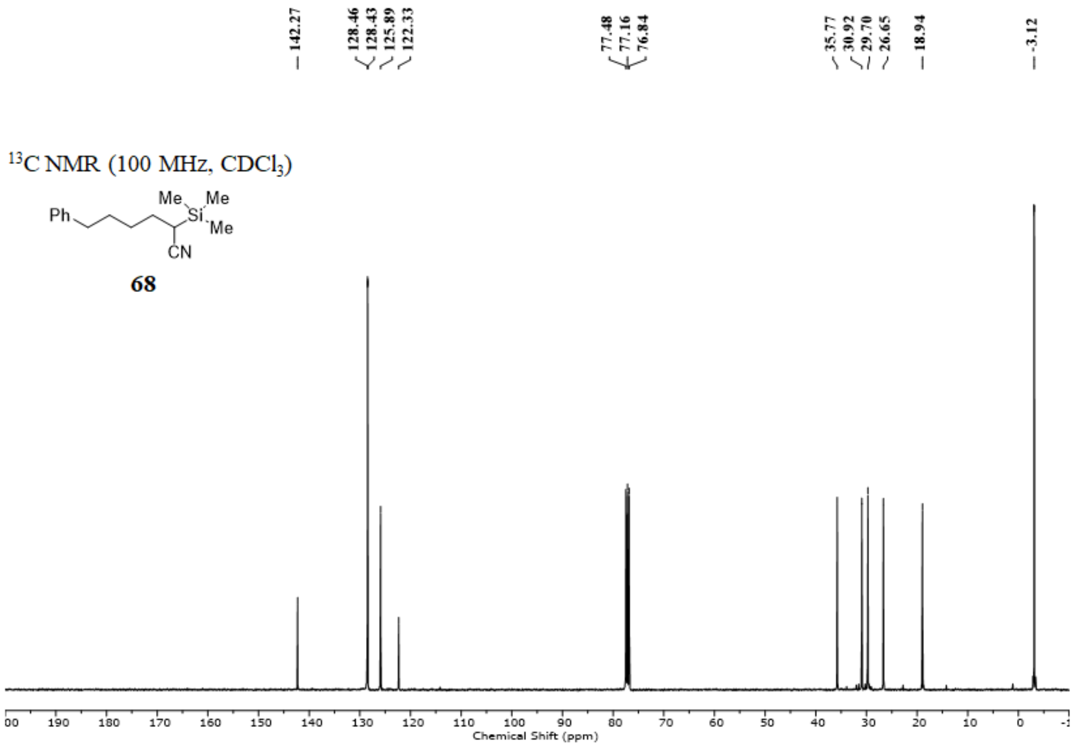

Supplementary Fig. 248. ^{13}C NMR of compound 68. The sample has been recorded in 100 MHz, $CDCl_3$ at 25 °C

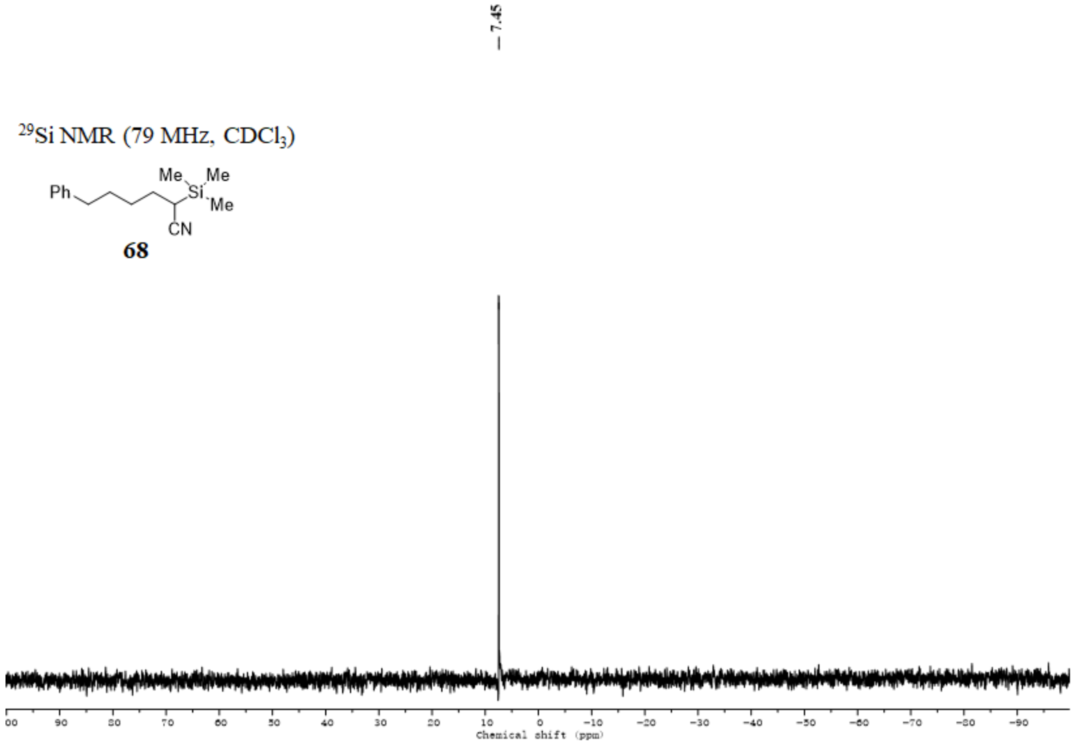

Supplementary Fig. 249. ^{29}Si NMR of compound 68. The sample has been recorded in 79 MHz, $CDCl_3$ at 25 °C

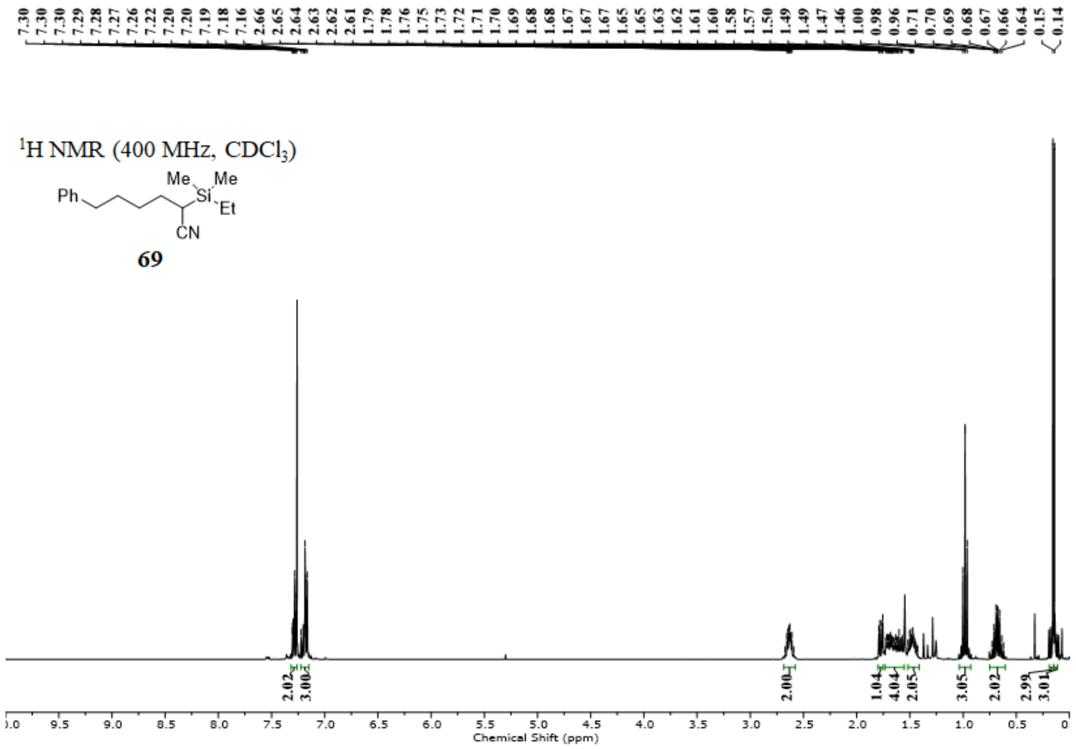

Supplementary Fig. 250. ^{1}H NMR of compound 69. The sample has been recorded in 400 MHz, $CDCl_3$ at 25 °C

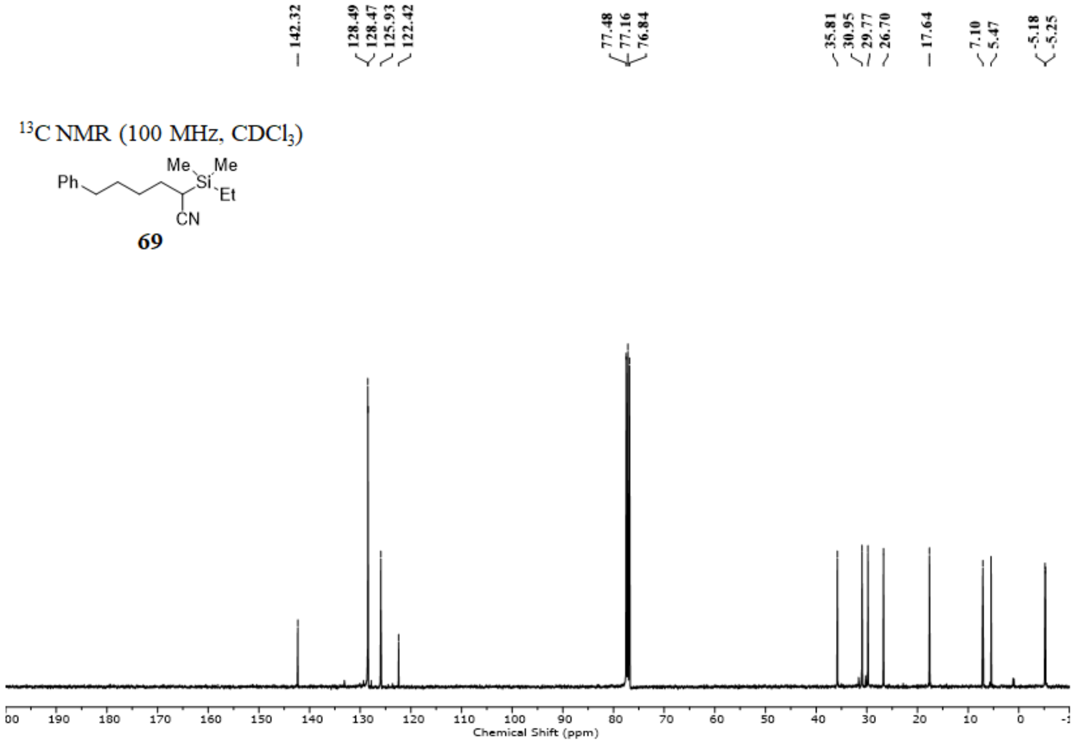

Supplementary Fig. 251. ^{13}C NMR of compound 69. The sample has been recorded in 100 MHz, $CDCl_3$ at 25 °C

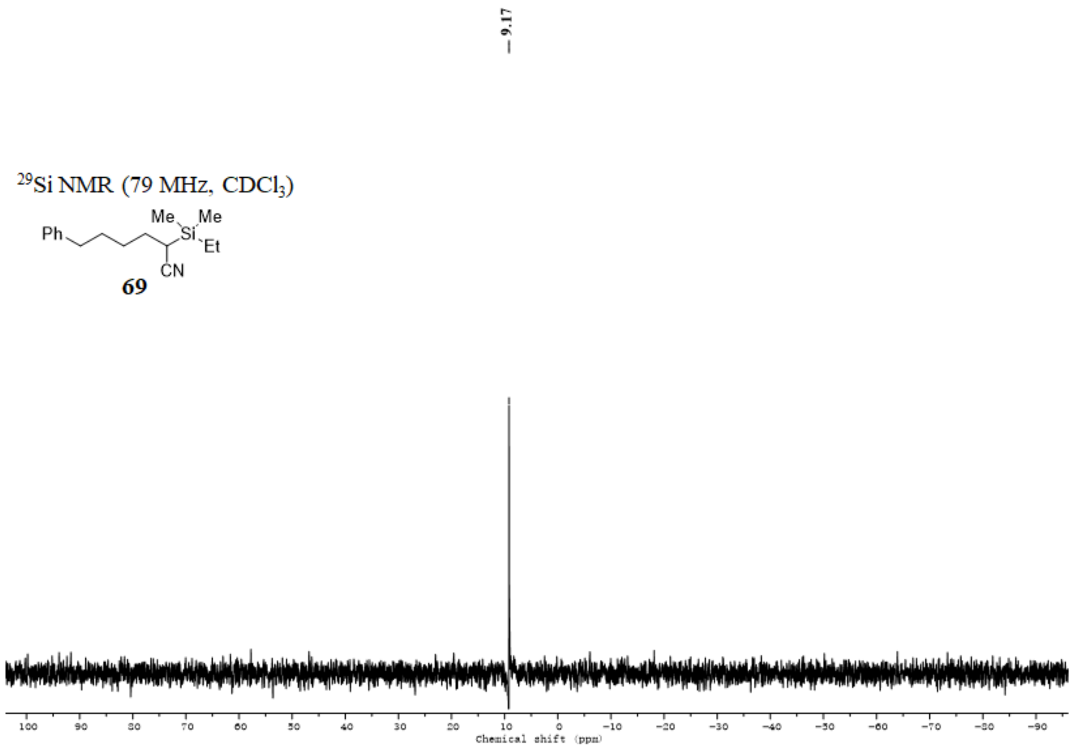

Supplementary Fig. 252. ^{29}Si NMR of compound 69. The sample has been recorded in 79 MHz, $CDCl_3$ at 25 °C

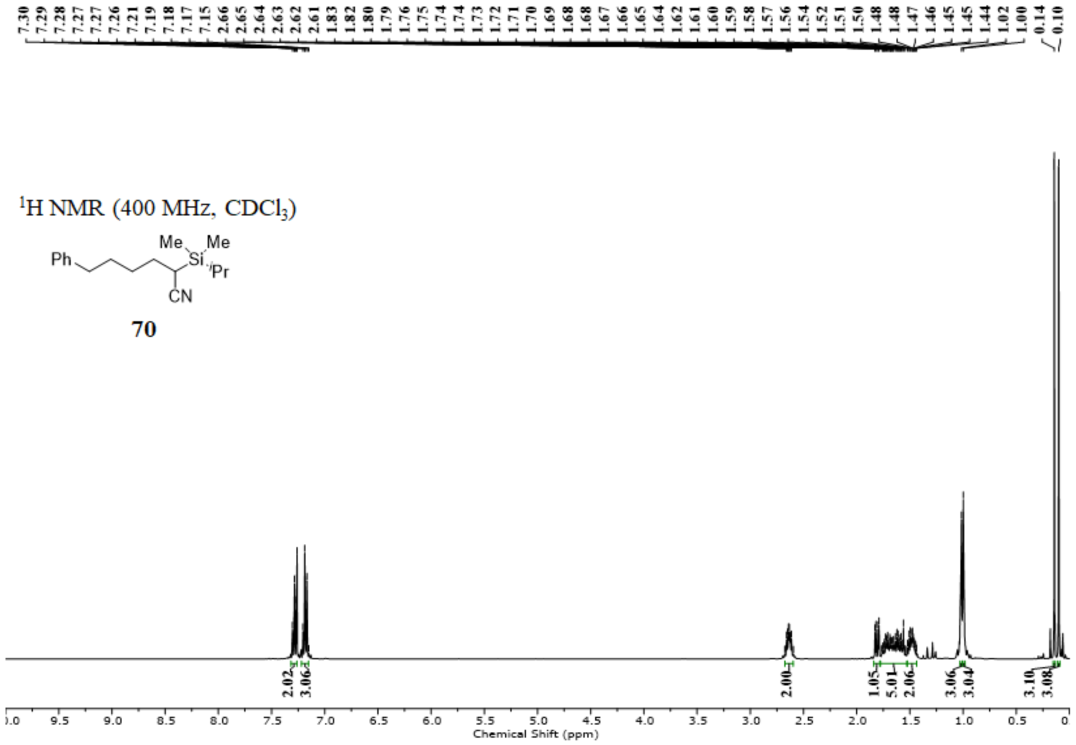

Supplementary Fig. 253. ^{1}H NMR of compound 70. The sample has been recorded in 400 MHz, $CDCl_3$ at 25 °C

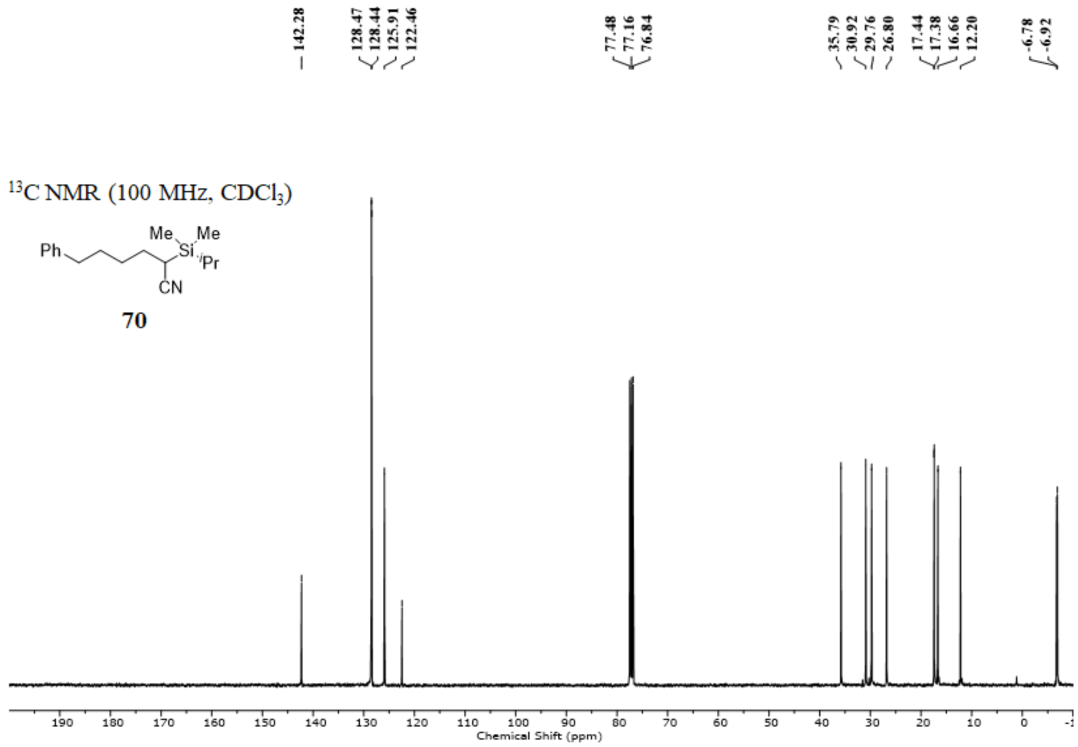

Supplementary Fig. 254. ^{13}C NMR of compound 70. The sample has been recorded in 100 MHz, $CDCl_3$ at 25 °C

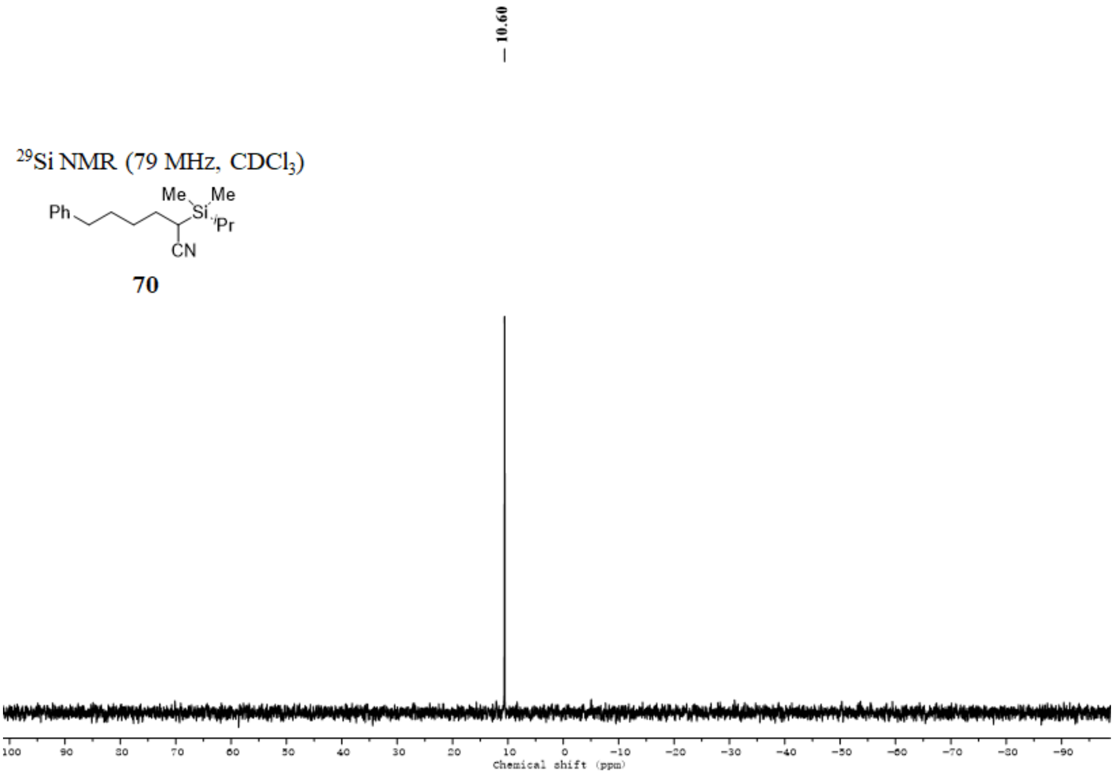

Supplementary Fig. 255. ^{29}Si NMR of compound 70. The sample has been recorded in 79 MHz, $CDCl_3$ at 25 °C

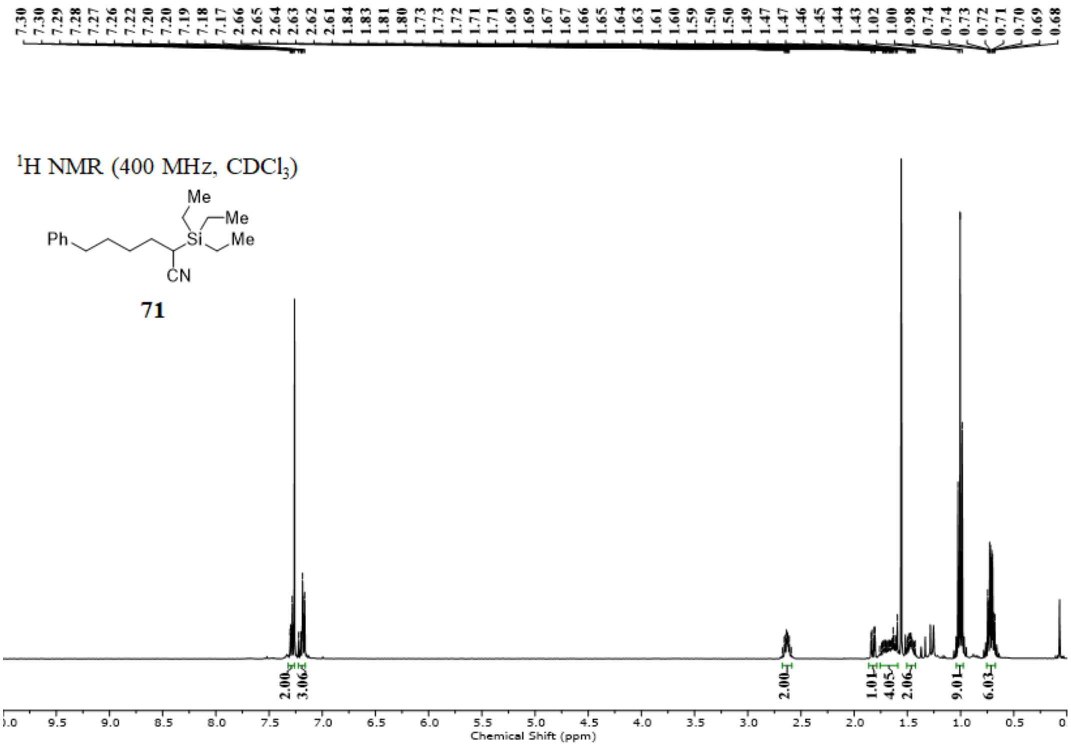

Supplementary Fig. 256. ^{1}H NMR of compound 71. The sample has been recorded in 400 MHz, $CDCl_3$ at 25 °C

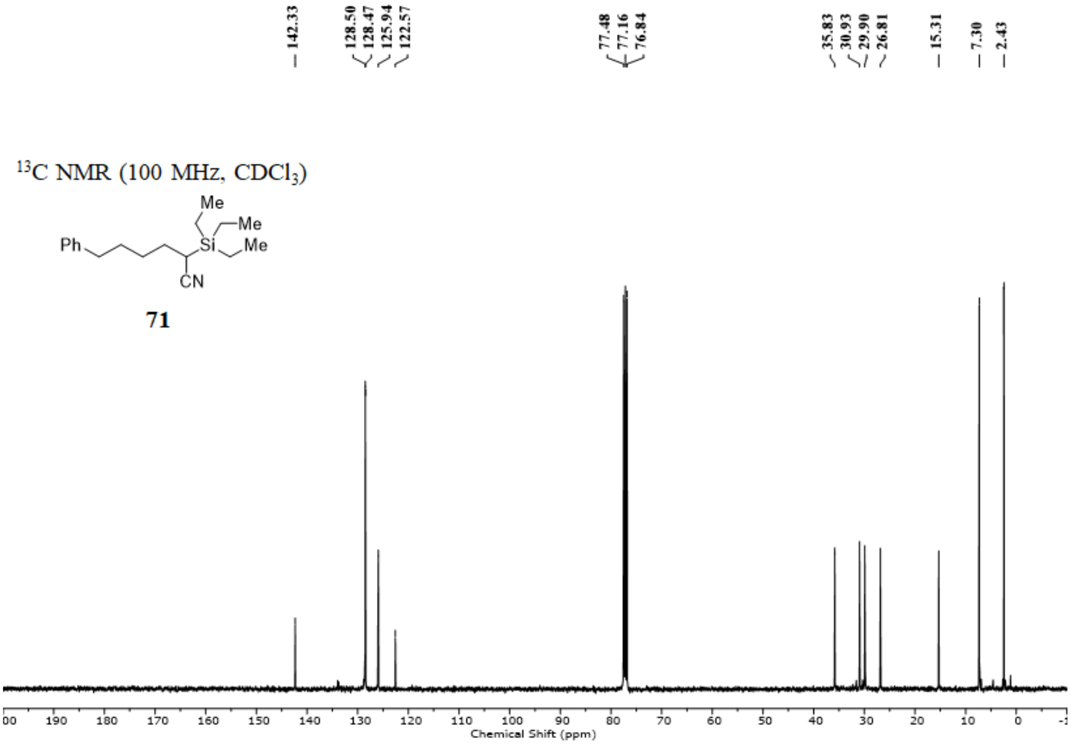

Supplementary Fig. 257. ^{13}C NMR of compound 71. The sample has been recorded in 100 MHz, $CDCl_3$ at 25 °C

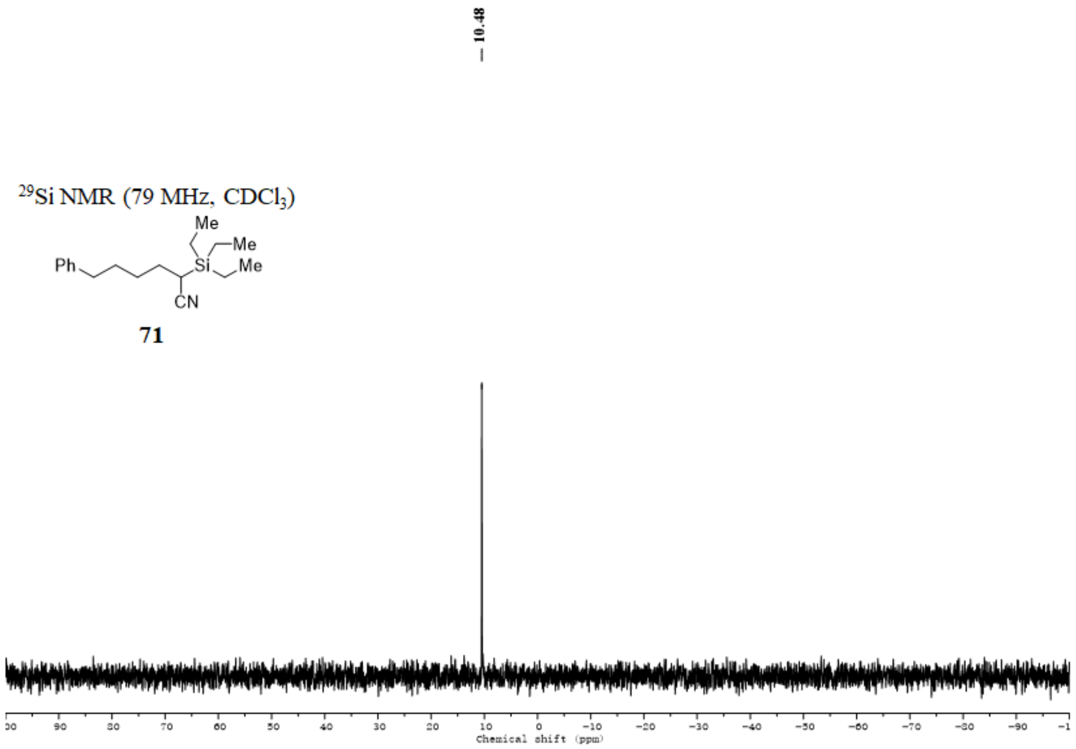

Supplementary Fig. 258. ^{29}Si NMR of compound 71. The sample has been recorded in 79 MHz, $CDCl_3$ at 25 °C

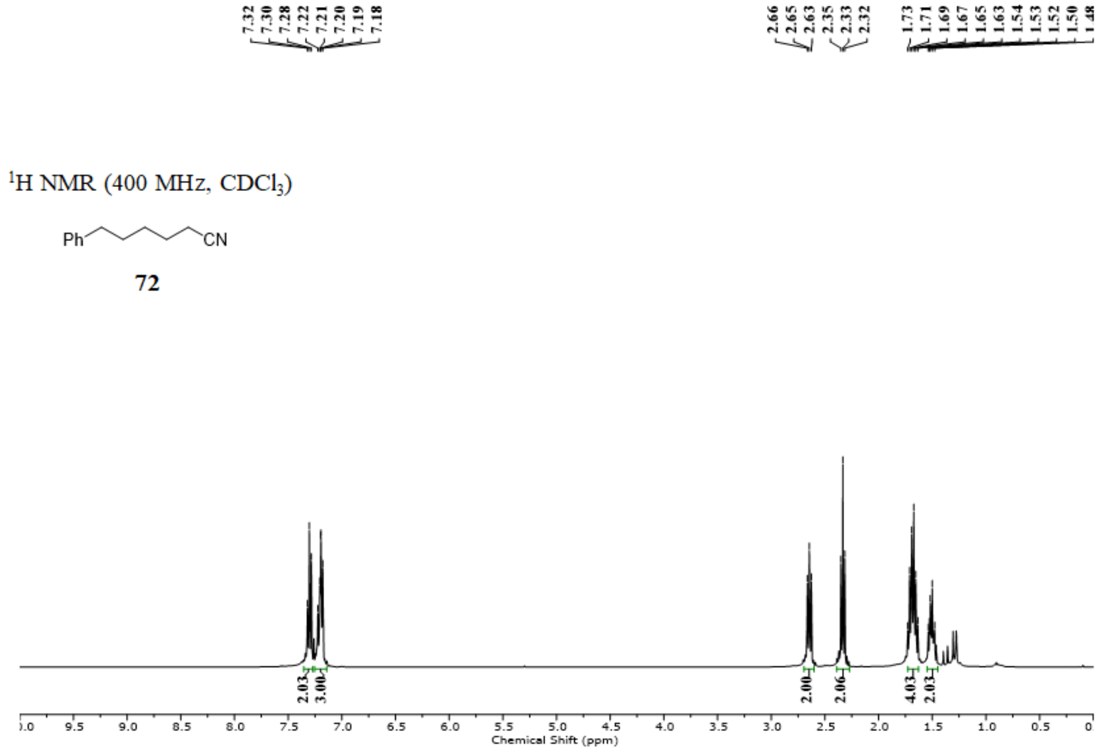

Supplementary Fig. 259. ^{1}H NMR of compound 72. The sample has been recorded in 400 MHz, $CDCl_3$ at 25 °C

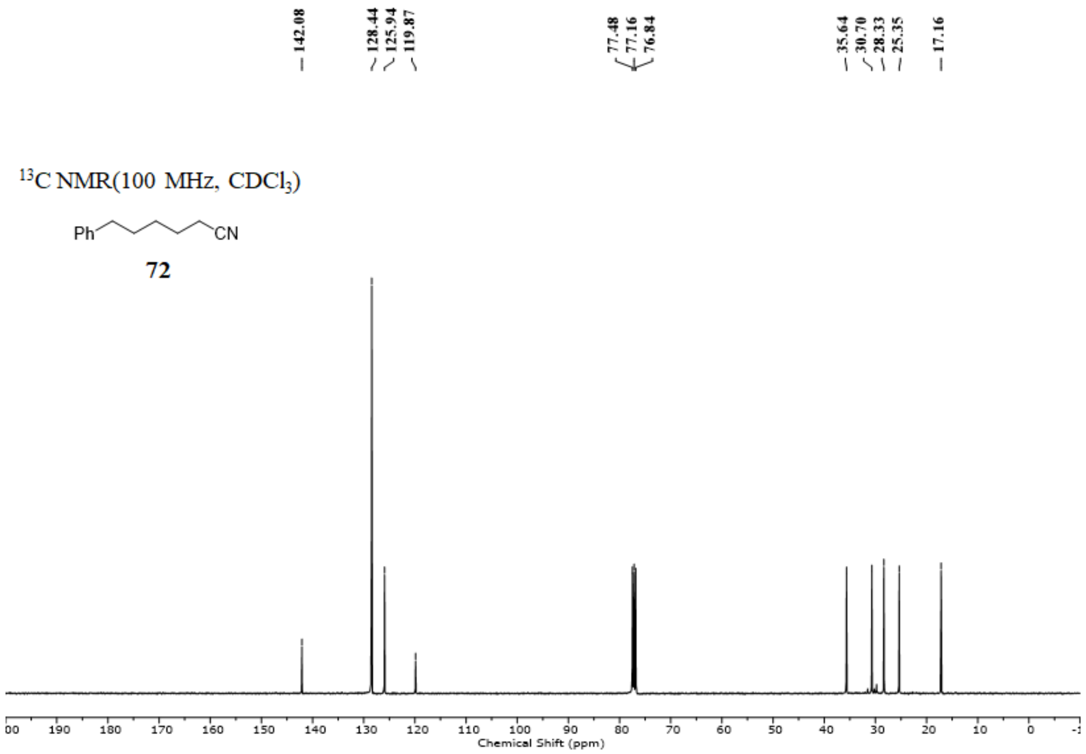

Supplementary Fig. 260. ^{13}C NMR of compound 72. The sample has been recorded in 100 MHz, $CDCl_3$ at 25 °C

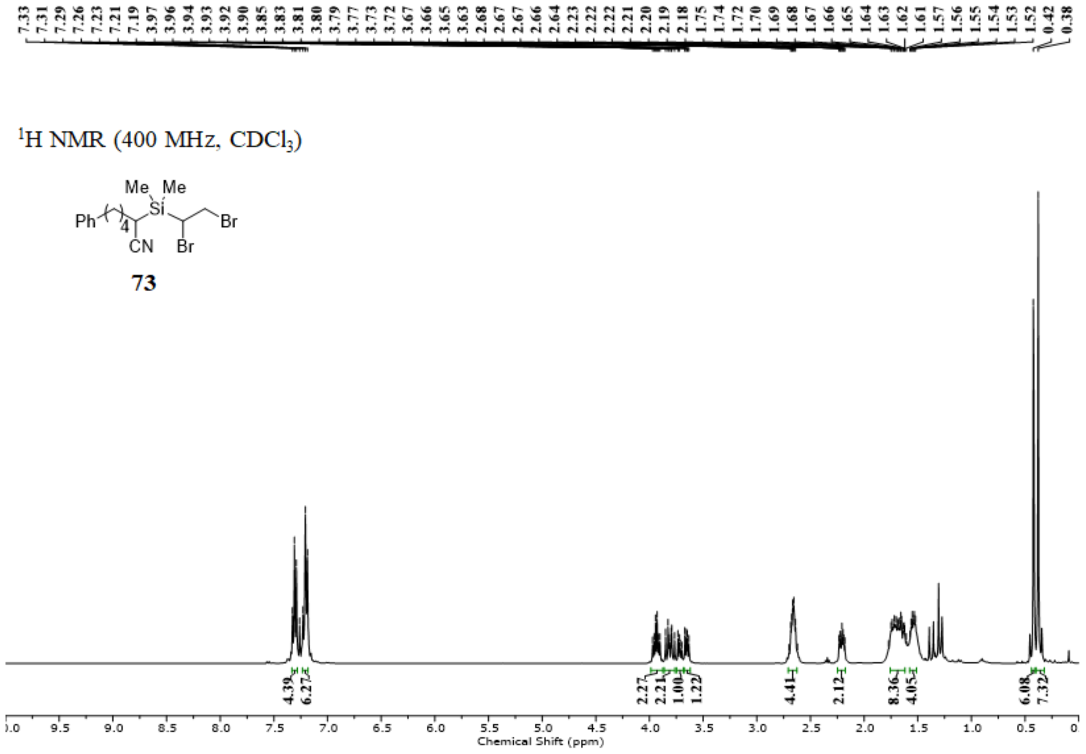

Supplementary Fig. 261. ^{1}H NMR of compound 73. The sample has been recorded in 400 MHz, $CDCl_3$ at 25 °C

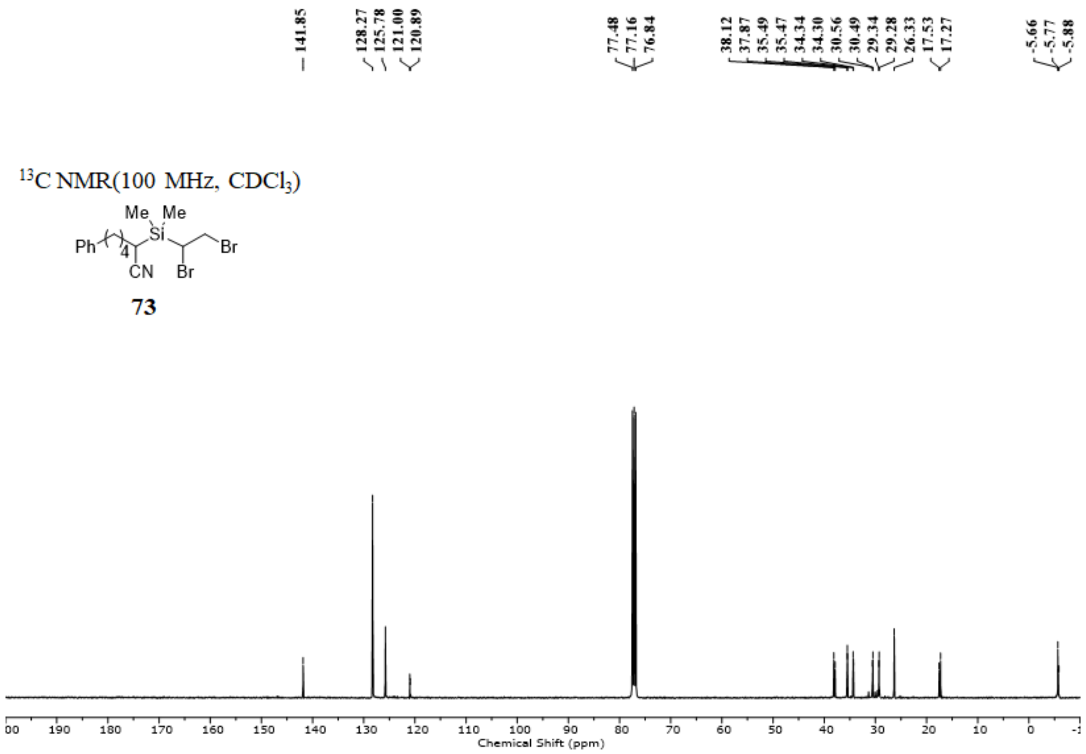

Supplementary Fig. 262. ^{13}C NMR of compound 73. The sample has been recorded in 100 MHz, $CDCl_3$ at 25 °C

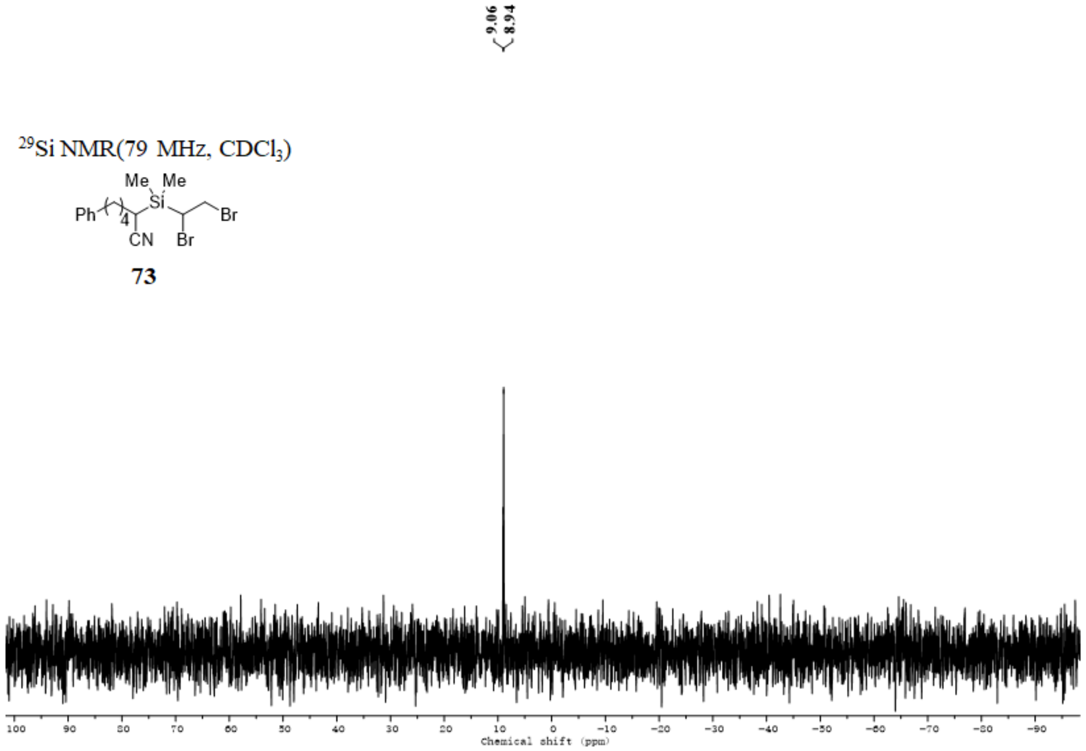

Supplementary Fig. 263. ^{29}Si NMR of compound 73. The sample has been recorded in 79 MHz, $CDCl_3$ at 25 °C

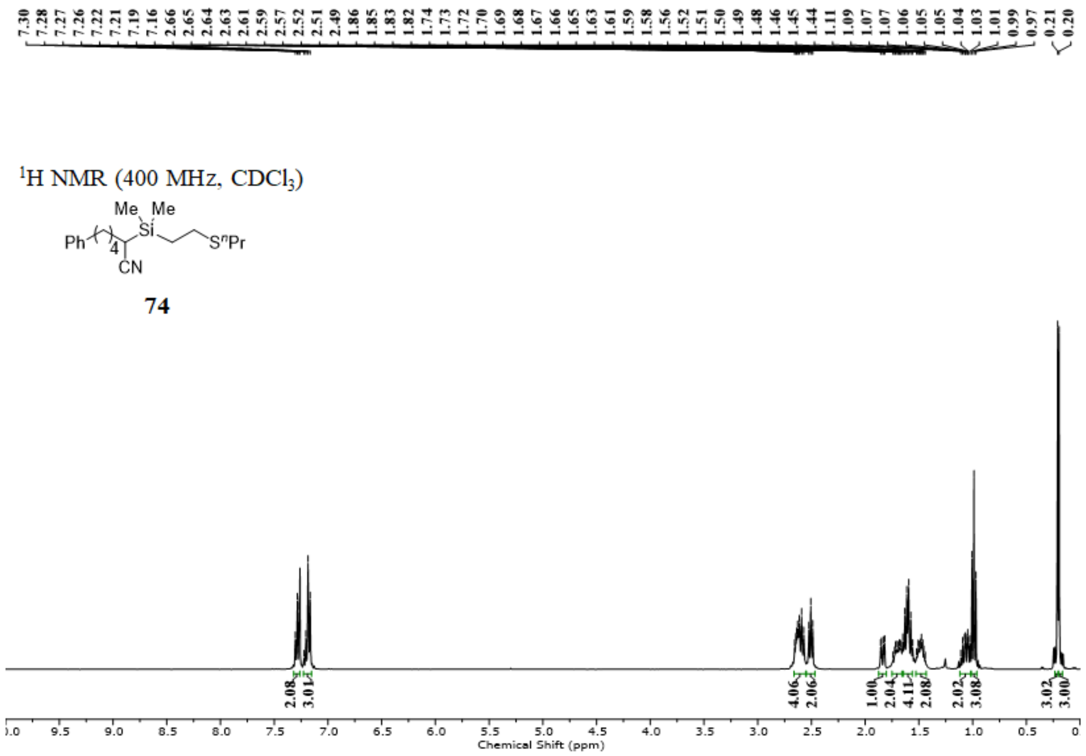

Supplementary Fig. 264. ^{1}H NMR of compound 74. The sample has been recorded in 400 MHz, $CDCl_3$ at 25 °C

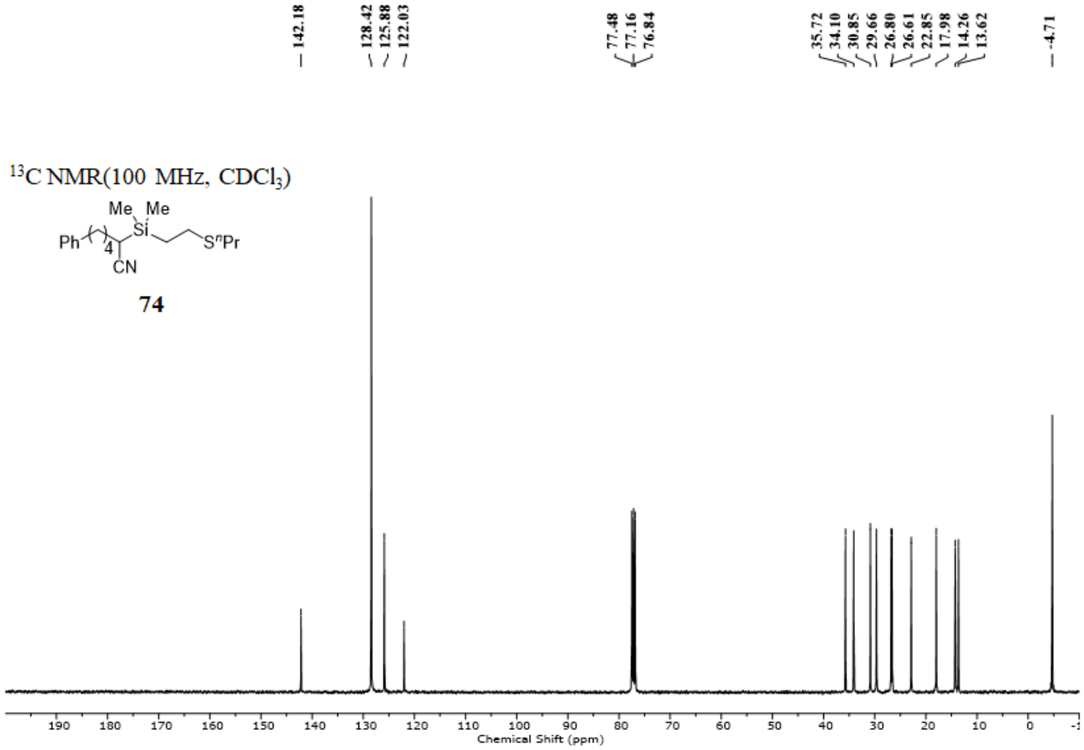

Supplementary Fig. 265. ^{13}C NMR of compound 74. The sample has been recorded in 100 MHz, $CDCl_3$ at 25 °C

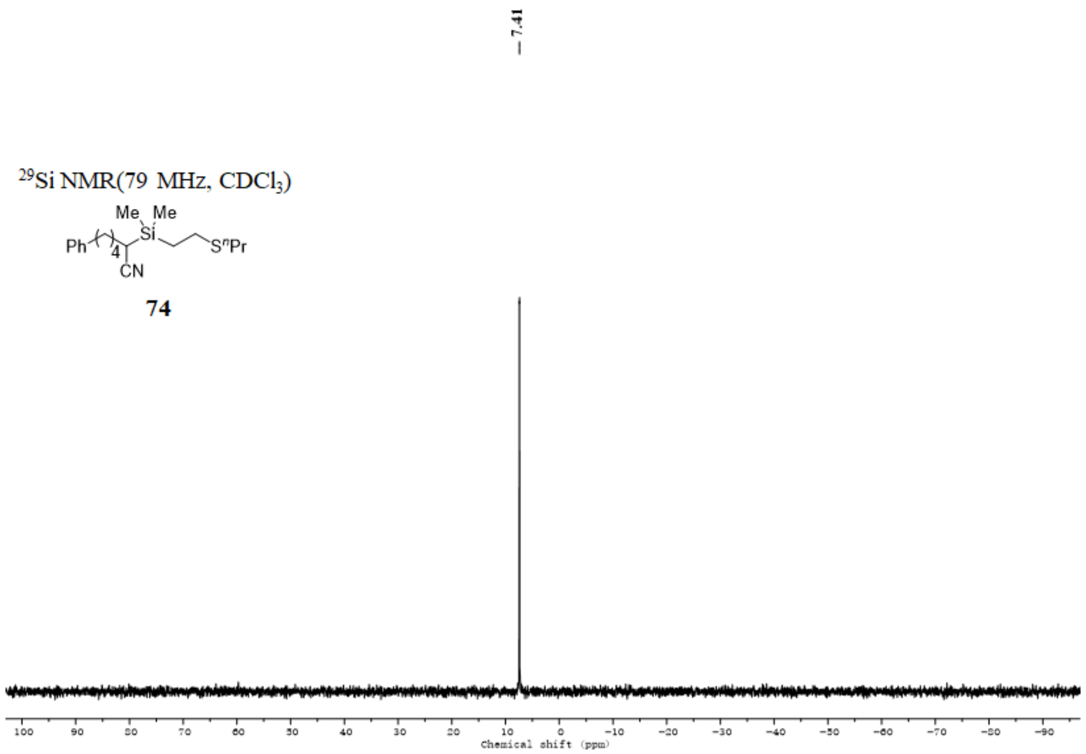

Supplementary Fig. 266. ^{29}Si NMR of compound 74. The sample has been recorded in 79 MHz, $CDCl_3$ at 25 °C

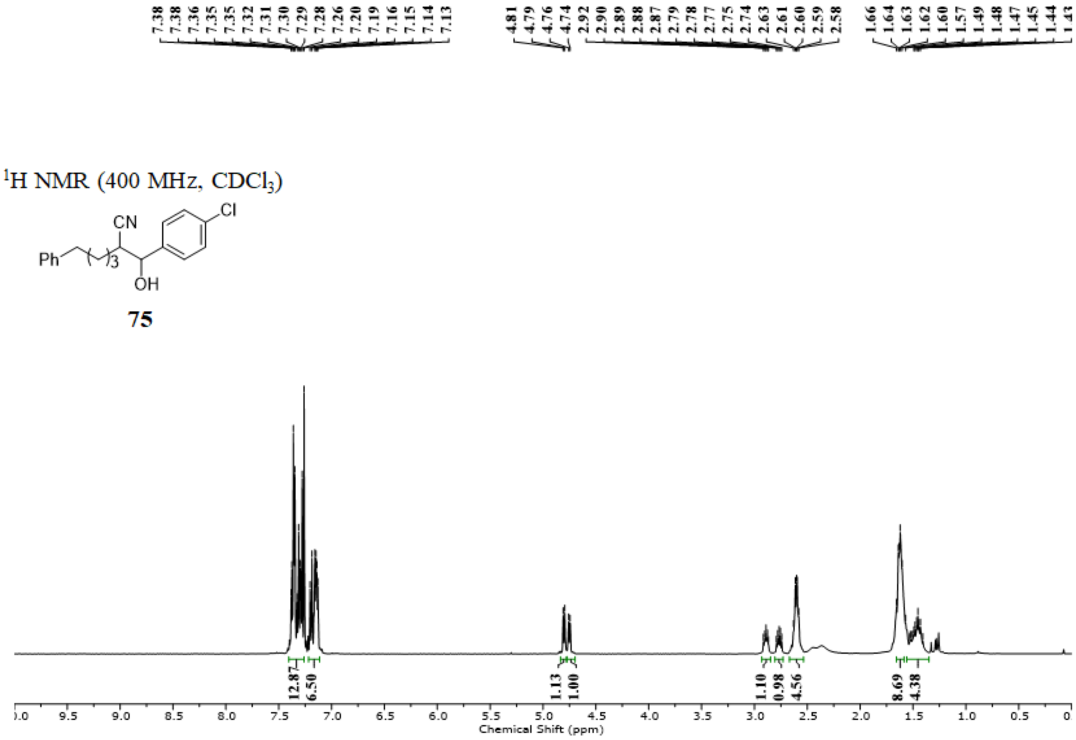

Supplementary Fig. 267. ^{1}H NMR of compound 75. The sample has been recorded in 400 MHz, $CDCl_3$ at 25 °C

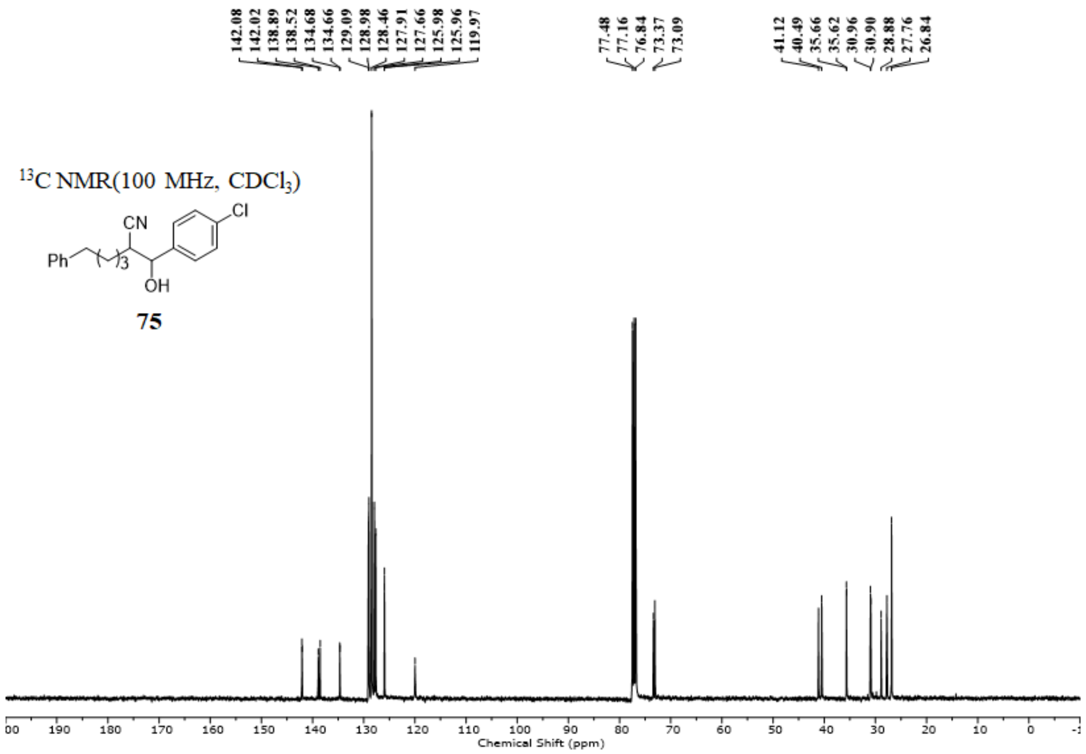

Supplementary Fig. 268. ^{13}C NMR of compound 75. The sample has been recorded in 100 MHz, $CDCl_3$ at 25 °C

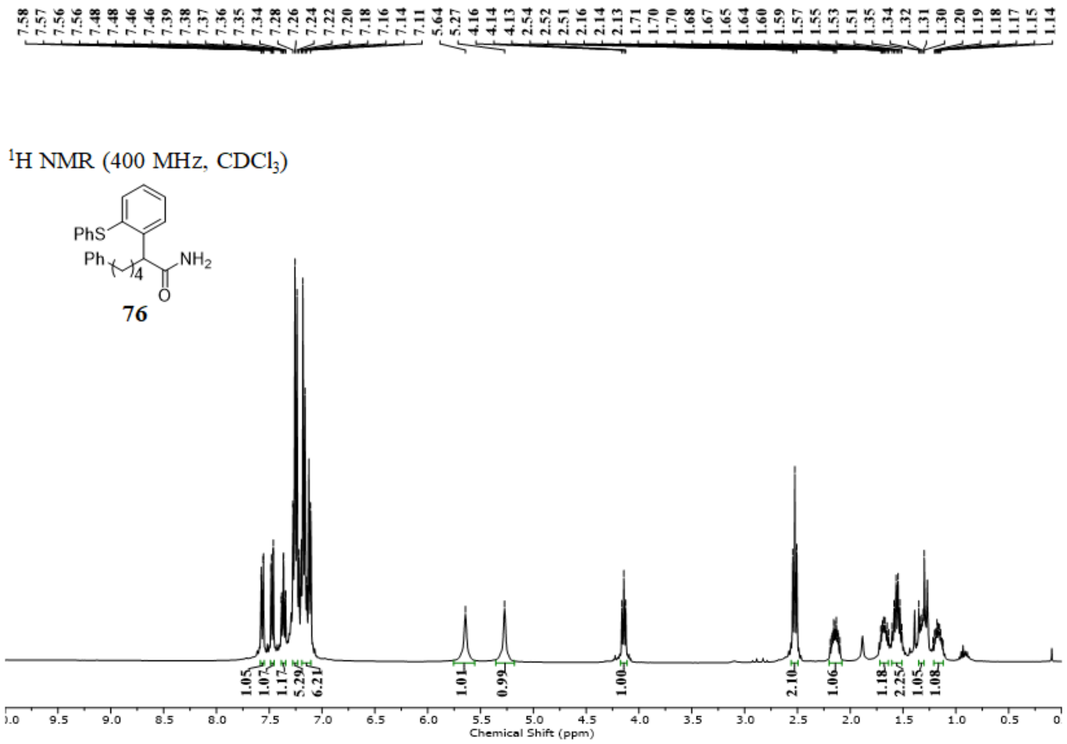

Supplementary Fig. 269. ^{1}H NMR of compound 76. The sample has been recorded in 400 MHz, $CDCl_3$ at 25 °C

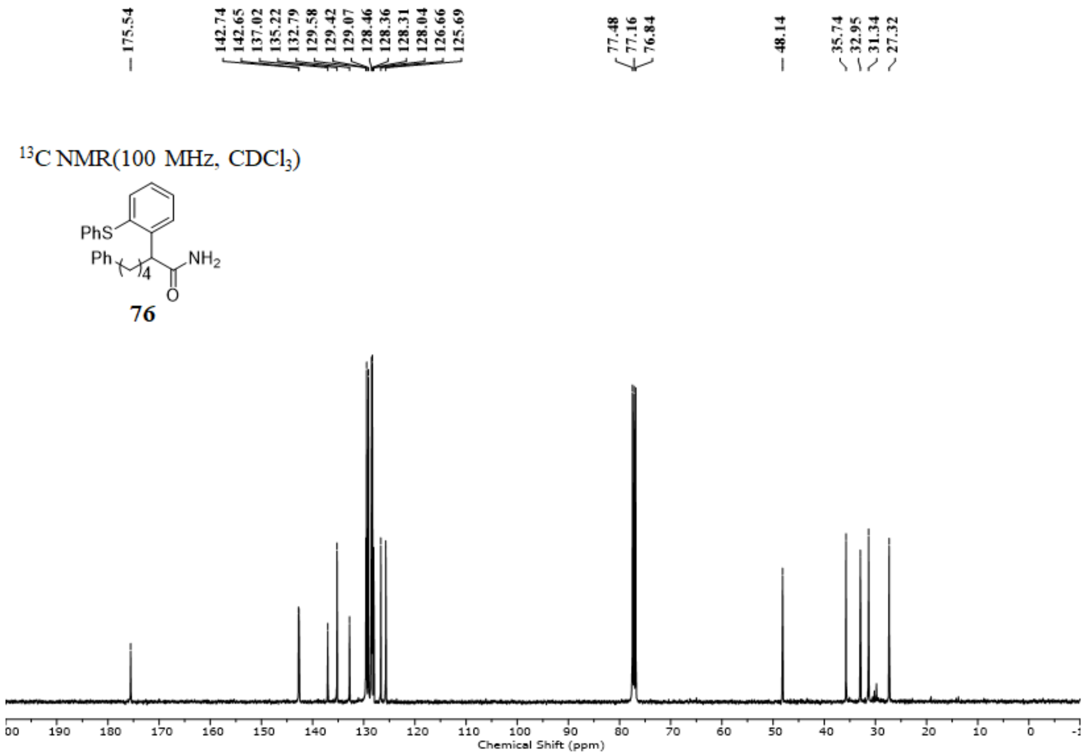

Supplementary Fig. 270. ^{13}C NMR of compound 76. The sample has been recorded in 100 MHz, $CDCl_3$ at 25 °C

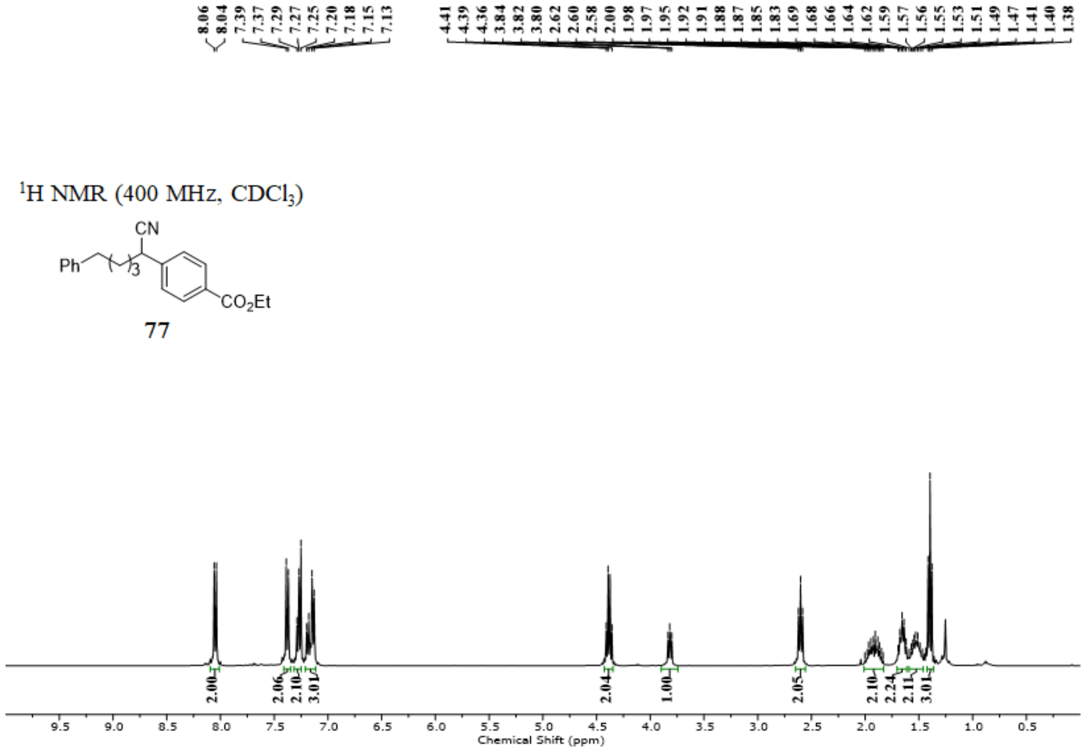

Supplementary Fig. 271. ^{1}H NMR of compound 77. The sample has been recorded in 400 MHz, $CDCl_3$ at 25 °C

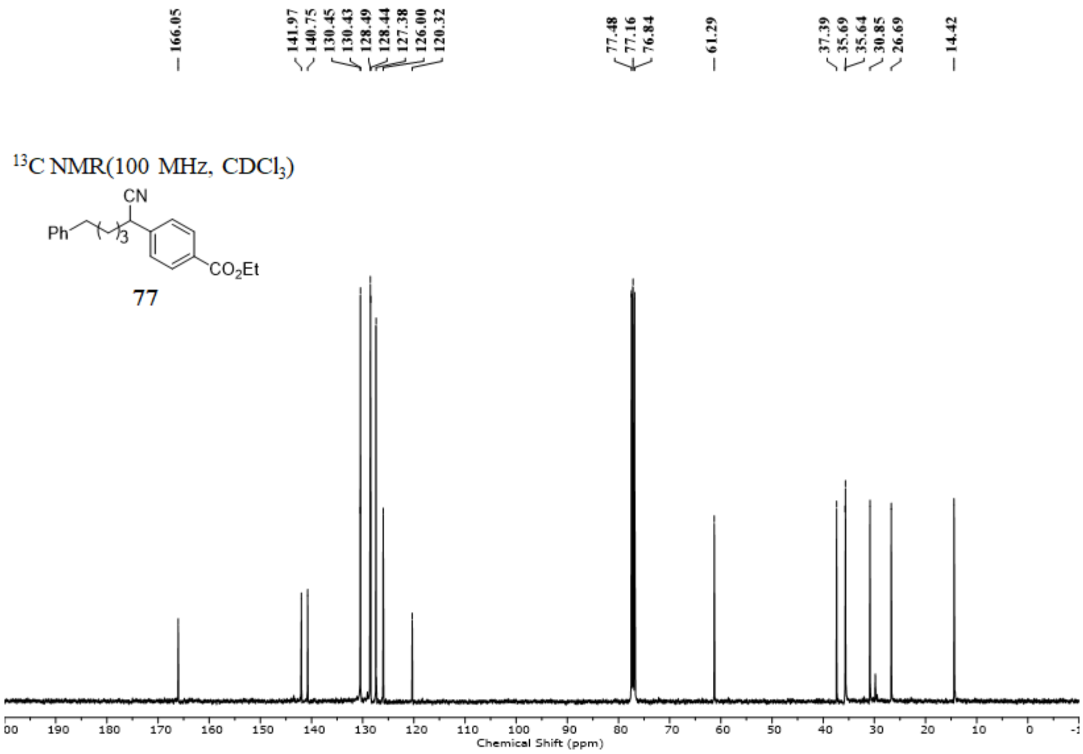

Supplementary Fig. 272. ^{13}C NMR of compound 77. The sample has been recorded in 100 MHz, $CDCl_3$ at 25 °C

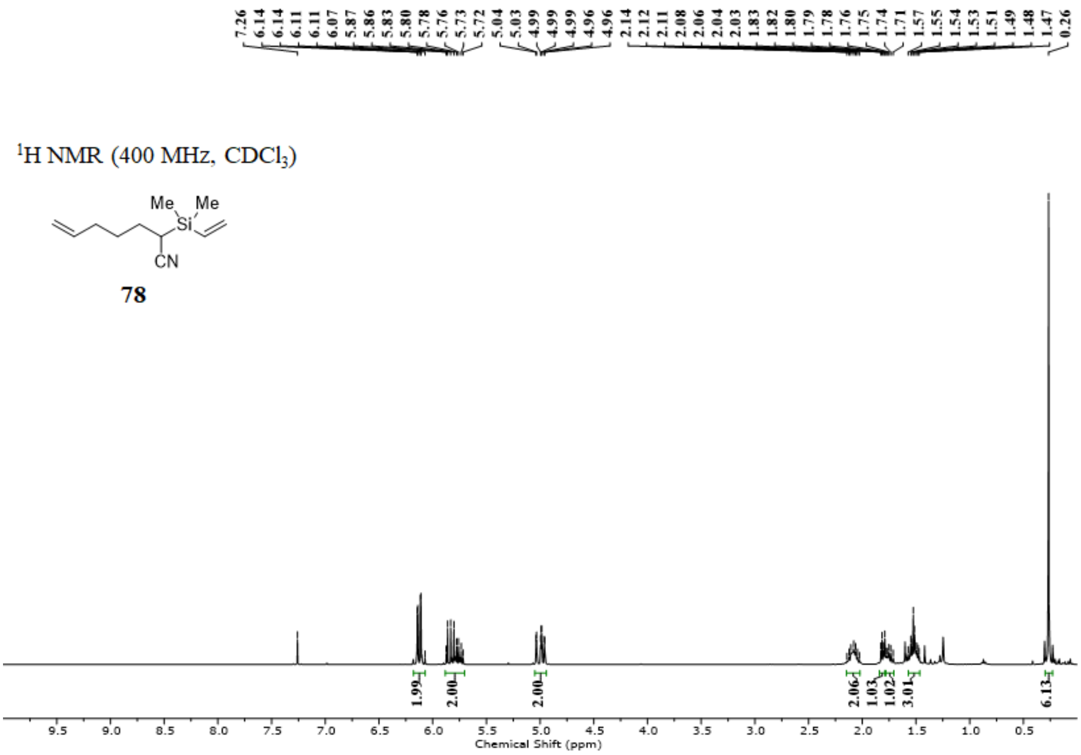

Supplementary Fig. 273. ^{1}H NMR of compound 78. The sample has been recorded in 400 MHz, $CDCl_3$ at 25 °C

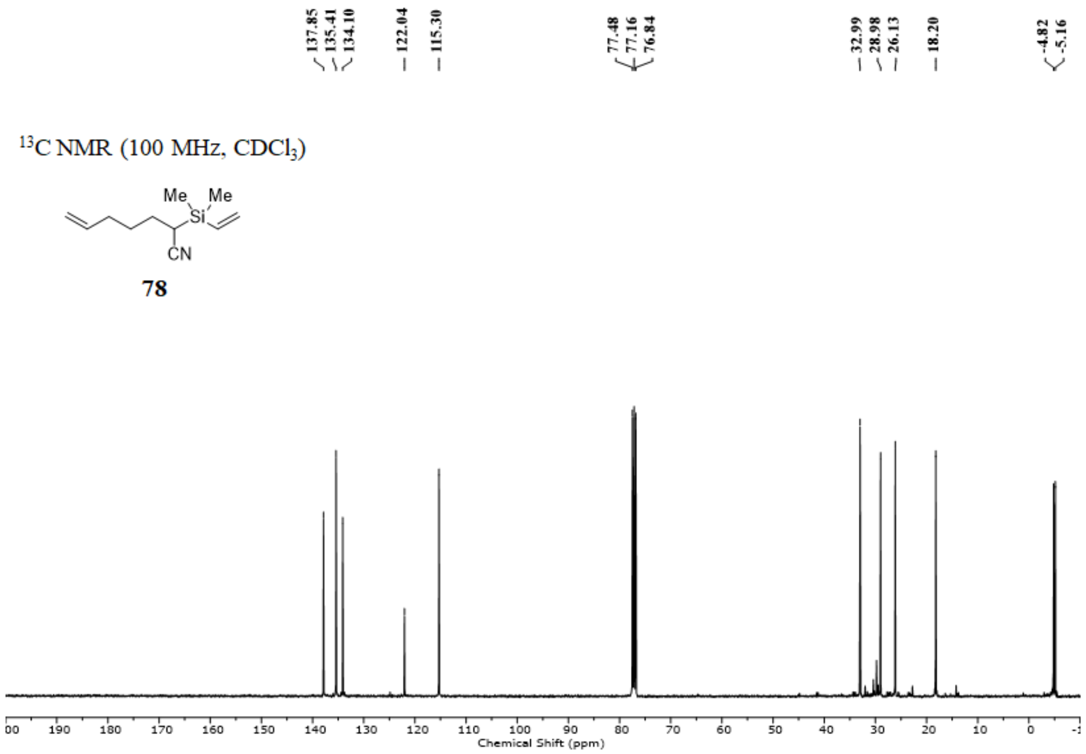

Supplementary Fig. 274. ^{13}C NMR of compound 78. The sample has been recorded in 100 MHz, $CDCl_3$ at 25 °C

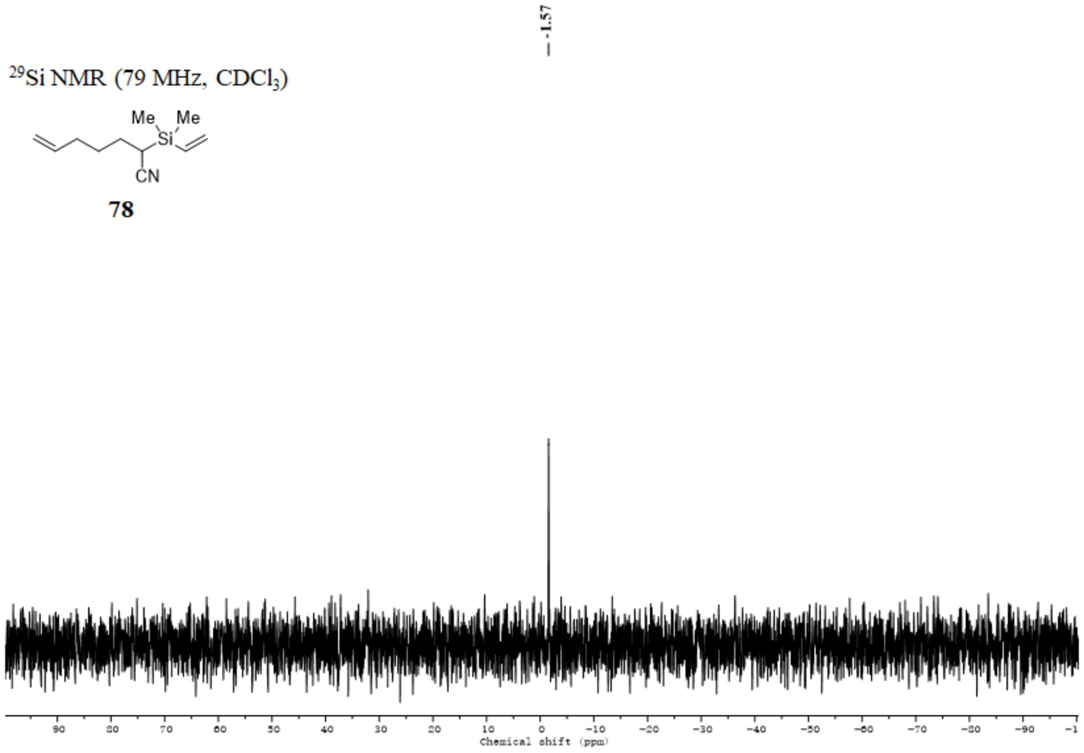

Supplementary Fig. 275. ^{29}Si NMR of compound 78. The sample has been recorded in 79 MHz, $CDCl_3$ at 25 °C

4. Supplementary References

1. Armarego, W. L. F. & Chai C. Purification of laboratory chemicals, 5th ed., (2003).
2. Pitts, A. K., O'Hara, F., Snell, R. H. & Gaunt, M. J. A concise and scalable strategy for the total synthesis of dictyodendrin B based on sequential C-H functionalization. *Angew. Chem. Int. Ed.* **54**, 5451-5455 (2015).
3. Lee, S. Y., Hong, Y. D., Kim, H. S. & Choi, S. J. Synthesis and application of a novel cysteine-based DTPA-NCS for targeted radioimmunotherapy. *Nucl. Med. Biol.* **40**, 424-429 (2013).
4. Yang, J., Song, H., Xiao, X., Wang, J. & Qin, Y. Biomimetic Approach to Perophoramidine and Communesin via an Intramolecular Cyclopropanation Reaction. *Org. Lett.* **8**, 2187-2190 (2006).
5. Mete, A., Bowers, K., Chevalier, E., Donald, D. K., Edwards, H., Escott, K. J., Ford, R., Grime, K., Millichip, I., Teobald, B. & Russell, V. The discovery of AZD9164, a novel muscarinic M3 antagonist. *Bioorg. Med. Chem. Lett.* **21**, 7440-7446 (2011).
6. Choubdar, N., Golshani, M., Jalili-Baleh, L., Nadri, H., Küçükkilinç, T.-T., Ayazgök, B., Moradic, A., Moghadame, F. H., Abdolahic, Z., Amerif, A., Salehianb, F., Foroumadi, A. & Khoobi, M. New classes of carbazoles as potential multi-functional anti-Alzheimer's agents. *Bioorg. Chem.* **91**, 103164-103173 (2019).
7. Jayasekara, P. S., Barrett, M. O., Ball, C. B., Brown, K. A., Hammes, E., Balasubramanian, R., Harden, T.-K. & Jacobson K. A. 4-Alkyloxyimino derivatives of uridine-5'-triphosphate: distal modification of potent agonists as a strategy for molecular probes of P2Y2, P2Y4, and P2Y6 receptors. *J. Med. Chem.* **57**, 3874-3883 (2014).
8. Pettit, G. R., Melody, N. & Chapuis, J. C. Antineoplastic Agents. 603. Quinstatins: Exceptional Cancer Cell Growth Inhibitors. *J. Nat. Prod.* **80**, 692-698 (2017).
9. Dai, C.-H., Narayanam, J. M. R. & Stephenson, C. R. J. Visible-light-mediated conversion of alcohols to halides. *Nat. Chem.* **3**, 140-145 (2011).
10. Yang, C.-T., Zhang, Z.-Q., Liang, J., Liu, J.-H., Lu, X.-Y., Chen, H.-H. & Liu, L. Copper-Catalyzed Cross-Coupling of Non-activated Secondary AlkylHalides and Tosylates with Secondary Alkyl Grignard Reagents. *J. Am. Chem. Soc.* **134**, 11124-11127 (2012).
11. Zhao, B., Shang, R., Wang, G.-Z., Wang, S.-H., Chen, H. & Fu, Y. Palladium-Catalyzed Dual Ligand-Enabled Alkylation of Silyl Enol Ether and Enamide under Irradiation: Scope, Mechanism, and Theoretical Elucidation of Hybrid Alkyl Pd(I)-Radical Species. *ACS Catal.* **10**, 1334-1343 (2019).
12. Tanemura, K. Silica gel-mediated hydrohalogenation of unactivated alkenes using hydrohalogenic acids under organic solvent-free conditions. *Tetrahedron Lett.* **59**, 4293-4298 (2018).
13. Ajvazi, N. & Stavber, S. Direct halogenation of alcohols with halosilanes under catalyst- and organic solvent-free reaction conditions. *Tetrahedron Lett.* **57**, 2430-

2433 (2016).

14. Wang, G.-Z., Shang, R., Cheng, W.-M. & Fu, Y. Irradiation-Induced Heck Reaction of Unactivated Alkyl Halides at Room Temperature. *J. Am. Chem. Soc.* **139**, 18307-18312 (2017).
15. Lutter, F. H., Grokenberger, L., Spieß, P.; Hammann, J. M., Karaghiosoff, K. & Knochel, P. Cobalt-Catalyzed Cross-Coupling of Functionalized Alkylzinc Reagents with (Hetero)Aryl Halides. *Angew. Chem. Int. Ed.* **59**, 5546-5550 (2020).
16. Wey, S. J., Augustyniak, M. E., Cochran, E. D., Ellis, J. L., Fang, X.-Q., Garvey, D. S., Janero, D. R., Letts, L. G., Martino, A. M., Melim, T. L., Murty, M. G., Richardson, S. K., Schroeder, J. D., Selig, W. M., Trocha, A. M., Wexler, R. S., Young, D. V., Zemtseva, I. S. & Zifcak B. M. Structure-Based Design, Synthesis, and Biological Evaluation of Indomethacin Derivatives as Cyclooxygenase-2 Inhibiting Nitric Oxide Donors. *J. Med. Chem.* **50**, 6367-6382 (2007).
17. Zhao, H.-W., Lu, C.-H., Herbert, S., Zhang, W. & Shen, Q.-L. Difluoromethylation of Alkyl Bromides and Iodides with $TMSCF_2H$. *J. Org. Chem.* **86**, 2854-2865 (2021).
18. Kim, D., Lee, G. S., Kim, D. & Hong, S.-H. Direct $C(sp^2)$-H alkylation of unactivated arenes enabled by photoinduced Pd catalysis. *Nat. Commun.* **11**, 5266 (2020).
19. Duan, J.-C., Wang, K., Xu, G.-L., Kang, S.-L., Qi, L.-L., Liu, X.-Y. Shu, X.-Z. Cross-Electrophile $C(sp^2)$-Si Coupling of Vinyl Chlorosilanes. *Angew. Chem. Int. Ed.* **59**, 23083-23088 (2020).
20. Zhang, L.-L. & Oestreich, M. Nickel-Catalyzed, Reductive $C(sp^3)$-Si Cross-Coupling of a-Cyano Alkyl Electrophiles and Chlorosilanes. *Angew. Chem. Int. Ed.* **60**, 18587-18590 (2021).
21. Duan, J.-C., Wang, Y.-Q., Qi, L.-L., Guo, P., Pang, X.-B. & Shu, X.-Z. Nickel-Catalyzed Cross-Electrophile $C(sp^3)$−Si Coupling of Unactivated Alkyl Bromides with Vinyl Chlorosilanes. *Org. Lett.* **23**, 7855-7859 (2021).
